# Self‐Regulation of Healthy Lifestyles in the Nursing Workplace: A Mixed‐Method Evaluation

**DOI:** 10.1155/jonm/2199578

**Published:** 2026-01-15

**Authors:** John Christopher Lambino Navarro, Siti Zubaidah Mordiffi, Sky Wei Chee Koh, Han Shi Jocelyn Chew

**Affiliations:** ^1^ Department of Nursing, Singapore General Hospital, Singapore, sgh.com.sg; ^2^ Department of Nursing, National University Hospital, Singapore, nuh.com.sg; ^3^ Department of Family Medicine, National University Polyclinics, Singapore; ^4^ Division of Family Medicine, Department of Medicine, Yong Loo Lin School of Medicine, National University of Singapore, Singapore, nus.edu.sg; ^5^ Alice Lee Centre for Nursing Studies, Yong Loo Lin School of Medicine, National University of Singapore, Singapore, nus.edu.sg

**Keywords:** health-promoting behaviours, healthy lifestyles, nurses, self-care, self-regulation, workplace environment

## Abstract

**Objective:**

To understand the experiences of self‐regulation and health‐promoting behaviours among practising nurses in Singapore.

**Qualitative:**

To explore the experiences of self‐regulating health‐promoting behaviours among nurses.

**Quantitative:**

To measure the nurses’ current health‐promoting behaviours and perceived stress levels.

**Background:**

Nursing is a physically and emotionally stressful occupation. While there is existing literature on the influencing factors of self‐care habits among nurses, little is known about the nurses’ self‐regulation facilitators and barriers of maintaining a healthier lifestyle.

**Methods:**

A concurrent mixed‐method study was conducted. Twenty‐four full‐time nurses from 15 Singapore healthcare institutions were recruited from August to November 2023 using purposive sampling. In‐depth interviews were conducted online through face‐to‐face virtual platforms using a semistructured interview guide. Thematic analysis was used to identify patterns and themes. Concurrently, 67 full‐time nurses completed questionnaires on their lifestyles and perceived stress. Data were analysed using descriptive statistics and limited statistical analysis and integrated with the qualitative study findings.

**Results:**

Five themes and 13 subthemes emerged. The five themes were (1) conflict between nursing and personal identity; (2) overwhelmed with time constraints; (3) power of intrinsic motivation over extrinsic incentives; (4) influence of close contacts at workplace on nurses’ lifestyle and (5) inadequate support for positive lifestyle change. The mean scores for the Health‐Promoting Lifestyle Profile II and Perceived Stress Scale‐10 were 2.39 (SD = 0.38) and 18.39 (SD = 4.58), respectively, indicating that most of the participants had poor lifestyles and were experiencing moderate stress. Additionally, there was a moderate statistically significant negative relationship between perceived stress and health‐promoting lifestyle behaviours (*ρ* = −0.461).

**Conclusion:**

Nurses’ healthy lifestyle challenges may stem from inadequate organisational support and low personal health prioritisation. Sustainable interventions should address workplace culture and work‐life integration to empower nurses to take ownership of their well‐being.

**Summary**

• What is already known?
◦ Nurses have poor self‐care habits, leading to adverse health outcomes.
◦ Quality of patient care is affected by nurses’ poor self‐care habits.
• What this paper adds?
◦ This study highlights nurses’ difficulty in self‐regulating their lifestyles due to the workplace culture and nurses’ tendency to prioritise their work over their own health.
◦ The self‐regulation perspective could provide a plausible explanation to how nurses’ health‐promoting behaviours are affected by the workplace.
◦ Explores nurses’ experiences on self‐regulation of health‐promoting behaviours, potentially providing direction on sustainable lifestyle interventions suited for nurses.

## 1. Introduction

Nurses play an essential role in promoting healthy behaviours such as healthy eating, engaging in moderate physical activity, refraining from tobacco use and stress management as part of their role in patient care [1, 2]. Despite promoting healthy behaviours to patients, nurses often struggle to prioritise their own health, leading to poor mental and physical wellbeing and increased risk of medication errors [3, 4] which threatens patient safety [5]. Ill health is also a common reason for the high absenteeism and poor retention rates among nurses, decreasing job satisfaction and exacerbating the long‐standing problem of nursing manpower shortage [6, 7]. Manpower shortage then triggers a perpetual cycle of absenteeism, increased pressure on the remaining staff, and eventual attrition [8].

Yet, studies have shown that nurses often lack the self‐regulation to adopt health‐promoting behaviours (HPB) and set good health behavioural examples to the public. This has led to an increased number of nurses with obesity, which reduces the public’s trust in their professional advice [9, 10]. Nurses were found to engage in lifestyle habits opposite to what they promote to patients, such as sedentary behaviour and unhealthy eating [11–14]. The prevalence of overweight and obesity among nurses was 31.2% and 16.3%, respectively, globally, with the Southeast Asia region having the highest obesity prevalence of 26.4% [15]. This phenomenon has been attributed to shift work, disrupting regular eating patterns [16], peer dietary influence [17] and reliance on substances such as tobacco to maintain wakefulness [18, 19]. More importantly, a study by Alkhawaldeh et al. [20] showed that 74% of nurses are faced with severe occupational stress, reducing the ability to self‐regulate behaviours and increasing the risk of developing physical and mental health disorders [21].

The interrelated terms ‘self‐regulation’, ‘self‐control’ and ‘self‐care’ refer to distinct concepts that collectively contribute to the integral element of human adaptive functioning. Self‐regulation refers to the all‐encompassing process whereby individuals monitor, evaluate and alter their thoughts, emotions and behaviours to enable effective pursuit of personal goals such as weight loss or external demands such as following rules [22]. As a subset of this process, self‐control refers to the capacity of individuals to overcome and inhibit impulsive thoughts and actions that will delay short‐term gratification for potential long‐term desirable outcomes [23]. In contrast, self‐care refers to the conscious engagement in sustainable behaviours that maintain or enhance the physical, emotional and psychological health of an individual [24, 25]. Self‐control is a component of self‐regulation that enables continued engagement of self‐care behaviours, especially when requiring effort to resist short‐term temptations that may interfere with these self‐care behaviours. Additionally, effective self‐care allows for the individual to replenish their psychological and physiological resources needed for continued self‐regulation and self‐control [26], creating a complementary relationship between these three terms.

**Figure 1:**
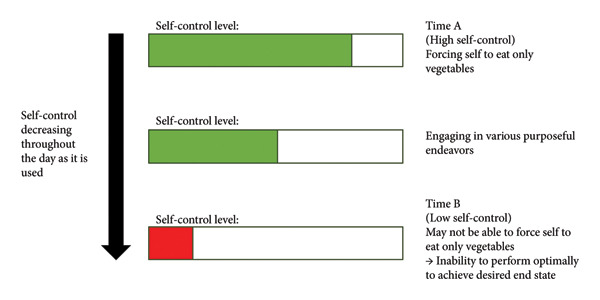
Simplified illustration to represent an example of ego depletion (based on the example given by Inzlicht et al. [22]).

While extensive research has brought about various self‐regulation theories and models, Baumeister and Vohs [27] strength model of self‐regulation (SMSR) remains the most well‐known model in social psychology and thus will be the primary model used in this paper. Its key element is the concept of ‘ego depletion’, which refers to the decreasing cognitive self‐control over thoughts, feelings and behaviours. This model postulates that self‐control is a finite resource that depletes with use and time [28]. We illustrate this model using Figure 1, with an example of an individual trying to adhere to a plant‐based diet. At time A, self‐control is exerted, and an individual is more likely to achieve his goal of maintaining his diet. However, self‐control wanes over time, and less self‐control can be exerted at time B compared to time A. After continually exercising self‐control, they may experience an ‘ego depletion’ state where they will have reduced control and decreased persistence in completing other tasks [29]. In a further study by Lin et al. [30], any tasks that require significant effort can contribute to the waning of self‐control, leading to this ego depletion state.

In a review by Englert [31] on exercise psychology utilising SMSR, self‐control strength can explain athletes’ lapses in exercise adherence and is a determining variable in whether an individual can successfully transform the intention to train into actual action and behaviour. Thus, he emphasised the need for more research on restoring and improving self‐control strength, akin to muscle strengthening through exercise, where frequent exertions can improve self‐control capacity [32].

While there have been other similar studies on self‐care behaviours of nurses [5], there has been limited research on HPB of nurses with a self‐regulation perspective. A study by Logan et al. [33] uses a similar self‐regulation perspective in relation with self‐care behaviours, reporting that work stress indirectly influences self‐care and self‐regulation. However, due to its quantitative nature, the study is limited to establishing the relationship between various factors, limiting its ability to answer questions on *why*. Thus, this current study will provide a more holistic and deeper understanding of the various factors at play by combining elements of both qualitative and quantitative elements via individual, in‐depth qualitative interviews and using a survey questionnaire to quantify measurable outcomes, respectively. The mixed‐methods nature of the study will also help in identifying overlooked but relevant facets of the topic [34]. To the best of the authors’ knowledge, no other mixed‐methods research on self‐regulation of HPB in nurses in a Singapore context has been done before.

Therefore, this study aims to explore nurses’ experiences on self‐regulating HPB and to quantify the current HPB and perceived stress levels of current nurses. It is crucial to gain an in‐depth understanding of self‐regulation of HPB to potentially improve health outcomes of nurses and retain the nursing workforce, which in turn could lead to increased quality of patient care. The research questions were (1) “What are nurses’ experiences on self‐regulating HPB?” and (2) “How does the workplace environment influence self‐regulation of HPB among nurses?”

## 2. Methods

### 2.1. Study Design, Setting and Participants

An inductive concurrent mixed‐methods study design was used for its ability to further the study’s analytical depth to derive deeper insights by substantiating the quantitative findings with qualitative results [35, 36]. Participant recruitment was conducted through social media (e.g., Facebook, Instagram and Telegram platforms) and convenience sampling (Supporting Information 1). Participants were recruited if they were (1) 21–65 years old; (2) full‐time nurses in a Singapore healthcare institution; (3) able to read and understand English and (4) able to provide informed consent. Participants were excluded if they have a health condition that disrupts one’s diet or physical activities. Before enrolment, participants were given a detailed explanation of the study and informed of the possibility of being invited for an online interview session. Once written informed consent was obtained, participants were invited to complete an online, self‐administered questionnaire on their sociodemographic profiles, HPB and perceived level of stress.

Afterwards, maximum differential sampling was used on the participants who completed the online questionnaire. With consideration to the sociodemographic profiles, a wide range of participants were selected based on their years of experience, position at work, current workplace, and their online questionnaire scores. This was to ensure a broad and meaningful range of perspectives were captured and participants who could provide rich and insightful information were selected.

An interview was conducted using a semistructured interview guide from August to November 2023. Online interview sessions were arranged according to the participants’ preferences. No relationship between participants and the researcher was established prior to the commencement of the interview and no monetary incentives were offered.

In 2023, there were 41,001 nurses working in Singapore’s public and private sectors. [37]. According to the Raosoft sample size calculator, 381 nurses were needed for an adequate representation of the population at 95% confidence, 5% marginal error and 50% response distribution. For the qualitative portion of the study, the sample size was determined based on data saturation.

Personal data including name and contact number were obtained for the sole purpose of recontacting the participants for an interview. Anonymity was maintained by assigning participants’ responses subject codes and pseudonyms. Ethics approval was obtained from the National University of Singapore Institutional Review Board (NUS‐IRB) (Reference Code: NUS‐IRB‐2023‐511).

### 2.2. Data Collection

Participants were required to complete an online questionnaire via a survey platform (Qualtrics). The questionnaire consisted of three components that asked them about their sociodemographic information, current health behaviours and perceived stress. Those involved in the qualitative portion of the study would then go through an interview session with the first author of the study.

#### 2.2.1. Quantitative Study

##### 2.2.1.1. Sociodemographic Profile

Information such as age, marital status, educational level and years of experience was collected (Supporting Information 4).

##### 2.2.1.2. HPB

HPB was measured using the 52‐item Health‐Promoting Lifestyle Profile II (HPLP‐II) [38], which categorized HPB into six dimensions namely interpersonal relationships (five questions), physical activity (seven questions) stress management (eight questions), nutrition (nine questions), spiritual growth (11 questions) and health responsibility (12 questions). Participants had to rate statements on their behaviours based on a four‐point Likert scale (4 = always, 3 = usually, 2 = sometimes and 1 = never). The maximum score range is between 52 and 208 (total average is 130 points or mean = 3). A poor adherence to HPB is indicated by a score of less than three, while a good lifestyle is indicated by above average score of more than or equal to three [39]. HPLP‐II remains to be extensively used around the world as a standard instrument for measuring HPB due to its high reliability and validity [40–42]. The Cronbach’s alpha coefficient for this portion of the questionnaire in this current study was 0.92, showing sufficient internal reliability.

##### 2.2.1.3. Perceived Stress

Perceived stress was estimated using the 10‐item Perceived Stress Scale (PSS‐10) [43]. Participants were asked to rate how frequently they experienced certain emotions within the last month on a five‐point Likert scale (0 = never and 4 = very often). A score of 0–13 indicates low stress, 14 to 26 indicates moderate stress, while 27 to 40 indicates high stress. Likewise to HPLP‐II, PSS‐10 is a widely used instrument to measure perceived stress around the world [44, 45]. The convergent validity with the Perceived Devaluation and Discrimination scale and Generalised Anxiety Disorder scale was *r* = 0.07–0.16 and *r* = 0.14–0.62, respectively, showing positive correlation and confirming validity [46]. The Cronbach’s alpha for this questionnaire in this study was 0.72, also showing sufficient internal reliability.

#### 2.2.2. Qualitative Study

A semistructured interview guide (Supporting Information 2) was developed based on prior literature to answer two main research questions namely “What are nurses’ experiences on self‐regulating health‐promoting behaviours?” and “How does the workplace environment influence self‐regulation of health‐promoting behaviours among nurses?” [21, 47]. The first author (HSJC) conducted the online interviews via Zoom after undergoing training on qualitative interview techniques by a research professor with extensive experience on qualitative interview processes, (HSJC). Only the interviewer and the participant were present in the Zoom call. Interviews were video recorded upon obtaining consent, and a pilot test was conducted with the first interviewee (P1) (Supporting Information 3). As no change was needed after consultation with (HSJC), a senior nurse researcher, data from the first interview was included for analysis. Open‐ended questions such as “As a nurse, how do you feel about self‐regulating a healthy lifestyle?” and prompting questions such as “How did your lifestyle change after you started to work as a nurse?” were asked to address the research questions [48]. Observational notes such as nonverbal body language were recorded for further insight into participants’ emotions as they answered the questions [49]. Moreover, no repeat interviews were done, and transcripts were not returned to participants for correction, but participants were reminded that there is a possibility they will be further contacted if the researchers need to clarify further details of their answers from the interview.

### 2.3. Data Analysis

A total of three researchers were involved in the data analysis. Data collection and data analysis were conducted concurrently, primarily by the first author. Constant comparative analysis between interview transcripts was done simultaneously with data collection to identify areas that required further elaboration. The coding process was also validated by two other trained researchers independently to ensure the codes and themes generated were coherent. Clarifications of codes were discussed thoroughly and resolved through consensus.

The 6‐phase thematic analysis by Braun and Clarke [50] was adopted for data analysis. For the first phase, ‘Familiarization’, each transcript was repeatedly read by the first author to gain familiarity with the data and derive insights into meanings and repeated patterns across the data set. For the second step of generating codes, initial codes were systematically generated by identifying relevant and interesting data extracts. Codes with similar ideas were then collated into categories. In the next phase, ‘Generating themes’, the categories were eventually sorted into themes by repeatedly re‐reading, narrowing down the number of codes, and recategorising into identifiable conceptual themes. Followed by the fourth phase, the themes were reviewed, ensuring that the component codes are all coherent and suitable for the specific theme. Codes would be removed or reclassified into other themes according to their suitability. Following that, the fifth phase was where each theme and subtheme’s representative essence is identified and renamed accordingly, ensuring each one is distinct from one another (Supporting Information 5). Lastly, the sixth phase was essentially where the data will be presented narratively in a cohesive and succinct manner. Video‐ and audiotaped reported interviews were transcribed verbatim and supplemented with observational field notes to enhance contextualisation of the interviews.

Due to the low response rate in the quantitative portion, very limited statistical analysis was done. Correlation analysis using the Spearman correlation coefficient was conducted to examine the relationship between HPLP‐II and PSS‐10 scores (Supporting Information 6). Descriptive statistics, particularly the mean and standard deviation of the HPLP‐II and PSS‐10 scores, were done. These analyses were conducted using jamovi software [51]. These findings were then used to supplement the qualitative portion of the study. Joint display analysis was also used to integrate qualitative and quantitative findings.

### 2.4. Rigour

Credibility was enhanced by the in‐depth, prolonged interviews and accompanying observational notes on each participant. During the interviews, summarising the main points was done to ensure correct interpretations of their responses. Furthermore, member‐checking postinterview via telephone contact was also done to further ensure accurate interpretations [52]. Additionally, the use of standard, peer‐reviewed questionnaire such as HPLP‐II and PSS‐10 for the survey ensured validity was accounted for. Dependability was enhanced by the comprehensive, constantly updated audit trail including the interviewer’s reflexivity journal that was immediately updated during data collection and data analysis. For enhancing transferability, extensive details on participants’ characteristics and the context of the study were provided for readers to determine if it is applicable to their own interest area. Confirmability was enhanced by having a professor analyse the list of codes and themes alongside one other researcher, discussing and resolving any discrepancies that were identified. Moreover, the validity and reliability of the instruments used, namely, HPLP‐II and PSS‐10, were both sufficiently high. This study was reported according to A Checklist of Mixed Methods Elements in a Submission for Advancing the Methodology of Mixed Methods Research (Supporting Information 7) [53].

## 3. Results

### 3.1. Health Promoting Lifestyle and Perceived Stress Levels

Sixty‐seven nurses completed the quantitative survey. The majority of these participants were female, Chinese, single degree‐holder staff nurses (SNs) working in public hospitals with rotating shifts (Table 1). Table 2 illustrates the summary scores for the adapted HPLP‐II and PSS‐10 questionnaires. The mean HPLP‐II score is 2.39 (SD = 0.38), indicating inadequate adherence to HPB, while the PSS‐10 mean of 18.39 (SD = 4.58) suggests that most nurses in this study are experiencing moderate levels of perceived stress. Additionally, there was a moderate, statistically significant negative relationship between HPLP‐II and PSS‐10 scores (Supporting Information 6) (*p* < 0.001), indicating that the higher the perceived stress, the lower the engagement in HPB.

**Table 1:** HPLP‐II and PSS‐10 mean scores.

**Sociodemographic characteristics**	

Mean Age (SD)	31.6 (8.8)
Sex, *n* (%)	
Female	57 (85.1)
Male	10 (14.9)
Race, *n* (%)	
Chinese	50 (74.6)
Malay	7 (10.4)
Indian	3 (4.5)
Others	7 (10.4)
Religion, *n* (%)	
Buddhism	15 (22.4)
Taoism	3 (4.5)
Islam	8 (11.9)
Christianity	18 (26.9)
Hinduism	2 (3.0)
No religion	18 (26.9)
Others	3 (4.5)
Marital status, *n* (%)	
Married	16 (23.9)
Never married/single	50 (74.6)
Divorced/separated	1 (1.5)
Highest education level, *n* (%)	
Bachelor’s degree	53 (79.1)
Diploma	5 (7.5)
Nitec in nursing	1 (1.5)
Master’s degree	8 (11.9)
Current workplace, *n* (%)	
Private hospital	4 (6.0)
Public hospital	55 (82.1)
Community hospital	1 (1.5)
Polyclinic	5 (7.5)
Hospice	1 (1.5)
Others	1 (1.5)
Position at work, *n* (%)	
Enrolled nurse	1 (1.5)
Staff nurse	55 (82.1)
Nurse manager	2 (3.0)
Nurse educator	2 (3.0)
Others	7 (10.4)
Dependents outside of work, *n* (%)	
Dependent children	6 (9.0)
Aging/disabled family	13 (19.4)
No dependents	44 (65.7)
Others	4 (6.0)
Work hours, *n* (%)	
Morning only	4 (6.0)
Rotating/variable	49 (73.1)
Office hours/others	14 (20.9)

*Note:* Rotating/variable: While there are minute variances between each institution, this generally mean that they have 3 different working hours; morning, afternoon and night/overnight shifts. The shifts are as follows: morning shift: 0700–1600; afternoon shift: 1300–2100; night shift: 2030–0730. The roster which dictates what shift the nurse is working for a day is usually planned a month in advance by the nurse managers. Additionally, office hours typically refer to 0800–1700.

**Table 2:** HPLP‐II and PSS‐10 mean scores of the 67 participants.

	Frequency (*N* = 67)	Mean (SD)	Minimum	Maximum
Health Promoting Lifestyle Score (HPLP‐II)		2.39 (0.38)	1.44	3.38
Unfavourable	62 (92.5%)			
Favourable	5 (7.5%)			
Health Responsibility		2.04 (0.46)	1.22	3.22
Physical Activity		2.24 (0.61)	1.13	4.00
Nutrition		2.29 (0.45)	1.33	3.33
Spiritual Growth		2.61 (0.56)	1.11	3.89
Interpersonal Relations		2.80 (0.51)	1.56	4.00
Stress Management		2.31 (0.48)	1.25	3.63
Perceived Stress Scale score (PSS‐10)		18.39 (4.58)	9.00	29.00
Low (0–13)	8 (11.9%)			
Moderate (14–26)	54 (80.6%)			
High (27–40)	5 (7.5%)			

*Note:* For HPLP‐II, the mean of the total average is 3. Unfavourable lifestyle is indicated by < 3, while favourable lifestyle is indicated by more than or equal to 3.

With reference to the HPLP‐II scores, the average scores of each dimension from highest to lowest were interpersonal relations, spiritual growth, stress management, nutrition, physical activity, and health responsibility. ‘Health responsibility’ domain which is defined as an active sense in taking accountability of one’s own health is scored the lowest. While the domain of ‘Interpersonal relations’ which is defined as communicating with others through verbal and nonverbal means to form meaningful relationships with others is scored the highest.

### 3.2. Influencing Factors of Self‐Regulating Health Promoting Lifestyle

Out of 35 nurses invited, 11 declined participation due to unavailability or simply not being keen for the interview. Data saturation was reached at the 20^th^ participant, where no new information was derived. Four more interviews were conducted after data saturation to ensure no new findings emerged. Ultimately, a total of 24 participants were interviewed; each interview lasted 32–111 min. This prolonged engagement with the participants allowed for rapport to be built and a greater breadth of responses [54].

The mean age of the interviewed participants was 28 years of with an average of 5.6 years working experience. The majority were working in public hospitals as a SN with rotating shifts, had a bachelor’s degree, were unmarried and had no dependents outside of work (Table 3). Five themes with 13 subthemes were identified to address the two research questions (Table 4).

**Table 3:** Sociodemographic profile of the 24 interview participants with pseudonyms (P1–P24) collected using the questionnaire.

Participant pseudonym	Age (years)	Years of experience	Gender	Race	Marital status	Religion	Highest education level	Current workplace	Position at work	Dependents outside of work?	Work hours?
P1	26	3	F	Chinese	Single	No religion	Degree	Public hospital	Staff nurse	Nil	Rotating/variable
P2	24	5	F	Malay	Single	Islam	Diploma	Public hospital	Staff nurse	Nil	Rotating/variable
P3	62	40	M	Indian	Single	Hinduism	Degree	Public hospital	Nurse educator	Nil	Other arrangements (0800–1730)
P4	25	1	F	Chinese	Single	No religion	Degree	Public hospital	Staff nurse	Nil	Rotating/variable
P5	24	1	F	Chinese	Single	Buddhism	Degree	Public hospital	Staff nurse	Nil	Rotating/variable
P6	38	18	F	Chinese	Married	Christianity	Degree	Public hospital	Nurse clinician	1 child	Morning only
P7	25	1	F	Chinese	Single	Buddhism	Degree	Public hospital	Staff nurse	Aging/sick family members	Rotating/variable
P8	33	9	M	Chinese	Divorced	No religion	Masters	Private hospital	Nurse clinician	Nil	Rotating/variable
P9	27	4	F	Chinese	Single	No religion	Degree	Private hospital	Staff nurse	Nil	Rotating/variable
P10	30	5	F	Others	Single	No religion	Degree	Polyclinic	Staff nurse	Aging/sick family members	Other arrangements (office hours: 0800 to 1700, Mon–Fri)
P11	25	1	F	Chinese	Single	Christianity	Degree	Public hospital	Staff nurse	Nil	Rotating/variable
P12	30	7	F	Chinese	Single	No religion	Degree	Public hospital	Nurse clinician	1 child	Rotating/variable
P13	26	3	F	Chinese	Single	Christianity	Degree	Public hospital	Staff nurse	Nil	Rotating/variable
P14	27	3	F	Others	Single	Christianity	Degree	Public hospital	Staff nurse	Nil	Rotating/variable
P15	23	1	F	Chinese	Single	Buddhism	Degree	Public hospital	Staff nurse	Nil	Rotating/variable
P16	25	2	F	Chinese	Single	Christianity	Degree	Public hospital	Staff nurse	Nil	Rotating/variable
P17	26	3	F	Chinese	Single	Christianity	Degree	Public hospital	Staff nurse	Nil	Rotating/variable
P18	25	1	M	Chinese	Single	Islam	Degree	Public hospital	Staff nurse	Aging/sick family members	Rotating/variable
P19	24	2	F	Others	Single	Islam	Degree	Public hospital	Staff nurse	Aging/sick family members	Rotating/variable
P20	25	4	M	Chinese	Single	No religion	Diploma	Public hospital	Staff nurse	Nil	Rotating/variable
P21	27	2	M	Chinese	Single	No religion	Degree	Public hospital	Staff nurse	Nil	Rotating/variable
P22	24	2	F	Chinese	Single	Buddhism	Degree	Public hospital	Staff nurse	Nil	Rotating/variable
P23	26	3	F	Chinese	Single	No religion	Degree	Public hospital	Staff nurse	Nil	Rotating/variable
P24	36	13	F	Chinese	Single	Buddhism	Masters	Public hospital	Nurse clinician	Aging/sick family members	Other arrangements (office hours: 0800–1700, Mon–Fri)

*Note:* Rotating/variable: While there are minute variances between each institution, this generally mean that they have 3 different working hours; morning, afternoon and night/overnight shifts. The shifts are as follows: morning shift: 0700–1600; afternoon shift: 1300–2100; night shift: 2030–0730. The roster which dictates what shift the nurse is working for a particularly day is usually planned a month in advance by the nurse managers. Additionally, office hours are typically 0800–1700.

**Table 4:** Themes and subthemes that emerged in relation to the research questions.

Research questions	Themes	Subthemes
What are nurses’ experiences on self‐regulating health‐promoting behaviours?	Conflict between nursing and personal identity	• Importance of taking care of oneself holistically
		• Nurses’ tendency to go beyond at work
		• Pursuing personal interests outside of nursing
	Overwhelmed with time constraints	• Shift work
		• Time as a limited resource
		• Inadequate rest times
	Power of intrinsic motivation over extrinsic incentives	• Consideration of individual’s own condition
		• Maintenance of sustainable lifestyle change is determined by intrinsic factors

How does the workplace environment influence self‐regulation of health‐promoting behaviours among nurses?	Influence of close contacts at workplace on nurses’ lifestyle	• Nurse managers’ role in influencing lifestyle of nurses
		• Emulating lifestyle of close contacts
	Inadequate support for positive lifestyle change	• Difficulty in self‐regulating a healthy lifestyle as a nurse
		• Ineffective current measures
		• Workplace culture establishes a new baseline lifestyle for nurses

#### 3.2.1. Conflict Between Nursing and Personal Identity

Nurses have a “self‐sacrificial mindset”; “to go above and beyond” at work for their patients at the expense of their own health. However, many of these nurses also have a desire for work‐life balance and to pursue their own interests. These personal goals are then in conflict with their professional identity of a ‘nurse’ which values patient‐centredness.

##### 3.2.1.1. Importance of Taking Care of One’s Self Holistically

Almost all nurses shared that their definition of a healthy lifestyle is that it is multifaceted and work‐life balance is a must.

“I feel like a healthy lifestyle would be a balanced lifestyle, a good balance between work between personal life and between rest. Yeah, that′s what I’d define a healthy lifestyle.” (P14, 27‐year‐old staff nurse [SN])

##### 3.2.1.2. Nurses’ Tendency to Go Beyond at Work

Many nurses expressed their tendency to overprioritise work, indicating that their self‐control resources are often depleted by work demands, leaving insufficient capacity to engage in HPB.

“When nurses right now are spending like all their time and effort to just take care of their patients and do the best at their job. They′re not going to have a lot of time and effort left for themselves.” (P18, 25‐year‐old SN)

##### 3.2.1.3. Pursuing Personal Interests Outside of Nursing

Subsequently, the majority of nurses also verbalised their desire to maintain an identity outside of the workplace by engaging in their personal interests. This suggests that investing time in self‐care and personal development can enhance, rather than compromise, patient care.

“You, yourself have to know that you have to fall in love with yourself. Once you love yourself, you can share the joy and the love with others. So, if you don′t love yourself, you have your own concerns, then you cannot do, cannot do a good compassionate, passionate nursing.” (P3, 62 year‐old Nurse educator)

#### 3.2.2. Overwhelmed With Time Constraints

Most nurses expressed difficulty in engaging in self‐regulation activities due to having to juggle multiple commitments with their extremely limited personal time. Additionally, the majority of them revealed that they are too exhausted after a shift to spend their time productively.

##### 3.2.2.1. Shift Work

Shift work is a major barrier to self‐regulation due to the extra layer of difficulty it introduces in scheduling activities outside of work, hence impacting their self‐regulation of HPB. Also, nurses are reluctant to ask for adjustments to their shift work schedule, as they do not want to be an inconvenience to others.

“…shift work is not regular, right? … it’s quite a lot of trouble, because, first of all, I have to talk to my Sister right? Then again, how do I ensure that she definitely will grant my request? Right? So that is a consideration, because otherwise I cannot sign up for something and pay for something that I know that I cannot attend regularly.” (P9, 27 year‐old, SN)

##### 3.2.2.2. Time as a Limited Resource

Nurses shared that their personal time is already divided into various outside commitments, underlining the difficulty in allocating sufficient time for self‐regulation. With various commitments, time for oneself is usually a luxury that many cannot afford.

“So I think I struggled a lot with like planning my time. Like, when do I have like my self‐care days? When can I like slot in a workout? When can I go out with friends, or spend time with family, for example?” (P17, 26 year‐old SN)

##### 3.2.2.3. Inadequate Rest Times

Almost all the nurses mentioned postshift fatigue as a hindrance to self‐regulation, as they are simply too exhausted to engage in HPB outside of work. Perhaps due to inadequate rest time, many nurses remain in an ego‐depleted state.

“…The demands of my work was a bit exhausting, so I don′t want to do anything, yeah, anymore. I don′t even want to cook. I don′t even want to move. Sometimes I would just come home, sit on a sofa still in my uniform until like seven or eight plus.” (P10, 30 year‐old SN)

#### 3.2.3. Power of Intrinsic Motivation Over Extrinsic Incentives

Many nurses share the perception that intrinsic factors, such as self‐responsibility, are greater determining factors for sustainable HPB compared to extrinsic factors.

##### 3.2.3.1. Consideration of Individual’s Own Condition

Participants shared that for an individual to have the impetus to change their lifestyle, they will have to be cognisant of their own body’s condition and be intrinsically motivated to change it, either to avoid chronic conditions or for aesthetic reasons.

“…And prior to this I have never fallen sick this often. So that was a big, a wake up call that I should at least get my health back together. Because yeah, falling sick‐ It′s really the long‐term effects of it is really not good. I don′t want to like grow old and have a bunch of different illnesses, just because I didn′t take care of myself when I was younger.” (P19, 24 year‐old SN)

##### 3.2.3.2. Maintenance of Sustainable Lifestyle Change Is Determined by Intrinsic Factors

Nurses shared that being intrinsically motivated, such as having self‐discipline, is ultimately the most important factor to maintain sustainable healthy lifestyle changes.

“I think in the end, the decisions, the health lifestyle decisions that we need is based on ourself.” (P7, 25 year‐old SN) Ultimately, intrinsic motivation allows for oneself to push themselves beyond the ego depletion state.

#### 3.2.4. Influence of Close Contacts at Workplace on Nurses’ Lifestyle

Most nurses felt that social aspect of the workplace was a major facilitator of self‐regulation of healthy lifestyles. The close contacts in the workplace shape the kind of lifestyle that nurses would emulate.

##### 3.2.4.1. Nurse Managers Role in Influencing Lifestyle of Nurses

Most nurses expressed that the nurse managers’ role is to facilitate self‐regulation either through fair rostering or by being a role model for the nurses to emulate, hence indicating nurse managers as major influencing factors that can affect nurses’ ability to self‐regulate HPB effectively.

“I think most managers, they do play an important role. For instance, I think the main role of nurse managers in facilitating their staff′s health is to number one, ensure that the staff get sufficient time and sufficient rest off because the main responsibility of nurse managers is to, hmm, organize the roster.” (P13, 26 year‐old SN)

##### 3.2.4.2. Emulating Lifestyle of Close Contacts

Many nurses express the influence of their colleagues on their lifestyles. Consequently, the patients serve as a real‐life example of what could potentially happen if they continued with their poor lifestyle habits, encouraging many to engage in more self‐care.

“…there are times that they [Colleagues] will ask you out for lunch, and they choose the not so healthy diet, and in order to, you know, sometimes to linger with them, you will also join them.” (P6, 38 year‐old Nurse clinician)

#### 3.2.5. Inadequate Support for Positive Lifestyle Change

Almost all the nurses have mentioned that while there were active attempts, there is currently inadequate support for nurses’ self‐regulation. Therefore, many of the nurses expressed difficulty in self‐regulating due to a myriad of organizational issues.

##### 3.2.5.1. Difficulty in Self‐Regulating a Healthy Lifestyle as a Nurse

The high‐stress, high‐workload nature of the nursing occupation, coupled with an unconducive workplace environment, makes it extremely difficult to engage in HPB, contributing to unhealthy lifestyle choices.

“Even on break we are still doing work. Like the stress is always constantly running through which isn’t good.” (P21, 27 year‐old SN).

##### 3.2.5.2. Ineffective Current Measures

The majority of the nurses felt that their institution’s existing efforts to promote healthy living in nurses were largely inadequate. Perhaps, the current measures do not effectively aid in restoring one from an ego‐depleted state.

“I don’t think the institution is doing a lot to encourage healthier lifestyles. But maybe they are, but I’m not aware…” (P23, 26 year‐old SN)

##### 3.2.5.3. Workplace Culture Establishes a New Baseline Lifestyle for Nurses

The workplace culture is often cited as a positive or negative influencing factor that can aid or hinder their attempts to maintain a positive lifestyle, respectively.

“…Because I′ve been in some, like other attachments where, like the culture, wasn′t quite as friendly. And then, in that sense, it really detracts against self‐regulating, because even if you have the time to take a break. Sometimes you don′t. Because you′re worried about how other people will perceive you and like whether that will get you a complain…” (P16, 25 year‐old SN)

## 4. Discussion

**Figure 2:**
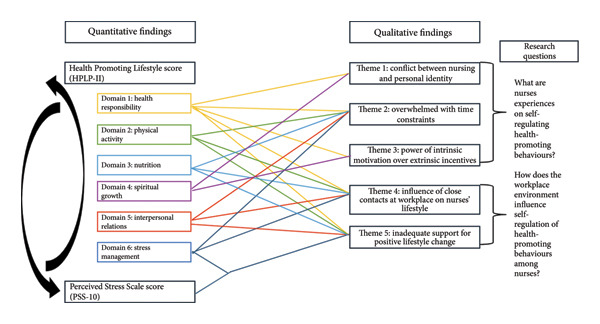
Convergence of the quantitative and qualitative results.

This study delved into Singaporean nurses’ experiences on self‐regulation of HPB and how the workplace environment impacts these self‐regulation habits. Our quantitative analyses revealed that higher stress levels impair HPB. It also provided us an overview of the situation, as it highlighted that nurses have poor health‐promoting lifestyles and are experiencing moderate stress in general. Our qualitative findings identified factors that impact nurses’ HPB and how the nursing workplace is a major influencing factor. Utilising joint display analysis (Figure 2), explanatory connections can be derived from the two data sets, providing a more robust understanding together, allowing for more insights to be expanded upon [55]. The colour‐coded lines in Figure 2 represent the relationship between the quantitative results and the qualitative themes, showcasing how the different domains of a health‐promoting lifestyle are affected, emphasising the need to address it holistically. This also showcases that the ‘Health responsibility’ domain is ever‐present in all the themes despite ranking the lowest score among all the HPLP‐II domains, indicating that while the workplace does indeed influence various aspects of self‐regulating a healthy lifestyle, cultivating a stronger sense of self‐responsibility for one’s health may ultimately be the main factor for effective self‐regulation.

### 4.1. Workplace Environment’s Influence on Lifestyle

Together, the quantitative and qualitative findings revealed that nurses’ struggle to self‐regulate their lifestyle behaviours is tied to the workplace. A recurring theme among many of the participants was the inadequate organisational support and the current measures in place to help manage the workplace stress, which are largely ineffective, leading to impaired self‐regulation. This is supported by the high proportion of respondents indicating moderate to high PSS‐10 scores (mean score of 18.39) and the HPLP‐II’s ‘Stress Management’ domain mean score of only 2.31 (SD = 0.48). Our study remains consistent with previous literature which also expounded that high work stress present in the highly demanding nursing workplace can negatively affect the psychological well‐being of nurses, leading to poorer self‐care and self‐regulation [33]. Aligning with Baumeister et al. [32] SMSR, the findings in this study reflect the idea that self‐control is a limited resource that is continually depleted with time and effort. Given the stressful nature of the nurses’ workplace environment, this depletion of self‐control occurs at an accelerated rate due to more cognitive effort being expended, resulting in mental and physical fatigue, and ultimately contributing to poorer self‐regulation of HPB among nurses. Hence, with self‐regulation negatively affected by stress as shown in our correlation analysis of HPLP‐II and PSS‐10 scores, self‐care is ineffective, leading to a continued state of low self‐control [26] or ‘ego depletion state’. This continued state of ‘diminished’ self‐control due to stress can then further explain previous literature on how the high‐stress workplace leads to negatively affecting the physical activity and dietary habits of nurses [17, 56] as they tend to forgo exercising to rest and engage in binge eating or unhealthy diets for short‐term satisfaction.

Fortunately, our qualitative findings also found that a positive workplace culture can positively impact the lifestyle of nurses. The domain of ‘Interpersonal relations’, which is defined as communicating with others through verbal and nonverbal means to foster meaningful relationships with others, has the highest score among the HPLP‐II domains, highlighting the significance of the social aspect of the workplace, as many of the nurses engage in adaptive coping through interpersonal connectedness. Nurses’ professional necessity to communicate effectively in clinical settings to facilitate patient care [57] is also often reflected in how they communicate in their personal lives, potentially explaining why this domain is high. Moreover, Singapore’s nursing workforce comprised a large proportion foreign nurse [58], who unlike their local counterparts, often lack the traditional family support common in Asian cultures [59]. With little social support outside of the workplace and the emphasis of teamwork in the ward setting, this has perhaps built a stronger bond between the local and foreign nurses, unwittingly resulting in an increased susceptibility to peer pressure. Many shared they were influenced by the unhealthy lifestyles of their peers to fit in with the majority and maintain the social dynamics of the workplace. Thus, comparable to other literature, the social culture of the workplace can be a key influencer in nurses’ lifestyles [60], affecting various aspects from physical activity and nutrition to even their stress management styles as they adopt habits and emulate their close peers.

### 4.2. Perceived Barriers and Facilitators

Our study findings were consistent with prior literature that many nurses attributed poor lifestyle habits such as unbalanced diets to the nature of shift work, which led to irregular meal timings and a lack of rest between shifts to relieve stress [5, 18, 61]. However, some nurses also mentioned that shift work facilitated easier scheduling of physical activities, as they had more flexibility with arranging activities during nonpeak timings, allowing them to engage in more exercise. This inconsistency could be explained similarly to the Forcada‐Parilla et al. [62] study, whereby because most of the nurses in the sample who had shift work arrangements were younger than 34 years old with fewer family responsibilities, they were more likely to engage in physical activity outside of work. This suggests that younger nurses may have more self‐regulation over their HPB, but nonetheless, shift work remains a major influencing factor on nurses’ lifestyles.

Despite only having the majority of the participants with a mean age of 31.6 (SD = 8.8), the physical activity domain of the HPLP‐II is the second lowest, indicating that maintaining physical fitness is not a priority for many of the nurses. Coupled with concerns over having limited personal time, this can explain why self‐care becomes less of a priority, hence not being able to replenish the ‘self‐control’ resource from an ‘ego depleted’ state. A workplace culture that emphasises organisational demands with little margin for error can inadvertently compromise staff well‐being, leading to excessive overtime and the blurring of the boundaries of work and personal life [5]. This phenomenon is now more common, as there is a global nursing shortage that has led to a greater strain on the existing nursing workforce and, consequently, lower quality of patient care [63]. Thus, these results imply that nurses are in a constant ‘ego depleted state’ as suggested by Baumeister et al.’s [32] SMSR; hence, the unwillingness to self‐regulate after work adversely affects their work performance.

However, another major theme is the power of intrinsic motivation over extrinsic incentives. According to Baumeister [64], strong motivation can temporarily counteract ego depletion, enabling nurses who are highly motivated to maintain their active lifestyles despite professional demands. This is further supported by our qualitative findings, where participants who actively engage in healthier diets and/or physical activity outside of work mention that maintaining their healthier lifestyle from their nursing student days is due to them cultivating this mindset when they were younger and simply maintaining it. These nurses also mention they have developed a habit of regular exercise as to overcome negative consequences of stress which is aligned with previous studies [65]. Nurses who have developed effective stress management habits are more likely to maintain their self‐regulation of HPB, which is supported by our quantitative findings whereby there is a negative correlation between stress and engagement with HPB. Thus, further highlighting the need to inculcate effective self‐care habits such as effective stress management to replenish self‐control and self‐regulation [26]. Additionally, we theorised that highly motivated individuals who can ‘overcome’ the ego depletion state are more likely to engage in effective stress management, bringing about more engagement in self‐regulation of HPB. Hence, more research may be done on how nurses can cultivate intrinsic motivation to bring about sustainable behavioural change.

### 4.3. Professional Nursing Identity

Lastly, the most notable finding in this study was the dichotomy between the professional identity and personal identity, as evident from nurses’ responses. An essential component of the nursing identity is patient‐centredness [66]. This was highlighted in our study, where the nurses prioritised patients’ health and their work at the expense of their own. This can also be seen in our quantitative results, where the ‘Health Responsibility’ domain of the HPLP‐II is the lowest domain (mean = 2.04). ‘Health responsibility’ which is defined as an active sense in taking accountability of one’s own health is scored the lowest is surprising considering nurses are meant to encourage their patients to actively make changes in their lifestyle to improve their conditions. While the tendency for nurses to go beyond for their patients is often celebrated, this will become counterproductive for the organisation as the nurses’ well‐being declines, leading to a decrease in quality of patient care [67]. Notably, after the COVID‐19 pandemic, outpouring support for nurses manifested into them being heralded as heroes. However, the public will need to recognise that nurses are not ‘superhuman’—they are human beings who have interests and identities outside of their occupation [68]. Thus, the findings highlight the conflicting messages of institutions to encourage healthy living and self‐care among nurses yet commend the image of a selfless nurse.

In summary, the findings in this mixed‐methods paper provided more insights into major facilitators such as intrinsic motivation and social support to encourage healthier lifestyles, confirming the importance of intrapersonal and interpersonal elements of Pender’s Health Promotion Model for nurses’ adoption of HPB [39], which solely with a quantitative or qualitative study would be unable to provide a comprehensive and holistic overview of the complex interconnectedness of self‐regulation of HPB and its various elements. While the environment contributes to the nurses’ lifestyle choices, ultimate responsibility for maintaining their own health lies with themselves. Hence, more research on how intrinsic motivation can be cultivated is needed.

### 4.4. Implications

In terms of policy and practice, these findings suggest that the workplace culture is malleable to becoming more conducive for self‐regulation with more organisational support. It also suggests that lifestyle interventions implemented for nurses should be less time intensive and convenient to address the time constraints imposed by the nursing lifestyle. An emphasis on nurses taking ownership of their own health instead of propagating a strong self‐sacrificial mindset as a rebranding of the nursing image may prove beneficial. Engagement in effective stress management as part of the nursing education can also be cultivated early to increase self‐regulation as student nurses transition into full‐fledged nurses. For research, more could be done on developing interventions that focus on actively establishing a positive workplace culture for facilitating self‐regulation of HPB. This study’s findings could also inform the development of lifestyle interventions specifically tailored to nurses for sustainable adoption of HPB. Additionally, with insights gained on self‐regulation, future research could explore its transferability to the other populations such as the patient population for positive, long‐term behavioural change. For education, this study’s findings could be incorporated into nurses’ training and education, emphasising self‐regulation and instilling the importance of self‐care into the nursing identity early.

### 4.5. Limitations/Strengths

The study had several limitations. Namely, the low number of survey responses for the quantitative portion of the study. Due to this, there was a lack of statistical tests that could have been done. However, the descriptive statistics still provided a brief overview of the situation and provided sufficient context for the qualitative portion, supplementing it. Because of the first limitation mentioned, the pool of participants for the qualitative portion was mostly homogeneous, which may compromise its representativeness of Singapore’s diverse nursing workforce, which comprises a large proportion of foreign nurses. The small sample size increases the risk of type 2 errors, potentially limiting the study’s ability to detect significant differences. However, the sample still included nurses from a variety of institutions and specialities, providing a diverse snapshot. Additionally, the younger demographics of this study unexpectedly provided more insights into the struggles of maintaining an active lifestyle as new full‐fledged nurses transitioned from student nurses. Moreover, there was only a single coder for the generation of the themes, affecting the dependability of the results. This was addressed with two other researchers who evaluated the appropriateness of the generated codes and themes, discussing and resolving discrepancies when necessary. Additionally, the use of open‐ended questions in the qualitative portion of the study enabled new themes that are different from existing literature to be evoked, allowing for deeper exploration [69].

## 5. Conclusion

This study explored the experiences of nurses on self‐regulation of HPB and the influence of workplace environment on these habits. The findings of this study highlighted the nurses’ difficulty in self‐regulating their lifestyles into a healthy one due to low prioritisation of their own health. Moreover, the current workplace environment is unconducive for facilitating self‐regulation due to inadequate support and the current workplace culture that propagates unhealthy lifestyles. The findings call for attention organisational support to bolster nurses’ capacity to adopt HPB and to improve their mental and physical well‐being. Future research should be on developing interventions that actively foster a more positive workplace environment for self‐regulation.

While this study highlighted difficulties nurses face, it also offered the nursing workforce a unique opportunity to redefine the professional nursing identity into one that truly embodies health by embracing self‐regulation of HPB and to shape a positive workplace culture that supports this. Nevertheless, transforming these flaws into opportunities will require a strong collaborative effort between the leadership across all nursing domains and the day‐to‐day nurse as they take greater ownership of not only their patients’ health but also their own.

**Nomenclature**
HPB: Health‐promoting behaviours
GP: General practitioner
SMSR: Strength model of self‐regulation
MOH: Ministry of Health
SN: Staff nurse

## Ethics Statement

Ethics approval was obtained from the National University of Singapore Institutional Review Board (NUS‐IRB) (Reference Code: NUS‐IRB‐2023‐511).

## Disclosure

Ms Wei Lin Sandy Ang has contributed to this study by reviewing the interview results and serving as a second coder. An earlier version of this study titled: “Self‐Regulating Healthy Lifestyles in Nurses: A Mixed‐Method Study” was published in various institutional repositories/university’s bachelor theses database [70, 71].

## Conflicts of Interest

The authors declare no conflicts of interest.

## Author Contributions

John Christopher Lambino Navarro: conceptualization; data curation; formal analysis; investigation; methodology; roles/writing–original draft; writing–review and editing.

Han Shi Jocelyn Chew: conceptualization; formal analysis; methodology; supervision; validation; visualization; writing–review and editing.

Siti Zubaidah Mordiffi: writing–review and editing.

Sky Wei Chee Koh: writing–review and editing.

## Funding

The authors received no financial support for the research, authorship, and/or publication of this article.

## Supporting Information

Additional supporting information can be found online in the Supporting Information section.

## Supporting information


**Supporting Information 1** SM 1: The poster that was created and used to promote the study and increase recruitment of participants. It was propagated on social media platforms.

**Supporting Information 2** SM 2: Interview guide used for all 24 semistructured interviews in this study, providing a brief outline of the questions and follow‐up used during the interviews.

**Supporting Information 3** SM 3: The full interview transcripts of participants P1 to P24. It also includes observational notes of participants and displays first author’s reflexivity.

**Supporting Information 4** SM 4: An overview of the quantitative portion of the study: the questionnaire made on the Qualtrics platform that each of the participants had to do prior to being selected for the qualitative interview.

**Supporting Information 5** SM 5: The coding tree excel sheet that shows the generation of subthemes and themes that was done during data analysis.

**Supporting Information 6** SM 6: Correlation analysis results between HPLP‐II and PSS‐10 scores.

**Supporting Information 7** SM 7: A Checklist of Mixed Methods Elements in a Submission for Advancing the Methodology of Mixed Methods Research.

## Data Availability

The data that support the findings of this study are available from the corresponding author upon reasonable request.
