# Supplementary material for: Self‐Regulation of Healthy Lifestyles in the Nursing Workplace: A Mixed‐Method Evaluation
Source: J Nurs Manag. 2026 Jan 15;2026:2199578. doi: 10.1155/jonm/2199578 (PMC12807584; doi:10.1155/jonm/2199578)
Supplement: Supplementary file 1 — Supporting Information 1 SM 1: The poster that was created and used to promote the study and increase recruitment of participants. It was propagated on social media platforms. [file JONM-2026-2199578-s001.pdf]

As a nurse, you manage your patients' health...

BUT HAVE YOU MANAGED YOURS?

Research study for nurses

What is it about?

This research study aims to find out more about the struggles of nurses in maintaining their own health.

Participants will be asked to do an online questionnaire. 30 participants will then be selected for an interview to elaborate. Further details will be shared to selected participants.

Why participate?

- Your responses could potentially help to shape future policy changes to improve health outcomes for the nursing workforce.

Who can help? :)

- Currently working as a full-time nurse in a Singapore healthcare institution
- Adults aged 21-65 years old
- Does not have health conditions that impact their ability to normally engage in physical activity (e.g. pregnancy).

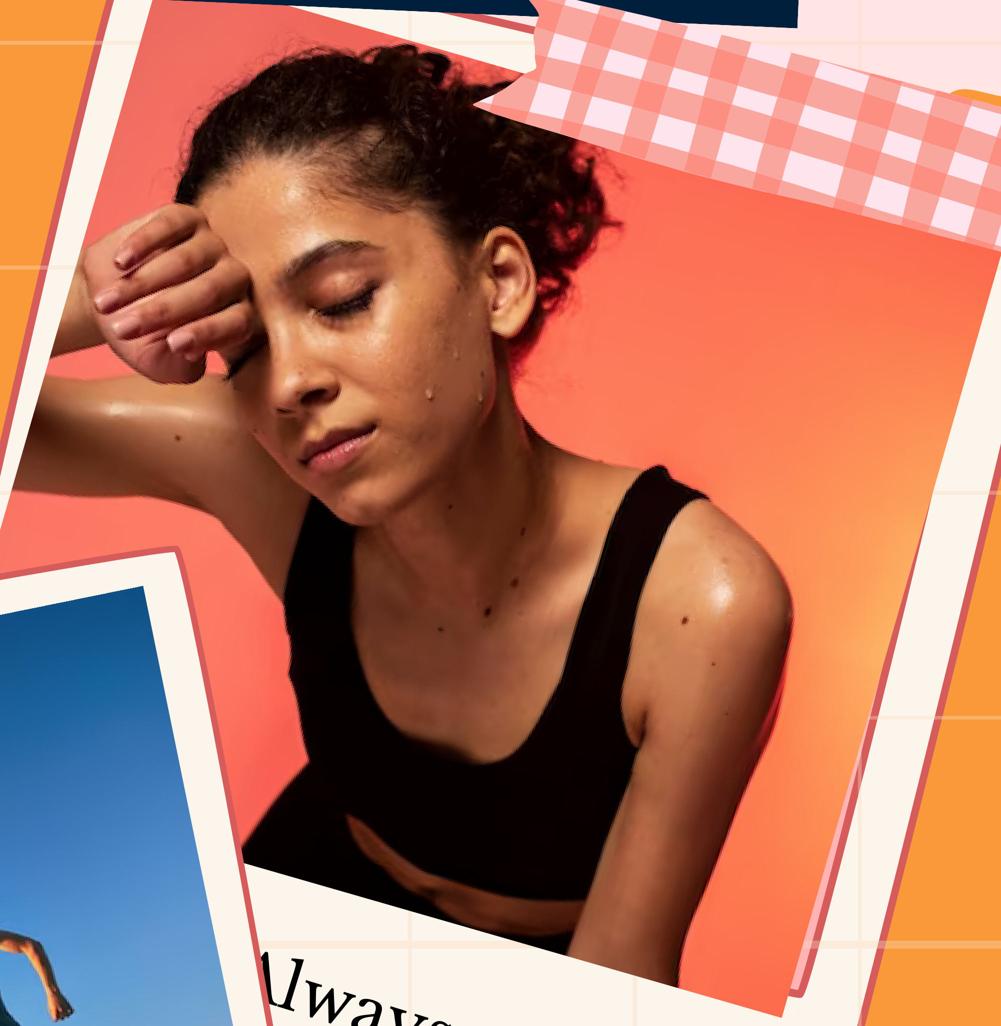

[illegible]lways stressed?

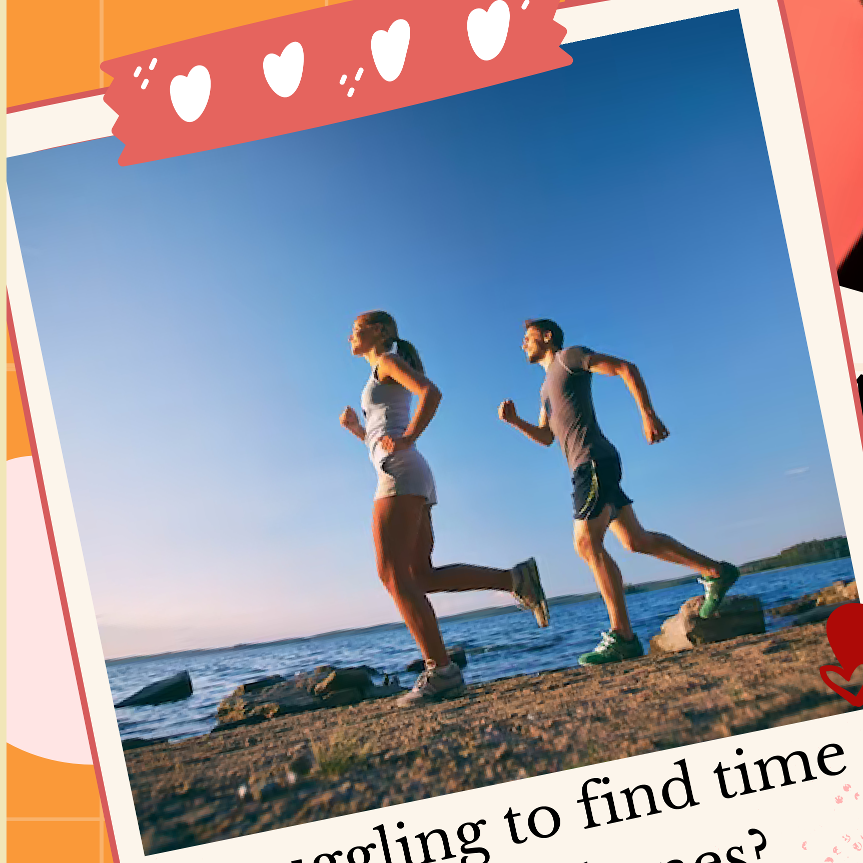

Struggling to find time with loved ones?

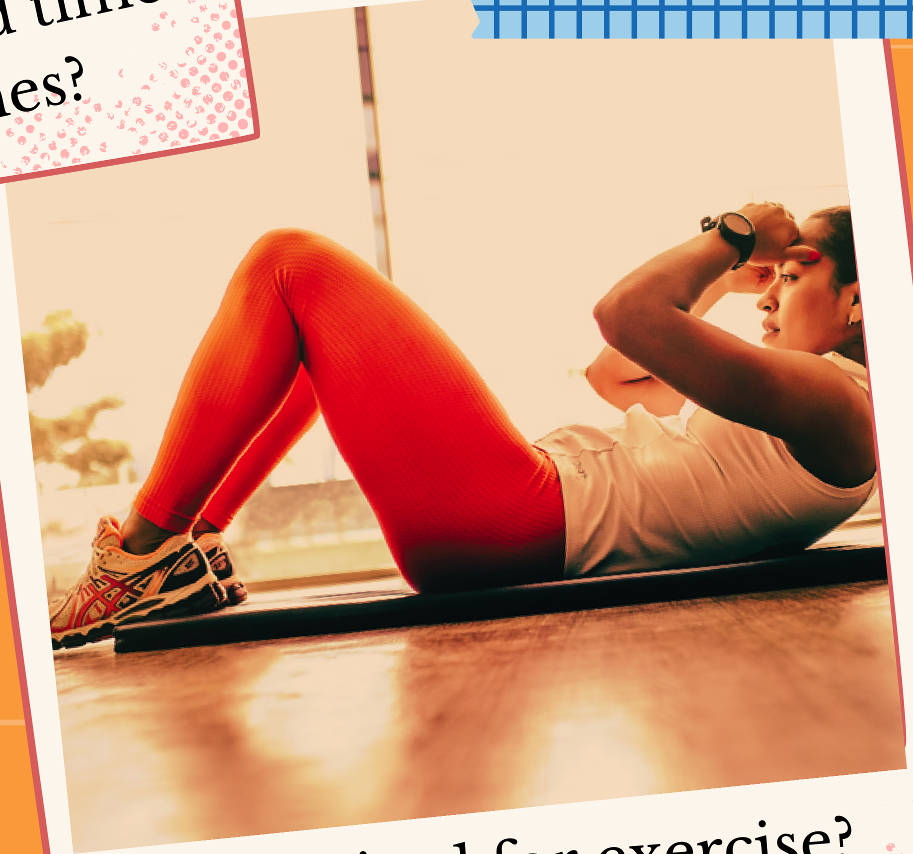

Too tired for exercise?

➡ Interested? ⬅

Help by scanning the QR code to do the questionnaire!

For more information, you may call or email a member of our study team:

Navarro John Christopher
81899741
e0540258@u.nus.edu

https://tinyurl.com/nurseshealthylife

IRB #: NUS-IRB-2023-511 The Principal Investigator of this study is Dr Jocelyn Chew. You may email the Institutional Review Board at irb@nus.edu.sg
