# Supplementary material for: Self‐Regulation of Healthy Lifestyles in the Nursing Workplace: A Mixed‐Method Evaluation
Source: J Nurs Manag. 2026 Jan 15;2026:2199578. doi: 10.1155/jonm/2199578 (PMC12807584; doi:10.1155/jonm/2199578)
Supplement: Supplementary file 2 — Supporting Information 2 SM 2: Interview guide used for all 24 semistructured interviews in this study, providing a brief outline of the questions and follow‐up used during the interviews. [file JONM-2026-2199578-s002.docx]

**Supplementary material 2**

Attached here is the interview guide used for all 24 semi-structured interviews.

Self-regulation of healthy lifestyles in the nursing workplace: A mixed-method evaluation

Qualitative Interview Guide

Type of interview: Semi-structured

Estimated duration: Approximately 1 hour

Pilot status: Done

PREPARATION

Context

Each individual interview will be conducted in a conversational style. Wording and order of question will change accordingly to participants responses. Prompting questions will be carefully used by the interviewer for further details and clarifications. The table only contains a few of these prompts and it is not comprehensive. In this study, the target population is nurses, and the focus will be on their perspectives of self-regulating their lifestyles.

Aim: To explore the experiences of self-regulation of health-promoting behaviours in nurses.

Research questions:

(1) What are nurses’ experiences on self-regulating HPB?

(2) How does the workplace environment influence self-regulation of HPB among nurses?

INITIATION

Start of interview

Good morning sir/miss,

Firstly, I would like to thank you for taking the time to speak with me today. My name is Christopher.

The aim of this interview is to get a better understanding on the experiences nurses’ have on self-regulating their own health behaviours, and how various factors may influence this. I want to find out what are your views, opinions and experiences on this. I would really like to encourage you to speak what comes to mind. There are no right or wrong answers. May I have your permission to continue video recording the session?

Introduction

Let us start with some basic questions,

Which ward are you working in? (Environmental context)

EXPLORATION

*Core questions and potential prompts to be asked during the interview*

| Research questions | Core questions | Potential prompts |
| --- | --- | --- |
| What are nurses’ perceptions on self-regulating health-promoting behaviours? | 1. As a nurse, how would you define a ‘healthy lifestyle’? (Transition)  2. As a nurse, how do you feel about self-regulating a healthy lifestyle/maintaining such a lifestyle?  3. What are your main facilitators/motivators or barriers in self-regulating/maintaining a healthier lifestyle?  4. How does your personal lifestyle habits/self-regulation habits impact your work performance? | How did your lifestyle change after you started to work as a nurse?  Specifically on diet/physical activity, how do you feel about your current lifestyle? Do you feel like changing it?  How do you feel self-regulation affects your life?  Outside of work, what do you think influences your self-regulation/lifestyle?  Are there any tools that you are personally using to self-regulate your lifestyle? |
| How does the workplace environment influence self-regulation of health-promoting behaviours among nurses? | 5. As a nurse, how do you think the workplace influences your own self-regulation/lifestyle habits?  6. How do you feel about the workplace environment/culture among nurses in helping to facilitate or hinder self-regulation/maintenance of healthy lifestyles?  7. How do you think your institution can help you to self-regulate/maintain a healthier lifestyle? | What do you think about the role of nurse managers in helping nurses self-regulate/maintain their own health?  Are there workplace health promotion programmes in place? |

TERMINATION

(Summary of what has been shared)

Do you have anything else that you would like to speak about that was not mentioned in the interview? Any further comments you want to share?

This marks the end of the interview.

Thank you for your time and participation.
