# Supplementary material for: Self‐Regulation of Healthy Lifestyles in the Nursing Workplace: A Mixed‐Method Evaluation
Source: J Nurs Manag. 2026 Jan 15;2026:2199578. doi: 10.1155/jonm/2199578 (PMC12807584; doi:10.1155/jonm/2199578)
Supplement: Supplementary file 3 — Supporting Information 3 SM 3: The full interview transcripts of participants P1 to P24. It also includes observational notes of participants and displays first author’s reflexivity. [file JONM-2026-2199578-s003.docx]

**Supplementary Material 3**

Interview transcripts of P1 to P24.

# **Interview #1**

Participant and interview details
 Participant number: 1 (P1)
 Date of interview: 11 Sept 2023
 Interviewer Number: Navarro John Christopher Lambino (NJCL)
 Time IDI started: 2000
 Time IDI ended: 2047
 Observational notes: Participant seems cheerful at the start

Section I: Interviewer’s summary
 The individual interview was conducted via Zoom and the participant appeared to be very relaxed, in the comforts of her home. As this was the very first interview, this was meant to be the pilot interview. However, there was no need to make any changes and this interview will be part of the data for analysis. Perhaps, due to it being the first interview, I was a bit apprehensive, and stumbled on my words a bit and tended to over-explain my question, fearing that the participant misunderstood. There was no need for it as patient understood most of the questions and answered on point. During the interview, she was very forthcoming in sharing about her personal lifestyle habits, especially on workplace culture. Freely elaborates when prompted and explains her views clearly. Due to the basic Zoom account limitations of 40 min per session, there was an interruption in the middle of the interview. Participant was very understanding and the interview continued as normal. Subsequent interviews will not suffer this limitation. Additionally, this interview had minor inaudible segments due to connection issues. For some context, participant is relatively new nurse with 3 years of experience. She has transferred from a general ward to SICU and seems to generally favor her new setting. Manpower issues was cited as a major detriment to healthy lifestyles. Her experience could be attributed to the peak of COVID epidemic where there was an unsustainable nurse to patient ratio of 1:18. Using her own experiences of transferring from general ward to SICU as a reference, she was able to give strong examples/comparison when answering questions regarding workplace culture. Interesting points mentioned was regarding horizontal bullying, having goals outside of work and putting an effort to go back to ‘baseline’ healthier lifestyle. Additionally, shift work is a major barrier to maintain a healthier lifestyle.

Section II: Transcript

**NJCL: Okay. Yes, is it recording? Alright. Firstly, thank you so much for taking the time to speak with me. My name is Christopher and I am a Y4 Nursing student. The aim of this interview is to get a better understanding on the perspectives nurses have on self-managing their own health and health behaviours and health habits and so on. And how various factors influence this. I want to find out what are your views, opinions and experiences on this. I would really like to encourage you to speak your mind. And there are no right or wrong answers. May I have your permission to record this?**

P1: Sure.

**NJCL: Alright, okay. Can. Alright, how about we start with some basic questions? Like uh, which ward do you work in? I mean, clearly I can see your [Zoom] username has the words SICU…**

P1: [NJCL: Participant laughs] Sorry.

**NJCL: But could you tell me more about that ward in particular?**

P1: Okay, so, I am in [Participant’s hospital] working in surgical ICU. Before that, I was working in a general surgical GW for 2 years. So I only worked in SICU for 1 year. I am a 3^rd^ Year nursing- I mean I only spent 3 years in nursing so far. So far, in SICU, I think the work life balance is more manageable. So I deal with critical patients everyday, so the stress is a bit different from what I experience in a general ward level where everything was more task-orientated. Like I’m trying to finish- get everything done by the end of the day. But over here, I am stressing over how to keep my patients alive.

**NJCL: Wow, I see. Alright, thank you for that. My next few questions will be regarding your lifestyle in general. So, for example- for for my first question: As a nurse, how would you define a healthy lifestyle?**

I think, mentally you have to be resilient to tackle what is happening at work and in your life as well. So uh, there will definitely be stressors in your life, but how you cope with it is one thing. So being mentally strong and having a good support system is important, that’s one aspect. And the other aspect to take care of your own physical health. What def- what I feel is healthy, is you know you maintain- you have an active lifestyle and you watch your diet well, I mean not to the extent where its restrictive, but you know you don’t actively binge eat or something like that. So I think that is what a healthy lifestyle is to me. And mainly to keep a more relaxed and happy outlook on life.

**NJCL: I see. Could you elaborate more on the support system?**

P1: Oh okay, support system meaning, ummm, who can you look to when you’re feeling very stressed or overwhelmed at the moment. For me I have my family and friends and even work colleagues so maybe for work related issues, I can speak to my work colleagues who probably be able to give me better advice. But other stressors in life, I can look towards my family, my personal friends and my partner. Like, to give me advice, or maybe just have a- for me to rant out frustrations only.

**NJCL: I see. Hmm. Then, your earlier answer also mentioned something about watching your diet. Could you elaborate more on that?**

P1: [NJCL: Participant laughs here before answering] Okay um, maybe some background history. Before I joined nursing, I had a very active lifestyle, but I was a little underweight. So when I joined- after my first 6 months of nursing I was so stressed, that I lost even more weight which I became even more underweight. And then after that, you know the stress level is different from your first 6 months of nursing and the subsequent 6 months. Because the first 6 months you’re trying to float, you’re so stressed that you can’t even work, your scared to even turn up for work. But the next 6 months once you get into a certain routine, you are so stressed that you cannot finish your work on time. Or you are just overrun with work. So when you go home, you just eat a lot. So within that 6 months, the first 6 months I lost around 4kg but the next 6 months I lost about- no, I gained 10kg. It’s very drastic. So that definitely wasn’t good. Ya, so diet wise, meaning eating regular meals. I think for nurses we all know that our mealtimes are quite unpredictable. Or has to be a little different from the general public. Cause, let say we even go for break for morning shift you have somewhat of a brunch at 9 or 10 o’clock, I mean, if you follow the usual break timings. Then you won’t have food until like you know, you end shift at 4, then you go for like. I don’t know what’s the next meal. Yeah, something like that. Or you don’t eat any food throughout the day, because you’re so busy you don’t get to go for break. You end up eating one big amount of food later on which is also not good for you. Ya, so when you’re so hungry, the type of food you tend to eat differs from what you would actually want to eat, or what your body really needs. So you go for the easiest or most convenient food, right? Ya, but, other than- if you have time to like- you know like normal people who go to work on regular hours, they can probably go out and prepare food for themselves. So I feel like my colleagues also experience the same problem. We just head for 7-Eleven food just to fill our tummy quickly and get back to work. Yeah something like that, but not good for us in the long run.

**NJCL: Hmm, I see, I see. Then earlier, you mentioned that you actually used to more active before nursing.**

P1: Ya.

**NJCL: Then only, only after joining nursing then- so what happened there, how did your lifestyle actually change because of your work as a nurse?**

P1: Okay, so before that. I had- when I was still schooling like you. I had a very disciplined lifestyle. Wake up at 6, go to sleep by 10pm. Something like that when I was schooling. I had extracurricular activities like I would play squash for the school or I will go for runs and participate in marathons. But when I started nursing, obviously all that had to change because you are starting to work shift work. And work was quite overwhelming with the extra overtime hours that you put in. So I think, what happened was, I use to exercise at least 6-7 times a week. Then, it cut down to 3 times a week, and sometimes it was so difficult to go out to exercise and naturally the intensity of the exercise dropped as well. Ya so, my body wasn’t feeling used to it- I know compared to a regular person, probably 3-4 times is sufficient but it was just very different for me. And I think at one point of time, I even stopped exercising for one whole month because I just gave up completely. Ya, so, my body didn’t feel good also. And once you stop, it’s very hard to start back- kickstart again. So I think that was what happened to me. But right now, I managed to get back into routine already. But it takes some time to get there.

**NJCL: I see. Ohh. Okay. Hmm, so okay, moving on to my next question. It’s actually related to what you have been saying, it’s basically on how- as a nurse, how do you feel about maintaining such a, such a lifestyle, lifestyle? With reference to how you defined a healthy lifestyle earlier, how do you feel about maintaining such a healthy lifestyle?**

P1: Hmm, okay. I think it’s a lot to do with time management, so I think one of the biggest challenge that we face is the 3 rotating shifts like how you get into a routine because one day your doing an AM shift, next day you’re doing a night shift, and then after that you only have off day and then you go back to work the next day with a PM shift. It’s very disruptive. But actually I see some benefits in such a shift, roster I mean. You get to see your roster 1 month in advance right? So you can plan it- it’s all about planning and discipline. You just have to see which days you can work out more, which days you can work out less. Because for me, I am also training for a half-marathon.

**NJCL: Wow. I see.**

P1: So I am following a marathon program. So some days when you know you have a training program, you put it on your off days or the days when you have your first night shift, and then yeah so you have the luxury of planning your workouts accordingly. I mean, another benefit is you can use the gym at off peak hours so you can do what you want. But it really requires a lot of planning. And you need a group of- a community who will exercise with you. So for me, I am very lucky. How I got back into routine after stopping for so long was that I had one friend who invited me to a gym randomly. She said just join together for the trial session. And somehow we liked it, we stuck with it. And we have been doing this for 1 and half years for now and that’s how we kept a regular routine. Ya because- if I don’t go, I know my friend is at the gym and I can’t just leave her alone, right? So I have no choice but to be forced into the gym. Ya, something like that. And, I think it’s more- the community will really help me a lot. Even in my workplace right now, I also have a group of friends who are exercising with me, so it’s about being with like-minded people. And you know having a goal to work towards to, so for me I have a half-marathon to train for, so yes. That helped me a lot in that aspect as well.

**NJCL: I see, hmm, goal to work towards. I see, I see. I think I understand.**

P1: Ya, I think it’s like just remembering that you have a life out of work. And you’re not just defined- because I know you spent a lot of time at your workplace but you have to remember that your life goes beyond what they look at you at the workplace, so outside of work, who are you, what are you working towards to. I think that’s something that most of us tend to forget. And I really lost track of that especially when I started of as a young nurse as well. Because you have a lot to proof to others as well, so you get so absorbed in your work that you give 110% but you forget that oh, I have to rest myself and take care of myself as well. Something along that line.

**NJCL: Wow. Quite insightful, quite insightful. Okay, moving on to my next question. What do you think are the main facilitators/barriers in maintaining your healthy lifestyle. I mean, I’m guessing one of your facilitators was having a community, having a goal. But what about barriers, what do you think is stopping you from having a healthier lifestyle?**

P1: [NJCL: Inaudible segment] My pantry has a lot of junk foods

**NJCL: Sorry?**

P1: My ward pantry, you know, we have a pantry, ya? Normally they don’t stock the pantry with the most healthy food. It is mainly just biscuits or whatever, tidbits, easy to eat food.. Quick and uh, quick and go foods. So I think that- and my ward is very loving, so we have- we always have snacks. Our colleagues will always bring snacks and top up the pantry. It’s too much, it’s like a mini-supermarket. So that’s one of the barriers. But another barrier, I would have to say is really the shift work. Cause- like I said even though there are some pros to eat like you know can plan your workouts in advance. But some days, after night shift it’s really very difficult to get yourself out of the house to exercise. So, that’s when the facilitators help like you know, you have friends who push you, and you feel obliged to go you have friends there waiting for you. Ya. But I think another facilitator for me, is, like, I think, when I was growing up, I was already active. So, I feel compelled to keep to my, to a certain lifestyle I was used to already. So I didn’t just give up to my routine completely, I really made an effort to go back to where I was.

**NJCL: So to summarize what you have been saying, your main facilitators would be your community of your friends always encouraging you, having goals to work towards outside of the workplace.**

P1: Ya.

**NJCL: And then you’re also compelled to a certain lifestyle, because of your past experience. Your past lifestyle.**

P1: Ya, ya.

**NJCL: Then for barriers, it would be your pantry is always stocked full of unhealthy snacks.**

P1:[NJCL: Participant laughs here] Ya.

**NJCL: And shift work. Ya. But then you-**

P1: I think I forgot to add on, I think for the mental aspect- the barriers as well. The perception that we have to go above and beyond for our patients sometimes, so you bring back some of the work with you. So you can’t- sometimes you feel so frustrated at what you experience at work, but you’re expected to have a certain empathy level. So, when you want to vent out your frustration to someone, you know, for them to have a listening, people can’t relate to you. And you feel very stressed. Sometimes you have pressure from your supervisors, and then your work colleagues. Sometimes there’s horizontal bullying. I am not denying that. So you don’t know who to vent out to. Because from the top, you can’t express yourself well, your concerns well. To your colleagues, yes. I mean, some of them you can vent out your frustrations to some of them, but at horizontal level, not many people could help you also [NJCL: Inaudible segment. Connection issues] Incidents like bullying occur, and even I myself feel hopeless in how I can help. Or when patients have unrealistic expectations of you. You can’t fight back, because you know you’re a nurse You’re suppose to be compassionate, you’re suppose to have empathy. So you bring all these frustrations home. So, I think it’s how like the public perceives us sometimes that makes it very hard for you to vent out your frustrations like how others do at their workplace, “this person is so annoying”, so and so, but I can’t say that my patient is so annoying so and so because he’s sick. Something along that line. Yes, so I think the mental aspect is very difficult to manage also. It’s really just hard to leave everything at work sometimes. Sometimes you just bring it- you bring some parts of it back with you at times.

**NJCL: Wow. I see. [long pause] Could you elaborate on the horizontal bullying?**

P1: Okay. So, horizontal bullying is you know when people with the same rank as you will talk about you in very nasty ways or you know, they will do some deliberate actions that will make your life very miserable. This happens at every place. At every workplace. It’s just a matter of how, the extent of how bad it gets. So maybe I just give you an example. It didn’t happen to me, thankfully. But maybe a person will just openly tell everyone that this so and so is a very bad worker or purposely exclude her from meetings, or you know, conversations. Or say very harsh words during handover, like “how can you not know this?” or “You don’t have time to do this? Why are you so useless?” It- it’s what’s said to other people right in their face or behind their backs. Or just you know, throwing things at them. It really happened before. So, you- I mean, yes, you leave what you face at work, work is work. People will keep saying that, but you will feel hurt. Some things- once you say it out, the hurt will be there forever. **[NJCL: Observational note: participant sounds emotional at this point.]** So, this kind of things, no one can really help you. Especially, lets say, I see it happening to my friend, I will feel second-hand hurt, because I can’t help my friend. What can I do? I can only tell my managers, “hey this happened”. But-what. The managers can’t do much apart from counselling the perpetrator. And you can’t speak up for yourself. Because that person for example has a lot of power. In other ways, maybe not managerial power, but you know, influence [NJCL: Inaudible segment, connection issues]. May just tell her “it’s okay” and not to worry in the working environment. But other than that, what else can we really do? Yes, we can speak up. Probably I can say “maybe no la she’s not like that. Probably she’s stressed, or she’s busy at work or her shift was really occupied that’s why she couldn’t do this”. That’s all I can do for my colleague. But it’s very difficult for an individual to step up and say what you’re doing is wrong, because soon you’ll be the next victim. But no one wants to be the next victim, right? So, that is something that I am trying to work towards to but I know that it’s very difficult. And I feel that the responsibility lies in everyone- the person being bullied to speak up for herself, her friends especially. And then the manager to make sure it doesn’t happen but it’s inevitable. Humans are humans. This kind of powerplay will always exist in workplace and stuff.

**NJCL: Would you say that in your, maybe in your workplace specifically, is there any like system in place to actually help prevent this kind of thing?**

P1: Yes. Yes there is

**NJCL: Could you elaborate on that?**

P1: It’s not in my current ward right now, I won’t say which ward either. But yes, counselling- [NJCL: Participant laughs]. Because I mean, I know this is going to be transcribed so I need to say that it’s not in my current ward. But I won’t tell you where it happens.

**NJCL: No problem, no problem. Your identity will be kept confidential. [Laughs]**

P1: [NJCL: Participant laughs] So this happened in my previous ward. So what the manager did was, yes, sit down one on one counselling. And because, this incident was eventually reported to the HR. So the HR actually had to investigate the matter. So, everyone was interviewed, like you know, this really happened. To just get a transparent view on what was going on. So eventually yes, a warning letter was given. But sometimes, a warning letter may not be sufficient. It will just encourage the perpetrator to get back at the person. But ya, what happened was, the manager actively tried to put them in separate stations so that their interactions would be less so that the working environment will be less hostile for that person. But eventually, the person being bullied decided to transfer out of the ward sadly. So yes, there are systems in place, there are avenues to reach out to. But sometimes its still not sufficient. So, if from my point of view, I feel that it is very unfair that the person had to leave because she couldn’t take it anymore. But the person who is actually doing the bullying is still there. I mean, you wont get the perfect happy ending. But there are measures in place for such incidents. It’s just whether you like the ending or not.

**NJCL: Wow. I see, I see. Hmm. [long pause]. Okay. Let me just transcribe a bit. Hmm. Would you say that this kind of horizontal bullying happens more in new nurses or experienced nurses?**

P1: I think definitely new nurses get it more. Because, ya, I think everyone- when you’re new people will not trust you so readily, right? They will watch you, they will doubt you. So, yes, new nurses get it more. And by, they already have their own clique of friends, the experienced nurses. So they have more protection than us, basically. So it doesn’t happen to them as often.

**NJCL: Alright. Okay, understand. Okay, moving back a bit to lifestyle habits again. How do you think your personal lifestyle habits impact work performance?**

P1: [NJCL: Participant laughs before answering] Well, I can run pretty fast, so my work performance, I’m always running to the blood bank to get bloods. Or you know, you have more stamina to go and endure the shift. For physical wise, ya, apart from enduring the whole difficult shift. I think the mental aspect affects your work performance more. Because, if you’re more resilient, you can take criticisms in a more, uhh, you don’t take it too hard, you don’t take it personally, but you see it as a way to improve. You will progress very fast. You will learn very fast. So it’s more of the mental aspect that will affect your work performance better. So for me, I am very lighthearted. So if anyone criticizes me, yes I will say sorry. I will learn, but I won’t take it too hard. I will you know, just make sure I learn from it and move on. And I move on really fast. Or if anyone scolds me, I am quite shameless in the aspect that I will dare to speak to the colleague who scolded me and still make jokes with them. Like any criticisms I will take it seriously, but I will respond back lightheartedly, so that you know, I won’t get- I don’t feel so bad at myself. Like. you know to me and my senior, would be more open in telling “hey, I made this mistake” so that she won’t think I always take things personally. So it’s a win-win situation for me. I think for me, I made quite a lot of mistakes when I was transiting from a nursing student to a full fledged nurse. So ya, of course, I cried many times. But I took it, I took my lessons seriously. And I moved on. And within a week, it was out of mind. But I know what I need to improve on. Something like that. Ya.

**NJCL: So, judging from what you said, is it right to say that, your personal lifestyle habits, actually influence your physical and mental strength so that you’re able to go through your shift better before you started?**

P1: I’m so sorry. Could you repeat everything just now? I think the screen just froze for a while.

**NJCL: Yes, sure no problem. Just ah, summarizing a bit of what you said earlier. It is right for me to say that, how you think your lifestyle habits impact your work performance is because your lifestyle habits influence your physical and mental strength to be able to endure shifts, criticisms and whatever better?**

P1: Ya. I would agree so. But more heavy on the mental aspect. Because I mean, if you’re not- I mean if you’re a healthy human being, you don’t need to be physically fit to survive the shift. But as long as your healthy then you can work then ya, its fine. But it’s really the mental aspect that makes or break a person during the shift as well. Like how well they cope with it, or how they cope in general. Ya.

**NJCL: Hmm. [long pause] Hmm. How about for example, in a hypothetical scenario, for example, do you think that your diet affects how you give specific dietary advice to patient?**

P1: [NJCL: Patient laughs before answering] Oh, I will never give dietary advice to patient. To each their own body, to each their own lifestyle.

**NJCL: I see, I see.**

P1: And patients sometimes, some of them have really different dietary requirements. So let’s say if I- normally I am in a surgical environment right? And a lot of patients sometimes have DM, diabetes, so I wouldn’t know- I’m not an expert to tell them “hey which food has lower GI that probably won’t spike your sugar levels” Which one would you recommend? The thing is about us nurses giving advice, they would take your words for it, and they would quote your name. So if anything goes wrong, it goes back to you. And it’s quite dangerous also, and maybe some colorectal patients with a stoma, they need a low residue diet. I’m not the right person to advice, so what they need is not in line with what I’m working towards to. So, our goals are not the same, so I can’t really give them advice. I will leave it to the dieticians. I won’t be stealing their jobs.

**NJCL: Alright. Okay. My next question is somewhat similar to what you were talking about earlier, regarding the workplace. As a nurse, how do you- do you feel that the workplace influences your lifestyle habits?**

P1: Yes. Definitely. So my previous ward, there was not a lot of likeminded people like me. I mean, who wants to exercise. And it was very very hectic and stressful. So for example, a PM shift, I can end work at 1 am and still be at the ward. And then the next day I have an AM shift. So I really go home, sleep for 2 hours, then I go back to work. That kind of thing. It does happen. So those days, obviously you can’t bring yourself to go out and do anything else. All you want to do is just rest after work. So that was a very dark period. [NJCL: Participant laughs here] A very dark period of my life, so, and I don’t have likeminded friends who I could say, would go out with me to exercise. That kind of thing. No, I don’t have that. But in my current ward, they respect my time better. So let’s say, I have to give in extra hours for them, they would pay me back my hours. It’s just a- a work culture thing. So I have a personal time to do what I like. I can take on additional projects and I can be okay with it. And ya like I said, I have colleagues who are also quite health conscious and they will exercise. And I have a lot of new mothers in my ward now. Like, so they’re very health conscious of their food. They will bring healthy food and they will share with us, or say “hey this food is good, this food is probably not so good”. They will give advice in that aspect and I will just listen, and ya, it really influences how you behave and how you live.

**NJCL: Okay. Other than your colleagues, how about nursing managers, like people with higher rank than you. Do you think they influence, facilitate or hinder your own lifestyle?**

P1: I-

**NJCL: Like the brothers and sisters…**

P1: I think lifestyle in a whole, no. Maybe in a mental, how stressed you are, they will influence that more. They don’t really- I don’t really like- I mean I don’t really talk to them about my personal life also, so, for me, no they don’t play a huge role in that.

**NJCL: Okay, hmmm. [long pause] Would you be able to elaborate on workplace culture which you mention earlier. Is there anything in workplace culture that hinders your- you from maintaining your lifestyle?**

P1: Hmm, okay. First of all, I would start- I would preface this with everyone is not a bad person, it’s just the environment that forces them to be what they are. [NJCL: Participant is smiling] Okay, so… you can see where this is heading towards to. My previous ward was very intense, so like I said, can even end up to end work at 1 am. Or AM shift we can end at 7pm. The reason for that is because handover doesn’t mean you can handover your work to your colleagues. They will ask you why this is not done. Because they themselves have a lot of work to do for that shift, so I will never blame them if they’re asking that question. Because if I were in their shoes, I would also be unhappy. So that’s why you feel very obliged to finish all your work. Like whatever happen during your shift, you will complete the task and you will go home. So that is what, affects me in that aspect. But ya, in my current ward now, handover means handover. The work, the type of work is quite different. In ICU, the main goal is to keep the patient alive, keep them stable. You don’t need to do additional paperwork like oh, I’m gonna discharge this patient. This needs to be followed up. This patient is going for surgery later, some labs are still not done. You have- you will stayback to take the bloods for them. Or like you know, this patient has a funny dietary request, or this patient requires an update from the doctor but can’t reach the doctor, so you keep trying to call. Or this patient has to get a scan. There’s really less miscellaneous work like that. Ya, you need a scan in ICU, but the patients are always given priority, so you get them first, so you rarely need to chase for all this paperwork. Or you know, things that I feel are really- non-nursing related tasks. So we do less of that, so you have less things to follow up on. These non-nursing related activities, you don’t really wish to hand them to your next colleague, ya because they won’t have time to do it either. But for a critical care setting, what your handing over is essentially the care of the patient to keep them stable. So that can be handed over more easily. And you are only handing over 1 to 2 patient, and that is reasonable. Compared to, okay back then the ratio, where I was at, 1 nurse was in charge of 16 to 18 patients, depending. So you can’t hand over 16 to 18 patients worth of non-nursing related work. You will feel really bad for handing it over to them. That’s why you stay back and stress and you feel easily irritable. Ya. So it does make a huge difference and it does affect the culture as well. Because everyone was so angry in my previous ward, but you don’t blame them for being nasty because they are so stressed out. That’s why they’re acting a lot like this way. Sometimes you’ll feel hurt and you keep telling yourself, they don’t mean it. But it still hurts at some points of time. In my current environment, it’s less stressful and expect people can talk to you, smile to you. Busy but, yes we still have teamwork. Ya, we won’t say so hurtful words to each other easily. Unless, I mean, they’re really pissed off, or something really happened. And so, yeah.

**NJCL: Hmm, can I verify what you just said. The workplace culture, the main like, hindrance, the main barrier, is the workload. Because this workload will influence, how-**

P1: Yes.

**NJCL: How angsty everyone is, and the culture will become negative. Uh. Moving back to workplace culture, what are- how do you think workplace culture among nurses influence healthy lifestyles. You mentioned something earlier, having nurses with likeminded individuals, being able to influence each other to exercise. Could you elaborate more on that?**

P1: Hmm. I can only give you the perspective of like fresh nurses coming, because I am still considered a fresh nurse. Cause, I mean I did switch environment. For me, when I first joined, no how I felt this place was much better for me is because how well they welcomed me. They set up- they invited me to a new group chat with other newer girl- newer nurses. And then, when we have any like, information, we share with each other. We learn together, we grow together. So it’s how well your community is, how welcoming they are that you know, influence you on how well you cope in your new environment. And yes, like-minded people to exercise together, giving each other pep-talks. Supporting each other during work. I think, really, colleagues make or break you in the ward how you cope. Apart from that, how the colleagues affect, I can’t think of anything else.

**NJCL: [Zoom notification that meeting will end soon] I’m so sorry, but I think the meeting might end any moment, I’ll just send you a new invite…**

P1: Sure, sure.

**NJCL: I only have 2 more questions and then we’re done, I didn’t know it would last this long…**

P1: It’s okay! You can take your time. I will leave the meeting, first okay.

**NJCL: Alright thank you so much, I will reinvite. Thank you.**

-Zoom meeting ends due to Zoom basic account can only have meetings that last for 40 minutes.-

-Reinvited participant-

**NJCL: Okay, hello. Okay. So, so just to reiterate what was mentioned earlier. Regarding the question, how do you feel about the workplace culture among nurses in helping to facilitate or hinder maintenance of the healthy lifestyle. For facilitators you mentioned having likeminded individuals to influence you to exercise or eat healthier in the form of like the new mothers in your ward. Then for barriers, it’s regarding the workload and all the miscellaneous work that will contribute to the workplace culture becoming negative. Uh, is that all, or is there anything you would like to add?**

P1: Ya.

**NJCL: Okay, can. Then regarding my next question, how do you think your institution can help you to maintain a healthier lifestyle?**

P1: Um, for my institution, there are activities- they do try- we have this ‘Work balance group’ that organizes some running event, some exercise event. Or like you know, art. Can you hear me?

**NJCL: I think you cut a bit somewhere in the middle there.**

P1: Can you hear me now? Okay, I will repeat.

**NJCL: I can hear you, but I think your screen froze.**

P1: Now, I’m not really moving. Oh.

**NJCL: Okay it’s back!**

P1: Okay, so my organization has this work life group, so they will plan activities like, run, or some exercise session, taichi yoga. Or some art therapy, music therapy sessions. Or some random events like bouquet making workshop. This kind of things. I mean, the slots are very limited, probably like 20-30 slots for the whole group of nurses. Which I think is not- I mean there are courses available, there’s a peer support group so lets say whenever we need- a staff member meets an overwhelming situation. And needs counselling, the supervisor will ask permission from the staff to refer them to this, or the staff can reach out to them for themselves. To have them a life outside of work, active lifestyle. They do value [NJCL: Inaudible, connection issues] nurses opinions on this as well. And I think recently my organization has spoke out on bullying. I mean not bullying, um, healthcare worker abuse. That is one thing that they are trying to protect healthcare workers. Another thing is, I’m not sure about other institutions, but for mine, we have this thing “Sick leave without MC” So, on the surface, it’s more of like, there’s some minor ailments where you don’t feel well, but not that serious where you don’t need to go to work. But to me, it’s also a mental health break for most of the workers. They’re so tired, that they will take the sick leave without MC. Yes, there are people who abuse it, but I welcome it, because people can take their rest, if they really really need it. Like they’re so sick of it, at the breaking point, they use that MC. It’s- it’s an avenue for them to use, so I do appreciate that also.

**NJCL: I see. Earlier you mentioned that your institution organizes a bunch of activities, like Taichi, yoga, art therapy. How frequently do you participate in these activities?**

P1: Honestly, I don’t participate in any. But I do participate- I mean I just choose what I’m interested in. For example, there’s this SGH run or something like that. But it’s more like- I’m going with my ward, I’ll just run with them since they run. There’s also this ‘step challenges’ as well like you know how many steps you take. Ya, I do participate in that as well. I basically just choose what I like, I won’t go for things like art therapy or stuff. But, to each their own.

**NJCL: I see, I see. Earlier, you mentioned that there are limited slots for these activities. Other than limited slots, can you think of other reasons why someone may not want to participate? I mean, other than limited slots, not interested.**

P1: Oh ya, definitely. Umm, probably the hours because it’s a fixed timing. They’ll tell us, oh, there’s this upcoming. I mean the information, how readily available [NJCL: Freeze in this section, probably due to connection issues] So if you don’t actively look for it, you will never know about this. So I only knew about it, after only 1 and half years of working. Oh so these events do exist. It’s just I never had time to you know, look for it. Or like, my colleagues would never tell me about this. They will tell me about work right, but they won’t tell me about all this kind of thing. Ya. I only knew of it when someone invited me to join them. Oh this thing exist. Apart from that, once they post the information, you don’t get- you can’t really plan in advance. Like cause, all these sometimes depends on your roster. Are you free to attend the event? Are you available, you have to pay a small fee also. Like maybe $20? I’m pretty sure it’s heavily subsidized for some of it. But mm, sometimes you wouldn’t want to pay for it. Ya.

**NJCL: I see, I see.**

P1: And I think, since the age group of nurses is quite wide sometimes, like your young nurses and you’ll have nurses in their 60s-70s. So you won’t really know who you’re going with also. Yes, you can go with a friend, but in such a small compact class. For me, I think I get a bit put off when I want to go for a yoga class but I see a very umm, different age group of people, like I can’t connect with them as well. I’m not trying to say I don’t want to connect with different age groups, it’s just very hard to connect sometimes.

**NJCL: I see, I see. Alright. Okay. Moving on to the very final question. Outside of work, as a nurse, what do you think influences your lifestyle?**

P1: Hmm. I think my goals, like I mentioned before. What I’m working towards to, I mean my baseline lifestyle before I started work, what was it like. My friends… and I think, actually these are the main things. Ya. Can’t really think of anything more.

**NJCL: Okay.**

P1: Oh ya, no. I thought of one more thing.

**NJCL: Oh, can can.**

P1: I think it’s seeing patients, whatever ailments they are dealing with, you don’t want to end up in their shoes. So sometimes you’re a bit more fearful of what- how you live your life. Something like that.

**NJCL: So the patient’s condition actually influences-**

P1: Ya.

**NJCL: Ohh. Alright. I never thought of it like that. Okay, alright. Do you have any further comments** **that you would like to share that was not mentioned in the earlier questions or anything?**

P1: No, I think I’m good.

**NJCL: Alright, then this will mark the end of the interview. Thank you for your participation.**

P1: No problem. All the best.

**NJCL: Thank you! Okay, I will stop the recording.**

-End of transcript-

#

# **Interview #2**

Participant and interview details
 Participant number: 2 (P2)
 Date of interview: 13 Sept 2023
 Interviewer Number: Navarro John Christopher Lambino (NJCL)
 Time IDI started: 1801
 Time IDI ended: 1833

 Section I: Interviewer’s summary
 The individual interview was conducted via Zoom and the participant was still wearing their uniform. Prior to the interview, she said that she needed a bit more time as she was stuck in traffic. Hence, it is safe to assume she was on her back from a shift. Despite this, she was able to still answer energetically and elaborated appropriately when prompted. Key points mentioned by the participant also included work-life balance and the impact of social media. She also highlighted the fact that break times tend to not flexible and she will just take whatever break was given to her despite her own lifestyle habits of perhaps intermittent fasting. Interestingly enough, she also mentioned being a Muslim, and how this break times are fixed for them as the last timings as they do not use this breaks during the fasting month to buy food anyway. Fatigue also played a part in contributing to the decision of whether they engage in more physical activity after work, despite already having a rather regular timings in endoscopy. Compared to P1 which said that nursing managers did not have much of a role, P2 said they had a minor role of being broadcasters of activities organized by their institution, and that the nurses on the ground were close with these managers, and even go out together for activities for extra bonding time. Maybe because compared to P1 who has not formed a close bond with her nursing managers, as she just recently transferred to her new ward, she does not enjoy the same close bond and thus have less influence on them. Perhaps, subsequent interview can talk about how closer relations between nurses can strengthen the facilitating or hindering effect on maintaining of healthy lifestyles. Overall, I think I used more prompting questions for this interview, and I should have instead ask participant to elaborate more instead of going straight into the prompting questions.

Section II: Transcript

**NJCL: Okay, let me see, everything is working. Okay.**

P2: Okay.

**NJCL: Alright. Good evening, Ms. P2. Firstly, I would like to thank you for taking the time to speak with me today. My name is Christopher, and I am a NUS Y4 nursing student. The aim of this interview is to get a better understanding on the perspectives that nurses have on self managing their own health behaviours, and how various factors may influence this. I want to find out what are your views, opinions, and experiences on this. I would really like to encourage you to speak your mind, and there are no right or wrong answers. May I have your permission to video record this session.**

P2: Yup.

**NJCL: Alright. Can. Okay, let's start with some basic questions. Uh, which, ward are you working in?**

P2: I'm in endoscopy centre.

**NJCL: Could you tell me more about your ward and like what you do there?**

P2: I’m mostly a procedure, room nurse, so I will be assisting the doctors, the procedure lists. And uh in endo, the terms are similar to OT. So there’s circulating, there’s assisting. So I will be doing the scopes with the doctors. Assisting them to remove any polyps or anything inside. Uh, but there's also the recovery area. So, recovery, there’s pre- and post-procedure for patients. Phases of care. Yeah. So it's basically from start to end la. Patient’s journey.

**NJCL: I see. Okay, thank you for that. Now, moving on to my next question: As a nurse, how would you define a healthy lifestyle?**

P2: To me, I think it's work-life balance, I guess. Yeah. So if- for me, Endo is quite… is giving me the opportunity la because it’s not shift work. It's semi- it's rotating shift, so there's no night like the whole night for us. And then, if we end at this certain timing. We really end at that timing. We- we will just go like, there's people to cover us. Yeah. So, the only thing, the only place difference is we have on calls. On call activations sometimes. So, we will be on standby. Uh, if there's like emergency cases at night. We will have to answer the phone and come back to work. But I think the rotation is enough for us to have work-life balance. Mm.

**NJCL: Hmm! Then uh- for your- for physical activity, how how do you feel about your current amount of physical activity you're engaging in?**

P2: I think it's moderate. Ah umm. Personally, I go to the gym sometimes. And do like lightweight training for strength. Because we need to carry patients ah. So, I think that's best to to to keep that going. And then also, gyming helps me to like uhh take care of my back. Because when we carry heavy things, we tend to let go of our back and that’s where our back pain hurts la. Back pain comes. Yeah. Yeah, nurses usually have back pain problem or even legs. Ya, so, gyming helps me to strengthen everything. So that I'm like, ready to work lah.

**NJCL: I see. Alright. Now, specifically, specifically on diet. How do you feel about your current dietary habits?**

P2: Hmm! Not very healthy. But it, it- it makes me happy. So I think that's the best thing for me. [NJCL: Participant laughs here]

**NJCL: I see. I see. Just know, could you elaborate more on what you mean by not healthy? Like your diet?**

P2: I don't eat vegetables. Yeah, so I don't eat vegetables. I sometimes eat fruit. I prefer fruits more than vegetables. And not healthy in terms of… there's no routine. There's no like fixed- even though my shift is considered not bad, there's no fixed mealtimes. So for breakfast breakfast I would definitely have, because it's already like in me to have breakfast. But lunch it will depend on what time I go, and then it will be uh depending on the time, because if it's during lunchtime there will be a lot of people in like the Kopitiam and all that. So that makes me tend to buy like, just bread to eat for lunch. Then for dinner, it depends. If I'm okay to cook or lazy. Then it’s either I have dinner or don't have dinner. Yeah.

**NJCL: I see, I see. Okay. Then, compare your lifestyle to before you were a nurse to now that you are nurse. How-how would you say, your lifestyle is now? If you compare these two?**

P2: Hmmm. I don't think there's much difference. Yeah, I cannot think of anything. So I can’t think of difference.

**NJCL: Okay, then, just to summarize what you've been mentioning so far… To you, a healthy lifestyle is about work-life balance. Yeah. Having having the the the time for doing what-what makes you happy like you mentioned going to the gym to actually work out and like. although your diet isn't as a regular, which depends on your your schedule as well la. Am I right to say so?**

P2: Yeah, my schedule and my laziness, I guess.

**NJCL: Yeah. Hmm. Then. what do you think- or how do you feel about maintaining such a healthy lifestyle?**

P2: How do I feel?

**NJCL: Like being able to maintain a work-life balance, something that you mentioned earlier.**

P2: I think it's very important because, if not, then it's all about work. And if it’s all about work, then I'll be stressed, and then was nearly like I have- I have eczema, so if I am too stressed, my eczema will flare. So I rather chill than have eczema la.

**NJCL: I see. Okay, okay. Then, what do you think are like facilitators or things that help you maintain a healthier lifestyle?**

P2: A good support systems. Family, friends. And if you’re off work- friends doesn't mean have to be outside of nursing. Even colleagues, off-duty, we can go uh, send them together and and de-stress. For family wise, I guess, when we are home after shift, we might be tired. They are there to help us with- with like, doing anything like, like.. like my mom cooking, And my brothers can like talk to me, talk to be about their life, so that I take my mind off for a while. Yeah.

**NJCL: Other than a good support system. Can you think of anything else that could help you maintain healthier, a healthier lifestyle?**

P2: Hmm. Happiness at work

**NJCL: Happiness at work?**

P2: Yeah. Because, if I’m not happy at work, I don't think I don't think my mind would be satisfied being there. Yeah.

**NJCL: Could you elaborate more on what you mean by happiness at work?**

P2: Our relationships with our colleagues- that's usually easier to to build. Yeah. So but the challenging part is, if our colleague is to… experienced than us like, yeah. So they might think we are still naive or immature to to be a senior or things like that. So, we need to learn like- for me ah. personally, I had to learn how to engage with them, how to to communicate well with them. How to better manage my emotions also, like I- I like how to react, and all that. Ya, then. For patient-wise, my part- my job is quite… I would say, better than what nurses in the ward are facing ah. Yeah uh, the the patients are patient with us. Yeah, in endo.

**NJCL: I see. I see. I see. Then, do you think there are any like barriers or hindrance to maintaining a healthier lifestyle as a nurse?**

P2: As much as our work is- my work is not so draining, I would think. There are still a number of people that that utilizes their sick leave, clear their leaves, and all that. So if the numbers are too high, like for the manpower, then the rest that are on duty has to top up that skillset. Even though the numbers don’t match. Yeah, so that would be… That will lead to burning out ah for our department. Yeah.

**NJCL: [long pause] Do do you think there are any more barriers for maintaining healthier lifestyle other than manpower issues?**

P2: Hmm, I can’t think of anything.

**NJCL: I see. Okay. okay, for the next question: how does your personal lifestyle habits impact your work performance?**

P2: Lifestyle habits… Like?

**NJCL: Like for example, you mentioned going to the gym. So you're stronger and can lift your patients without injuring yourselves, without injuring yourself. Can you think of anything else like maybe, how your diet can impact, how you give dietary advice to other patients?** P2: Oh. yeah. As I say, my health, my diet is not so healthy. But learning things. Then I know, like what the correct thing to say to patient like how to give the correct advice to patients. Um, how to say it, like? I know how to separate work and my own personal like lifestyle. So personally, I know it's not so healthy. But if the patient says: “Oh, I’m doing the same thing. I'm not eating vegetable and all that”. But working in endo, I can see the effects of not eating vegetables enough vegetable. Yeah, so I can advise them with my work knowledge. Ya. Ya.

**NJCL: I see. Okay. Can. And then I'll just summarize what you've been seeing so far in terms of like the facilitators and barriers and maintaining healthier lifestyle for nurses. So for you, your facilitators is having a good support system. which means having family and friends spending time together and de-stressing and also happiness at work which talks about like your relationships with your colleagues and the patients. These are, what you think will facilitate maintaining healthier lifestyle. Then for barriers, it's more of the manpower issues and actually… ya. Manpower issues mostly. Then you also mentioned that your you are able to separate your professional and personal lifestyle. So you're able to give advice. You know your own lifestyle affecting it. Is that correct?**

P2: Yes.

**NJCL: Okay, thank you. Then, moving on to my next question: what are your views on prioritizing your own health?**

P2: Ah. what are my views? It's important ah. like I feel like, if I'm not healthy. Then I cannot take care of other people. Ya.

**NJCL: Okay. Can. And then moving on to my next question. This will be mostly regarding the workplace. As a nurse, do you feel that the workplace influences your lifestyle habits?**

P2: Mmm. yes. Yeah. Example is okay, Before work, before starting my career I didn't take caffeine. and now, since working, and all the all the shifts and all the on calls. I depend on caffeine. Yeah. Then working also, it made me more organized. I guess. Like I started- I don't wanna say OCD, but there's like similarities. I started to get um more particular about hand hygiene or hygiene practices la basically. Yeah. So every time after shift, I will wipe everything down. Yeah. And and I don't change out of my clothes, because the moment I come home. I will just go straight into the shower. Ya, so I don’t consider “waste” a good pair of clothing ah.

**NJCL: Yeah, I see. Other than caffeine. How about your general diet. Do you think the workplace- cause earlier you mentioned something about like how the work schedule will impact your diet. Could you elaborate more on that?**

P2: Okay, so if… the earliest break time that we have is 10 o’clock. and our site the break times are fixed. From 10 to 2. So it's an hour for each person. We will definitely get an hour- is just that we don't know which part of the hour in that period we get. So if we get it around like 12 and 1. That’s the busiest time ah, because it's lunchtime for other people also, right? So that's when a lot of other health care professionals, and and even visitors in the hospital goes for lunch, and then we would- we would even take like 15 min to 20 minutes to queue up for food and get our food before we can even go up to our department and and eat. So that shortens our break time. And then sometimes for me, I don't. I don't want to wait that long to eat. Because if I wait too long to eat; I will eat, and then I won't have time to rest and and and digest, I guess. Yeah. So to me, like, if the queue is too long, I would just get bread in 7-11, or the bakery eat something- at least bread. Yeah, and then I have time to rest. Yeah.

**NJCL: Then earlier. You also mentioned that you go to the gym to workout. How do you think your your workplace schedule like impacts this time for physical activity for you?**

P2: Okay so, my shifts the latest. It ends is 7pm. So if I end 7 I would still have time to go to the gym if my day wasn't as tiring. So it depends on the days easy-ness. because in endo we have procedures to do every- every from 8 from 8 to 5. There's procedures ongoing all the time. So it depends. If my particular procedure room is fully listed or not. So it’s back to back cases. If it’s back to back, and then the that that means the workload for me is capped already ah. If that day happens, then at the end of the day. I won't go to the gym. It just depends on that. Yeah. the shift timing all that, there’s no issue.

**NJCL: Yeah. Okay. All right. moving on to my next question. how do you feel the how do you feel about the workplace culture among nurses, in helping you maintain your healthy lifestyle?**

P2: My colleagues sometimes go on diets, like sometimes, on salad diet or juice diet, or something I don't know what kind of diet. But we will go on it together. So they would. They would invite. They would try to invite me because these vegetables I don't go on it. But sometimes I go on a juice diet with them. Sometimes. And then sometimes also we will have bento meals prepared for us from vendors, Vendors meaning like companies that are we are buying products from them, like the things to we use during procedure. So sometimes we get rental meals from them or desserts also. Even the doctors sometimes treat us some goodies la.

**NJCL: Then do you think there are instances where the workplace culture among nurses this is actually hinders your maintenance of healthy lifestyle?**

P2: Yeah, sometimes. Like, if I'm on like intermittent fasting and then I'm not like able to follow my timing like, for example, if I need to eat it at 12. So my, cycle is for 12pm. And if they make me break. Wait, it's not make me. If they assign me to go for 10'clock break, I can’t. I can't say no, because if I say no, then the plan for everyone else will be disturbed la, so the only way is for me to just follow, and then I just had to make it up by eating dinner earlier or later, depends on the timing. Then intermittent fasting is like the most- some more I’m Muslim. So sometimes every year we will have this fasting month, right? So our break would be used for what you call rest, and they would put us at the last break, since we are not buying any food. Yeah, so not eating the whole day, and then resting for like 1 hr I think I mean, it's normal already. Since young already, we've been doing that. I think there's the- depends on the workload that day, I would say.

**NJCL: Okay can. Okay. Yeah, let me try and summarize what has been spoken? So for you, the workplace culture among nurses in your workplace is actually okay, cause you and your colleagues go on like special diets, like salad or juice diets so like together, then you are like influencing each other. Am I right to say that?**

P2: Mm. Yeah.

**NJCL: Then for barriers, it's more of how like you. you cannot say no to the break times given to you. It's it's assigned to you, and you, you just have to stick with it- So that's why you-**

P2: Hmm.

**NJCL: Okay, can, thank you. Then building on that, what do you think the role of this managers in helping nurses maintain their own health?**

P2: What's their role? Hmm. For my hospital there's this union that that is the welfare and union, so they will organize some activities for us to to join. Depends on level of- fitness level ah. But there's yoga, there's there's badminton, or even bowling. All that they organize. If I'm not wrong every month there would be a thing. An activity. Yeah. So my, this manager is usually the person that is conveying that message to us, like forwarding to us that particular like brochure, so that we can see. And then, if you are interested, we can go. And then we are also quite close to like, all of us are quite close to our managers and our nurse clinicians. So we even sometimes go together with them. Yeah, to bond more, I guess. Yeah.

**NJCL: Okay. [long pause] Okay. And my next question is: how do you think your institution can further help you to maintain healthier lifestyle? Other than what you mentioned, which is the every month we organize some activities for you.**

P2: I hear other organizations or departments. or hospitals that they have food provided for their snack time, break time. Yeah, So I think maybe it's too much to ask, but it will be good to have it, since the only food that is coming to our pantry, I would say, would be healthy food. Like hospitals won’t fund unhealthy foods. So if there’s healthy food available, I think a lot of people will be more settled with what we have. Yeah.

**NJCL: As in right now, currently, your pantries are stuck with unhealthy food?**

P2: Oh, no, all pantries are empty. What we have is what we bring. Yeah. Pantry, in terms of break room.

**NJCL: Other than helping you stock the pantry with healthy food, is there anything else you you think can help nurses maintain their lifestyles?**

P2: Hmm. more, nurses. [NJCL: Participant smiles] More manpower.

**NJCL: Ah, manpower issue. Currently. Are there any like work place health promotion programs in your institution?**

P2: Uh, does vaccination count?

**NJCL: Yeah. I guess. Like vaccination for flu?**

P2: Flu vaccines. Yeah they keep us up to date. Yeah. I don't know what else.

**NJCL: Okay. Other than flu vaccine. That's all that you're aware of?**

P2: Yeah.

**NJCL: Okay. Then, moving on to my final question outside of work, what do you think influences your lifestyle?**

P2: Well. social media; how people are going about with your personal life outside of work. So, if I see something they are doing is interesting. I would have the feeling of wanting to try. My family, my mum, and cooks. So when I see her cook, I might want to- sometimes I might want to learn from her. Yeah, because after work usually I don't want to do anything. Because I want to rest. If it's something really nice, or I know that I love the the dish. I would try to want to learn. Yeah, but mostly it’s social media, because we follow everyone, and anyone can do anything with their time. So like, it’s a way to learn that there are other things to do.

**NJCL: [long pause] Currently, what kind of social media are you- do do you think will influence you?**

P2: Instagram.

**NJCL: Instagram?**

P2: Instagram, that’s the only platform I use. Yeah. Facebook is- I guess every one has it. But it's mostly for like family relatives. So I don't really look at like influencers all that. Yeah. Social media yes. I mean Instagram.

**NJCL: Okay, okay. Hmm. Do you have anything else that you would like to ask or speak about. There was not mentioned in the interview any further comments?**

P2: No? Yeah, nothing.

**NJCL: Okay. Then that marks the end of the interview. Thank you for your time and participation. We're done.**

P2: Okay.

-End of P2 transcript-

# **Interview #3**

Participant and interview details
 Participant number: 3 (P3)
 Date of interview: 20 Sept 2023
 Interviewer Number: Navarro John Christopher Lambino (NJCL)
 Time IDI started: 1555
 Time IDI ended: 1633
 Observational notes: Participant seems relaxed at the start

Section I: Interviewer’s summary
 The individual interview was conducted via Zoom and the participant appeared to be very relaxed, in the comforts of his office at work. Initially, the interview was supposed to start at 1600, but the participant delayed it until 1630. Other than the initial audio issues, there were no interruptions. I mostly agreed with the participant’s responses. I also believed in the importance of work-life balance and being able to disconnect and connect with work. Perhaps due to these beliefs, I did not ask further on how he maintains this balance. Key ideas shared by this participant is importance of mental wellness and self-compassion. As well as the fact that employee has a self-responsibility to maintain their own health. SPIRITUALITY also plays a huge role for this participant. Compared to P1, he mentioned that nurse managers/people in higher position have a responsibility to engage with the ground. As a clinical instructor with more experience compared to the previous 2 interviews, he might be able to understand the impact of how supporting nurses can help make for a healthier workforce. Similarly to other interviews, work-life balance is also a concern. Having something to look forward to outside of work is also touched upon. Lastly, I should summarize per section instead of everything at the end.

Section II: Transcript

**NJCL: Okay, can. Just making sure everything is working. Alright. Good afternoon Mr P3.**

P3: Good afternoon

**NJCL: Firstly I would like to thank you for taking the time to speak with me today. My name is Chris and I am a NUS Y4 Nursing student. This interview is to get a better understanding on perspectives that nurses have on self-managing their own health and how various factors may influence this. I want to find out what are your views, opinions, and experiences on this I would really like to encourage you to speak what comes to your mind. There are no right or wrong answers. May I have your permission to carry on the video record.**

P3: Sure

**NJCL: Alright, okay. Then uh, can we just start with some basic questions?**

P3: Yes please.

**NJCL: Tell me about your… which ward are you working in right now?**

P3: Okay I’m working in a psychiatric hospital. In Singapore. Which is [Public Hospital name].

**NJCL: Hmm. Uh, would you like to share more about like your specific ward you are working?**

P3: Sure, I'm actually a clinical nurse educator. And then my role is to just facilitate students. attachment for the 2 weeks. Once they come to make sure they meet their objectives. That's what is my role here.

**NJCL: Alright, thank you for that. And then I'm moving on to my next few set of questions.**

P3: Sure.

**NJCL: Can ah. Firstly, as a nurse, how would you define a healthy lifestyle?**

P3: That is a very tough question. How to define a healthy lifestyle. It's basically to have self-awareness. And it is all a little bit of intrinsic factor where you have to think about how to have to maintain a work life balance and how to make sure that you are mentally well at all times. Saying that, in Singapore it is not that easy. So what I do for myself normally, when I talk to students or talk to any of my staff. I always focus on mental wellness. With good mental wellness, there is not even a single physical illness. and I also believe in compassion fatigue, where most of them have gone through, probably going through and burn out. So, I always have this saying that calls you have to be self-compassionate. That is how I maintain my mental wellness.

**NJCL: Hmm! Could You elaborate more on your self-compassion?**

P3: Self compassion is uhh, hmm okay. The burnout rate in nursing is actually increasing. due to the demand and challenges. So, because of that, I'm also a stress management consultant. I find that most nurses do not feel sorry for themselves, knowing that they have to face a lot of emotional bombardment from various sources like family, nursing staffs, managers. coordinators, and even maybe clinical instructors. I won’t know. That has actually caused a lot of burnout turbulence for that. I always advocate even to my staff to make sure that you are mentally able to stay agile. And I myself, how do I manage? Is basically I go for a coffee, maybe 30 minutes to relax with a coffee one of the strategy. Second, maybe I'll go for bowling. Third. I go for some massage. And sometime I just go to the park and just walk alone by myself. Yup.

**NJCL: Hmm! I see. Hmm. Earlier you mentioned something about work life balance, and it actually being difficult in Singapore. Would you elaborate more on that point?**

P3: Okay, I believe that recently, I think our ministries, or you know, our leaders have actually been told to advocate work-life balance. Work-life balance is basically about having time for your family. I would say, quality time for your family as well as work. Now, work itself is not everything in life. I would say that every staff, everybody should be able to focus equally to work as well as family. So work, life and work-life balance is actually advocated a lot nowadays by most of the leaders, and even in nursing it is advocated even in every sector. I believe it is going on as try to have work-life balance, and I think they are doing a lot of support as well to support Singaporeans to maintain a work life balance by providing a lot of channels like healthy lifestyle. And HPB [NJCL: Health Promotion Board]. is coming in, and a lot of communities are engaging in a healthy lifestyle, and so on. That really helps, and it is going on, which is a good start off for some time.

**NJCL: Hmm, hmm. Then, going back to one of your points. You also mentioned about being mentally well is something that is having a healthy lifestyle. Can you elaborate on being mentally well?**

P3: Okay, there's a lot of kind of a taboo saying that physical illness is always comes anytime. Right? So, there is a saying that goes. If there is no mental wellness. Then there is physical illness. Without mental illness. You can never get a physical illness, so psychologically, emotionally, you have to go through a problem. A mental or mental uhh, psychological, emotional thing. Where actually you find that you are going through some form of stress. And then you did not find a solution or a way out of coping mechanism. Eventually you go into distress. and from distress you tend to go into mental illness. Now, mental illness in Singapore is 1 in 5. Depression, and it's actually getting a little bit more and does depression as supersedes ?[surpassed]? mental schizophrenia. Another mental illness. Apparently the mental wellness does keeps you going and keeps you healthy. So one of the focus in psychiatric which I believe to share with everybody is that no matter how, try and find strategy's scoping skills to keep your mental agility well.

**NJCL: Hmm okay. [long pause] Yeah, hmm moving on to my next question, is regarding- because, just how you already defined how healthy lifestyle as maintaining work life balance, being mentally well. As a nurse, how do you feel about maintaining such a healthy lifestyle?**

P3: Uh, for me, recently, post-Covid I actually go used to go to gym and do a lot of exercises. So currently, post-Covid, I am going for gym and do some fitness exercise, and I also go swimming. And I also go some form of nature. I'm a nature lover. So I actually tend to go to park and take a walk or breeze walk. So and on. And this actually are some of my ways of maintaining my healthy lifestyle and my health wellness.

**NJCL: Hmm. [long pause] So this the examples you give are about maintaining like your physical- uh physical fitness.**

P3: Yup. Yes.

**NJCL: Do you feel that you are having challenges?**

P3: Umm, there are always challenges around. The challenges are again you need to look at the intrinsic factor of what you have inside you. How you view your environment, how much you can put yourself in a positive note. That means you must have some positive thoughts. Okay, so you need to just tell yourself that. like for me- very simple. We go to work. You just have to fulfil 8 hours. You fulfil and go back- back to your family. So you have to disconnect and connect. I hope you can understand that. So when you come into nursing them, or when I am connecting into nursing, or maybe facilitating a student group after which, once my office hours or my duty is over, I go back disconnect. I go back as a son, brother, you know, husband or friend. So, you need to have that type of positive awareness, and always have these things going on in you every day, every minute of your life.

**NJCL: Hmm, hmm.**

P3: Answers your question?

**NJCL: Yeah. Then what do you feel like are your main facilitators in maintaining a healthier lifestyle?**

P3: My main facilitator? [NJCL: Participant seemed confused]

**NJCL: Cause just now you were mentioning, like mostly like the barrier, the challenges lah, so now is like what motivates you to maintain?**

P3: Motivates? Okay, one of the most important things in rather nursing is, you must have a form of compassion and passion. Now, once you have the compassion and passion there is around the corner which is compassion fatigue. And you must know how to have self-compassion. So there are some of them just come. They do their job, and then they go back. Okay. Maybe they lack compassion or passion, they won't be that much affected as those who are having compassion and passion. So that compassion to do something to some one who needs help, especially mental health issues, is a lot more challenging and meaningful. It is so fruitfully meaningful for me to get, I mean, have having engagement with my patients, or even with my students for that sake. So you have to continue to have that type of mindset. That is what makes me going all the way the last many years to come.

**NJCL: Hmm. [long pause] hmm, hmm. okay. And hmm. Then, moving on to my next question, how does your personal lifestyle habits impact your work performance?**

P3: Okay. Personal lifestyle is a very simple lifestyle. My lifestyle is simple. Come to work. Do my work. go back and forget about work. Once I go back I take over the family. Stand, and once I go back to family, focus on issues that are important. fulfil them and then go and have some time. My time. Maybe an hour or 2, maybe even see Netflix to ease your mind. And then eventually you are preparing for the next day, but make sure that you need to go to bed early. Nowadays. Students, they do a lot of instagram, Facebook. And all these things up to 12 o'clock now that is a new generation. And I wish them well. [NJCL: Participant is smiling as he answers] Hopefully, they do something about it. For me, I'm not a social media person. So, I escape that part of my life which uh gives a lot of you know- addiction, so called. Blehh.

**NJCL: [long pause] Hmm. hmm. okay. Then moving on to the second half already.**

P3: Okay.

**NJCL: As a nurse, do you feel that the work place influences your own lifestyle habits?**

P3: Workplace, have a lot to influence you because half your life is in working place and half is at home. So working place contributes a lot to your own personal lifestyle. It can sap a lot of your energy every time you come in with a good energy it can sap up because I am dealing with mental health issues, mental health patients. So, a lot of mental energy is depiciated ?[depleted]? Kind of like used up. So you can have mental energy green, correct? So, for that I have something which I always use is yoga, meditation, and some time on my own. And this yoga, meditations all really help me to be ready for the next day. If I don't have this spiritual awareness, yoga, and meditation, then I think I'll be- my personal life would have been affected.

**NJCL: [long pause] Hmm. hmm! Do you feel that the workplace culture among nurses actually helps maintain your healthy lifestyle?**

P3: [NJCL: Participant is smiling as he answers] That is a very serious and sensitive question. Intruding into that area. Okay, I will say. the work culture is another area. So now we have multi multi country, or other people coming from overseas and foreigners and all these things. So we need to understand. They’re culture. They call it cross cultural competence, I believe. You need to understand. And you need to learn to work with all generation. We got about 4 generations from baby boomers all the way to Strawberry and Durian and all this generation. If you don't understand and work with them, then I don't think nursing is a team effort or team player approach. So that part actually need to change. And we can't expect the whole thing of the whole country to change. But I think leaders have a very serious role to play to maintain that the working environment of their own organization are in check and given support to the people on the ground. So that is a very important part of maintaining or changing the culture to a more, uh wellness culture. Okay, things like open communication. Things like, uh uhh what you call interaction; engagement session with the the ground. All these things can actually slowly change a better culture. That means a culture that can actually be more sound and can help the nursing industry to work together as a team to fulfil the obligations of patients who are admitted to hospital. That is something that I dream of. And I hope it is going to it happens or it will happen one day and hope to see it happening.

**NJCL: Hmm, hmm, hmm. I see I see. Hmm. hmm! So, for my next one, I mean? I think you answered- answered it partially but how, how do you think your institution can help maintain a healthier lifestyle?**

P3: Okay, institution is actually- my institution is doing a lot. Get to help. the ground, or every really helped the organization by having fitness clubs. a healthy lifestyle. and then even promoting umm holidays where they actually have this.. I think one year they got about few $100 where they can utilize for travelling, and they also give out uh some cinema tickets like you know. And then they also engage them to bring them like cycling, and then they have gym, and they also organize more outings to get engaged with the staff, and then to see like how they can bring them to a healthy wellness, uhh. approach. Or stayed rather. And I've no doubt about my institution is actually really doing a lot, but it is up to the individual to take the opportunity to go. So it’s both ways. We have a lot, but if nobody comes forward to participate and make sure that they are mentally well, then, it is a choice that is given to the employee, correct? So that's where it plays the important part. Like for me. okay, we go forward. We have a gym. I go forward weekly, but I go to my own gym. But if there is a gym I would go. If I don't have a gym outside. So it all depends on the individual. And it is important to encourage and motivate them to continue to have these. Then, if possible, I like to see a family involved as well. The family of these employees are also involved in getting some support. Then it goes a very long. long, long way to make sure that the family is also mentally well, no? Mental wellness as well and healthy lifestyle.

**NJCL: I see. Hmm. Earlier, you also mentioned that nurse managers, or like the people higher up have a role to actually help maintain the health of like the employees, right? Could you elaborate on that?**

P3: Okay, umm if you look at work culture. And work-related stressors. It is not from just- it is all the way from the ground to the management level, right? So, the most important thing, the management and the ground should work in unison. Collaborate. And then make sure that what is whatever is going on the ground is actually keeping their, so called their population or their employees to the maximum wellness. Mental wellness, or health wellness. So if that can happen, then it will be a better situation for the organization to have a healthier population working in their organization. Basically, if we look at all these; a lot of of them, on maybe, go on medical leave, and uh alot of them fall sick. So, there's a very strong need to make sure that there should be something that can be done about it. So, there are areas for such areas to go in and look at what are the needs and provide the needs and not to forget, of course, we are aging population. As such. there is going to be a lot of all these elderly folks related illness or other form of disability that also we have to take into consideration to look at it in another- from another angle as well.

**NJCL: [long pause] Hmm, hmm. I see. Hmm. okay. Then, moving on to one of my final questions…**

P3: [NJCL: Inaudible mutterings in the background by C’s coworkers]

**NJCL: Outside of work, what do you think influences your lifestyle?**

P3: Okay for me ah. The strongest influence for me per se is spirituality. The belief that you are- there is someone. Higher. Higher entity where the spiritual support is one of the most important things because one of those components or dimension. When you look at holistic lifestyle approach, a total approach is made up of emotional dimensions, psychological, physical, physiological. emotional, social, and spiritual. We look at a Maslow hierarchy of law. It also looks at all these things, so not to neglect the spiritual part is one of those important thing to understand. So, nurses also should understand that if they do not know the cultural culture, value, belief and tradition of that particular religion, they are only half a nurse. So, if I do not know what is Hinduism, then I cannot manage a Hindu patient. If I do not know a little bit about Christianity, then I would not be able to bring the patient towards and understand the patient and bring along their belief. Now that part is very important, for nurses, especially nurses. Because most of them are coming from many, many parts of the world and if we do not understand that part of the culture of how they, what they feel mental illness is how they perceive, how they perceive, how they assume. All these things need to be actually worked; out ironed out. So for me, as a nurse. I learn about all the religion. I don't focus per religion. Spirituality, spirituality is something- a vast thing. You, yourself have to know that you have to fall in love with yourself. Once you love yourself, you can share the joy and the love with others. So, if you don't love yourself, you have your own concern. ?[partially out]? of love, then you cannot do, cannot do a very called, compassionate, passionate nursing that that's the angle that I always look for.

**NJCL: Hmm.**

Very difficult to advocate that part, because a lot of people do not understand the spirituality aspect, which helps a lot. All that time. Domains. Yes, but spirituality should be combined a little bit. There is such things that a spiritual nurse per se in overseas. So maybe you know, that may have, may come into this ah, our part of the in Singapore may be to look at the needs of uhh the patients. Because most of the patients they believe in their own belief, right? So, we need to go along with the belief and see how we can recover them. That's what I am coming to. Point.

**NJCL: Hmm. there. hmm.**

P3: Ya.

**NJCL: I see, I see. Hm. And this, moving back a bit, you mentioned something that one of your roles is a stress management specialist.**

P3: Yup.

**NJCL: Could you elaborate more on that?**

P3: Okay, I was trained by my hospital as one of the member to give stress management talk to Singapore per se. Anybody can just request [NJCL: participant’s hospital], and then they will actually send us to give a talk. I've been actually giving this talk for not the last 5 years, maybe 6 years. No, but previously, about 15 years or so, I've been giving talks to many places in Singapore like shipyards even hospitals, even like SIA, even like nursing homes, and some of those corporate companies to understand what is stress and the causes and how to cope. [NJCL: Inaudible segment. Participant accidentally muted.]

**NJCL: Hmm. hmm. I see, I see. Hmm. Could you maybe share a bit on the coping strategies?**

P3: Yep. Sorry. [NJCL: Participant unmutes himself]

**NJCL: Oh, sorry.**

P3: Yeah, can?

**NJCL: Can you hear me? Uh…**

P3: Yeah can.

**NJCL: Sorry, could you uh share a bit more on like some of the coping strategies for stress?**

P3: Mainly there- there are strategies for stress. You need to understand the basic thing about what causes stress. So, some of the research um evidence-based studies have shown that it is a very simple thing about understanding stress is; our cell. Each cell in our body need to be in harmony. Yeah, got that part? So each cell got some things like intracellular and extracellular stuffs, so if each of yourself is not in harmony, then your potassium sodium can go haywire because of the chemical imbalance that can be caused. So, if every cell is not in unison and in homeostasis, the chances from the cell, it will go to the tissue, tissue to organ, and eventually the whole body, and it will affect your mental agility or mental wellness. So that's where you need to make sure that you have to cope. Coping mechanism is one. There are so many: need to get a lot of rest, need to talk to some good friends to to ventilate. If you have something to talk about, or you can see a counsellor. or you can actually go for a walk, go for holidays. The main thing that I normally tell them is, go and do something that gives you pleasure. Something that gives you pleasurable, some form of pleasurable activity that you have forgotten to enjoy. So, all working population are working so much and not having enough time, or having creating enough time. which is one of our own self responsibility to go for yoga meditation, pilate, you name it, go to the beach, go for a swim, have a cup of coffee, go for a movie. All these are coping strategies which you can choose anything that you want. Like for me, well, I am very happy. My stress coping mechanism is go for a coffee. I love bowling, so go and enjoy bowling. so that particular 1-2 hours, your mind is actually relaxed. Here you are kind of a de-stressing. Then you come back. You're stress free. And then you look out for the next day, and then again, maybe one week, once have a my day. That means that particular day is meant for you. I encourage this [NJCL: Inaudible segmennt. Participant accidentally muted. Almost immediately unmutes.] So uh these are few things that I can value in that sense. And of course, I got more. But it will take some time to really explain [NJCL: Participant is smiling here.]

**NJCL: Yeah. Okay. okay. And then I will just summarize, uh try and summarize what you actually shared with me.**

P3: That’s a lot ah. [NJCL: Participant is laughing].

**NJCL: I'll try. So firstly, when I ask you how you define healthy lifestyle you already mentioned about having it in- the self awareness, the intrinsic factors on basically maintaining a work life balance, being mentally well and actually, you also shared on how like the mental or psychological emotional will lead to distress, and then mental illness. And then you mentioned that in Singapore it's actually not that easy to maintain such healthy lifestyle cause of the work-life balance being difficult. Uh, but then you also mentioned that ministries, or like leaders, are actually trying to push for work life balance- there’s more priority and work life balance now so that there's like more focus on mental wellness, reducing compassion fatigue, burnout. And I think, importantly, you also mentioned having the self compassion for yourself.**

P3: Yep.

**NJCL: Then, when I ask about the what are your main facilitators and barriers in maintaining healthy lifestyle, you mentioned that it's about the me- being mentally agile enough to actually manage yourself by going on like, at least personally, for you, you mentioned that you drink coffee, go bowling, getting massage, or like walking in the park alone. So, having the the mental awareness to go for this activities is like a facilitator for you.**

P3: Okay. The word facilitator was my role facilitating student nurses in meeting the objective. So that is the the coping skills that what I do to make myself relax. Of course, holiday is another one. Yeah, okay, go on.

**NJCL: Okay. Then, when I ask on, how does your personal lifestyle habits impact your work performance. you said that uhh, basically be be able to disconnect and connect.**

P3: Yeah.

**NJCL: Always be aware that, aware that you have this disconnect and connect awareness every day, every minute.**

P3: And being positive as well

**NJCL: And being positive Yeah. And you also mentioned that you must have the compassion to do something.**

P3: Yes.

**NJCL: Because if you have the meaningful engagement, this actually, uh would it be right to say it actually helps you perform better if you are…**

P3: Definitely.

**NJCL: If you are really engaged. Okay.**

P3: Yes.

**NJCL: Mmm. okay. Then, moving on to the oh, ah. Then, you also mentioned about the you, you personally having a simple lifestyle. So you are able to come to work. And and then when you go home you actually forget about work and be preparing for the next day. And you are not into social media that much. So, you're able to avoid the addiction of social media.**

P3: Yep.

**NJCL: Okay, then the second half is about the workplace. Then, when I asked you about, do you feel that the workplace influences your own lifestyle habits. You were able to say that it does because you- a person stays at work more than 50% of their life. Uh then you, you mentioned about having a mental energy drain at the work place. and that, for you, how you manage this is to go meditation having some time on your for your own.**

P3: Yes.

**NJCL: Then you also mentioned about spiritual awareness at this point.**

P3: Yep.

**NJCL: About workplace influences. Hmm. Then, when I ask you about the workplace culture among nurses in helping to facilitate or hinder maintenance of healthy lifestyle, you were saying that you must be able to understand the culture of others so that we- become a team player in nursing.**

P3: Yeah. Team approach. Yes.

**NJCL: Then you mentioned that the working environment needs to have a more wellness culture, which means open communication, more engagement and interaction from the top up with the bottom. Uh, this for the entire nursing interest industry that you hope to see happen.**

P3: Yep.

**NJCL: Then, when I specifically asked you about your institution. You mentioned that your institution is able to do a lot to help, but you also mentioned that the employee itself, him or herself, has a room to play, actually engage with this activities.**

P3: Correct.

**NJCL: And you also mentioned that the nurse managers they. They need to be working in collaboration in unison with the employees.**

P3: Employees and then also collaborate with the leaders. That means management team. Yep.

**NJCL: Hmm. alright. Then, for for your outside of work, what do you think influences your lifestyle. You're mentioning that the strongest influence for you is actually spirituality. And how nurses should be able to understand all all forms of religion, to be able to provide care. Compassionate care you, you mentioned like. in in other words, you mentioned. If cannot love yourself, how are you able to give out compassionate care for others.**

P3: Yes.

**NJCL: Oh, and you, lastly, also shared about stress management as stress management specialist.**

P3: Yes.

**NJCL: Okay. alright. Then that is all. Do you have anything else that you would like to ask or speak about. That was not mentioned in the interview? Any further comments?**

P3: I would say that, you know nursing is a very noble profession. So, there are a lot of challenges, demands by the population of patient. So is all about doing your best. So all of us have our best at different levels. Just do your best, be happy, give yourself a pat, and when you are leaving, going back home. put this work off your mind and then go back to family. Don't bring family problem to work and don't bring work problem to family. That is one of the strongest ways of keeping your mind healthy and being wellness, health wellness in that sense. That's about all.

**NJCL: And then this marks the end of the interview. Thank you for your time and participation.**

P3: Welcome. Thank you. Bye.

-End of P3 transcript-

#

# **Interview #4**

Participant and interview details
 Participant number: 4 (P4)
 Date of interview: 25 Sept 2023
 Interviewer Number: Navarro John Christopher Lambino (NJCL)
 Time IDI started: 1530
 Time IDI ended: 1633
 Observational notes: Participant seemed fidgety at the start

Section I: Interviewer’s summary
 The individual interview was conducted via Zoom and the participant appeared to be energetic. Initially, I thought her responses were rather quick to the point, and would require further prompting. However, as the interview progressed, she was becoming increasingly more forthcoming with her responses. She is a relatively new nursing graduate. She mostly used her time as a student as a reference for comparison with working life when answering the questions. Key themes highlighted were on the identity as a nurse and creating one’s identity beyond just being a nurse. She also mentioned how pay disparity plays a role, the difference between Singaporean nurses, and nurses who came from overseas to work in Singapore. The importance of family support and pay, distance from the workplace, all play a part in maintaining a healthier lifestyle. We need to recognize the ‘privilege’ of being a Singaporean nurse- e.g. living with parents and not paying rent, having someone to cook and help you prepare each day. There were no significant interruptions. This participant believes that nursing managers play a huge role in helping nurses maintain their own healthy lifestyles, unlike P1 and P2. Self-responsibility also comes into play here, as she mentioned about having formed the habits of maintaining her own healthy lifestyle has been done during her schooling years and it continued to her working life. Having activities out of work is also mentioned here again. Perhaps, I should have asked her more on what she thinks constitutes a ‘nursing identity’. Overall, a pleasant interview where participant gave ample inputs.

Section II: Transcript

**NJCL: Okay. Just making sure everything is working. Okay. Good afternoon Ms**

P4: Can

**NJCL: Firstly I would like to thank you for taking the time to speak with me today. My name is Chris and I am a NUS Y4 Nursing student. This interview is to get a better understanding of the perspectives that nurses have on self-managing their own health and how various factors may influence this. I want to find out what are your views, opinions, and experiences on this. I would really like to encourage you to speak what comes to your mind. There are no right or wrong answers. May I have your permission to carry on the video record.**

P4: Yeah, can.

**NJCL: Alright, can. So let us start with some basic questions. Which ward are you working in right now?**

P4: Ward? As in discipline, is it?

**NJCL: Yeah**

P4: General medicine. Actually, my ward also takes in surgical cases as well. So yeah.

**NJCL: Could you tell me more about your ward in general?**

P4: What would you like to know? [NJCL: Participant laughs]

**NJCL: Like, maybe like, what kind of work do you do in your ward?**

P4: Okay, I’ll try. [NJCL: Participant is fidgeting with a pen] Okay so, basically.. The ward census is about 39. We have three rotating shifts. Take care of medical, surgical patients. I usually spot 10 surgical bits then the remaining would be 29 right? So 29 would be medical cases. Yeah, so basically this will take about 8 to 13 cases per shift. A day shift would usually be 8 to 10. Then a night shift will be 10 to 13, depending on how the staffing is like. Yeah. [NJCL: Participant laughs]

**NJCL: Alright, I see. Then, moving onto my next set of questions. Regarding like healthy lifestyles and everything. As a nurse, how would you define a healthy lifestyle?**

P4: Personally, I don’t feel that being a nurse is my only identity, right? So, I would very much prefer a separate life outside of nursing. So work life balance is super important to me. I exercise 3 times a week. Yeah. I do gym. I do pole dancing. I also do horse riding, like on occasion. But for commitment wise right now, it’s super difficult to be able to ride as regularly as I used to. Yeah, so usually it’s about maybe once every 2 weeks. Something like that. Healthy lifestyle. I mean healthy lifestyle to me is more like, you know, mentally and like physically. So I have to be able to do the things that I want to do outside of my job. [NJCL: Participant nods and laughs]

**NJCL: Just now you mentioned that there’s a change in the frequency that you get to go horse riding. So how did your lifestyle change after you started as a nurse?**

P4: Back when I was in school, because. I was in Year 4. So Year 4 was work from home at the point of time cause of COVID? So after the restrictions lifted, I actually had a lot of time and leeway to plan my own schedule and basically, what I did was, I actually travelled to Malaysia to ride. Firstly, it is, I guess, cheaper. [NJCL: Participant laughs] And also the environment there is much bigger, bigger than the space that we have in Singapore. So, yeah. That’s what I meant when I say that I ride less. But here actually, there are equestrians here in Singapore, but those are more expensive.

**NJCL: You also mentioned you go gym, do pole dancing. You think the frequency has changed in any way after starting work?**

P4: Yeah, for sure. Right now, I actually do pole dancing classes at least once a week. Right. Previously, when I was still a student, I actually do, maybe 2 to 3 classes a week. But because of scheduling issues, because you know it's not just your schedule that you have to care about. You have to be in consideration of the ward schedule, other people's schedule as well. So currently, I only keep one class per week when my bosses are quite kind. I guess. They let me take off the same day every week. I know some wards don't allow that. Yeah. For fairness and whatnot. But as much as possible they try and give me off on the same day every week. Yeah. So, definitely, the chances of me being able to take classes definitely decreased. But then I just practise on my own. Same for gym actually. That’s why gymming became more like my current choice of doing. Cause I can do it any time of the day. Like, today, I just went to the gym.

**NJCL: Okay. Earlier, you were mentioning, like do you think healthy lifestyle is more mentally than physically, along those lines. Could you elaborate more on that?**

P4: I don’t think it’s more mentally or more physically. I think it’s a balance of both. In a sense that, as a nurse, you face a lot of things at work that could affect you mentally. For example, patient’s demands, doctors, demands of family, patient family. You know you have to be able to take what they dish out, I guess in some sense, because, you know you have to be understanding. They are sick, you have to empathise with them. Sometimes they are very worried about their family. So sometimes they say things that they don't mean to say. But you know, as a nurse, and more so as a person, you feel bad when you hear these kinds of things, right? So you have to be able to discharge these emotions. You have to be able to redirect the negative energy, the negative feelings into something else. And I mean, obviously you can’t be shouting back at your patients right? [NJCL: Participant laughs] Right. So that’s what I mean when mentally, you have to have an outlet.

**NJCL: Okay. Then, just to quickly summarise what you’ve been sharing. To you, a healthy lifestyle is having an identity outside of being a nurse. Being able to do things that you want. And also being mentally resilient.**

P4: For sure.

**NJCL: Okay.**

P4: And I think also, additionally, I mean I did mention physically a few times. I think it’s very important. Because as a nurse right, it is a job that is very physically demanding, especially at least for my cases. I meet a lot of patients that are wheelchair bound. You have to have a certain amount of physical ability. I mean you have to be physically fit in order to be able to care for the patients. You know like, doing sponging, doing diaper changing, transferring, you know all that takes a certain amount of, certain level of physical fitness for that to happen. And it would be very, you know, not very good for the patient, you know. If the nurse is not physically able to transfer them from chair to bed, bed to chair, especially when it’s supposedly beneficial to the patient. And, I mean, I personally feel that I don’t want to be the nurse that I can’t give them a better quality of life because I’m physically unable to meet the demands. Right.

**NJCL: Hmm. Then onto a related question, regarding specifically diet. How do you feel about your dietary habits? In your current lifestyle.**

P4: So I feel like it’s definitely changed, because shift work really messes with your schedule for sure. Like for example, I mean, I eat breakfast at 5 or 6, just to meet, you know, being able to go for morning shift. Next day, afternoon shift.. Sorry, in morning shift, I would have lunch at around like, 3 to 4 o’clock? Yeah. Which means that dinner gets pushed back or sometimes I skip them altogether. For afternoon shift, before I start shift, I start eating brunch at like, 11, maybe dinner at 7. So, the afternoon shift is a bit more normal, I think. Yeah. And then, night shift, because you sleep in the day right? So you definitely.. Messes you up a bit more. I think it has changed my dietary habits, in terms of the timing, right. But I think it’s also finding regularity in irregularity, you know. I do eat at a fixed time every, most times. So, if I do wake up for morning shift, I’ll definitely be eating breakfast. And then, I’ll definitely eat before shift, and in during shift, I will do my best like, to get a quick bite, if not, have a whole meal? Yeah. Something like that. In terms of the type of food that I eat, I don’t think it really changed much, actually. I think it’s super important to eat breakfast. Yeah. I still try and have lunch. Sometimes, if I skip dinner, I may or may not have a little snack. [NJCL: Participant tilting head] In place of, like, a full meal. Yeah.

**NJCL: Okay. Hmm. Then, with reference to what you were saying about a healthy lifestyle. As a nurse, how do you feel about maintaining such a lifestyle?**

P4: Hmm. I mean, it definitely takes a certain amount of determination on my part. [NJCL: Participant laughs] Because I definitely have colleagues who.. They choose to not exercise, or they choose not to pursue other interests outside of work. Because, I mean, you have to admit that it is very tiring. The job is very tiring, you know. At the end of the day, at the end of the shift, you just wanna crash, you just want to lie down and you know, scroll TikTok maybe. [NJCL: Participant smiles] But I feel like, I mean, while I do scroll TikTok sometimes and sometimes I do want to rest, you know, I feel that having the personal discipline, like the self-discipline to make it to the gym after work, before work, it really gives me more endurance. In the sense that like you know, when I first started working, probation was hell for me because like, trying to juggle gyming, and working, and then still having to like, find time to sleep. Yeah, it was quite problematic at first. But I think I’m balancing that quite well right now. Yeah. Yeah, I think it’s all about discipline really. Like, you have to want to do it, you have to value certain parts of yourself enough to, you know, show up. Yeah. [NJCL: Participant laughs]

**NJCL: I see. Weird cycle.**

P4: [NJCL: Participant laughs]

**NJCL: Then, what do you feel are the main facilitators/barriers in maintaining your healthy lifestyle?**

P4: I presume that you mean healthier lifestyle means in terms of like, food, in terms of like, mentally and physically, right? [NJCL: Participant gesturing with pen] I think a lot of it is like being able to sleep. Like another thing outside of those is also being able to sleep. Because if you don’t rest well, it will become a barrier. Because, you don’t even have enough energy to show up at work, right. How are you going to be able to, you know, want to gym after work. You’re so tired, you just wanna sleep, right? So I think.. Actually, I maintain quite a regular schedule for sleeping. Yeah, I mean, barring P to 8, which I sleep like, maybe 12 to about 5:30. Actually a regular schedule in a sense that if it’s morning shift, I sleep at 11 to hopefully 7 o’clock everyday. Unless I have to show up for morning shift. And then it will be like, 5:30. So sleeping at the same time everyday actually helps. Yeah. And then, I think another barrier I guess, or like facilitator. Hmm. Let me see.. [NJCL: Participant staring off to the side, fidgeting with pen in hand] I must say, I have really good family support. In a sense, that like usually, I’ll pack food to work because my workplace doesn’t really have places where I can buy food. So that becomes.. To some of my colleagues, they actually just skip a meal because rather than you know, having to go outside and buy food and then having to like order Grab or whatever, they just choose to not eat or they eat after the shift. So for me, my family actually helps me prepare my food to work. Yeah, so I think that that part helps a lot. Because like, no matter what I wake up, you know, I always have lunch to bring to work. Yeah. [NJCL: Participant smiles] So family support.. Discipline, wanting to look good, feel good about myself. Yeah. [NJCL: Participant nods] Yeah, I think that’s most of it.

**NJCL: Okay, then I’ll just summarise what you’ve been sharing so far. So for you, you feel that your main facilitators is mostly discipline. Having good family support, so they can always bring you meals to your workplace. Then-**

P4: Oh no no, they pack me food, and then I bring to work. It’s just that you know, like if it’s dinner that day, then I will pack the leftovers then that will be tomorrow’s lunch.

**NJCL: Ohh ok ok, can.**

P4: But at least I have food, you know, I don’t have to like, hunt for food, you know that kind of thing.

**NJCL: Alright, okay. So discipline, good family support, these are your main facilitators. Then, barriers, it’s mostly fatigue, like getting enough rest so you’re too tired to engage in activities outside of work.**

P4: Yeah [NJCL: Participant nodding]

**NJCL: Is that all?**

P4: [NJCL: Participant fidgeting] Yeah, I think it’s also like your own mental state actually. Because if you don’t feel good about yourself, before you start work, or you know, during, starting work, all that stuff. Like whatever people say or do will affect you personally. But I think it’s, I mean, we always say right, not to give personal leave, but sometimes it’s so difficult to do that. Right, because, you know sometimes they say things that aren’t really nice stuff. Being able to maintain that mental state and be like, it’s okay, you know, like, they are just seeing this because you know they’re not feeling well, they’re not feeling good, it’s fine. You’re there as their nurse, just give them support that you can give. You don’t have to do more than, you know, what you’re able to give at that point in time, but you know. You can always come back to them, talk to them again. Yeah, I think having your own like, mental stability is very important. Yeah. Something that I trained up when I was in school, I think. Yeah. It’s very important too. You know. I think that nowadays, I think my workplace has this thing, where they do this program called ‘We Care’, where basically, it tells you to do is just meditate, you know. I think we have this in NUS as well, right?

**NJCL: Not that I can recall at the moment.**

P4: Oh okay, so they were pushing this thing about like mental wellness, like back when I was in school and basically it was like meditation, I mean, I don’t really meditate. [NJCL: Participant laughs] So like, you know, like being able to redirect, you know, the mental focus. Being able to... Yeah. I think like I said, discharge the negative energy. I think that’s very important as well. Like, in terms of like regaining healthiness of like your own self. Yeah.

**NJCL: Then, personally, how do you maintain your mental stability? Without like, meditating, like what you said.**

P4: Okay, I feel like I may have said something wrong in terms of that because I feel like meditation comes in all shapes and forms, right? I mean, what I meant is that I don’t sit down there and like say for 15, 20 minutes and like meditate, right. So. Sometimes I read books. Yeah. So, reading does help me. You know, like, learn more things, absorb more, new information. Sometimes, there was a period of time where I was interested in like, coding. I tried to pick up coding Python. I think that helps in the sense, that like, you’re redirecting energy. I think the main focus is not really that you sit in there and absorb the negativity. It’s more like, you know, you redirect your attention somewhere else. Yeah, I think that’s what I meant by that.

**NJCL: I see. Okay. Then, moving on, how do you think your personal lifestyle habits impact your work?**

P4: Oh, definitely, it does, right. I think. I think people tell me that I am a very cheerful person at work, but I am.. [NJCL: Participant laughs] That’s a work persona. Yeah, I mean the thing is like. I feel that, you know, in order to help your patients, you know, feel better about themselves, you can’t, you can’t be negative, right? You cannot show them that, you know, it’s a dreary place, you know, they are here, they are forced to be here, you have to listen to me because I’m the nurse and you are sick and you’re a patient. So, I think. All in all, actually, I think being able to maintain what I have as a person outside of nursing, helps me be a better nurse. I feel like I’m a better listener. I give patient choices. Okay, this is another thing, where like, when I want them to do something, you don’t tell them that. Oh you need to do this, you, you let them pick like, two options, which both of which you want. [NJCL: Participant laughs] So do you want to take the medication now or later? Like 5 minutes later, or now, you know, something like that. So I think, it helps in a sense that you know. It’s like gentle parenting for people older than you. [NJCL: Participant laughs]

**NJCL: Okay.**

P4: Yeah, so I think I become a more patient person, or at least like my workplace persona is more cheerful. I am more patient with them, I explain a bit more, you know. Sometimes like, I just feel like you just wanna know what’s happening. So I take the extra time to explain, extra 5 minutes, they want something, I will get it for them, you want ice water. Oh god. Someone asked for ice water in the middle of the night like 3 times. And where am I going to find you ice water, but yeah, cool. Oh yeah, all that stuff. You know. As much as I can do I will try and like, give it to them. I think it makes me a better nurse in that sense. I treat them like a person. I want to treat them like a person. Because, I feel that I am a whole person and therefore you must be a whole person as well. More than a patient, more than enough. [NJCL: Participant smiles] Yeah.

**NJCL: Okay. Then, is it right for me to say that you having a work persona.. Which is then, you as a person are quite different, so that this actually in a sense influences how you work.**

P4: Hmm. I don’t think I’m a separate different person in that sense. I mean, it’s not so much like a split personality but more like a shield kind of thing. Because like, when I step forward and I have my work persona, I think I take things less personally. And able to take more feedback, criticism.. I will do more for them, and I would feel that, you know, it’s because I want better for my patients. It’s not really because, you know, it’s like, a complete different. I feel like my personality doesn’t change, it just gets like, enhanced in some sense. Yeah, that you know, you make your empathy bigger, [NJCL: Participant laughs] your patience a little bit more something like that. Yeah.

**NJCL: Okay. Hmm. Alright. Then, being ready to the second half. it's going to be regarding the workplace in general. As a nurse, do you feel that the workplace influences your own lifestyle habits?**

P4: Yeah, for sure, for sure, for sure. Like I think I mentioned earlier, right? I have a quite understanding, boss actually. Like, when I sit there like, you know. please, can I have like one specific day of week? I want to do pole dancing. you know, like they give it to me. I think that's very important. Because I do know that some of my friends, right, when they do make such requests, they get turned down. And yeah, like, in a fairness of like, everybody, like everybody should be like doing the same, receiving the same treatment, and therefore you shouldn't get one specific day off, you know, accounting. Think it's very important to have like, support from your bosses. In terms of like things you want to pursue. I think I'm very grateful that my boss understands that I am more than just a nurse, and more than just like a subordinate. I'm more than a member of the Board. I think, like the work culture really helps. You know, you develop outside. I do know friends who are doing like bachata outside. So, you know, like being able to pursue your interest outside, you be a better nurse I feel. Well, always helps you be a better worker in that sense. Yeah, of course, the the thing that everyone experiences which is like shift work definitely changes. You know how you see things that like. For example, weekends are never weekends for me. Weekends are every time I have off like today. Today's a weekend to me. But you know, you go outside, everyone's else is working. I think that changes a little bit on your perspective and like perception of the 9 to 5, the Monday to Friday thing I have friends who are like, Oh, hey! It's the weekend! A long weekend. And then to me it's like. What's a long weekend? Yeah, but my long weekend is 4 days long, you know, like, you know, if they are kind enough to give me 4 days straight, I do have a long weekend, but you know, the long weekend spans from Monday to Thursday. Yeah. Think perspective changes. Definitely support in the ward, culture. Yeah. It does really change your view on life. Hmm.

**NJCL: I think you really touch upon what I'm going to ask next, but how do you feel about the workplace culture among nurses in helping facilitate/hinder maintenance of healthy lifestyle?**

P4: Oh, yeah, I did want to bring that up also. I feel that, I mean, in comparison to my peers. I think my company really does a good job on emphasising the fact that, you know, you can pursue interests outside of the workplace. Uh. Perhaps the individual bosses do make a difference in that sense, because I do presume that there are people there who do not accommodate for such changes, but having a good working culture with your bosses that are supportive of like, pursuing your interest outside. That's definitely helpful. And also yeah, I, yeah, I don't know how else to elaborate this. But yeah, it really makes a huge difference in terms of like, because if you're so tired at work, they're not understanding, I don't think I'll make that step to, want to, you know, pursue my interest cause I can't right? Like work is already in the way, I'm already tired, people don't say yes to my request. Yeah, I think, I think it, I think it changes a lot.

**NJCL: Okay, how about between nurses? Like the same rank nurse and the same rank nurse, and how they interact in the world. How- How does this influence your own lifestyle habits?**

P4: Hmm, if I get you correctly, you mean, like, colleague relationships, is it?

**NJCL: Yes. Yes, exactly.**

P4: In between, say, staff, nurse one, or or in general, just off nurses?

**NJCL: Yeah. Let’s say in between like, staff nurses.**

P4: Okay, uhm. There's definitely office politics, right? I mean, where there's people, there will always be drama. [NJCL: Participant laughs] Uh. I'm very grateful that I have a really good mentor. Even now, we're- we're still kind of friends. As friends, as friends, as colleagues as can be, right. When I have like, it really helps when you know, you put yourself out, to be willing to learn. Very helpful. I think people appreciate that. And firstly, you have to be able to take that step to- form the bond, right? Yeah. So back, when I was in probation, I tried my best to form good relationships with my colleagues. And in turn, I think most of my colleagues are okay with me. Yeah, I wouldn't want to presume that, you know. We’re like best friends and chummy and all that right, but at the very least, uh. You don't tell the catch my woke. At the very least, if I need help, I know that they will answer the call. Yeah, I think that's very important. I do know of colleagues who are isolated, and that would be very detrimental to, I suppose, your mental health as well. Because as a nurse, you take on not just the job scope that's prescribed to you, or advice to you back when you're in school, you actually do more things that not prescribe to you. For example, helping physiotherapists call patients for therapy training. It sounds very simple, but it is another load on your mind as a nurse. Another one is patient family asking the doctors to call them back, but the doctors refuse to call them back. Then it's your part as a nurse to, you know, stand up and advocate for your patient, and say that ‘You know what, they have not been outdated for the past few days, and I've been looking at your notes so like I'm watching you. Call them back,’ you know something like that. [NJCL: Participant laughs] And I think if your colleagues can beg you. And you know you ask a colleague you'll be like, “Hey, uh? They haven't been calling like the family for the past 3 days, and that makes me like, really anxious like, how can I put it to the doctors?” And if you're clicking, they give you good advice, or they're willing to give you advice. And instead of like, ignoring you or like telling, ‘oh, you just call yourself’, you know. I think that helps. Because, you know, having that kind of support, having a validation, I guess in some sense like, it really helps. So yeah, I think colleague relationships also play a part in like, being healthy in that sense, mentally healthy. [inaudible]

**NJCL: Yeah, I'll- I'll just summarise what you've been sharing so far. So, for the question about ‘how the workplace culture among nurses actually help to facilitate, hinder the maintenance of healthy lifestyle’, some facilitating points actually having bosses that are very understanding of situation. You also quoted having a good mentor as a support for you. They also mentioned that having colleagues who can back you up, give you good advice. This actually helps maintain a healthier mental state. The nurse in mission. Then I'm guessing for the hindrance, or like, barriers in maintaining healthy lifestyle in terms of workplace culture, is the office politics that may- may be happening in the world. Will you elaborate more on how this office politics can influence?**

P4: I mean, I adopted the “Egg will live longer”, [NJCL: Participant laughs] [inaudible] mentality, right. I mean, I definitely know when someone's having beef with someone else. And sometimes they rope you in right. They’ll be like ‘Oh, I don't want to help this person, because they didn't help me the last time. You shouldn't help them as well’. So what- is like to me is like, okay, I won't help this person in this aspect, but you know I will help the person in another situation. You know something like that, “Hey. So sorry. I can't help you in this thing, because I'm so busy. But it's okay. I prep this thing for you. On the site, you know”. So so I think finding a balance of like you know, staying in everyone's good books is, is- is very important, and if you don't want to be involved in office politics, I mean, okay. I cannot say for sure that people are not oh, involving me unknowingly in office politics, but at the very least I feel I shouldn't be participating in office politics. I mean.. The job is a means to an end right? In very blunt terms. The job pays me. I get the pay. I do my best for my patients. I leave the job, and I pursue other interests also. [NJCL: Participant laughs] Alright, so uh, I do not feel that I need to put in, you know, mental energy, or take on the burden of involving myself in office politics, I think. Yeah. But I'm pretty sure there is

**NJCL: Okay, other than office politics. Is there any other barriers you can think of in, in terms of like workplace culture and maintaining healthy lifestyle?**

P4: Actually, you did mention staff nurse to staff nurse relationships. Right? Actually, your assistance actually plays a humongous role in whether or not you have a good shift. I think. Because I think we're talking about like ENs or locums, or people you know, part- part of everyone who goes by in your cubicle, in, you know. in taking care of the same people they are taking care of. If they are reliable. and they know what they need to do. It will make your job infinitely easier. Because currently, my job takes in locum ENs. Basically, it means that we- we internally do not have enough uh, ENs, and therefore we have to hire outside. That also means the training is a little bit different for them. They may or may not know.. For example, vital signs. What are normal vital signs.. when the vitals are normal, you need to inform the staff nurse right? So there are cases where a figure of 39 does not get raised to the staff nurse. Like, if the patient's hired having high BP in the 1 90 s. And the staff nurses don't know about it. So what this means, is as a staff nurse, you have to have an additional mental burden of having to check everyone's vital signs. Everyone's BSL, you know. Make sure that everything's within range. Everything is fine. The patient is fine, clinically. And all this stuff. Right? Yeah, I think I think it's very important to have a good relationship with your assistants, and also have capable assistance as well. Because if you have too much to govern. Sometimes you let things slip, relationships with other types of colleagues… Could you remind me what the question was again? [NJCL: Participant laughs]

**NJCL: The question was, how do you feel about the workplace culture among nurses in helping to facilitate/hinder the maintenance healthy lifestyles?**

P4:Okay, so like colleagues, and all this stuff.. Timing. Yeah, I think I covered most of it. I think, as far as I can. You know, come up with.

**NJCL: Okay, alright. And we can move on to the next question.** **How can your institution help to maintain a healthier lifestyle? How can your institution help to maintain-**

P4: [inaudible]

**NJCL: How can your institution help to maintain a healthier lifestyle?**

P4: [NJCL: Participant laughs, adjusts in seat] Okay. So basically, because I'm super booky right, I'll read all my emails and I'll find out about these things. So my, my hospital actually has this thing where they have this thing they buy monthly. So once every 2 weeks they'll come up with some kind of exercise thing. Sometimes it's Zumba, sometimes it's Pilates. Sometimes I think there was Yoga. There was some aerobics thing as well. But the thing is, you know. Of course, the option of having it there helps the nurses, you know. Be aware that, you know there's something that we're making an effort. But the thing is when you're on shift for usually the time does not matter, and sometimes the timing is like 730 AM. You start shift at 7 right? So like, 8. So if you start shift already, how are you going to attend these classes? Right? So maybe maybe they're not meant for nurses? I- I'm not sure but the fact is that you know they they do try to. Encourage you to have a healthy lifestyle. There's an in campus gym. I guess there are people who use the campus gym. I think I think the fact that the facility is there is also a reminder, for instance, to have a healthier lifestyle, or whether or not you use it as another story. Right? Yeah. I think, at least at least for physical activity-wise. There is that. Another sad thing is the thing about diet, right? My hospital does not have food. [NJCL: Participant laughs] I think currently, our cafeteria only has 3 stalls. Yeah the food options are not great. They try to encourage healthier eating. But when there's no diversity. People tend to eat Mcdonald's, you know. Picking things that aren’t, you know, a healthier choice, that kind of thing. I definitely think that would affect like, does a diet will affect like, your healthy lifestyle. You know the fact that having choices would give you better options, and therefore you make better decisions. Right? So I think that that would be a barrier. So food, and then also exercise. Ah, yes, so my hospital did this thing where all morning shifts now starts at 8 instead of 7. Yeah, sweet, of course you end 1 hour later. Than the rest of your peers. But the fact is that when you start at 8. I feel that you'd be better prepared to enter shift. Of course, it does mean that your night shift will help to give morning medications. It will make night shift a little bit more taxing. But the exchange is that you know, you're on rotating shift. So you do get the benefits. You do night, and you do morning right? So you do get a benefits in the sense that morning shift is slightly not so hectic. Yeah. I think that was a good move. TBH, Actually, I was so on that before I joined the hospital. [NJCL: Participant laughs] I heard about it and I was like, Hey. [inaudible] [NJCL: Participant continues laughing]

**NJCL: Okay. I see, I see.**

P4: Yeah.

**NJCL: Okay, just to summarize what you've been seeing. You feel that the institution actually tries to- to encourage nurses to actually go for activities, for physical activities. And then you also mentioned that your institution has limited food choices in terms of diet. It's not really good. This also, like institutional change where you mentioned that they change the morning shift to start at 8 AM. Is there any more that you want to add on?**

P4: Hmm. Can't think of anything at the moment. But if I do, I will bring it up.

**NJCL: Okay, no problem. Okay. Then, earlier, you touch upon this already. By saying, like, having supportive- supportive and- and understanding nurses actually help to contribute to your mental state.** **Can- Can you elaborate more that on like, what do you think about the role of nurse managers in helping nurses maintain their own health?**

P4: Oh, sure, big, big difference. So back when I was a student. I think I was like. What's the thing where we did about fourth year? Ah, CCP. Okay, so I was in the same hospital because I was bond there right? So so in a different ward in [Public Hospital Name].. Man, if if I went back to that ward, I would quit my bond. [NJCL: Participant laughs] So basically, I actually observe like this. So a patient had a skin tear that only the afternoon shift found out. Presumably it happened in the morning shift. Perhaps during showering. The skin tear was at the back of the calf, so it's very difficult to see, especially when the patient is lying down a bit. So you know you do rounding right, and when you take over, so I suppose the afternoon shift made some checks and they found the skin tear. Uh. So this EN, went to inform the nurse manager of the ward. And I don't know what happened. I wasn’t in the conversation, I was walking by, but right at the corridor, the nurse manager was shouting at the nurse. Uh. “How can this have happened on your shift? How is it that you're only reporting it now?” But the thing is, the nurse was definitely the afternoon nurse right? And as soon as they found out they reported it. And I think in that ward, there's a culture where they are afraid of reporting mistakes. And I think that's very dangerous, right? We learn in school that if something happens for the sake of patent safety. You have to advocate for the patient. Something happened immediately. But better late than ever. Right? I think that's very important. In terms of like bosses. If they instil a sense of fear, to report mistakes, unintentional mistakes. Right? I mean. who goes around saying like, “I want to give my patient a skin tear”, or like, “I want to give my patient the wrong medication”. Nobody does that right? So the thing is that as bosses or as nurse managers, you have to manage your own expectations of the nurses that are under you. And I think it's very important to, hmm. Build a culture where reporting mistakes is reporting mistakes. Right? Everything is for the sake of the patient. You want the patient to be well, you want to rectify it. You don't wanna wait until the patient, touch wood, passes away. And then we do autopsy and then we find out, hey, it's a nurse's problem, you know. If something happens. You want to know it now, you want to know it fast. Think that's very important. So in stark contrast, my current bosses do advocate for the patients, in a sense that if something happens but most definitely strict, right? She would be like, “Oh, how did this happen? Go write reflection.” And you need to like, reflect on this thing. And everybody needs to know about this kind of mistakes, and everybody reflects together in accounting. But at the very base, or at at the deep end of it. There's no blame culture. It would be more like. “How did this happen?” “Why did this happen?” And in terms of like RAMs, right, like risk assessment. How can we prevent this from happening again? And that's more important than finding out who did it. I mean, of course, you know who did it right. I mean, you trace it, and you know. But it's more important than this. How do we make sure that everyone does not make the same kind of mistakes or similar mistakes. And if they do make similar mistakes, is there any way we can rectify it or prevent it. It's very important. I think the attitude that the bosses take towards their- their nurses is very important, rather than you know blaming, or, you know, trying to find a culprit. I think finding the source of the problem might be more beneficial. Yeah.

**NJCL: Hmm, hmm. Okay, moving on to my final question. Outside of that, what do you think influences lifestyle?**

P4: Outside of that would influence my lifestyle? Uh. I think a large part of it is a personal factor, right? Uh. Personally, I think that I mean, it's like a value system, right? I think that I am a person. Therefore my patients are a person. and therefore I want to treat them like a person, right? So that's why I want to build an identity outside of nursing, outside of the fact that you know we introduce. I mean, I saw on TikTok once right. They were like, “How will you introduce yourself without mentioning your age, where you study, what you work as, how you introduce yourself?”, and I think that's something that stuck to me. I- I don't want to just be a nurse. And if I want, if I'm going to be known as a nurse, then, and I want people to remember me more than you know, The Nurse, right. I think, that really influenced how I treat my life, and therefore how I treat other people's lives. That's like, in terms of like personal belief system. Another thing is, I think I'm a very active person back in school as well. So I think back in JC, I was doing judo, I was in UG, I had multiple CCAs, even in uni. I did not give up judo, I was in. I was still in UG, and now on top of that I picked up pole dancing, and now I do horse riding. So all of that form the basis of the person I am today, right cause. I don't want to give up the things that I have in my head. And as much as I can I will double all of this stuff. All of this interest. Including now the new thing, which is working. Yeah, I think maybe being greedy [NJCL: Participant laughs] is what influence me to, you know, have the kind of active lifestyle I have right? Because I don't want to drop a single thing. Yeah, and also, I think, wanting to be healthy, physically at very least. Uh. Was something that I held close to me, even as a student so definitely would have carried to working. I do know of friends who do not develop a habit to exercise regularly when they're in school definitely, when you go to work, you wouldn't want, because, firstly, you're really tired. And then, in order to work, and then still pull out extra effort to, you know, form a new habit of which [inaudible] right? You've got to bring yourself to the gym. You got to put in more effort to like, plan what you're going to do. I think that would have a barrier in that sense, because you don't already have a habit, or have an ideation of how your life wants to be back when you're in school. Then when you're working, it's like, free from all that. Right? So you you are in control of your own free time. Yes, then people tend to follow the habits that they have. Umhm.

**NJCL: So is it right to say, because back when you were schooling, like, personally, you already had the habits, the personal discipline. So this actually continued onwards to your working life?**

P4: Yeah, definitely. Yeah, for sure, for sure.

**NJCL: Okay. And then do you have anything else that you'd like to ask, speak about, that was not mentioned in the interview?** **Any further comments that you want to share?**

P4: Uh. I think I wanna elaborate on like family support as well. I mean, I did. I did briefly mention about family support there. They're cooking for me. And all this stuff, right? I think another barrier to healthy, active lifestyle, right? Because, you know, when you know in your ward, you definitely work with people who are not local. I mean, I'm- I acknowledge fully that I have the privilege of living with my family. They cook. I benefit, you know, they cook, I eat, and I get to daobao to to work. But I think it's when you're living alone. Or when you're living, you know, outside on your own. You do not have the family support. It's actually really difficult to meal plan right? It’s an extra step. I don't plan my meals. I just eat what's on the table, and you know, taobao it. But a meal plan- and meal planning would also entail the fact that you have nutritional knowledge. You know what healthier choices to make. You are aware, barring your own culture, because you know, in Southern cultures do have a slightly, I guess in a general nutritional viewpoint, slightly less healthy options. Right? So if you don't have the nutritional knowledge, it's so difficult to be healthy. And then also. That's on the nutrition side. I think another one is, especially when you're living alone outside. How? I mean, money theory wise right, I'm lucky I'm staying with my parents. I pay no rent right. Having to have to separate your paycheck and have to accommodate for rent, to accommodate for a roof over your head definitely reduces the amount of money or accessible income that you have to exercise. Right? Of course you say that. ‘Oh, there's Youtube, like, you can definitely follow youtubers. And like, Do Yoga or Pilates.’ But that's also another extra effort, right? As compared to if you have good facilities. You pay for a gym. I think that will also bar people from having, you know, a healthy, active lifestyle. And also. The fact that the paid disparity you know, if you have less pay, you definitely will not be able to afford the comfort of travelling. Staying near to the hospitals I mean, my hospital is quite near town, or most hospitals are quite near town. The rent in town area is- is sky high. I mean. I'm recently I was talking to a colleague right? She was saying that she, she currently pays like $600 for rent, and she's staying with someone else in the same room. So imagine that you're buying privacy. You want a single room. You stay on in your own room with no adjacent toilet is a thousand and two dollars, right? So I mean, and you're a NUS student and NUS student. We earn a bit more than you know. The people who come from other countries. And I think that that pay difference actually, definitely does, you know, bar people from having an active lifestyle. So when you generalise and say nurses. I think we also have to be aware of privileges. Yeah, we're definitely.. In terms of like financial, in terms of like family support, in terms of you know, location where we stay, right? I mean, personally, I- I picked a hospital where I live near. But we may not have that kind of options open to them. I do know of a colleague who spends an hour and 15 min traveling to the ward. I do not know how she does it. But the rent is cheaper, and therefore it seems like a better choice. So yeah. I think that's about it. I think privilege plays a very big part on, you know, being able to have a healthier lifestyle. Hmm.

**NJCL: Hm. Okay. [inaudible] Okay. I think.. I think that's all I have to ask.**

P4: Great.

**NJCL: This marks the end of the interview. Thank you for your time and participation**

P4: No problem. All the best for your.. Your you know. [NJCL: Participant laughs]

**NJCL: Thank you, let me stop the recording.**

-End of transcript-

# **Interview #5**

Participant and interview details
 Participant number: 5 (P5)
 Date of interview: 25 Sept 2023
 Interviewer Number: Navarro John Christopher Lambino (NJCL)
 Time IDI started: 1810
 Time IDI ended: 1850
 Observational notes: Participant seems extremely lethargic.

Section I: Interviewer’s summary
 The individual interview was conducted via Zoom. Upon entering, patient requested to continue the interview without turning on the video. Despite not seeing the patient, I could feel the patient was exhausted. It was later found out that she had a previous Zoom meeting that was supposed to end at 5pm but was prolonged until 6.10pm. This interview was suppose to be scheduled for 6pm, but she was only able to join at around 6.10pm. As such, participant’s responses were very brief. I was unfortunately unable to efficiently ask patient to further elaborate on their responses. Perhaps, the fact I had a previous interview at 3:30pm contributed to the fatigue I had when interviewing. Unable to see the participant, and the lack of energy of both interviewer and interviewee contributed to a rather lacklustre interview. As she was my first interviewee which did not truly elaborate on her answers, I was also stumped on how to get her to be more open. I will be consulting my thesis advisor on tips to manage this kind of participants. Additionally, she only has 2 years of working experience- the participant that has the least amount of experience so far. She also mentioned that she does not engage much in activities outside of work, citing fatigue and stress. This is in stark contrast with the other participants so far who have mentioned that they have goals, or activities outside of work. She also mentioned that nursing managers have minimal role to play in getting nurses to engage in healthier lifestyles. Outside of work, social media also plays a role in affecting her sleeping habits. Since I did Interview #4 just before this interview, the contrast between this 2 participants is emphasized. P4 was a lot more ‘driven’, who had activities outside, while P5 had a more idle lifestyle.

Section II: Transcript

- **NJCL: Okay. Alright, good evening, miss. Firstly, I would like to thank you for taking the time to speak with me today. My name is Christopher, and I'm an NUS Year 4 Nursing student. The aim of this interview is to get a better understanding on the perspective nurses have on self-managing their own health behaviours and how various factors may influence this. I want to find out what your views, opinions and experiences of this. I would really like to encourage you to speak what comes to your mind, there are no right or wrong answers. May I have your permission to continue video recording this session?**
- P5: Yes.
- **NJCL: Okay, then, let's start with some basic questions like, which ward are you working in right now?**
- P5: Currently, I'm working in the contact precaution ward. But I'll be going to Covid ward soon.
- **NJCL: Could you tell me about like the workflow, what kind of work you do in your current ward right now?**
- P5: Okay, currently I'm working in [Public Hospital Name]. So… It's the [Public Hospital Name]. [Location of Public Hospital]. So it's a bit different from [Public Hospital Name]. Because, if you uh… working in [Public Hospital Name], mostly you’re in the same ward, or you are deployed to other wards within the same level. But for [Public Hospital Name], it’s a slightly different case. Because depending on the manpower needs for the.. the wards. Let's say, if the COVID ward suddenly, there's a rise in cases, then there'll be more subdued over to help them. So my site currently is, I've been in the contact precaution ward, for about 2-3 months. And now I think COVID is on the rise now, so they are deploying me over to this COVID ward.
- **NJCL: Okay, let's see.. Then at your current ward now, how… How would you describe the workload?**
- P5: Oh, it depends. Because sometimes we have the patient profile mostly.. Is uh.. A1, A2 assisted. But mostly it’s state bounding. Some of them have to do tube feeding, then some chest tube, some uh.. Patients with [inaudible] for me, then the chemistry is different. And sometimes, if the patient is caregiver training, then the patient equity is considered higher. So, depending on the ward assignment that day. Yeah
- **NJCL: Okay, then.. Moving onto my next few series of questions… Regarding your lifestyle, as a nurse, how would you define a healthy lifestyle?**
- P5: Hm. Having a healthy lifestyle is having enough rest and having time to eat during shifts, and also just have a short break and… finishing work on time. [NJCL: Participant lightly laughs] Yeah. So, sometimes for us, we cannot find time to go for break to eat, or even go for short [inaudible] because we are overwhelmed with the nursing path, and then the… if it's like morning round, then we need to fill out with the morning changes. Like, let’s say if the team, they order some morning medicine, or some like, socks we need to call it in all those. So having healthy lifestyle to me, maybe, if having enough time to rest, eat and sufficient rest before the next shift.
- **NJCL: Then, specifically on your diet, how do you feel about your current dietary habits?**
- P5: Hmm… Current dietary habits depends on the shift that I'm working. So if morning shift, mostly me, I don't have time for breakfast, so I'm having lunch about 3, 4 PM. Then dinner will be delayed. And if afternoon shift, sometimes I have the time to go for break. Sometimes I don't. So I see, the dinner will be pushed later, to 9, 10 PM. And then for next shift right, normally I will eat some snacks, some bread, or some.. Instant noodles for night shift. Cause I get hungry at night. Then.. I wouldn’t say it’s very irregular, maybe on my off days it’s three meals a day. Other than that.. When I'm walking, it depends. Yeah.
- **NJCL: Hmm.. then, regarding, like your physical activity, physical activity-wise. How do you feel about your current lifestyle?**
- P5: Hmm. physical activity, normally, I only go out of the house for work. So.. In the ward, there's a lot of walking now, because in NCID, it's mostly two beds in a room. So there's a long pathway along the hallway. And then [inaudible]. Then, other than that.. I'm just resting at home. [NJCL: Participant lightly laughs] Yeah.
- **NJCL: Hmm.. Do you feel that there was a lifestyle change when you started working as a nurse?**
- P5: Hmm.. Lifestyle change. In terms of dietary or…? Daily exercise?
- **NJCL: Dietary, exercise.. Yeah, any of that.**
- P5: Lifestyle change definitely yes. In terms of sleeping habits. Yeah, I find myself sleeping better nowadays. Cause.. Hm, I’m working night shifts almost, almost every week. Then, need to transition back to the day shift, afternoon shift.. It takes time to adapt. And then the eating habits wise.. Uh. As mentioned earlier, it also depends on the shift that I’m working. Then for physical exercise.. Hmm. There's not much change as compared to before I started working as a nurse. Hm. Yeah.
- **NJCL: Okay.. Then, to summarise what you were saying earlier, what a healthy life is mainly about having enough time to rest. Then this also means like being.. Having time to actually go for your breaks and to eat during the.. during these shifts.**
- P5: Hmm. Yeah.
- **NJCL: Is there anything else?**
- P5: Hmm. Healthy lifestyle.. I think also about the workflow.. Like managing stress at work. Hmm.. Sometimes I feel very overwhelmed. Then, at the end of the shift, I just think back on what happened. And then, I go home and rest. And then the next day I come back to work again and have to stress again. [NJCL: Participant laughs]
- **NJCL: I see..**
- P5: Yeah, it's a repeating cycle. Yeah.
- **NJCL: Okay. Then, with reference to how you answered defined healthy lifestyle. How do you feel about maintaining such a lifestyle?**
- P5: Uhh… Maintaining such a lifestyle.. As in the current lifestyle that I’m having?
- **NJCL: Like what you said, uh.. Healthy lifestyle like, having enough rest.**
- P5: Hm.
- **NJCL: How you feel about like, actually getting enough rest for example?**
- P5: Hmm. On my off days, when I actually have enough rest, I feel very energised. And.. I just.. I want to stay at home. I don't want to go out of the house, because it's my off day and I just want to- to rest at home. Hm. Yeah.
- **NJCL: Hmm. Okay. then, moving onto my next set of questions.. What do you think are like the main facilitators, barriers, in actually maintaining a healthier lifestyle?**
- P5: Mmm. Facilitators. Hm… Maybe I'll talk about the barriers first.
- **NJCL: Can.**
- P5: Uh.. Variables maintaining a healthy lifestyle.. Hmm, working hours. Hmm. Stress levels.. And then, fatigue. Fatigue. Whether I have the energy to maintain this healthy lifestyle. Then facilitator.. Hmm. Depends on the work equity. Let's say if there are lesser patients that day, or if there are lesser admissions or transfers or discharged, then there'll be lesser things to do in addition to the nursing tasks, so.. I think that will help. To make me feel not so stressed. Not as tired, and then I will have time to go for my breaks and uh. Eat something, grab something from downstairs. Then that will help me. Uh. It will not have stress at work and I think this will help with.. Help have a healthy lifestyle. Yeah.
- **NJCL: Hm. Earlier you mentioned about stress levels. Elaborate. Like, these stress levels?**
- P5: Hmm.. Stress levels. I think, because uh, this is my second year of working, then.. Depending on the situation of the patient. If patients suddenly disagree, or if they uh.. Suddenly there’s a lot of uh.. IV medications ordered, or if there are some blood transfusions, then, I don’t like to think of that. Then, if other patients press a call bell, and if they want something, uh. I’m gonna need to prioritize that.. But then, if they cannot wait, then they want their.. Their medicine needs to be met at that point in time, I need to.. To uh. To quickly find.. Find a comfortable compromise with them, while I settle the patients who require the most attention. Then. I think that shift will be quite busy and quite stressful as well.. Yeah.
- **NJCL: Okay So I guess I’ll quickly summarize what you've been mentioning. When I asked you about the ‘What are your main facilitators or barriers in maintaining a healthier lifestyle’ for you, you mentioned that the barriers are working hours, stress levels and fatigue. Whether you have enough of the energy to actually maintain your lifestyle.**
- P5: Mhm.
- **NJCL: Then for facilitators, you mentioned that it depends on the workflow activity like the workload, whether it's very busy for you. So.. Like, if it's so busy to an extent that it can.. You don't even go and eat for your break.**
- P5: Yeah.
- **NJCL: Yeah. Is there anything else you'd like to add here?**
- P5: Uh.. No.
- **NJCL: Okay. Then, moving on to my next question, it's how does your personal lifestyle habits impact your work performance?**
- P5: Hmm.. Personal lifestyle habits.. I think similarly, having enough rest is very important. It allows me to think really at work, and also having to read up more on all patient.. Patient diagnosis that I'm not very sure of and like what I need to take note of. Then.. Sorry, can I get your question again? I think I’m.. Unsure. Yeah
- **NJCL: Sure, no problem. Uh.. How does your personal lifestyle habits impact your work performance?**
- P5: Hmm... Personal lifestyle habits.. Hm. Can give any examples?
- **NJCL: I.. One example.. Maybe.. Uh. I. I remember one of my participants, she mentioned that because she actually like engages in physical exercise, like going to the gym, she is more able to endure the long hours of shift work, something like that.**
- P5: Hmm. Oh, for me.. Hmm. I'll watch some dramas online. Then help me to destress, and just uh.. So it brings me away from work, and the stresses of the workplace. And then just.. Help me to destress. And then I can rest. And the next day I will go back to work again. Yeah.
- **NJCL: Then, moving along with this category, other than watching dramas online, do you have any other stress coping strategies?**
- P5: Coping strategies. Hm… Okay. The friends who are also in the nursing profession. Even though some of them are in different specialties. But I guess generally we do relate on a single level, because we also work shift works. And then uh.. Patient profile, even though it's different.But previously, because we all have clinical attachments before. So we kind of do understand what each other are going through, and then just ventilating our thoughts. Then can you feel.. Help release the stress of working. Talking to Seniors help as well. Seniors in the nursing ward.
- **NJCL: Okay, so you mentioned that talking to friends in the nursing profession, talking to seniors in also the nursing profession actually help with stress management.**
- P5: Mhm.
- **NJCL: How about talking with friends outside of nursing?**
- P5: Hmm. Talking with friends outside of working.. Perhaps more about.. Because.. Exactly. I'm.. Cause most of them are in uh.. Working in the corporate industries, so their working hours are actually different. And if I want to maybe meet up with them during working hours, it’s usually harder, cause they are working on weekends so much, then weekends have their day off. Then.. Uh.. The part where we can relate on is perhaps on.. com- communication, like communication with our seniors in the.. in the ward, like they can give advice on how I can.. If I'll communicate and manage stress. Yeah. Hm
- **NJCL: Okay. Alright! Then moving on to the second half of the series of questions. It's regarding the workplace in general. As a nurse, do you feel that the workplace influences lifestyle habits?**
- P5: Hm.. Workplace influence my lifestyle habits.. So far, hmm. I believe, yes, it does influence to a certain extent like, eating habits, keeping healthy based on the working shifts, and also work etiquette.
- **NJCL: I see. Then, how do you feel about the workplace culture among nurses in helping to facilitate or hinder the maintenance of healthy lifestyles?**
- P5: Workplace.. How does workplace help?
- **NJCL: Like the workplace culture nurses…**
- P5: Workplace culture? Hmm. Some of my nursing friends who ask me out to go gym sessions together. So I guess it helps me get more physical exercise outside our work. And yeah.
- **NJCL: How about.. Any barriers?**
- P5: Workplace culture?
- **NJCL: Yeah..**
- P5: Sometimes the workplace like to.. They organize physical activities or some activities to help destress ourselves, I mean.. Sometimes because of the shift hours, they say the event is held in the afternoon by walk-in night shifts that can attend, and then sometimes, if the. Uh. Have too many nurse with nothing to do in the afternoon, and then or.. If in the morning and I cannot finish, then I have to follow up after hand over, then I also cannot attend this kind of events that the workplace has. Uhm. Kind of awkward.
- **NJCL: I see.. Then you feel like the.. What do you think of the role of like nursing managers in helping us maintain their health?**
- P5: Mmm. Nursing managers..?
- **NJCL: Yeah, like brothers and sisters.**
- P5: I think they not so much of a rule that they do is more of, ask whether we have time or whether we can, ah.. can go. Have enough time to.. Whether we have time to eat or drink, or use the washroom. They can at least.. It’s more on us. But I guess in the assistant office they will put some snacks there, there, we can just help ourselves to have a quick bite there. Other than that. Hmm, not much.
- **NJCL: Okay. Let me just try summarizing what you shared so far..**
- P5: Mhm.
- **NJCL: You feel that the workplace actually influences your lifestyle habits cause it, it influences it to a certain extent. For example, your eating habits.**
- P5: Mhm.
- **NJCL: Is there anything else you mentioned in this part?**
- P5: Hmm. I think. No.
- **NJCL: Okay. Then, when I asked about the how the workplace culture among nurses helps facilitate or hinder maintenance of healthy lifestyles. Your first answer I'm guessing regarding regarding facilitators is your.. some of the missing colleagues actually invite you to go out for gym sessions. So that helps you to maintain your physical activity. And then for barriers, it's more of like.. It’s your shift hours a barrier, because even though they are like activities organized by the institution not able to attend it. Is that correct?**
- P5: Mhm.
- **NJCL: Is there anything else you wanna add at this point?**
- P5: Hm.. No.
- **NJCL: Okay, then my next question is, actually, you touch upon it with your answer previously. But how do you think your institution can help you to maintain a healthier lifestyle?**
- P5: Uh.. Well, sometimes during.. The most of the events they give, they do give out some vouchers. I remember, for this year's nursing day, they give out some tote bag containing uh. Mr. Bean healthier drink voucher for the [inaudible]. Then I think there's some skin care voucher as well. Then, uh.. Also Sketchers vouchers. So we can purchase shoes that they can wear a for work. Yeah.
- **NJCL: Does your workplace have any like, workplace health promotion programs?**
- P5: Uh.. Sometimes we do some promotion programs. Uh. I think it is a yearly event that they organize some.. inter professional game sessions. Then, what else.. Hmm. Health programs. That's all I can think of at the moment.
- **NJCL: No problem. Okay. Then.. Almost finishing. Outside of work, what do you think influences your lifestyle?**
- P5: Outside of work? Uhm. Family and.. Family and friends around me. Yeah, they influence my lifestyle the most. And sometimes, when I.. when I scroll through social media. you know. See some suggestions there.
- **NJCL: Can you elaborate more on this points, like how your family and friends influence, you, how does your the social media influence you?**
- P5: Hm.. Mostly they influence me.. Friends and family influence mostly on diet-wise. Cause sometimes, at home, when my mother.. When she cook, she tends to cook healthier options for me at least some vegetables, some green bean, and I'll just eat some fruits as well. Then for friends wise. When we go outside, we just eat whatever we want, based on our cravings. Then. Regarding social media wise.. I think it affect my sleeping habit. After scrolling through before sleeping. Yep.
- **NJCL: When you scroll through social media, are you.. Would you say your influence to maybe like go and exercise more frequently, that kind of thing?**
- P5: Hmm. Not really. Well, if sometimes.. We. Uh. Some friends and I.. we do have some hiking sessions, just around [inaudible]. [Inaudible]. And uh, yeah. The videos I mostly see on social media are food videos. So [NJCL: Participant laughs] So not really exercise related. Yeah.
- **NJCL: Ah then, since it's about food, does it influence your dietary habits?**
- P5: Not really. It's just I just see how they cook the food or how they prepare the food. And yeah, that’s about it. Hm.
- **NJCL: Then, based on your answers. Regarding dietary habits, would you say you're very like.. conscious of like, the nutritional value?**
- P5: Hmm. For me, not as much. As long as I'm hungry, then if.. Let's say if after shift, I'm hungry, and then I’ll just want to go somewhere near to eat. Then usually it's in the Novena area, so it’s just like, near like Kopitiam, or like, somewhere in the bus stop or just uhm. Somewhere nearby. Sometimes fast food as well. Yeah. Only- Sorry. Just add on, only at home it’s a.. more contrast, like course my mother cooks, for she will try to prepare like.. healthier food choices. Yeah.
- **NJCL: Okay. So for this question, just to summarise, the question outside of ;’what do you think influences your lifestyle’, you said that your family and friends actually.. mostly on diet wise, they will influence you to eat healthier, at.. At least because your, your mum actually cooks for you and your friends wise, they invite you out to eat. And then there's also scrolling through social media which affects your sleeping habits. Is there anything else you want to add in this?**
- P5: No, don’t have.
- **NJCL: Okay. Uh, just moving back to one of my previous questions. Can you elaborate how does.. How does your work affect eating habits?**
- P5: How does your work affect eating habits.. Hm. If.. Uh. If there's sufficient time to eat, then I'll go downstairs to purchase the food. But then, uh sometimes the distance from the ward to.. Because in NCID, there's food court downstairs, but they only open during the weekdays. So only if I didn't have food, then I need to go all the way from the ward in [Public Hospital name] to Kopitiam side, which takes about 15 minutes. So by the time I purchase the food and go back, I'm only left with 15, 20 minutes to eat. So it’s a rush. So sometimes I don't go downstairs to buy food. I just get a Milo in the pantry, and then I'll take some biscuits to eat. So yeah, the.. whether it's weekend, or weekday will also affect. Then, uh.. If I'm eating in the hospital, then it means it’s the healthier option.. Cause they only provide healthier option in the hospital. Then.. If after work then, I'll just eat around Novena, depending on food cravings. Yeah.
- **NJCL: Okay. Then, specifically on physical activity. Do you engage in any form of exercise outside of work? Maybe even inside**
- P5: Hm. Outside of work. It’s not as much because it depends on.. Mostly I exercise with.. With friends and It depends on when I'm free and when they're free. So it depends on our schedules that if we can match, and then. if the weather allows, then we can go outside for hiking or gyming sessions, but.. but not very often.
- **NJCL: Do you have an estimate of like, the frequency?**
- P5: Mmm. Maybe , 3, 4 times per month.
- **NJCL: Okay. I think.. That's all I want to ask for that. Do you have anything else that you would like to ask, speak about? That was mentioned in the interview. Any further comments you want to share?**
- P5: Uh. So far. Don’t have. [NJCL: Participant lightly laughs]
- **NJCL: Okay, then, this marks the end of the interview. Thank you so much for your time and participation.**
- P5: No worries, welcome. Thank you for the interview
- **NJCL: Thank you**
- P5: Thank you
- **NJCL: I’ll stop the recording.**
- -End of transcript-

#

# **Interview #6**

Participant and interview details
 Participant number: 7 (P6)
 Date of interview: 26 Sept 2023
 Interviewer Number: Navarro John Christopher Lambino (NJCL)
 Time IDI started: 0930
 Time IDI ended: 1020
 Observational notes: Participant looked alert.

Section I: Interviewer’s summary
 The individual interview was conducted via Zoom. Initially, interview was scheduled at 0900, but participant requested it to be pushed back to 0930. Participant understood the interview questions and answered to the point but required a bit of prompting to further elaborate. P6, similar to P3 also advocated a lot for mental wellness and staying positive as a definition of healthy lifestyle. Interesting points put up were about using her Christian beliefs to strengthen her mental fortitude. The bible as a tool. She also mentioned about taking care of her children and how that occupies most of her time. Kids are a priority. Social circle also plays a part in maintaining one’s lifestyle. Lastly, she mentioned having a habit ingrained in her and how it continues onto working life. Perhaps subsequent questions can be more on the impact of child rearing for nurses with children in self-regulating healthier lifestyles.

Section II: Transcript

- **NJCL: Alright. Firstly, good morning, miss. I would like to thank you for taking the time to speak with me today. My name is Chris, and I'm a NUS Year 4 Nursing student. The aim of this interview is to get a better understanding on the perspective nurses have on self managing their own health behaviours, and how various factors may influence this. I want to find out what are your views, opinions, and experiences on this. I would really like to encourage you to speak what comes to your mind. There are no right or wrong answers. May I have your permission to continue video recording the session?**
- P6: Yes.
- **NJCL: Okay, then, let's start with some basic questions. Which ward are you working in?**
- P6: Currently, I work in a specialty nursing department. Yeah. There is no particular ward, it is just a department.
- **NJCL: Could you tell me more about like, what kind of work you do at your ward?**
- P6: Okay. Currently, I'm a director nurse, so as a direct treatment, I see patient in the ward and also in the clinic. Yeah. So I do not particularly in.. in charge of one of the ward. So I just.. Whenever there's a patient that I would just go to see.
- **NJCL: Okay. Then how would you describe the work?**
- P6: Sorry. Describe the workload is it?
- **NJCL: Yeah. Yes**
- P6: Uh… Workload. So. currently, with me, I have another three of my colleagues. So we split the workload evenly. So whenever there's a referral usually, I'll go and see the patient first. [NJCL: Participant clears throat] If subsequently there's a need to there, I will ask for my colleagues to help me. Other than that. Most of time my other colleagues will be handling the clinic cases. Yeah.
- **NJCL: Okay, I see. Okay. Then, moving on to my next few series of questions, it will be regarding your.. Your uh.. Lifestyle in general. As a nurse, how would you define a healthy lifestyle?**
- P6: Hm. Uh… I think a healthy lifestyle is having to work.. Work life balance as well. So being able to have self care, to have time with the family, uh.. Able.. Having.. Like, having to eat, you know. Then, go to work, then uh.. And feel good. Yeah. [NJCL: Participant grits teeth]
- **NJCL: Uh.. Could you elaborate more on what you mean by ‘feel good’? Like having to work, to feel good?**
- P6: It’s having a good health, to have the strength, you know. To go and work.. To have the energy, to have the strength again to do what you like to do. Uh. Not feeling tired, not feeling sick. Yeah. Something like this. [NJCL: Participant nodding head slightly]
- **NJCL: You also mentioned self-care. Could you elaborate more on that?**
- P6: Uh. So self-care is like doing things that energise you like, for example, things I like.. I like to do.. Spending time with my family, having my own time, like going for massage. [NJCL: Participant smiles] Uh. Going for recreation. Yeah, exercises, that can energise oneself. Yep.
- **NJCL: Then, you also mentioned about having a good diet. Can you tell me more about that?**
- P6: Okay, so having good diet like. Like, after you take a good diet, you feel that all your body feels good. You would not feel that, “Ah It's too oily,” or it's too salty, that makes your body, uh. You know, uh.. Very somehow can makes you feel very overloaded. Yeah, your whole body. Yeah. So having good diet also makes you feel.. Feel happy, like, for example, if you something that I like to eat. Like, salad, vegetable, fruits.. So after eating diet like this, it makes you feel body feels like that your body is digesting well. Yeah.
- **NJCL: Then, lastly, you also mentioned the work life balance.**
- P6: Mhm.
- **NJCL: Could you expand more on that?**
- P6: [NJCL: Participant clears throat] So what life balance for me, I think. Uh. Not just focus everything on the work itself. Or bringing work home to do, but rather to have a balance between. Uh.. When you're at work, you fully concentrate, you fully, ensure your work is done before you go home, and when you go home, you fully... Your attention is fully with your family, with yourself, with the people that you love. Yeah. So rather than bringing work home. And you have your second half of your work at home. Yeah. So rather than that, I think it's important to have that work life balance. It’s also to maintain yourself. Sanity. Yeah. [NJCL: Participant smiles, laughing lightly]
- **NJCL: I see. I think you touch upon this.. This a bit, for my next question. Cause specifically on your diet, how- how do you feel about your current dietary habits?**
- P6: Uh. So far I've been. I will say that my dietary habit has been quite good. I usually choose less salt, less sugar, less oil, diet. Uh. More vegetable, more fruits. Yeah. These are the things I like to eat. Yeah.
- **NJCL: Then, uh moving on to your specifically on physical activity, how do you feel about the amount of exercise you get in?**
- P6: Actually, not much, because I have a kid. [NJCL: Participant smiles] So I feel that after work, coming back to look after my child can be quite physically tiring as well. So I don't really have time to really.. Like, set a time up for exercise, but if I do, uh, it is mainly to play with my kids or during weekends, go out and run around with them. Yeah. So that there is my exercise. [NJCL: Participant smiles and laughs]
- **NJCL: Do you feel like you want to change to have more exercise? For example.**
- P6: Sorry can you repeat?
- **NJCL: Uh, because earlier, you mentioned that your.. You're not getting as much physical activity because of taking care of your children. Do you feel that you want to change that to have more exercise?**
- P6: Uh.. I think it’s a setting, a routine.. Which sometimes can be.. I can be quite lazy. [NJCL: Participant laughs] I feel like after work, you.. The- the time that you have in between is quite short. Yeah. So after work is like 6, but can come home 6:30, for.. I mean, you have your dinner there. You don't really have time to actually go for exercise. Yeah. So after yeah, after dinner will be a bit of play time with your kids. Then, subsequently, by 8:30 , you have to, you know, shower your kids, and then bring him to- to.. To bed, yeah. So- so the time to really exercise, I think, is quite short. So I don't really have that uh.. Regular plan for, for my exercise. Yeah.
- **NJCL: Okay. Then, going back a bit. Uh, how did your lifestyle change after you started working as a nurse?**
- P6: How my lifestyle change? [NJCL: Participant furrows brows briefly] Cause all along I’ve been a nurse, so nothing much change. [NJCL: Participant laughs, scratching ear]
- **NJCL: As in, before you started your work as a nurse. Like when you were, maybe a student. And then you became a nurse afterwards.**
- P6: Uh.. I think pretty much similar. Not not much of change. In terms of exercises, you mean is it?
- **NJCL: Uh, in terms of exercise, diet..**
- P6: Oh, okay, so so, yeah, yeah, of course, I mean, after being a nurse, I think my diet also did make a bit of changes. I.. Uh.. Been more conscious of having to eat those oily food. Maybe last time, I used to like to eat fried chicken, [NJCL: Participant smiles and laughs] uh, the skin, you know. Yeah. So after being a nurse, I think, be more cautious about it, because having to see a lot of patients, you know, falling sick, so.. I do not want to be like them. Yeah. So, so more health conscious in that way. Yeah.
- **NJCL: Okay, then, summarizing what you've been sharing so far. When I ask about how you would define a healthy lifestyle. For you personally, it's having work life balance, self care, time for your family, having diet, having.. Uh, feeling good when you’re going to work. Uh, is that correct?**
- P6: Yes.
- **NJCL: Would you like to add anything else?**
- P6: Uh.. I think also, plus the mental health. Yeah, having a good mental health, have a positive mindset as well. Yeah.
- **NJCL: Can. Then. moving on to my next question, with reference to your answers on ‘how to find a healthy lifestyle’, as a nurse, how do you feel about maintaining such a lifestyle?**
- P6: Maintaining.. I think. Have to be very uh.. You need to be very uh.. You need a plan your time. And be very uh.. What is that word? To.. Uh? Stick to it? Yeah. So I think sometimes the motivation is not really there. So how do I maintain.. [NJCL: Participant turns head, looking around] Uh.. Maybe having someone to.. To.. To.. To buddy with you, having someone to uh. To work out with you, to give me fuel. Yeah. Maybe that will bring more motivation for you to do more exercise. [NJCL: Participant nods and smiles] Yeah.
- **NJCL: Okay. My next question, I think you touched upon it. Cause I wanted to ask, like, what are your main facilitators or barriers in maintaining a healthier lifestyle? So I think you mentioned like, buddying up like, having someone to, to work out with you. I'm guessing that will be like a facilitator. Can you think of any other facilitator or barriers?**
- P6: Uh.. Yeah, I think, for facilitator will be mainly my husband. I won’t- I don’t really like those instructors, you know, to buddy with you. Because you have to pay money. [NJCL: Participant laughs] So, try to save a bit. Uh, so.. Mainly to, you know, buddy with your- I mean with my husband. So, sometimes, we will go cycling. Sometimes, if we can find time off, we do go out for some exercise as well. Hmm. Like, to find a common- a common time. Yeah.
- **NJCL: Uh, are there any more facilitators that you feel?**
- P6: Maybe with friends like, sometimes I do, uh.. Sometimes our hospitals do have some free program, like Zumba, that kind of exercises, but also have to depend on the timing of the exercise. Yeah, either the timing, the people that go with you. And whether.. Yeah, how, How is your day that day. Yeah. So if your day is bad, unlikely you.. You want to go for exercise. You may want to, you know. Settle your things at home, or set up things first before you go. Yeah.
- **NJCL: Uh, can you define what you mean by the day- the day was bad?**
- P6: Like, maybe you have a lot of work to do, so you can't finish the work. Then you have to stay back to finish your work. Yeah. So in that sense, you can't make time for exercise. Yeah.
- **NJCL: Hmm. So is it right for me to say barriers on maintaining a healthier lifestyle is mostly the- the timing which you mentioned earlier, and the.. depending on how the day went. Very busy..**
- P6: Yeah. Also the people that go with you, whether are they the motivating type, whether you know.. You, you the.. The partner that goes with you, whether uh.. Yeah. Sometimes you feel awkward if you don't really know the person. Yeah. So must be someone who you are comfortable with. Yeah.
- **NJCL: Okay. Would you like to add any more, maybe facilitators or barriers maintaining a healthier lifestyle. Or is that all?**
- P6: Barriers also can be depends on the weather. If the weather is raining, then a bit difficult. Although you can.. Yeah, play like indoors. Barriers can be… Yeah, like, if you are sick, of course you can’t go for exercise. Uh… What else.. There is like, maybe the.. Maybe.. Yeah, the time I told you already. Yeah, basically like that. [NJCL: Participant nods] Yeah.
- **NJCL: Uh. I think. I think because you also mentioned about having a mental.. Being mentally healthy. So in terms of like, the mental aspect, do you feel like there's any facilitators or barriers in maintaining a healthier, like, mindset?**
- P6: Yeah. So I believe in, uh.. That having a mental wellness, is about wellness is about, having a positive mindset. So being with people who are positive in their thoughts, in their talk, in the way they talk, I think will motivate me and keep me uh.. I think mentally well as well. If you're always with someone who is very negative, who always like talk back on someone else, then I think somehow you will have the not so.. Uh. Good vibe, yeah. So- So it's always good to choose the, the right person to talk with. I think it, it helps with your mental health. Yeah.
- **NJCL: Okay, then, for my next question. How does personal lifestyle habits impact your work performance?**
- P6: Personal life habits.. Oh, okay. Uh.. I think if you’re healthy, you have the positive mindset, it will somehow infect your work life because you will be more positive when you face challenges in your work, then. When your friends are, or your colleagues are negative about certain project, like changes they can’t seem to see a breakthrough. Then, at least before positive mindset, you.. somehow able to push the boundaries. Yeah. So I guess. It's important to have a good mental health as well. Yeah. And not keep thinking about the negative part about your work. Yeah.
- **NJCL: You mentioned something about pushing past the boundaries. Maybe elaborate more that?**
- P6: Uh.. Like. You won't give up easily. So having to push the boundary is.. First, the positive mindset, you want to dare to try new things. And even if feels bad, you know that. Yeah, that is part of the challenge that you need face. And you will work out something else. Yeah.
- **NJCL: Hmm. Yeah. Do you use any kind of like, maybe tools or apps that help you to maintain your lifestyle? Like, for example, maintain a more positive mindset. Do you use anything to maintain that?**
- P6: Hmm. Okay. Because I'm a Christian, so I will have a Bible app, that reminds me about a verse everyday. So it does somehow. Uh. Keep me in line with, with, with the positive thoughts, yeah, when I do my work, when my, when I do my.. The- the day that is ahead, I know, that is maybe going to be challenging. So with my faith in line. And I'll be likely able to go through it. Yeah.
- **NJCL: Then, how about for, specifically, for maybe diet or physical activity, anything to maintain level of diet, or physical activity?**
- P6: Diet, I think it's already ingrained in me so doesn't really need an app to follow through. Uh.. I think there is this HPB app as well to count the number of steps you have, so that one I do have it in my apps. So it's how it help me to.. Keep myself aware that how many steps I have in per day. Yeah, something like that.
- **NJCL: Uh, so you mentioned that your diet is already ingrained in you, can you elaborate on that?**
- P6: So after like earlier, I mentioned earlier, after I become nurse, I'm more aware about diet, then uh.. And all along I have been eating less salt, you know, less salt, less sugar less oil, so, in every where I go I will always ask to- I will always order less to the whatever food I order, then. Ask ‘can you put that less oil or less salt?’ So, if.. If possible. Yeah. So I always have a habit to do that. Yeah.
- **NJCL: So, you already formed a habit beforehand..**
- P6: Yeah.
- **NJCL: Okay.**
- P6: Don't really need an app to remind me. [NJCL: Participant laughs] Yeah.
- **NJCL: Okay, then, for my next series of questions, it will be regarding the workplace. So as a nurse, do you feel that the workplace influences your own lifestyle habits?**
- P6: Yes, yes, I think it plays a part as well, because, most of your days you spend at work so the food that they have in the canteen, the- the people that you eat with. I think all makes a difference, like if your friends always like to eat fast food. Then, of course, I think your diet won't be.. You won't have a good, healthy lifestyle, I guess. if your workplace don't provide choices of uh, you know, good food. They, of course, or healthy food rather, then most of time, you choose unhealthy food. Yeah.
- **NJCL: So for your answers for this question, it's mostly the physical environment. Then my next question will be more on the, I- I guess social aspect? So like, how do you feel about the workplace culture among nurses in helping to facilitate or hinder your maintenance of your lifestyle- of healthy lifestyles.**
- P6: Uh.. I think the colleagues themselves, they play a part. Like I do have some colleague, who only eat vegetable during their lunch [NJCL: Participant laughs] or salad during their lunch. yeah. So it does.. more on. It does help to encourage you to be aware of .. about the food that you eat, and.. and of course the.. the choices that you made. What- What kind of lunch you have in our workplace. Yeah.
- **NJCL: Uh, then. Do you think any anything in the workplace culture among nurses will actually like, hinder your maintenance or healthy lifestyle?**
- P6: Hinders..
- **NJCL: Yeah, like barriers.**
- P6: I think not really, because. Like, I don't really like to eat things that is too unhealthy, like those oily stuff. But sometimes I do eat. If I have colleague who always or every day eat, right, I would want to follow. Yeah, lifestyle. Yeah. Cause I still prefer food, that is, healthy and eat it. It- It helps me feel better, like after eating a good healthy lunch. Yeah.
- **NJCL: Okay, then, just to quickly summarise what you've been sharing. For how do you feel about workplace culture among nurses, in helping facilitate or hinder the maintenance of a healthy lifestyle, for you, you say that how it facilitates is mostly through your colleagues encourages you to eat healthy cause, you see them eat healthy, then feel encouraged to follow them as well, but at the same time.. So. You feel like your colleagues, those who eat less healthily they will. They might also like, sort of influence you, am I right to say that?**
- P6: Yeah. So there are times that they will call like, bring- ask you out for lunch, and they choose the not so healthy diet, and in order to, you know, sometimes to linger with them, you will also join them . Yeah.
- **NJCL: So is it right for me to say, like colleagues can both facilitate and hinder a healthier lifestyle?**
- P6: Yes, yeah, definitely. Yeah.
- **NJCL: Then, other than colleagues, have any other thing that you think. facilitates or hinders the maintenance of healthy lifestyle?**
- P6: Uh. I think the distance or the.. The.. The physical environment, whether they have those food that is healthy. Or.. If the healthy.. For example, a healthy food is very far away, then you don't have time to travel so far, then, definitely you won't choose that. But you choose somewhere nearer to, to your workplace. Yeah.
- **NJCL: Okay. Then, moving away from diet and more of the mental aspect. Right, you said healthy lifestyle includes mental wellness. You think, uh, the place culture impacts this mental wellness. The- the workplace culture among nurses impacts this mental wellness.**
- P6: Mental wellness.. Yeah, I- I think so. Cause if everyone is positive in, yeah, my side, I guess the working environment would be a better place, but if oh.. If you are in the place where is, uh, you know, toxic like, for example, say bad things about each other or having to.. Toxic, I guess it will somehow affect your mental health. Health wellness as well. Yeah.
- **NJCL: Then, what do you think about the rule of nurse managers in helping nurses maintain their own health?**
- P6:: What is the role of nurse manager, Is it?
- **NJCL: Yup. In helping nurses maintain their health.**
- P6: Oh, okay, uh.. So they can be the ambassador as well to advocate good diet. Regular exercise, having a positive mindset, I think all this the higher management, or rather the nurse manager, have a big role. Yeah, cause I think that if you don't advocate, then people will not want to follow or, have nothing to follow. So at least. When you advocate for it, people who are more health conscious, they will feels that ‘Oh actually given a way to maintain a healthy lifestyle.’ Yeah.
- **NJCL: Okay. Alright. Then, I think you mentioned a bit earlier. But how.. How do you think your institution can help you to maintain a healthier lifestyle?**
- P6: Like, the physical environment. The cost of the food as well. I think it's important. If it's too expensive, then unlikely, you will want to buy. And then uh.. yeah, and the people around you, yeah, and whether the manager or the higher management, they advocate for- for us as well. Yeah.
- **NJCL: Then, specifically. Ah, sorry. Could you maybe elaborate how like, the higher management can advocate for you?**
- P6: Okay, so our.. Currently our hospital, they do have this weekly kind of program. For like exercises, like earlier, I mentioned about having the zumba exercise, then they, I think every Monday they do have the fruits. They as well. I mean having someone to sell, you know, fruits to the public and also to our own colleagues. So I think all this, it makes a difference. Yeah, for you to.. You want to buy an egg, you know, to go and share your family. So all this is I- I see them see as how they facilitate for us, and advocate for- for healthier lifestyle. So you also see how higher manager also participate in those activity or exercises. Yeah.
- **NJCL: Okay. Then, for my final question. Outside of work, what do you think influences your lifestyle?**
- P6: Outside your work would be your family, your friends outside your word, they’re who can influence you.Yeah. So whether or not they like, have a healthy habit, whether they are the one who, well, ask you out for exercise, or for example go out to eat healthier food, all this, I think, plays a role. Yeah.
- **NJCL: Can you elaborate more on how you believe they actually like, influence you?**
- P6: Yeah. So if like, for example, my. Uh. Maybe my husband likes to eat food that is unhealthy. Usually they.. He will also buy food back for us to eat, for example. So if.. You, you cannot choose whether you eat, you know. Because it's already, you know, it's already buy for you. So I guess, uh. Yeah. It somehow influence what you eat on that day. Yeah
- **NJCL: Okay, then let me just quickly summarize what you've been sharing with me. As a nurse, ‘how you feel that the workplace influences your own lifestyle habits’ on some of this is, yes, once you said that, like the physical environment, the kind of colleagues you work with, which can also act as both a facilitator and hindrance to maintaining a healthier lifestyle. Then, for ‘how can the institution help to maintain a healthier lifestyle?’ You mentioned the physical environment, the cost of the food and the people around you. And like how the higher management can advocate for you. Is this all right so far?**
- P6: Yeah, it's all right.
- **NJCL: Okay. Yup, then is there anything you wanna add at this point?**
- P6: So far. No.
- **NJCL: Okay. Then, finally, when I ask you about outside of what you mentioned, the family and friends can actually influence you like, if they eat healthier or eat healthly as well. Maybe if your husband buys food outside, and it's a bit unhealthy. You can't really do much cause it's really bought for you.**
- P6: Yeah
- **NJCL: Yeah, then, is there anything else you want to add? Like outside of influences.**
- P6: Uh. No.
- **NJCL: Okay. Can. Okay. Then, do you have anything else that you would like to ask, or speak about that was not mentioned in the interview? Any further comments you want to share?**
- P6: Uh… Healthier.. Maybe talking about like, for example, also, smoking, I think also not some healthy habit. So I wouldn't really mingle with friends who smoke. Yeah. So yeah, aside from other diet and mental health. Yeah. Those bad habits, I guess. Yeah.
- **NJCL: Can you maybe elaborate on what you mean by not mingle with this kind of people, like smokers?**
- P6: Yeah, because I know that third parties also does affect your health. So I will try to avoid them. And yeah, not.. not mingle with them around. Or if there is someone who smoke around that area, then I won't purposely work towards that area, I will try to edit my route. Yeah. [NJCL: Participant smiles] Just to avoid the- the smoking part. Yeah.
- **NJCL: I see. Then, just to ask, is there a lot of like colleagues that actually smoke?**
- P6: Ah, I don’t know. [NJCL: Participant laughs]
- **NJCL: Okay, okay. Yeah. Cause I'm thinking, like, if- if your colleagues like to smoke, then it's a bit hard to always having to avoid them.**
- P6: Yeah, true. But thankfully, my colleagues know. They.. They. They don't. They don't smoke.
- **NJCL: Oh okay, okay. Do you feel like maybe.. Okay, sorry. Nevermind. Yep. Okay. Then, other than smokers. Is there any further comments you want to share?**
- P6: Oh, that's all.
- **NJCL: Okay. Then, this marks the end of the interview. Thank you for your time and participation.**
- P6: Okay, thank you very much.
- **NJCL: Let me stop the recording..**
- -End of transcript-

#

# **Interview #7**

Participant and interview details
 Participant number: 7 (P7)
 Date of interview: 26 Sept 2023
 Interviewer Number: Navarro John Christopher Lambino (NJCL)
 Time IDI started: 1200
 Time IDI ended: 1300
 Observational notes: Participant looked slightly tired.

Section I: Interviewer’s summary
 The individual interview was conducted via Zoom. Participant mentioned that she has just woken up. Participant required a bit of prompting, but as she warmed up to the interview, became more forthcoming with her responses. Also perhaps due to her without an audio device and solely using her phone, she was having trouble hearing the questions, and I would have to repeat and rephrase multiple times for her to understand. Participant is a relatively new nurse working in a geriatric ward. Again, mention of shift work, manpower issues was made apparent. She was also another participant who expressed her lack of a healthy lifestyle and how her current cycle is ‘go to work, sleep, then go to work again’. There was a huge emphasis on having the ‘determination’ to maintaining a healthier lifestyle. She also expressed that nursing managers play a minimal role in helping nurses maintain their health as she believes one’s health is one’s responsibility. Interesting point put up was about family being a barrier for maintaining one’s healthy lifestyle. For her, she had to take care of her grandmother, and perhaps her fear of what could happen to her grandmother is exacerbated by the fact she is working in geriatric ward. She had other people to take care of outside of her work, other than her patients. Workplace culture is also mostly a hindrance for her, despite it being positive workplace (Everyone caring for each other). Peer pressure is cited as a reason. Subsequent interviews should ask about impact of having to take care of others outside of work.

Section II: Transcript

**NJCL: Okay. Making sure everything's working.. Okay, good afternoon, miss. Firstly, I would like to thank you for taking the time to speak with me today. My name is Chris, and I am a NUS Year 4 Nursing student. The aim of this interview is to get a better understanding of the perspective nurses have on self managing their health behaviors, and how various factors may influence this. I want to find out what are your views, opinions and experiences of this. I would really like to encourage you to speak what comes to your mind. There are no right or wrong answers. May I have your permission to continue video recording this session?**

- P7: Yeah.
- **NJCL: Okay, then, let's start with some basic questions like.. Which ward are you working in right now?**
- P7: Currently, I'm working in [Public Hospital Name]. So I'm working in geriatric specialist ward. So usually most of the patients are 75 years old and above. But there is also overfill from other specialty as well, because technically, the hospital is overcrowded in that sense. Yeah.
- **NJCL: Hmm. Then, in terms of your workload, how- Can you describe to me how much you work these days?**
- P7: Oh, sorry. Could you repeat again?
- **NJCL: Oh, sorry. Describe to me like, workload?**
- P7: Oh, workload. Usually for staff, nurses wise, we would take up to 14 patients, cases for students and spikes nowadays, because there is a lack of manpower. For us wise, we how do I say them. [NJCL: Participant looking around] In the ward, there is 41 patients- patients, but the nurses wise.. On a bad day, there's only 5 nurses. So there is 3 teams. There is team 1, team 2, and team 3. So each team, there is one nurse, which are registered nurse, giving medicines and all. Then our- So [inaudible] specific cases, then for our junior wise, they will do the diaper changing, and so on. But as I'm speaking, those juniors will be taking 20 to 21 cases. So one person- Technically one person will be changing diapers. Yeah, so. But on a good day, there will be 6 nurses. So, maybe one nurse can take 7 cases. But you have to do everything by yourself. Yeah. So I would say that once I started working, the workload has been quite insane now, yeah. [NJCL: Participant smiles and nods]
- **NJCL: I see. Okay. Then, moving onto my next set of questions, it will be regarding your- your lifestyle.**
- P7: Mhm.
- **NJCL: So as a nurse, how do you define a healthy lifestyle?**
- P7: Uhm. honestly, I don't think I have any, because we don't really take breaks. And then we also don't.. I mean, once we end shift, we just go home and sleep. Then the next day, work shift again. And because now our hospital, they changed the time system. [NJCL: Participant gesturing] So basically, now our next shift, we work 12 hours. And then for our day shift wise, we don't.. I mean, we work from 12 to 9 for PM shift, then 9 to 7 or 3 for AM shift. [NJCL: Participant gesturing] So if let's say we want to have a healthy lifestyle, we can only do it after morning shift. So.. But after morning shift, we’re all very tired. So technically, I don't really have. Yeah, I'm so sorry. [NJCL: Participant smiling, laughing]
- **NJCL: Then, in.. Then, for you, what is an ideal, healthy lifestyle?**
- P7: Hm. For health wise.. I guess if I have the free time and energy, I will do some.. I would like to do some light jogging. Which is what I do in the past. But currently, for now. The work is from gym [NJCL: Participant laughs]
- **NJCL: Okay. You mentioned you jogged in the past, so, how- How did your lifestyle change after you started work as a nurse?**
- P7: So previously, when I was still studying. We had a more within lifestyle. So basically, you know, study, then after that, evening.. [NJCL: Participant gesturing] You know, it’s a set timing. Then maybe after that, maybe after dinner, I'll go for some, like jobs. After that I'll just come home, do my work and sleep. So the day just repeats like that, but currently for now, because there is rotational shift. So for us to really go and plan our time to have a- to exercise, it's quite difficult, because sometimes you work PM shift, then the next day you will AM shift. So by the time I reach home, is around 10 plus for PM shift. Then I cannot really go and jog, because the next day, I have to wake up around 5 to go and to start my day to go in work. So then, after that, when I come home I'll be quite tired, because the previous day I don't have enough sleep. Then when I sleep, the next day, when I wake up I need to work night shift. So I have to continue sleeping. So that I have energy for next shift. So, uh. I'll say the.. The shift timing really.. Yeah, it's not really good for you to have a routine and set lifestyle. Yeah.
- **NJCL: Okay. Hmm. So, talking more about like, physical activity wise, how- How do you feel about your lifestyle like, feel like changing it?**
- P7: Changing my lifestyle?
- **NJCL: Yeah, changing-**
- P7: Yeah, yeah, of course. I will feel like, I would like to change my lifestyle to a healthier one. Yeah.
- **NJCL: Okay, okay.**
- P7: Yeah
- **NJCL: Hmm. Then I think, diet wise-**
- P7: Hm!
- **NJCL: How do you feel about your current dietary habits?**
- P7: For now.. Hmm. It's not as good as last time, because last time, I- I will make more wise choices, because I have the time to think, but currently, for now, I don't have breakfast in the morning because I need to go to work then.. For lunchtime, usually.. Because we don't have a break, my lunch is around 3 plus. And then my dinner wise, it's around before I sleep, so it's not very.. Good in that sense, the timing wise, and then the food choices wise, I just eat whatever there is. So.. I don't really think because when you're hungry you don't really think. [NJCL: Participant laughs] Just want to eat. I think the work really affects the- yeah, the lifestyle and the food choices.
- **NJCL: Hmm. Do you feel like improving your dietary habits?**
- P7: Sorry? [NJCL: Participant gets closer to screen]
- **NJCL: Do you feel like you want to improve your dietary habits?**
- P7: Dietary habits?
- **NJCL: Yeah. Want to improve it?**
- P7: [NJCL: Participant gets closer to screen] So sorry..
- **NJCL: No problem. Okay, let me rephrase. Do you feel like you would- How- how do you feel about uh, improving your diet?**
- P7: Oh, I would like to improve my diet very much. [NJCL: Participant smiles] You're asking me about the steps, is it?
- **NJCL: Yeah, like, in your ideal situation, how do you think you would like to improve your diet?**
- P7: In an ideal situation, I think I would like to start meal prepping, so I wouldn't just eat nonsense and eat whenever I'm hungry. Yeah.
- **NJCL: Hmm.. Okay, then, okay, my, my next question, you sort of touch- sort of like touch upon what you have been mentioning. So as a nurse, how do you feel about maintaining or changing your lifestyle?**
- P7: I think. Like what I said, it's quite difficult because of the rotational shift. And if let's say I will want- If let's say I'm determined to change my lifestyle. I think planning ahead would be very important. Yeah. So.. If the so called manpower is getting better, I think I will have more time and more mental space to really plan for my uh.. Plan for my lifestyle changes in a sense, [NJCL: Participant smiles and gestures] you know, and I guess, for now, if let's say you want to tell me to change my lifestyle, I think the lifestyle change will be a very short term instead of a very long term lifestyle change. Yeah. And if I were to really.. If I really want to change for the long term. I think planning ahead is very important for nurses wise. Is that a good answer?
- **NJCL: Yes, yes.**
- P7: [NJCL: Participant smiles]
- **NJCL: You mentioned something about like, the- It will be a short term kind of behavioral change. Can- can you elaborate more on why you feel that it will be short term?**
- P7: Hm! Because I guess a lot of these comes back to the shift hours, and the rotational shift. Depends on the day. If let's say it's a good day like, Sunday or Saturday, usually Sunday, Sunday or Saturday for the ward wise. There's only one consultation, one round consultation. So basically, the medical changes. There's not a lot of medical changes. So, on the weekdays, usually I have more time for myself. But on the- Sorry- On the weekends.. But on the weekdays.. Because there's a lot of changes to do, so sometimes I have to stay quite late to finish up my changes before I can end shift. So by the time I want to go home, I think I'm quite tired already to even, you know, want to go and exercise. Yeah. So even if I were to really go and exercise, I think I will check the ward list. Yeah. So I think it will be a short term.
- **NJCL: Then, what do you think will allow you to have a long term behavioural lifestyle change?**
- P7: I think.. The.. First of all, I don't think the shift timing will change in the long run, so I think the changes will come… I have to change myself, so I guess planning ahead is very important for me for the long run. And secondly, I guess determination for me. And why do I want to have a healthy, healthy lifestyle. I think the purpose is also quite important.
- **NJCL: Yeah, just to summarise what you've been saying. When I asked about maintaining, changing your lifestyle. You feel that it will mostly become just short term change. Because there you feel that the shift hours, or like the rotational shifts, is very… It's a very big barrier changing your lifestyle. If you want a more long term change, you need to be able to change yourself first, plan ahead and have like, determination. These changes, this.. Is this correct?**
- P7: Mhm
- **NJCL: Okay, then, for my next question, it's regarding the facilitators and barriers in actually maintaining or changing your lifestyle to a healthier one. So I think you highlighted a few barriers already in terms of like, the shift hours uh, and like.. And like fatigue as well. So these are like barriers. Do you have any barriers maintaining a healthier lifestyle?**
- P7: Other barriers, is it? [NJCL: Participant pulls away from the screen, looking upwards] I think.. Uh.. [inaudible] Currently for my situation wise, because I.. Uh. One of my parents has passed on. So I have a elderly grandma, and my mom is still.. Not working so in that sense, I'm still- I'm the breadwinner of the family in that sense. So a lot of times I need to go home and take care of my grandma. So that gives me very little time to really have, like you- Have spend time for myself. Yeah, because if I were to be.. When I end, I have to go home. Then, after, my grandma wants this. And you know, I have to take care of her, for daily living since young. Yeah, so I think family is another barrier for my case, yeah. [NJCL: Participant gets closer to screen] Hm.
- **NJCL: Hmm, then for facilitators like, what do you think can help maintain your current lifestyle?**
- P7: Hm.. Facilitators.. Hm. I think having a social circle, friends that are a mix.. Good lifestyle choices. Then I think I can make friends with them, and then go on.. And change mine as well... So sometimes. My.. What.. Colleagues they would want to go hiking, [inaudible]. So, they will ask me to join. So from there, you know, you can make sense as well as do some exercises in your lifestyle. And also sometimes, when they usually.. Someone will help us buy food, so from there I can also ask them “Hey can help me buy some healthy food?”. Yeah. So I think, the ward wise, my social circle.. I think those are my facilitators for lifestyle changes. Hm.
- **NJCL: Other than having a social circle…**
- P7: Hm! [NJCL: Participant is close to screen, nodding]
- **NJCL: Can you think of any other facilitators?**
- P7: Hmm.. [NJCL: Participant is silent, staring outside the screen] I think.. Applications? Mobile app. Let's say, you know, the ‘My fitness counter’. [NJCL: Participant smiles] Yeah. I mean, I used to use that when I started working. Yeah, but really not good. I can track my.. Diet. But currently, for now, not much, not much. But I guess if I were to be more determined, then, I think I'll start using that. One of the facilitators.. So, technology I think can help. Hm.
- **NJCL: Other than the ‘my fitness app’, do you use any other application or tools to help maintain or change your lifestyle?**
- P7: The.. Apple Tracker
- **NJCL: Apple Tracker.**
- P7: When we go [inaudible]. Yeah.
- **NJCL: I'm sorry. Could you elaborate more on the Apple Tracker?**
- P7: Uh.. you know, when we go for jogging. [NJCL: Participant gesturing] It will track the distance and all. Yeah.
- **NJCL: Okay, okay.**
- P7: Yeah, I don't know what's the app name. [NJCL: Participant smiles]
- **NJCL: I see. Oh, I also not so sure. But I- I understand what you're saying. I- I- I see it before. Okay, then I'll just quickly summarise what you've been sharing. So for you, in terms of like, the like, the facilitators and barriers in maintaining or changing your lifestyle, you feel like, the barriers are other than fatigue, there's also personal barriers, such as your own unique family situation.**
- P7: Mhm. [NJCL: Participant is close to the screen]
- **NJCL: And then for.. Yeah, family is actually a barrier. Is- Is that right for me to say?**
- P7: Yeah, can say that
- **NJCL: Okay, then for your facilitators, it's having a social circle and utilising technology, like ‘my fitness’ app, or the Apple Tracker, that you mentioned**
- P7: Mhm.
- **NJCL: Okay, then, are there anything else you want to add in terms of facilitators or barriers?**
- P7: I… Don’t think so, no. Thank you.
- **NJCL: Okay. Earlier you mentioned something like, if you were more determined- If you were more determined, maybe you would change your lifestyle more. Could you elaborate on what you mean by that? On ‘determine’.**
- P7: I guess, to have for purpose, to why I want to change my lifestyle to have a healthier one. Cause currently, I had none. [NJCL: Participant laughs] Cause previously, I had some Yeah, so I guess, uh.. My grandma will be a very big factor. So if I want to- The purpose of you- So if I want to be healthier, maybe I can help her out in more things. And then, you know, she don't have to worry about ‘what if something were to happen to me’. Yeah. So, yeah, I think if it was for my grandma. Yeah. it's important. Other than that, also, in the future I may want a family. And then.. The lifestyle changes that I- The lifestyle that I have now, you know, may not be good for my health in the future. I guess. If I want a family, I should change for the better. Yeah, the.. The dietary habit as well as my exercise. Hmm.
- **NJCL: Okay, then. Just to clarify, you becoming more determined, is about having, like personal goals…**
- P7: Mhm! [NJCL: Participant is close to the screen, nodding]
- **NJCL: In- In at least for your case in terms of like, personal goals, is like your grandma, or having a family in the future.**
- P7: Mhm. I think another one is also [NJCL: Participant adjusts hair] being scared, because I mean, we see, I mean, I'm taking care of elderly people, but sometimes there's overflow of patients, some patients as young as 27 years old, or even my age, I’m 25 this year. They come in for illness, that there are only- For only- You only see for elderly people. So when you see those kind of pieces, you'll get scared for yourself like ‘oh, what if I become like this?’. So I think being scared is also another factor. Yeah.
- **NJCL: Oh, I see. Like, patients’ conditions actually.. Okay.**
- P7: It’s sometimes scary. Cause.. It's not.. I've seen quite a lot before, since I start working. I've seen quite a lot of people, young people, 20+ years old, come in for diagnosis issues. So these kind of things are only for elderly people. You mostly, you see, for elderly people. But more and more young people are getting this. You know, so, and the should not live through all this, which is quite sad. And you also don’t want it to happen to you. Yeah.
- **NJCL: Okay, moving back a bit.. You also said something like, previously you had some determination.**
- P7: Hmm!
- **NJCL: Can you elaborate on that?**
- P7: Uh. I think, because previously there was- When I was studying, it was during the COVID period. So there's a lot.. [NJCL: Participant gesturing] I have a lot- Quite more friends like that since. So we would be like, ‘Oh. Let's zoom work out today!’. And all. So from there we, you know, we get to have groups and then have more determination because there's more social. You know, your friends are there to support you. Yeah.
- **NJCL: Is- Is it right for me to say this is like, more social support for you too?**
- P7: Hm! Can say that.
- **NJCL: Okay. Alright, can. Then for my next question, how do you think your personal lifestyle habits impact work performance?**
- P7: I think.. [NJCL: Participant murmurs] I think for me, made me more lethargic first. Previously, when I could get out and exercise, I'm more energetic. But for now, you- If I end, I just want to sleep. I'm just hungry. So once I reach home, I just sleep. The next day, I just work, so in a sense that it makes me more lethargic. But also mentally. It makes me dread working because I just sleep, eat, work. Sleep, eat, work, you know what I mean? Yeah. And also, for us wise.. I mean, nurses work 5 days right. But you know, when we work in night shift, the night- night- [NJCL: Participant gesturing, pointing fingers to represent the week] we work night shift, it’s an off day.. But the off day includes in the 2 days of the off day. You understand what I mean?. So, [NJCL: Participant gesturing] night day, night day, by right, for students, we have sleep day off day, alright, but our off day is the sleep day. Yeah. So. I mean obviously is a scam. [NJCL: Participant smiles] But..
- **NJCL: [Laughing]**
- P7: You know what I mean.
- **NJCL: I- I- From my own experience. Yeah, I know what you mean. I understand.**
- P7: So for us to have the motivation, to even work- To even go and have a full day of exercise. That is only one day. And for that one day, it's very precious. Yeah, that one off day. Yeah. So.. So I think mentally, it affected me to be more lethargic at work. And also, yeah, to dread work.
- **NJCL: Okay, then. So.. Other than being more lethargic at work and actually mentally dreading working, can you think of anything else that- That impacts your work performance due to your lifestyle habits?**
- P7: I think.. [NJCL: Participant murmurs, eyes are closed] Yeah, I think I think that's about it. Yeah.
- **NJCL: Alright. Okay. Okay, then, for my next set of questions, it is regarding the workplace.**
- P7: Mhm. [NJCL: Participant is close to the screen, nodding]
- **NJCL: As a nurse, do you feel that the workplace influences your own lifestyle habits?**
- P7: Yeah. The type of friends that you make in the workplace. I mean, firstly, the work rotation shift. Cause my partner, my boyfriend in a sense, is walking in our clinic. He's also a nurse. He’s working in a clinic. He has a more.. He has a routine lifestyle in a sense. After work he can exercise, then he can. You know, he has the freetime and sleep. The next day is also off day in weekdays. He has 2 full weekdays to have his ‘me time’, but for me wise, though, like what I say, the shift wasn't all we- I don't.. I have to plan one month ahead. You know, if I want to get something done, if I want to meet my friends, or if I want to exercise or go out to exercise, I need to plan one month. But the.. So that's the rotation. That's the shift issue. And also the work load, sometimes, if we cannot finish our work on the day itself, we have to stay back and finish, like our notes and voice for the patients. So that one also eats into my personal time. Yeah. So sometimes, if I let's say today, I- I end work at 3, but I have to… But because of this, I end work at 4. So my lunchtime is at 4. You know what I mean. So so I think that is one thing. The second one, I guess, is the friends that you meet at work. So I will say that my friends.. My colleagues and friends in work are not bad.. They sort of encourage your health, your lifestyle, but also sometimes they also have to treat. So after PM shift, they will want to “Hey let's go to have supper”, “Let's go for a drink”. So I guess. Yeah, I guess it also affects. But.. Uh. So the company wise, they actually implement some stuff like health check-up, free health check-up for nurses. Yearly, annual health checkup, for instance, the one they implement to help to sort of, motivate us, to have a healthier lifestyle. Then also, they also implement activities like. there's this thing called what… Physical 100, I mean it’s the adaptation from the Korean show. But they also implement this event in in the hospital so that we can join. Yeah.
- **NJCL: Ah, sorry, could you elaborate more on this ‘Physical 100’?**
- P7: Oh so.. It's a work event. So if you want to join, you can join them. So you go there. Then it's like a competition, ‘which ward is the most fittest’. So there's a series of games for us to play. So it's like a carnival. But physical. Yeah, physical. Yeah, you have to. [NJCL: Participant is gesturing, imitating walking] Duck walk, metres. Yeah, and running and all that. And [inaudible]. You know. So yeah. I think on a on a larger scale, the company tries to let us have a healthier lifestyle. Yeah. But don't think it's working. Sometimes we [inaudible] [NJCL: Participant smiles]
- **NJCL: Why- Why do you not think it's working? Personally?**
- P7: Personally, I- why I don't think it's working? Because it's an annual thing. Yeah.
- **NJCL: As in the- The frequency is the problem?**
- P7: The frequency of the event?
- **NJCL: Can share the event?**
- P7: It's a annual thing, then.. Yeah. I- I mean this for my company, but for.. if I know for other hospital like [Public Hospital Name], they have a yearly NAPFA test.
- **NJCL: The nurses?**
- P7: NAPFA, you have to run 2.4.
- **NJCL: As in, it's organised solely for the nurses or?**
- P7: Yeah.. In [Public Hospital Name], I think so. Yes. You have to pass the NAPFA test if I'm not wrong.
- **NJCL: Wow.. [Laughing]**
- P7: But- But I not sure.
- **NJCL: I see. I see. My first time hearing about it as well. Okay, I think, moving back a bit, you mentioned something that's related to my next question. So my next question is about, how do you feel about the workplace culture among nurses, in helping to facilitate or hinder maintenance of healthy lifestyles?**
- P7: I think in plays a very big place, though, because we see our colleagues everyday, and then we have lunch with them every day. Then sometimes it's also. How do I say.. The FOMO? If let's say you don't have lunch with them? [NJCL: Participant smiles, adjusting hair] And then.. So, because usually one person will volunteer to buy food that day. So if let's say that person want to eat Mala that day, everyone has mala today. [NJCL: Participant smiles] If you understand what I mean. So if one person have teh ping today- One have a bubble tea today, everyone will have bubble tea today. Yeah. Yeah. Oh, peer pressure, that’s the word. Yeah. Then.. So it affects. And then sometimes for night shift post night. Then they were like, “Hey, we work 2 days straight together. So it's 24 hours together, right. So let's have breakfast together”, and everyone must have breakfast together, I guess. Because my- For my ward, the culture is quite good. Everyone's- Everyone is friendly. So I think that's why there's more peer pressure. Yeah. [NJCL: Participant smiles, nodding]
- **NJCL: I see..**
- P7: So I guess it affects my- my diet, because if let's say I want to- If one person wants to eat mala, “Hey, can I get something healthier?” Then.. You know the person who is buying the food have to cater to my choice, and then go to another place to buy the food. Yeah.
- **NJCL: Okay..**
- P7: Hm! You said what, sorry?
- **NJCL: Yep yep, just agreeing. So, is it right for me to say that, because of the workplace culture among nurses, at least in your ward right, it will hinder your ability to actually maintain a healthier lifestyle cause you don’t want to be like, in other words, like, extra like, make the person have to buy something different, when everyone is buying unhealthy food. Something like that?**
- P7: Right. And [NJCL: Participant smiles, adjusting hair] I tried this experiment before in the.. In the starting of the year, you know, starting of a year, we always have goals? Then I said, Okay, let's- I want to be vegetarian for one month. So I told every single person in the world, I'm being vegetarian, don't give me food- Don't give me meat. But after two weeks it failed. Cause everyone started to spam me with food. You know. So I guess.. [NJCL: Participant laughs] I think it really affects a lot. I think that the colleagues that you hang out with, it will affect you. Yeah.
- **NJCL: Okay.**
- P7: Hm!
- **NJCL: Are there instances where your colleagues, or like the workplace culture, instead of like hindering you, they actually facilitate and have a healthier lifestyle?**
- P7: Yeah! Sometimes we would want- Sometimes they would jio me to go and cycle. Yeah. And sometimes they would- Like the physical 100 event. Oh, I didn't mean to volunteer for it, but they just put my name down, and I have to go. You know what I mean? Yeah. So.. Sometimes they don’t, sometimes they do help me in my lifestyle goals. But sometimes it depends. Yeah.
- **NJCL: Then you overall. You think the workplace culture actually facilitates more or hinders more, your healthier lifestyle?** **Yeah, the culture.**
- P7: To be very honest, I think it hinders.
- **NJCL: Okay. Why do you think so?**
- P7: Because of the peer pressure, they ask me eat the same thing with them. And then I don't know how to reject.
- **NJCL: Yeah. Hmm. When you say you don't know how to reject, iIs this like, is this a personal thing, or like a workplace culture thing?**
- P7: I think it's a personal thing, yeah. [NJCL: Participant laughs]
- **NJCL: Okay. Okay.**
- P7: I think because sometimes, my my friends, they'll help me buy food. Okay for me. Usually I like to stay back and do all my work nicely before I eat, so sometimes they will be like, ‘Oh P7, want me to buy food for you?’ and I say ‘I don't want to eat. I’ll go home and eat.’ Then they will be like ‘why you don’t want to eat, you must eat! You never eat the whole day!’ Yeah. So in the end, if I don't even ask, they still buy.
- **NJCL: I see**
- P7: Yeah, I think it's nice, though it's nice.
- **NJCL: Okay. Then, moving on to my next question. It’s.. What do you think about the role of nurse managers in helping nurses maintain their own health?**
- P7: Nurse managers..
- **NJCL: Yeah, the brothers and sisters.**
- P7: Hmm. No, my sister just encourage me to go for all the events and go for health checkup and all. But mostly not much, not much. [NJCL: Participant adjusts hair] Yeah. And they are also seldom on the ground working. Because they- they are doing other admin stuff that are important as well.
- **NJCL: Well, moving forward, would you say that- Do you see that the nurse managers and the nurses on the ground are not that close?**
- P7: Sorry? Not that close?
- **NJCL: Yeah. How will you describe the relationship between the nurse managers and nurses in your ward?**
- P7: I think, for our ward personally, the nurse manager, my sister and the nurses are close. However, most of the time she's not present. Yeah, because she really has. She has her own responsibilities to do other stuff. So yeah, so.. even if sometimes she wants to help out in the ward, she cannot. Because you don't have the time management and have to go for meeting, so I don't think.. But as much as she's. She's close to us. There's still a hierarchy system there. Yeah. I think even between nurses, there's also a hierarchy system like the senior nurses and the junior nurses. So.. sometimes, even if I want to be close to another, senior nurse, is also quite difficult.
- **NJCL: So saying that this hierarchy, this system is actually- is a barrier, in nurses. Between the relationship of nurses**
- P7: Yeah, very much of this..
- **NJCL: Then, in the ideal situation, do you feel that nursing- the nursing managers should do more? Let’s help nurses maintain their health.**
- P7: Hmm! I think in the end., the decisions, the health lifestyle decisions that we need is based on ourself. But I think the managers wise, the most what they can do is help with regards to the manpower issue. I don't think they can do much as of now. [NJCL: Participant sniffles]
- **NJCL: Okay. Moving on to my next question. I think you did touch on it. But how can your institution help- help you to maintain a healthier lifestyle? I think you mentioned like Physical 100.. and some activity they organise?**
- P7: Health check up as well. They do yearly have check up. Yeah, I think. Oh, yeah, also, because we.. If let's say we were to be sick, we can go to visit the health clinic. Oh, sorry. The staff clinic. It's 24 hours- I think it's 24 hours, and then it's free consultation
- **NJCL: Consult.. Okay.**
- P7: Yeah, I think the benefits for nurses in terms of health benefits, I think not bad. Like dental we get.. I mean. [NJCL: Participant gestures] health, lifestyle changes and health.. Let's say, if we get into really health situation like we get hospital leave. We also get dental checkups. I mean, not dental check up. We get dental reimbursement. The staff clinic we go to. Consultation is free, we get insurance as well. Hospital insurance as well. Yeah. So I think the hospital is really trying to help. If let's say something, were will happen to us.. Yeah.
- **NJCL: Then, could you elaborate about the- I- I think you mentioned, like the organised activities? Nurses help you to encourage you all to have a healthier lifestyle..**
- P7: Yeah
- **NJCL: Can elaborate on that?**
- P7: So sometimes the hospital will help with the.. They would organise events like, let's say, earlier, I say the Physical 100. They will also organise events like, yearly health check up purposes. Other than that, I think they recently started this thing whereby.. [NJCL: Participant puts up hand] Pauses. Wait. I go and see the Whatsapp.
- **NJCL: Okay**
- P7: Sorry. I see the Whatsapp. [NJCL: Participant checks phone and mumbles] Where.. This EMD, EMC trip. ‘Every moment counts’ [NJCL: Participant puts phone down and back to the original place] So yeah. So recently, they started this EMC Trip: ‘Every Moment Counts’. So every week they will give out healthier treats, healthier snacks to nurses. So this week they gave out vegetable chips. Honestly, not nice. [NJCL: Participant smiles] Yeah, but- Yeah, but that's what I think that is some of the steps that the hospital is trying to make. Because I guess, nurses [inaudible], they just snack at the nursing counter. [NJCL: Participant gesturing, imitating eating chips] Yeah, I think, yeah. So events, I guess that's what they are trying to do as well as for some insurance policies.
- **NJCL: Do you feel that institution is doing enough help for nurses to have a healthier lifestyle?**
- P7: I think that's the most that they can do. But in the end it still comes back to us. The nurses themself. Yeah..
- **NJCL: Can elaborate on that?**
- P7: Uh, like as much as they can give us all the healthier snacks, as much as they encourage us to go for those events or any other health screening. Is- In the end is whether- It's up to us if we want to join or not, because all of these are voluntary. Yeah.
- **NJCL: Then, moving to my final question**
- P7: Hm!
- **NJCL: Then, outside of work, what do you think influences lifestyle?**
- P7: Outside of work? [NJCL: Participant stares outside of screen] Social circle, myself. Yeah. I think determination and purpose is very important. The nurses themselves needs to have peace.. Determination, and purpose. And if we don't have, I don't think we'll even start on having a healthier lifestyle.. And social circle also. Hm.
- **NJCL: Social circle, as in?**
- P7: The friends at work. Yeah. Like our.. Friends and colleagues. But also, oh no, all my friends are nurses. obviously friends. Yeah.
- **NJCL: [Laughing]**
- P7: [NJCL: Participant laughs] So it doesn’t really help..
- **NJCL: So, how these social circles influences you is how- How exactly?**
- P7: So, for my friends that are not nurses.. They would tell me, “Hey, let's go up to hike..” [NJCL: Participant gestures, rock climbing] What's the thing called? The climbing thing?
- **NJCL: Uh.. Rock climbing?**
- P7: Yeah rock climbing! [NJCL: Participant smiles, scratching head] They ask me go for rock climbing and all. Then, I mean, I will go. I'll get very tired and I’ll complain a lot. But for those, nurses friends, we'll just meet up, sit down, just don't worry. We don’t move. Yeah. So I think that type nurse- That type of friends that you have are quite important. I think. Yeah, if you were to ask any nurse, they'll always tell you that they are tired.
- **NJCL: Okay. You also mentioned self, as you said myself. Like, as an influence outside of work. Elaborate on that?**
- P7: Hm. For me to have the determination to plan what I want to do like for me to want to exercise on my work- off day. I think that one takes a lot of determination and a lot of planning beforehand. Cause, like like, yesterday, I wanted to zoom call you, and then, after that I go for like a light jog or something, but in the end, I have work. So it just destroys the whole plan, you know what I mean. So I guess for me to really want to have a healthier lifestyle.. I think it takes a lot of determination. Yeah. [NJCL: Participant nods] Yeah. Because a lot of times, especially for junior nurses. Oh, you will get called back a lot, there's not- not a lot of manpower. And also, we will have more night shift. Yeah. I don't know, because, for those senior nurses, they usually have their family. So most of the time, weekends they cannot work. Or most of the time. They cannot really work night shift because they have young babies, below 2 so so, unmarried nurses.. [NJCL: Participant laughs] In a sense. All those younger nurses have to pick up those extra night shift. So now for us, every week I have night shift. Yeah. But maybe, for you know, the senior, more senior nurses, it's not that often. Yeah. Yeah. So so for me to want to use my only off day in a sense, to do something productive with regards to my health. I think it's- It takes a lot, you know..
- **NJCL: I see. I see. I think, you mentioned earlier about your family situation. Could you just reiterate to me how this impacts or like influences your lifestyle?**
- P7: Oh, okay, so usually for me. After work,, I will go home and take care of my grandma. My grandma is quite independent. However- I'm grateful of that- However, because you know that a lot elderlies come in for 4, and a lot of 4 causes a lot of elderly causes a lot of things. So I think it- it cost me to be scared. It made me scared. “Oh, what if this- What if my grandma fall?” So, after work, I faster go home and then make sure that that I'm at home so that I'm present. And if something were to happen to my grandma, I'm there, you know what I mean? Yeah. And then from.. I mean. Yes, my mom sometimes is at home. My mom's at home to take care of my grandma, but she also needs her own me time. So. So, in a sense, if my mom wants to go out or something, I have to be home, at least one person needs to be home. Yeah. So if let's say I want to go out to have you know, to jog or meet my friends. I need to make sure one person needs to be home before I can go out. So I guess a lot of fear is a lot of fear that sort of affect my lifestyle.
- **NJCL: Yeah. By fear, you mean like fear of what will potentially happen?**.
- P7: Yeah. What will happen to her if no one is at home. Yeah.
- **NJCL: Okay. Alright. Then I'll just summarize what you briefly mentioned to me. Outside of work, what influences your lifestyle. You cited that your social circle, like your friends outside of work, or like, see your nursing friends. Then you also talk about yourself, like having the determination, plan what I want to do and be productive, that kind of thing, and like having a- having a purpose. Then, lastly, it's about, how have family take care back at home. Is that all? Is there anything else you think influences outside of work?**
- P7: No. no. yeah, think I'm just lazy. [NJCL: Participant smiles]
- **NJCL: [Laughing] Okay. Could you elaborate on that?**
- P7: I love sleeping [NJCL: Participant laughs] Also. I think. Cause in the workplace, a lot of time- Not a lot. Most of the time, I’m mostly standing. Or running around. So by the time you you know you end your shift, you're already quite tired. So when you go home, you just want to relax. You just want to lie down on your bed and sleep. Yeah. Yeah. And also because our ward is a geriatric ward, a lot of people are bedbound, a lot of patient patients are not mentally there. A lot of time during shift, I need to restrain a patient, that one takes a lot of strength. And then also sometimes uh, yeah sometimes need to restrain a patient while changing diapers, and then a lot of patients are much more bigger than- Heavier in a sense. Especially male patient. But we still have to change ourselves. Because there’s- just not enough manpower. Yeah. So by the time you end work, your back is painful, your leg is painful, you just want to go home. Yeah.
- **NJCL: Hmm. Ok, I understand.**
- P7: Yeah.
- **NJCL: Okay. Do you have anything else that you'd like to speak about that wasn’t mentioned in the interview? Any further comments you want to share?**
- P7: Hmm. I think.. I mean, it sounds like I'm complaining about the work. But in the end, I guess there's pros and cons. Yeah. There's pros and cons. I mean. It depends on yourself and the friends that you hang out with. Yeah, that really affects your lifestyle and also your here. [NJCL: Participant points to self] Myself especially. I think in the end, the most important factor is ourself.
- **NJCL: Yeah, I see. I think that was very insightful. Okay, sorry. Do you have any more comments? Any- Anything else?**
- P7: No.
- **NJCL: Nothing. Okay, then this marks the end of the interview. Thank you for your time and participation.**
- P7: Thank you! Am I answering [inaudible] question?
- **NJCL: Yeah, can, can. Thank you so much.**
- P7: Ok, thank you.
- -End of transcript-

# **Interview #8**

Participant and interview details
 Participant number: 8 (P8)
 Date of interview: 27 Sept 2023
 Interviewer Number: Navarro John Christopher Lambino (NJCL)
 Time IDI started: 2000
 Time IDI ended: 2115
 Observational notes: Participant seems energetic.

Section I: Interviewer’s summary
 The individual interview was conducted via Zoom. P8 was very relaxed and casual during the interview. Used various colloquial terms during the interview. For context, participant is a private hospital nurse. He transferred from community hospital after 8 years due to lack of career-progression. Provided various insightful tidbits on the comparison between private and public/restructured hospital in Singapore. He was very forthcoming in his sharing. Interesting points put up by participant is regarding forming an identity outside of nursing as evidenced by his various volunteering/physical activities he participate in outside of work. He also mentioned about finding meaning. Mostly, his responses have been about his intrinsic factors. The idea that forming a healthy lifestyle will be mostly from within someone. Additionally, very interestingly, he mentioned about having ‘CREDIBILITY’ as a nurse who is obese may not be heeded by patients when listening to dietary advice for example. He also mentioned about the struggle of transitioning from ground nurse to a supervisory role, as there was lack of formal training program. Basically, the difference between public and private is made apparent. As a nurse with a supervisory role, he says nursing managers role to help others nurses self-regulate, its very difficult and can only act as facilitators.

Section II: Transcript
**NJCL: Alright. Good evening, sir. Firstly, I would like to thank you for taking the time to speak with me today. My name is Christopher, and I'm a NUS Year 4 Nursing student. The aim of this interview is to get a better understanding of the perspectives nurses have on self regulating the health behaviours, and how various factors may influence this. I want to find out what are your views, opinions, and experiences on this. I would really like to encourage you to speak on what comes to your mind. There are no right or wrong answers. May I have your permission to continue video recording the session?**

P8: Yeah, yes.

**NJCL: Alright, thank you so much. And then let's start with some basic questions like, which ward are you working in right now?**

P8: I'm working at [Private Hospital name]. It's a private ward. Yeah.

**NJCL: Could you tell me more about like, what kind of work you do and the workload?**

- P8: So I am currently in a supervisory position. So it's a bit like ward manager. But then, in [Private hospital name], we don't use like, the terms, nurse manager, everyone is just a nurse clinician, nurse assistant. So so. But but my my job scope is in a supervisory position. But then, that being said, we unlike some hospitals, where those nurse manager just sit in the room all day. We are quite hands on like like like- For example, in my morning shift, I will also help out on the ground. Until about maybe 11 or 12 o'clock before I go back to the office to to settle some administrative stuff lah, yeah. Yeah. So that that's the nature of my work lah.
- **NJCL: Hmm! How will you describe the workload for you?**
- P8: Workload ah? Uhm. It's.. It's manageable lah, I think, but like, because, like, how to say, like compared to nurses who take case, right, like my my ground nurses would take case, after they hand over to afternoon shift. Technically they can. They can relax a bit, then go home, you know, on time and all that. But for us in the supervisory position. Your work never ends, kinda. Like- like, see just now I mentioned right? 11 plus , 12 plus, then I go back to office then there are complaints to settle the roster- Roster issues to settle.. They then... And then, like, for example, today, I started my work at 7 AM, right. I only left work at about 6 o'clock. So that's nearly 12 hours. That's why there’s always extra time lah, right my shift should’ve end at 4. But because last minute there was a complaint to handle, so I had to stay back to settle. Yeah.
- **NJCL: Is this like, overtime, very common?**
- P8: Uh.. I will say, it depends on when.. On self control, like I. I've known colleagues who they they know they know when to stop and then they know when to like. “I'll- I'll continue this tomorrow”, cause. Actually, technically speaking, a complaint is not time urgent lah, you know. I- I could have just left it till tomorrow to continue, but it’s.. It’s a bit- like a bit of OCD. You know. You don't want it to be nagging on your mind when you go home and all that, and plus, you know the the customer. The patient is waiting for a reply to her queries. So in this particular case, this patient was asking for a discount because dissatisfied with something lah. So I thought, it's also better not to let her- leave her waiting ah, right? So, so, so, so. It's a personal choice lah. I do know, colleagues who, who who knows their limit. They will. They will end work on time. They'll continue it tomorrow. There are colleagues that will do that like, yeah, yeah. Yeah.
- **NJCL: I see.**
- P8: Yeah.
- **NJCL: Uh. Were you always working in [Private hospital name]?**
- P8: Uh, no, actually, it's- I've only been working at [Private hospital name] for one year. In fact, today is by one year mark. [NJCL: Participant laughs]
- **NJCL: Oh, congratulations!**
- P8: So previously for for the for the large part of my career, I work at [Public Hospital Name]. Yeah. So government government sector, yeah.
- **NJCL: If you can make a comparison between your previous workplace and your now work place. How how you describe the- How would you compare them?**
- P8: Uh.. So the thing is, when I was at [Public Hospital Name], I was a ground staff. So my job scope is different. And [Public Hospital Name] be the typical one. You know, you take your case, do some maths, do your doctor’s’ plans, changes. Then, after that, hand over and then go home. You know that that was that was the job I was doing at [Public Hospital Name] lah. That being said, the difference is, your your patient demographic is different. Government sector, you get your most most largely majority is the local patients. Cause. I was in a sub. I was in a typical normal ward, one of the private class ward. So. So. So you just get all your your typical elderly uncle, aunties, these subsidized kind. But then, like in in [Private hospital name], you get a lot of rich people, foreigners. A lot of people come in just for for small operation, and then they go home. The turn over rate- The turn over rate at [Private hospital name] is much higher, like the patients come and go much faster compared to a government hospital. The doctors you're working with is different. Government hospital. Your hospital, your your patients- Your doctors, are mostly- You. You get your like, HOs, FOs, or, in the case of- in the compass, I was at your resident physicians. Yeah, then, then, like one consultant covers, maybe 2, 3 wards. So so you don't see him as often, you know. You see, you see them when there's only a special case discussion, or during your multidisciplinary meeting rounds. Then then you get to see the consultant. Uhm. There's the government sector lah.. But like in the private sector. Every single doctor is a consultant. Yeah. They only have, like, like 5 or 6 resident physicians only. They only come up when when you need them, to do some procedure which the consultant don’t want to do, you know, like IDC insertion, and I think, too simple for the consultant to do ah, then you encounter the the resident physicians at [Private hospital name]. Otherwise, you are dealing with the consultants directly. Everything which is.. Different lah. [NJCL: Participant stares off screen, nodding] Cause like. When you're dealing with MOs, resident physicians, you you can be more like, like.. Easy going. More- Like, less formal with them, you know? You can “Hey doctor this one order for you ah” That kind of thing. But then for consultants.. You have to be. You have, to be sure, you know, like, this is the correct thing to ask or tell him, if not, you're gonna get scolded. [NJCL: Participant shakes head] Yeah, yeah. Yeah. Alright. Yeah.
- **NJCL: Would you say it's common to get scolded more in your current institution compared to the previous one?**
- P8: I think learning ah.. Learning is.. I think, partly self-driven. If if you have the the curiosity and the and and you know what to ask, actually you you can learn in both, either places ah. I mean. I've also encountered people who are just doers like like, for example, the doctor order this blood, Doctor order this medication, then they will just literally do this blood, and then give this medication without thinking, considering like, oh, because patient has this condition. That's why we are doing this blood then, you know, like learning, probably, you know, like “Ah so next time, if I see this same type and no one sees, I'd probably- They will do the same management”. Like. You will encounter lah. Once you graduate and you work with your future colleagues ah. I think, a large portion of people are doers now. Because to to be, to think ah, to be a thinker, you take a lot of time.. So like. For example, right, uh. I have colleagues who can serve medications 2 or 3 times faster than me. But then they are literally just looking at meds, looking at meds, and then serving ah. They they don't really like like think through and all that, or or like cash problems. Like, for me right, I- When I used to serve meds ah, when I was at the previous institution. I I can. I am considered quite slow lah. But then, while I am slow, I feel like I also pick up problems..? You know, like some some medications have to be served, previewed, so some of the meds have to be previewed and all that. Then very often the people who are poor ah, like they chop and don’t let me finish my work ah, they don't catch this kind of thing. But like, I feel like, I- I do catch like, “Hey, this medication wise, you know, not scheduled in the correct timing.” Yeah, you know that kind of thing. Yeah. Yeah, yeah. So I think so. So then, similarly, like, in in a private setting, right? Where doctors are all consultants right. If you are willing to learn, and you ask the right questions, the consultants will explain to you. Okay, depends on which consultant lah. Some, some, some are really more impatient than others lah. But, generally speaking, if you, if you ask the right questions and all that. They are willing to explain themselves lah, then there's learning already lorh right. But then of course you can be also a doer. Whatever the consultant order, you just do as per what you write. Then you're- Then they're not gonna learn. You're not gonna learn anything yeah. So so if you've asked me if I gonna evaluate whether- Is there a better learning experience than- If I'm a public sector, if I if I. If there's a link better learning experience in the public or private sector, I would say, it's up to the individual. Yeah.
- **NJCL: Okay, okay, thank you so much. That was very insightful, considering the differences between public and private. Then moving on to my next series of questions. It will be regarding, like specifically on lifestyle. As a nurse, how would you define a healthy lifestyle?**
- P8: Okay, I feel that we need to have hobbies out of work. I think too often. Or maybe they people just don't want to say first, when I used to like chat with my colleagues on that, I'll be, I'll ask them. So after after shift and going back, very often the- the default answer people will be like, ‘I'll go home and rest more’. Cause they’re tired, you know. Lie on bed, watch show, or sometimes like. Sometimes people have a standard off days, 2 or 3 day off. Or maybe during their leave, they're going back to their country and all that, and you ask them “Hey what you do ah?”, then they say, “Oh, I we I do 2 hour turning in my bed”, [NJCL: Participant smiles at camera] you know that kind of thing, like like. I don't know if they're being literal or like, they just don't want to share so much. So they just give you a very like bland people answer. I cannot say for sure lah. I I I I do have to to say that I am not like super close with anyone, so maybe they just don't want to share lah. Yeah. But but in any case, I I feel that having hobbies out of work is important. Because, like you can-, you cannot make work your your whole identity ah. Then like, touch wood lah, if one day you lose your job, or what. Then like. I think people who make work their entire identity will will face identity crisis. Yeah, yeah, so so like, like, like, maybe having a voluntary welfare organisation that you volunteer in, you know that kind of thing, and having then, like, like, as you become more senior in the in the voluntary Welfare organisation, you you have a secondary identity. Like. Yeah, like, like, if someone asks you like, what your interest, you can be like, “Oh I'm doing this as well”, you know. Then, or like hobbies also can include like, like exercising. Picking up certain sports and all that lah. So so so hobby I I what I mean, when I say hobby, I don't mean like, just like crafts and games, or like flower arrangements, whatever lah, [NJCL: Participant waves hands] Everything that is done in in for the purpose of recreation is hobby lah. Yeah, yeah, yeah, yeah. Yeah. So so so, so, so, yeah, that's what I feel lah. People should have a- have a hobby out of work. It takes their mind off work also, you know, so that they have a better work life balance kind of situation. Yeah.
- **NJCL: Can you elaborate like taking their mind off work and what you mean by work life balance?**
- P8: Okay. So like uh. Before I came over to my new workplace, while while I was still at my old workplace right, I used to do a lot of uh. Martial arts. Uh. I was, I think, at a point of time I was simultaneously doing about 3 different kind of martial arts? Uh. Taekwondo, of which I obtained a coach licence. Which is kind of what I talked about just now lah. Like like like, becoming so senior in your hobby that you you- it's an alternative identity kind of situation. Yeah. So I I did my taekwondo until I was a second done black belt. Then uh, I did a Singapore program where I got my coaching licence and all that. So technically, if I lose my job as a nurse now, I can actually still be employed as a coach. Save income. But I'm not. I'm not working as one now ah, I'm just saying it's it's a it's a it's a parachute, you know. In case, yeah. So yeah. So I did. I did taekwondo. I did jujitsu. And then I also did historical European martial art, sword fighting. Yeah. So. Uh. it takes my mind off of work in the sense that like like, let's say, okay, if you're angry work, you know all that physical activity, all that kicking and punching helps you vent a bit lah. Yeah, then, plus it's something entirely unrelated to health care. So so while I'm focused on learning a technique trying to implement a technique or while I'm sparring, my mind is totally off health care, totally off work. It's it's focused on something different, different entirely lah, which is I think it’s helpful ah. For for for me to to to to destress. If if something at work is distracting, it’s it’s being, is is occupying my mind. Yeah, at least for a while ah, for 2 hours at least. Yeah.
- **NJCL: I see.**
- P8: Yeah.
- **NJCL: Then, moving on to something a bit more specific, like, you mentioned a a few like physical activities that you did right? So like, how do you feel about your current lifestyle right now? Maybe in terms of physical activity.**
- P8: Yeah, okay yeah. So so it was uh. When I changed organisation, right. Actually, private sector. They don't follow the the public sector's work hours. So instead of having 2 off days per week, I had only 3 off days per 2 weeks. So it's alternate ah. 2 rest day, 1 rest day, then plus, I told you about how like sometimes I have to stay back after work and all that. Then, plus my my new, my current workplace is actually further from home than my previous work place. So all the factors add on together, I I realised that it was very difficult for me to to make it for my- like for all my other, my martial arts, and all that. So actually, I gradually drop one, drop another one, drop, so I see, I think by December last year I dropped the historical european martial art. And so actually, currently, I'm not doing any sports at all ah. Yeah. Yeah. So if you want me to compare like, oh, how was I physically- Now versus, just say 1 or 2 years ago, I would say that I was physically fit. Fitter ah, fitter ah, fit is a very big term ah, I was not fit by any account lah. [NJCL: Participant smiles] I was physically fitter 1 or 2 years ago. But I think, is, I have to strike a balance lah. I feel like, if I am like tired from work, tired from travelling, and I'm still dragging myself to something that's gonna make me more tired. And then in the end, I would just have have a net, net loss of rest and energy lah. Yeah. So so I I decided to to. It was a hard decision lah actually, because actually, I'm someone who who treasures like like, I just- Like just now I mentioned, really about like, you know, like being see- Being so long in doing something that you know you're senior enough in something, that that it gives you a certain state- “Status” lah. In quotations lah. Like like I was doing taekwondo since my uni days until I got my coach licence. I was doing historical European martial arts since 2-0-1-7 til last year. Yeah, yeah. Yeah. Yeah.
- **NJCL: Oh my god.**
- P8: Yeah. So so I'm someone who who treasures like the time put into something. And so it was really a very hard decision lah. Maybe when my work is settled, settle down. Or maybe, if let's say I I I get to move house to somewhere new closer to work or what, I may pick them back up lah. Yeah. But at the very moment, at the current moment, is difficult to to balance. It. Balance the things for time. Plus the cost eh. I also realise that, like you, you do a monthly subscription, monthly course fee for something that you barely attend ah. Once a month, or sometimes even 0 times ah, you’re just throwing money down the drain also lah. Might as well stop it, for now, once your your schedule is more settled, you go back in. Yeah. So, so, so I had to consider all those things ah. Then, but- In in any case, not that I don’t- not that I don’t do- that I totally don't have any activities whatsoever lah. I I am very active in volunteering. I I volunteer for my My Grassroots, I volunteer with NUS alumni. I'm on the ExCo. Uh. I volunteer with SNH, [inaudible] Association. I am the editor for the Newsletter, like like so is like, I created, spending more time in physical activities with time spent in more mental activities…? Like like things that are not so physical. But use my brain more lah, yeah, yeah, yeah. So so so it's not as though, Oh, I’m like the ‘go work and come home sleep, go work and come home sleep’, I’m still doing a lot of things. It's just- it's not exercise related.
- **NJCL: Ah.**
- P8: Yeah Yeah, yeah.
- **NJCL: So would you say, these mental activities is uh, is a way for you to disconnect from work?**
- P8: In a way it does, because now, in my volunteer activities, we are coming up with ways to to help people, you know, help residents and all that. So so it it does take. I think it. It's both. It does take my mind off work, in a sense, but it also. There's also a certain, what you call that, like synergy with my work. Cause like like volunteer with SNH, which is nursing related. It's still- is still nursing related lah, but not in the direct clinical sense, you know. It's more of a ‘How do we promote this thing to nurses?’ ‘How do we fight for more welfare for nurses?’, is kind of nursing. So it's a bit different or same as the NUS alumni also. As the NUS alumni is like we are, we are. We are thinking about how we can outreach to fellow juniors. You know how we how we create activity- come up with activities, organise activities that that alumni are interested to come and you know, network and bond in. So so it's like, it's still nursing, but not really, not really work related. So it it does take my mind off of work lah. Yeah.
- **NJCL: Okay. Then, moving on to something a bit specific again, specifically diet. How do you feel about your current lifestyle? And do you feel like changing it or anything?**
- P8: Ha ha ha, ok diet ah. Firstly, I- I don't cook. So uh. When I was living on my own, it’s mostly takeout food Grab. It's just mostly just Grabfood all the way. Uh. I don't. I don't specifically choose like healthy diet, you know, like what 2 portions of salad a day, or what, or eat fruits, or what, I don't, but because I have IBS… So I mean I do. I do watch out for certain things in my diet lah. I try not to take anything that is that has dairy. Because diary really will make me very bloated. I try not to take food that is too salty. Eh not salty, uh, spicy, not salty. Yeah, so, so, so things that will trigger my IBS I try to avoid. So then, when when on on days where I'm feeling particularly bloated, or what, then.. Then, I will usually just eat like lighter food, like fish soup that kind.. So so I don't. I don't purposely choose healthier food, but I do eat simpler, slash, blander food on occasions when I'm not fit. My my stomach is feeling more bloated lah. Yeah, yeah. And plus anyway, I also feel like as as I'm growing older, like, okay, I used to drink like bubble tea at like 100%. Used to ah. That was many, many years ago, but then like I don't know. Like as I grow older, I feel like ‘Wah the sweet is too- too overwhelming already’. So so nowadays right, I'm I'm even willing to drink 0% bubble tea. 0%, 25%, all that. Yeah, I mean, so so I'm not purposely being more health conscious, but somehow my my taste has has also.. What you call that. Evolve in the new way. Yeah. Otherwise, no lah, no, no, no purpose, purpose, purposeful conscious decision to eat healthy lah, yeah, yeah, yeah.
- **NJCL: Then, talking about this topic, like, what do you think will make you want to have a purposeful decision diet?**
- P8: Uh. If it is easily available. Like by buying, Taobao-ing, buying. Like. Yeah, yeah. So, for example, I’ve been eating out for so many years right. I realised that is difficult to get vegetables. Like, let's say, you're ordering chicken rice. What? Just 3 slices of cucumber, or let's say you are ordering fish slice bee hoon, there's no vegetables in fish slice bee hoon, maybe a few slices of tomato or something. Yeah. Yeah. So like, personally. I know. I know that's like stereotype lah, people don't like veggies and all that. Personally, I like veggies like, like, if I eat like the Chinese Zi Char style. I I like bean sprouts. I like kai lan, I like. I like broccoli, cabbage. I I’m totally fine with vegetables. But then, if you're not eating that style of food, Zi Char style or the or the other economic rice style right, it’s very, very difficult to have veggies in your food. Yeah. Yeah. So I think what would make me eat healthier is if it's easily viable lorh, easily easily available, like off the off the shelf ah. Then then, yeah, actually, I totally don't mind eating. Yeah.
- **NJCL: Okay. Okay, okay. Moving back a bit. When I asked you about what, how you would define a healthy lifestyle, I- I'll just summarise what you mentioned, you said that you did have hobbies outside of work like, forming like a secondary identity. Work can’t be your entire identity. Then you also mentioned about taking your mind off work and like having work life balance.**
- P8: Uh-huh.
- **NJCL: Is there anything else you want to talk about this, regarding like defining a healthy lifestyle?**
- P8: Uh. I mean, typically, you would say that healthy lifestyle is is eating healthy and exercising. But obviously right now I am neither exercising, nor eating healthy. [NJCL: Participant laughs] Yeah, but I don't. Uh, I will say that I am sedentary, despite not exercising. Oh, yeah, you asked me if I'm doing like moderate exercise 3 times a week, or half an hour, 3 times a week? I'm not doing that lah, but, like, you know, nursing work already OT most of the time, I think even per day is easily easily 10 000 steps, and then, I think, secondly, my my volunteer activities right? I- Also keeps me on my feet. You know I volunteer at CDF. I volunteer with with uh, CRT committee, emergency response team and all that. So I think so we do. We do like house to house visits, to give flyers about fire awareness and all that. So I think a lot of my activities are still mildly, moderately physically- Still, still, still a bit physical ah, probably still doesn't clock into the national recommendation. But I don't. I don't feel like I am sedentary lah, yeah, correct. So, yeah lorh. I I I don't know what more- much to add to the healthly lifestyle thing ah.
- **NJCL: Alright. Then, moving to my next question. It's with reference to your answers in the previous question about the healthy lifestyle. As a nurse, how do you feel about self regulating your lifestyle or self regulating a healthy lifestyle?**
- P8: Uh. So I think my old organisation, they they say that like as nurses, right, we need to be good examples. If not, we don't have the credibility where advising patients, you know, like, like, if you're obese nurse telling the patient to lose weight, there's no credibility. Yeah, right? So so I I do agree with this ideology. Uh, yeah. So I mean, relatively speaking lah, like, you know, I don't. I don't. I don't smoke. I'm not obese. so I I don't think I'm I'm too too bad, you know, as an example to to advise patients lah, yeah lah, definitely definitely. There's still room for improvement, I could still be better, exercise more, eat veggies more. Yeah. Yeah. I mean, as a nurse, yeah lah, you got all the, you got the knowledge from the patient. You got a knowledge of what you should do to stay healthy, and then then, when you don't do it as much, that's a bit of.. A bit of guilt. But I I will say that because I have not reached that stage where like oh, I I have a chronic illness, and I'm obese and all that, so may maybe the the little bit of guilt not not strong enough to to make me into exercising. [NJCL: Participant laughs]
- **NJCL: Ohh**
- P8: Yeah.
- **NJCL: Earlier, you were mentioning, like how there's no credibility to the nurse advice to the patient if like their own health isn't.. isn’t up to stuff? Will you elaborate more there?**
- P8: I don't know if you heard about it, but like, [Public Hospital Name] right, they don't hire unfit nurses. I don't know if you heard about it or not?
- **NJCL: First time..**
- P8: Yeah, because they they feel that right, that what I just say lah, like, if you're fat. And then you're trying to advise patients to lose weight or what, patients don’t believe you ah. ‘You also fat, you ask me to lose weight?’ You know that kind of thing. Yeah yeah yeah, so so it’s literally that kind of thing ah. Like like you, you can’t. You can’t tell the patients not to do something if if you are also doing it ma? Or like like, uh, for for example, more extra, more unrelated example- But you tell your let's say, let's say your your parent and you tell your kid ‘don't drink alcohol’. ‘Don't smoke’. Then you you as a parent, you drinking alcohol and smoking, your kid also like ‘Hey, kong kong one ah, I don’t believe you ah’, because so so I think, is in that sense that you have to. You have to lead by example. Yeah. So like, I tell you, you can, I can see your whole identity, you know, you have to find something outside. I am doing things outside a lot of things outside. And also, so I did. This is the kind of lead by example. Yeah.
- **NJCL: Okay. Then, just now you mentioned something about guilt actually like pushing you to change your lifestyle, can you elaborate?**
- P8: [NJCL: Participant smiles] Ah, I said that guilt is not enough to push me yet. Yeah yeah yeah.
- **NJCL: Oh, okay okay okay.**
- P8: Because you like, you you you know you’re supposed to eat healthy. You know. You know to exercise 3 times a week but then, because you’re not doing it ah.. So like, like, having the knowledge of it is the, is causing the guilt lah. Yeah, cause you'll be like, Oh, I if if let’s say, touch wood ah, that I develop a chronic illness then I be like ‘Yea lorh’ because I know the cause mah? In that in that sense, that’s the guilt, but then because I currently don’t have a chronic illness and I’m also currently not obese, so then, the guilt that is nagging at me that is nagging at me is not enough, you know what I'm saying? Yeah, I guess I guess. Touch wood lah. Let's say I I slowly become fatter and fatter, then then I guess that guilt will start nagging me louder and louder lah. [NJCL: Participant laughs] Yeah.
- **NJCL: Hmm. So is it right for me to say for you personally, it... Your your idea of like self self regulating lifestyle depends on your own, like health health condition?**
- P8: Hmm. [NJCL: Participant squints eyes, contemplating] Self-regulating ah.. Self-regulating is a very broad term ah, right. Like self regulating can also not just about health. It can also apply to vices, you know, like, like what I say ah, whether you drink, whether you smoke, whether you hang out outside too late, you know, sleep late, sugary drinks. Yeah. So so so it's a very broad term ah, whether I I link self regulating lifestyle to health and all that. Yeah. Like, I guess, like I said, everything is in moderation. Yeah. Yeah. So so as as long as you're able to to to regulate yourself in the sense that you do everything in moderation. Then you should stay relatively healthy lah, right?
- **NJCL: Okay, can. Then, I'll move on to my next question. It's about, what are your main facilitators, motivators or like, barriers in terms of like a self-regulating lifestyle?**
- P8: What are my motivators ah.. [NJCL: Participant faces away screen to think] Uh. So, like for me, when I used to do the martial arts and all that. Why I do so many, ah, it's not because of health and fitness. I'm not doing 3 martial arts, because I I want to stay fit lah. Not really. That is because I I have interest in in like, martial arts, self-defence, techniques and all that. So so it's it's it's driven by my interest in the techniques and not so much the exercise portion of it. Yeah, then, like, with regards to the volunteering and all that. I I don't like to stay at home so like if it's my off days, or like during my ALs, or what, I I don't like to just like laze at home, lay in bed, put on a show and all that. So so, so originally, when I started going out for to do like one volunteer activity, my very first one it was motivated by by me not wanting to stay at home. Then I figured, if I'm gonna go out and do something, I might as well do something that helps others, you know meaningful things rather than like, go out and waste time, loiter, spend money. Yeah. Yeah. So then, when you start doing one you realise, ‘Eh, if I I can do, I can do more’. So so so that I started wandering in more things. Related, related, the thing most other thing that I volunteered is healthcare related lah. -Ish. Kinda. CDF, yeah, I teach people CPR, first aid, then. And I volunteer with nursing alumni. And and SNA, both are nursing related. Then I volunteer with CRT, which is [inaudible] response team which which is kind of healthcare also. Also, you know, it's still CPR, is still First Aid. I also volunteer with health surf, as a cleaning assistant medication dispenser. So it's all still like healthcare related, you know. Yeah, yeah. So so it's like I I'm trying to tap on the the synergy synergy of like my knowledge base and then I not wanting to waste time at home, that kind of thing, yeah, and then helping others. So so my motivator, my motivator for this is is, firstly, to help others, and firstly, to not waste time at home. Now. Yeah, yeah. Yeah. Then, like, then, if you look at my other motivators, I just now mention about my diet. So physical condition lorh, you know, I got IBS. So you control your food intake in a particular way. So so unconsciously I'm I'm eating more bland bland foods on more occasions because my stomach cannot take it, you know, if I take too oily, too, too spicy food [NJCL: Participant scratches head] and all that. Yeah. So, so, so, yeah lorh. These are my motivators lorh. Yeah.
- **NJCL: Hmm. Earlier you mentioned something about like, meaningful things. Can you elaborate on that? Doing meaningful things?**
- P8: Uh. I think, okay, so what meaningful, meaningful is also a very broad term lah. Many people find different things meaningful. Some people some people want to want to watch their kids grow up, then that's meaningful, you know. Some people want to uh, achieve a lot in their career, and that's meaningful for them, I think for me, being able to to do things which which help others, you know. That's meaningful. And then I think last time I also thought about this before in the sense that, like. I don't know if you, if you have experienced this in your attachments and all that. But sometimes our local patients ah, quite unappreciative lah. They will say like, ‘Oh, I pay taxpayer money, so I'm paying your salary’, you know that kinda thing. Like ‘you have to treat me in a particular way’. They are very unappreci- unappreciative. And then, when you do volunteer work right. The beneficiaries right. They are actually very grateful. Then, when you, when you do those things, you know like, during my my clinic, my clinic assistant bond, volunteering thing assistant thing, and then doing my CDF thing, and then then you you meet beneficiaries who are thankful for your time, thankful for what you've done for them. Gives you, you know, meaning lorh, you know, like, like, if you like, okay, what I'm doing, it's being appreciated, you know. It makes a difference in someone's life. You know I'm not. I'm not doing it for- doing. Yeah, I'm like doing already and people still appreciate it, which is what some of the patient are showing already ah, Yeah. Yeah. So so I I guess that that gives meaning lorh. And then then I, as a tangent-tangent to what I mentioned at the start about about uh, what did I mention ah, like, I think, in a sense that it it. It makes me not give up on health care, you know, I tell you. If you just purely keep working in the hospital. No hobby, no hobby to distract your mind. No, no, something to renew your purpose and meaning. Then you'll realise that right, like working in healthcare, is very is is a very unappreciated- a lot of people- Unappreciative-. Unappreciative. But yeah, cause your patients take you for granted. You know. Then maybe your bosses ask for you to do more. You know their colleagues also may take advantage of you. Yeah, tha, I think you, yeah, yeah, it's like that. [NJCL: Participant laughs, adjusts hair] Work is like that. So so I think I think being doing my voluntary work out there, right. Like, gives me like a renewed look in health care, you know, like, like. Okay, yeah, what I'm doing is being appreciate. It it’s of use to other people. You know. Yeah, yeah, I I. And I think also because it's in a voluntary capacity. You don't have the obligation to, you know. Like, like, if it's a job right, on your day job right, you'll be like boss asked me to do extra work if I say no, she won't promote me, you know you know what I mean? There’s this thing weighing on you all the time. This is nowadays got a lot of the people like lying flat out. You know all the the new ideology they want to quiet quitting, you know, quiet quitting, lying flat, all the new terms and all that. So it's like, essentially, those people are just doing, they are saying that they want to do just just their job scope and nothing more, you know. But then, for people who wants to climb the current ladder and all that, you'd be like, okay. Boss asked me to do more I'll do more lorh, cause the more you do, then the more likelihood that you can show that, like you're, you're willing to do extra projects and all that right? So so I think the refreshing part about doing voluntary work is that you have no such obligation literally, I do until my clock out time by then I go. So it's like, is that is is a win win, you know, like. I feel appreciated and I don't have to do more! Yeah, so like, so like, for example, III have been volunteering with health surf. If you Google health surf, is like a migrant worker. They provide like health care, I think during COVID period they were quite active in helping to support the migrant workers. So I volunteer with health surf ah, for the past 10 years. Yeah, like, like, I mentioned again. I I'm someone who treasures time spent in something. So once I'm in it and I and and there's no strong motivator I will quit. I I won't like just just just stop doing that. So like, for example, for help. So I've been with them for 10 years. Since 1-3 since before I even graduated nursing. Uh. And essentially, in the past 10 years, I've been doing the same thing. Packing medication, dispensing medication, you know. Then going home. I I I don't. I don't do more. I don't stay back more. Yeah, so far. I mean, I think it's a win win lorh, you know it renews your app-, renews your appreciation, you know. For for healthcare you feel, appreciated for for helping people, and you don't feel the pressure of trying to fight for co- fight for promotion. You don't feel like, “Hey, why, he promoted before me?”. Nothing! Cause I'll be like ‘I'm just a fellow volunteer’. ‘I I'm here longer than you ah. So I'm just available’, you know. Yeah.
- **NJCL: Oh I see**
- P8: So I think that's how it's helping me. In like, you know, like find meaning, since your your topic was about meaningfulness lah. Yeah lah, I find that it. It's helpful in helping me find meaning in health care and helping others, and in the meanwhile it also helps to like how to say, like like buffer away the negative emotions from working full time in health care. Both are mostly health care like different the different sides of the coin of health care, you know.
- **NJCL: Yeah, I get. Okay. So I will just try and briefly summarise what you've been sharing here-**
- P8: Yeah
- **NJCL: So for the question, on ‘what are your main facilitators, motivators in self regulating your lifestyle?’, your answer is mostly by the fact, it's is it right for me to say it's mostly intrinsic? Such that, like even like your physical activity wise, it's not that you wanna be. Uh. It's mostly driven by interest in the martial arts themselves. Then also cited volunteering, your volunteering experience. And it's- This volunteering actually gives you the- like a meaning, like a sense of purpose, so that it actually buffers away the negative impressions you feel at work. Hmm, yeah, is that all your facilitators and motivators? Or is there anything-?**
- P8: Hm, kind of, kind of correct. And and if you look at, I mean a slightly off topic. But if you look at like all the like topics on change, on motivation, you know. They always say that, like intrinsic factors are always more effective than extrinsic factors, you know, like, yeah, you can. You can give incentives and all that to motivate someone to do. But then but then, if if they don't internalise it and make it intrinsic, they will give up. You know. So I think I'm lucky that like like that, I I have certain intrinsic factors in me that want to do certain things, anyway lah. Yeah. Yeah.
- **NJCL: Hmm. And then, moving away from facilitators and motivators. How about like barriers in you self regulating your lifestyle?**
- P8: Which I think I did kind of touch on also, like like, let's say, see my time, my schedule, distance, costs, you know they were, they were primarily the reasons why I stopped going for my martial arts, you know. Cause of my change in schedule, then my workplace is further away, you know. So so these are all barriers so like I I did mention. Let's say, somehow I I managed to move house to somewhere near to my workplace. I may pick it back up again. Yeah. So that's reducing the barrier already ah, right?
- **NJCL: Yep**
- P8: What else.. Uh. Yeah lorh. Oh, I think that. Oh, and then, like I I did mention about how I don't cook also. So if if I can buy food, vegetables and all that is easily available, you know, from outside, that's really cool, then then I would definitely consume more. But at at a current state like, food that I buy outside somehow largely don't include vegetables, so I think, because vegetables are harder to keep. So the vendors don't really carry. Aah [swear] one lah, that one I don’t know. [NJCL: Participant shakes hand, smiles] I don't know how they yeah. So so so so I get, that is a barrier also lorh, availability, you know, if it's available, I’ll buy it, then it’s no longer a barrier already ah.
- **NJCL: Can can.**
- P8: [Mumbles inaudible]
- **NJCL: Just to clarify. The barriers are- for you are the time and schedule. Firstly like- Like you mentioned, your schedule is a bit different now already.**
- P8: Yep
- **NJCL: Like, 3 day off every 2 weeks**
- P8: Yep
- **NJCL: Then it's also the distance that you transfer to a further place from your workplace.**
- P8: Yep
- **NJCL: That's also a factor.**
- P8: Yeah.
- **NJCL: Then there's like the cause of like maintaining a healthier lifestyle in terms of like-**
- P8: Yep
- **NJCL: Paying for this. And lastly, the availability or lack of availability of like healthier options.**
- P8: Yep yep. [NJCL: Participant nods]
- **NJCL: Okay, can. Thank you for that. Then, okay, then I'll move to my next question. It's how does your self regulation, your personal self regulation habits, impact your work performance?**
- P8: Uh… Does it impact my work? [NJCL: Participant looks off camera, thinking out loud] Hm. I I don't know. I guess I guess. Like, maybe because I am more motivated to to do things, so I'm a bit more ambitious like, I I I I really try to. I want to climb the ladder, but I don't know if you know.. Nursing ah, climb very slow one.
- **NJCL: Oh..**
- P8: There's a prescribe, there’s a prescribe like, oh. You have to be 3 years in this rank. You have to be 5 years in this rank. So right. So like, like, currently in my workplace right, where I get to see a lot of rich people ah, because private mah, you see uh what, 30 something year old director of a company, you see. I saw- I met a 30 year old professor. Yeah yeah, you’re you’re doing- You’re Year 4 academic now right, can see 30 year old professor, you know how young that is or not? [NJCL: Participant smiles] As like, a new professor
- **NJCL: I see…**
- P8: Yeah, then then, so so, you think to yourself- But nursing cannot. No matter how fast you come out. First class honors, they will still say you need at least 2 years as an SN before you can become SSN, 3 to 5 years before you become SSN 2, SSN 1. You no way you can- Some more, are you local?
- **NJCL: Yep, I am Singaporean-**
- P8: [Inaudible] By the time you graduate, you're like what? 24? No way you can reach what, director level at or assistant director level at Year 1. No way. Yeah. So so so I think, like I I try very hard to to to push my my my career. But, there's barriers lah, in our particular occupation. Yeah. So like, like, for example, I went and own self pay. And did my masters, and all that. Cause, I think if you wait for the company to sponsor you ah, then you're gonna wait until donkey years already. Then plus if you, if you, if you wait for sponsorship, then then there is a bond. Then you know, you have less freedom to choose a job, and all that. So so I think if you look at my my personal motivators right, where I want to push myself to do more things and all that. Then then, yeah lorh, I I did. I did push myself to get a masters. I did push myself to do more, to pick up more projects and and all that to, because I I want to to go further in the career. So that's a motivator plus I mentioned again. I I value time spent in something. So actually, I am not one who drop jobs, see. This is, I told you this is like my first time change job right? I was in my previous organisation for actually 8 years. Then, after that, after that 8 years, I I feel like it's very difficult for for me to get promoted, even though I'm trying very, very highly. That's why I choose to jump, even though I would have stay on because I I treasure that aspect lah, you know, staying in something for a very long time. Yeah, yeah. So that's my interesting motivator as well. Yeah.
- **NJCL: I see.**
- P8: Yeah
- **NJCL: So is it right for me to say, because of your own like personal-**
- P8: Drive
- **NJCL: Vision level, yeah, personal drive. Yes, that’s the word. This, this drives you to actually try and push yourself to go to to go higher in career progression.**
- P8: Uh, yeah, that's what I'm trying to achieve lah. But then we are facing with facing with actual. You know, limitations in the in the job, where there is no way I can go any faster. You know. There'll be like “Ah you're not an SN 2, for- You’re not an SSN, , for 3 years yet, I cannot promote you”. It's a hard stop eh. There's no “Oh, we may consider on a case by case.” This is, no, it's hard stop, not 3 years means cannot. Yeah. yeah, that's what we are facing our in our job. Yeah.
- **NJCL: Okay, so the my question earlier was somewhat like some regulation habits impacting work performance. How about like- how does- how do you think your own self-regulation affect your life?**
- P8: I think it's okay, leh. Quite positively leh, like okay lah, like, up till I up till I stop exercise up till I stop all the martial arts. It helped me stay relatively fit, I guess. I mean, I did get injured also juju jitsu. I tear my ACL. Yeah. So I guess there's a downside to lah. [NJCL: Participant laughs] Oh, impact my life yeah lorh, tear my ACL. Yeah, yeah, but otherwise I think it's a net positive lah. Like like I mentioned physical wise. I kept healthy for quite a number of years until recently. Mentally wise motivation wise, you know. You help me keep my passion for health care, you know, despite the the kind of patients we face in public health care help me feel appreciated, help me have a secondary tertiary identity out- that is purely not- Not purely based on nursing. Still health care. But it's not purely based on nursing. Yeah, yeah.
- **NJCL: Okay. And just asking, like, out of patron. Are there any like tools or apps that you personally use to like? Self-regulate your lifestyle?**
- P8: Hm, no. [NJCL: Participant squints] You mean, like some kind of app, to tell you when to exercise, that kind of thing?
- NJCL: Yeah. That kind of thing
- P8: Count the calories or what.
- NJCL: Yeah, yeah.
- P8: Don't have, I think, healthy, healthy 360 tried to roll that out before, counting of calories ah. I used it for a while. Uh, it was too tedious for me. Because, okay, it’s that, it's just helping me track. It's not caught- is not making me change my habits so then then, it becomes I’m just logging in what I’m eating lorh. Yeah, yeah. Yeah. So so, No lah, I'm currently not using any apps to to make me exercise more or make me eat healthier. No lah. Yeah.
- **NJCL: Not only apps like any kind of tools, maybe like at home. You have your own weights?**
- P8: No, no, no, my my arms. [NJCL: Participant points camera to show arms] Does it look look like lift weight arms to you? [NJCL: Participant laughs]
- **NJCL: Haha, still still fit. Okay. Then. Okay, then, for the next few set of questions is regarding the workplace. So as a nurse, how do you think the workplace influences your own lifestyle habits?**
- P8: Uhhuh, I think I think kinda throughout our own conversation is kind of already. Peppered- peppered through already, yeah. So so like, I said, the amount of rest days you have, your work hours will definitely affect. What kind of activities you can. You can accommodate lah, you know, like, when I was in public health care, where the work hours was slightly less, and there were 2 off days. I can accommodate 3 martial arts, you see. Plus all my volunteering activities. So so I didn't start my volunteering activities after I stop my martial arts ah. They were all simultaneously ongoing ah. I mean, if not, how? How would I have done my health cert for 10 years, right? Yeah, yeah. So, so, so, so, being in public healthcare just has it’s disadvantages ah, the work hours, the office, and all that. Got to accommodate so much things. Then coming out to private. You know, private lah, they are profit based, so they’re squeezing a bit more of the workers ah. So so I had to work more hours and all that. So so I can only accommodate less activities lor. Yeah. So so that's how your work affects lah. And plus, I think I also mentioned about how- no matter how driven you are, how motivated you are at work. There's a hard barrier to how fast you can climb your in your career, which I think can be a demotivator ah. Which is why I left my old organization ah. Yeah, because because they they they are not promoting me. So III jump to private where they are willing to give me a a slight promotion. Yeah, yeah. So so that's how your work also limit limits limits you lor. And plus plus, you see in our liner. Like I mentioned it before, the the patients, you know, you know, no matter how passionate you are about nursing, you know, you meet all the patients you meet them one week, one month, one year, two year. Sure will make you sian one, right. And yeah, yeah. So I think that that's how I see how work affects affects my motivation lor. Yeah.
- **NJCL: Could you elaborate more on how? How the patients, how the type of patients affect you affect you and your lifestyle habits.**
- P8: Uhm. So so right now, right now, how? How I'm linking is that like like like, you can be very passionate about your work right then. Then it it leads to like positive mindset lah, you know, like like being multi- motivated, happy, and all that. Then if you encounter negative negativity at work, don’t say just patients lah, if colleague negative, negative supervisors. Then then you get more and more stressed when going to work. You know, like like going to work becomes a negative event for you, then, I I have encountered people who take MCs because they’re too stressed at work. You know, it becomes like psychosomatic stress. Then they get migraines, stomach aches. Yeah, yeah. Fevers even so. So then, after that, you know depression, even PTSD, I. I've encountered not not nurse lah, but I encountered. Uh. Patients who I think they work with. They work with some shipping company or what, then during the tsunami. They are. They are activated to to help with like clearing the dead bodies during the tsunami. Then they got PTSD depression. PTSD, yeah, yeah. Yeah. So so I mean that, that's how I see how work affects your health lor, your motivations and all that. Yeah.
- **NJCL: Just now you were mentioning something about like stress in general, could you elaborate more on the stress?**
- P8: Like stress, stresses at work ah?
- **NJCL: Mhm!**
- P8: Uh. Can be.. Can be tight deadlines. you know, like they give you a task to do, and then you only have like 2 days to complete. Then can be uh.. Un- Unclear expectations? Like you expected to to do- You've been told to do something. Then, then, depending on how how nurturing your boss is ah. Sometimes they throw the task to you. Never tell you what to do. Then you do wrong, then you kena scold. That stress also I think, depends on how how patient your boss is. I have had. I have had uh, are also reporting officers who are very perfectionist, and also very strict, very fierce. So ah, really scrutinize your work, then really scold you for everything, then yeah lor. So then, when submitting a document ah, then you feel stress in a sense like, “Ah shi- kena scold one. I do also wrong. Don't do also wrong”, you know. Yeah, those are stress orh, yeah. I think there are those that question your decision-making- So like. Especially like, if you start if you do like, go up to the supervisory role one day, I think. I think, that it's something that that that is. That that should be explored. In a sense that through our nursing careers, right? We as ground. We. We are trained as ground nurses for all your nursing career. Throughout your nursing school, your trainers ground nurses, and actually the skills that you need the soft skills, people skills, even hard skills that you need as a manager, as a supervisor is vastly different from what you use as a ground nurse, you know. How you how you counsel people, how you how you manage people's expectations. All the paperwork, budgeting, EQ ah. Interacting with people from other departments and all that. I think it's it's something that is very foreign to most ground nurses. And then, I think, without the proper guidance. It's a very stressful transition, plus. There's no, there's no, there's no lesson class course textbook nothing eh. It's purely based on what you pick up along the way. I guess, as you grow more and more senior in your job, you start picking up more and more roles lah, you know, [inaudible] making, CI, and all that. So you start picking up all these small small roles which which by right should slowly accumize, acclimatise you to the full advisory role lah. But but ultimately, when you, when you reach the top, the the supervisory role already, is still very, very different, very, very different from from being a ground nurse orh, yeah, so I think I think without the proper guidance. And it's largely on the job training. You. It's it's a very stressful. It's very stressful lah. Yeah, I think so. Yeah.
- **NJCL: The the transition from ground nurse to supervisor-**
- P8: Yeah yeah, correct correct. So basically ah, in school, you have your what, transition in between student to staff nurse.Then actually, I feel ah, there’s also ah, a jump lah. The transition from ground nurse to supervisory role. Yeah, and it's something that is not explored often, I think? Yeah.
- **NJCL: Yeah. Interesting**
- P8: Yeah.
- **NJCL: Okay. And wait ah. Okay. So for- Just to summarize what you've been mentioning, the workplace influences lifestyle habits, you mentioned that it's because of the the day offs, like the timing is all different. Then also, there's, there's a hard limit. How much you can climb which can like, demotivate you. Then there's also, like various stressors, like patients, colleagues, bosses and having tight deadlines, and lastly, also mentioned, like the transition from nurse to Supervisor. It's also quite stressful. Is there anything else worth adding here?**
- P8: Yeah, more or less, what we- What I say. Yeah.
- **NJCL: Okay. Then it's- My next question is something related. I I think you touch upon it a bit. But how do you feel about the workplace culture among nurses in helping to facilitate hinder self-regulation of healthy lifestyles?**
- P8: Uh. I didn't see your group of friends. Now see, see, I think as with any anywhere as with, as with any social group and all that it really cause. There are all kinds of people in the world. So you know, you got your aunties. You got your your youngsters would like to party and all that. I I think I think each ward is just a microcosm of of what you, who you will see in society, anyway. So it depends on who you hang out with lorh. So I think there was a period of time when I was in a ward where there were a lot of relatively young drinkers. Not, not, go out and having drink ah, it’s go to each other's house ah, then drink, that kind of drinkers. Yeah, yeah, yeah. So so so we we just if all, we all seem off day, they will hang out in someone's place. Drink, play game, you know. [Inaudible] That kind of thing. Yeah. So but then I also do know that like in my current workplace, I know that there are some colleagues who who do a cycling like round the country round the country, cycling kind of situation. So so if you ask me, how does your workplace or colleagues affect your health healthy lifestyle, I would say, it depends who you hang out with lorh. Yeah, then, who you hang out with also depends on your interest lorh, like, let's say for me right with my [inaudible], and then and then you know, I don't really go out spend, all that even if even if I I know that there's a cycling group I also won't join. Yeah. So it really depends. Back back to intrinsicity, if you're interested in cycling, then you will seek out fellow colleagues who cycle. Then you will. You'll be a positive, perpetrating cycle, you know. Everyone will go and cycle together. Then, if you don't cycle, you're a drinker, and you will seek out friends, colleagues who are a drinker, go for a drink. Then you'll be a negative, perpetuating cycle eh. “Oh, you just always drink on your off days lorh”. Yeah, yeah, that's how I see it lah.
- **NJCL: Ah.**
- P8: That’s what I think ah
- **NJCL: Okay, then, is it right for me to say, you personally like the workplace culture? It can be both a facilitator and a hindrance to your own self-regulation of a healthy lifestyle.**
- P8: Yeah, it's true. Yes, I agree. Yeah, I I think I think it goes back to your interest thing lorh like who you gravitate towards then who- What activities you will do lorh, right, I cannot force the drinking guy to go cycling ah. [NJCL: Participant smiles] Yeah.
- **NJCL: Hmm. Oh. So how- How do- How- What do you think of the idea of like peer pressure?**
- P8: Peer pressure.. But then. Oh at work orh, like. Then then you will have to somehow have a disproportionate amount of, let's say, like your ward of thirty staff ah, thirty very small ah but then hypothetically speaking, your ward of thirty staff and you need what? Half to 3 quarter of them are health nuts eh, fitness nuts. For some reason at all, or 3 of them. 3 of them like to do tennis, five of them go cycling, another 5 go spin class or something. Then I mean you need to have a majority before peer pressure will work on the minority of unhealthy people right? But then, like I mentioned just now, each ward is like a microcosm of society. You're not gonna get, wha, 3 quarter of healthy people, anyway, cannot be one. Yeah. So so so I think your peer pressure wouldn't work, because let’s say, if I would judge when I hang out with the cycling guy there, I don't hang out with the cycling guy off. I hang out with the drinking guy orh, [NJCL: Participant laughs] you know.
- **NJCL: Okay**
- P8: Yeah.
- **NJCL: Hmm. Okay, then, my next question is. I I feel like it might be [inaudible] on this. But it's basically, what do you think about the role of nurse managers in helping nurses maintain their own health? I mean moving your in a supervisory position now**
- P8: I think it's difficult lah. Like. How to say that. Okay, so in my old workplace right, uh. My, my, my supervisors, did frequently asked for exercise- Team building, they call it. They call it team building. They. They put it like for example, every Friday, so no matter what, cause we no shift ma, so cannot be everyone be free. So every Friday, no matter whoever is free. We'll go to this basketball court, and then do exercise together. That is, that is a very positive way of thinking about it. But then it will die down, because eventually people will say, “Why am I using my off day to do a work related activity?” They are viewing it as well related, because it's mandated by the boss mah? Right? So I think, even if you like you, you stroll online and order- some people also like Reddit, and all that, people are also asking questions not not just interesting, even in the corporate world, whether they're asking drinks after work, you know. “Do I have to attend?” “Is it compulsory?” You know then people advice you, “No ah! It’s your work hours, is your own free time. You're not obliged to attend,” other people will say, “Oh, you attend! Because got networking, you know! Show your office. Your boss knows who you are,” so as a Boss ah. If you mandate after work hours exercise, people will say “Why you eat into my rest time? Not fair,” some some more, some more in my current place, people got less, less rest time. You know. Yeah, or like, Oh, you make me come now, are you giving me back an off? You encounter staff, they tell you this kind of thing. Are you giving me back an off? Is this paid time?, you know, so is difficult. You can facilitate lah. You can be like “Ay, this Saturday. Let's all go and do this,” but then I think, in the end your participation rate ah. Who will go ah, will be your, just now I mentioned your cycling, your cycling gang ah. Yeah, the non biking gang maybe maybe will turn up once, not twice, just to show face, you know, show boss “Ay, I I take part by your initiative ah.” But if it's like, every week, I’m not gonna bet it's gonna happen every week ah. Yeah. yeah.
- **NJCL: You mentioned something about facilitating like, How- how do you think you can facilitate? Could you elaborate on that?**
- P8: Uh. So sometimes, when people don't do the activity. Let's say, let's be. Let's say if it's a basketball or cycling sometimes is because they they don't want to plan ah. You know, they don't want to book the basketball court. They don't want to figure out where to get to buy cycling trail and all that. So I think facilitate in this sense is that like. If you make the system easy to be access. Then, people just if they can just just join, then it will help them. You're reducing the barrier lah, you know, in a sense, like let's say. Okay. I already chope this basketball court every Friday. You all just have to come down. Don’t even need to bring your own ball. No need to even bring your own water. You know. Just come in t-shirt and shoes and shorts, and you can play. Then then they will come down lah. More likely is they will come down. Then you tell them, “Ay, you go book a basketball court ah and you bring your own ball ah”. Then people don't want come down already lah. Then what if the ball guy is on duty eh? [NJCL: Participant laughs] Yeah, “no ball today sorry”. Yeah. Cause I don't think every single time is gonna buy a ball what. Then what? Then, you have to plan a schedule thing just to bring the ball down meh? Right? Yeah. So I think the same goes to cycling. For example, the the, the my staff that do the cycling group. They own their own bikes. Some of them own very expensive bikes. See that as a barrier to entry. Let's say I have a new staff now, and I'm joining by cycling eh, oh must cycle and buy a expensive bike. What if, I try one time then I don't want to cycle leh? You know, so that that is a barrier to entry. So then, as a facilitator, what I can do is, oh, I got a spare bike at home. Or, or, yeah yeah. We'll be at this rental place where you can just rent the bike, you know, and go. Even better! I sponsor you to rent- I sponsor you for the bike renter. Then then they just have to turn up only ah. Then that’s facilitating already lorh. Yeah.
- **NJCL: Ah-**
- P8: So I think- For example right, there’s going to be an upcoming bowling competition, I think SNA. So, my, my chief nurse right, encourage people to join, she want to facilitate right. She say she sponsor. Any team that join, she sponsor. The uh, the entrance fee lah, for the competition. Ah, then that’s facilitating already lorh. Yeah, cause you know, people will be like, especially if your EN’s or what, their pay are not high ah, “Hey, sister, expensive eh. Join this competition, then I don’t know how to bowl, not strong, then waste money.” Wash the gutter, then pay 70 bucks, then then then there's a barrier already mah? Right? So so I think that's that's in this sense how how I view the work facilitating lah, you know, reducing the barrier, ,making as carefree, as easy as possible for them to join activity. Then they will join lorh.
- **NJCL: Can, that is- I see. Okay, then, so just to summarise for you as a supervisor. You think that the role of like manager or supervisors in helping like nurses, ground nurses maintain their own health is a bit difficult, but there are at at least you can you can facilitate**
- P8: Mmhm.
- **NJCL: Is there anything else you want to add here?**
- P8: No lah, is like that. [NJCL: Participant scratching behind ear]
- **NJCL: Okay, then, moving on, almost at the end. But how do you think your institution can help you maintain a healthier lifestyle?**
- P8: How ah? So. Back to the [Public Hospital Name] example, I don't know if you know, but they have a fitness challenge every year. You know that? [NJCL: Participant smiles] Like, IPBBT, uh.
- **NJCL: I I think I heard about it?**
- P8: Heard about it? Yeah. So so [Public Hospital Name] right. It's part of the appraisal every year. Every year's appraisal ah, they will say, but then you, they will say whether you attend the the health screening and whether you attended the fitness challenge. Okay lah at 10 ah, they never say you have to pass, have to get gold. Nothing lah, just attended.
- **NJCL: Okay.**
- P8: Yeah, yeah. At the very least, there’s that lah. Yeah. So so I think in one way. That's how they incentivize people to stay fit lorh, by making it compulsory or part of their appraisal. Yeah, yeah. Then, like I know that while I was at [Public Hospital Name]. They had a lot of interest groups, you know. I think weekly Zumba all that. So so those those are are ways where they they can help. As an institution, they can help make people more.. Like I say ah, facilitating lorh, or reduce the barrier lorh. Or, you know, maybe like this guy. Actually, I'd be interested in exercising, but don't know where to find or expensive, you know. Then if it's a free Zumba organised by the hospital, then people will join already lah. Yeah. But then that's- I think that's also a barrier lah. Zumba people think it a female thing ah. Guys won’t join ah. [NJCL: Participant smiles, laughing shortly] Most of the time. Yeah. But but it's general ah. I think that's also other interest group, soccer interest group. Then then then, that's how an institution can help to uh, encourage. This kind of a healthy lifestyle ah. Yeah.
- **NJCL: Ah, then specifically for your current institution?**
- P8: My current institution? Like I mentioned, it's profit driven. So there is not that much incentive cause anything that you spend money means is less profit mah right? So that's not that much. But but then recently, my my chief nurse did- Because, I think happened so happened that one of the, one of the new nurses that join my team, my my hospital, she has experience in teaching Zumba. So so recently, they did start a Zumba class that that is run by the new nurse. Yeah. I think I think, what what's what's better. Is that because the new nurse is working office hour so so she can actually have a set calendar to say every Friday is Zumba day. You know, but if they’re a shift worker. Then, hah, you cannot set every day as Zumba day ah, unless you specifically request in your roster. Yeah, yeah, yeah. Yeah. So so my current organisation at the moment, is just this, this Zumba class lah. [NJCL: Participant smiles] Yeah.
- **NJCL: Then. Your current institution, is there like any other- like workplace health promotion program other than the Zumba?**
- P8: Hm. [NJCL: Participant resting chin on hand] There's a free health screening lorh. And then they offer offer you all the flu, jabs, and all that lorh. I mean, you can count those as help, I guess. But otherwise uh, no, I don't think I encountered anything that is facilitating me to exercise. So yeah.
- **NJCL: Okay, alright. Then moving on to my final question. Outside of work, what do you think influences your lifestyle?**
- P8: Huh. [NJCL: Participant smiles, gesturing with hand] I think it's what I’ve been saying over and over. Uh, uh. For me and I it's mostly intrinsic lorh, you know. And then I think you cannot. Just ‘whack’ ah, everybody go running, everybody goes swimming. I think if you are doing a sport that you are interested in, then you're more likely to stick to it, you know, and do it lah. Like, for example, for me, I I really like the martial arts techniques. So the the exercise part is the side effect of it ah, cause I like the techniques ah. So like, for example, if you force me to run, that's not gonna get, that's not gonna get me anywhere. Then, like, for example, my, my mom likes running so so she run around, run. She can run Marathon eh, at her age, she can run marathon. She can do hiking trail trail trail, don't know, hiking or something. Then she also does dancing, you know, ballroom dancing, Latin dancing, and all that. So I think to make people exercise. You need to find out. What is it they like to do in the first place. Ah. If there's a mismatch ah, if they don't like to run. You make them run. They don't like to ping pong ah. You make them ping pong. It's not gonna stick orh. Yeah. Yeah.
- **NJCL: And then so to summarize what you shared outside of work, your influences- Your lifestyle are mostly just intrinsic factors.**
- P8: Yeah
- **NJCL: And like, interest.**
- P8: Yeah.
- **NJCL: Is there? Is there anything else you want here?**
- P8: Anything else ah? I guess if you're a current intrinsic.. Having a friend [NJCL: Participant squints] Like, “Hey, let's go do this together,” can help to a certain extent lah like like. When I was doing my NS, I did pick up Karate for a short while, because one of my camp mates is like, Hey- Doing- Cause my my NS time, I was doing a regimental policeman. So it's a guard duty. Just guard duty all the way. My whole NS life is guard duty. So so my, my, my fellow and NS NSF. Was like, “Hey let's go and do something”. Then I went along with him and picked up Karate for a few years ah. I I I picked up karate during NS right, I did it throughout my NUS time. I was the NUS karate kid.
- **NJCL: [Chuckles]**
- P8: Until I stop- until I graduate from NUS. Then I stop doing karate. So so so let's see, just follow friend ah, anyhow. Anyhow, also did it for about 5 years lah. 5 , 6 years ah. Yeah. [NJCL: Participant laughs] Yeah. So so I think if you want to count something that's intrinsic ah, having company lah, having a friend might overcome a bit of your lack of interest. So let’s say. I'm ambivalent about cycling. Maybe I don't hate it, but I also don't like it. Then you have a friend that say, “Hey, let's go cycling together eh”. It might. It might motivate me to do the activity. Yeah. So that’s what I handle orh.
- **NJCL: Okay, okay, so other than intrinsic, or extrinsic factor, it's having company. These kind of things. Okay.**
- P8: Yep, yep
- **NJCL: Can. And is there anything else?**
- P8: Nope, that’s good. [NJCL: Participant holds a thumbs up]
- **NJCL: Okay. Then you have anything else that you would like ask, or speak about them, mentioned in the interview. Any further comments want to share?**
- P8: Uh, I don't think so. [NJCL: Participant shakes head and smiles, laughs]
- **NJCL: Okay. Then this marks the end of the interview.**
- P8: Okay
- **NJCL: Thank you for your time and participation.**
- P8: No problem. [NJCL: Participant holds a thumbs up, laughing]
- **NJCL: I'll stop the recording.**
- -End of transcript-

# **Interview #9**

Participant and interview details
 Participant number: 9 (P9)
 Date of interview: 27 Sept 2023
 Interviewer Number: Navarro John Christopher Lambino (NJCL)
 Time IDI started: 2130
 Time IDI ended: 2240
 Observational notes: Participant seems comfortable at her home.

Section I: Interviewer’s summary
 The individual interview was conducted via Zoom. Participant was relaxed at the comforts of her home. Another private hospital nurse, she is the 2^nd^ participant from private hospitals that was interviewed so far. Unlike P8, another private hospital nurse, P9 has always been working in private hospital since student nurse days and thus have no frame of reference for outside her institution. Compared to P8, P9 can almost be described as sedentary. Outside of work, her hobbies include dance. She mentioned how she was overwhelmed, and had to sacrifice her life outside of work, and only now after gaining some stability and confidence she is slowly going out of her way to maintain a hobby. Interestingly, for P9, she is the first participant to not believe in ‘work-life balance’ as she mentions that work is part of life, and only after removing this demarcation, did she feel more at ease. However, she also mentioned that she enjoyed her work, and she had passion so her view may not be congruent with the rest of the nurses that she know. She is also cited to be almost completely satisfied with her current lifestyle hence, there is no impetus to change. To her, nursing managers role is mostly to help nurses on the ground and ensure they have time to go for breaks. They also help facilitate hobbies to continue outside of work

Section II: Transcript

- **NJCL: Okay. Okay, good morning- Good evening, miss. Firstly, I would like to thank you for taking the time to speak with me today. My name is Chris, and I'm a NUS Year 4 Nursing student. The aim of this interview is to get a better understanding on the perspectives nurses have on self regulating their own health behaviours, and how various factors may influence this. I want to find out what are your views, opinions, and experiences of this. I would really like to encourage you to speak what comes to mind. There are no right or wrong answers. May I have your permission to continue video recording the session?**
- P9: Hm. Yes, okay
- **NJCL: Okay, thank you, then, can we start with some basic questions like. Which ward are you working in right now?**
- P9: I'm actually working in the A&E, yeah, in a private hospital. [NJCL: Participant clears throat]
- **NJCL: Can you tell me more about like, the type of work you do?**
- P9: Uh. So basically, uh, yeah, in the A&E, we have like the priority, like 1 to 3. Yeah. So how the A&E in our hospital is uh, formatted, basically the critical care area, which is the P1, P2 area. And then we have the walk ins or the cases that are less urgent, which is in the P3 area. Yeah. So uh, for now most of the roles that I do. Uh, yeah. So usually, now, I do a lot of I usually am in charge of like the, [NJCL: Participant clears throat] like the different areas. So, depending on my assignment for the day, or sometimes I'll be in charge for, like the P1 area. Then I just technically have to oversee all the patients that come in. And then, yeah, basically just sort of organise the flow of everything. Whereas, for the P3 area, [NJCL: Participant clears throat] for private hospital is a bit more different. So the kind of work that we do uh. For P3, what we call P3 in charge lah. So technically, P3 in charge really oversees the entire A&E for the day and uh, so there is a lot of like sort of manpower movement, or even like, we do things like hotel or house calls and coordinating the doctors, all that sort of stuff. Yeah. So for P3 in charge, it's more and administratively heavy. Yeah, so that's that's it. Hmm.
- **NJCL: Can you describe the workload that you experience?**
- P9: The workload is it?
- **NJCL: Yup**
- P9: Hmm. So I would say that. Uh, because I guess, for the A&E, things are very unpredictable, so you don't know when a patient will come in. So there are times when, things are actually like slow and manageable usually in the morning for my hospital, because we also do take in SCDF cases like just like a government hospital. So hmm, I would say that. Yeah. In the- in usually like in the afternoon before noon, that sort of timing is when all the ambulance that come in. Yeah, then it can get very busy. Yeah. But I would say that. I sort of like the setting there, because I get a good balance of uh I rest, and, like, you know, having a lot of work to do because there are times when is quiet, and that really is like the time for you to, you know. Just chill. Yeah. But when, of course, when work comes in, then just, you just have to do it.
- **NJCL: I see.**
- P9: Yeah.
- **NJCL: Uh, does the primary hospital have, like phone calls? Or like, they call back nurses, to do that kind of thing?**
- P9: So for I guess different host, different institutions. They they practise like different things so within my hospital, I've done. I've also worked in the wards before. Before I eventually settled in A&E so. The culture of the ward is that yes, they tend to sort of call the nurses back when they’re on their rest days, on their public holidays, even on the ALs like when they really need them, they'll call. Rest for the AnE. The culture is a bit different. Which is what I like, is that on the assigned rest day, they do- They would never, ever disturb you. You know it's like, regardless of how much how much short of manpower and how much you're struggling, or you know how many patients there are. You really just, you know, like the people who are working on that day, you just gotta push through it. Yeah. And if you really need help, sometimes the the management side, you know, the managers sometimes will come down to the to the ground to help out. Yeah. So that's the thing that I like ah, is that when I say it's my rest day, I just get rest. Yeah.. [NJCL: Participant smiles]
- **NJCL: And then going back to the work would. So is it right for me to say the workload, it depends mostly on the time of day?**
- P9: Hmm. I would say that. Yes, it depends on the day and uh, and the assignment for that day. So like, I said earlier, nowadays I tend to do a lot of in charge. So as in charge. Then you sort of need to make sure that you know everything goes well. So you have to cover a lot of ground, then that's like, naturally, you get a lot more workload than the others. Yeah. So whereas, if you know sometimes, if I'm lucky, then I can get some easier assignments. Then I just get more relaxed day ah. [NJCL: Participant smiles]
- **NJCL: I see. Okay. Can, thank you so much for that. Then my next few series of questions is regarding the lifestyle in general. So as a nurse, how do you define a healthy lifestyle?**
- P9: Hmm. [NJCL: Participant smiles] I guess. Hm. I think specifically for nursing, what I find would be considered unhealthy right, is sort of like not being able to compartmentalise your work and your personal life. For example, when you're no longer on shift right, and your mind constantly things about your patients and work, then I would say that is not very optimal. And I think a lot of uh, like for me also. And my friends, when we first started out, and there's a thing that we sort of like, struggle with. Because when in your mind, you know, it's just like thinking about work all the time. Then, you know, like at night you can't even sleep properly, cause you'll be thinking like, Oh, yeah, you know I I have all these patients tomorrow morning. That's my assignment tomorrow morning. Then I have to. You know. What should I be doing? Then you're planning about work tomorrow when you're trying to sleep. Yeah. So that is. That is one unhealthy thing I would say. But for the rest, okay, III think there isn’t a.. I think the lifestyle is okay. Yeah, I I cannot really specify anything else that might be considered unhealthy. Yeah. Hmm. [NJCL: Participant sniffs]
- **NJCL: Earlier, you mentioned something about when you first started. Keep thinking about well, having trouble sleeping that kind of thing. Then how did your lifestyle actually change after you started to work as a nurse?**
- P9: Hmm. I would guess. Hmm. First of all, irregular sleeping hours. Cause of the shift work. Yeah. So like over the years I've gotten used to it. And I I sort of accepted it. I'm okay with it now. But yes, at the beginning it can get very difficult, especially uh, like the transition from night shift to day shift, that kind of thing. Yeah, cause we do night shifts like, per round it's about 3 to 4 nights every 2 weeks. So uh yeah, the body just has to like, self adjust. And sometimes it just cannot adjust in time. Then you have to go back for like to do shift again. Yeah, so that itself kind of sort of, I guess the mental capacity cause sometimes errors tend to occur when when this sort of stuff where the person is just too tired, and like might have not enough rest. Yeah. Hmm. And also personally for me when I started nursing, then, again because of the shift work. I sort of have to give up certain aspects of my personal life, so like in the past I used to dance a lot right. But oh. ever since I started nursing, like having to do shifts, and like not knowing, especially like taking leaves and whatnot right these always have to be planned like far ahead in advance, and it has to get approved. So in the past I used to do like dance shows and performances and whatnot. So I just like, completely stopped because it's just not possible to have like, for example, like maybe I, every Tuesday and Thursday, I should be having practice at night right? But having shift work, I I just cannot promise that I'll be available on those days for practice. Right? Yeah. So those are the things I I eventually gave up on, and I eventually accepted that, like in the past, like I I couldn't really accept it. Then I just got very upset with it. Yeah. But now, now, it's okay for me. Yeah.
- **NJCL: Uh, can you elaborate on what you mean by get really upset with like, not being able to accept?**
- P9: So, I would say that at that time I considered it as a sacrifice, so I had to sacrifice my interest and my my hobbies for nursing. Yeah. So and like, before I started work right. [NJCL: Participant clears throat] Most of my social circle, like my friends we all dance right. [NJCL: Participant smiles] So now that I am no longer like doing that, then I lost that part of like the social circle for me as well. Yeah. So I I I no longer maintain those like friendships and relationships, though, yeah. [NJCL: Participant smile fades, rests chin on hands]
- **NJCL: Then, talking about like physical activity wise, how do you feel about your current lifestyle?**
- P9: Okay. So actually, when I was doing the questionnaire right. Then there was some questions about like physical activities and exercise. [NJCL: Participant smiles] So okay, current. So very recently, I I started to sort of incorporate so I guess I consider dancing as like a physical activity. Yeah. So just few months ago, I started to incorporate it back in into my life again, cause I feel like. Finally, like, after a few years at this point, I'm starting like I sort of like am used to work, and I feel like I've settled down at work properly for me to venture into like my personal life and my other hobbies. Yeah, uhm, so in the past. So actually till now, I I still do that is that because in the A and E, we are always running around right. And so, my hospital, we do that we don't have helpers, so, like the nurses, we push their own trolleys. We admit the patients. We send the patients to scan. And we do a loan. So like one person push one trolley. Yeah. So uh yeah. So I just consider that as my physical exercise. [NJCL: Participant laughs]
- **NJCL: I see**
- P9: Yeah, yeah. So I I really do like, like, I move around a lot. And yeah, so that was what I I used to compensate for my lack of exercise lah. But I've also spoken to my other co-workers. And yeah, they they. They know that it's not like proper exercise, like they try to encourage me to go and run, or something. Sometimes I’m just too tired. [NJCL: Participant laughs] So yeah, so for now I just do dance once a week. But uh, my approach is not for like, healthy living or what ah, I it's just. It's just a hobby. Hmm.
- **NJCL: Uh, when you say it's just a hobby, it's it's mostly out of your interest. Is it right for me to say?**
- P9: Yes, correct. [NJCL: Participant nods head]
- **NJCL: Okay. [inaudible] got you back into dance?**
- P9: I would say that I've never lost interest. But uh, so from like where I started work till Covid. And now that finally Covid has, you know. Sort of, sort of phased. Then I just feel that like I am at this point of my life, that I feel I'm I'm satisfied with my own work, and I think that I can cope and juggle, you know. Just other things out of work yeah. Oh.
- **NJCL: Okay. Then, moving to the next topic on like, diet. So like, how do you feel about your current dietary habits?**
- P9: Uh, I guess eat whatever I want. [NJCL: Participant laughs] Actually, yeah. Yeah. So uh like, yeah. So like, back to the questionnaire I did right. So I actually, I had some like hesitation about answering, because I wasn't sure if the question was like, for example like, do you? Do you eat food? Or that's something in low cholesterol or low sugar something like that, right? And uh, I don't like, the food I eat, I don't intentionally think about like, oh, this is low in cholesterol. So I eat it. Then I will pick this food because it's low in cholesterol, but it's more of like my diet naturally gets- sort of get to like, I like to eat veggies, so it just like, I will just eat veggies, but not because it's healthy, it’s just because I like it. [NJCL: Participant smiles] Yeah. Yeah. But I would say that. So I lost a lot of weight since I started working. Yeah for additional info. Yeah. When I started working, I before I started, I was 48 kg, then the lowest I got was 36 kg, which was last year. Yeah, during Covid, [NJCL: Participant laughs]
- **NJCL: Oh! Wow!**
- P9: Yeah, it was like it was uh, it was like, maybe the second second- third wave of Covid already that time. Yeah, it's just horrible, like, because of work it just in time. We just have no time to eat. And when I get home I just want to sleep. So I just I just still eat, maybe like on average, I eat one meal a day that time. I think. So. Yeah. So to compensate for my week after I got I became 36 kg, then I just like loading up like very unhealthily for a period of time, just to increase my sugar intake, you know, just to bring my weight back. Yeah, yeah, it a bit extreme. But I think now it has sort of like, reach the equilibrium which is about 39 , 40 , which is still still less heavy than my, uh, than my past ah. Yeah. Sort of, okay now
- **NJCL: Back then like, had no time to eat or- can elaborate on that?**
- P9: So uh, for AnE. Our break time is 45 min. Yeah. So I guess it's also dependent on like the access that we have for food like. So within my hospital, like 1-3 years we didn't have any like, no food court in the hospital, you know. Like if you wanna eat. Then you must like travel off the hospital. Then you go and buy food. Yeah. So uh that was one like sort of obstacle right then, sometimes I tried to bring food from my own home. But yeah, like I said, cause. Hmm. I guess in a private hospital uh, the manpower is definitely less as compared to a [inaudible] structured. So uh, it really like. When they are really like just that many patients. And so you sort of just have to sacrifice your own break time to to to just deal with the patients lah. Yeah. Cause. Yeah. Then, earlier, I also mentioned that so in charge, you have to oversee everything right? So when you're in- As an in charge, right. Then you feel obliged to make sure that the rest of your team gets to eat. So usually, you sort of assign yourself as the last person to have food. Yeah. So a lot of times I sort of make sure everyone else in the teams eat first. Then, if there's a extra time then, okay, or if I can afford the time, or, you know, like things are stable, things are going okay. And I I feel that oh, my presence can be absent for a while. Then I'll go and have food. Yeah. [NJCL: Participant sniffs]
- **NJCL: Then. Then, also, just now you mentioned, like. You'll.. To compensate losing that much weight, you sort of like, started like, building up? Can you elaborate on that? Like-**
- P9: [NJCL: Participant laughs] Just eat, I just have like very high like, high caloric food. Yeah, like, intentionally, cause I when I was working I didn't realize the weight lost like until I saw a photo of myself. And then I was very shocked. [NJCL: Participant laughs] Yeah, I became so skinny, yeah, then, I just I just like, and I didn't eat very healthy things actually like, I really just bought doughnuts like, I just eat like really high calorie things, too. Yeah, to compensate lah. Yeah.
- **NJCL: Uh, is it right for me to say you started that because you- was shocked by the amount of weight loss like? What- What motivated you to actually like, get back that weight?**
- P9: I I I yeah, I guess you can say I was shocked. It's just like I felt like, Hm. that weight was just a bit too abnormal for me already. Yeah, it's just yeah. It's like a bit. It was a bit too excessive lah. So I thought that. Yeah, I should do something about it. [NJCL: Participant sniffs]
- **NJCL: Okay. Then. Moving back a bit when I yeah, I'm just gonna summarise a bit of what you mentioned earlier.**
- P9: Mm mm!
- **NJCL: So when I asked about like, How would you define a healthy lifestyle? Your answer was mostly on how to- How do I say this? Ah, you answered, mostly on what is considered unhealthy, which is not being able to come- Compartment- compartmentalize your your life and your work. Is there anything else you want to add on, how to define a healthy lifestyle?**
- P9: Hmm. [NJCL: Participant moves head while thinking] I guess, I I think what I said earlier was probably more of like mental and emotional health. Like for me. Personally, I don't focus much on physical health ah. Yeah. So uh, I I don't have much to say about that. Obviously, you know the ideal, the ideal, healthy lifestyle. You know, it's like, ah exercise 3 to 5 times a week, eat healthy food, have a good diet, have you know, regular sleep, water, that sort of thing. Yeah. But uh, just personally for me. Uh. It's like, I think I mentioned about like the you know, the mental, and the emotional part. It's because, like, possibly, like for me specifically, these are the aspects that I struggle a lot with when I started nursing. Yeah, the physical aspect has like, never, really been a problem. So I just don't really think about it. Yeah. [NJCL: Participant nods head]
- **NJCL: So when you when you said like mental and emotional health. Okay, but like compartmentalising work. And your life right? Can you elaborate on the emotional part?**
- P9: So yeah, I think this can sound very cliche, but sort of. But like, really, as nurses, you see a lot of like sickness and death right? And uh, I guess at one point you have to deal with your own emotions. For example, about a patient's passing. Yeah. So yeah, uh, I remember, really, when when I started out working and like the first death I encountered. And it really hit me quite hard like, personally, yeah. So from that point onwards, uh, I realize I just got to like, regulate my own emotions. Yeah. But uh, I have to say I do. I don't really have a solution for this. Till now. It's something that I still struggle with at times. Yeah. So yeah lah. [NJCL: Participant laughs, clears throat]
- **NJCL: Okay. Then, I I think you touch upon it a bit. But for my next question it's about, as a nurse, how do you feel about self-regulating a healthy lifestyle?**
- P9: I would say that. You mean like self regulating like, I sort of self initiate this like, healthy habit is it?
- **NJCL: Like with reference to what you were talking about in** **in how you answered the having, the how you define healthy lifestyle earlier**
- P9: Hm.
- **NJCL: Like, how how do you- cause to you personally mentioned, like emotional and mental health is like important like, how do you feel about actually self-regulating this kind of lifestyle?**
- P9: Hmm, I would say that, this thing is like, not really done alone. Right? Like, I think you need a strong, like social support for this. Yeah, so uh, you know. So like having just like your colleagues or co-workers, or like, you know, people from the team to discuss about like, maybe certain things that happen to a patient, and you know, just like you, just you just don't feel very good about it emotionally. Then talking and expressing your feelings to your colleagues can sort of.. It's sort of like, it just helps to cope, you know. I would say, it's like a coping mechanism. Yeah, cause you sort of like rationalize your feelings and everything. Yeah. And hearing the opinions of the rest of your team, will also make you feel like you're not alone, I feel. Yeah, cause you know that you know, others are also experiencing the same kind of feelings. Yeah. Then. Yeah. So I would say that, like, in in my nursing career. Right? I had a, so I had a preceptor who was my preceptor when I was a student, so we eventually became friends, and her having more experience than me, right? Yeah. Knowing that I have a friend that I can go to for advice, and knowing that she has probably gone through similar things. Yeah. So like, I can sort of, like gain life experiences from her. So there is a thing that I use to sort of, to cope with my own struggles, I guess.
- **NJCL: I see. I see. Okay. Then, to summarise for you, for the question, as a nurse, how do you feel about self regulating a healthy lifestyle, mentioned that having a social support is very important. Like having friends. Come and express yourself in some way or similarly, we have a very experienced preceptor that you can lean on whenever you need. Is there anything else you want to add at this point?**
- P9: Hm, no
- **NJCL: Can. Then, moving on to my next question, it's about what are your main facilitators, motivators in self-regulating healthier lifestyle?**
- P9: I'm not sure how to answer this question. [NJCL: Participant laughs] Can give me some examples of what people say?
- **NJCL: It’s okay! I think what you mentioned earlier about like the social circle can be considered like your facilitator or facilitator to maintaining.**
- P9: Yes,
- **NJCL: Yeah**
- P9: Yes. But yeah. Motivation wise? Uh. I don't think I have any motivation [NJCL: Participant laughs] to self regulate
- **NJCL: Your like.. Maybe thinking about like, physical activity, wise diet? What is motivating you to continue with that kind of lifestyle?**
- P9: Hmm. Hmm. I would say that like for diet, right like, I what I said, I don't really pay much attention to diet right? So I don't think I can say that there's anything that motivates me, because of how I approach my diet is that.. To be honest, yeah, I guess a lot of it is based off like from my experience and work right, if I if I don't really have time to eat at work already, and I'm working so hard. Then, after work then, what I think to myself is that since I work so hard, then I just want to eat and buy what I want. Yeah. So uh, which is why, like, I don't think much about the health consequences of my diet, I think it's really just for like, possible satisfaction. Yeah. Then, I guess, like uh, exercise, for example, like, for example, now I started to, I started to get back into dancing. Then it's like, I guess it's more of like sustaining an interest or hobby. Yeah. So like, having interest out of work is important. Yeah. Cause. Yeah, work, it's not just gonna be like, the only thing you do. Yeah. So having interest and yeah lah, and other stuff. And I still think that yeah, social support is important, though. Cause, uh sort of having friends that can sort of pull you out of work, you know, and be like engaging in other activities is also equally important. Hmm. [NJCL: Participant nods, rests chin on hand]
- **NJCL: I see**
- P9: I have nothing else to add [NJCL: Participant laughs]
- **NJCL: Can can can. Okay, then, moving on from facilitators. What are your main barriers in self regulating a healthier lifestyle? What is stopping you from becoming healthier? In other words.**
- P9: Hmm. I guess fatigue from work itself. So hmm.. I just don't have enough energy to want to go for a run, for example, yeah, or even at times now, when I sort of still can go for dance classes after, perhaps I know, early in the week I'd be thinking, okay, like a Friday night uh, after my morning shift, I'm going to go for a dance class. Then, when Friday actually comes. Then I'm just too tired. [NJCL: Participant laughs] Yeah, I just say, Okay, I think I just want to sleep. Then I just stay at home and I sleep. Yeah. So uh, that was one thing, I think fatigue. And.. Hmm.. I think that's about it lah. Yeah, yeah. Yeah. To be honest, I think I'm actually like, at this moment, I'm quite satisfied with my lifestyle. Yeah, I feel like I've I've gone. I've came a long way from like 4 years ago when I started nursing to reach this point where I feel like, I really feel okay. Like I I sort of have everything in balance, though it might not seem healthy like [inaudible]. [NJCL: Participant laughs] Yeah
- **NJCL: I see. What what do you think is like the main difference between 2 years than now that focus, while you're in your words, satisfied with your lifestyle now, compared to [inaudible]?**
- P9: I think 4 years ago was when I just started out as a nurse, right? Just uh, just like okay within a work setting. It’s just, it's hard to feel confident about the things that you do, because you are very new. So yeah, at a point where you’re constantly learning. And you're trying to keep up with the rest. And like, really, just like trying to cope within the work environment that you don't have much time or energy to think about life outside of nursing. Yeah. So even, you know, like after work, then you think about all the things that you don't know at work. And yeah, it's really, it's really like the entire thing. And the entire time I’m trying to like, like, keep up with the rest. Yeah. Then, then I would say that, yeah, so uh, my situation is, might be a bit different from others, because also because of the hospital that I worked in so uh, I had, cause I'm bonded to the hospital. Then uh. I had to rotate departments 3 times lah, so that was the requirement. So I stay in one department like 6 to 9 months normally, then, I change to like a different setting. So because of this rotation, every time I’m put in a new setting. I sort of have to like, do everything all over again. Like I I just, I reset again. Then I like, I feel I’m a newbie again. Yeah, I'm learning. And I'm trying to keep up with the rest again. Yeah. So only like, at the beginning of last year, did I finally settled in AnE, so I think it's just the, like being having- being able to stay in the same environment and sort of like building up my skills in the same area just makes me more confident. Yeah, so like, now, like, finally, I think this year, I feel like, okay, I feel like, steady at work you know, just like. I know that I can also be a reliable team member like, I feel happy about myself. And then now, finally, like this, work part is settled. Then I can move on and explore other areas of my life. Yeah.
- **NJCL: Earlier, you mentioned about like, having to like keep up with the rest like, how how did you self-regulate- your thinking change from like thinking of like, keeping up with the rest? How your mental health is right now?**
- P9: You mean, like as compared from then and now, is it?
- **NJCL: Yeah**
- P9: Like my way of thinking?
- **NJCL: Yes, yes**
- P9: I think it, it really like, uh. You change because I just like, I gradually like did improve. [NJCL: Participant laughs] So it's really like, after getting more experience, then I know that I am better now. Therefore, my thinking changed. Yeah.
- **NJCL: Ah, okay. Okay. And so, I'll just summarise- unless you want to add anything at this time?**
- P9: Hm, no. Don’t have
- **NJCL: Okay. Then I'll just summarise what you've been mentioning so far, like regarding your main facilitators. It's mostly on having a having, like a friend- a social circle that encourages going out, having hobbies outside of work. That's your main facilitator that you mentioned then for barriers. It's mostly fatigue. And maybe the fact that like, personally satisfied with your current lifestyle. So this doesn’t make you change?**
- P9: Yeah [NJCL: Participant laughs]
- **NJCL: Okay. Is there anything else I'm missing out for the facilitators and barriers part?**
- P9: I think no.
- **NJCL: Then we go to my next question. It's regarding your how does your like self regulation habits impact your work performance?**
- P9: I think the only habit I said I went to dance, right. [NJCL: Participant laughs] Uh. I wouldn’t say.. Uh. Okay. It doesn't affect my work performance. I I I think, whatever I do. But so like, okay, so I I guess it's more about like, trying to have a hobby out of work now, right. Like what I said earlier. So uh, okay. So one thing that affected me right, was that. Well, I feel sometimes at times I will feel frustrated. Because, for example, perhaps I know, like after my shift. Then I'm going for dance right? And then at times work can get a little unpredictable. Then, you know, it's sort of like. You- maybe I don't get to end on time. Then I end late, and then, therefore it then affects the activities that I have after work. Yeah, all that. So this dancing that I'm doing is sort of like a regular session every week, right? And again, shift work is not regular, right? So I really have to put in like, I really have to intentionally have to request to make my- that particular day afternoon- like Suddenly afternoon. It must be free. So then, you know, it's quite a lot of trouble, because, first of all, I have to talk to my my sister right.. Then. Then again, how do I ensure that she definitely will grant my request? Right? So that is a consideration, because otherwise I cannot sign up for something and pay for something that I know that I cannot attend regularly. Yeah. So that's the part that actually, even at the beginning of nursing where I told you that I stopped dancing right? So this that was also a factor that eventually led to that decision, because I found it very difficult to have a regular schedule, and that request. Like for shifts cannot always be granted. [NJCL: Participant gestures with hands] Yeah, so and now I'm finally trying to have a regular hobby. Then I'm facing some trouble, you know, trying to to just free up that part of the week to to do that. Yeah. So there is some frustr- frustration in that I would say. Hmm. But again, yeah, it doesn't really affect my work directly. It's just how I feel. [NJCL: Participant laughs] Hm.
- **NJCL: Alright. I think outside of like, physical activity-wise, and like, diet wise. You also mentioned about your mental like, your way of thinking so like, can you elaborate on how? Because you mentioned something about well, now, to gradually improve lot more experience, your way of thinking change also. So how do you think did this do anything work performance, like your way of thinking?**
- P9: Hm. I think it doesn't change my work performance. But it changes the way I feel about work. In the past, I always felt like, I guess work is sort of responsibility right. Then. You know, generally lots of people like to talk about work life balance. Do you think that work life balance is sort of healthy, right? Then. Yeah. So in in the past I felt like I was like, so consumed by work that I had no life off that. But these days. I treat work as like part of my life. If that makes sense like, I don't necessarily like separate it with my personal life. I I I sort of integrated into my own life. And then, when I stopped thinking about the liability. But just, you know, part of something I enjoy, because I actually do enjoy nursing. Yeah, just by changing that that way of thinking. Then I approach work like, I I guess. Just I think of. I think less of it as a burden. But just just sort of like . I guess you can even say like, it's like an interest passion thing, you know, you just doing something that that you like to do. Yeah. Then you just feel like less burdened by it. Hmm.
- **NJCL: You mentioned work life balance, will you elaborate on that, and in terms of that?**
- P9: So actually, [NJCL: Participant laughs] now, I don't. Now, uh. It might be. It'd be strange to say that. But no, I don't think there's like, such a thing as work life balance actually. So. If like so recent recent recent months, there was this article right, by this, you know the DBS CEO. Yeah, I think, Gupta, yeah. So he he received a lot of attention because in an interview he said that he doesn't believe in work life balance which in the past, I guess I will have been like one of the haters right. But now, I I understand what he means, because he says that he just believes that workis is a part of life. Yeah, I I. So it’s a bit hard to explain it, I guess. But like, for, for example, I I I have seen people that, you know, like hate getting like calls after work. They think that, you know, including on my personal life account thing like, Oh, I will answer your emails after- until I'm in the office, that sort of thing. But I- like now, I no longer think that way. I guess it's just that I I'm okay. If I I guess if I'm an office worker. I'm okay with being contacted because it's just a- like you are responsible for your own work and, I'm sorry I don't really know how to explain it. But-
- **NJCL: It’s okay**
- P9: Yeah, but it's really just like you sort of treat your work as part of your life. Then then you- Then there's no there's no like boundaries, like obvi- Obviously, things still have to be reasonable. But you don't have to draw like such lines to, you know, and you know, like anything about work crosses like into your personal life, and you get pissed off that kind of thing. Yeah, yeah. [NJCL: Participant adjust self in seat]
- **NJCL: Then. Is it right for me to say that like. Do you personally, or your identity will be not yourself. How you identify yourself, would be like separated from your nursing identity, like your identity at work, and your identity outside of work is mostly similar, the same?**
- P9: Yeah, I would say, it's the same. Yeah.
- **NJCL: Okay. Could you maybe elaborate? Why, why you think it's the same?**
- P9: Oh, okay, when you mean by identity, right. Can you like describe what you mean?
- **NJCL: Uh**
- P9: My identity at work versus my identity out of work.
- **NJCL: Cause maybe. How a person behaves at work might be a bit different from how they portray themselves outside of like that kind of idea.**
- P9: Hm..
- **NJCL: Is this applicable for you?**
- P9: Hm. I would like to say that I think I'm pretty similar. But uh, actually, I would. I think I would prefer having a- I would. I don't wanna say different identity, but uh, I would prefer that my work identity, identity, personality, or I would prefer it if it is slightly different from my personal one. Because I would say that, like like my my personal, my identity in my personal life, right like I do, have, I do have like, negative traits as well. I guess you know, like I have a bad. I have a bad temper, and what not so like this sort of things obviously, I I do not want to show it when I'm working. So I would say that you know that I try. I I would prefer that it's different. But unfortunately, I think it's pretty similar, like now, like, you know, parts of my personal life. Personality is like trickling trickling into my work, and you know sometimes, at work I I also like, you know, get pissed off at things, and and then I show my temper, which is something that is not ideal. And I I don't want that lah. Yeah. So I don't think it's a bad a bad thing. Yeah, there's neither good or bad. It's really just uh, I guess what kind of person you want to portray yourself lah, in a professional setting the first in your personal life. Yeah.
- **NJCL: Okay. And then my next question is regarding like like, are there any like tools or apps that you are personally using? Maybe self regulate your lifestyle?**
- P9: Uh. The only app I own is a, I don't know whether you heard of it, it’s the [inaudible] app, a lot of the nursing-
- **NJCL: [Nodding] I’ve heard, I know**
- P9: The nurses use it right, [NJCL: Participant laughs] yeah, yeah, so that’s the only I think, useful app I have because uh. Yeah, I still have a few friends from school that I still keep in contact with, and interestingly, so after working for 4 years after I graduated 4 years ago the 4 of us have only met together once. [NJCL: Participant laughs] So it's just like-
- **NJCL: Once?**
- P9: Yes, so it has been extremely, extremely difficult. And you know, really, we use the app right to keep track of like one another’s shifts. And, it’s like, just like that is just impossible that all 4 of us will be available at a certain day or like, at a certain hour. That sort of thing. Yeah. So we still keep in contact like online. But really, like the last time we met was was 2 years ago. Yeah, so yeah, yeah. But I would say that that app sort of help us, you know. Sort of plan dates no, or like even with my other friends who are not from nursing. But they also do shift work. So they use the app as well. And yeah, it's just easy to to sort out our, like our common times, so that we can meet. Yeah.
- **NJCL: Okay. So other than the app, no other apps lah?**
- P9: Hmm, hmm. [NJCL: Participant looking down, might be looking at phone offscreen] Nothing lah, nothing already. [NJCL: Participant laughs, not looking up] My health app.
- **NJCL: Okay.**
- P9: Uh. [NJCL: Participant still looking down, might be looking at phone offscreen] Yeah, I I don't. I don't use any particular apps
- **NJCL: I see. Okay. Can**
- P9: I see ah.. [NJCL: Participant mumbling, still looking]
- **NJCL: Alright. Then, for my next series of questions, it's more regarding workplace in general. So like, as a nurse, how do you think the workplace influences lifestyle habits?**
- P9: I I would say that. Oh, yeah, the the eating thing ah, yeah. Like, I guess. Just yeah. So for my hospital, like within our A&E, we do a few funny shifts that we have the regular AM. PM night shift, and we also have these like, shifts that are longer hours so they can be like 13 h long, 12 h long. That sort of thing. So, for example, for our 13 h shift, we have one 45 min break, and we have one 30 min break. So uh. So most of the time. I would say that, like 50% of the time when I'm doing such shifts right, I will get one proper meal and the second break. I either have no time to go go at all, or I just like have a snack. Yeah. So I would say that like, work definitely affected my my eating habits, and just like, just like uh, very irregular meal times. Yeah. So I I I would say that, yeah, that is not healthy. I agree. Yeah. And hmm. Yeah, like, I said earlier about the fatigue lorh. So it it sort of impedes me trying to get, you know, exercise. It's like I have the intention to. But yeah, just like the fatigue is just too much at times. Hmm. [NJCL: Participant sniffs]
- **NJCL: The the fatigue like, beats the intention?**
- P9: Sometimes yes.
- **NJCL: Okay. Then so just to summarise, you mentioned about, you know. Like your meal times are being affected by the workplace. The fatigue is also affecting how much of your physical activity. How about like, how does your workplace maybe influence emotional, mental health?**
- P9: Hm. So I guess. Uh. The A&E actually improved my mental health. [NJCL: Participant laughs] So one of the reasons I chose I chose to stay there was because, like I think earlier in the interview I mentioned about, you know like going to sleep and still thinking about your patients the next day. You know that sort of thing. I'm glad like within the A&E setting. I don't have to do that because you know, the patients like it's a touch and go, you know. You sort of like within the A&E setting, you stabilise the patient. Send the patient to the ward right. And then you'll never see the guy again. Yeah. So uh, some people may not like it, but because that there is no like, continuity of care right. I I do not have to think about like the patients, you know, after work like I'm done. I don't think about “Oh, do you know, tomorrow tomorrow, when I go back, for shift, yeah. I know I'm taking the same cubicle of patients.” Yeah, you know, like, “oh, how complicated the case is”. It's gonna be so busy like, “oh, I know that the patient is planned for”, you know whatever things the next day. Then you don't have to go and worry about these things, cause there's nothing to worry about. Yeah. So for me, it is a positive thing. Yeah.
- **NJCL: Can can. So for you it, the workplace can both positively and negatively influence you**
- P9: Correct. Yeah
- **NJCL: I see, I see.**
- P9: Yeah. And I would say that like my workplace, I would say that generally it's a positive one like. It probably is a net positive rather than negative, cause. Like. Like to be honest, I I don't really place a lot of emphasis on diet and whatnot. So in the first place, it's not my focus lah. Yeah, my focus has always been more of, just like managing my emotional health. Yeah. Hmm.
- **NJCL: Okay. Then, my next question is. How do you feel about the workplace culture among nurses in helping to facilitate or hinder the self-regulation of healthier lifestyles?**
- P9: Hmm. [NJCL: Participant purses lips while thinking, shifting eyes around] I find it a bit hard to answer this question, because I don't have much healthy, healthy, lifestyle habits. [NJCL: Participant laughs] But I will say that. Sort of like, feeling as if you are working in a team is important. Yeah. So knowing that uh, you have the support of your colleagues right like. But like for me, it makes me feel at ease. Yeah. So I would see that- you can see that like mental slash, emotional health wise. I am okay because of them. Yeah, or like, even, you know. Uh, earlier when I said that, you know at times in the beginning, in the past I will feel adequate, right as a nurse, like, as I am lacking. So even we didn't like. Now, at this point, though, I have said that, you know, I know that skills wise, I might have improved. But of course. There, you know, you know, in health care there will be a million of things that like, even though you have never encountered them, you don't even know that they exist right? So though, you don't even know how to manage that thing. So I I am also aware of my own shortcomings. Like, I would say also because of the lack of experience. So knowing that behind me is a team of like seniors, you know, that I can always depend on. Then, it is very reassuring to me. Yeah. Hmm, yeah. But otherwise, hm yeah, I I will also say. Hmm. They also act as a form of social support. Yeah. So for our department. Some- A few of them are quite [inaudible], you know, they sort of go for runs. And they’ll ask, you know, like whether you want to join them, that kind of thing, I I guess these can sort of help to incorporate some healthy lifestyle habits lah, yeah.
- **NJCL: Then, moving away from like facilitators of like. How your workplace culture actually facilitates healthy lifestyles. How about like, barriers or hindrance? Do you think the workplace culture actually can hinder your self-regulation of a healthy lifestyle?**
- P9: Hmm. I can only say about the eating thing. [NJCL: Participant laughs] Which I think you know already right. I’ve said it earlier. Yeah, it's just hmm. Yeah lah. It's just like uh, sometimes you just have so many responsibilities that you just have to sacrifice your your own time for the patients. Yeah, and like for the work. Hmm.
- **NJCL: And then. Moving onto my next question, it's about what do you think about the role of nurse managers in helping nurses like self regulate their health?**
- P9: I would say that, for my situation right, like during the crazy COVID times. Very often our managers, and, like the NCs, the clinicians they usually be in office. But during those times when we, you know, like the the number of patients just really exceeds like what the manpower of the nurses can provide, they really like come onto the ground very often to cover for us. And yeah, and because of them, because they're covering for us, you know, we can, we can actually rest and go for breaks and have food. And you know, just just like, take a little break while we are working. Yeah. And earlier, I also mentioned to you regarding the rosters right. So like no- having a an assigned free time per week just to go and pursue my own interest. Yeah. So uh. So I I think I'm quite lucky to have an NC that that is quite understanding. And she yeah, sorry I have like told her, and even last year, when I just did some like other classes, you know, out of my own interest. Then I just told her like, Oh, you know, like, just on that I may- Every Tuesday, can you like, let my- every Tuesday night, I just want it to be free, so you can put me on AM shift, you can put me on like those funny shift, you know, those 13 h shifts. But as long as like, those hours of that day is available. I'm okay. So and that's what she did. I just told her, you know, that things gonna last 8 weeks, and every time I put in the request, then she so, and she will see it, and she will grant me the request. So yeah, I guess it depends on how understanding the sisters or the managers are. Yeah, and how, how willing they are to, you know. Like, just just like, give you a favour lorh. Yeah. Cause I've like through my experiences, in other wards as well, they- not all of them tend to behave this way, so I guess I just got lucky. Hmm.
- **NJCL: To summarise, the role of nurse managers in helping nurses self regulate their own health, for you, it's mostly they actually like go on the ground to help the nurses, the buyers that they can replace to have their meals. And so, they- them being understanding and actually allowing activities outside of work. It's like they're they're helping you facilitate. I see. Okay. Can. Then, earlier, about the workplace culture of nurses, the facilitators and the barriers mentioned that for facilitators it's mostly the- Uh, how do I say this. Okay lah, for for barriers is mostly the it's the food you mentioned. And then for facilitators, it's like the social circle of being in a team and having a team behind you so that you can feel assured.**
- P9: Mhm. [NJCL: Participant resting chin on hand]
- **NJCL: So that's for the workplace culture of nurses. Do you have anything else to add for this part?**
- P9: No
- **NJCL: Okay. Then. Okay. into the next question, it's how do you think your institution can help you to self-regulate, maintain a healthier lifestyle?**
- P9: Hm. So for my organisation, in the past. So we have uh. [NJCL: Participant leans head back] We had this uh, recreational club. So we do have some managerial people who are in this club, and they help to sort of, facilitate some- some- exercise- exercising opportunities, you can say so. In the past they have worked with some, I think yoga studios sort of thing. And you know, they help to obtain like corporate rates for the hospital staff. And yeah, they have approach like studios just opposite the hospital, you know. And they arrange sessions for our own staff to to attend. Yeah. So I guess these sort of increases, you know. The amount of physical activity that we do. Yeah. And hmm.. [NJCL: Participant squints] What's the question again? About what can the hospital do to facilitate healthy lifestyle? [NJCL: Participant smiles]
- **NJCL: Yeah. Mostly. Yeah.**
- P9: Hm. I would say that it would be great if the hospital can have like, our own food like, a staff canteen. That would be a good option. Yeah, we don't have that. And uh, I've heard of like, other private hospitals that that do that so, and they sort of offer, you know, even like meals at like a staff like a discounted price. Yeah. And just I guess if the hos- the hospital can sort of have a canteen, they offer, you know, some healthy meals. I'm sure it would be popular, and it would be helpful. Hmm. Hmm. Yeah. Uh, I've heard some talks about the management planning to open a gym within the hospital for our own staff. [NJCL: Participant laughs] So that that can be a consideration. Yeah. Yeah. But I think that's about it lah. I will say that our hospital doesn't do much [NJCL: Participant laughs] for its people.
- **NJCL: Okay. Earlier you mentioned the Recreational Club, and like, the- your supervisors, actually like organising activities and all this. Do you personally think it's effective in helping encourage healthier lifestyles?**
- P9: I would say, specifically for nursing wise? Not really. So. I've seen some of the up- the the the people who take these like, these- who attend this event right. Usually they are the corporate side or the admin side of the hospital because they have the time. [NJCL: Participant laughs] Because they they usually these events are held, you know, after office hours in evening or night times. Yeah. So the Admins. Usually the Admin people would go for these things. Yeah. Whereas, nurses wise, yeah, it's just like, just work, I guess. Then, usually we don't have like the time, or energy to attend these events. Yeah.
- **NJCL: Have you personally attended one of these activities?**
- P9: No, I have not. [NJCL: Participant laughs] Hmm.
- **NJCL: Then how about the opening of the gym? Uh, do you think this will be a effective way to encourage healthier lifestyles?**
- P9: Hmm. It is possible, I would say, because I guess, just like the proximity to the gym itself, already serves as an incentive to go and exercise right, and uh, and I guess if the access to the gym is like, complimentary for the staff. Then that is even better right. Cause- I don't know. Actually, I don't gym, so I cannot comment, [NJCL: Participant laughs] but I I guess the gym membership can be costly right? So yeah. So I guess with the the lack of barriers, then people would probably be more incentivized to to go. Hmm.
- **NJCL: For your institution. Are there any like, workplace health promotion programs in place? Like?**
- P9: Mmm. [NJCL: Participant thinks, looking at ceiling] Health programs is it?
- **NJCL: Yeah.**
- P9: I I don't think there are any. Yeah. So… Hmm. No lah, but we have the typical, I guess. That we have this thing, I guess maybe probably all institutions do, you know, if you sort of gone through some sort of like traumatic experience while at work. Yeah, then you get like uh, you get to. Then they will send you for counselling with the counsellors, you know. Like, just to, just to sort of like talk your feelings out. And yeah, that's sort of thing. But other than that. I don't think we have any. Uh, oh, though, I think I just saw an email today. I I think that the hospital is offering all employees like a basic health check up. Yeah, yeah, yeah, yeah, like, yeah, I think that's it lah, just just the health check up.
- **NJCL: I see. Alright then, moving to my final question, outside of work, what do you think influences your lifestyle?**
- P9: I would say uh, my life outside of work, or even work is- For me, it's very self-driven. So uh. Like my habits or my lifestyle are are usually because of my interest. And what I want. Yeah. And it's like. I do whatever I want to do physically. Yeah, I I guess I some of my friends. So I have influence from my friends as well, I guess, like, for example, dancing, you know, like still having this sort of connection, and being able to talk talk to them about it. Then it sort of drives me to want to go and do it more. Uh. Yeah. And hmm. Otherwise I would say, there isn’t- there isn’t that much. Yeah, yeah. [NJCL: Participant laughs] Sorry, I can’t really answer this question.
- **NJCL: It’s okay. Yeah, okay. Then let me summarise for you what you mentioned yeah. So for you, it's outside of work for you. It's mostly self driven- uh actually, maybe elaborate, more on like, what you mean by self motivate?**
- P9: Hm. Cause your question. When you ask your question, you you said like what motivates me, right?
- **NJCL: What influencers-**
- P9: What are some motivat- yeah, what influencers, or like out of work right? Uh. I would say that nothing really influences me, [NJCL: Participant laughs] and I guess that's what I meant by like self driven. Whatever I do, is sort of like whatever, it's all just what I want to do. Like like like, nothing, nothing influences me to to do whatever I do. If that makes sense. Yeah.
- **NJCL: Like, your your influence is- Sorry ah, raining. So your influence is mostly from inside of you- yourself. If that makes sense. Right?**
- P9: Yeah, yeah, yeah, yeah. That’s what I meant, I guess yeah, yeah, I I, yeah, but like, I don’t know if that makes sense [NJCL: Participant laughs]
- **NJCL: It it makes sense, it’s just that I- it’s very hard to descri- Very hard to say. [Laughing]**
- P9: Yeah. Yeah, it’s just like, yeah yeah, I, yeah. Like no external. Everything is like, internal. Yeah, like everything. Everything. I-
- **NJCL: Intrinsic?**
- P9: Yes, yes, yes, everything that drives me is intrinsic, like. Normally, I'm not very affected by like external factors. Yeah, so yeah, that's what I meant by self driven. Hm.
- **NJCL: Okay. Okay. And then I'm going back to summarising. So outside of what influences you is like, said intrinsic factors, your own like your own interest sort of way. Then, other than that, it's also the- your friends by having a common, maybe common hobby like dance for you. So having friends can- can connect with. That is also another influence for you. Other than this, is there anything else that influences it?**
- P9: Hmm. No, I guess.
- **NJCL: Okay. Alright. Okay. Can. Then, do you have anything else that you like to speak about? That was mentioned in the interview. Any further comments you want to share?**
- P9: Hm. No. [NJCL: Participant laughs]
- **NJCL: Alright, then this marks the end of the interview. Thank you for your time and participation.**
- P9: No problem[NJCL: Participant laughs]
- **NJCL: Thank you.**
- P9: Thanks.
- **NJCL: I stop the recording.**
- -End of transcript-

# **Interview #10**

Participant and interview details
 Participant number: 10 (P10)
 Date of interview: 28 Sept 2023
 Interviewer Number: Navarro John Christopher Lambino (NJCL)
 Time IDI started: 0900
 Time IDI ended: 1050
 Observational notes: Participant has a cheerful disposition.

Section I: Interviewer’s summary
 The individual interview was conducted via Zoom. Conducted early in the morning, P10 is my first participant who has had employment in polyclinic. P10 was expressing much on the fact that she provides various healthy lifestyle advices and yet, she herself does not follow/compliant to her own advice. She mentioned that she may be more convincing if she follows her own advice. She describes it as a ‘nagging’ feeling, and no mention of ‘guilt’ was mentioned as previously by P9(?). Friends and social circle are also said to be of lower influencing factor for her as she is quite lazy/lack discipline to make changes. However, she interestingly mentioned the fact that her family has a history of diabetes, and her fear of this chronic condition as well as seeing a lot of other patients with chronic diseases. Her concern for her well-being is a major influencing factor for her. Fatigue is also a major factor and despite not having shift work in polyclinic, time is still interestingly a factor to planning self-regulation habits for her. There is also the idea of a ‘reward’ either through indulging in good food or just having a day to rest. P10 also does not like to exercise in general and requires someone to accompany her. The availability of food choices is also a major hindrance for healthy lifestyle choices. Nursing managers, in her opinion, also do not care about health of their ground nurses. That is their own responsibility, according to her. She also elaborated upon the demands outside of work that may hinder self-regulation/changing of one’s lifestyle.

Section II: Transcript

- **NJCL: Okay. Good morning, miss. Firstly, I would like to thank you for taking the time to speak with me today. My name is Chris, and I'm a NUS Year 4 nursing student. The aim of this interview is to get a better understanding of the perspectives nurses have on self regulating their health behaviors and how various factors may influence this. I want to find out what are your views, opinions, and experiences on this. I would really like to encourage you to speak what comes to mind. There are no right or wrong answers. May I have your permission to continue video recording this interview session?**
- P10: Yes.
- **NJCL: Alright, thank you so much. Then let's start with some basic questions like, which ward were you working in?**
- P10: I was in the polyclinic actually.
- **NJCL: Could you describe to me like what kind of work you were doing?**
- P10: Oh, primary healthcare! I was a senior staff nurse in primary health care since I graduated. So for primary health care, nurses are divided into 2 different groups. I think it depends on the institution, but our institution divides into acute care or chronic care, I was under acute care. And basically, it's everything other than care managing patients. So that's what chronic care nurses do. Acute care nurses do all the other services, dressing, child immunization, adult immunization. We do have triage. We do have treatment services. Diabetic retinophotography as well as foot care. Yeah, I think that's pretty much it.
- **NJCL: How will you describe your workload? How would you describe the amount of work you have to do?**
- P10: Oh, the workload? Okay, I think it depends on the different stations that you're assigned to, the different roles that you're assigned to on a daily basis or on a weekly basis depends on the institution as well. For my clinics, specifically my roster is set, however, depending on manpower, I might be shifted on a daily basis. I think there are certain stations. There are certain areas in which the patient load is high, especially for vaccinations. Child immunisation is also high as well as dressing is is a bit heavy. I would say, because I was rotated in child immunisation, vaccination a lot more it's quite a bit to handle. If you were to compare to acute care in a hospital. I think I think it really really differs quite a bit, so I might not be able to give you a comparison on that. But yeah, depending on the stations we were assigned to, the workload differed as well.
- **NJCL: Hmm. Then, just to get a comprehensive understanding of your work history, you were always are working in a polyclinic institution?**
- P10: Oh, okay. So since I graduated in 2018, I started my work with Polyclinic, because I wanted the office hours. So yeah, since then I've been in a polyclinic for for the past 5 years. Just one institution.
- **NJCL: Yeah. All right. Thank you for that. Hmm. Then, moving on to my next few series of questions, it will be regarding lifestyle in general. So as a nurse. How would you define a healthy lifestyle?**
- P10: Oh, I think this is part of something that we educate, especially in a polyclinic, I guess. Before our institution separated the nursing services to chronic and acute care. We did kind of do a bit of chronic work as well. So that was probably the first 2 years of my nursing career. We do have the basics of exercising, I guess. The healthy plate. Yeah. Different diet managements. Educating patients on different fats and cholesterols, sugar, salt. I think healthy lifestyle means incorporating all these advises that we've learned and we've been giving our patients. Yep
- **NJCL: Actually, could you elaborate to me the kind of advices?**
- P10: Oh, okay. So, for example, healthy plate, you do know that half of your plate should be fruits and vegetables. A quarter of it should be protein. A quarter of it should be carbs. Then you are also advised to exercise 3 times a week minimally, and it has to be a certain level of exercise at least half an hour for 3 times a week. For a different sugars, you are advised to limit and if you can try to choose the healthier drink option, healthier beverage options. As for salt, I believe apparently there are recommended amounts of salt that you're supposed to take. So that might mean changing your choices when you choose the food options to something that's less salty, I guess. Yeah.
- **NJCL: And then specifically on diet. Since you were talking about food choices, how do you feel about your current dietary habits?**
- P10: Oh, okay. I think I- to give a background, my family has a very strong history of diabetes especially my paternal side. My dad has diabetes. His siblings has diabetes, his parents also have diabetes, so I've always been self-conscious. I do try to make changes, but I do eat quite a bit of rubbish food. [Participant laughs here]. So I don't think I'm very healthy. I do enjoy really unhealthy food. But if I've eaten that in a row. I will try my best to change it up a little. I think, when I was still adjusting to the first few years of work I did not- I ate out. I eat out quite a bit, but after a few years, maybe perhaps my fourth or fifth year, I started cooking a bit more at home to prepare my lunches and my dinners. So then I would know how much rubbish food I'm eating in a in a week, I guess. Better gauge then. There are, of course, days where I do feel a bit lazy to prepare. Then, if that's the case, I will try to supplement that with healthier snack options like fruits I guess.. Yeah, that's what I might be an alternative. I don't think I am following all the advices that I give to my patients. No, definitely not. I am not very compliant. But I think I do try. Hmm.
- **NJCL: Hmm, could you elaborate more on what you mean by rubbish foods?**
- P10: Well. Depends. Fried food, I especially love fried food. There are also fast food. A lot of junk food. I think rubbish food in general, something that is not as nutritious as it's recommended. For example, you know breakfast, you are advised to eat a certain amount of- for example, a healthy plate, your advised to eat something like the healthy plate, but I don't. Perhaps I might not. I might miss out on the protein. I might miss out on the vegetables or the fruits. Yeah. If every meal, I have to follow the healthy plate, yeah, I don't do that. Yeah, I can't, or I'm a bit lazy too. Yeah, I think comfort food might be a better expedition of rubbish food and unhealthier options. Cooking method wise, I mean, something that's fried and not steamed. Something that's dipped in or cooked- marinated in a lot of sauces instead of sprinkling it on top. Then I do enjoy going out to eat, especially with my friends, my family. So they might not be the healthier option. I recently went for a symposium that I had. So we do learn that. Apparently over the last 10 plus years, the sodium amount of food has increased by 20% in hawkers and restaurants. So yeah, so eating out might not be the best. But I do love eating out. Yeah. So those might be considered the unhealthier option, I guess.
- **NJCL: I see I see. Then earlier, you also mentioned how last time you used to eat out lot more. So going into that timeframe, how did your lifestyle change after you started to work as a nurse?**
- P10: Okay. I think. Okay. Back when I was in school, I think my weight was what, around 45? When I became a nurse, maybe towards the second or third year. I was my heaviest at 50, yeah. And there was a point in my life where I reached around 52? And that was- It wasn't so much about the weight itself, the amount itself. I think it was the effects it had on me. Sometimes when I gained too much weight. I didn't realize this because I was never obese or or fat- when I gained too much weight, I get very tired easily, first thing. Sometimes even moving around got me a bit breathless. and I feel very heavy, like it is a very odd way to describe. I feel, I can feel the heaviness. Hmm. Well, of course there were other effects, like my clothes couldn’t fit. My uniforms were a little tight. So that was one of the reason why I decided I had to start doing something or changing something. And what I did was perhaps to really cut down on unhealthy food. That's one thing. If I could, I would try to cook especially when I come back home for dinner, because I stay with my brother, so I also wanted to kind of take care of my brother, so that he doesn't eat rubbish food. And then I think I did try to exercise a bit more, so fortunately I do live in a close-by neighborhood to my workplace so I could walk. There was an option to walk. And that's when I started walking to work, which takes me about 20 to half an hour, depending on the speed that I walk to. So yeah. I think- I'm not very sure about hospitals, but being in a polyclinic. There’s less vigorous work, because we are kind of deskbound. You rarely do walking up and down of your workstation so that resulted in me, perhaps snacking in between or being less active than I usually would be. Yeah. And I think also being surrounded by different types of people. I don't really wanna be stereotyping, but in a polyclinic, there’s a lot more mothers. a lot more older nurses. There are young ones, I do have to say, but being in that environment might have affected my food choices as well. There are some nurses who are not health conscious also, in my polyclinic. So our lunch options, when we do eat out, it might, it might vary. How else has nursing affected? I think I'm not sure about the other industries, but I think because I do get tired by the end of the day. That might have been one of the reasons, where I don't- where I'm too exhausted to prepare food for myself, so that might have resulted in eating out. But I guess it could also be me adjusting to the demands of my work.
- **NJCL: then, since you actually mentioned, walking, like physical activity. So specifically on physical activity. How do you feel about your current lifestyle? Do you feel like changing it?**
- P10: Oh, I definitely. I know I have to change it. Do I feel like changing it? Hmm. Ah. think. Why, I say, I know need to change it is because since graduation I've always had on-off shoulder aches. The neck shoulder pain, and it used to be every 2 to 3 months kind of an episode whereby I'll I would have a time where I need to take an MC to rest, or I need really really strong pain killers, or I, yeah, I just need some rest. But it started to get a bit more frequent this year. and I have been having a daily shoulder and neck pain since September? Oh, sorry. I think August or September. Yeah. So I have seen a physiotherapist as well as a specialist, and they both have told me to exercise a bit more, vigorous exercise. I think the only kind of exercise I actually do is walking. I do try to walk at least half an hour, but that's not enough. Do I want to change it? I do want to change it so that I don't have to be in such misery. How am I going to change it? I think I am the kind that needs a bit of company before actually exercising. I don't know how I'm going to change it yet. But I do know I have to change it.
- **NJCL:Then, backtracking a bit. Earlier, you also mentioned something about you're not very compliant to the advice that you give your patients, could you elaborate more on that?**
- P10: I think I've given a lot of examples. For example. when we see patients and talk about- and we're asked by doctors to give diet advice, we would actually go through on a- we'll sit down with them, and it will be like a 20 minutes session, whereby we would ask, What do you actually eat on a daily basis? Then they'll tell you. Oh, I eat, for example, breakfast. I eat 2 slices of bread with butter and sugar. That's the worst example, people usually don’t. But if that's the case, then oh, okay, what kind of bread is it? Is it all white bread? Is it a wholemeal bread. What kind of butter do you take? How much of butter do you put? So there'll be certain advices whereby changing your white bread to wholemeal bread might help you especially because you have diabetes. Low glycemic index. Sugar advisable to cut down. Okay, you might want to stick to unsalted butter, and butter, you shouldn't be putting, you know that thick one slice that you have usually with their kaya toast. That shouldn't be the amount. It should be paper thin. So those are the kind of advice that we give. Yeah, I don't put my butter paper thin. I do add strawberry jam, because that's that's one of my favorite jams as well. My bread isn't whole meal bread, I don't like it. I do enjoy my white Hokkaido milk bread. Yeah. But I try to compromise. Sometimes, if I can, I will choose jams with lower sugar content by reading the nutritional panel information. But I'm not very compliant. Yes. I think one thing I'm grateful for being in this industry is that it has taught me a lot of my life skills that I think general public should have. Things like being able to read nutrition information panel. Not everybody knows how. In fact, even majority of the nurses might not know how and that might be one of the important life skills. I wouldn't say I'm an expert in it. I did go for courses on my own. So yeah, that does somewhat help me. But I wouldn't say I'm very compliant to the advices. I think there are other advices that we give. For example, you know. majority of our older patients actually don't make time to exercise. Okay, not say, majority. Those that I've seen. And we do encourage them to incorporate exercises. For example, if lets say they do take public transport, they take a bus to and fro from work, if that's case, perhaps on the way back home they would walk one bus stop. Yeah, instead of taking the bus all the way home. Those are the kind of advice I give. I don't really follow. Neither do I do that. Yeah. So yeah, that might be another example.
- **NJCL: Hmm, does this non-compliance to your own advice, like. How does this non-compliance affect you personally?**
- P10: Like I mentioned, because of the history of diabetes, the very strong family history of diabetes that I have, it bugs me, it bugs me sometimes. I think it keeps me in check. But it's also a stressful fact- it's also a stress factor. It does give me stress sometimes when I don't, when I realize oh, I've not been eating fruits for the past few days. Then I would have to make it a point to go down, purchase fruits and eat it, and also not leave it to rot in the fridge. Yeah, I think it’s a lot more stressful than it keeping me in check- than a good thing. Yeah. So it's a bit more stressful. A stress factor, I will say. Yeah. Yeah, I think it's because it's coupled with the fact that I am a bit self conscious about me hoping to not have diabetes.
- **NJCL: Okay. Well. For my next question. I feel like you actually touch upon it by some of your sharing. But it's about, as a nurse, how do you feel about self-regulating a healthy lifestyle?**
- P10: The nurses that I've seen, the colleagues that I've had, I think there is a small percentage of whom actually- they do what they preach. Perhaps that might be a 10% or 15%. But the rest they don't really do what they preach. Sorry, your question was? Could you repeat it again?
- **NJCL: As a nurse, how do you feel about self regulating a healthy lifestyle?**
- P10: I think as a nurse and majority also, about 90% or 85% of the nurses are not compliant. I think it's because the majority of the nurses find it difficult, perhaps due to the workload that they have? After a long day dealing with people, I think it does get you a bit tired, so you might want to pick yourself up with the less healthier choices. It's also a bit difficult to make time to exercise, especially when you've had a very, very long day. And I think that's how most patients are. So when we do advice. Okay, I wouldn't say we, but when I do give advice I would try to put myself in their shoes and try to give as little changes as they can, but also changes so that they can do something about their health.
- **NJCL: Hmm.**
- P10: I think it's a bit difficult to be really honest, to self, regulate and be healthy. But it could also be due to the fact that I am not very disciplined, I guess. Yeah. But I think the reason why I wasn't compliant to my own advices, I didn't do what I preached was perhaps, I was a little exhausted from work. I I don't know if that's burnout. But yeah. Just a little tired. When you get home, you just don't wanna talk to anybody. You don't even want anybody to disturb you. And over the weekends, you just need time for yourself to be on your bed. And just, I don't know watch Netflix and do nothing. Yeah, not use your brain, I guess. Yeah.
- **NJCL: Okay. so I'll just briefly summarize what you shared with me. So for the question on as a nurse, how do you feel about self regulating a healthy lifestyle. You answered that it's a bit difficult. Only a very small majority, actually practice what they preach. The rest of the majority don't really find it difficult. So they do it. And then you, you mentioned that because maybe of, like the workload, the long day with people, that there's a lot of fatigue. So you cannot push yourself to actually go and engage into a healthier lifestyle. Then you also mentioned something about like why, you're not compliant. It's also mainly under fatigue that you mentioned. But you try to put yourself in your patient shoes to make as little changes but still have changes for them.**
- P10: Yeah.
- **NJCL: Oh, and you also mentioned about not being as disciplined, personally.**
- P10: Yeah, that might be one of the reasons. I realized my colleagues who do regularly exercise, they try to eat healthier diets. They are, I guess, a bit more disciplined. They make time, despite the fact that they have families they need to take care of. I don't. I only have a sibling because my parents are overseas. But yeah, on top of that. Some mothers have to take care of young infants, young babies, young kids. Yeah. So perhaps the demands of their work- of their life, of their stages in life, might be one of the reasons why they don't comply. But there are mothers. There are mothers with young kids who do regularly go to the gym, exercise. They eat fruits, they make time to cook. Yeah, there are. Not sure how they deal with it. I do know some nurses who do bring their own food all the time for lunch. And that's because they also have a helper who cooks. Yeah, I don't know how they have the discipline. I think majority don't have, I think. I would think so, or majority are actually bogged down by the responsibilities outside of work that yeah, they they don't maybe…
- **NJCL: Quickly elaborate on what you mean by responsibilities outside of work?**
- P10: For example, I do have a nurse who eats bread and fried food almost all the time for lunch, and one of the main reasons why she might not be able to have time to cook on her own is because she's also a mother. she's also a mother to a 2 year old child. She also has family members, her parents, to take care of. She doesn't fully take care of the parents all the time, because she does have siblings who might be able to help. But yeah. Perhaps her responsibility beyond her role, life role as a nurse. as a mother, as a daughter, as a sister. Yeah, that might be one of the reasons why she might not be able to choose a healthier lifestyle, I guess. Yeah. Perhaps that might be one of the reasons, I mean the reason why I, for example, for for myself, I might not be able to choose a healthier diet on certain days, maybe because after I get home I do have to do housework because no one's gonna do. Then after I'm done with housework, I just need to rest. Then I'll be too lazy to cook for the next day. Yeah, so that might be one of the reasons why I eat out. Yeah, I think maybe certain responsibilities outside of my work might be one of the reasons why I do feel, might not allow me to be healthy, perhaps. Yeah. But it sounds a bit like I'm a little lazy, because there are people who do make time. Yeah. So I think it's a struggle on my own as well. To be able to say, to be able to give you a reason why I am leading a rather unhealthy lifestyle. I think it's a mixture. I think.
- **NJCL: It's a mixture of?**
- P10: Is a mixture of maybe lacking discipline. Maybe the responsibilities that I have. Given the life circumstances that I have. Yeah and perhaps just the demands of the job. But I think I'm a bit hesitant to say the demands of the job is because pretty sure my patients who are unable to live healthier lifestyle, they do have the same demands or similar demands that's why they do express that ‘I have no time to exercise’, and I truly understand what they mean. I really don't have time to exercise, and I don't want to make time. I don't really, yeah. Yeah. People say, oh, you know, you could actually exercise, or whatever. No! Over the weekends, what do you mean? I have to clean the house. I have to change my bed sheet. Do the laundry. Yeah. So these things take time. And then I gotta- because I'm staying at home, I might perhaps want to cook. Yeah. So it's just yeah. I guess it's a mixture of reasons.
- **NJCL: I see. Okay. Okay, then, just to go back again to summarize what you mentioned. So it's for you, fatigue and self-discipline, or maybe lack of and the act of actually making time for it. Like prioritizing a healthier lifestyle. And also the responsibilities outside of work.**
- P10: Hmm.
- **NJCL: These are your answers to what you think about self-regulating for a healthier lifestyle.**
- P10: Yeah. Oh, when you did, you mention prioritizing, I think. It's. Yeah, that might be the right word. I do know, at the back of my mind I shouldn't be- for example, if I decide to eat for lunch, fast food, I do know, at the back of my mind. I'm supposed to lead a healthier lifestyle, and that's better for myself also, because of the fact that I do have strong family history of diabetes. But it's also me, oh, I need some reward for myself for having to survive this horrendous week I've had, or the tiring morning I've had or the difficult patients that I've seen. Yeah, I think that's one of the- yeah, so prioritizing might be one of the reasons. I prioritize my well-being, my mental well being, I guess, rather than my actual physical well being. There are days where I do have such struggles. I think even now I do have such struggles. Yeah. And sometimes I do try to compromise instead of maybe if I do eat Mcdonalds, I don't get- I do love my french fries so I would get it. But I will change the drink to perhaps a bottle of water instead of sodas. Yeah. but it's a struggle. but I'm not sure whether other people do feel this struggle. I do feel it. maybe because I am paranoid, that I might have diabetes or other chronic diseases, could I don't want to. Yeah.
- **NJCL: Okay. Hmm. yeah. So you mentioned prioritizing your mental well being instead of your physical well being. Could you elaborate more on that?**
- P10: I think, I'm not sure how applicable it is for me, especially whenever I am at work, every day there will be patients who are difficult. Every day. At least one patient who makes your work a little difficult and intolerable. If it's not patient, it might be management, it might be teammates. Yeah. So I think if there are such annoying events that happen. Especially if it's a few patients, or if it's a huge thing that that annoyed me, or pissed me off to a great extent. I tend to reward myself, perhaps, with bubble tea or something. Yeah, something that I might feel a bit better after eating to give me a mood boost, so that I can, either, last for the rest of the day, or I can recharge and feel better tomorrow. Yeah. Yes. That might be one of the reasons.
- **NJCL: So when you mean like, reward yourself, it's to- is it right for me to say it's like a stress relief kind of thing?**
- P10: Oh, yes, of course. I think, I'm pretty lucky to have nurses or colleagues who are- who do enjoy good food. I would say good food. So they do like to indulge. And that was one of our ways of coping with stress of the work or the demands of the work. One of the important ways. I think I would say, important, personally, important ways to cope with the stress of the work. Yeah. So indulging in comfort food, good food. Yeah, which might not be the healthiest option. I would think it might not be. It's not the healthiest option. Yeah. So that was one of the ways in which we could recharge.
- **NJCL: Hmm. okay, I think, for my next question. You also mentioned here and there the answer, but I'll just ask; what are the what are the main facilitators, or motivators in self-regulating a healthier lifestyle for you?**
- P10: For myself, I think, it's my family background and perhaps my job as well. Because I do see certain patients. I do see patients who are ill every day, who have chronic diseases every day, and they come and complain to you about why it's so bad to have chronic diseases. It is a bit scary at the same time. Yeah, so that might be one of the I wouldn't say facilitators, I'm not very sure whether facilitators might be the right word. Or that might be one of the yeah, perhaps a push factor. Yeah, a scary push factor. Yeah, I do have to let you know that for family history, my brother, my younger brother, actually has diabetes. He was recently diagnosed. So I'm even more self-conscious. And that's- and the fact that I don't want my brother's disease to progress might be one of the most driving factor as to why I would come home despite the fact that I'm exhausted from work. I will still prepare dinner at home, so that he doesn't eat out cause he tends to eat out. He's, he's too tired. Yeah, he's too tired from school and work, because he's juggling both. Yeah, so that might be one of the reasons. Did you also mention barriers? Was there barriers in your question?
- **NJCL: That's going to be my next question. But you can answer already.**
- P10: Let me see, what are the other facilitators? I do have to say that I exercise a bit more when I mentioned, I do need company, right? Yeah, I don't actually like to exercise alone. So when my mom is around because she visits maybe once every few months, we do make time to go walking and it will be an hour, an hour and a half walk depending on how often we go. We try to go every day, if not every, at least 3 times a week. Yeah. So, me having, me needing company- hence, if I do have company, that might be a facilitator. That might help me to motivate me to exercise. I think also one of the main reasons I did change my diet was the fact that I felt a little heavy, I mentioned, right? I was 52? Also I- because I'm paranoid about my health. I do a regular health screening with my GP. So you they would do the full screening. My cholesterol has been a bit high. So my own condition might be also a driving factor. I love butter. But yeah, I do have to change my lifestyle. Yeah, so that might be one of the reasons why. I would try to be healthier. As for the barriers, it's a bit- it's a bit hard to pinpoint what are the reasons. I think perhaps my laziness, my lack of discipline. I don't know whether to call it lack of discipline or laziness because lack of discipline sounds a bit better. It has a positive connotation as compared to laziness, but I think it might be laziness. I'm a little lazy to do anything- sometimes like I plan, oh, okay, since over the weekend I don't have anything. I might walk to Botanic Gardens, which is about a 40 minute walk, I think. 40 minute or an hour walk from my place. But then again, when morning comes, nah. It's a bit too hot. Yeah. So yeah, perhaps my laziness, I guess. What are the other barriers? I think. Well, at least, when I was a nurse, the fact that I had to deal with a lot of people. The demands of my work was a bit exhausting, so I don't want to do anything, yeah, anymore. I don't even wanna cook. I don't even wanna move. Sometimes I would just come home, sit on a sofa still in my uniform until like 7 or 8 plus. And then I would- yeah, cause we end around 5, so I would. Yeah, I would just be there for 3 hours, just scrolling through my Instagram, I guess, doing nothing. Then I would start to clean up, and that's when I realized I don't have time to prepare for my meals the next day. So the cycle will repeat, depending on yeah, how lazy I get. Hmm. That might be one of the barriers, yes, and then what else? What might be the barriers? Yeah, I think the barriers could be the fact that I've had such a terrible morning. Or, I've had such a terrible day today. I've had people pissing me off. Oh, so I deserve some reward. Yeah, I think that might be one of the reasons. Hmm.
- **NJCL: Okay, then I will summarize what you shared for facilitators, slash motivators. For you, the main facilitators or motivators is your family background, your job since you see a lot of patients with chronic diseases. So for you, it’s like a scary push factor, as you mentioned. Then facilitators also include like having company when you exercise, and also your own condition. So you're paranoid about your health so this pushes you. Would you like to add anything else for facilitators?**
- P10: Let me think. Yeah. So, as mentioned, in my family, my brother also forces me to try to be healthy for the sake of- sometimes, despite the fact, I might not be hungry at night, but I do cook so that my brother wouldn't eat rubbish food. That's one of the stressful factor. Because I don't need to eat dinner, I can just skip. Yeah, I don't have to. But I’m putting the effort, on certain days I put in the effort to sacrifice on my 2 hours of no brain time scrolling through Instagram and cook so that my brother, wouldn't eat rubbish food. That's also another push factor, right? So I guess. Yeah. Let's see facilitating. I think it might be a push.
- **NJCL: Yeah, so your family actually facilitates you. Alright. Then, for barriers, for you, laziness or lack of discipline. Then there's also the demands of your work. So it's a bit exhausting which can lead to fatigue. You don't want to do anything else afterwards. You also mentioned about having like a terrible day at work. So in a way, not doing anything is a reward for you. Would you like to add anything else or barriers?**
- P10: Hmm. [Long pause]. I think- okay, it might sound like me complaining, but if we do work a little less in a day. For example, we work 4 hours, I think I might be able to lead a healthier lifestyle. But because we work 8 hours and perhaps more in a hospital, especially if there are delays, might be a little difficult, but I do have friends who are able to regularly go for gyms, or able to lead a healthier lifestyle, despite that fact yeah. But because there were, there were times where I did take half days quite often. So on days where I am on half day, I'm able to make time to exercise. I'm able to make time to cook, make healthier life's choices and my mental well being, I guess, was a bit better. So that might have me resulted in being motivated to get up and and and do something. Yeah. yeah I do have to say, there was a period of burnout during my career. Yeah, so rest and doing nothing. Eating comfort food was very, very important at a certain point. Yeah, it became even more important during then.
- **NJCL: Do you mind elaborating more on this period of burnout that you mentioned?**
- P10: Okay, I don't know what was the cause. I don't really know when it happened. I think the first year of starting work was bit of excitement. I was eager to learn then. Second year, third year, by the time it came I felt- I don't want to sound a bit cocky. Neither was I, a very, very good nurse, but I felt like I've reached a glass- what do you call it? A glass ceiling in terms of my learning. And then, given more responsibilities, I should deal with a lot more. Hmm. Not a nice way to put it, rubbish patients. Yeah. I don't think people were very kind to nurses. Then COVID also happened. Yeah. So that. That was another nightmare of dealing with people. And even now I wouldn't say, people are very nice to nurses, and I think it was just the whole dealing with that, I wasn't able to handle people being rude to me. Yeah. Dealing with the perspective of nurses, I guess, or the demands of my job was a bit exhausting.
- **NJCL: Oh.**
- P10: Sometimes there are days where you do feel like. I think I'm not being paid enough for the work that I'm doing. Yeah. But neither can I afford to leave this job. Where am I going to go with, based on my skill set? Yeah. So I think I've had that burnout period. Or, I think perhaps year 3 or 4. By the time you reach, I think the fifth year, you kind of give up. Give up on making changes- on making changes. When I say making changes, I mean like trying to educate or trying to make people think different. To give us a bit more respect, you give up on that. Just okay. Sure, no problem. Yeah. So that was where I did have- I was a bit tired. I got exhausted every day. Ate a lot more rubbish. Didn’t exercise, gained weight. Yeah. Then it hit a point whereby, oh, I feel too heavy my clothes don’t fit. I think that was one of the main reasons. Yeah, then, and my cholesterol wasn't very, very good. And my GP was nagging all the time, so I had to do something.
- **NJCL: Hmm.**
- P10: I do want to mention, I think one of the facilitator or push factor was the fact that I had a dog. My dog passed away about a year ago, but when I had my dog I had to go for walks at least 15 minutes a day at least, at least, although my dog loves to go for longer walks. So that was one of the main reasons why I had to continue a form of exercise which is still not enough. Not reaching the recommended amount. Yeah, but that was one of the main reasons.
- **NJCL: Like the fact that you had to take care of a dog?**
- P10: Yes and the demands of my dog, the fact that he needs walks. It was one of the stress-giving factors that forced me to take 15 minutes of my day or at least half an hour to make time, to walk with him. That was my form of exercise, at least.
- **NJCL: Okay, going back to what you were mentioning about your period of burnout. When you mentioned on your fifth year, you gave up on making changes, you mean making changes for the patients or like trying to change the patient?**
- P10: Hmm. No, no, no. So when I had such treatments, whereby patients do not- they look down on you. I've always had a goal in life to change that mindset. We're not doctors. Servants, neither are we your servants. We are educated people with a certain amount of knowledge, who are able to give you decent advice. Valid advice. I guess just to try to just change the mindset of people about my job about my role. But I think because you've seen so much by the fourth year. You just give up on that. So okay, if you're going to be nasty to me. Fine, sure. No problem. I think you just yeah brush it. You try to brush it off. I wasn't very good at it. But I I tried. I think I was a lot less affected towards the fifth year. When people treat me with no respect, I think. yeah, I will just rant to my colleagues, and that's it. In the past I think I might have ranted. I might have like gotten really angry. And and perhaps, you know, ranted a bit more. But yeah.
- **NJCL: Okay, then moving on to my next question. How does your self regulation habits impact your work performance?**
- P10: Work performance? I think when I was a bit more healthier, perhaps it would- when I was a bit more healthier. I was able to do what I preach. Hence that might have given patients reassurance that it's possible. I think. I do recall a day where I had my sandwich on my desk, which I had and didn't have a chance to eat. It was for breakfast, and in that sandwich there was lettuce, there was tuna, there was apple. All wrapped up, tomatoes as well, I think, and my patient looked at it, said, oh, this is my breakfast. I've prepared it myself so you could do the same. Yeah, you can add, certain types of vegetables, so you do at least eat 2 servings of fruits and vegetables in a day. Yeah, so I think that might be one way. I'm able to. Yeah, it might be a bit more convincing for patients to believe that oh, I had the similar problems as you and I'm doing this this this to make the change. Yeah. How else has it affected my work? I don't know. I don't think I've been- I'm back to the most healthier self in order to see the health benefits. But I've lost weight. I'm back to my original 46 plus, yes, I have a bit more energy to do things. How it would affect work, I’m not sure, but it has affected- it has changed my life I guess.
- **NJCL: Increasing the scope, maybe not just work. How does your self-regulation habits impact your work and your life?**
- P10: Work, I really don’t know how else. But my life? I think my clothes fit better, although there are some that are still small. That's a bit more happier news for me. I do have a bit more energy to do things. And as I mentioned, I always struggle with the thought that I do have to eat healthily. But I do want to take comfort in the food that I want to eat, the rubbish food that I want to eat. So I struggled a little less. Yeah. So it's like this nagging feeling. You need to eat healthily. But yeah, I struggled a little less because I am trying to eat a bit more healthily. Hmm. Yeah, I think that's pretty much it. Because I haven't done the other component of changes that I wanna do. Which is the physical activity yet. Well, once I do, I hope I wouldn't have the shoulder pain. Because it's on a daily basis for the past 2 months. And it's it's affecting my well-being. Yeah, so hopefully. I do feel less affected by it. Yep.
- **NJCL: Sorry, so just to clarify the reason you haven't done the changes you want is mostly time?**
- P10: Yeah, I guess, laziness as well as me adjusting to my new work, and also the fact that I would prefer to have somebody to exercise with instead of exercising by myself. And I haven't found that somebody. Yeah, my friends are busy. My parents are not around here, so. But I still gotta do make the changes. But I'm not really motivated. But yeah, I should do it because this pain that I have is affecting not only my work, it's also affecting my mood. My well-being. In general. It’s affecting my sleep as well. I have sleep problems because it's painful, and there's no medication that works. I've tried like, panadol, anarex, diclofenac. Yeah, nothing works. Yeah. So yeah, so that's a huge problem that I need to settle. I know I have to settle. Yeah. And also the fact that my specialty's appointment bills or the physiotherapy bills are quite a bit. So I better settle this. So yeah, hopefully. within the next month I would start something. I don't know what yet
- **NJCL: You mentioned. You're not really motivated. Can you elaborate like, what do you think will motivate you?**
- P10: I think I need… Oh, yeah, okay. I think I need a bit more motivation than the pain I have. Maybe because I don't like exercising in general, and if it's a bit more fun if I have someone else to do it with. But neither am I willing to join, like, you know, running groups or any exercise groups, because I don't fancy all the exercises except walking. Yeah. So maybe because I'm also a bit picky. I would like to have someone who is able to do the same kind of intensity of exercise in and also the kind that I might enjoy. I would prefer. If you know. walking- wise, I don't want to just walk on a machine. I would rather, I don't know walk in garden. Walk around the city so that I can at least see things. Maybe because I have such specific preference. I'm not motivated. Yeah.
- **NJCL: Hmm.**
- P10: But, the fact that I am suffering from this everyday makes me want to change.
- **NJCL: And, hmm, then all right. then, for my next series of questions it will be regarding the workplace. So as a nurse, how do you think the workplace influences your own self-regulation habits. or lifestyle habits?**
- P10: Oh, I don't know how long has it been, but I think the last couple of years my institution had, had a comparison of staff health. So when we do staff health screening, I think, every 2 years- it wasn't an annual basis, but every 2 years we would put up, oh this clinic had very healthy staff. This clinic has, you know, full of obese staff. Or, yeah, this clinic, the cholesterol levels are all haywire. This clinic is doing well. Yeah. So then they started. I can't remember how frequent, but I think it might have been a monthly or bi monthly exercise space and not really exercise- they had a team gathering-cum-exercise days, so they would organize events to motivate staff to exercise. That might have been one of the reasons I did attend if it was an activity that I liked. For example, hiking, yeah, or walking. Yeah, that I did attend. But to be really honest it is once a month, or once every 2 months, so it's not as effective. But my clinic also had a nurse who recently got her yoga instructor license. So she did teach Yoga. I honestly have no idea how often it was, but I think at least once a month, or twice a month. Yeah, I didn’t attend that class, because I don't fancy yoga but I think if you do have colleagues who are a bit more self conscious, a bit more health conscious, sorry health conscious, you might be more motivated, I guess. Yeah. How else has the workplace motivated you? I think it's only those activities or the colleagues themselves that motivates you. I do have a close group of nurses who I hang out with. We do meet outside of work. So they are really, really close friends. They're not nurses anymore. They're friends. Yeah. So sometimes, well, it hasn't been very often. But we do go for walks, and we will walk for like an hour plus, and then we'll indulge in food. Yeah. But at least we exercised, yeah. So we do that, but I wouldn't say it's a regular routine. We have done it twice the past one year. So yeah. But I think these are a group of people, if I do say, ‘hey, let's make time to go and exercise’, they might say yes. and I do need this kind of people, I guess, for myself to motivate myself especially. Other than that…? I do know there are initiatives to give- to walk the talk. For example, for caterers, we are supposed to choose a group of caterers who gives a healthier choice options. Our snacks are often given as fruits. Then we do have- I think there was an initiative whereby there's a fruit basket in in the clinic. So our- any staff they're allowed to take the fruits and enjoy it for lunch. That's another initiative. What else? Yeah, I think that's pretty much it. Well other than facilitating, I think one of the main reasons why nurses do eat out, especially in my clinic, was the fact that they didn't have any food choices nearby. Will you be disclosing the place that I work, if I name it?
- **NJCL: No, I will censor it all.**
- P10: The [Public healthcare institution name] polyclinic, there's nothing nearby. Oh, well, there is a Kopitiam nearby. If you do search this certain Kopitiam, it is about 8 to 10 minutes walk, there's also another Kopitiam nearby. But they don't sell healthy food. It's a Malay stall. They do have very, very nice nasi padang. but it's very, very oily I do have to say. Sometimes when I eat it, I will just tell my colleagues I feel like I'm having- I can feel my heart choking. But so yeah, so like, yeah, I can feel my fat depositing in my, arteries. Yeah, so that's one of the main downfall, I guess. Well, nearby there's also a university, a private university. So that's also a food option because the canteen. But the food is terrible. In my opinion, it's terrible. So that's why my nurses tend to order in. They tend to indulge in. Yeah. That might be one of the factors why we might not eat healthily? I guess. Might be, might be. Well, that's for me, at least it was a reason. There's nothing nearby. So, might as well I just order in, and if I do decide to order in, since I'm going to spend the money, eat what I want. Yeah, eat what makes me happy. Yeah. I do know. There are a few polyclinics that have food places nearby or within the clinic itself. Yeah, within the clinic they do have food that they can buy.. Yes, I think that might be one of the main reasons. How else is the workplace? I can't think of any other. Yeah.
- **NJCL: It was quite comprehensive. Yeah, it actually touch upon, like, some of the- my follow up questions. Like, for example, like, how do you feel about the workplace culture among nurses in helping to facilitate or hinder self regulation of healthy lifestyles. So I think. like for that question you did mention your colleagues, so I would think that your colleagues are facilitators, a push-factor to self-regulating a healthier lifestyle.**
- P10: I think it’s a facilitator, not a push factor. So if I do decide to organize a walking event, I do know I might have company and that’s a motivating factor for me, yeah. Despite the fact that there are colleagues- not only nurses, there are doctors who regularly exercise. That doesn't really motivate me. I do admire them. [Participant laughs here]. But they don’t really motivate me. My colleagues too. Yes, I think that’s a small motivational aspect of the workplace.
- **NJCL: Hmm. Would you say, like the workplace culture among nurses in your workplace, encourages or discourages more healthier lifestyles?**
- P10: I think if there- okay, I would assume when you say that it's a top down approach right? Whereby institution decides to organize certain things. For example, the healthier caterer thing?
- **NJCL: I think I'm more interested in, like the relationship of the nurses, either like the same rank, or even like managers, how this can and motivate or demotivate someone into getting healthier?**
- P10: Okay. No, I don't think so. I don't think they do. Because majority of time, I'm not sure about the younger nurses. But well, at least, when I was in a clinical setting a lot of nurses actually don't come to work to find friends. That's one of the typical yeah, typical thing that they might say. I'm here to work. I'm not here to make friends. Yeah, so I don't think- unless you are friends, you meet them outside of work. I don't think they will. Yeah, they have an impact. Yeah. And unfortunately.
- **NJCL: So they don't really motivate or demotivate you to be healthier, right? The culture among nurses?**
- P10: Yeah, I don't think so. If you were to say demotivate, depending on the type of culture that you're in, if that culture is a bit toxic or stressful for you. I guess that might be a demotivator for you to be healthy, so you might indulge in- well, if you do indulge in food when you're stressed, I guess that might be one of the reasons that you are demotivated. For example, for our case, for my case. My nurses do like to indulge in food, they will say, oh, reward yourself! But every day is a reward day. So that's how they reward themselves. So. I wouldn't say it's a huge demotivator. But I do have to say sometimes, despite the fact that I brought healthy food. I'll be like, okay, I'll buy something that's a bit unhealthy. Yeah, I might indulge in nuggets today. So on top of the food that I have. I might, yeah get nuggets, but I don't think it's such. It has such a huge influence on me. I do say no, most of the time. Yeah, if I decide to be… But there are days. They might be an influencing factor, I guess.
- **NJCL: Okay, then, what do you think about the role of nurse managers in helping nurses self regulate their own health?**
- P10: Hmm. [Long pause]. Can you give me an example? I don't know whether you've had other participants who mentioned this or or yeah, what do you mean by nurse manages? How? How do they?
- **NJCL: By nurse managers, I mean the Brothers and Sisters in the ward?**
- P10: Yeah, how would they usually, help with that? What are some of their answers?
- **NJCL: I think, for my past interviews. It's a bit mixed. Some of them feel that they have minimal impact on the nurses on the ground. But there are also some that feel they can actually help a lot in making sure the nurses on the ground are a lot more healthier. So it's a bit mixed.**
- P10: Hmm. Personally, my sisters have had no influence, no influence. I don't think- okay, you might not- I might not be in a position to say this, but I don't think that they actually thought of that as even part of their role.
- **NJCL: Hmm.**
- P10: Yeah, I don’t think so from the way they behave? But if you ask me, would managers make a difference? If I was a manager, would I take that as a priority? I think that I might? I might, I might take it as a priority, perhaps to initiate things. And yeah. But I think in order for us to do that, you do have to be a bit more than colleagues to have an influence. I think, I think you do have to be a bit more than colleagues in order for you to be able to influence their lifestyle.
- **NJCL: Sorry when you mentioned that the sisters in your institution, they don't even consider it as a part of their role. Would you say this is a unique thing in polyclinic setting compared to a hospital setting, from what you hear personally?**
- P10: No, I don't think- okay, I don't think the sisters in general care about staffs’ health. Yeah. I mean, okay, with the talk of mental well-being of your staff, maybe they might be concerned. But physical health? I don't think so. From what I've seen, I think, no. That's not even part of their job. They don't even see it as part of their job scope or their priority. It doesn't even make it into the list, I think. Yeah, they’ll be like- I think there'll be like it's, your health, your responsibility. Why should I care? I think that might be
- **NJCL: Then would you agree with this mindset like it’s your health, your responsibility?**
- P10: Okay, when you asked me that, meaning if I am a senior nurse manager?
- **NJCL: Yeah, because earlier you did mention that if you were a nurse manager, you might initiate some things.**
- P10: If I was a nurse manager within the clinical setting that I was in my institution, I think I might make it a priority, because I think I am a bit closer to my- my colleagues were my friends. Yeah, I do think our relationship was a bit more than colleagues. Was a bit more than hi-bye kind of people. So I might have taken some initiative to make a change. Because I think maybe, because I do care about this people. I do care about their well-being and if they're not able to do it on their own, if they're not able to make the changes on their own, then I can help them. I am willing to help them if they're willing to join. I guess my initiatives. But most probably I might do it for myself. Rather than, yeah- I would do it because I care about this people. But I will do it also because I do need to do it for myself. I do need to be a bit healthier for myself. Yeah. So yeah. That's why I said I might have taken initiatives to change certain things.
- **NJCL: So when you mean you do it for yourself, it's because it's your own responsibility. Would you say that?**
- P10: No. Okay, when I mentioned the responsibility part right? I think most- what I meant was that most managers don't really care whether you're doing well or not, like healthwise. They don't care whether you have diabetes or not. I think they don't care. I mean, so many people have diabetes. They don't care whether you have cholesterol or not. They don't care what kind of chronic disease you have. Yeah. I mean, it's your health. You gotta deal with it yourself. I don't think they make it a priority, oh, because my juniors or my senior nurses in the clinic have this this this condition, I should try my very best to change this this this for them. No, I don't think, I don't think so. I don't think they have that concept. They would think of it as: it’s your own problem, it’s your own health. You should take care of it yourself. I think you will rarely find this kind of people who are actually, I care about this people this much in order for me to do something about it within my authority. No, I don't think so. And that's- I don't think that's their priority as well. They've got a lot more to be worried about. Yeah.
- **NJCL: Hmm. hmm. Alright. Alright. Then, moving on to my next question. I think you already mentioned a few examples, but the question is, how do you think your institution can help you to self-regulate a healthier lifestyle? So I think you mentioned that they organize like some activities, but it's not as frequent. Then they even have the fruits basket where nurses can get fruits for their lunch. Is there anything else that the institution can do to help you maintain or self-regulate your lifestyle?**
- P10: Hmm. Institution can help me… Well, I think one option could be. We could have, within the clinic, we could have restaurants or food places. It might be a bit healthier, and perhaps not too inedible food. Yeah, there might be one way. But these are so difficult to do sometimes. Yeah, like, okay. But yeah, if institution can, that might be one way. Another way. Let me think. I think the reason why the exercise-cum-team building activities were held so sporadically is also because of the fact that on those days, we could only do these activities after work. Yeah, majority of the time is after work or over the weekends. Hmm. Majority of time, people don't really want to join on weekends because they want to spend time with their family. As for the after work part, it might be a bit difficult to organize, because depending on the patient load itself- because it's a teambuilding. As mentioned, there are other people who are joining; pharmacists, doctors,nurses, allied health. They have to end at a specific time. Yeah. In order for them to make it for the activity. But if there are any delays in between, then you might not be able to make it. Yeah, so exactly. I think the reason why many people don't attend this initiatives could also be because it's a bit difficult to. Yeah, but not- but yeah- it's a bit difficult, given the fact that they have work to do. But if let's say, organizations were to declare one day of rest to exercise. Hmm! Would that really work? I'm not very sure. Well, another option could be, perhaps, they offer gym memberships, I guess. I do know, in the hospitals. There are gyms that they can have access to for nurses. Polyclinics, I don't think they have. Yeah, I'm not very sure. So maybe a membership somewhere. But then again, would I go? I don't think so. So yeah, I don't know how else they could help. And yeah. Perhaps the most practical or the most relevant way, within the polyclinic, we have some food areas that are available. It could be like, in house kitchen. whereby we have variety of food like like a buffet. Not really a buffet. Yeah, like, like, you know, school food when they give you. Overseas school food, I guess, because Singapore food is a canteen, yeah. Overseas school food whereby they give you specific things on a daily basis. Yeah. Maybe that might work. I don't know.
- **NJCL: Does your workplace have any workplace health promotion programs. I mean, other than infrequent activities that you mentioned?**
- P10: Workplace health promotion programs? I think it's not specific to my institution, cluster of polyclinics itself, the institution itself have put in place, yeah, those that I mentioned. The team building-cum-exercise thing. I think it's once a month. And then they have the healthier food caterers, if I'm not wrong. The list of it where you're supposed to choose from there. And not sure who initiated the Yoga session, perhaps my clinic? I don't think it was an institution thing. Yeah. So perhaps my clinic organized it? I do see quite a bit of them attend. Maybe- I mean our clinic has around like 80 to 100 staff. But maybe like about 10 of them attend. Yeah. Other initiatives specific to health promotion? Well, they've started the- it's like a report card. So every time we do staff health screening, they tell you. Oh,this clinic, a lot of you guys are, your BMI’s are too high. This clinic? Yeah, your BMI are all normal. This clinic, cholesterol is all good now. So that report card might be an initiative, I guess. Because it wasn't like that, the first 2 years of my clinical practice. Yeah. So once they started that- But I don't think that was a huge motivator. Yeah. But yeah. Other than that, have I seen any impactfull ones? No, I think only the exercise initiative that once a month thing might be one of the things that I might have taken part in, simply because of my own needs, like I need company in order for me to exercise. Yeah. And the kind of exercises I pick are very, very specific. So I've only joined those kind of events.
- **NJCL: So would you say, the rest of the initiatives are ineffective?**
- P10: And no, I don't think they are ineffective. I think perhaps it might not have given a more desired outcome. The fruit basket did help for majority of the people in the clinic who do not bring fruits, I think. Oftentimes it was actually depleting quite fast. I think they do top up. Yeah, so it will deplete quite fast. Oh, but I wouldn't say they have been very successful. Yeah.
- **NJCL: Hmm. Then, in your opinion, how do you think this could have been more successful?**
- P10: Hmm.
- **NJCL: So like, what do you think might be affecting the success rate?**
- P10: I would think what's affecting the success rate could be the same reasons for everybody. Similar reasons. I guess. The similar reasons as to why my personal- personally I'm not able to maintain healthier lifestyle. Maybe the demands of the work, the demands of outside of work. Oh. Yeah. Your health being less of a priority than other things that you have to deal with. For example, you know family wise. If you do have family commitments that might be a priority rather than exercising. Rather than yeah, eating healthily or or maintaining your weight. Yeah. So I think, thinking about why people might not indulging- why nurses might not generally, or what are the barriers of why nurses do not lead a healthier lifestyle might be one of the methods in resolving in in a top down approach on one of the methods in which the organization could think of solutions. That being said, I think the most, most successful option might be to actually have in-house canteen, I guess. or in house, yeah. You know how like, I don't know if you know, like the YG company? K-pop?
- **NJCL: Oh, I don’t.**
- P10: Oh, so in Korea, in Korean culture right, majority of the time if you do go to school, you carry a- what do you call it? A metal plate. So they will give you the food. So today, it's this this this. So you take how much you need. Yeah. So those kind of thing might perhaps be effective. But it's also like, you know. People might not like it. Different cultures. They might not prefer it. Food might not be as nice. So yeah, there, there might be barriers to this solution, but I think that might be an option, or at least have a have a canteen within the clinic itself. As well, bring in companies who might be able to give healthier option food. Yeah, there, there might be one way, I guess. I honestly have no idea how effective that might be given the fact that my nurses do love to indulge. Yeah.
- **NJCL: So this is one of the things you mentioned earlier, you said like health is less of a priority for some nurses. So what you mean by this is that they prioritise something else other than their own health?**
- P10: Yes. For example, I have a nurse who do enjoy fried food. She'll eat it every day, every meal. The reason why I think she enjoys it is because it's convenient to cook. It's frozen. So you gotta just put in the on the pan or air frier. Yeah. And why she might need convenient food is. Perhaps she has a 2 year old kid. She just has no time to prepare. Cut vegetables, cook the vegetables. So you forego, your health in order for you to have more time to do something that's more important to you. I guess there might be one reason. I think some people just don't prioritize health. Like they don't think- there's this saying like whereby a lot of people say, oh, would you rather be healthy and eat terrible food, or would you rather be unhealthy and be happy eating good food? So there are people who prioritize different things, I guess.
- **NJCL: Hmm. Then, in your opinion. Why do some people prioritise their health more than others?**
- P10: Hmm. Why do some people prioritise? [Long pause here]. Initially I thought it was a lack of knowledge. I prioritize health, I think more than my brother does. My brother doesn't. He doesn't care whether he eats too many sweet things or not. He will eat whatever he wants. And I will nag at him. Initially. I thought it was a lack of knowledge. But I think it might not be lack of knowledge. Not quite sure why they don't though. Perhaps they don't feel the need which I don't understand why. Because you're gonna live with this body for the rest of your life. Might as well take care of it so that you can at least use it for longer. Yeah, I don't know why. It puzzles me.
- **NJCL: Here. Okay, then, just my final question, it’s actually just to rehash what you've been mentioning like, outside of work, what do you think influences your lifestyle? You did mention there are like demands outside of work. Yeah, could you just clarify some of this?**
- P10: Your question was outside of work?
- **NJCL: Outside of work, what influences your lifestyle?**
- P10: Mmm. Yeah. So the demands outside of work, for example, house work is one of them. Housework, the need to do housework. Housework might be one of the reasons why I might not do things that are better for my health, like preparing food for myself, preparing food for the next day. Yeah, that might. That might be something. I guess if I do have family members to take care of, the most convenient way to settle meals would be to eat out. Yeah. Let me think. I think that might be the reason. Hmm. Your family status, your other responsibilities beyond work could limit- I mean, it might not limit for other people as well. I do know of people who are not affected by all this, despite the fact that they have like a thousand and one things to do outside of work. Yeah.
- **NJCL: Then I'll just summarize. For you, the outside of or work that influences outside of your lifestyle are the demands- sorry the demands outside the work include, like housework, family members that you have to take care of, like all your other responsibilities will limit your ability to change your lifestyle. You also mentioned family status. Could you elaborate how this influences your lifestyle?**
- P10: Yeah, for example, if not for me personally. But if I had a parent, an elderly parent, a sick parent, I had to take care of. I think I might not be able to make time to cook, especially if they do need a lot more caregiving. Hmm, yeah, if they do need a lot more caregiving. That might take a lot of my time. So I might depend on other ways of eating. Settling my meals. That might also take a lot of my time, so I might not be able to exercise. I would think so. I do have colleagues, who are like that. That might affect certain things. So because of the yeah, the family demands, the family situations that you might have. Not only family, you know, I mean, yeah, the demands of outside, the responsibilities that you have outside of work beyond the fact that you are actually a nurse, you do have you have other roles to fulfil in life. Like so that that might be one reason
- **NJCL: And so it's like having to take care of others outside of work is a major factor? Hmm.**
- P10: I would think so.
- **NJCL: You think you have anything else to add for this part?**
- P10: Hmm, no, I don't think so.
- **NJCL: Alright. And okay. Then, do you have anything else that you would like to speak about that was not mentioned in the interview? Any further comments you want to share?**
- P10: No, I don't.
- **NJCL: Alright, then. Okay, thank you so much. This marks the end of the interview. Thank you for your time and participation.**
- P10: Most welcome.
- **NJCL: Very comprehensive, thank you.**

P10: Oh, is it? Okay.

-End of P10 Transcript-

# **Interview #11**

Participant and interview details
 Participant number: 11 (P11)
 Date of interview: 28 Sept 2023
 Interviewer Number: Navarro John Christopher Lambino (NJCL)
 Time IDI started: 1600
 Time IDI ended: 1700
 Observational notes: Participant seems like she has just woken up.

Section I: Interviewer’s summary
 The individual interview was conducted via Zoom. P11 expressed that it was her post-night shift and her voice was hoarse, indicating she might have just woken up. P11 is a relatively young nurse, with only around 1 year of work experience. Her responses expressed the experiences of how much stressful the first 4-6 months were. P11 is also not as active as some of the previous participants, citing fatigue and laziness. Although she also expressed the fact that she prioritized “mental health over exercise”. Not overthinking about work and removing the feeling of guilt regarding work is also her main motivator, hence to be the best version of herself to give the best care was an idea present. Manpower issues, and feeling bad after a bad of shift work was also expressed. She also mentioned prioritising patients’ health over her own health. Current institution measures were deemed not ineffective as it was not currently applicable for her. She mentioned that perhaps only after her health worsens, will she feel the need to change.

Section II: Transcript

- P11: Oh, it’s been so long since I used Zoom.
- **NJCL: Good afternoon, miss. Firstly, I would like to thank you for taking the time to speak with me today. My name is Chris, and I'm a NUS Year 4 nursing student. The aim of this interview is to get a better understanding of the perspectives nurses have on self regulating their health behaviors and how various factors may influence this. I want to find out what are your views, opinions, and experiences on this. I would really like to encourage you to speak what comes to mind. There are no right or wrong answers. May I have your permission to continue video recording this interview session?**
- P11: Sure sure.
- **NJCL: Okay, thank you. Then let us start off with some basic questions like, which ward are you working in right now?**
- P11: Okay, so I'm working at- no need to censor anything right?
- **NJCL: Even if you mentioned, I will censor it.**
- P11: Yeah. Okay, I work at [Public Hospital name], at a geri gen-med, C class ward. So we take care of like up to 12 to 14 patients per team. Yeah, that's the ward.
- **NJCL: Could you describe like your role and what you do most of the time?**
- P11: Most of the time, I think, when it comes to Geri patients, it's like, yeah, there's active management, but it's mostly like more of comfort, or do not resuscitate kind of cases. Yeah. So mostly, we help a lot with their ADLs. Yeah. So yeah, a lot of feeding patients. Yeah. A lot of changing diapers, everything. Yeah. And a lot of them need assistance to like sit down and do more physical things. Yeah, not just medical care. So I think when it comes to Geri, it’s a balance ah. Yeah, the good thing is, we don't really have many like CPR cases and all that. So it's a bit less stressful and less changes because the consultant is not as messy, as in the normal gen-med or gen-surg. But yeah. It’s more holistic care because we want the patient to discharge and have a long term plan.
- **NJCL: How would you describe the workload?**
- P11: Workload? I would say, I think it's a bit like a normal medical ward. There are days it's good, and there are days when it's bad. But when it’s good, it's like, let's say, not a lot of NGTs. Most patients are like, they know where they are. They're not dementia patients, you know. They can walk around their own. But if it's bad, Yeah, I would say, like, maybe, like last month it was bad? It was like out of 14 patients, There was like 4 NGTs. And most of them are bedbound. So we have to do trolley bathing. There was also had active case- that just this Tuesday had to bring him to ICU. Yeah, so I would say the workload is heavy, both for the staff nurse in charge, who is like serving medications because we still have antibiotics to give, but also for the junior, who has to like shower patient, has to feed them. And then do NGT feedings because it's all like around the similar timings. Yeah. So I would say, on good days is good on bad days it’s really bad, especially now when the staffing everywhere is not enough. Yeah.
- **NJCL: Can you. Tell me, like, what is the nurse to patient ratio?**
- P11: Okay, before COVID- and I heard a lot from my ward, it used to be like 3 is to 14 or 3 is to 12? Yeah, now it's 2 is to 14 or 2 is to 12. Yeah, even sometimes, normally like the whole ward- because we got 3 teams. So it's like 36 to 38 patients, I don't know the math. Anyway, it’s around there. So there's around 6 people on shift, plus one sister, plus someone who will help serve the diet and do basic stuff. BCA, yeah. Like, if it's very bad, then there'll be like 5 staff. Yeah. So basically, the in charge will still take 12 to 14 patients. But the junior would take about 16 to 18. Yeah, the good thing about geri is that because it’s so physically heavy, so normally, the staff nurse in charge will also help with the junior things. So I would say, there’s more of like a teamwork, because we know we can't survive. Yeah, we can't go home on time if we don't help each other. Yeah.
- **NJCL: I see. Okay, thank you for that. My next few set of questions will be regarding lifestyle. So as a nurse, how would you define a healthy lifestyle?**
- P11: Okay, wow. Healthy lifestyle? Like physically or like?
- **NJCL: Yeah, like physically, mentally, emotionally like, yeah. Anything that comes to mind when you hear the words: ‘healthy lifestyle’.**
- P11: I think it’s like any normal person like sleeping enough. Eating enough. Exercising on and off. Yeah. I would say, that’s the most healthy lifestyle. For us it’s where, if we can eat on time. For us that is quite healthy ah. and then, if we managed to sleep enough, yeah, between shifts to me that's healthy enough, I guess. Mentally I would say that it's probably the same as any other job; having a work-life balance. And the good thing about nursing is that sometimes when I go home, I don't have to think about my patients, even though I'm stressed out about it. But at least I can rely on my friends, and the most they will text me for any follow ups that. So I would say, that's the healthy lifestyle and healthy mindset for me.
- **NJCL: Earlier you mentioned like being able to eat on time? Would you elaborate more on that?**
- P11: Yeah, I think it's in most of the nursing C class wards. It's like, no time to eat. Yeah, so normally, If I can go for a break for like 5 to 10 minutes. It’s like enough for me to like eat something like a biscuit or like drink a milo. Normally, I'll just go for toilet breaks. Yeah, because for geri patients, they are very high fall-risk. There's a lot of falls, and unfortunately the consequences is very high, and the good thing about like my ward that I- so far like we are- we always sacrifice going to breaks. We just stand there so it’s safer for patient care, especially like most of the falls we had like there wasn't really a fracture that. Yeah. Then, when we do scans it’s fine. So I'll say, Yeah, we don't really eat a lot, especially for morning shift, for afternoon shift and night shift it’s like much better. Yeah.
- **NJCL: Hmm. Just know, you said, like a good thing about the ward is like- when you said good thing about the ward you mean, like, good thing for the patient, or?**
- P11: A good thing for the patients, ah. I mean sure we have to put ourselves first, but if putting ourselves first makes the patient injured, or like in the end something will happen. I rather not lah, even though even though we always jokes about it, like ‘aiyah, just leave the patient alone lah, don’t care lah.’. But in the end, we won't do it, because, you know, it's like the, the not right choice. Does it make sense? Yeah.
- **NJCL: So is it right for me to say that your priority is on the patient instead of yourself, is that correct?**
- P11: Yeah. If I have to take care of like patients who can’t even like feed themselves, you know, all like sometimes they forget where they are, so they'll start. They start like trying to find their you know, their children are calling for them, you know, crying being drama. And it's like they are gonna climb out. And they're gonna like- worst case scenario they are going to fall down like, yeah. Then they wouldn't even know they fall down. Then in the end, like the family of patients were- like the patient will suffer and then the family will be angry, you know that kind of thing? So it's like I'd rather avoid it. Yes. Right.
- **NJCL: Like, rather avoid confrontation-**
- P11: I would rather avoid the worst case scenario, because, you know. There’s probably a lot of stories in the news also lah. Yeah. You know, the ahma own self shift onto the chair and then kena fracture. You know that kind of thing. Like definitely, the hospital will protect you because you have documentations and everything but the guilt will be so much higher for the nurse himself, because you are the one that caused the fracture. Like, can you imagine if it was your own grandma? Like you go hospital, then you kena fracture. Then you have more problem. It’s like confirm you will rage lah, honestly. So like, I rather treat them like how I would treat my own grandparents, that kind of feels.
- **NJCL: See. Okay, then. Specifically, diet wise, how do you feel about your current lifestyle?**
- P11: Hmm. Diet wise, I would say, out of shift it is okay. But during shift, it’s like, not really good. Yeah. Just normally will go for like fast food or like cai fan- cai fan is fine lah, but like, it’s still oily and stuff instead of bringing your home cooked food. And every time when it's night shifts like you start to crave all the potato chips, you know, all that kind of thing. I would say, I've been trying my best not to snack so much during shift, anyway, like also no time to snack, right. Yeah. So I would say, outside of shift, like my family will like cook food. Or I'll eat outside food, you know, at a regular timing, but like during shifts like, I'll just eat whatever is available. Yeah. And normally, nowadays, I don't really eat during night shift also because every time it's like, no matter how much I eat during night shift, I will still want to eat post-night shift. Yeah, regardless if I eat little, or if I ate more. So, I just don’t eat la. I just drink a cup of milo. Does that answer your question? I have no idea.
- **NJCL: Yeah. So when you say you are trying your best not to snack, you are actively trying to change your current dietary habits?**
- P11: My initial dietary habits. Yeah, when I wasn't used to like shift work and all that. Yeah, because you know if you- last time I used to school like you right, like we would eat a lot, we would eat breakfast, lunch dinner. Sometimes even go supper. But like now is so different, so I guess I had to change my eating habits according to my shift. So at least I think now my body kind of knows like for morning shift, I'll just like I won't eat until like 3 or 4 'clock, and maybe I'll just see a biscuit in between. Everybody knows ah. Then if it’s PM shift, I'll eat a early lunch and early dinner or for night shift, I’ll just eat a heavy dinner before going for night shift, and then eat a heavy breakfast. Yeah. It’s kind of like having a routine really helps. I guess, in coping. Yeah, I would say. Yeah.
- **NJCL: Can you elaborate what you mean by coping with… work?**
- P11: Coping with the fact I won't be able to eat at normal timings. But I still want to eat, you know that kind of thing? Like, my body still needs the sustenance. Yeah. So I would say, I have to- and a lot of stuff have to work around our shift timings. For us to like, eat properly. Yeah. So it is a challenge. But I think each of us have our own eating habits. Yeah, to try and cut, although I know I know people who have very bad habits like, there's a staff who like has IBS. So he always eats whatever he wants to, then he go toilet for damn long. It’s like, you’re not really taking care of your own body. Yeah. But I'll say, whatever lah. Whatever helps the person not to be so stressed, yeah I would say it’s fine. Yeah, probably.
- **NJCL: Actually, you touched upon it a bit. But can you elaborate more on how lifestyle actually changed after you started to work as a nurse?**
- P11: Wah, a lot sia. I think you'll relate when you start work depending on the ward you go. Like, certain wards have different. I'll say, different practices, for instance, like ICU, they must always go for that one break. But any other time that they would die lah, yeah. So for us- sorry what’s your question again? I forgot.
- **NJCL: How did your lifestyle change after you started to work as a nurse?**
- P11: I would say my eating timings have changed a lot. Like normally, we would lunch at like, 1'clock, and dinner at like 7? Yeah. But when it comes to shift work, everything kind of shifts around. I don't really eat breakfast any more, I will just eat- Oh, so yeah, it just depends on the shift. I feel. Yeah. But I would eat at like odd timings. For instance right, for morning shift, I won’t eat breakfast, because I will have to go to the toilet to pang sai. So in the end I will eat, probably at like 3'clock, 4'clock. And then I’ll eat another dinner, that kind of thing. Yeah. Yeah, that's for the eating wise, then, for like. Even for like going out, everything has to revolve around my shifts. The good thing about it is that most of my friends are like nursing friends. Yeah. But when I have to go out with normal people, right? I only have to- I only can go out during my off days or my post-night shifts, or like after morning shifts, or even before PM shifts, if I have to go for breakfast like it has to be like 8 or 9 o'clock. Because I have to go to work by like 12, right? Yeah, so I would say my lifestyle has changed a lot. But I would say it’s still manageable. I would say if you really really want to meet all your friends, have enough sleep, it’s very hard. But ya, it’s okay. For instance, like today, I really wanted to meet my friend. But post-night, I just came to her house to sleep ah. Yeah, it’s that kind of sacrifices I would say? Yeah. to try and maintain a normal life, I would say, yeah.
- **NJCL: I hope this interview didn't contribute to your sacrifice.**
- P11: [Participant laughs here]. No lah, no lah. It’s fine. I did Honours year as well, so I know how difficult it is. Like, I hated that year, I wish I didn’t go for it. I mean, I don’t know how you feel about it honestly. Do you like it?
- **NJCL: No comment.**
- P11: [Participant laughs here]. Who’s your mentor?
- **NJCL: Dr. JC. I’m not sure if you know her?**
- P11: Dr who? No, I don’t know. She’s okay ah?
- **NJCL: Yeah, she’s okay.**
- P11: If your mentor is good, then it’s quite okay ah.
- **NJCL: I see, I see.**
- P11: Okay, all the best!
- **NJCL: Then, we talked about dietary habits and all this? Then, specifically, like physical activity wise, how do you feel about your current lifestyle?**
- P11: Hmm, like exercising is it? I honestly, I don't go for jogs anymore. Yeah, I'll just go to the arcade. I don’t know if you but it’s something like Dance Dance Revolution. Like, it’s just Pump It up, lah. I just cannot jog, you know, because I don’t find it fun to do anymore. So I just go to the arcade for 1 and a half hour. It’s like a mini-HIT exercise ah, so I just go there for like once a week to exercise, maybe. But it’s like, if you know like when you work for long hours, then your standing for most of the time. Then it feels like it’s exercise, but it’s not, right? Like your teachers would say your heart rate have to go up and everything. But my legs are so sore, and I’m so tired. So, I would say that I would rather choose my mental health over exercising. Ya, but, exercise does help with feeling better, I would say. Yeah, so I try to at least get my exercise at least once a week. Yeah. I’ll definitely regret it in the future, but for now, it works for me.
- **NJCL: Hmm, then when you said you try to exercise, you’re doing this because of…? Like you mentioned, it makes you feel better?**
- P11: Yeah, exercising. Yeah it’s the same for everyone. You get the the high, and what do you call it? The endorphins. I don’t know, but you feel more awake? Like last time, pre-PM shift, I used to go for a runs, like you know, those fake people. Yeah, but I really couldn’t. Jogging just isn’t my thing. So nowadays I also go for like- okay I like K-pop, I go for like K-pop cardio maybe once a month? Like some weird K-pop dance once a month, at least. Just to make myself happy and also exercise. I think I really admire those people who really go for gym, you know that kind of thing before shift, after shift. And I know a lot of people who go right after their night shift, then they go home and sleep. I don’t get how they do it sia, it’s like, too much strength for me ah. Like you know, that’s why they’re in great shape ah, and I’m in a normal shape. But in the end, it boils down to whatever works for us ah. But at least we have to get a bit of exercise in.
- **NJCL: Earlier you mentioned that you chose your mental health over exercising. Could you elaborate on that?**
- P11: I mean, it's the same when you study right? Sometimes you just have no mood for any other thing other than to like rest. Or you want to like, watch a video or lie down? Yeah, I would rather choose those over exercising. I would say, yeah, kind of like winding down personally for me. Maybe for other people winding down is exercising. For other people, winding down, is like to go and see people. Yeah, I would say, my winding down is it's just head empty, no thoughts. Yeah, at least that 1 hour would rather rest. Than go exercise and push myself the limit. Yeah, to me personally, this is a very personal thing. I feel. Because when I talk to different people also, they have different methods of like, you know, like motivating themselves. You know, they go out and eat like a bowl of ice cream, or this, but for me. It's just resting. Yeah.
- **NJCL: Actually, yeah, you already mentioned a few things regarding my next question. So my next question is, as a nurse, how do you feel about self regulating healthy lifestyle?**
- P11: Not important in the short run important in the long run. Yeah, if I have to work shift work- for instance, I have colleagues that are 40 years old and still working shift work. I still do night shift with them. But if I want to work that long in like a ward setting, I would say, I really have to eat healthily, you know that kind of thing. If not, I'll probably have, like obesity, you know, diabetes. No, but when you're young, yeah, you're young and dumb, right? It doesn't matter. Like my friends are like working full time night shifts in like [Public Hospital name], that kind of thing. You know, that kind of thing works if it's not a long-term thing. Yeah, I would. So I would say, like, it's important. If you want to keep working as a chief ward nurse. But if you are going to go back to like 8 to 5, honestly, I would say, like, like, whatever ah. Yeah, but that is also a personal opinion. Yeah.
- **NJCL: Yeah, it's okay. Personal opinion is what I need.**
- P11: Yeah, okay okay. Can.
- **NJCL: And just now, you also mentioned, like some staff they go to the gym after their shifts. Could you maybe, like elaborate? Why do you think these people are maybe more motivated to maintain their health?**
- P11: [Long pause]. That is a question I’ve never thought of before. I'll say the emphasis is probably more on like taking care of themselves, or like taking care of their body. Know that kind of thing. Like they have a different goal ah. I think, when it comes to relaxing, and all that kind of thing, or like carrying on with life, you have to have a goal right. So when I talk to them, their goal is always like ‘oh my body, I need to take care of it’, that kind of thing. It’s more of their priority I would say. Yeah. And they know how brutal nursing can be. They are normally those who have worked a few more years more than me. I only worked for like 1 plus years, while they probably already worked for like 5 plus years. And they realise that exercise probably helps them to like focus. Helps them feel stronger- that’s what they say ah. Then they won’t feel so tired carrying patients, because just like what I said just now, like, geri is very tiring on the back. Honestly, if I had a bad night shift with like a lot of showering to do, because we normally shower our patients in the morning, then we sponge in the night or trolley bath at night. So it’s like very tiring on the back even though we wear the back brace. I will still feel very sore. My legs would feel like that swollen feeling. So I would wake up when I sleep, that kind of thing. Then they also say that, when they exercise, it helps them get more stamina, that kind of thing. They don’t feel so tired, you know like after night. I guess, that’s their motivation. And sometimes their coping mechanism is just exercising and just de-stressing which I personally do not understand. But I would say, it does help with stress, exercising. But then again, I have colleagues who don’t exercise, so.. To each their own ah.
- **NJCL: Yeah. So so for you personally, exercising is a way to de-stress mainly?**
- P11: Yeah. I would say I- I have fun because I go to the Arcade to like ‘fake exercise’ right? Or at least that's my kind of exercise. When my heart rate really goes up. I really sweat there for like 1 hour, like I feel good. After that I don't know how to say, yeah, it's really the endorphins. I feel my shoulders are lighter. After a tough shift, to exercise a bit, to sweat it out, like all the Qi come out, you know? I don’t know how to say it. It feels good to me ah. I like exercising, but I just don’t have the energy, I feel.
- **NJCL: Yeah. I think again, some of the points you mentioned will probably fit the answer for this next question. But like, what are you main facilitators, motivators in self-regulating a healthier lifestyle?**
- P11: Can you say again? Motivators ah?
- **NJCL: Yeah, like push factors that make you want to self-regulate your health, if there are any?**
- P11: Like I said just now, probably my most important thing is mental health or de-stressing to me. When I'm very stressed out, you know. I’ll go and exercise, or I'll sleep enough, or go and meet my friends, you know. I don’t know if there are other motivators. I’ll say having people who have about the same habits as you will motivate you, yeah. I guess- wait let me try to think. I think initially when I first started out, my motivator was just surviving. After a while, it turned into more of merging it into my- I don’t know. Yeah, I don’t know, but it’s probably me first, in the end. My motivator is taking care of myself. And then making sure I don’t revolve around my work, even though everything is about my work. But it’s making sure that I at least have the feels of a work-life balance. Even though it’s not. I think that is mostly my motivation. To give my best to my patient, for as long as I can. Yeah, so I need to be at my top. I don’t know how to say also, hmm. Okay, I can’t really express it lah.
- **NJCL: It's okay, I think I get it.**
- P11: Oh. It's like not so straightforward I would say. Everyone's motivation comes from different places. So my motivation comes from first of all, working helps to get money to fund my parents that kind of thing. And then but to do that, I also need to take care of myself. Take care of my mental health. And so I want to feel good. Yeah, I think, okay, here, I think my main motivator is feeling good. Yeah. Making sure that I don't feel lousy when I go to work making sure that I don't hate going to work. Making sure that I kind of de-stress after work, so I don't have to think about it 24/7 because that used to be my life during probation, and it was the worst feeling ever like I would go home. I think it's the same for everyone. They would go home feeling like shit because they didn’t do something for their patient. That kind of thing. Yeah, so I want to work hard, play hard. Hmm, yes, yes, work hard, play hard. Yes. Sorry. Sorry. Yeah. Yeah. I want to work as hard and play as hard.
- **NJCL: Hmm. Earlier, you mentioned something about you want to make sure you don't revolve around your work, you want work-life balance. Can you elaborate on that?**
- P11: Let's say you take your patients. There are few patients that are sick. And then sometimes you, I'll say that you miss out things, for instance, like referring to more specialists. Like, let's say, like COPD patient, they get a new medicine. By right, we should refer to the asthma team, you know, instead of like teaching. Okay, we can teach them how to use the medicine itself. But it's better if, like a specialist, take care of it right? Sometime you forget about it, and then you hand over to the person the person forgets about it, and then the doctor doesn't really specify, because sometime the MOs are like new, you know, they don't know certain things or so. It's only like, maybe like that night shift when the senior comes, the really senior takes over that team. Then they’re like “Hey, why you never do this kind of thing? Then sometimes they will directly text you, or when you go back the next day and they’ll tell you like “hey you actually forgot to do this and this,” and my ward has a very good practice of like in your face kind of thing. So if you do wrong, they won’t say behind your back. Actually that day you forgot this and that, and they’ll say it in a very nice way. But you kind of feel like shit you know. In the end you pass to someone, even though I know they are like taking over from me, so we are all doing our part to do this, but in the end, I delay patient’s care by ignoring all these kind of thing because the referral takes very long, all these. So initially, I used to go home and think a lot. Like shit, this this thing happened, or that happened. I didn’t push the PICC line right, you know that kind of thing? If you think like something’s gonna happen, you know that kind of anxiety? That kind of anxiety is very very scary. Then I would say, it’s not a very healthy way of living but because we care about what we do. I’ll say it’s the people who really know their consequences, then they will feel this kind of way. Because in my ward, most people are like that also. And I realise, it’s not the same compared to my PRCP ward. The thinking is very different, but in my ward, if we give like high controlled drug, sometimes we give wrong dose right, the first thing we think of is if the patient is okay? You know that kind of thing? So when I go home, I keep thinking- it’s a good reflection. But too much reflection and you’ll really feel like shit. Like instead of going out and hanging out with my friends, instead I’ll be ranting. Trying to get it off my chest. Yeah, it’s very stressful ah to think about it. A little bit of guilt, a little bit of thinking about work is fine, but when it comes to everyday, thinking about it, it’s mentally draining which I think why my probation was hell. And I think most people, the probation period was also very bad. Initially, the 4 to 6 months, you really feel like shit because you are learning everything. Everything is so new, and you miss out everything. Then your preceptor will call you out for it, because sometimes it will affect patient safety. Or like you’re just being dumb lah, that kind of thing. And you feel so inadequate, you don’t feel good enough. I just don’t like that feeling ah, so I just want to do my best at work. And then maybe think about 1 to 2 hours post-shift. Maybe talk to my nursing friends like, “hey today this and this happened”. So there’s a bit less pressure like, “oh no my patient is going to die”, you know that kind of thing? Once in a while it’s fine, you know, like it’s normal. We can’t control a lot of things in life, and I come to accept that. Yeah, sometimes my patient’s blood goes wack, it’s not my problem, it’s their problem. It’s just that I didn’t catch it fast enough. But I just have to accept it- that I’m human ah, I just can’t do so many things at one time. Yeah, so I would say that’s me working my best that my patient can have the best care and I get a piece of mind when I go home ah.
- **NJCL: Okay. We’ve been talking a lot on like facilitators and motivators for self-regulating your lifestyle. Are there any barriers to self regulation of your lifestyle?**
- P11: OT. [Participant laughs here]. Over time. I think that. Yeah, sometime I would like, okay, I'll plan out everything for the day already, like, my off, my post-night, you know, I’ll plan everything already. Oh, I call it my friends, you know, I cook food at home with my family like that kind of thing. Then, they asked me for OT. Okay, if I don't have a solid plan like my friends haven't settled anything, and the staffing is really very bad. I'll go back yeah. And it will ruin everything honestly but you know, I will feel bad if I don't go back. Yeah, I know. Normally I would- okay. I would- I would limit myself to like one or 2 OT per month. Yeah. But it really throws everything away lah. Yeah, even though the money is good. But yeah, I would say, it's- if I can don’t OT, it’ll be better. But yeah, I OT just to help the ward. I would say the other barriers are like normal human people, like you lazy lah, sometimes you have no mood. Yeah, that kind of thing.
- **NJCL: Hmm, could you elaborate more on being lazy, having no mood?**
- P11: Yeah. Sometimes you just don't want to do anything. Yeah. So in the end I'll just- instead of like eating a proper meal, you know, maybe I’ll just skip and sleep. You know that kind of thing. Or I'll eat unhealthy things, I think that normal. I just need to de-stress, you know it's fine. Yeah.
- **NJCL: And, hmm, regarding- just to go back a bit. Is over time in your workplace common or?**
- P11: Yeah, a quite common. If someone MCs, because staffing- okay, staffing used to be good. So MC Is like fine, you know, like we'll work with the minimum. But we won't work with like less than minimum. But now we are always working with less than minimum. Yeah. So normally they'll call people to come back. Yeah. So obviously, there always be those few that come back often, you know, to help out the ward lah, because they really like the ward that kind of thing. Yeah. But most of the time I go back is really when it's like not enough staff. You know, they always MC- Sometimes they MC at very specific timings like December 31st. People don't want to come in, you know, that kind of thing. But if I really have no plans, I would just OT ah. Not for the money, not for the money. But I need to help out in my ward because I know- and I think if you work long enough, you would know how it would feel to work a bare minimum. It’s so bad, honestly. I just hate that feeling so you don’t want others to go through it. Yeah, so I would say like, I probably worked 10 OTs compared to people? Don’t work too much lah, honestly.
- **NJCL: And then I will just like briefly summarize what you've been sharing with me. So for facilitators. For you, your facilitators are for your mental health. It's a form of de-stressing for you. Then, also, like having people who have the same habit as you.**
- P11: I I guess. Yeah.
- **NJCL: Okay. So having people with the same habit as you is like a influencing factor for you?**
- P11: It's just like our schedule just slot in together. I have 2 other friends who also arcade. Yeah, even though they play other arcade things. Like both of them are nurses, so like what's your shift today? So I take out myduty app, right. Do you use it? You use it right?
- **NJCL: Yeah, I know.**
- P11: Yeah, myduty app. Like yeah, ‘hey your shift is correct ah, it’s this one? Then want to come out? Are you free?’ Sometimes they are free, sometimes they aren’t. But it’s fine. But it’s like having someone that can feel with you, it helps ah. And then maybe afterwards go out for a meal. Like even now ah, me and my friend are post-night. So we both go out and chill, that kind of thing. It helps. Not exactly having the same exact habits, but at least like similar habits. Like talking about anime, this kind of things. And like eating the food that we like, you know, like eating fast food or Korean food. Yeah.
- **NJCL: Okay, then, other than mental health having social circle to support your self-regulation habits. You also mentioned a motivator is like feeling good because you are saying that. It's mentally draining. Always be thinking about work, Yeah. So it’s like you don’t want to feel bad to be always thinking about work?**
- P11: Yeah, I guess. Yeah. I like helping people. And you know, it's very fun to like learn about conditions, you know. Or like talk to the different doctors. It's fun, but with it also comes the stress. Last time, I don't really think about this kind of thing, but yeah, after talking to you. I realize, yeah, that in the end, I just, I just want to do my best and feel good. Yeah.
- **NJCL: Yeah. Because you mentioned that you need to be on a top condition to be able to take care of your patients. Something like that?**
- P11: Yeah, yeah.
- **NJCL: Hmm. then, moving on to barriers. Your main barrier is overtime. Yeah. Because once you schedule and plan-**
- P11: Wow, that sounds so sad.
- **NJCL: I mean, it's understandable. I can see how it will impact then other than overtime, you also mentioned, like self-motivation, like being lazy, having no mood, this hinders or prevents you from self-regulating healthier lifestyle. Is there anything else you would like to add for either facilitator or barrier here?**
- P11: You know, now that you say it, I think it stems from having too much responsibility at work, so I don't want anything else to do after work, even though you know I still have to do the chores and all that. But it’s like I really force myself to go, or like I really go and visit my extended family every week that kind of thing, really go visit like my, you know, extended family every week, like my grandma all that. Yeah, I think it stems from, I really don’t want to do anything, because I already did so much during work, but I still have to, you know. I would say the barriers are being lazy.
- **NJCL: Hmm, moving on to my next question. It's how does self-regulation habits impact your work performance?**
- P11: Better, I feel less stressed. Yeah, I feel like I can actually think clearer. I won’t feel so much pressure. Yeah, I used to, I used to succumb to it, like, you know, when too many things come over or like the doctor tries to talk to you when you’re serving medicine. Now, I can do it already. Like it's like much better. I don't know if it’s because I'm used to the routine now. Or like because I know what to look out for. So I'm like just flowing around with what I remember, you know, muscle memory, you know that kind of thing. I think maybe that's why it's better. Yeah. But I would say, it definitely helps that. Yeah,with like having a better mindset and having good colleagues also help. Yeah. I think that’s about it. Yeah.
- **NJCL: Then, outside of work, do you think anything else influences own self-regulation habits?**
- P11: You have an example?
- **NJCL: Maybe your family members?**
- P11: I mean, my. my mother does nag a lot. Yeah, if I go, I eat too much. you know. Like, don't come home for cooking or like I don't- instead of like sleeping enough, I will- hmm, it’s very mother things ah. Like, let’s say night shift, I only sleep, like from 10 to 2. Then I go out after that. Then I come home, and nap again. Then she will be like "why don’t you just sleep from 10 to 4, then you go out after that?”. But my friend is only free at that timing, so I’m fine with it. Or like, if I come home late, then I don’t eat, she’ll also be nagging. So, I won’t say it plays a part, but it helps me to see my bad habits. But it’s bad habits for a reason right, I will still do it. It’s just my mother cares for me, that’s why she just mentions it. Other things, I don’t know. It doesn’t come to mind now.
- **NJCL: It’s okay. Then, just now you mentioned the myduty app. Are there any other tools or apps or objects that you personally use to self regulate your lifestyle with?**
- P11: Let me check. I think nothing. Like what, physical? I have the healthy 365 ah, if I want to go for the free things. But that’s very rare. Hmm, but nothing eh. Coffee app? Nothing, really nothing.
- **NJCL: It’s okay. Okay, Then, I'll just move on to the next few set of questions. This one will be more specific to the workplace. As a nurse, how do you think the workplace influences your self-regulation habits?**
- P11: How the workplace influences?
- **NJCL: Like, how you self-regulate yourself.**
- P11: I feel like I’ve been answering all these questions but in a roundabout way.
- **NJCL: Yeah, I do agree you did touch on this.**
- P11: Yeah. I guess it’s mainly the stress thing, that’s about it. And working around the shift work and all that.
- **NJCL: Hmm. Okay, then, specifically on workplace culture, like, how do you feel about the workplace culture among nurses in helping facilitate or hinder the the self regulation of your lifestyle?**
- P11: Mine is good, I would say. You're not scared to ask questions. I think that is because our seniors built that kind of culture. Yeah so, if like there’s a new staff, you know. You ask a lot of questions. Sometimes you know people can be very paggro about it. And then they rage like, “what do you mean you don’t know about this? I thought you come from degree, you know. How come you don’t know all this thing?”. I heard a lot of horror stories about this kind of thing. But it’s mostly just my colleagues jabbing me ah, but in a very nice way, like “hey, I thought your uni student” you know, that kind of joking. But I would say, because we are always so stressed most of the time, so in the end the culture becomes a more happy mood. Then if our patients something happen, then we can still rant about it. Or like we’ll joke if our patient desat like “aiyo, my time sia” or like you know we pat patient- actually it’s a very serious matter but at the end, we joke about it. Yeah, but, it’s how we cope la. It’s how we cope. Yeah I would say, it’s better than other wards that I hear where they will scold people to cope with the stress. Like they will just let it out and rant on someone ah. Sometimes we need that, but it’s not healthy. It’s very not healthy. So I would say, on off days, if my friend really wants to rant in the pantry for all of us to hear, all of us will be like “yeah, yeah”, but it’s not an all the time kind of thing lah. But yeah, it helps.
- **NJCL: So would you say that the workplace culture, at least specifically for you, it facilitates self regulation of your stress because you’re able to like vent out and it's quite supportive towards each other?**
- P11: I think it’s not perfect ah. Because of the stress, everyone MCs right, so it creates more stress, that’s the bad part about our ward. Yeah, but I think in general, the people are fine. It helps to- you know when you have that culture, where people come in, they will also join the culture. Yeah, so if it’s a bad culture, people will also join that bad culture. So I would say it’s mostly good.
- **NJCL: Then. What do you think about the role of nurse managers in helping nurses like self regulate their own health?**
- P11: Asking us to come back for OT, I don't think they care. They will verbally say it, but they won’t reinforce it. Like, “hey why you never go home on time?”, “why you never go and eat?”, you know? Why you this, why you that? But will they enforce it? No. So I would say they are there for like support, but you know, different kind of support. More, I would say management is management lah, what can they do about it. Like, what can they about bad staffing? Call back more staff? Or like, what can they do about hard patients? Ask you to go and apologize? Speak with their family? I would say that they help out but tangible wise, there’s not much they can do also.
- **NJCL: Hmm, that's it. Hmm. Hey. Just summarize a bit when I asked about the workplace culture, for you, you say that overall, it actually facilitates self-regulation of lifestyle. Then you also mentioned that the nurse managers may say things to maintain your health, but in the end, they can't really enforce that, they can’t really influence that.**
- P11: [Participant laughs here]. Wow, that’s so toxic, but it’s true. Yeah, yeah, that’s it.
- **NJCL: Is there anything else that you would like to add here?**
- P11: Yeah, no. It’s whatever just comes to mind.
- **NJCL: Then for the final question, how do you think your institution can help you to self-regulate?**
- P11: They make a lot of effort, like they say “oh, can go for this exercise together”, or like go and eat healthily, then can go for this workshop to see all this stuff. Will I go for it? No. I don’t know how else they can help. Because in the end it’s like- that’s not what we need at the moment. But then again, that’s higher management. I don’t know what else they will do besides like, give us more leaves. Because they already increased our pay, and we’re still dying. So I don’t know what else they can do. Oh, more staffing. More staffing would be good. But staffing is another whole issue. Yeah, that’s it. Nothing much.
- **NJCL: Then are there any workplaces health promotion programs in place other than the activities that you have mentioned?**
- P11: I don't know. They always handover but I always zone out. I think there probably is. That time, there was somekind of food fair or something, where dieticians speak about what and what, but I honestly don’t remember.
- **NJCL: Then so would you say like these things the institution are doing are effective? Or not effective?**
- P11: I think not effective. Even when I ask my colleagues, they will be like, so where’s the extra leave? Or like public holiday, when am I gonna have it? Like this kind of thing, because you know, election day that kind of thing right. But ya, I don’t hear anyone talking about it.
- **NJCL: Like personally, you don’t go for this activities?**
- P11: No, I won’t go for it ah. Yeah, like, why waste my time right. When I could be going for something I really want to.
- **NJCL: So what do you think will make it more effective, or make you want to participate?**
- P11: K-pop stuff? I don’t know, something that’ll interest me? But then again, I don’t think they’ll ever have this kind of thing. Because like, it’s very general right. I mean like last time, Nurses Day, they did try to have a dance and stuff, but I’m not going to participate. It’s not my thing eh. Maybe if it’s a ward thing, I also don’t really go for it. It will be hard to motivate me. Yeah. Is that what you’re trying to go for?
- **NJCL: Yeah, yeah, it’s relevant. Hmm, okay. Then, there's something you mentioned. You said something like ‘at the moment, it's not something we need right now’, ‘something you need right now’. Can you elaborate on that?**
- P11: Maybe when I grow older I will have more time. Maybe when I grow older, I will have less time? But maybe at that point, I need to like learn more about my health, you know. Touch wood something happened. Maybe then I'll go for all this of extra things that were be relevant to me, but for now it’s not relevant to me. Yeah. Yeah. So I don’t really need this kind of thing.
- **NJCL: Then, just to maybe to clarify a bit. So you're saying that if if there's a change in your condition, then you are more keen for these kind of activities. But because you are still considered healthy, you don’t feel the need to prioritise your health? Is that right for me to say?**
- P11: Yeah, I guess so. Yeah. for me. I will say, if it's next time, it's more applicable to me. For instance, like eating healthier. But then, you can google stuff right? But it will be nice to hear from an expert, I guess. Or if I have like no more activities to go to and I want to exercise, or like free coffee, then I think I will be inclined to go.
- **NJCL: Hmm. Okay. Then to summarize. When I asked about how do you think your institution can help you self-regulate a healthier lifestyle. You say that they do organize, they do try to organize activities for the nurses but you feel that overall it's ineffective because it’s not your interest. It's not something that's applicable to you right now. And how you think they can really help is to have more staffing. Is that all? Is there anything else you want to add here?**
- P11: That’s the best summary. Maybe if there's enough staffing I'll go for it. Wah, toxic. But aiya. You know, like there’s the thought right, but whether it happens…
- **NJCL: Okay. Then do you have anything else that you'd like speak about that was not mentioned in the interview? Any further comments to share?**
- P11: No.
- **NJCL: Okay, then this marks the end of the interview. Thank you for your time and participation.**
- P11: Huat ah. Long sia. I didn’t expect.
- **NJCL: I’m so sorry.**
- P11: It’s okay.
- -End of P11 Transcript-

# **Interview #12**

Participant and interview details
 Participant number: 12 (P12)
 Date of interview: 28 Sept 2023
 Interviewer Number: Navarro John Christopher Lambino (NJCL)
 Time IDI started: 1900
 Time IDI ended: 1950
 Observational notes: Participant looks slightly annoyed.

Section I: Interviewer’s summary
 The individual interview was conducted via Zoom. Supposedly, interview start at 1830, but due issues, it only started at 1900. P12 compared to previous interviews is very formal, less casual. Despite this, she gave straight and relevant responses. Again, shift work, time and the idea that one’s lifestyle is dictated by one’s own inner preferences and discipline. Very interestingly, she mentioned that there will be no impetus to change one’s lifestyle without a certain conditions: genuine interest to change, or reason to have to change (Health condition). She also mentioned that nurse managers do not have the capacity to help, since too busy with their own time. She also used the word ‘hypocrisy’ when describing nurses who do not practice what they preach. Perhaps more should be explored on the idea of nurses not practising what they preach. She says that someone fitter will be a better example and hence more convincing to. Lastly, she mentioned the changing needs of healthcare, everything becoming more difficult adds to the mental stress of the work. Institution can help by reducing manpower issues.

Section II: Transcript

- **NJCL: Okay. Firstly, good, good evening, miss. Firstly, I would like to thank you for taking the time to speak with me today. My name is Chris, and I'm a NUS Year 4 nursing student. The aim of this interview is to get a better understanding of the perspectives nurses have on self regulating their health behaviors and how various factors may influence this. I want to find out what are your views, opinions, and experiences on this. I would really like to encourage you to speak what comes to mind. There are no right or wrong answers. May I have your permission to continue with this interview session?**
- P12: Oh, yes. Maybe just speak a bit closer to the microphone as it tends to break.
- **NJCL: Oh, okay. Then let's start with some basic questions like, which ward are you working in right now?**
- P12: I'm currently working in the ICU.
- **NJCL: Could you describe the kind of work and your workload?**
- P12: The work that I do is to take care of critically ill patients in the ICU, and also to help manage the ward. And also guide new staff that we have on duty so essentially just, patient care, and also sometimes involving also some additional time to look after our other staff and to set assignments depending whether I'm taking a case, or I'm managing the ICU for that day.
- **NJCL: How would your describe your workload?**
- P12: Sorry, could you repeat?
- **NJCL: How would you describe like the amount of work that you have to do, right now?**
- P12: My work is relatively, physically intensive, because there's a lot of of movements that's required, and also a lot of your own body weight that is required to manage the patient, and it can be quite heavy and stressful as well because of the intensity of the work that we do and the criticality that it involves in as well.
- **NJCL: Okay, and thank you for that. Then, moving on to my next series of questions is regarding lifestyle in general. So as a nurse, how would you define a healthy lifestyle?**
- P12: Hmm. Healthy lifestyle probably would be to have adequate rest, and adequate time to eat. And also exercise, and, as well as the mental health aspect, to have time to offload and de-stress such that your don’t bring your baggage home, or let it affect you outside of work, or even in work as well. You just have to be quite able to be in control of the situation and not let the situation control you.
- **NJCL: Sorry. Could you elaborate more on your last point about being able to put through the situation.**
- P12: Because there's always things that are unexpected. So if you let the situation overwhelm you, and then you will not be able to be in control and make the necessary movements and actions you need, because you're too overwhelmed by everything around you. So if you have a proper understanding or framework in mind for yourself on how to deal with difficult moments or stressful situations, then it will be easier on your body and on your mind.
- **NJCL: Hmm. Earlier you mentioned the mental health aspect, and like having the time to offload, can elaborate on that?**
- P12: Because work itself is already stressful, your own nursing activity, your own nursing work, and you also have to take into account that there's also the social aspect of work where you have your managers and your colleagues, as well as your family as well. So if you have to put all this into perspective, like doing, your work could be difficult. But there are other factors that come into play whereby the situation may not help if you have a difficult manager, or you have difficult colleagues, and maybe. like your family, is not able to understand, or get to know what's going on with your work, because it's difficult for them to relate. So it gets stressful if you do not have an outlet to get rid of this troubling thoughts that you may have, or, like the difficulties that you have encountered.
- **NJCL: Hmm. You mentioned like having a outlet for this stress. Could you share with me more of your like personal outlets for this?**
- P12: I will take the time to talk to some of my friends or colleagues who are in similar backgrounds, such as healthcare and also exercise. So that's one of the other outlets that I have. And probably just do a little bit more reading on either self-help or reading about the difficult parts that I do not understand, to get clarity on what I'm experiencing. So it's more of trying to again a deeper perspective of what I am encountering. Instead of trying to be reactive to the situation
- **NJCL: Right. Then, you mentioned exercise. Physical activity wise, how do you feel about your current lifestyle?**
- P12: Hmm. quite okay. With regards to my, the amount of physical activity that I participate.
- **NJCL: Could you elaborate more on what you mean by ‘okay’?**
- P12: Just I try to exercise at least on a daily basis. If not. I'll just go at least 4 to 5 times a week. So, to ensure that at least I still am a bit healthy, and well enough to manage my own weight.
- **NJCL: Hmm. then with regards to exercise and physical activity. What do you think motivates you to engage in these activities?**
- P12: Sorry?
- **NJCL: What motivates you to engage in physical activity?**
- P12: To maintain my weight and to stay healthy. That's all. Hmm, yeah, that’s all really. It's just to maintain my weight and to stay healthy.
- **NJCL: I see, I see. And then regarding specifically on diet, how do you feel about your current dietary habits?**
- P12: Dietary habits? I’m satisfied with my current diet. I have no particular diet that I follow. So I just eat and drink things that I enjoy, and I want to without restrictions, so I do not impose any diet on myself, and I just choose what I want to eat.
- **NJCL: Okay. And hmm. Then moving back a bit. Can you yes, explain to me, or like, tell me about, how did your lifestyle actually change after you started to work as a nurse?**
- P12: So yes, my lifestyle didn't change. It was a little bit more sedentary than it currently was because it took me time to get used to my work. And it's only after a period of study that I went, and I realized I had put on a bit of weight, and I felt more sluggish because of the inactivities that I- that the lack of activity I have rather and just having a free diet without any output. And I realized I was not able to support myself and work because my work is quite labour intensive, so that gave me a bit of reason to exercise and to maintain my uniform size as well, if you want a very honest reason, yeah. Because, I want to maintain the same uniform size since I'm in a uniformed industry. Yeah.
- **NJCL: Hmm, hmm. Understand. Then moving a bit as well, some of your answers did touch on my next question. It's regarding- as a nurse, how do you feel about self regulating a healthy lifestyle?**
- P12: I think that it's a very individualised thing, because every nurse is different. If they have different circumstances, like singles and married individuals. We'll definitely have different priorities. So I am still not married, so I am still able to have the time to exercise, and have the liberty to do it more frequently on a more frequent basis compared to some of my other colleagues. And also making use of whatever is near you. And what are the motivations that can help you at the exercise. If having a buddy helps, then maybe it's good to go with a buddy to exercise, or if you need to use classes to motivate you to stay on track because it's paid classes. Then it's also another alternative to use or just utilize the facilities that are near us like our house gyms, or the work gym. If it's really necessary to just use something near you and it's a low cost as well.
- **NJCL: Earlier, you mentioned that self regulating a healthy lifestyle is individualized thing, and every nurse is different. Other than building on this, other than like the nurse circumstances, can you elaborate more on this?**
- P12: How would you like me to elaborate further?
- **NJCL: Like, hmm. Maybe. How some nurses might be motivated to self regulate themselves compared to others. What do you think makes these nurses more motivated self-regulate compared to other nurses?**
- P12: I guess it's just having the mental strength and the determination of wanting to continue to exercise. Everyone has different motifs for their own reason. To exercise some is purely for physical appearances, some is to maintain their strength, to build their strength. Probably others are also just for health purposes, to stay fit for this for their age. I mean, we have nurses of all ages, and they can exercise for different reasons, either for rehab, for maintenance of figure and maintenance of health as well as personal interest in exercise as well.
- **NJCL: Okay. I think my next question, you also touched upon it. So just clarify some points like, what do you think are the main facilitators and motivators in self-regulating a healthier lifestyle for you?**
- P12: I believe, a similar like-minded community would be one of the factors So if you do have more peers or people, you are around to also exercise, and are willing to exercise with you. And do not put off exercise. The likelihood you would exercise is higher, because there is another motivating factor whereby the social aspect comes in. And also, personally, I feel that it's very ingrained to the person who wants to exercise because we are no longer mandated, like in school, whereby you have to go for physical activities, like in PE classes. But now it's a choice, it becomes a personal choice to exercise. So if they have a wiill to exercise and do participate in physical activity. it's probably have to stem with something that resonates with them. Either they want to lose weight or they want to maintain their figure, or they enjoy physical activities. Or they are motivated- more motivated to participate if they have a circle of friends or family members who exercise as well. Yeah.
- **NJCL: Hmm, okay. Then. moving on from facilitators, slash motivators. What do you think are barriers in self regulating a healthy, healthier lifestyle for you?**
- P12: Oh. barriers will probably be time, and fatigue. Because we are working adults, and it's quite tiring to set up the time to go and exercise. So it's either before- unless it's either early in the morning before afternoon or after your shift, whereby it's probably in the evening. So there's no guarantee how the day would be. So we just have to choose a day and a time to exercise and regulate your own intensity of the activity that you choose to partake in. And also your own schedules. So if you are doing your own part time studies, then that will also take that time from your other free time to exercise. So, apart from what your own personal activities can also come into play. and maybe personal commitments as well. If you need to be home to help with the family. And you don't have the time and luxury to have a long exercise, probably a short one, rather. and that would be a barrier on choosing what activity you can choose. Yeah.
- **NJCL: Man. Okay. Then I'll just summarize what you've been sharing so far. So for you, facilitators, slash motivators is having a similar like minded community like a social circle, and also actually having it ingrained in you like. I would say, intrinsic, Intrinsically you are motivated by yourself, is that correct?**
- P12: Yes.
- **NJCL: Okay, then, for barriers. You mentioned time or lack of time. There's also fatigue and your own schedule and personal commitments that may clash with your intention to actually engage in a more healthier lifestyle. Is that correct?**
- P12: Yes.
- **NJCL: Is there anything else you would like to add for facilitators and barriers over here?**
- P12: Maybe cost can also be a barrier if you choose to participate in more costly activities that are available in the market. But you like them, but you don't want to pay so much for them. So you probably may not go for such classes that often. And also memberships, and outside gyms are also relatively costly, and to make it cost-effective, you probably have to go quite frequently, but if you don't have the time for it, you may rather just miss the whole thing save on that money. And probably motivators would also be, maybe, if there are incentives or rewards within the company. If you choose. if you have, like participated in some exercise games that they have hosted, and you stand in line to win some prizes so that can help to kickstart the the journey. Yeah. probably another barrier is also the fear of wanting to display yourself. If you are not fit. Or you feel that you don't look fit enough to exercise outside, compared to people who are already exercising on a regular basis. and the lack of knowledge or not what to do if you choose to, if you want to exercise on like specific activities and without sustaining injuries at the same time.
- **NJCL: Hmm.**
- P12: Yep.
- **NJCL: Okay. For your last point, the lack of knowledge is regarding like what kind of activities you're engaging in?**
- P12: So perhaps those who are interested in using the Gym setups, but they do not want to engage with a trainer, and they want to do it on their own, but they are not sure how and where to seek for help, so they would just skip it altogether because it's a hassle unless they are really interested and yeah, willing to seek for advice and help. So if you choose to exercise on your own, but you have no knowledge on how to safely exercise. The risk of injury is very high, and when you injure yourself there will be a downtime, and the recovery period after downtime may be quite long, and your lull period will probably make it harder for you to pick up.
- **NJCL: I understand. Hmm. Then, moving on to my next question. How does your self-regulation habits impact your work performance?**
- P12: Could you elaborate on self-regulation habits?
- **NJCL: It's like your own personal lifestyle habits like, for example, you mentioned that for you, you actually like to exercise and engage in exercise for like 4 to 5 times a week. So does this affect your work?**
- P12: No, it does not. In fact, I feel that it gives me a bit more strength to assist with my patients better, so that I am able to hold them stronger and be of help with my teammates as well. Also. I just need to know how to better schedule my time and be in control of it. So the only thing that would probably delay my- probably would affect the scheduling is when you work. There is no definite- there is no exact end time unless you know specifically that it's going to end early or end on time because some days we will never go back on the dot, it is highly unlikely in my area. I'm not so sure about other areas, but my site is harder to end the work on time. Probably there'll be some delays by half an hour or an hour depending on the situation that we encountered that day so if it's a very busy day, then it'd be harder to motivate yourself to go for an exercise whereby you will want to relax and rest.
- **NJCL: Hmm. Hmm. Do you utilize any form of like tools or apps that help self regulate your lifestyle?**
- P12: Ah, just fitness trackers from the phone. Just that one only to track the physical activity that I do. That's all.
- **NJCL: How do you think this fitness tracker apps help you?**
- P12: It will just show the amount of energy and how many minutes I've exercised and the frequency of exercise. So that's all I wanted to check. So that's all I needed to check as well. Perhaps if it's all distance related, then I can see how long and how far I went. And maybe that can also be a source of motivation if I need to maintain or improve on the speed.
- **NJCL: Yeah. Hmm. Just moving back a bit regarding the your personal self regulation, habits, impacting work performance. So for this question, you answered that you're actually able to help out more after like exercising because, you feel fitter, and you can actually lift your patients more. Then, you also mentioned something about being able to better schedule your time?**
- P12: Yeah.
- **NJCL: Is there anything else that you'd like to add for this part?**
- P12: Not really. I'm not sure what other information you require. I-
- **NJCL: It's okay, yeah, it's okay. Hmm, okay, then my next series of questions will be regarding the workplace. So I think you answered a bit here. As a nurse, how do you think the workplace influences your own self regulation, habits?**
- P12: I believe that if your workplace is a like-minded community that enjoys eating then yes, everybody would surely schedule time to eat, but if your workplace is also one that enjoys exercising, and then they will also schedule, maybe, like minded activities. So in my area, we have a lot of people who do exercise, so that is a source of mutual motivation whereby some of us will get together and go for a class or go for a gym session together and participate in physical- like some sort of activities, or recommend different types of activities to one another, to try. And that is a way that we motivate each other to exercise because everyone engages in physical activity. So those who are not and are keen on starting. They have a team of people that they can ask for help, and they can bring them along to just show them a little bit of the ropes or start the whole process together.
- **NJCL: Hmm. So far, what you've been sharing is regarding the social aspect of the workplace, other than social aspect of the workplace. Is there any other way the workplace influences self-regulation habits?**
- P12: Do you have some self-regulation habits that you can suggest that I can build upon?
- **NJCL: I think for example, maybe your diet, being more regular in eating, perhaps even like your mental habits. How your way of thinking has changed because of your workplace something like that.**
- P12: I guess, if it’s like for dietary, then the organization itself has healthier food choices if you are buying from the workplace itself. So there isn't much of like unhealthy food that's available, if you are asking. So people who choose to eat there, maybe- I won't say limited, but they have lesser, unhealthier options to choose from and with regards to maybe mental health aspect of self-regulation. I guess, maybe because work is more- it gets difficult sometimes, and it's not always the easiest thing. So people are always relatively penned and stressed at work. So there may be moments where people are very tense and situations are tense. And the way they regulate their temper or anger is probably to be very direct and just talk it out in front of everyone. So it's a very direct way of communicating. At least at my area, to release that frustration that they have.
- **NJCL: Hmm**
- P12: Other self regulatory habits, there's no regular time for food, because we work by shifts. So it's really our dinner time is really earlier than most people, and our lunch time as well. So it's not really well regulated, although we do try to get everyone to have time to rest, or, if they choose not to eat, at least time to rest. Time off the rest. Yeah.
- **NJCL: Okay, then moving on to my next question. It's regarding the workplace culture among nurses. Earlier, You already mentioned, like having a workplace with like-minded people will actually help facilitate your self regulation of healthy lifestyles. How about like the opposite? Does the workplace culture among nurses actually hinder the self-regulation of healthier lifestyles?**
- P12: Ah, yeah, I guess it does hinder, especially with regards to sleep schedule. So like by working on shifts, we have relatively irregular sleep schedules. So if we are doing afternoon shifts and the next day you're doing morning shifts, you're probably getting maybe at most 5 hours of sleep a day, and if you really required longer hours, then it's very hard to get that. Because you are going back to work within the next 12 hours or lesser. So if work ends late, and then you have to be back on time the next day, so that gives you a very short time, and irregular sleeping schedules can make people very irritable, and sometimes affect their regulatory habits, such as binge eating or wanting to sleep more. And having very bad control over themselves. And also like, if you do night shifts, we're always at risk of weight gain and a lot of other health conditions. So it also puts those who do night shift at risk for developing poor behaviors as well and poor habits. Yeah, so the shift itself is quite tiring. Shifts are not easy, because it takes a while for the body to get used to.
- **NJCL: Hmm, hmm.**
- P12: Yeah. So rest is often compromised on our side. And if you're tired, then you probably wouldn't want to do other things.
- **NJCL: And then for you, what do you think about the role of nurse managers in helping nurses self-regulate their health?**
- P12: I don't think the nurse managers are in a position to regulate our health whereby they don't even have time to- I mean they don't even look like they are fit to begin with, I'm so sorry to say that. Half the time they are mainly either very obese or like they are- they don't even have time to go home on time. So they are going home late, like after 2 shifts. So I don't even think they have time for their own self if they start at 7 and you leave at 9, so I don't really know where to get the time to set an example to regulate good behaviour. So nurse managers, I don't think they are the best examples, because they don't seem to be doing a part. And yeah, we don't really see. I don't really see how they can actually help and support physical activity.
- **NJCL: Hmm, hmm. Earlier, you mentioned something about setting an example for behaviors, could you elaborate on this?**
- P12: Setting an example for behaviors?
- **NJCL: Good behaviours. You mentioned like nurse managers aren’t even fit, so they can’t really set an example for good behavior, something like that?**
- P12: No, if they aren’t fit and you are asking them to promote fitness, it doesn't make sense, that's what I'm saying. If you're asking to use the nurse managers to promote that, and then, if you're asking nurse managers to promote maybe good communications and like de-stressing, then they better- they have to be someone who is good at communicating or someone who is not easily angered, and probably speaks with a lot of tension.
- **NJCL: Hmm, hmm.**
- P12: If they do have all these, and they are fit, and they are- they look fit, they look healthy, and they regulate- like promoting all these behaviors, then. Yes, it does make sense. But from what I do observe, it's like they don't always seem to be the fittest people around you. They have a very busy schedule as well. And also very tense because of the nature of their work. So not everyone is a good example. If you want everyone to be a good example, that'd be difficult, like maybe they can have other champions. But to expect the nurse manager to be the one to be the champion is a bit too much. It's a bit hard to attain.
- **NJCL: And so for you, if you would see a fitter, a healthier nurse manager, you would follow- you will more likely follow this nurse manager's health advice?**
- P12: Perhaps, perhaps.
- **NJCL: Hmm. Then, using this idea, do you think this applies to when a nurse is giving advice to patient?**
- P12: Definitely. I mean, if let's say you are someone who is obese trying to educate someone on weight loss. Or if you are a patient who is buying food, and the nurse in front of you asks for 100% sugar in her drink. And the next moment she's telling you on how to prevent diabetes. Then there's a lot of hypocrisy and contradictory statements like within the action and the statements. So it just becomes something that is knowledge that they have- the nurses have on what's good and what's bad, but not practice because of the lack of interest of practicing, or their own personal preference is to have and to enjoy their current lifestyle.
- **NJCL: So is it right for me to say, from interpreting what you were saying, like, the nurses who don't practice what they preach, there's no impetus for them to change, because it's their own personal preference?**
- P12: Yeah, yeah, they have the knowledge on what is good and bad. They are aware. However, it is their duty to educate, it is our duty to educate our patients on what's good and what’s bad. But whether you yourself choose to practice is another set of issues altogether. And it's on your own onus to practice it. And if there are no penalties or if there are no rewards to encourage you to probably practice healthy behaviors for some people, I would say, then they would choose not to. But if they are genuinely just interested in managing their own health, then they don't need the rewards to begin with.
- **NJCL: So you're saying that people without this genuine interest to take care of themselves, we’ll never change their health- their lifestyle?**
- P12: In a sense, yes, until they find a reason that they resonate with like, maybe a health condition strikes them, or a loss of a loved one from a condition that could have been prevented. Then that would be probably the impetus. Or perhaps, like the factor that sparks the change. Otherwise, for most of them, or most people rather, if there is no reason to change. they probably don't see any value in adopting something new unless they can justify for themselves why they want to do it.
- **NJCL: Okay. Then, actually moving on to one of the final questions. How do you think your institution can help you to self regulate a healthier lifestyle?**
- P12: Can I ask for less patients?
- **NJCL: Okay.**
- P12: I mean, workload is quite heavy, not just because of patients, but because of the demand from your patients as well. So yeah, so yeah, of course, if you have lesser patients, we will be very thankful. So like, if patients can manage their own health and not be admitted, then that'd be great. But of course, like the demands of the care and the demands from patients and their families gives you a lot of stresses and a lot of additional things. And there's also a lot of additional work like additional minute things that needs to be done as part of protocol and policies. So that becomes time that you need to do and like checklist that you need to check off. So that's quite a lot of things. I mean, they do try to tell you that there are activities. There are freebies, there are staff rewards and benefits, but- and like, maybe dinner and lunches you can attend. But half the time you have shift work. And this doesn't apply to you. Because these events start probably from 12 to 2, and then you are back into the ward. You're not like the target. You don't feel like you are the targeted audience because it's the office hours people who benefit most from such things because they have more fixed hours, and these events are organized by people who have fixed hours. So it is not something that you would say you want to try and look forward to, because it's almost impossible based on the nature of our work, where it's very busy and turnover is high and the demands, expectations from not just your managers, but from the hospital, the organization itself is already high, because they are mandated by MoH to have to release your admission, your discharges as soon as possible. People are just sticking to KPIs So there’s a lot of factors. I mean, the hospitals can say “oh the institution can try”. But if everyone is all trying to follow a KPI or achieve their KPI, it becomes something that doesn't really affect those who are doing inpatient work. Yeah, of course, everyone is just chasing numbers at that point in time. Yeah. So I'm not so sure what the organization can help us rather than just reducing the number of beds that it can have. And probably, maybe if they really want to reduce our mental health stress from the patients, or like from the family, is to be very firm with like patients’ families who are demanding, unreasonably demanding or not safe for us to work with. That's one of the areas, but other than that I don't really know what else they could do to help us.
- **NJCL: Earlier, you mentioned that the institution actually tries to organize activities. They organize freebies, lunches, dinners. Do you think overall these measures are ineffective to you? Is that right?**
- P12: It doesn't affect people who do inpatient care, like ward level care. I would say, because mainly these activities happen around our busiest time and not our break times. So it happens during the breaks of people who have office hours, it happens during their break time. So, thus, it doesn't really have much of an impact on us because it's hard to find time to go down. Unless, like your boss mandates you to go there and participate in the activity.
- **NJCL: Hmm. then, moving with this idea. Do you know of any existing workplace health promotion programs that your institution has?**
- P12: Yeah, they have organized like classes, like zumba classes for those who are interested or like pilates, and they pay like a small fee for those classes, and they have those- I think now that COVID is more or less settled, they have restarted those interest groups like badminton interest groups. And those who are interested can join. Then people put up notices to us interested parties to join. Yeah.
- **NJCL: So personally, have you attended any of these interests groups?**
- P12: Uh. No.
- **NJCL: Do you know of anyone who-**
- P12: I think there are some people who are interested, so they will participate.
- **NJCL: So for you, would you attend a similar interest group that fits your own interest, or do you think it's too much of a hassle?**
- P12: Personally, I would find that it is too much of a hassle, unless I'm very interested in it. I would consider. Otherwise. I think I'll just settle it on my own. Yeah.
- **NJCL: Okay, can. Alright. Then I'll just summarize what you've been sharing so far. So for you, the question on, how do you think your institution can help you self-regulate a healthier lifestyle. You mentioned that they tried these activities, lunches, and dinners. But unfortunately, it's not really catered for ward level nurses because there's a clash in the schedule and and all that? Am I right to say that?**
- P12: Oh, yes, that's what I would assume- that’s my believe.
- **NJCL: Then, for you, you feel like the only way the institution can actually help you is to lessen the amount of patients, if possible, like manpower issues. I would, I would think.**
- P12: Yes, I guess so, because I mean, we have people that are leaving definitely. And we have a lot of seniors that are leaving as well. So you do have new staff, new numbers coming in, but it takes time to train them, and if you are in a more senior role you are expected to oversee, so that can be another mental stress, because you are taking on a teaching role as well. And you are also doing your own things that you need to maintain, and you need to impart adequately as well. So that comes a little bit of a stressor as well. And I guess, like the changing needs of like the healthcare is also a bit of a stressor. So things are a bit more complex nowadays. so that becomes a bit- things can become a bit busiier as well. So yeah, just having to navigate through everything.
- **NJCL: By busier, you mean like there’s a lot of additional things like miscellaneous work?**
- P12: Miscellaneous work, or like newer treatment plans and more complex treatment plans and things are not as clear cut as previously. Like, you can have multiple ways of managing our case, and it can be very redundant at times, but because you're not in a position to overwrite your medical team, so the medical team can offer many different kinds of life-prolonging therapies for patients at the expense of the patient. But for the family, if not, it can be vice versa for the patient who chooses it. Or there are a lot of new technology that's coming out also. So you have to get to learn them as well. So yes, also, like changing needs of the, of the industry. Yeah. And while there's like automation to help you and and technology, there are still some redundant activities within the work that hasn't been clear out yet. So you know, it will take time for them to be cleared out.
- **NJCL: And and, hmm. Hmm. Then, additionally, what you've been saying, you said something about the institution can help by actually being more firm with patient families are unreasonably demanding. Or like not safe to work with.**
- P12: Yes. I mean, that would be good if it can happen. There is a lot of changes in the current families that we see so- and the expectations of Singaporeans are always very high and expecting things to be done fast, and everyone sees themselves as a priority. So if everyone wants to be a priority, then there is no such thing as priority at the end of the day, because they all just one things to be done their way and probably just neglecting explanations from the medical side or the nursing side as well. So yeah, it's having to manage increasingly difficult demands which you probably- we probably won’t have learned it or experienced it in the school setting. So, it's something that is a skill that will have been, which has to be learned via experience. It's a difficult thing, I would say, because it changes, demands changes quite quickly. and they are getting difficult day by day, because everyone's getting more educated nowadays.
- **NJCL: Well, yeah. Hmm, hmm, hmm. Okay. Yes. Okay. Then, earlier, you also mentioned about like institutions just trying to achieve their KPIs. They mostly seemed more focus on chasing numbers than actually caring for the the staff?**
- P12: Oh. That would be a staff's perspective definitely. That would be a staff’s perspective.
- **NJCL: Okay. Can, alright. Is there anything else you would like to add at this point?**
- P12: Uh, no.
- **NJCL: Okay. Is there anything else you’d like to speak about that was not mentioned in the interview? Any further comments you want to share?**
- P12: Oh, not that I have any, unless you have any more questions for me?
- **NJCL: Oh, no more questions**
- P12: Alright. Then, I’ll take it that we’re done?
- **NJCL: Yeah, this marks the end of the interview. Thank you for your time and participation.**
- P12: I hope my answers were insightful.

**NJCL: Yes, very!**

-End of P12 Transcript-

# **Interview #13**

Participant and interview details
 Participant number: 13 (P13)
 Date of interview: 30 Sept 2023
 Interviewer Number: Navarro John Christopher Lambino (NJCL)
 Time IDI started: 1130
 Time IDI ended: 1220
 Observational notes: Participant seems extremely lethargic.

Section I: Interviewer’s summary
 The individual interview was conducted via Zoom. The interview was initially suppose to start at 1100, but participant asked for some time to delay to 1130. Upon entering the interview, participant looked alert and ready to start the interview. P13 would take some time to think of her responses after every question compared to most of the other participants. Therefore, because of this small but relatively significant time to think about her answers, she gave very comprehensive answers to all the questions, always linking it back. Little prompting was required to illicit relevant responses and she was answering parts of the next question before even being asked. Her responses was primarily about how self-regulating one’s health is one’s responsibility (She used the word ‘accountability). She also went through the different aspects: physical, mental/psychological, emotional aspects of health compared to other participants who tend to focus on physical. Perhaps subsequent interviews will need to be prompted to address these other aspects. P13 is relatively a motivated individual who had activities outside of workplace and involves herself in various activities. Interestingly enough, also mentioned by another participant, SHIFT WORK IS A FACILITATOR as it allows for planning of events/flexibility. She also elaborated on how her lifestyle habits impact her work performance. She described her workplace in detail, giving context to her answer. Like most other participants, P13 also expressed how current activities organized by her institution are not meant for nurses due to clash in schedule. She gave a suggestion on how it could be held multiple days. Lastly, her response on the role of nurse managers was elaborated on and she believed they have dual role: roster practices for optimal health of ground nurses, and being in touch with staff health by being on the ground. She also expressed that institution cannot do much as health is a personal thing

Section II: Transcript

- **NJCL: Alright. Good morning, miss. Firstly, I would like to thank you for taking the time to speak with me today. My name is Chris, and I'm a NUS Year 4 nursing student. The aim of this interview is to get a better understanding of the perspectives nurses have on self regulating their health behaviors and how various factors may influence this. I want to find out what are your views, opinions, and experiences on this. I would really like to encourage you to speak what comes to mind. There are no right or wrong answers. May I have your permission to continue with this interview?**
- P13: Ah yes.
- **NJCL: Okay, thank you so much. So let's start with some basic questions like, what ward are you working in right now?**
- P13: Hey. So I'm working in [Public Hospital name] NCID ICU. So it's not in the main building. Rather, it's an infectious disease ICU, yeah.
- **NJCL: Could you describe the kind of work you have to do?**
- P13: Hmm, I care for patients who are admitted for mainly respiratory tract diseases. COVID, as well as other immunocompromised conditions where they require isolation.
- **NJCL: Can you maybe describe the workload?**
- P13: Sorry. Can you repeat that?
- **NJCL: Sorry, can you describe like, the amount of work you have to do each day?**
- P13: Okay, so basically. My day starts off with me taking over the case. And then I have to assess my patient's condition. Usually we take about 1 to 2 patients per nurse on shift, and so we have to ensure that all the prior documentation has been done. Conduct our assessment. Follow through with the changes that are required, as ordered by the medical team and administer their medications and deliver any treatment necessary within our 8 to 12 hour long shift.
- **NJCL: You mentioned 8 to 12 hour long shift, can you explain to someone who is maybe not in nursing why is there 8 and 12 hour shifts?**
- P13: Okay, so day shifts for my ward, day shifts are 7 am to 3 pm, PM shifts are 1pm to 9pm, and overnight shifts are 8pm to 8am. So the shift hours vary accordingly to what shift you are scheduled on.
- **NJCL: Okay. Thank you so much for that. Then my next series of questions will be regarding lifestyle in general. So, as a nurse, how would you define a healthy lifestyle?**
- P13: I think health is very multifaceted so it can encompass your physical health, your mental health, your psychological health. And your emotional well being as well. So in order to have a healthy lifestyle, all these aspects of health need to be addressed. That's my definition of health. Is that what you're asking?
- **NJCL: Yeah, yeah.** **Then, personally, for your lifestyle. Like, maybe talking about dietary dietary wise, how do you feel about your current lifestyle?**
- P13: Actually, I don't think my dietary health is very optimal. Number one, because shift work, is very- the hours are very varied. Sometimes, I, hmm, how do I explain this? Sometimes we don't eat at regular hours because of shift work and especially because when we are working in a ward based setting, there are no fixed hours for meals. So your shifts- so your lunch hours actually depend on what break you are assigned. So some days for example, I can go for lunch at 10:30, or other days. I can go for lunch, as early as 2:30 pm. For dinner-wise, the earliest break may start at 4:30 pm. And I will have to finish my dinner by 5:30 pm. Alternatively, some breaks, they are scheduled at 7:30 pm. So I can end- so I eat much later. Secondly, I think personally, I haven't really focus on having a lot of balance meals because of the hassle that food preparation takes. So yeah, I think the combination of these 2 factors are why my dietary health may not be the best.
- **NJCL: Then what do you feel will make you want to change your dietary habits to a healthier one other than what you mentioned, which is the hassle of food prep?**
- P13: Hmm. What will make me want to improve? I think, I think actually, I do want to improve my dietary health, but it requires a lot of conscientiousness. We have to always be conscious of what we're putting into our body, what we would like to prepare for next meal. There's a lot of meal planning and thought involved. But I think that as we work in such a stressful and taxing environment, I think I also believe that it is important for us to take- to not take dietary health for granted, and to also, incorporate a good balance of nutrition every day, so that we don't- we are able to sustain, you know, have a energy level sustained and manage our workload. And because I do notice that I get very lethargic easily sometimes if I don't eat very regularly, or if I eat very unhealthy food.
- **NJCL: Then, Maybe specifically on physical activity wise, how do you feel about your current lifestyle?**
- P13: I would say that my current lifestyle is relatively quite active. I do exercise outside of shifts. I visit the gym, and I engage in other recreational activities that are quite strenuous. Yeah. So in terms of physical health, I think it's better than average.
- **NJCL: Then, since you say, it's better than average, what do you think is keeping you to maintain this active lifestyle of yours.**
- P13: So what are the factors that make me want to live actively? Okay, I think I would just like- I'd like to explore a range of activities that are outside of my work. And I do find that sometimes nursing can be a bit repetitive. So I like to be spontaneous outside. And it is a personal preference. Yeah. And I I think I wouldn't enjoy being sedentary, outside of work. Yeah. So as physically tiring and demanding I think nursing is because we have to stand long hours. That's quite a bit of physical exertion involved during like turning patients, it’s still very necessary to engage in physical activity outside, to not only improve your physical health. But also your psychological health.
- **NJCL: You mentioned psychological health, can you maybe elaborate more on that?**
- P13: Okay, okay. So I think mental slash psychological health has been like a very rampant topic recently. Especially for nurses, because we do face lots of stress at work. So it's important for nurses to be mindful that- be mindful of our stresses and actively engage in activities that reduce our stress. So for me, that would be engaging in physical activity outside, like running, hiking, swimming, kayaking, and also to find a supportive group of friends outside of work. So you maintain that balance between work and personal life. So these 2 things, these 2 things- the activities that I engage in outside as well as my support system will really help me to maintain that mental health. Yeah.
- **NJCL: Maybe. Could you tell me more about how your lifestyle changed after you started to work as a nurse?**
- P13: Okay. So initially, when I started work, it was during the COVID- the height of the COVID pandemic. So it was 2020. There were a lot of restrictions in place. We couldn't really go outside other than to go to work. There were restrictions on exercise outside of home. So I think during that period, my physical health wasn't the best and we couldn't really socialize as much as well. So I gained- I gained weight and I felt very unhealthy, I felt that my mood was low. And 2 years later. I think about 2022, last year, when things, when activities began to resume back to pre-pandemic status, I realized the importance of being physically active. Yeah. So that's when I started resuming all my physical activity. Yeah, did that answer your question? Okay.
- **NJCL: Yes. How about like your mental slash psychological slash emotional health from before you started to work now? Did it change?**
- P13: Okay. I would say that my mental, slash emotional health was really quite turbulent. Especially during the start of work, especially when I transitioned from being a student to be a working adult. So because, as I mentioned, it happened during the peak of COVID it was really quite a stressful period. And being in a COVID ICU I was not fully mentally, and emotionally prepared for the amount of stress that I would face at work. There was a period of time after I started working where I felt that I felt a lot of self-doubt and I felt I was in very low moods. It was a duration of 6 months. And it was during my precepteeship. Yeah, so that was a very challenging time. And I almost considered quitting the field. But eventually things got better gradually. So I found some mentors at work who actually guided me through that time. I found my university friend group of nurses. We were very close, so we supported each other through that time. And I also managed to speak to a few of my nurse managers and let them know how it was doing. So they will check out on me regularly. So all of these helped me to get through that very difficult time. And now I feel much happier. Yeah, not just because we have now returned to pre-pandemic state. But then, also, because of the support system along the way. Yeah.
- **NJCL: And thank you. For my next question, I think you actually touched upon it with your answers here and there. But as a nurse, how do you feel about self regulating a healthy lifestyle?**
- P13: Hmm, okay. I think that self regulating a healthy lifestyle is really quite essential. Number one, because nobody can force you to be healthy. And everybody, I believe that everybody should take accountability for your own health. I do see nurses and colleagues who don't really regulate their health in the best way. And there are a lot of effects that come with time. So, for example, a colleague who does not exercise, and does, for example, permanent night shifts, she gets very stressed easily. And she has some hormonal changes, and she has to rely on melatonin to sleep. My other friends who don't really regulate their mental health or emotional health well, they get stressed very easily, and they get a lot of gastric issues which are stress-related which impacts them not only during their work, but it also affects their personal life. Yeah. So I think that it is very important for us to take accountability for our own health and not just our physical health, but also mental and emotional health.
- **NJCL: Hmm. Earlier you mentioned that it might affect their personal lives. Could you elaborate more on that?**
- P13: Hmm. Okay. In terms of general health and well-being, right? Okay, let's see, I'll go through aspect by aspect. So for example, I think in terms of physical health, if it's not properly regulated, or if effort is not taken to maintain physical health, often times at work, you might face a lot of injuries. For example, back strains, wrist injuries from the constant turning and patient maneuvers that we do which have- which actually happen more often than I realized when I first started. So all these injuries, they don't only affect work life, but they also affect your personal life because you carry those injuries back home, and they impact your ability to, for example, do household chores or to go out and spend time with your family and friends. In terms of let's say, your emotional, psychological health, if it's not properly maintained, if there are no- if you do not take active steps to ensure your mental and psychological and emotional well being, then you're more susceptible to stress at work. You're more susceptible to stress in general, whether it is family, whether the sources are family related stress, work related stress or other kinds of stress, and also affects your, I think, hmm. Your preparedness to any unforeseen circumstances, whether at work, for example the pandemic or whether at home, if we are facing a personal crisis.
- **NJCL: Okay, Very comprehensive. Yeah. Then my next question is. What are your main facilitators slash motivators in self regulating your lifestyle?**
- P13: Can you give me a second?
- **NJCL: Okay. No rush.**
- P13: [Long pause]. Okay. Actually what facilitates? So what facilitates me to lead an active lifestyle is actually, quite surprisingly shift work, because I do acknowledge that sometimes it's a drawback, because the hours are not regular, but at times it gives me the flexibility to be able to exercise during non-peak hours or exercise during hours where I feel more rested. Then let's say I would, than if I were to be doing a regular 9 to 5 job. And I like that shiftwork gives me this flexibility because I can involve myself in in range of different activities. So it helps to- so it actually, in a way does facilitate my active lifestyle. So for example, if I'm free on a random Tuesday morning, I could just go for hike if I wanted to. Yeah. But rather what what motivates me is more of friends? I do have a group of- I do have groups of friends who also share similar hobbies, interests. And as such we do- we align our lifestyles that way. I think that physical activity right, is rarely a solo thing. And friends really help to keep you motivated. Also, I think, personally, one has to be intrinsically motivated to involve themselves in physical activity. Whether be it for long-term health reasons or for aesthetic purposes. Yeah. All these are factors as to why I'm personally motivated to upkeep an active lifestyle.
- **NJCL: So earlier, you mentioned shift work, so would you say, shift work is more a facilitator than a barrier for an active lifestyle for you?**
- P13: Initially it was a barrier, but I found ways around it such that I involved shift work. Well, I managed to weave in activities in my shift work. So rather than having, for example, having fixed hours where I only can exercise at fixed hours, now I can choose what activity I can involve myself in at that particular time of the day when I'm available. So, for example, if I'm going for a night shift, then in the morning I am free to do- to go for hike or go for a swim, or something like that. Yeah. Rather than confining myself to maybe only going to the gym in the evening, where it's very, very crowded, because the post-work crowd; because the post-work crowd is there.
- **NJCL: Okay. okay, so far, we've been going through facilitators and motivators. How about any barriers in self regulating a healthier lifestyle for you?**
- P13: Hmm. I think barriers. Hmm. I think barriers would actually be the accessibility of unhealthy food in Singapore. Yeah. And also not only that, but the nature of how my friends and I socialize. Usually gatherings are centred around food. Meetups are at cafes or restaurants, or even hawker centres. So with every social gathering mainly centred around food, it's very hard not to fall into the trap of you know, consuming all these unhealthy food very regularly. Which is something that I find very challenging, because it is natural to, to want to take part as to what would be involved. So I'll- if I were to try to strike for healthier diet I feel like sometimes I might be unable to- I might be unable to hang out, as I would normally do. Yeah. Other than that, the convenience of, the convenience and accessibility of unhealthy food in Singapore really is a quite huge barrier. Yeah. I guess another barrier would be self-control. Yeah. Because I guess even with the convenience- the convenience can be there. But if someone has sufficient self control they will be less likely to consume food that are not nutritious. Yeah.
- **NJCL: Okay, can. I'll just summarize what you've shared with me. So for your facilitators and motivators, you mainly cited shift work as giving you the flexibility to ctually plan your activities. Then you also mentioned your friends motivating you, since they have the same interests as you. So they help to keep you motivated. Then you also mentioned personally, one has to be intrinsically motivated, either for long term health reasons or aesthetic wise. And then mentioned something on how shift work was initially a barrier. But now you're able to weave your activities in shift work. You have anything else you want to add for your main facilitators or motivators?**
- P13: I think- that's yeah. That sums it up, yeah.
- **NJCL: Then, for your barriers. You mentioned the convenience and accessibility of unhealthy food in Singapore, and also mentioned of how the nature of how your friends and you actually socialize together, it's mostly centred around food. And then you also mentioned having the self-control, like, even if the convenience is there. But if you don't have the sufficient self control you, you will still be likely to consume unhealthy food. Is there anything else you want to add for barriers?**
- P13: Yeah, correct. I think that’s about it for me.
- **NJCL: My next question is also something you already touch upon. But how does your personal self-regulation habits impact your work performance for you?**
- P13: Okay. Okay. So previously, I mentioned that I had it really rough during the COVID pandemic period, and that is also when my self-regulation mechanisms, weren't optimal and it significantly affected my work performance because I was constantly in a very lethargic state with a very low mood, and the overall, I believe, more unpleasant demeanor. I wasn't dealing with stress at work very well, and as such I wasn't able to handle complex cases independently not just because I was new, but because I wasn't able to actually process and manage the case as well as my preceptor would have hoped. There are also a lot of careless mistakes made because I was not really fully present mentally at work. And I felt that I wanted to often disengage. Oh. It took me a while to improve my performance and that was with a lot of guidance from my and NOs, my mentors at work, and a lot of support from my friends and family. Yeah. But eventually. with all these improved states of physical, mental, emotional health. Now, I think my work performance has actually improved quite a bit. And generally, I do feel happy at work. I feel that I am able to manage complex cases with minimal help and I also am involved in a range of projects outside the clinical aspect of nursing. Yeah. So all in all, I do believe that. My physical- my overall health has improved, and this has led to a significant improvement in my work performance. Okay, yeah.
- **NJCL: Okay, can.Thank you. So my next series of questions will now be involving the workplace. So as a nurse, how do you think the workplace influences your own self-regulation habits?**
- P13: Just to clarify, are you referring to the nature of work or like more of the social environment?
- **NJCL: More on like, maybe the physical aspect of the workplace, physical environment of the workplace?**
- P13: Sorry, could you repeat your question?
- **NJCL: As a nurse, how do you think the workplace influences your own self-regulation habits?**
- P13: Okay. Hmm. [Long pause]. Oh. Okay, I think because the nature of work, it is physically strenuous. It does make me want to upkeep my health in- during my non-working hours. So as a nurse, the activities that we engage in are quite physically demanding. So like turning, sponging patients, especially in the ICU with fully bedbound patients who can weigh up to about 200kg- I had a patient who was that heavy once. And it required about 5 nurses to lift him up. Yeah. So the physical nature of work, I would say really leads me to believe that I have to actually take care of my health. Because I do have colleagues who, they do get injured during work and because of repetitive, patient care activities. The constant bending down makes one very prone to back strains and back pains and knee injuries, especially because of the constant standing and walking around. So. Yeah, I think these are the ways that my work environment kind of nudges me to take care of my physical health. Yeah.
- **NJCL: Okay, then, moving on to like the physical environment, the next question will involve more of the social aspect. So how do you feel about the workplace culture among nurses in helping to facilitate or hinder the self regulation of healthier lifestyles?**
- P13: Hmm, okay. [Long pause]. Hmm. I say that? Oh, I think that this is very subjective, because I can only for my personal workplace. I think that my hospital, my ward have organized activities to focus on staff well-being and staff physical health. For instance, I think a month ago they had a Yoga session for nurses in my ward and I think the hospital also organizes yearly Netball or Badminton tournaments and they encourage employees of different healthcare groups to join and compete. Yeah, whether you are novice or you're slightly better at the sport. So that's for organization-led initiatives. However, because for nursing, it is very, It is very difficult to get all the nurses on the ground to be involved in one activity at a single time. Some of these activities do not cater to everyone. Because there definitely must be someone running the ward while others are participating. So I would say that it's a little bit difficult to get everyone, to be involved in the same kind of activity. So even with all these in place. I think that there's still a gap as to how the workplace can promote a better lifestyle for employees. More on the on the well being side, the sorry, not on the well being side but on the more staff-led initiatives, my ward does have a does have a committee. So a [Long pause, slight distortion]
- **NJCL: [Participant camera freeze] Hello?**
- P13: And they do organize activities such as fruit- like, they organize healthy eating parties for ground staff. Yeah. But I think that physical activity, in terms of physical activity, it’s more self-led. So it's more on how every staff participates in their own activities during their own free time. Yeah, that’s all.
- **NJCL: So, would you say that the workplace culture is actually beneficial in helping self-regulate your lifestyle. Like overall?**
- P13: Overall, I would say that there is a culture of healthy lifestyle. But it really is up to the individual, because all these activities are voluntary. Yeah.
- **NJCL: Then just now you mentioned the hospital actually like organizing a few activities to improve like the physical health of the employees. Have you personally participated in these activities?**
- P13: Actually, personally, I had not. Mainly because of constraints. Sometimes these activities are organized during my working hours, or sometimes they are organized during hours that are inconvenient for me because of my work schedule. So I guess that is the main barrier to participation in them. Hmm. So personally. I have not. But I think organization, the organization-led activities. I haven't involved myself in them. But for ward-based activities organized by my ward’s staff well-being committee, I have participated, and they do make an effort to include most staff. For example, holding multiple sessions over multiple days. So people working on different shifts can be involved in the activity. For example, the Yoga session that was held in my ward.
- **NJCL: Just clarify a bit. The ward-led initiatives were held over multiple sessions. But the organization-led activities were held only on a single day?**
- P13: They are more often than not a 1 or 2 day event. And if we were to participate it would not be included in our working hours. So I think that would disincentivize some people from joining.
- **NJCL: Perhaps, could you personally think of what will make you want to participate in more of these organization-led activities?**
- P13: I think I would be more incentivised to attend these activities if we were given protected hours. However, I do understand that that is a constraint, because it falls outside of scope of patient care. So, if I were to join an activity and request for a day to be blocked out, somebody would have to cover my workload. So that is, I think that is the biggest barrier. Because it is- Oh. It is not mandatory for people to join. So hence I think that is also a barrier.
- **NJCL: Could you maybe elaborate on how not being mandatory is a barrier?**
- P13: Because all these events are held on very voluntary basis. So people who are not inclined towards physical activity, or they rather engage in other less vigorous activities, they would not be very keen to join even if these activities do mention that they are beginner friendly sometimes. If I were in the shoes of a person who doesn't regularly engage in physical activity, I would not. I would not be very keen on these kind of activities. For example, all the various sports tournaments that are organized by the organization. I think some of them might only go if they are heavily pressured to. Yeah.
- **NJCL: Okay. Then. Going back a bit. Personally for you, what do you think about the role of nurse managers in helping nurses self-regulate their own health?**
- P13: I think most managers, they do play an important role. For instance, I think the main role of nurse managers in facilitating their staff's health is to number one, ensure that the staff get sufficient time and sufficient rest off because the main responsibility of nurse managers is to, hmm, organize the roster. So there are guidelines in place for them to follow. For example, there must be 2 off days after every 2 night shifts unless a request is made by the staff to have only one off day. So guidelines like that in place are followed by the nurse managers to ensure that the ground staff do receive enough rest post-shift so that they do have enough time to rest and recharge for the next working days. Secondly, I think another responsibility of nurse managers is to be sensitive and in touch with the ground staff. So if they notice, if they do happen to notice that any staff is not feeling well, or for example, their performance is not up to standard. I think it's essential that they talk to the staff or intervene. Or approach the staff to ask whether he or she needs any form of support from them. Yeah. So I think these 2 main roles, they are not directly involved in the staff’s help, but they do play a big role in ensuring that staff's general well-being is there.
- **NJCL: Hmm. Then we are to almost my final few questions. How do you think your institution can help you to self regulate a healthier lifestyle?**
- P13: Hmm. I actually think my institution can't do as much. Because I think that health should be- it's a very personal thing. However, I think the institution can facilitate staff to have healthier lifestyles. Personally, I would like them to maybe provide us with some form of… fitness facility? [Public hospital name] does have a gym, but it is relatively small, and it does get crowded at times. For me, I think at this current stage it is sufficient. But I would perhaps- ideally, they could maybe have a partnership with a gym and allow discounted rates for staff. Yeah, but I think overall there's not much, there's not much an institution can do other than having small things like that to incentivize staff to exercise and keep healthy. Alternatively- actually, they do already- they already implement certain activities for staff. However, that's on me, because I don't really participate in them actively. I think after COVID there was also the staff mental wellness day that they gave every staff. So I think. Yeah. That is what the organization does. And I'm not really asking for more.
- **NJCL: Other than what you mentioned about like the activities your organization actually organized, are there any workplace health promotion programs in place?**
- P13: I'm sure there are more than I know, but I think I haven't really taken an active effort to find out what they are. Yeah, for what I know- yeah,I don't really know much.
- **NJCL: It’s okay. Yeah. Okay, then I'll just summarize what you've shared so far, at least for the last few questions. So when I asked about how do you think your institution can help you. You mentioned that the institution can't really do much, because health is a very personal thing. Maybe they can help facilitate by doing small things to incentivize the employees.**
- P13: Yeah.
- **NJCL: Then, for workplace health promotion programs. You're not that aware of them. I mean you are aware there are, but you don’t specifically know what they are. Am I right to say so?**
- P13: If I could just amend that question [Participant laughs here]. Okay, hold on. I think those are very, I think, for the ones I know, they are very generic. So, for example, all stalls in the cafeteria are- they have the healthy eating healthier options. Those food vendors like [Bubble tea shop name] who have set up shop, they only offer, they’re only told to offer drinks, 50% sugar and below. Yeah, so these are a few small things that come to mind when I think of health promotional activities. But I don't really think that there's anything very large scale. These are all just very small regulations that in place, so that healthier options are available to anyone who visits the hospital. Okay. Mmm.
- **NJCL: Then, moving out of the workplace a bit, do you personally use any like tools or apps self-regulate your health or lifestyle?**
- P13: I actually do. I have an apple watch. So I use the apple fitness checker. And then recently, there was, there's an app called ‘Lumi Health’. It’s by HPB [NJCL: HPB: Health Promotion Board]. So you clock a certain number of steps a day and move a certain number of hours. and you clock your step goals, you are given points which can be converted into HPB vouchers. Yeah. So I link my apple, the fitness tracker to this app. and I can claim rewards from there.
- **NJCL: So you personally feel this, this is quite helpful in motivating you to move around more, or?**
- P13: It does. Because for instance, there is a 12 hour standing goal per day. So I have to hit it, if not, I don't get the points. And I cannot claim the money. Yeah. [Participant laughs here]. Yeah. So it's just a small- it's just a small extra thing. So every time I know I'm sitting down for a very long duration, I'm just incentivised to stand up and take a short walk and come back down. Yeah.
- **NJCL: Okay. And I see, okay. So for the question on like tools and apps, you personally use Apple watch that has an Apple fitness tracker. Then you also set this app Lumihealth, which is by HPB to Incentivize you to actually get money or HPB vouchers from doing small things.**
- P13: Yeah, from hitting the fitness goals that they set.
- **NJCL: Is there anything else?**
- P13: Oh, no that’s about it for apps.
- **NJCL: For apps. So you have other tools you mean, or?**
- P13: Other tools? Because not sure if this counts. But I have, I do set like personal goals for myself. So that also motivates me to hit the goals weekly, yeah.
- **NJCL: Could maybe elaborate on an example of one of these goals?**
- P13: At the end of the year in December, one of my goal is to run a marathon. So, I have signed up for the [inaudible segment] Marathon, which is happening on the first week of December. So I do keep track of my own progress week by week. So, in addition to these trackers, I do have a log of my progress. And I try my best to hit them. Yeah, in preparation for the marathon at the end of the year. Yeah.
- **NJCL: Would you say, having like personal goals actually motivates you to maintain your health?**
- P13: Yes, yeah, as I said, they definitely do because if not for these goals, I would be significantly less motivated to exercise. Yeah. And because it's something that I've committed to, it reinforces the need for me to stay healthy because I do have hit that goal that I set up.
- **NJCL: Okay. Do you have anything else that you would like to speak about that was not mentioned in the interview? Any further comments, you want to share?**
- P13: Not that I can think of.
- **NJCL: Okay. And then this marks the end of the interview. Thank you for your time and participation.**
- P13: Okay.
- -End of P13 Transcript-

# **Interview #14**

Participant and interview details
 Participant number: 14 (P14)
 Date of interview: 1 Oct 2023
 Interviewer Number: Navarro John Christopher Lambino (NJCL)
 Time IDI started: 1000
 Time IDI ended: 1055
 Observational notes: Participant seems prepared for the interview while still in her uniform.

Section I: Interviewer’s summary
 The individual interview was conducted via Zoom. P14 has just completed her night shift. The interview was planned for 0900 but she was delayed on the way home, requested the interview be delayed until 1000. For context, she has transferred internally 2 times, finding a better workplace environment for her. She also mentioned having stress induced hormonal imbalance due to her experience in the other previously unsuitable wards she worked in. There have been many similarities to what other participants has mentioned. Shift work, manpower issues as major limitations. Points of interest however are: culture of nurses prioritising patient care instead of their own health, this can be seen in how nurses do not go for breaks; having less time constraint means more time for oneself; lack of control and choice over one’s time since it is decided by rostering. Lastly, not enough emphasis on each one’s personalized needs. Current measures ineffective for P14.

Section II: Transcript

- **NJCL: Alright. Good morning, P14. Firstly, I would like to thank you for taking the time to speak with me today. My name is Chris, and I'm a NUS Year 4 nursing student. The aim of this interview is to get a better understanding of the perspectives nurses have on self regulating their health behaviors and how various factors may influence this. I want to find out what are your views, opinions, and experiences on this. I would really like to encourage you to speak what comes to mind. There are no right or wrong answers. May I have your permission to continue with this interview?**
- P14: Can.
- **NJCL: Okay, thank you so much.** **Let's start with some basic questions like which ward are you working in right now?**
- P14: Okay, I'm currently at [Public hospital name]. I've been working for 3 years. I started off in a cardiorespiratory ward for 2 and a half years, and I recently transferred to acute medical ward for short stay patients.
- **NJCL: Can you describe the work, like what do you do on a daily basis?**
- P14: Hmm, okay, so I provide basic nursing care. I am a staff nurse, so my routine usually revolve around parameters taking, caring for daily ADLs, like changing diapers, feeding patients, and also administering medications as ordered. And also do appropriate assessments as and when doctor orders, such as neurovascular assessments. And also facilitate care between nurses, doctors, and allied health professionals, PT, OT medical social workers.
- **NJCL: Could you describe your workload?**
- P14: So actually, when I was in [Public hospital name] main block, that was a much bigger ward, so the ward had around 30 to 40 patients. So when I was in main block, my workload for morning shift, would be typically 1 cubicle. Which means 1 nurse to 5 patients with accompanying junior which is an enrolled nurse or HCA. But when there is MC, when there’s not enough people, I will have to take 2 cubicles. So that means 1 main nurse to 10 patients, with an accompanying junior or enrolled nurse. Or if there’s not enough enrolled nurses, then you take 1 cubicle. I will take 1 cubicle for myself. So me to 5 patients alone. Night-shift wise, when I was in the main block, it’s ranges from 1 of me to 7 to 8 patients. And if there is any MC, or lack of manpower, I will take 10 to 15 patients at night. With an accompanying junior as well. But over here, because my current ward is a lot smaller, we are single room 13 bedded ward. So my maximum workload is 1 nurse to 7 to 8 patients with accompanying junior. So it’s a lot better currently.
- **NJCL: So, the ratio is a lot more sustainable, would you say?**
- P14: Yeah, I feel like it's a healthier workload, I would say.. Currently I feel that the workload is a lot healthier and I feel that I am actually providing quality nursing compared to when I was in main block. And the workload was 1 to 8 to 15. Back then right, it was very touch and go, I could barely provide or interact with my patients. It was pretty much diaper, para, meds, diaper, para, meds. Like you finished changing the diaper, next thing you know, it’s time to change diaper again. So I felt like care back then was very stressful and I didn’t have a lot of time to actually higher order thinking. Like I didn’t have time to sit down, look at the plan and analyze if this plan makes sense for the patients, suggest quality improvement projects, or suggest like how can the patient care be improved. Because there’s just enough time for you do to everything that’s basic. And then it’s time for you to go home.
- **NJCL: I see. Okay. Thank you for that. Then, for my next few questions, it will be regarding the lifestyle in general, so like as a nurse. How would you define a healthy lifestyle?**
- P14: Hmm. I feel like a happy lifestyle would be a balance lifestyle, a good balance between work between personal life and between rest. Yeah, that's what I’d define a happy lifestyle. So, hmm, that means, let's say for me, I like to look at time as ratios. Let’s say we have 24 hours, 8 hours is at work, then there should be an equal time, which is like another 8 hours, for you to do that is something that is personal enrichment, then another 8 hour for rest. That's all I would say for balance. But honestly, it is very unrealistic to live your life in junks of threes like this. And also because of shift work. So yeah. Our hours are constantly shifting around, then some days you will find that you have a lot more time outside than at work. Some days, you find that you have very little, 3 to 4 hours rest in between your shift.
- **NJCL: Could you elaborate what you mean by like, the not enough time in between the shifts?**
- P14: Like for my afternoon shift, it’ll be from 1 to 9. Then my morning shift will start at 7 am. So if I am afternoon to the morning transition, so when I work till 9, I will get home, maybe around 10 to 11 to take care of like additional changes, any issues during my shift, and like transport hours. Then by the time I get home, 11. Shower, go to sleep, 12 midnight to 1 am. Then the next day you have to wake up at 5 am again to prepare for morning shift. So the time between that is very short, and because the roster is so unpredictable, so you have PM to AM transition, you also have AM to PM transition, where it’s a huge chunk of time in between the two shifts. Then also you have night shift, then between the two night shift, you end work at 8 am. Then that same day, 8pm, you have to show up for work again. So, it’s again a very short period of time when you get to rest between your 2 shifts.
- **NJCL: Can, understand. Then, still talking on the topic of like healthy lifestyle specifically, on your diary habits. How- How do you feel about your current dietary habits?**
- P14: I feel that I am not practising what I'm preaching and telling my patients to. Honestly, back when I was at main block, right, breaks are a luxury. You don't really get to eat or drink water, or go toilet during your shift, so it's very much like the whole day, you just kind of eat one or 2 things. Yeah. And we like to cut down wasted time. And because our break is so short. So we have to move out of the ward, go down to buy food. Wait for the food to be made and then, go back down to the ward then you can sit down and eat. Then it’s like 20 to 30 minutes of your break has already gone just by getting the food. So a lot of times I just fall back on to convenience store food like 7/11 or prepared or processed food and pre-shift also because like PM to AM transition there’s very little sleep that you get. So I rather sleep more and spend lesser time preparing food, so things like instant noodle, processed foods or like ready-made food are my constant day-to-day diet like to the point that I have a U-card, just so I can get more points for 7/11 [Inaudible segment] whenever I buy anything in the 7/11 at my hospital.
- **NJCL: Would you say that, right now, currently, you're not really satisfied with your diet?**
- P14: Not at all. [Participant laughs here]. Yeah. I feel like I’m eating a lot of carbs, a lot of processed food. And I’m not getting the fiber that I would like myself to get. Because at the convenience stores, it’s not easy to get fresh salad at a cheaper price so like you find fresh produce are a lot more expensive as compared to sandwiches or packed food.
- **NJCL: Also, earlier, you said something like, you're not practising what you are preaching. Can you elaborate more on that?**
- P14: I feel like as a healthcare professional, you always tell your patients like, ‘oh, you need to eat less salt’, ‘you need to eat more vegetable, you know’, ‘having a balance diet’, ‘drink more water’. In your patients plan, you always see that they encourage patients to drink 1.5 litres to 2 litres of water a day for a healthy normal adult, but yet I feel like all of us healthcare workers are barely getting half a bottle of water a day if we are on shift. And personally holding our bladder longer than most people. And most of us are actually very prone to having UTIs or having GI problems.
- **NJCL: Hmm. Then, currently, do you feel like changing that diet?**
- P14: Sorry?
- **NJCL: Do you feel like changing your diet?**
- P14: I have tried a few times, but it’s very hard to stick, because the lack of time to prepare meals is a very big problem. And because I’m a foreigner living in Singapore, and I rent a room in a flat, and you don’t have the luxury of your family cook for you or preparing meals in bigger portions. You only have you to cook for yourself. I feel to me personally, the time spent preparing the meal, I rather prefer to spend that time resting, because I feel I'm constantly sleep-deprived in between shifts. So that's why I always hold back on to like very convenient, very fast and more unhealthy diet, I feel. It's been quite hard to change, to be honest.
- **NJCL: As in right now, it's quite hard to change because, the availability of convenient food is better- like you prioritize your rest more than actually preparing for this kind of thing.**
- P14: Hmm, yeah, I feel like it's convenient. Yeah, and also cost wise, also. And as well as the lack of time to do meal preps, all these.
- **NJCL: Okay. And then, being away from diet, how about your physical activity? How do you feel about your current physical activity?**
- P14: I feel actually, that's not too bad, like, I feel like I’m very active at work. Like, I walk a lot, and there’s a lot of diaper changing, so I’m constantly sweaty. I feel like I am physically active as compared to let’s say, an office worker, and I always manage to clock in at least 5000 steps a day, being in shift. But the only struggle, or the only difficulty, that I have is to go beyond that. I feel like this is my current baseline of activity level. But if I want to lose weight, or if I want to even become healthier, or have higher stamina, I will need to spend time at the gym, or doing higher intensity activities. So those are, those are the things that I struggle to find time to do. Yeah.
- **NJCL: Is it right for me to say if you had maybe more time, you will be motivated, change your physical activity levels?**
- P14: Yeah I feel like time is a huge factor for me, in a lot of my lifestyle decisions, and honestly right, the shift work- because you work 5 days a week and you have 2 days off, but most of the time the roster is not realistic this way. Because majority of the time, they like to put your off days after your night shift. So technically, you only get 1 day off. And that is if your lucky, that you get 2 off after your night shift. Some of the time they put night, off, your back to work, and then they put you on off again. Yeah, so I feel like, off time is very precious. And if I want to let’s say, do their e-learning, have any career progression by going through projects outside of work, then things like my diet, things like going to the gym, get kind of pushed down to a secondary priority.
- **NJCL: Hmmm. Then, just moving on a tangent with what mentioned, what do you think will make your own health or your own lifestyle, more of a priority?**
- P14: I feel that, if I have a choice to have more flexible working hours. If I have more rest time, or if I have, like secured sleeping day after my night shift, that will help because I can portion out my time more evenly and it doesn’t feel like my rest day is very cramp in between shifts. It won’t feel that I’m constantly scrambling to find time for other things like my family, my loved ones and other priorities of my life. Because I feel like, my own perception of time is currently, my rest time is so limited that it almost feels like a waste to spend it on myself. That’s why it keeps getting pushed down, pushed down in terms of priority because meeting friends or doing projects to secure my next career progression, these seem a little bit more important at this juncture of my life. So I think like if I had a more protected rest day, that is a little bit longer and not so segmented, like after night, you get a few hours and the next day your back to work. Like today I’m night, then tomorrow I’m morning shift again. So it’s like I only have today, then by 10pm to go back to bed and prepare for tomorrow. So then, I don’t dare to sleep, you know? So I can squeeze just a little bit of doing this with you or meeting with friends. Then things like gym-ing all these doesn’t seem as important to me.
- **NJCL: Okay. So far you've been sharing on your diet, on physical activity wise. How about your current emotional, mental well-being? How do you feel about that aspect of your current lifestyle?**
- P14: Currently, I feel like I'm in a much better place as compared to when I first started out. But that is after 3 years of being in this line and like and having shifted my work environment- this will be my second transfer in my work. Yeah. So I went through a lot of trials and error, a lot of experimenting to try to find what works for me. But initially, honestly, it was very difficult. Especially from a student nurse to a full-fledged staff nurse was very anxiety-inducing. Especially within the first year, I remember that I had very regular mental breakdowns. I had anxiety before coming to work. I had trouble sleeping, and like what is- whenever I close my eyes, I kind of like get stressed from work. Like, I keep replaying mistakes I made at work as well. So it was not a good experience at all, and I think it was very detrimental to my mental health at that time. I felt like, I went through the first 2 years of my nursing life, thinking whether I should actually continue being a nurse. Like the thought of quitting actually came very, very often. And when it was very bad, like let’s say, when my patient workload is very high, is to the point that every shift I came to work, I kept thinking ‘this is not for me’. I rather like, I would really like to quit this.
- **NJCL: So this was your experience when you transitioned from a student nurse to a full-fledged staff nurse?**
- P14: Yeah. When I was back in main block where the workload was a lot higher. And I was in a cardioresipiratory ward, so my patient acuity was very high, we had patients collapsed every week, like code blue is a common thing for us. And adrenaline rush was always there to the point that everytime I ran a code blue, I ran a resus, right, the next morning, like the next day when I shower, my hair would start falling out excessively. Like I could feel the stress putting a toll on my health. And like I would not have proper appetite, and I went through lots of hormonal issues because of this. And already after I come out and transition here, then when I seeked treatment because I thought it was stress-induced symptoms whereby I couldn’t have menses for many many months, my hair keep falling out, I gained weight and lose weight very drastically. Then, it was diagnosed that it was stress-induced.
- **NJCL: But now your current lifestyle is a lot better?**
- P14: It's a little bit better because of the more balanced workload. But I have transitioned here for 1 or 2 months. But yet I'm still experiencing the health issue that I accumulated in the past. Like, I'm still on medication to like regularize my hormones and things like that
- **NJCL: It's gonna take a while for you to actually go back to having a healthy mental well-being and all that?**
- P14: Yeah, my normal levels. Also the doctor said that, it might 6 to 8 months for my hormones to stabilize and for me to be able to wean off the medication that she has put me on. Yeah and she thinks its very much because of my previous working experience and the level of stress that I was exposed to. Actually, a lot of my friends in the cardiorespi department, they actually developed heart issues as well, yeah. Like, a lot of nurses have gastritis, very common. UTI, very common. Then a lot of them also have palpitations. Some of them are diagnosed with SVTs, things like this because of the stressful environment. Like, whenever they have a stressful work, then they realise that they have palpitations and when they go for workup, then they realise that they are having arrhythmia related to the work.
- **NJCL: Wow. I see. Hmm. Okay, actually, for my next question. You've been saying bits and pieces of it, like the answer here and there. But, as a nurse, how do you feel about self regulating healthier lifestyle?**
- P14: I feel like it's a lot harder than I thought it would be, because of like, I have so much medical knowledge. I know exactly what needs to be done. But yet, at the end of the day after, like a tiring shift, right- I mean for me, I have 0 motivation or 0 energy left to do anything else for myself. Yeah. So a lot time I find myself completely drained after work, to like better myself, in a sense, yeah. It constantly feels like I'm giving, giving, giving and I don't have enough left for myself. Yeah. So that's something I’ve been trying to work on, coupled with the lack of rest time because of my shift hours.
- **NJCL: Could you maybe elaborate on like, how you're working on this?**
- P14: Hmm, so finding a good environment for me was what I really tried for very long, like transferring departments, finding one that is more balance, finding what I prefer. So when I was very busy, then I found a less busy department. So I went from a cardiorespi ward to a general medicine department because I thought that it will be a lot less acute. Like the acute care will be lesser. But then, after that, I realize that the patient workload- the patient to nurse ratio is still bogging me down. Even though the patient acuity is lesser but because one to so many patients, I still couldn't cope well, and I still couldn't find like a good mental health balance. So I transfer to an area where there is a better patient-nurse ratio. So experiencing the different environment and pinpointing what works for me, what doesn’t, and balancing between what I enjoy in my clinical work, as well as what I need for myself to have a more balanced self is what I focus on at this point of time to so called, change my lifestyle and better my situation.
- **NJCL: Okay, thank you for that. My next question also answered a few bits in there. So for you, what do you think are your main facilitators or motivators in self-regulating a healthier lifestyle?**
- P14: Regulators and motivators?
- **NJCL: Yeah, yes.**
- P14: I think one major factor, that for me, is my health, my health situation, because I have been diagnosed with hormonal imbalance. So it's a very objective finding for me to keep in mind that if I don't change, or if I don't think about this, this could be a medical issue that I will have for the rest of my life, and that's just not something that I want to live with. So that is the main motivator. So this one of them, to change my lifestyle, find a more balanced lifestyle and hope that is less like, less stressful and also more balanced. Yeah, so that is one of the main motivator for me right now. Another thing is money, because it's actually linked to the first one as well. Because actually, medical care is extremely expensive, given that I’m a foreigner. So I'm burning off all my medical benefits, and like a lot of bonuses on treating the disease I got from work. That just doesn’t make sense for me. So that’s another motivator that I have. Then the next one would be, at this point of the time, after 3 years I realise that maybe, I don’t want to spend the rest of my life in a three three three segment. Perhaps I want to sleep in a little bit more, enjoying life than spending it at work. So it was a shift in mindset for me compared to when I first started out. When I first started out, I was a bit ambitious, I wanted to head straight towards APN route. I wanted to be big in my career. But at this point of time, I realised that I placed a bit more importance on enjoyment, on taking time off, on having rest and I am willing to do that at a little bit of the expense of my career progression. So yeah, I think the shift in mindset is motivator for me to gear towards a change in lifestyle that I’m heading towards now.
- **NJCL: Okay. So far you shared with me like you main motivators. How about your main barriers in self-regulating?**
- P14: My main barriers, to be realistic, is the fact that I haven't been able to just like get out or, like, like, you know, take actual rest for myself would be logistic issues like having a job security. I'm not a Singaporean. I’m not a PR.So I need a work pass to remain in Singapore. And also to transition out of this industry or to actually try new things in this industry has been quite difficult because of the hierarchy in place. So, for example, let's say, I find that I'm not suitable for the ward environment, because it's too fast paced for me,workload is too heavy. If I want to transfer to a clinic setting. It takes a lot of hoops for me to go through to actually get to experience that and finding what works for me. And a lot of my friends also have problem transferring out or finding a place that they would like to see whether it suits them better. Hmm, so that's another barrier. Then the next one is what I already shared with you; the time issue, the rostering, the lack of control over our roster. Like everything depends very much on the sister and how other nurses plan their rosters as well. Like, we need to plan our annual leaves one year in advance, you cannot clash your annual leave with other nurses who might request in the same spot as you, and if lets say the other nurses, the pregnant nurses, they are going for marriage leave or they are going for like major hospitalisation leave, then all your plans would be affected. And then, you lose the control over your own time.
- **NJCL: Okay. Then I will just briefly summarize what you mentioned for this question. So the question on like, ‘What are your main facilitators, motivators, and barriers in self regulating a healthy, healthier lifestyle?’. You mentioned that your health situation, because currently you're having a hormonal imbalance. It's objective way of you actually keeping track of your health. Then, money is another main factor, because it's actually extremely ex expensive to go for all your, this medical care for your current situation.Then so mentioned that there’s a shift in mindset. You are now gonna place more importance on yourself compared to career progression. Is this right so far?**
- P14: Yes, it is.
- **NJCL: Okay. Is there anything else you might want to add for motivators?**
- P14: No, I think that’s it for now.
- **NJCL: Then for barriers, you’re saying that there are logistical issues because of the fact that you’re not Singaporean, and actually, transitioning out of like nursing is very difficult. Then there's a high hierarchy system that makes it difficult to transfer out to a more suitable working environment. And then, lastly, the time issue which is that there’s a lack of control, because of the rostering in charge will be the nurse managers and how other nurses plan their time will clash with yours, and then you don’t have enough planning involved to come with your own time.**
- P14: Hmm!
- **NJCL: Is there anything else you might want for barriers?**
- P14: I think that’s about it.
- **NJCL: Quite comprehensive, thank you. Then, my next question would be, how does your self regulation habits impact your work performance?**
- P14: Hmm. I feel that- okay, so this is me comparing my current self with a bit more improved self-regulating habits compared to my past self in the work environment where I was not prioritising my health and was going through a lot of stress. Currently, I feel that my nursing care, my work performance has improved. So my nursing care quality has improved significantly when I have a bit more control over what I want to focus in. Like for example, back when I was in a very stressful, very fast-paced environment, and when everything was just task to task to task, I didn’t have enough time to do what I was truly good at, in my own opinion, which is critical thinking to actually analyze the care plan, improve the care plan. I didn’t have the time to do that. But currently when I have bit more balance in my workload, I feel that I get to learn more, I get to interact more with my fellow colleagues, I get to interact more with my doctors and actually get to enrich myself with medical knowledge that I didn’t have the chance to learn prior and then that makes me a better nurse overall because I can improve my patient education, I can improve on my suggestions for the next patient’s care plan who may be similar to the patients I have encountered before, and I feel like, I find myself giving more quality suggestions to the medical team more often in my current workplace as compared to last time, where everyone was so busy you barely have time to talk to anyone throughout your shift. So with better self-regulating habits, or mental state, I feel like my work performance improved. Another area is that, I have more capacity to actually take part in clinical improvement projects as compared to last time. Yeah, I find that I have more spare energy, outside of my clinical tasks to actually progress nursing profession as a whole or progress my workplace, in terms of the workplace environment and not just my own practices. Then another thing is that, I found myself to be more pleasant to be around in terms of like as a nurse, a person, as a friend. Like, I was very high-strung back when I was in the very stressful environment. And then now, when I regulate myself a little bit better, I’m in better control of my emotions, and my behaviours I would say. And I have more energy to attend to other people in my life beside patients. Yeah. I think these are the main differences I have noticed.
- **NJCL: Your last point you were saying, like, you have more energy to attend other people in your life beside patients. Does this mean outside of work as well?**
- P14: Yes, outside of work, yeah. And also like as a fellow colleagues like in other capacity besides as a direct nurse to patient relationship.
- **NJCL: So will you say that your self-regulation habits impact not only work performance, but also your life in general?**
- P14: Hmm, I would. I would say, so
- **NJCL: I see, okay, okay. And alright. Thank you. Then, for my next few questions it will be regarding the workplace in general. As a nurse, how do you think the workplace influences self-regulation habits?**
- P14: I feel that the workplace actually has a lot of control over employees’ self-regulation habits, because we spend a significant amount of time in work environment, like one quarter of our day is at work. So if you extrapolate that out, it is essentially one part of your adult life is at work, so you have to acknowledge that a lot of factors at work will be influencing you as a person, and will be influencing your lifestyle as well, even how much time you spend. So because, I feel like in our culture as well, there's a lot of emphasis on career progression in being a good employee in hmm, having a good performance overall at the expense of your personal life or at the expense of your health, at the expense of your self-regulating habits. Yeah, so, if I talk about specifically like what I mentioned is mainly is the rostering issue. I don't have control over my time. Yeah, I don't have the choice of when I want to visit my family. I don't have the choice of whether I have enough time to meal prep. Yeah. And I don't have the control of like when I can do certain things, I cannot book classes in advance, and a lot other aspects of society functions in a lot more predictable rhythm than shift work. Yeah. So let's see if I want to go for a gym class. I want to go for a physical engagement class. Usually they will be on weekends, Saturday, Sunday, but I can never, promise someone that I will be available on that day or time. Yeah. So I think that takes away a lot of choices. And it takes away a lot of motivation for me, myself, to engage in these kind of activities. Yeah, because commitment is a real issue outside work for me.
- **NJCL: I see. Okay. Actually, you already touched upon some of the answers for the next question. How do you feel about the workplace culture among nurses in helping to facilitate or hinder self-regulation of healthier lifestyles?**
- P14: There's not enough emphasis on this. Yeah, like, I feel like as a nurse, there's a little bit of friction between what you would deem as a good self-regulating habit for yourself versus what your manager may deem as suitable as a regulating habit for you. Like I've had managers who tell me ‘you can just sleep on your off day, what, why do you need your off day to do something else?’. Like lets say the manpower is down, they call me back for work, when I’m on off day, so I tell Sister ‘Sister sorry, I already made plans’. Then Sister will say ‘How can your plan be important? We need you back at work.’. Something like this, there is a friction between what other people may deem as important and what you may deem as important. And I think there is not enough emphasis on healthier lifestyle- on the personlisation needs of each person in terms of the way that they regulate their own habits. Like how I view a good regulaized lifestyle maybe is that I just need 1 day a week to go to the gym. That’s good enough for me. I can work 6 days a week, no problem. But someone else may not be okay with that. Someone else may need 2 or 3 days of like scattered out engagement and they may need a more different approach of how they might spend their time. So things like this, I feel that there is not enough, leeway in the workplace and I feel like in the workplace, the big important thing that we all have to cater to is manpower problem, is the fact that the shift work demands us to work this kind of hours and that if you don’t show up, or if you voice out the need to have more flexible schedule, you are deemed as a difficult nurse or a difficult employee to deal with. Or like if you ask for special roster request. So that’s why a lot of nurses actually don’t dare to speak up or they will just take MC for the things that they want to do. They will just swap amongst themselves because they don’t care come to Sister or the manager for this kind of like request.
- **NJCL: Hmm. Actually you have been talking about nurse managers quite a bit. What do you think about the role of nurse managers in helping nurses self-regulate their own health?**
- P14: I feel like, because nurse managers are the direct supervisor of a staff nurse, and they are the one who plans the roster, so a lot of time, our friction is directly with them. Yeah. So for them, I know that they have to juggle between like organizational needs, the patient needs and their staff needs as well. But I feel like a lot of them- like my own personal feeling is that they prioritize the organizational needs over the employee needs most of the time. Like. If you let's say, I have a fellow colleague who went through a little rough patch and required mental health help, like medical care. So usually like that will require a long leave. Yeah, like 2 to 3 months of like leave just to take out of work and go for treatment. That became a big problem, because when a nurse take leave for 2 to 3 months, who's gonna work for extra shift? Yeah. So after that, for the nurse manager, their main issue is fulfilling the manpower needs because of the current situation and the very high, patient loads that we have. I think, what they can do better to encourage nurses to be healthier people, to regulate ourselves better would be to understand the specialized need that different people may have. And I feel that, more can be done to make sure that the nurses stay healthy. There need to be more ground engagement, I feel. More checkings with the supervisors, and if only they they themselves, can get a little bit of allowance with the manpower- I’m sure that they are actually good people, they don't mean to like restrict other nurses, because their hands are also tied. They only have so many nurses that they can deal with.
- **NJCL: I see. Then, okay, moving back a bit, just some clarification on what you mentioned regarding the workplace culture among nurses in helping to facilitate or hinder self regulation of healthy lifestyles. For you, you mentioned that what's hindering is that there's not enough emphasis on the personalized needs of every nurse, which means that they, they need to be able to understand that each nurse have different approaches on how to spend their time. There's not enough emphasis on this, and you also mentioned on how for nurse managers, their hands are tied. They will usually prioritize organization needs over their nurses needs. In the sense they are so like hindering the self-regulation of lifestyle of nurses, albeit reluctantly. Then you also mentioned rostering also plays a part in actually hindering self-regulation. So is there any way the workplace culture among nurses actually facilitates healthier lifestyles?**
- P14: I'm sorry again.
- **NJCL: Does- Do you think that the workplace culture among nurses actually does anything to facilitate self-regulation of healthier lifestyles?**
- P14: Hmm, I think so, yeah, I strongly believe that if you, if you can find a good workplace culture, you will be a lot happier at work. It will make you a better or happier employee, which then eventually leads to a happier person, yeah, because you spend a lot of time at work, like what I’ve mentioned. So I think, I can draw comparison between what I experience personally, and versus what my friend has experienced. I've been very fortunate to have a very young workplace that I started in. So most my colleagues are around my age, they understand and share the same interest in what I find interesting, and also they understand and share the same sentiments as what is a healthy or balanced work life. Yeah. Versus a friend of mine who started work in a place where her colleagues are generally older, like 20 to 30 years older, different generation of nurses altogether, and there was a barrier in communication. They don't share the same interests, and because of this, what she finds is healthy lifestyle or more balanced lifestyle is completely different from what her colleagues may find. So because her colleagues are a little bit older, they have families. So for them regulated life or more balanced life will be to have enough time between work and family. Yeah. And self care is a bit lower on the prioritisation for them so the way that they approach each other, like let's say, if my friend wants to swap shift because she has a, let's say a Zumba class, then she will feel judgement from the rest of them, where they view that this is not justified reason for you to swap leave, and because what they view as important is like family or work itself, whereas for me, when I have a work place of people that can empathize with me and encourage me to pursue my same definition. My definition of a balance life, it is a lot easier to easier to feel accepted. It’s a lot easier to speak out and to be able to pursue my own definition and my own, like specialized ways of like what I feel is balanced. Yeah.
- **NJCL: It's like having a social circle to actually help encourage you to self-regulate yourself better?**
- P14: Yes, correct. Yeah. Like having a community that kind of gets you. I think that kind is very important. And also a big issue, I feel, is like, the lack of break time at work. So if you are supported with community that that you can relate to, things like helping each other, team work comes very naturally, very easily to you and doesn't feel very forced. Yeah. So when things like this are in the work culture, it's a lot easier for you to feel empowered, and to feel healthier in a sense that I can actually take some time off to have a meal, to go for a toilet break, because you know that your colleagues will take care of you versus an environment where you are not sure, or you don't trust a colleague, then it's a lot harder to hands off because patient care, you know? There's like real consequences to real people, and we are always always very scared to hands off, yeah.
- **NJCL: Okay. Hmm. And so just to briefly summarize. For you having a strong, positive workplace culture in terms of having a social circle that can relate and support you, will actually help you to self regulate your own lifestyle because being in a more supportive environment means becoming happier and better nurse and a happier person overall. And they can also motivate you to engage in activities plus having this supportive social circles also means that they will be able to provide help, or work in tandem together more efficiently.** **So this are mostly for the facilitators. Okay. I see. Okay. Just going back a bit. There was one part where you mentioned: ‘there's a lot of factors that will influence you and your lifestyle, and in this culture as well, career progression, being a good employee’, is is sacrifice in favor of- I mean, career. progression is prioritized over your own health? Will you elaborate on some on that?**
- P14: You mean the cultural aspect, is it?
- **NJCL: Maybe nursing in general, like how their health is not as prioritized when it comes to maybe patients health or their career progression?**
- P14: Hmm. Well, I think a major issue that I found myself struggle with is taking break during shifts. Yeah, like back when I was in the main block environment whereby patient acuity is very high. So, for example, if the patient blood pressure is low. There's no way I'm gonna go and eat, and just like let it be, you know? So I will do a thousand other things before I think about whether I'm actually hungry or not. Yeah. And before I know it, the shift is over and I haven't eaten, I haven't gone to the toilet for over 8 hours. Yeah. So when it comes to patient care, like, I feel a lot of nurses are very scared because clinical setting is very unpredictable, and you don't want to be the reason why or be the contributing factor to someone’s deterioration. And that, and I think that being a healthcare worker, I feel like most of us are altruistic, to a certain extent. Where we will prioritise someone’s- okay not say say prioritize, we will feel that someone’s well-being is very important and sometimes we will prioritize that over ourselves because we always feel that ourselves as the able-bodied person, you know? You are a nurse, you are caring for someone who is sick, who is ill. So you are in a position of an able-bodied person and therefore you can afford to “neglect” yourself a little bit, neglect, in a very rough sense, to care for the other person who is sicker. So this is an area where I feel that it is very common for nurses to not go for break and the reason behind it. Then, what else? Let me think… Oh, and also regarding culture wise, right, like what I’ve mentioned, the manpower issue is a big issue with the nursing workforce, and how management struggles a lot with manpower. Also, there are certain times where you can actually spot patterns of favouritism between managers and staff nurses. And some of these patterns correlate to how regular, or how willing a nurse is willing to come back for shift when someone else MC’d, or when the manpower needs require. So when you see enough patterns like this, you start to actually think that it’s okay for you to not have an off-day, or it’s okay for you to not have enough rest to come back and help the warwd and put the organizational needs before yours, even though you may not be well enough to work. Yeah, I feel like it's very like, very, nuanced signalling in the workplace.
- **NJCL: So you feel that most nurses would actually put organization needs over their own needs?**
- P14: Hmm, yes, when it comes to patient care, like all the nurses that I have encountered will always for patients care over their own and yeah, like. They always put the patient care tasks above the breaks, above their own health needs. Hmm. And also like this is very enforced in health care. Because let's see if you go for a break when your patient is unwell, your colleagues will not be happy with how you handle it as well. So it's just kind of like a cycle where it forces like upon each other.
- **NJCL: Hmm, okay. So actually moving on to like one of the very last questions already how do you think your institution can help you to self-regulate a healthier lifestyle?**
- P14: Hmm. Well, this is the million dollar question, you know. Like, if we can answer this, and would really be in this situation? Yeah. But I do have to give props to MoH organizations to try to target or try to solve his problem. I do realize that ever since COVID rolled-out because during COVID duration a lot of people leaves got cancelled. You are working the whole year without having a break. So things like being able to in convert our leaves to cash- turn our ALs into cash. Having more bonuses and also rolling out more programs like more health related programs like, now, my hospital, have a Zumba class if you want to sign up for it, and we have very discounted Gym membership rates. Yeah. So things like these are signs that actually, they do care about us. But I think how the solution that they come up with doesn't really quite target the main issue with what I face, which is the rostering issue. So I feel like they are rolling out options for me to regulate myself better. But it doesn't solve my issue, which is the fact that I don't have time to do any of this. Yeah. So having the option doesn't really do much for me, but it looks very good when you look at it as like an effort. Yeah.
- **NJCL: Hmm. So overall, would you say this efforts that the institution do for you are mostly ineffective?**
- P14: I would say so personally, yeah. But have to give credits where it's due; it’s better than nothing. And some other nurses may find it effective for them. Like maybe Zumba works? Yeah. Maybe for some nurses, the problem is the lack of activities or not knowing where to go, then this will definitely solve their regulation issue. Whereas some nurses or staff will be the lack of time or the lack of rest day between very long shifts. Hmm.
- **NJCL: Hmm, then, other than the Zumba classes, are there any workplace health-promotion programs in place for your institution?**
- P14: Actually, there have been a lot of health promotion programs in the institution like there is mental health well-being days where last year they give us an extra day off for mental health Day. Yeah. So like, you get one off day, but these also come with the manpower issue where say, a lot nurses actually didn't get to claim that off day, because the roster didn't quite allow that. So the ideation and the implementation. There is still a bit of a gap because of the logistic constraint in the manpower issue that we have. Yeah. then, now they are enrolling in a lot more Care Ambassadors. Where the the goal is for them to offload some of the work that nurses may have. Like attending to call bell, changing diaper. They can assist us with this. So they are pumping in more manpower to help relieve the workload off the nurses to help us, but they also come with unintended consequences like let’s say, clinical incidents that happen because these people are not medically trained. So, it’s very reactive I feel when they roll out a solution, so when they rolled out the Care Ambassadors, clinical incidents happen, then it becomes the nurses responsibility to supervise the Care Ambassadors. So yeah, there’s always a little bit of gap, and there hasn’t been a perfect solution. But yeah, efforts have been made lah.
- **NJCL: Hmm, Then, then I will just summarize a bit of what you've been sharing. So for you. When I asked about how the institution can help to self regulate you mentioned that they are trying, they are, but for you, like the main issue of like, the rostering is not really solved so overall, it's ineffective measures for you personally. Then you also mentioned that there are health promotion programs. But then, the implementation leaves a lot to be desired. Because a lot of people actually didn't wasn't able to, for example, during the mental health weekday, the one off day wasn't claimed because of the rostering issue. Then now there's this Care Ambassadors, healthcare ambassadors to actually help. But then this unintentionally caused more clinical incidents. And now, in the end, nurses have more things to do. So it’s a very reactive solution to a problem and no perfect solution exists for now. Is there anything else you want to add for this part?**
- P14: I think. Oh, I guess they have been rolling out a lot of educational materials on how to take care of yourself on mental well-being. Then also they rolled out support system that there's a hotline where you can call in. If you need mental wellbeing support, and also they provide very good staff support. Let's say you require psychiatry support or mental medical care. So these are the things that I think has been effective. But it doesn't do much for the prevention part. But yeah.
- **NJCL: Hmm. You mentioned, there's educational material on how to take care of yourself. Is this very effective? Considering as a nurse, you did mention that you already have the knowledge on how to take care of yourself?**
- P14: Actually, no, I don't think so. But it's like, it's nice to see. [Participant laughs here]. It boosts your morale a little bit, but honestly, I don’t think it has been very effective, it’s just another message to pass along.
- **NJCL: Okay. Is there anything else you want to add here?**
- P14: I don't think so. Yeah, that's it.
- **NJCL: Okay. Then do you have anything else that you would like to speak about that was not mentioned in the interview? Any further comments you want to share?**
- P14: I don't think so.
- **NJCL: Okay, then, this marks the end of the interview. Thank you for your time and participation.**
- P14: Thank you. Thank you for having me. All the best for your thesis!

-End of P14 Transcript-

# **Interview #15**

Participant and interview details
 Participant number: 15 (P15)
 Date of interview: 6 Oct 2023
 Interviewer Number: Navarro John Christopher Lambino (NJCL)
 Time IDI started: 1030
 Time IDI ended: 1125
 Observational notes: Participant was unable to turn on video recording.

Section I: Interviewer’s summary
 The individual interview was conducted via Zoom. Rescheduling the interview for this participant happened quite a number of times. In the end however, we settled on 1030. At the start of interview, participant was reluctant to turn on video recording and sensing this, I offered that it was alright not to turn it on. Throughout interview, her responses were quite straight to the point, and I realised I had not needed to ask her to elaborate. Perhaps because her responses were quite similar to all the previous interviews, I was not pushing as much to further expand on her point. And there was a strong emphasis on self-determination for one’s health and having the healthy lifestyle before working as a nurse continue on to full-time nurse. She was also a bit reluctant to answer about the role of nurse managers, as she did not seem like she wanted to paint them in a bad light. I reassured her that the interview will be kept confidential to assuage her concerns. Interesting point she raised was that, nurses should have known shift work was a part of the work and they should have expected it from the start. The lack of free time is also a common point of interest. Overall, interview felt faster than other interviews but P15 was still willing to share her views on the matter succinctly.

Section II: Transcript

- **NJCL: Okay, good morning, miss. Firstly, I would like to thank you for taking the time to speak with me today. My name is Chris, and I'm a NUS Year 4 nursing student. The aim of this interview is to get a better understanding of the perspectives nurses have on self regulating their health behaviors and how various factors may influence this. I want to find out what are your views, opinions, and experiences on this. I would really like to encourage you to speak what comes to mind. There are no right or wrong answers. May I have your permission to continue with this interview?**
- P15: Ah, ya.
- **NJCL: Okay, then let's start with some basic questions first, like, which ward are you working in?**
- P15: Currently, I'm in [Public hospital name] gyneoncology ward.
- **NJCL: Could you tell me like, what kind of work do you have to do?**
- P15: Because ours is a gynaecology ward, so sometimes we do receive basic gyne cases, post-op, pre-op but the main pool of patients are oncology patients, so patients who have underlying gyneonco conditions. So they come in for any symptoms or complications they have for operations. And sometimes we do run chemotherapy in the wards.
- **NJCL: Could you describe your workload like, what’s the amount of work that you have to do for each day?**
- P15: So we do cubicle nursing. So usually for morning and afternoon shift, so for the in charge 1 to 6 patients to a cubicle. Then usually most of the time, the cubicles are full. So workload wise, it really depends on how complicated the cases are each day. But usually, every day is quite busy, like we don't get to sit during our shift, we all would be constantly on our feet moving around. Yeah.
- **NJCL: Thank you for that. So for my next series of questions it will be regarding your lifestyle in general. So as a nurse, how would you define a healthy lifestyle?**
- P15: The ideal lifestyle for a nurse, or like…?
- **NJCL: The ideal lifestyle for you.**
- P15: Say ideally, ending work on time, which never happens. [Participant laughs here]. Because for our ward- I think it’s because of the ward culture, we don’t really go for our full break. A full break is technically 45 minutes to 1 hour in the contract, but we don’t do that. So we just go for a short while, buy our food, finish eating and we will come back out to complete whatever work we have, just to help out other colleagues in case they need help also. So we have to eat very fast everytime, so that’s not very ideal. Then, um, sometimes, because we sometimes work night shift, we work 5 days a week with 2 days off, right? But most of the time, one out of those 2, one of those day off, is post night off, which isn’t really a full off day? Because you end in the morning, then you go home and sleep, then after you wake up, it’s probably evening time, you only have that less than half a day of rest or free time, then the next day you have to go back to work again. So maybe post night off shouldn’t be considered as an off-day in a week itself. Then other than that, yeah.
- **NJCL: Then, specifically on diet wise, how do you feel about your current dietary habits?**
- P15: So at work usually we just buy from the Kopitiam that’s in the hospital, right. Option wise, it’s your typical Kopitiam; Japanese, Yong Tau Foo, chicken rice, all this. 90% of the time, I just go for Yong Tau Foo, because we need to eat fast, right? So, personally I just need something to fill me up, I don’t need nice food. I’ll just eat better food after work. But during work itself, it’s just, something to fill myself up. Then outside of work, I do try to opt for healthier options but sometimes after work it’s so stressful, so I end up snacking quite a bit.
- **NJCL: Do you feel that you would like to change your diet to a healthier one?**
- P15: I wouldn't say that my current eating habits is very unhealthy also. So maybe I wouldn't change too much of my current diet. Yeah.
- **NJCL: Moving away from diet. The next question would be regarding like physical activity. So how how do you feel about the amount of physical activity you are having** **in your current lifestyle right now?**
- P15: Because I do work out. I have a gym membership. So I do work out around 3 to 4 times a week. Yeah. So it's still okay, like, I try to fit it into my schedule. So sometimes, maybe before the start of afternoon shift, I'll go, or sometimes after morning shift, I'll try to go. So yeah. So I still have time to work out despite the working schedule. Yeah.
- **NJCL: What would you say keeps you motivated to actually go for gym sessions after like a timing day of shift work?**
- P15: I guess, because I started gyming before I even started working as a nurse. So that itself was a habit or lifestyle that I had prior to nursing. So after joining nursing, I just wanted to continue that lifestyle and I guess, sometimes after a long day a work, it’s kind of an activity to unwind, a release outlet, I feel, for me.
- **NJCL: Okay, yeah. Just to clarify a bit. So it's like, because you already had this like habit before being a nurse: that you continue this lifestyle until you actually started working?**
- P15: Hmm!
- **NJCL: So you you mentioned something about your lifestyle before and after being a nurse. Could you like elaborate more on how did your lifestyle change after you started to work as a nurse? If there's any changes?**
- P15: I think, prior to starting work as a nurse, there was more free time as a student. So, I had more time to socialize, spend time with my family and socialise. And then I feel more energized on days. But after starting working, I do try to socialize as well after work or my off days, but I guess the energy level is really different compared to prior to working. I still go out but I don’t feel myself as energetic as I used to be because maybe lesser sleep throughout the days, and just mentally tired sometimes as well. That’s like comparing student days to working adult days, I guess. That’s the major difference that I felt.
- **NJCL: Other than being mentally, physically more tired. Is there any more changes you could think of?**
- P15: I guess the shift in your priorities now. Like previously- plus your free time now is much more limited. So you will prioritize things that are really really important to you and you will spend time with people who are more important to you on your days when you are off. Yeah. Other than that, I try as much to still maintain a balanced life.
- **NJCL: Could you elaborate on that? What do you mean by a balanced life?**
- P15: Still doing what I do prior to working at the start of work. For example, like I mentioned, I used to work out last time already, so I still tried to maintain that even after working. I socialize, I still continue socializing and not like, completely like, cut down on socializing. After work. Yeah.
- **NJCL: Earlier, you also mentioned there’s a shift in your priority. Could you elaborate more on that?**
- P15: I guess last time,I had more time, so I would spend more time with friends instead. But now, because of the limited time I have. I would shift my priority to spending time with my family instead. Yeah, and then I'll fix days to spend with my families. Then, if I have extra time from there, then I'll go meet my friend. Okay, yeah. Something like that. Yeah.
- **NJCL: Okay, so, earlier you shared with me like your dietary habits, the amount of physical activity you're doing, which is mostly like your physical health. How about more specifically on your mental, emotional well-being, how do you feel about your current lifestyle?**
- P15: Hmm, okay, at the start, because I started working for only one year plus, then at the start it was a very mentally challenging because it was a very specialized ward. And I didn't know that I was being put into a oncology ward until I started my first day of work. And prior to that, I didn't have much exposure to oncology because in school they didn’t- sorry is the construction, can you hear the construction?
- **NJCL: No, I can’t hear.**
- P15: Okay, yeah.. In school we also didn't learn much about oncology. They didn't in-depth go through it. So it's a whole new thing. So the learning curve was quite steep, and then it was an environment where everything is very fast paced. So. I have to learn on the job, right. Pick things up and stuff so initially it was very stressful. But thankfully the, all my colleagues in the ward are very nice, so I feel that like having a very strong bond with your colleagues, having that environment and culture of helping each other out really makes the whole working experience different. Yeah. Because I do hear, my friends, from other wards complaining about how their ward lacks team spirit, in a sense that everyone just care about their own job, you know, I take care of my cubicle, you take care of a cubicle. They won’t offer to help, even though, like, yeah, they are free-er compared to you when you're busy. Yeah. But for my ward everyone help each other out. Hmm, so that really helps a lot. And I guess, like sometimes it's really very tiring, and like you feel like giving up, but at the back of my mind. I just keep reminding myself that I have my colleagues who will always be there to help me, and then like if they can, if they can work in this particular ward for so long, I can do it, too.
- **NJCL: Yeah. I guess, just to summarize a bit of what you've been sharing so far. So when I ask you on the question on like how would you define a healthy lifestyle, or like what is an ideal lifestyle for you. You mentioned that you would hope you end work on time, but that doesn't really happen according to you. Then you hope to also go for full breaks, so you can actually like finish eating in and enjoy your break. Then you mentioned how that one of the of off days out of the 2 is actually post-night off. So that really cuts down the amount of free time you have. Then, for dietary habits, you mentioned that outside of work, you would actually try to go for healthy options. But generally you, you find your your diet isn't unhealthy, and you wouldn't really change much. Then for physical activity, you actually still go to the gym, 3 to 4 times a week. So I mean, I'm guessing in terms of physical activity, there's nothing, much you want to change?**
- P15: Yeah.
- **NJCL: so regarding like emotional and mental well being, you were saying that initially, it's actually very challenging because of the steep learning curve. So because of your work environment, which includes your colleagues, there's a sense of team spirit. So you would say- is it right for me to say, this sort of, improves your, your emotional and mental well-being, being in a environment filled with strong social support?**
- P15: Yeah.
- **NJCL: So right now. compared to like the initial few months. your emotional and mental well-being is improved, or…?**
- P15: Okay, because initially, it was the stress from having to learn things right, but now, sometimes it gets stressful but now because of the workload itself. But I think I learned to better manage my emotions and don't let it affect and just think that every shift will end eventually. Yeah. And I guess the interaction with my patients also keep me going. Because it's an oncology ward. So most of the time, patients come in and go out very frequently. So it's always the same familiar faces. And you build rapport with your patient. And you know that you’re doing it for your patients lah. So that's what keeps me going now as well.
- **NJCL: So when you said you're doing it for your patient. it's like giving you a sense of purpose. Am I right to say this?**
- P15: Yeah, a sense of purpose to continue, even though sometimes it might be tiring.
- **NJCL: Okay. Then, moving on to one of my next questions, as a nurse, how do you feel about self-regulating a healthy lifestyle?**
- P15: Self-regulating… I guess like it's true that we do shift work. And it's harder for you to kind of like fix your schedule to make healthier lifestyle options like working out, like sometimes like there are classes, and then certain like workout classes, they are fixed on certain days right, every week. But as nurses, we can't do that. Because our cause of our shift, right? Because you might not be working today, but next week on Friday you're working. But there are other options, so we can still work around it. If you yourself are determined to lead a healthier lifestyle, you'll still be able to. And for eating habit wise, I think it's also the same like. If you really want to have a healthier diet, you can. You can cook your own food and drink if you have the time. Or just shop for healthier options in the Kopitiam. Because, I think most of Kopitiams in hospitals, they have healthier options as well. So it's very whether or not you want to. So the being a nurse doesn't really restrict me from making healthier life choices if I want to.Yeah.
- **NJCL: And so for you, you would say that making healthier life choices is more- it really depends on would you say intrinsic factors, like your own self- determination to make changes?**
- P15: Yeah.
- **NJCL: So for you, you won’t be affected by extrinsic factors?**
- P15: Okay, there will be extrinsic factors that will affect you somehow. But at the end of the day, because lifestyle is a long term thing, right? But these extrinsic factors only, like happens, probably on the day itself. You can let it affect you for a day, a few days, or maybe after a week, but after that you can still go back to your normal self and be motivated by the intrinsic factors. Because intrinsic factors are like longer term, compared to extrinsic factors, right? So the external factors will affect you for a while. But after that you can choose to let go of it, forget about it, or like then continue being motivated to meet a healthy lifestyle for the long run. So maybe for the few days you will not be motivated, but slowly, you can get back on track again. Yeah.
- **NJCL: Okay. Can. Then, moving on to my next question, you actually touch upon it with your answers here and there. But what are your main facilitators or motivators in self regulating a healthier lifestyle?**
- P15: I guess, yeah. I think I touched on it. Because prior to working, I already led an active lifestyle. So I feel like starting work shouldn't stop me from continuing that. Yeah, beause it's already part of my lifestyle prior to working. And then working is just another phase of life, right? So we study. Then we work. So yeah, it's the mundane part of life. So I should continue with whatever I want to do outside of work, outside of studying. Yeah. And specifically, because nursing, the job of being a nurse, we are there to serve, to help our patient, right? So that sense of fulfilment that I get from the job keeps me going as well. Yeah.
- **NJCL: So your main facilitator or motivators is prior to work, you already have a healthy lifestyle and you're just continuing it. Then, also, having a sense of fulfilment as a nurse actually keeps you going, keeps you motivated.**
- P15: Yeah.
- **NJCL: Is there any more you would like to add for main facilitators or motivators?**
- P15: No, I think these 2 are like the main.
- **NJCL: Okay. Then, going away from facilitators and motivators. What are your main barriers in self regulating a healthier lifestyle?**
- P15: Main barrier? Mmm. I think. I would say the main barriers will be like days where your workload is more than usual. Yeah, but it's not like a constant barrier. It's just sometimes these days happen, these days you’re more busy than usual for your shift, your patients have more complications compared to other days. Yeah. So on days like this, I’ll feel physically and mentally drained by then. I guess it's just a temporary thing, like a temporary barrier for that day or for a few days. But afterwards it doesn't affect me anymore. Yeah, so there isn't really a constant barrier that is present to defer me from maintaining or regulating my lifestyle. Yeah.
- **NJCL: Then, specifically on shift work. How do you view shift work in relation to your healthy lifestyle? I think, earlier you already mentioned that it will make it harder for you to fit your schedule. Maybe. Could you elaborate on that?**
- P15: Yeah, okay, it. Okay. Doing shiftwork makes it harder for you to plan ahead of time, right? To schedule outings or whatever with your friends, right? But we step into nursing, knowing that it's going to be shift-work bound job right? So it's a factor that we should have already considered before we have decided to join nursing. Yeah. So it's more about working around it to find your time to do whatever you want. Hmm. yeah. I mean, because if one, if a nurse is bothered by the shift work thing, and like, if that's deterring them from socializing or regulating their lifestyle, then maybe they can consider working in a clinic or something where there is fixed hours.
- **NJCL: Hmm. Okay. So is it right for me to say shift work is not much of a barrier for you?**
- P15: I mean, its kind of is a barrier to in terms of flexibility like it doesn't have, it doesn't have as much flexibility compared to fixed shift right? But then, it's just something that we need to work around it, because it's just the nature of the job. Yeah. Hmm.
- **NJCL: So I'll just summarize what you've been sharing so far. So on my question, on like, what are your main facilitators or barriers in self-regulating your healthier lifestyle. For main facilitators, you mentioned that it's already your existing lifestyle and it's also the sense of fulfilment you get from doing your job. Then, for main barriers, you mentioned that it's not really a constant barrier. But there are days when the workload is too much, and you're too physically and mentally exhausted. Is there anything else you would like to add for any of this?**
- P15: Hmm. No, that's that's above it.
- **NJCL: Hmm, then, my next question would be, how does your self regulation habits impact your work performance?**
- P15: Work performance? Okay, I'll say, that because of my lifestyle habits… at work because we’re constantly on our feet and moving around, having to like lift patient, shift patient. So I guess that helped a bit because I do- I’m okay with carrying certain amount of weights so like that isn't as physically challenging for me in the physical aspect, and then having to be constantly on our feet. So in a way, in terms of the physical aspects, it's okay. So it helps me. It isn’t a challenge for me in terms of the physical aspect of caring for our patients. Then I feel like that my lifestyle doesn't really affect my work performance. There isn't really a link. Yeah.
- **NJCL: Hmm. And if not, work performance, how about like your life in general? Does your self-regulation habits impact your life in general?**
- P15: I guess it makes me more, I have stronger perseverance, I guess, because I set a structure, a routine, sort of like a routine for myself. So at work also, it's also a routine, right? So I'm used to being in this routine structure in like- I guess it helps me to press on more, be more determined, and not give up so easily at work, and not like let my emotions control me doing work. Don't let my emotions affect myself at work. Yeah. Yeah. So I just keep telling myself that at the end of the day, no matter how hard the day is, the shift will end. So I just press on like that because usually, all along I had that mindset already, so I bring it into work as well. Yeah.
- **NJCL: Okay. Thank you for that. So my next few set of questions will be regarding the workplace.So as a nurse, how do you think the workplace influences your own self regulation habits?**
- P15: Self-regulation habits? I will say that the people you work with, your colleagues are people that influence your choices, your habits. So I do know of- I do hear of some people in their ward they do order Grab food, or order food outside from the hospital, and so usually like one person, will ask like: ‘oh, do you wanna order?’ Then the other together? So I guess that would affect your lifestyle choices, your eating habits. So sometimes you didn't think of ordering like, lets say, fast food, but then your colleagues suggested to do that. Then you'll be like, Oh, okay. I'll order too. Then end up eating whatever they are getting. Yeah. So I guess colleagues in the workplace kind of affects your diet choices or something like that. But there are also colleagues where they will ask you, ‘oh, do you want to go for this class together?’, 'Do you want to go for Yoga class together after work?’ That kind of thing. Yeah. So you'll be motivated to. Maybe you didn't think of going for a workout class, but because your colleague asked and you can see it as a bonding activity with your colleague so you’ll be okay and open to go and try. Hmm. I guess colleagues really influence us in the choices we make. Yeah. And like I guess the nature of the work is physically demanding right? So I do have some colleagues who like pick up exercising because they want to be able to perform physically better at work, because maybe they're not used to the bending, the constant walking all this. So they want to change their lifestyle a bit to adopt healthier lifestyle. So they start, start picking up working out something like that. Hmm.
- **NJCL: I see. Hmm. Okay, I think you touch upon it a bit. But how do you feel about the workplace culture among nurses in helping to facilitate self-regulation of healthy lifestyles?**
- P15: The culture?
- **NJCL: Like the social aspect of the workplace.**
- P15: I think the so far, we never really hear about them promoting regulation of a healthy lifestyle specifically. But then they do, they do try to arrange like brisk walk activities, volunteering activities. Some, some sort of activities now and then to encourage nurses to join, to lead a healthier lifestyle. But then I wouldn't say that they’re actively doing so. Yeah, it's just an on and off thing. But then not a lot of people are keen in joining or so, because it's probably just a one time thing. So I'll say, like, I don't think they are actively promoting for nurses to need a healthier lifestyle.
- **NJCL: So just to clarify, your workplace, there's a culture of like promoting on and off like one time activities to try to encourage?**
- P15: Yeah, to just try.
- **NJCL: Do you think the workplace culture among nurses, instead of encouraging, does it like hinder or discourage healthier lifestyles?**
- P15: I wouldn't say, there’s much that will hinder or discourage a healthier lifestyle in terms of the culture. Yeah. But like, I guess, rather than culture, it's just- sometimes it’s really just physically tiring for some, so they just want to rest after work. So that deters the from leading a healthier lifestyle, rather than the culture itself.
- **NJCL: Hmm, so it’s more of the workplace environment?**
- P15: Yeah, environment rather than culture.
- **NJCL: Hmm. then, what do you think about the role of nurse managers in helping nurses self regulate their own health?**
- P15: Hmm. Because nurse managers, they they manage at ward level, right? So each ward will have their own nurse manager. So I'm assuming that you’re referring to each ward’s nurse manager?
- **NJCL: Yeah.**
- P15: Yeah. Okay, because they're overseeing that pool of nurses, only right? So maybe they can, you know, take the initiative to encourage and organize some activities like rather than the organization organizing like one time brisk walk kind of thing. Maybe the nurse manager can be the one to initiate like healthier, like a team bonding physical walk or something kind of thing, like once a month at least. So maybe they can be the one initiating because for them, they will target only a small pool of nurses that is under their management. So it’ll be easier for them to initiate. Yeah, then, if each ward’s manager do that, then each ward’s nurses will be exposed to it more rather than the organisation doing it. But then it doesn't- not everyone will be keen to join. Yeah. So I guess, like the nurse manager may have the ability to motivate if they take the initiative too? Yeah. But then I'm not too sure, how busy are they also lah, whether they have the time to organize such events or to motivate. Yeah.
- **NJCL: Hmm. So for you, you believe the nurse managers have a role in helping the nurses on the ground to actually self regulate their own health?**
- P15: I wouldn't say they have a role, but if they want, do I believe they have the ability to motivate? Yeah. But then? they themselves have to be motivated first. Yeah. They can, if they want to. Yeah.
- **NJCL: Then, specifically, in your ward, would you say your nurse managers actively take part in this, in motivating the staff?**
- P15: No, they they don't. [Participant laughs here].
- P15: Yeah.
- **NJCL: Okay, don't worry, it’ll all be kept confidential. Sorry. Could you elaborate on- could you maybe, like guess or like estimate, why, they don’t take a part in actually encouraging the nurses on the ground regarding healthier lifestyles?**
- P15: I guess because they themselves don’t really try to regulate or lead a healthier lifestyle themselves and probably because of the workload that they have also. Yeah. And that's why they don't. And I guess it’s a habit? Usually nurse managers are older, right? So I guess, since young, since they started working as a staff nurse. They already don't have the habit of leading it, so eventually it just continues through their life. And now they have reached the stage of being a nurse manager. So it just continues. Yeah. So personally, they don't lead a- they don't try to- okay, maybe they do. But then, like- yes, that's why they don't motivate because in the first place, they don’t do it themselves.
- **NJCL: So going on with this like train of thought. So like you're saying that because the nurse managers themselves don't have this habit, they they cannot really encourage their own nurses on the ground. Then is this also true when it comes to nurses and patients? Do nurses who are unable to lead a healthier lifestyle able to encourage patients to lead a healthier lifestyle?**
- P15: I guess, like, it's part of the job for you to encourage your patient to lead a healthier lifestyle as a nurse, right? So, even if you don't do it yourself, you can still verbally encourage your patient to do so. Yeah. And then I guess your interaction with your patient isn't like- it's just throughout their stay, right? And usually you encourage them, probably during their discharge. So you just like, give them discharge advice. They're like, Oh, you should avoid this. Avoid that. You should try doing this more. Try eating healthier, less salt, less sugar, that kind, but these are all verbal advices, healthier advices that you give to your patients. So you don't need to be- to do it yourself for you to verbally advise them to do so. So in terms of that, the verbal aspect, you you still can do. You still can execute it. Yeah, it's like you have- you have the knowledge of it, of how a healthier lifestyle should be, even though you don't execute it. So you still can pass on that knowledge to your patient. So it's also up to your patients whether or not they want to execute it as well. Yeah. So I would say, like even though a nurse doesn’t lead a healthy lifestyle herself, she will still be able to educate her patient on it. Yeah, as long as she has the knowledge of what the healthier lifestyle is like.Yeah.
- **NJCL:Okay. Moving on, to my next question. Then for you, how do you think your institution can help you to self-regulate a healthier lifestyle?**
- P15: I think personally for me it's more of the rest time that needs to be more regulated. So yeah, the eating, the break time thing. I guess. I'm not too sure why, but then usually for afternoon shifts for people in my ward, We go for shift right after we take report- so our break, even though afternoon shift is from 12:30pm to 8.30pm, our breaktime is usually around 2 to 3. So it’s at the very start of your shift, but I think for other wards, they do go for their break for afternoon shift in the evening, around 5, 6, in the middle of the shift. Which I suppose breaks are supposed to be scheduled that way. So I guess, like the organization could like fix that every ward, every shift should have this fixed timing to go for breaks and maybe I guess, can regulate it. And go for like break- to emphasize the need to go for your full break. Then maybe do something about the post night- off. But I guess the post-night off thing is like they do count the hours that we are working, and then it adds up. Like the hours we work in a week, adds up, and it's and it's within the hours, so they can't afford to give us more off days outside from post night-off. So I guess, like there isn't really a solution to that. But yeah. Ideally, I do hope that post-night off shouldn't be considered as a full day off. Yeah.
- **NJCL: Earlier you mentioned, there are like activities that institution organize for nurses. Do you think those are effective, ineffective?**
- P15: Personally, I have never joined by any of the activities they have organized so far. So I'm not too sure exactly how many people have actually sign up or went for the event that they have organized. So I would say, it's not very effective. Yeah.
- **NJCL: Hmm, what what do you think will make you want to join these kind of activities?**
- P15: Hmm. The thing about this kind of activity is that because we do shift work, right? So in the first place, we don't even know whether we'll be working during that day where they planned the the event or activity. Hmm. I would say, like- because, usually how they promote such activities through email. So they would just mass send emails out to everyone. But then, what if I don't check my email? Then I wouldn't know that there's such an activity that's gonna happen right? So I guess the the promoting of the event, the way that they promote it can be changed like instead of emailing. But not everyone will check their email right? They can like, print it out and advertise it, probably in the Kopitiam. Stick it in the Kopitiam, or something. So that, like more people can see. Or at the lift lobby kind of thing rather than sending out via email. Yeah. Rather than just like having that one day for the event, right? Like, let's say, they just fix that one day for the brisk walk event. Probably they can schedule like different days instead of just one day, so that like, if I can't make it for that one day I still got the option of another day where I can join. Yeah, something like that, yeah, increases the possibility and availability of the nurses joining the activity because of their shift work.
- **NJCL: Just to clarify. So they, when they promote the event, they promote one week before the event, a few days before the event?**
- P15: I think they sent out the email quite early. But depends on how soon you see the email yeah, nothing, like that, yeah.
- **NJCL: Hmm. Are there any workplace health promotion programs in place in your institution?**
- P15: For nurses?
- **NJCL: Yeah.**
- P15: I don't. Yeah. yeah, I don't think there's any. Not any that I know of. Yeah.
- **NJCL: Okay. just to summarize what you've been sharing me so far. When I asked about how do you think your institution can help you. You gave mostly like things the organization can do, such as like your break time thing. Because you say that your break time is around to 2 to 3 which is at the very start. So you you would hope the organization can actually fix the timings, and like emphasize the need to go for full break, and also mentioned that you hope that they do not count the post-night off as off day. Because you don't have much free time after resting. And you also said, like, personally, you never went for much of the activities the organization actually organized. So it's not very effective because it's like, how they promote it through email is not very desirable. You would hope they maybe make a post and put it in Kopitiam. Then you also hope that they schedule it for more than one day, so that it increases the chances of like more nurses going for the event. Then, lastly, regarding health promotion programs, you said that there's not much that you know of. Is there anything else you want to add here?**
- P15: Ah, no. That’s it. Yeah.
- **NJCL: Okay. Then. do you have anything else that you would like to speak about that was not mentioned in the interview? Any further comments you want to share?**
- P15: No, that's all.
- **NJCL: Okay, then this marks the end of the interview. Thank you for your time and participation.**
- P15: Thank you.
- -End of P15 Transcript-

# **Interview #16**

Participant and interview details
 Participant number: 16 (P16)
 Date of interview: 7 Oct 2023
 Interviewer Number: Navarro John Christopher Lambino (NJCL)
 Time IDI started: 1705
 Time IDI ended: 1815
 Observational notes: Participant looks alert.

Section I: Interviewer’s summary
 The individual interview was conducted via Zoom. Due to participant’s technical difficulties, interview only started 5 minutes after the agreed timing of 1700. P16 is a relatively fresh nurse who has worked in ED department. Additional context; this interview was conducted post-night shift. From her answers, she is actively engaging in physical activity and self-care outside of work. Many similarities with other participants. An interesting point of view she mentioned was healthy lifestyle is holistic, and due to different circumstances, some people may be prioritising a certain aspect more than others. For example, emotional aspect of self-care instead of physical so does not go and exercise as often. In addition, she also mentioned that nurses should empathise and understand the circumstances when giving advice; do as I say, not as I do mindset. This also applies to nurse managers, who she believes have an active role in helping nurses maintain their health as they have control of the rostering and also role modelling- doing actions to show they are there to support the ground staff. Perhaps due to being distracted with my own assignment deadlines, I found it quite difficult to follow the train of thought of interviewee. The links between her answers to the questions has to be inferred through context, and some responses almost do not feel like they answer the question. Overall though, there is sufficient relevant data from this interview that can be used for data analysis.

Section II: Transcript

- **NJCL: Okay. good evening, miss.** **Firstly, I would like to thank you for taking the time to speak with me today. My name is Chris, and I'm a NUS Year 4 nursing student. The aim of this interview is to get a better understanding of the perspectives nurses have on self regulating their health behaviors and how various factors may influence this. I want to find out what are your views, opinions, and experiences on this. I would really like to encourage you to speak what comes to mind. There are no right or wrong answers. May I have your permission to continue with this interview?**
- P16: Yep.
- **NJCL: Okay. Then, let's start with some basic questions like, which ward are you working in right now?**
- P16: Sorry?
- **NJCL: Which ward are you working in right now?**
- P16: Oh, I'm in the emergency department.
- **NJCL: Could you elaborate on like, what kind of work you have to do daily?**
- P16: So in emergency, we do a lot of onsite care. So it's a lot of acute care like giving prompt meds. And then we also do a bit of ward work for all the patients who don't have beds. So we'll just operate like a normal ward for the lodger areas. And we also do a lot of like point of care of kind of care, like, stabilizing fractures, all that kind of stuff that basically.
- **NJCL: Hmm, how would you describe your workload?**
- P16: Uh. Chaotic. [Participant laughs here]. Very heavy?
- **NJCL: As in in terms of like, maybe how many nurse to patient ratio, like the amount of work you have to do?**
- P16: Oh. For nurse to patient ratio, I’ll probably go by our lodger areas, because in our acute areas it can be like, 2 to 30, sometimes more, but it’s dependent on the area, and the level of care lah, because they are triaged in. So for the more stable patients, we can have like, 2 nurses to like 40 because they can all walk and talk and everything. But, in lodger areas, where they are serious enough to require admission which is probably the normal kind of ratio, I think we would have max, 2 to 12 or 13? Yeah.
- **NJCL: Hmm. so would you say this is quite manageable for you?**
- P16: Not exactly. But I mean, you cannot do anything about it. [Participant laughs here].

**NJCL: Okay. My next series of questions will be regarding health lifestyle in general. So as a nurse, how would you define a healthy lifestyle?**

- P16: Hmm, I would say for that you have to go on a more holistic basis. So you have to look at like sleep, nutrition, exercise and then also, like the mental health aspect like, if you're feeling all right, if you're feeling burnt out, how do de-stress, stuff like that? So if you manage to take care of yourself in a relatively all rounded way, I would venture to say that it's pretty healthy. Yeah.
- **NJCL: Could you elaborate more on what you mean by take care of yourself in a rounded way?**
- P16: So like, you know all the factors that I listed just now, they don't exist in solitary, so you have to make sure that you eat well, that you sleep a good amount of hours a day like ideally, maybe 7 or more, that you try and exercise at least do the 2 to 3 times a week, and stuff like that. But you also cannot look at all of these in isolation. You cannot be like oh, I slept well, but I didn't manage to like eat or stuff like that. You also don't have to beat yourself up about like not meeting any particular criteria for like certain days, because, like some days, are just worse than others. And then you just try and do what you can, where you can if that makes sense. Yeah?
- **NJCL: And then specifically on your diet. How do you feel about your current dietary habits?**
- P16: I think I'm definitely snacking more than I used to. And then I try and get more like protein options, or like not so fried stuff. But especially with night shifts, usually the only thing that is open is Cheers. So you just get a lot of instant food. So where I can, I try and supplement it with eating more vegetables where I can and getting more protein. But yeah, I'm definitely eating more instant food and more fried food than like I did before I started working. Yeah.
- **NJCL: Hmm, hmm, You mentioned that your dietary habits changed when you start the working so like, how exactly did your lifestyle change after you started working as a nurse?**
- P16: Well. I think the thing that affected it- hmm, part of what affected it would definitely be logistics, because with shift work now you have to plan a lot of things. Like for my night shifts. I tend to skip lunch because I'm gonna like sleep in and then, like, eat during my night shift. And then It gets a bit hard to schedule things like exercise as well, because a lot of them are. They have like weekly classes, or whatever so like you automatically can't really go for those. Or you have to like request a lot of shifts from your nurse clinician and stuff like that. But I think it's also that, once you start nursing care, and you get into a point where you're interacting with patients every day. And there's a lot of direct patient interaction and bedside nursing; because of the high amount of social interactions that you do and like, because it's a high stress situation usually, particularly for the patients, as they are like all sick and like uncomfortable and stuff, it tends to be quite tiring on the social and emotional scale, because you have to manage a lot of people, and you have to manage a lot of their emotions and also try and find a way to make what they want happen if possible. So that one also tends to leave me very drained after each shift. Because, like, yeah, you're trying to juggle so many things at once. So then, when you come back, you're a bit too tired to really do anything else but rest. Yeah.
- **NJCL: You mentioned something on the social and emotional scale. So is it right for me to say that, once you started working your way of managing your own thinking, your mindset, your emotions have changed compared to when you just started working?**
- P16: Hmm, what do you mean?
- **NJCL: For example, maybe because of your experience, you’re more able regulate your emotions?**
- P16: As in has how I regulate, changed?
- **NJCL: Yeah, yeah.**
- P16: Oh. Okay. I think, not so much. Because I'm essentially doing the same things I used to. It's just that I probably have to do them more often. I suppose. Yeah, like, it's just- it's the same. But it bears a heavier weight now, lah. Because there's more placed on you during the shift.
- **NJCL: Hmm. Okay, going back a bit earlier regarding the dietary habits question, you mentioned that you actually, try to supplement your diet with more protein options and like not so fried stuff. Is there any anything that motivates you to change your diet?**
- P16: Well, I don't want to eat too much instant food and too much unhealthy food, because I know it's bad for my health. And, I suppose it's also that I like it as well because like if you just eat instant noodles, it's a bit sad, so sometimes I try and bring like an omelette. Or like a bit of vegetable. Then I can eat it with the noodles, or whatever yeah. At least more interesting. [Participant laughs here].
- **NJCL: I see. Then moving away from dietary habits. Now, specifically on like physical activity. How do you feel about your current lifestyle?**
- P16: I think it's alright, because recently, I've also been making like more of a conscious effort to attempt to exercise more. So like now I am doing like once to 2 times a week. But previously it was quite hard, especially with, like the logistic issue, because of like night shift and shift work and everything. But, I've recently been able to tap into like more community support like, get more friends to go with me. So even if the timing doesn't quite match up like you have quite a few friends, so you can just find one that does. and that helps with motivation. It helps with motivation a lot more. And then, yeah.
- **NJCL: So, for physical exercise, just to clarify a bit, you're currently making a more conscious effort to actually exercise more?**
- P16: Yeah, I generally do about one to 2 times a week, where I think maybe half a year ago it would have been once every 2 weeks or so. Yeah.
- **NJCL: Hmm. And what do you think is the main impetus that made you consciously do this?**
- P16: I think it's mostly the social aspect like I joined a few-I got to know a few people who are also interested in the sport that I do, and are also more advanced in it, and they- they want to exercise with me. But I can see that there is a skill gap in that sense. So I am actively trying to bridge that in order for, like our exercise sessions, to go out more smoothly as well, and and also as to enjoy myself more. So definitely, the feeling of wanting to catch up with them. And want to join them has like made me, try and work out more to practice.
- **NJCL: Okay. Moving on to my next question, I think, previously, you also mentioned a bit on it, but how about on your emotional and mental aspect of your lifestyle. How do you feel about it? Do you feel like changing anything?**
- P16: I think for now I'm not in any sort of crisis in the sense where work is tiring, but like I don't feel like burnt out, or I don't feel like, especially like, exhausted, or anything like that. I think that was something that happened like when I first started work, because I found it very hard to acclimatise to the fast-paced environment. But now I've actually gotten more used to the routine. So it doesn't feel as overwhelming so as of now, I would feel that emotionally, I am doing okay, like, I'm not- how do I say, in crisis? Liike I'm not, I'm not like, you know, like feeling really depressed or anything. But like, I'm also feeling on like average or slightly below average. So I do feel like it could be better. But I'm not drowning if that makes.
- **NJCL: Okay. So is it fine for me to say, because you're not in any sort of like, in any sort of crisis, you feel that your emotional regulation can remain constant?**
- P16: Yeah, relatively. Like, it could be better. But it's good enough that it's not the main problem right now. Yes.
- **NJCL: Moving on to my next question, it's, as a nurse, how do you feel about self regulating healthier lifestyle?**
- P16: I think it's very important. There’s quite a strong train of thought, especially amongst the general public that, like healthcare, only really happens when you step in like the clinics and the hospitals. But I think because you have worked there so many times. You know how much of these problems actually stem from daily habits. And that sickness isn’t- it can be acute, but a lot of times it's just the slow build up of your daily actions. So when you know something like that like, you know, it's hard to make yourself do the work like exercise and like diet and like try and go to sleep at a reasonable time, but like at the same time, I am faced with the consequences of not doing that like quite often. So I do know that it's important to make an effort where I can. I mean, there's definitely going to be genetic issues and stuff either way. But in general I can see how health lifestyle really helps health a lot, and it's very connected. So where I can, I think I am more motivated to do the work.
- **NJCL: Yeah. So just to clarify. Because you have the knowledge of like the consequences of not regulating, of maintaining your own lifestyle that you actually feel more motivated to regulate your lifestyle?**
- P16: Yeah, something like that, like, for example, I had a patient who I saw once, that had really really like dry skin to the point where it was cracking and flaking everywhere, even like changing diapers or changing their clothes, was like actively hurting them, because I could see a lot of like superficial dermis and epidermis actually being torn off even when we were being as careful as possible.I think that really stucked with me. And since then I've been moisturizing quite frequently. Like, yeah, you know that kind of thing. Yeah.
- **NJCL: I see, I see.Then, what are your thoughts on nurses who try to promote health but do not follow their own advice?**
- P16: Sorry! Can you say that again?
- **NJCL: You mentioned that, because you have the knowledge on all these consequences of not self regulating. Therefore you self regulate yourself. But then what about nurses who know the consequences, but they do not listen to the same advice they give their patients? How do you feel about these nurses?**
- P16: Oh, I mean. I feel like everyone has their own struggles. And like. even though- hmm! How do I say this? Because everyone is kind of like making choices according to what they feel is right. Let's say, if they are not exercising or choosing not to do certain things that could be beneficial to their health. It's also important to look at it from a holistic point of view because a lot of time like, let's say they're not exercising. It's because they're like tired. you know, like their emotional health is also taking a toll so like it looks like they're not trying to help themselves in a specific factor. But at the same time that could be because they are assigning a greater priority of care to another aspect of themselves that they feel needs more work. And I also feel like I'm relatively privileged in the sense that I'm still quite young. I don't have kids. I don't have like critically ill family members that are like requiring a lot of time, requiring a lot of care or even money. Because a lot of these things also come into play because we don't live in a silo and like a lot of times when they are trying to make ends meet, or like trying to like, make more money, or like really just have their needs stretched like, let's say, at home they have to take care of kids or something. Then, when they have time to themselves, they want to rest, you know. They want to like give more priority to like their emotional side more than like the physical side, or more than something else. So I feel like It's hard to judge someone else because like we don't know what they're going through. And in that sense also, then, when we do give advice to patients like, it's kind of like. Follow what I say, not what I do, you know. Like where I can, I know what will help a person's help, and that's why, I'm also advising the patient on such things. but like because of everybody's context, they might or might not be able to like do things. So I feel like as long as they extend the same kind of kindness and like understanding to the people that they are talking to. We can all kind of like share healthy advice, even if we're not necessarily doing.
- **NJCL: So, to clarify what you have been sharing regarding this. Saying that it's because of different- how to say? Because of different circumstances a person is going through, they might give more priority to other aspects of themselves?**
- P16: Yeah, something like that, because lifestyle is a holistic kind of thing. And some of these factors you don't see, so you cannot tell if they're working on it as well.
- **NJCL: Then, moving on to my next question: what are your main facilitators or motivators in self regulating a healthier lifestyle?**
- P16: Are you saying what motivates me, or like what I do, like facilitate…?
- **NJCL: Yeah. What motivates you to self-regulate?**
- P16: Oh, I don't know. I've just always felt that it's quite important to self-regulate. Because I I'm not really sure what motivates it, but I suppose it's the like. If you're not regulated, then your quality of life doesn't feel as good and mental health wise, you get a lot more volatile, so like that doesn't really help in the building of any kind of consistent habit, or like anything that you might find joy in like creating social bonds, making jokes with friends, getting closer to people, learning things. All these require a general need for you to be relatively self regulated, because, like, if you come in and you're like all over the place, like, even if people want like talk to you, or like, you know- even if you want to get things done, or even if you want to learn something, it gets very hard to do so, because then you'll be struggling more with yourself than with the external factors. Yeah.
- **NJCL: Okay. To summarize what you just shared, for your main facilitators, motivators for self-regulating. It sounds like, it’s mostly intrinsic? Inside of you? Because you did mention that you always believed that it’s quite important to self-regulate. It also helps maintain a certain kind of mindset for your mental health so that you're able to actually forms social bonds or for learning things that you actually need to. Is that correct?**
- P16: Yeah, like, if you're unregulated. It generally detracts from your quality of life, and like the experiences that you get to go for.
- **NJCL: Then, away from motivators and facilitators, what are your main barriers in self-regulating a healthier lifestyle?**
- P16: I think time. Yeah, because you have like, rest as in like sleep. And then you have like exercise. You have things you do for fun. And then you have, like social time. And like, if possible, you will also like to have like time spent outside, like going for walks like being in nature, soaking in some sun or whatever, and, like all of these, are nice things to do, and occasionally, where you can, you can combine these things like meeting a friend at the park, or like yeah, like. maybe going to a quiet place with someone or like journaling in a pub or something like that. But at the end of the day there's only so much that you can combine, and you'll still have to make it work with the rest of your life, because there is sleep. And there is exercise and there is all those factors I said just now, but there's also like personal responsibilities, like chores like cleaning the house, maintaining any pets or anything that you might have. So yeah, a lot of times. It's just not having enough time to fit everything together.
- **NJCL: Other than time, is there any other main barriers that you feel like that’s preventing you from self-regulating?**
- P16: [Long pause]. Not too much. I would suppose time is the main thing. A small thing would be logistics, but other than that…
- **NJCL: By logistics you mean…?**
- P16: Scheduling when you want to exercise or like, or like meet up with friends, or go out or anything. Because sometimes you make a plan and it starts to rain as well, so you can’t go out. Or like, I do like to enjoy swimming, but I have to make sure when to do it, because if like, let's say I assume right after shift, it works out timing wise in terms of efficiency. But I do find that I tend to get more nauseous because I haven't slept for a while. So you really wanna like find the perfect chunks of time here and there for certain activities. And they kind of depend on each other. So like the mental scheduling is a pain sometimes.
- **NJCL: Is it right for me to say like it also depends on fatigue. Fatigue also, plays a part in whether you will go for this other activities?**
- P16: Yes! Definitely. Sometimes you'll do something, and then like after shift, you are so tired. So you just really need to like get sleep done first. And then after that you can see what you can do. But hmm! Especially if like, let's say you have, like a frequent succession of night shifts, or like PM, AM, then PM. For that chunk of time you just focus on like sleeping and eating. Getting a bit of exercise where you can, but like the restful mental kind of rest, you just kind of leave for like the off-day, and then you see where you can like juggle that in as well, and see where you can find the space to like self-regulate, because those types of activities usually take a bigger chunk of time. So you just kind of do, if it makes sense, emergency care until you get to like the endpoint, then you rest up. Then you go.
- **NJCL: Hmm. Okay. Let me just summarize so far what you've been sharing with me regarding this question on facilitators or barriers for self regulating a healthier lifestyle for you. Your main motivator is mostly intrinsic. Because you intrinsically believe that it’s important to self-regulate and that maintaining a good mental health will result in a greater quality of life. Then for main barriers, it's time, the lack of time. Time to maintain yourself versus like having your own personal responsibilities. There's also like logistics, such as like the scheduling of some things. And lastly, fatigue. When you're just tired to actually do anything after a shift. Other than this for motivators and barriers, is there anything else you'd like to add?**
- P16: Not particularly, yeah. I think you summarize it pretty well. Yeah.
- **NJCL: Okay. Then, next question would be, how does your self-regulation habits impact your work performance?**
- P16: I think on the quality of performance aspect, it impacts it quite well because I've noted that when my when I'm more unregulated, I do tend to like space out more and like have a greater amount of time having to come up with what to do next. So I'm not quite as on the ball, I don't get things done as quickly. It's a bit hard sometimes to like interact with other people as easily as I could have when I'm more regulated. But also, on the other hand, as I said, about regulating taking a longer amount of time. That does mean that I don't get as much quantity of work put in, in the sense. Like, they're always asking for like ‘bank shifts’ or if we can work overtime. But like as I- as it is currently, I feel that that really detracts from it, because then you don't have as much self regulating time. So like when I was doing overtime or accepting ‘bank shifts’, I could note that even though I was physically there, and I was helping in the sense like, you know, the department is understaffed. They do need more hands, and like, kind of an all hands on deck situation. So even if you're not feeling the best. It's better than not having a person there at all. But I do notice that the work gets slower, so it's a bit of a give and take with how many hours you can do like, a quality quantity kind of thing. Yeah.
- **NJCL: For you, there's a sort of like conflict between self regulating yourself and actually doing a bit more work? Is that correct for me to say?**
- P16: Yeah, kind of.. Because, like, if you say how it affects what performance, I would say, it makes it better. But it makes me have to focus on quality versus quantity lah. So then- because a lot of people always talk about self care. But then, after that, on the second hand they turn around, and then they ask if we can work another shift if it can become another time on the block, and it's like. If I am to do said self-care. then I cannot possibly spend my time somewhere else as well. Yeah.
- **NJCL: Then, outside of work performance. Do you think self regulation affects your life in general?**
- P16: Yeah. Like I said, like you do get a bit more brain fog when you're not self-regulated, and then you tend to be more irritable with other people around you. There’s a great focus on sleep. You don't quite feel as motivated to do the things that you care about. So it's quite detrimental, I feel.
- **NJCL: Then, do you use any like tools or apps that help you like self-regulate your lifestyle?**
- P16: No, I tried a few, but I don't like paying for things. So, I've just mostly been using like free videos on Youtube or like- a lot of my stuff, generally I can do on my own like taking a walk or doing some journaling. So for the most part, I don't really use apps, I think, for the most will be like those kind of like free yoga, meditating videos. But yeah, that’s about it.
- **NJCL: You mentioned ‘journaling’, so does that mean you keep track of your amount of exercise, that kind of journaling?**
- P16: I do that, too. That one is like a planner kind of thing. But journaling is more like emotional processing.
- **NJCL: Hmm. So the tracking part, do you track calories with your diet and things like that as well? Or just-**
- P16: Nah.
- **NJCL: Okay, I see. For my next series of questions, It will be involving the workplace. So as a nurse, how do you think the workplace influences your own self regulation habits?**
- P16: [Long pause]. I suppose. At work, you have to use them more? [Participant laughs here]. It definitely stresses you out faster than being at home. But it's also like, I guess, the colleagues and the culture. Because if you're with a good team, then you don't really find it as much of a struggle, because they'll also be supporting you to take care of yourself, but they'll be encouraging you to go for breaks, or like, if they see that you're having difficulty managing a certain patient, then they'll probably help you, and vice versa. But if you have some colleagues that kind of like are a bit more passive or kind of like, leave you to do a lot of things on your own. Then it's definitely like, not only do you have to like self-regulate with the patient you have to self-regulate with the colleagues as well. So then, that one definitely fills your cup faster as well. Yeah. I suppose. Sorry, can I clarify your question, again? Sorry.
- **NJCL: As a nurse, how do you think the workplace influences your own self regulation habits? Yeah, I mean, your answer regarding the workplace culture is valid.**
- P16: Yeah. I'm not sure if I'm answering your question. [Participant laughs here].
- **NJCL: It does, it does. No worries.**
- P16: Okay. Other than that. It's also like, since at work, like I said with the PM, AM, PM, AM shifts, you don't really have a time for like long form, self regulation. So like you just do the kind of like deep breaths. Take a minute to yourself, try and like mindful eating or whatever, and then, like you try and just like plaster it until you get to like the off-day where you can really just take the time to relax and go through things slowly. So I think that would affect it as well. Yeah.
- **NJCL: My next question, you actually touch upon it by mentioning, like the social aspect of the workplace. So how do you feel the workplace culture among nurses helps facilitate self-regulation?**
- P16: Okay? I think if in that sense, it really depends on the workplace culture and the kind of behavior you promote from each other. Because a lot of social behaviors stem from the culture of the general workplace. So if everyone is kind of like: encouraging you to like self-regulate as in like they encourage you to go for breaks. They encourage you to clock in on time and go out on time. There's a general culture of helpfulness in the sense of like. I mean, there are some things that I cannot do, for, like my colleagues like, if they're already halfway preparing a medication, I can't just take it out from their hands. But, like, let's say, like vitals, diapers, like all of these are things that we all need to do. So. If you see your team as a unit. Then you can kind of focus on yourself. But also if there is that sudden degree of trust and implicit knowledge that everyone will help where they can. It's also a bit of a relieving factor, because you know that if you're in trouble, someone will try and help out or like- if anything happens and I happen to be free, I would want to go to like my other areas and see if they need, like an extra pair of hands or anything like that. And I think doing such things actually like, have to say, culture is something that is very prevalent, but at the same time very changeable, because it starts with every single person. So like, if you’re going out of your way to help when you can, and when you have the mental space, it also shifts a lot of the blame culture that we see. Because then everyone knows that if you have the time, and if you have the ability you would be helping. So if you don't help out, then they would naturally give you the benefit of the doubt in the sense where they will assume that you have your hands full as compared to sometimes with other cultures where the moment you like, don't step in or do like exactly what's expected of you. Then they will start pointing fingers, and they will start looking at who to blame. And this kind of thing is- it really is encouraged and strengthened by everyone in the unit. So it's something that depends on each person. So in the supporting of other people's self regulations, whether it's in taking breaks or letting them vent. Or just giving them a little bit of quiet space like if you can help out, give them a few minutes like that helps, too. Because I've been in some, like other attachments where, like the culture, wasn't quite as friendly. And then, in that sense, it really detracts against self regulating, because even if you have the time to take a break. Sometimes you don't. Because you're worried about how other people will perceive you and like whether that will get you a complain. Or like, let's say, if you technically don't have anything to do, but you need to watch the patient nonetheless in the case of like dementia, or suicide precaution like then it gets a bit frustrating because you could be taking a break if someone just takes a look at your patient, but like you can’t at the same time. So it's kind of like, even those 15 minutes are hampered because like at the back of your mind, you're still watching for something. You cannot remove yourself for a while.
- **NJCL: So just summarize a bit of what you have shared with me, you believe that the workplace culture- having a positive workplace culture, where everyone plays a part, to actually support each other, this can help facilitate self-regulation.Meanwhile, if there's like more of a negative kind of culture where people don't really help each other. This can actually also negatively affect the self regulation, because people can't do for breaks when they need to.**
- P16: Yeah. that’s about it And they just don't feel as well supported
- **NJCL: I see. Then, on the same vein of this, what what do you think about the role of like nurse managers in helping nurses self-regulate their health?**
- P16: Hmm! I think it's really important, in terms of like the culture as well because, like, it's something- because nurses nurse managers technically like, just by virtue of their role. They have more official power and also technically more soft power as well, because their emotions and their own speech means more. So, even in terms of like intonation and stuff. You have to know that they are the ones doing all the logistics and stuff. So if they are, if they tend to be like resistant, to, let's say like a staff taking MC- like technically, they can't say no. But you can hear it when they don't want you to. And yeah, in that sense, then it gets harder to advocate for yourself, because you know that they don't quite approve of it in that sense. like, because saying you support self care is one thing, but you need to like. put in the effort to actually make it so that people can find the opportunity to self-regulate and do things like that. And it's not even in, let's say, like taking MC or taking breaks. It's also in everyday speech, like it's very common for workers to take cues on their manager as to how they are supposed to feel about a certain thing. So if the nurse manager is also part of a more blamed culture kind of system, it's very easy for that to trickle down to the colleagues lah. Liike at the end of the day, because you know that the manager will be looking for someone to blame. So you naturally don't want to help as much, because then that's more like you have your fingers in more pies. So like more things are traced back to you as well. And If you don't believe that people are going to give you the benefit of the doubt, you feel less inclined to give it to other people. and that only gets stronger when you have people in higher positions of power feel the same.
- **NJCL: For you, is it right for me to say that you feel that nurse managers play a sort of role model for the ground nurses?**
- P16: Yeah, yeah, definitely.
- **NJCL: Okay. Because, earlier, you mentioned something about like nurse managers can say they support self-care but they need to actually make an effort. Could you elaborate on that point?**
- P16: Like, in terms of scheduling like, you'd want to see that they are taking like what you saw into account. Like, let's say, if you're like requesting for shifts, or even when like, let's say, with mistakes or emergencies, you want to see that they- how to say? You want to see that they are kind of looking to understand you from your point of view. And they are trying to make it work, because, definitely like no one can accomplish everything that everyone wants. Because yeah, there's like scheduling and everything like that. And sometimes you do make mistakes that really are bad. But like it's like, if you don't feel adequately supported- like when something goes wrong, they come to you to ask for your opinion as well or like., if, like whenever a patient complains, if they immediately take the patient side, and like they don't, they don't bother to like really see what's going on. I think, then it could be really hard for your mental health, because then you don't feel like the workplace is a safe place for you to just really come to work, do what you can, and like, be the safety net for other people when, like other people aren’t being the safety net for you, you know what I mean? It's also in the sense of with role modeling, like I want to like if they are like, let's say public holidays. Like some managers will try and put the public holiday next to your off days, so you get a longer break, which is better, whereas some just like make it really stilted like if you have an off day. Then like shift, shift, PH, shift, off. So even when you have it, it’s like, more stilted and you can't quite do anything with the time, even though they've technically given you the time. And yeah. And I also want to see them like doing it for themselves like honestly, it makes me very happy to see when they like go on vacation, and then they come back happier. Because you know then that they understand it as well like, like, they understand the importance of taking time for themselves, and they actually take it. It's not in the sense where they're like, oh, yes, self-care, self-care, but at the same time, you see them like 9 to 9 in the office, and you see them like, just dying in the corner. [Participant laughs here]. Like, you know, like really important for them in that sense, to walk the talk. Or at the very least, you see them making actions where it's clear to you that they are trying to support you where they can instead of just completing their managerial duty in like you need a PH, so I'm gonna just slot it in somewhere, like, yeah, kind of thing.
- **NJCL: Okay. Understand, yeah. Nearing the end already. How do you think your institution can help you to self-regulate a healthier lifestyle?**
- P16: [Long pause]. Well, one thing that they've already done, I'm not sure if it's an institutional thing, it might be a MoH [NJCL: MoH: Ministry of Health] thing. But like, they introduced the sick leave without MC. Like the time where, if you just really feel like you have a headache coming on, and stuff like that. There’s a max amount of days but you can just request for off without going to the doctor, and in like. just like taking the MC which like really- I mean at the end of the day, if I really was sick and, like I am, I can like go to a doctor anyways? I think that the introduction of such a thing, of like the not requiring an MC, it really does feel like kind of like a gesture of trust. They trust you enough to know that if yes, if you're really sick, then you'll call, and if you're not, then you'll obviously come to work, lah. It's like an olive branch of of sorts? Other than that, I think- in terms of supporting mental health, I know that my institution has like mental health activities. Like, I know, there's a monthly mindful something like sometimes it will be drawing, sometimes it will be like crafts, sometimes it's like a dance or whatever. And I think those kind of help as well for people who need a stepping board in the sense where they know that they need to regulate themselves a bit better, because they're like not being the best at like work and their life, but like they don't know where to start so that could definitely be something to springboard into and there's definitely that. But at the same time I don't know a lot of people who actually go for such activities? Because even when you schedule such things we have to go on our days off. We have to make it work like during the shift, and like with the fatigue and everything, not many people will want to like take off and then go for these things. So they could also have this thing where- this is quite farsighted of me, because I do know that everywhere is very short-staffed, but like they could also have the thing where I know there are some companies who have like- well, they have it on Fridays, but we could have it so like on one of the shifts every few months. It's kind of like a mindfulness or like a self-regulatory day where you could either go to this activity or you could you could go back to work which, like in that case, like most people, will be going for the activity. So then it will be more encouragement for them to like really go out and do the thing because it's one thing to say like you should do it more, and like to have the activity there. But it's another thing to not acknowledge the time and fatigue that actually goes into making these things work. Yeah.
- **NJCL: I'm just curious. Are there any workplace health promotion programs in place for your institution?**
- P16: What’s a health promotion program?
- **NJCL: Hmm. For example, maybe like they organize activities for nurses, encourage them to workout. Something like that.**
- P16: I think so. Like there are the mindfulness activities. Then there are also like a bunch of physical activities. I think they have like, they try to have like 3 to 4 programs a month. So each program will be like, let's say, 2 days of HIT. Maybe in like one week, one week, and then another one will be like every Tuesday, for like one month, or like another, one will be like, I don't know, Vinyasa or something, but like just one day inside the program. And then they regularly do that and send it out through our emails. So like, that one is definitely they organize. But the bulk of those that I see is more in that they connect us to already existing physical activity, like if there's a charity run or something, then they'll ping it to our emails. Or like if there's the opportunity to like join like some- I think there are like band performances by disabled people or something like that. They will also like, ping it to our email. And I know all of these things are not like organized by them. But it's kind of like letting you know that it's there and giving you the link to like access it as well. But yeah, I've never tried them, so I wouldn't know.
- **NJCL: Considering you don't really participate in this kind of like events that they have organized, would you say it is ineffective?**
- P16: It’s hard for me to say, because I am one person. And, I tend to have a more close knit circle so I don't usually tend to go for more social activity. So I do feel that it's just not quite suited to me, but definitely it is a step in the right direction, staff would probably benefit quite a lot from these things, and I don't know about the exercise programs, but I do know that the mindfulness ones occasionally get sold out so I can say that they are effective, like there are people going for it. And I think, like my department has- not my department, but, like my hospital has like inter house games and stuff like that. And I mean, they post pictures. So like, there's definitely people going. [Participant laughs here]. So I can’t say they’re ineffective. Yeah, but maybe just not all encompassing. But, okay lah.I don't think that’s a bad thing as long as you have like a variety of different things. It doesn't have to be everything for everyone. As long as there's something for someone.
- **NJCL: What do you think will personally make you want to go for these kind of activities?**
- P16: Hmm. I guess maybe if my colleagues wanted to go, or I knew someone who was going for such an activity. So yeah, and like if my shifts lined up. And it wasn't too complicated, because, like for those, maybe like, one to 2 sessions I can request. But for those like, let’s say, one program is 4 times, so 1 time a week for the whole month, that gets a bit hard to like request into my roster and stuff like that, and if I don't feel that I can make it for all the sessions, I don't really feel as inclined to go, because I feel like someone else would like, make better use of this slot. So maybe like a, not so much package, but more like subscription days like you go for like 4 sessions, but it doesn't have to be like 4 consecutive. You just go when you have, like the time and stuff. I think that would really help scheduling and make it more accessible as well.
- **NJCL: Thank you, I see.Just to summarize what you've been sharing so far. Regarding like how your institution can help you to self regulate, you mentioned the sick leave without MCs. There’s also like mental health activities, mindfulness, kind of activities that are being organized that could be used like a springboard, to actually tell staff if they need to self-regulate themselves better.But then you also mentioned that there's a lot of difficulties with this kind of activities. Because of like scheduling and also the fatigue, and some people might simply not just be interested in these kind of activities. Then, regarding like workplace health promotion programs, you mentioned this mindfulness activities like 3 to 4 programs a month, there’s even a band performance that are like, all of these details are being sent to your emails. It’s not organized by the hospital, but it’s letting you know that there are these activities. You think it's a good start. At least there's something for people to go to. But personally for you, you say it's not really your thing, it’s not suited for you. But you do see pictures of people going for it, so you can't say it's not ineffective. Then, when I asked on like what they can do to make you want to go for this kind of activities, you mentioned that if there are colleagues or people you know, going for it, you might just join them if your schedule actually lines up. And then you suggested, like, maybe it could be subscription based. So you can just go whenever you're free and not consecutively. Is everything correct so far?**
- P16: Yeah.
- **NJCL:Do you have anything else, you’d like to add in like how your institution can actually help you to self-regulate?**
- P16: No, lah, I think that's about it. Just basically like, make the time.
- **NJCL: Make your time. Sorry. Could you elaborate? Make the time as in…?**
- P16: Like I was saying, maybe like, have those things where, like. you can have a half day and then go for the mindfulness thing, or you can just stay for the whole shift. Like, make these activities more mandatory because, as of now, they feel more like compliments in there. Mental health is important, self-regulating is important, but it's something to be done on your own time. So we're gonna give you email, we're gonna give you possible things that you can go for and stuff like that. But at the end of the day, it's just kind of like, oh, it's your responsibility you have to like, go and do that which is like- I mean, it's not not our responsibility. Like definitely, you have to take an interest in your own self-care and stuff like that. But if they really wanted to push it to us then, like, I said, you could have a thing where, like whenever you can like, there’s like 2 hours- like we do it for in-services, we do it for meetings and sharing sessions as well, you can have it, for like a mindfulness session and really just bring it into the daily life, where I think then, my colleagues and I would see that it really is something that they care about instead of something that they just say that it's important. But it's kind of like up to you.
- **NJCL: So you believe that the institution should actually be more forceful when it comes to…?**
- P16: I don't think can be called forceful. It's more like- How to say this? Because forceful kind of connotates that instead they will make you do the mindfulness activity which is outside of working hours, and I very greatly believe in a work life balance, I mean, more like: in the sense of like. It's 2 hours of worktime, and they are like, you can go, or you can. If you're really not interested, you can go back to shift that and do stuff. I know some colleagues who would be like more interested in doing that. Because, like you know, like, maybe they're just really not interested in, like the stuff that's going on, but just having the option there and just having it not be something that you have to make a concerted effort of scheduling an extra time for and just showing that you are doing it like, maybe, if, like my nursing managers and, like my colleagues, actually did it like, it would be like a lower barrier to entry, in that sense. Yeah. Or like having them come down and talk to us or something, I don’t know.
- **NJCL: Okay? I understand. Do you have anything else that you would like to speak about that was not mentioned in the interview? Any further comments you want to share?**
- P16: Nope.
- **NJCL: Okay, and this marks the end of the interview. Thank you for your time and participation.**
- P16: Thank you. Good luck for your research. Jiayou.
- **NJCL: Thank you.**

-End of P16 Transcript-

# **Interview #17**

Participant and interview details
 Participant number: 17 (P17)
 Date of interview: 12 Oct 2023
 Interviewer Number: Navarro John Christopher Lambino (NJCL)
 Time IDI started: 1230
 Time IDI ended: 1331
 Observational notes: Participant seems alert and cheerful.

Section I: Interviewer’s summary
 The individual interview was conducted via Zoom. P17 is a public hospital ICU nurse, with 1 year of experience in ICU. From her responses, her workplace is positive and she seem to be in a satisfied state over her work and life. She expressed the need to be healthier, but has also mentioned that she is not living a generally unhealthy lifestyle so there is not much to be changed, but she still attempting to be healthier through more exercise. Similarly, she has mentioned that people need to have the intrinsic motivation to have the impetus to change their lifestyle. Overall, her responses mostly reflect the majority of the participants opinions. One interesting point was regarding the difference between lifestyle back in nursing days and now as a nurse. She mentioned that now as a working nurse, after figuring out her schedule, she finds herself rather more free to pursue her interests outside of work compared to her nursing days, which was in contrast with the other participants. Overall, her demeanour was cheerful and she answered the questions with comprehensive responses that can be easily linked and understood back to the question.

Section II: Transcript

- **NJCL: Okay. Good afternoon, miss. Firstly, I would like to thank you for taking the time to speak with me today. My name is Chris, and I'm a NUS 4 Year nursing student. The aim of this interview is to get a better understanding of the perspectives nurses have on self regulating their health behaviors and how various factors may influence this. I want to find out what are your views, opinions, and experiences on this. I would really like to encourage you to speak what comes to mind. There are no right or wrong answers. May I have your permission to continue with this interview?**
- P17: Yeah, okay.
- **NJCL: Okay, then, let's start with some basic questions like, which ward are you working in right now?**
- P17: Oh, I'm working in [Public Hospital name], surgical ICU.
- **NJCL: Could you tell me more about like, what kind of work you have to do on the daily?**
- P17: Oh, so the the I mean usual, like nursing jobs. So all the sponging of patients, basic nursing care, feeding patients. We give medications. Take charge of the patients. Specifically, in ICU, we take trauma patients. So patients will come from accidents. Yeah, so there's a lot of spinal nursing, wound dressings as well because like we, we have surgical patients to come in. But we also take cardio patients. Yeah, in our surgical ICU. Yeah, I think that’s about all.
- **NJCL: Could you describe your workload? Like maybe the nurse to patient ratio?**
- P17: Oh, okay, so in the ICU it’s either one is to one or one is two. So depends on, it depends on staffing, and then it also depends on how heavy the the workload is for caring for a specific patient. So if there's a lot to do like for the patient, like for example, if the patient is on dialysis like CRT, then usually there'll only be one nurse, or if the patient is ventilated then they will try to like, have just one nurse up. But sometimes because of manpower, so it might be, you might have to take one ventilated case and also one simple like PCI case cause I mentioned we take cardio cases as well, right? So we have those urgent PCI. So usually, those are quite stable, because they're not ventilated or anything. Usually they are left for like monitoring for the first 24 hours after their stents. So yeah, usually, that's about workload.
- **NJCL: And then, for you, would you say it's manageable?**
- P17: I think there are some days when it is more stressful than others. Because some days, even though you may get 2 patients. But 2 patients are also like relatively stable, but some days you get one patient and in ICU, you cannot expect what kind of patients you get. You may get the very unstable kind. So like I mean, even like just yesterday- especially admissions. So when I took an admission, but the patient is so sick that you keep having to have like STAT medication, like almost like almost every hour. I think my patient was fitting as well. So you can imagine, like you had to call the doctors. The doctors are coming in, there are new orders coming in constantly. So then you have to react to the situation. And yeah, it's like I couldn't finish my work until over time lah. I was suppose to leave at 8:30. But I left at 10 because, like, there were just so many things that I had to do even with one patient. So I guess there are on and off days.
- **NJCL: So would you say, overtime is quite common for you?**
- P17: Yeah, yeah, it's quite common, like, quite common, that, like, you have busy days. And then like- busy weeks, and then after that, maybe you may get one patient that is like, oh, easier to manage. But in our ICU, we have the culture that we will help our colleagues as well. So if you are like not so busy, then you will help another colleague who is like, having a patient who is like, very sick. And you’ll just help them with like, basic care and stuff like, yeah, cause, yeah, we are one to one. So we don't really have like a junior per se, compared to like general wards, high dependency? So your colleague will volunteer to be your junior, for example. Yeah, so yeah, you get used to it. But I would say that the stress is still there, like it's just a constant adrenaline rush, especially like when is like a busy resuscitation. Or if your patient is very very sick and you have to be on your toes. Like to watch, like the vital signs, to make sure that nothing fluctuates too out like control and to manage that. So yeah. [Participant laughs here]. I don't know how else to describe it. Yeah.
- **NJCL: I see.Okay, thank you for that. My next few set of questions will be regarding lifestyle in general. So as a nurse, how would you define a healthy lifestyle?**
- P17: As a nurse, how would I define a healthy lifestyle? I would say like eating well, taking care of other well-being aspects. Because there’s also like mental health as well, right? I think it's so important as a nurse, because you already working shifts and especially like I don't know, night shift is particularly hard like as we age right. Because, you want to sleep. but then in night shift, you have to stay up, and then especially the transition from like post night to either morning or afternoon shift, it’s so difficult. And then sometime’s right we don't have a lot of time for our own family or our friends, right? So I think, mental health is very important. Yeah. So I guess, like healthy lifestyle will be like eating well, making sure you have avenues to take care of yourself, self-care. Er, what else? Um, yeah I guess these two things.

**NJCL:Earlier you said something about taking care of your other aspects of your well-being, such as mental health. Would you elaborate on that?**

- P17: Oh, okay. so like, knowing what like helps you to like, relax, or like what helps you to like take your mind off work. I mean for for example, right like in my line of work. Like especially in ICU, it’s like when you are at work, you are fully present for your patient and it can get very intense at times. So it's like a constant adrenaline rush, right? But then it’s very strange because once you’re off work, you're off work. You don't care about- I mean. Not you don't care lah- but it’s like because you’re not in charge of the patient anymore, so you're back to reality, you're back to your own life. And then you have your own responsibilities in life to handle. So it can be very stressful if you don’t know how to switch on or off, like between duty and off duty. And then also like, sometimes like, the adrenaline rush is still there even after your off work. And it can get very stressful so, you have to learn to like- okay, some people cope with like listening to music. But for me, when I get off work right, when I get on the bus, right? Then I will try to listen to very very calming music because I find that it helps me to de-stress. I would say like for some people, exercise helps. Like I have colleagues who go for yoga and pilates. Personally, I don’t exercise a lot. I do try, because it’s important. But it’s not the best yet. [Participant laughs here]. Like, I don’t know. Just once in a month kind of thing. Yeah, then there are other things also. Some people will watch like Korean dramas. Or spend time with friends and families. Whatever that helps you to relax and take your mind off the stress loh.
- **NJCL: Okay. I see. Then, earlier, when I asked about healthy lifestyle. There were 2 main answers: eating well and making sure you have avenues for self-care. Could you elaborate on a second point?**
- P17: The self care one?
- **NJCL: Yeah.**
- P17: Wait, just now, it was the same question?
- **NJCL: I guess. Yeah. Cause the the previous question, you were saying something about taking care of your mental health wellbeing, so, I'm guessing what you meant for the second point of like making sure you have avenues for self-care, is talking about other than taking care of mental health. Maybe like, emotional aspect of self-care?**
- P17: Oh, okay, okay. yeah. I mean, like other aspects of self-care is like making sure that- okay, like, I mean, like for girls right, you want to go for facials. You want to do your hair. We can’t do our nails because we’re nurses. Doing fun things lah, things that like you know, you’re still taking care of yourself. Yeah. Like, to make sure that even though we’re having a busy schedule, right, but then yet you are still taking care of your personal well-being, whether it’s grooming- like it’s very important. We still have to feel that we’re put together. Ya, how you look in the outside will affect how you feel in the inside also. Self-care… I mean, you’re sleeping well? Sleeping well is another aspect- so important. Ya, I guess like basic things loh. Eating, drinking enough water. Drinking water, that’s something a lot of nurses neglect because we don’t have time to drink in between our shifts. Yeah. So like just taking care of very very basic things that we also need as people to live, or survive.
- **NJCL: Earlier, you said personally for you, you don't exercise a lot, only maybe like once a month kind of thing. So could you elaborate how you feel about that? Do you actually feel like changing that current physical activity level?**
- P17: Yeah, I don't think I'm the healthiest in terms of like exercise. [Participant laughs here]. The recommended is like, 3 times a week, right? So like, personally- okay. So when I was younger, I don't have a habit of exercising. It's not something that is from like my family as well. It’s not a habit that I have from young. So, in fact, actually, I used to hate exercising so much. Like, when I was younger, you know, you are like overweight, then they put you in the ‘Tough club’ [NJCL: Tough club is a physical activity program for overweight primary school children in Singapore, which encourages and organizes extra sessions of exercise for these children to participate in], make you run like many rounds, so I really hated it lah. But as I got older, especially since uni right, you recognize that it is important to put in exercise. Actually, I’ve been trying to find more avenues of exercising. Like in the past, I use to go to F45 [NJCL: F45 is a commercial gym that has very intense group, high intensity workouts], like once a week.[Participant laughs here]. And it was quite a lot actually. And I was quite fit at some point. But the only problem was that because it was those kind of fast paced, HIT exercise, it made me very stress also. I think it’s because like, my work and my lifestyle is already very packed. Then you couple it with this exercise that pushes your adrenaline into the highest level, right. So I became very very stress overall, and I found out that this workout is not for me. So then, I started to find other things. I like to do pilates. I used to do home pilates, whenever I can. Like just on YouTube, find a instructor, do some pilates. And I think it helps, because it’s a bit more slow-paced, kind of exercise. You get to breathe, you get to relax. And I guess like, that’s like part of the exercise as well. Same as yoga. Like they will always tell you to focus on your breathing. So, I like those kind of exercises. So then, recently, I signed up for ClassPass and I’ve been trying to go for pilates, so that’s why it’s once a month. Because of the number of credits that I have in my ClassPass. Like, I have like 20 something? And each pilates class is like 14? It’s quite expensive. So I can only afford to go like maximum 2 a month. Then with my schedule, I can only find once a month. So, it’s not ideal yet. I guess like, as I go along- I mean, this is just the beginning stages, right? Just to be honest, ClassPass is only like 3 weeks ago then I began to discover; oh, this is something I can try. So, still figuring this out and, as it goes, I do hope to increase the number of frequency of exercising. Even considering like doing home exercises and workouts outside. Yeah. But for now, it’s more like the work schedule, so yes. [Participant laughs here]
- **NJCL: What what do you think is your main impetus that made you want to improve your physical activity?**
- P17: I think it's like, to be very honest is like, when you get older you realize your metabolism slows down so like [Participant laughs here], if you don't exercise- in the past can eat anything, right, and you won't gain weight. But now it's like. if you don't watch your diet, and you don't like, and and you don't exercise, you realize that you can gain weight very, very quickly. And it's very difficult to like lose weight. So I think that was like one of the motivations for me. Like, okay, I need to have an avenue for getting some exercise in, if not I will really keep on gaining weight. And it’s not healthy! And I guess another thing is that because, you know you have your annual health screening, right? It's every year. So then, my cholesterol levels on the like borderline high level. and I didn't think that it was bad. But then, like my my colleagues saw it, right, and then they were like, ‘oh, how come your cholesterol levels are so high?’. Yeah. And that was when I thought I was very healthy already because I'm not like very overweight, right? And I feel like compared to like, maybe my peers, my work requires that I walk around a lot. So I thought, like, okay, maybe like, it’s not so bad. I’m considered healthy. I don’t have any chronic illnesses. But then, like, oh, my cholesterol levels are like on the higher side, and then compared to my colleagues like they're in a healthy range. So then, that’s where I realized that, okay, like, maybe I need to start like being healthier. If not, this will get to me in the future. Right? Yeah, I might not have hyperlipidemia now, but I will have a higher risk for it in the future. And I don't think I want to get it like, young ah, so, yeah. Which is one of the factors. And then maybe like a bit more recent things, is that I'm getting married next year, so I don't want to like-
- **NJCL: Congrats!**
- P17: Thank you. Oh, yeah, so like it's like, I don’t want to look overweight in my wedding photos, just to be very real. [Participant laughs here]. So I guess it’s just one of the things- yeah these 3 things.
- **NJCL: I think earlier, you mentioned something about your diet as well. So specifically on like dietary habits, how do you feel about your current lifestyle?**
- P17: I think I'm not sure. because, like. I don't think what I'm eating is like, like, you know, the the ideal, the recommended, like how many like- you know, some people count their macros and stuff? So, I don't know how to count my macros. I'd been trying, I asked many fitness like friends, but I still don't get the science behind it, or like the math behind it. I don't know. I just cannot figure it out. So, anyways, I just try my best to like, you know, not eat like deep fried food. Like avoid, deep fried food. Try my best to not eat so much eggs. In the past, I used to be like- every meal has to have an egg. But now I will try to, like, can I substitute it with like tofu or like beancurd, to have like a healthier option. And then like, eating smaller portions. I think when you take out right, like they always give you a lot. So maybe if I’m in the mixed rice stall, I will ask for less rice, like half portion rice. Eating less rice- I wouldn’t say it’s the best lah, but I would say like, I do make efforts to reduce like my cholesterol intake for example. Reduce like the calories and try to be a bit more watchful. If it’s like on my off days, I’m home most of the time, then, I try not to eat too much because I wouldn’t burn as much calories as compared to when I’m at work. But if I’m at work, I would eat lah. I would eat to make sure I have energy to sustain myself. And like for night shifts right- okay, I wouldn’t say it’s the most recommended, like night shift- most of us would eat. Even late into the night. But I tried before, not eating before the whole night shift. And I found that, it was very very hard for me. Because I didn’t have the energy to sustain the 12 hours. So, like while it’s not recommended to eat suppers, but especially for night shift, then I realise that I need a basic level of glucose, so I need some snacks to eat. So I will try like sandwiches, like small portions, not full meals. So like, eating what my body needs but also watching, to make sure it’s not too over.
- **NJCL: So I'm assuming the reason you are maybe more conscious of your diet is similar to why you're also trying to be more physically active?**
- P17: Yes, yes.
- **NJCL: Okay. Then, how would you say your lifestyle changed after you started to work as a nurse?**
- P17: This is like general or like with like the exercise, and…?
- **NJCL: General, so like beyond exercise and diet and like may maybe your mental, emotional habits as well?**
- P17: Okay, I think you get used- okay, I initially like, in my first one year, I really struggled a lot. Because I found that, right, when you work shifts, you’re opposite like from everyone else. Everyone else is like in the morning they are awake, then in the evening, they are asleep, right? But shifts you don’t have that luxury. So I think I struggled a lot with like planning my time. Like, when do I have like my self-care days? When can I like slot in a workout? When can I go out with friends, or spend time with family, for example? And then, in terms of eating habits as well, because I won’t have a fixed meal time because of my breaks. My break timings are staggered right, so I won’t have fixed meal times. Night shift also, I won’t have fixed meal times. So how can I take care of myself in that aspect? So that is different from before I started work, right. When like uni, the normal schedule is like you wake up in the day, you sleep at night. And then you have regular breakfast, lunch and dinner. Sometimes supper. So I think it’s just figuring that out, like you know, when you wake up- my breakfast timing is different from everyone else and that’s fine, you know, because that’s like my body’s rhythm at that point of time. Because of like the schedule, and so that’s is different. And then, you have to be very intentional in making sure that your off days are like okay, maybe I meet my friend and like- and then on the day when I’m free, I do my self-care. [Participant laughs here]. Yeah. And then, I mean, outside of work right, I’m very very involved in church. So like, I have a lot alot of activities, either is it planning for things, or going for events. So that’s the other things that I have to factor in, and how I try to manage that is like- at work we have this quite good system that we can roster requests, right, so the culture at my ward is good in a sense that, if you request for a morning shift, they will try their best to give you a morning shift. So I hadn’t had problems where I had try to request for a day off, or a day that I can go for classes, either exercise class. Currently I’m learning Japanese, so I have Japanese class as well. So, I hadn’t had a problem where I couldn’t go for these things because I wasn't granted the shift that I wanted. So yeah, I would say that, once you get used to like, figuring out your pockets of time and everything. You can actually do more in pursuing your interests in things, and even hobbies. Self care actually, exercise. I think right now in my life, I’m in quite a healthy rhythm of things. Like, I have a healthy balance of work, and then as well of other things that I’m doing outside that you know, like I feel like I have a life. [Participant laughs here]. So I guess, ever since work started, I actually realised that as an adult, I could pursue more of my other interests outside of work. So, which is a lot better than in uni. I don’t know why, also. Maybe because when you’re studying, you’re just focused on studying. So yeah. Then, when you go out to work, you realised that outside of work, you don’t have to think so much about work things anymore. So you have a bit more space to consider other things outside, so yes. I hope that answers your question?
- **NJCL: Yeah, it does. Very comprehensive. Earlier., you said like you were quite involved with church. So does this mean? You were already involved with church even before you started working as a nurse?**
- P17: Oh, yes. In my student days as well. Yeah.
- **NJCL: So for you, you actually find it more manageable to manage interests outside of work when you started working as a nurse compared to when you were a student?**
- P17: Okay, maybe manageable is not the word. Let me think, how can I phrase this? I think that somehow, like when I was working versus when I was a student, I have a bit more space to consider like interests outside of work. And somehow, like, I have a bit more time. Yeah, I don't know. It's a bit counterintuitive, now that I say it. But somehow, like when you're working, shifts you still- when you when you figure out like how do you use the pockets of time, right. Then you will realize that actually I have a lot more time, because, like, for example, I have weekdays off, or like my weekdays are free, so I can go to like do things while other people are working, right. Then I wouldn't say, it's more manageable. I think that is equally as difficult, actually, in terms of like, like, how I feel about it. Yeah, because, like I mentioned, I struggled like quite a bit like in the first 2 years, right? So I can say this now, because, like, with relative ease because, like I've been through the whole process of like struggling and figuring out. I guess it's like over the years, you gain that competence of like figuring out your time and then now that you are here, then, it’s like okay. This is how it is, and, like now it’s only manageable. It’s only manageable now. Like after figuring it out, yeah.
- **NJCL: Is it right for me to say that planning your time is a lot more important?**
- P17: Yes, yes, definitely.
- **NJCL: Okay. Okay. And then my next question is like. what do you think are your main facilitators or motivators to self-regulating a healthier lifestyle?**
- P17: Facilitators and motivators… Let me think ah. [Long pause]. I guess the first one, health related, right. I want to be healthy, even when I'm older. Like, I guess, that kind of mindset. Because, like every day, you see, all kinds of patients right? And like, especially like- okay, I mean, before, when I was working. ICU, I was working in a stroke unit. So like the patient that come in are people with stroke. But they usually have past medical history like diabetes, hyperlipidemia, hypertension. And some of them are very young, like I’m talking about 50s, 40s even. So right, it makes you think. It’s actually quite scary if you think about it. If you don’t care of your health now, then you might get these things early. Forty, fifty. And then you end up like that. And then you start to think like, I don’t think this is the quality of life that I want. So that’s quite a big motivator to me. So telling myself that I need to be as healthy as possible. I want to be able to live a good quality of life for at least many more years. So yeah, I guess that’s the first thing. Actually I think, that’s maybe the biggest motivator. I can’t really think of other things, right now.
- **NJCL: It’s okay. And then, moving away from facilitators, motivators. In terms of barriers, what are your main barriers in self-regulating a healthier lifestyle?**
- P17: Busyness. Especially like when- stress and busyness. There are like weeks or months where work is very very stressful. So then, on those days, you really feel like, ‘wah, I’m so tired already, I don’t want to do other things’. Then you start to get- okay, I wouldn’t. Quote on quote, maybe it’s being lazy. But I think also, it’s an aspect of like trying to manage- knowing your body, lah. Because there are some days where you’re really really tired. So you will ask to maybe reschedule things, you want to sleep in more just to make sure you have enough energy to just sustain work. Because it is so so tiring. Then you will lag behind lah. For example, maybe you are planning to go for exercise, but then the day before, you had a very very tiring shift. So you decide, I’m gonna put off exercise, for example. Or you had a very very tiring shift, after work you are like ‘I need to reward myself! Let me go and have an icecream.’ or like ‘let me go and get bubble tea’. Then you will be a bit setback in your diet. Maybe I’m suppose to go out right, but then I’m so tired, I just want to stay at home. So, I guess, it’s just stress and busyness can be quite huge barriers. I think it’s just these two main ones.
- **NJCL: By busyness, you mean, conflicts in schedule?**
- P17: Sometimes, conflicts in schedule. Sometimes it’s just back to back things. Like after work, then I’m going to this other event. Then after this event, I’m going to somewhere else. Then you know, it’s like I have a very very long day, and then the next day, it’s work early in the morning, kind of thing. And then maybe it happens for a few days, consecutively. So, sometimes it happens. But then busyness also in terms of- because work is very physically demanding. So, maybe like, for example, I have a very very busy patient, right, like I mentioned earlier, my admission is very very sick. So then I have to be walking and moving around a lot, then your legs and your whole body is aching. So yeah, I started to have this backache also. Like, I don’t know. Nursing hazard. So then, you start to like feel body aches, because of the busyness that you were having at work. So yeah.
- **NJCL: Okay, then I’ll just briefly summarize what you shared with me. So for you, for your main motivators or facilitators that you shared with me is you have this: ‘I want to be healthy mindset’. Because after maybe, seeing your past patients, the kind of patients you're getting, you don't want to end up like them. So you are motivated to actually be healthier. Then for your barriers, it’s mainly stress and busyness. Beause, for example, when you had a very tiring shift, you don't feel like doing anything else afterwards. So maybe you have a shift, and then you have like, outside responsibilities that you need to go for, you don't really have time to like, maintain your own self. So because of these barriers, it's a bit harder to maintain a healthier lifestyle for you.**
- P17: Yeah.
- **NJCL: Other than these, is there anything else you want to add for motivators or barriers?**
- P17: [Long pause]. Can I say that friends is like a motivator for, like healthy lifestyle? Because, like I have friends who like, for example, like, if they're going for exercise class, then they'd be like, ‘do you want to come along?’. Yeah. So I think that helps. Or like other friends who, ‘oh I have a cooking class going on’, can bring people. Then they’ll bring you for these kind of activities, so it helps you just get your mind off work as well. So I guess, friends?
- **NJCL: Okay. Anything else?**
- P17: Nope, I think that's all.
- **NJCL: Okay, alright. Then my next question is, how does your self-regulation habits impact your work performance?**
- P17: When myself is well taken care of, And I know that, like, you know, I'm okay. I'm actually a lot more efficient at work. A lot more efficient, and, I think like your outlook on work- even though it may be like, you know, you know, you're gonna have a tough day. You know you're gonna take a very heavy patient, but you are still optimistic, and you're still cheerful while you're doing your work. But when, like. it's been a long time since, you know, you've taken care of yourself. And you’re like, super super stress. Then you can feel the difference because it's harder to concentrate at work. You still get the work done but maybe it’s not like at the optimum efficiency that you can actually function at. And, you feel very frustrated even at the smallest things. So, ya, I think that’s what I noticed about myself, lah.
- **NJCL: Does this apply outside of work as well? Like, how how do you think your self-regulation habits impact you outside of work?**
- P17: Oh, for sure, like it affects outside of work as well. I am generally- for example, right. I mean, I am generally quite a patient person. I don't get angry easily. But ever since working in the ICU, I do realize that my temper is a bit shorter. Especially towards family, sometimes towards my fiance, for example. Friends as well. You realise that you have a lot less patience. Because you already spent your emotional energy somewhere, so it’s like you spent a lot of your emotional energy at work. Like taking care of your patients, taking care of patients’ families even. So, if I don’t take care of myself, I realise that the people- not just work right, not just my patients but my family, my friends, my loved ones are affected as well. So, yeah, I guess, that’s answering your question?

**NJCL: Yeah, it does.Do you use any tools or apps to help self-regulate your lifestyle?**

- P17: Let's say, no, not really. Yeah. I mean, not like, specifically like apps for like a- like to plan out like the whole day kind of thing. So, I mean, there are like- I used to. I used to try, but it didn’t really work out for me. Because like, I wouldn’t like log in everyday to chart my water intake, how much I’m eating, how much I’m exercising. I used to try that, lah, for awhile. But it’s not sustainable for me, so I didn’t continue.
- **NJCL: Okay. Just an additional question regarding what you've shared so far. So what do you think will increase someone's determination to change their self-regulation habits?**
- P17: [Long pause]. I think they need to have- the first most important right is that- okay, I don't know. I guess this is the word? But they need to have like intrinsic motivation they need to, they need to know for themselves that I need change. Because if they don’t realise that, no matter what like, how ever like other people try to motivate them, others things around them to motivate them, they will not be moved. So I guess, the most important thing is that they need to have a good reason for themselves to decide that okay, I need change. Then, I guess the second thing is- sorry, sorry, what’s your question again?
- **NJCL: How can you increase people's determination to actually change their self-regulation habits?**
- P17: Okay, okay. Increase determination… [Long pause]. Yeah, okay, I mean, back to the first, like, the intrinsic motivation, right? So then, I guess the most important thing is that they need to have awareness of where are they at, honestly. And like, what is the healthy standard. Not saying that- I don’t think everybody will be able to reach the healthy standard, right? But at least, you have some sense of like, ‘okay, what I have now in my life right, maybe this part is not good; I need to change’. So yeah, the awareness comes in. For example, I told you, oh I saw my cholesterol level then I was like ‘oh okay’; I was quite shocked by it. Yeah. So then, the awareness will help people to be more determined. Um, I guess like, taking baby steps to encourage them to a healthier lifestyle. And to realise that- I guess finding what works, and when you find what works, you’ll be more motivated to realise that: ‘oh actually, this is an easy step. Okay, I can do this.’. And then once they achieved this small goal, then they realise, okay, maybe I can set another goal. Specifically for me, will be like the workouts loh. Some people really, you know, they can go to the gym. They can do HIT workouts, no problem. But I realise it was stressful for me, and like, it’s very very difficult. So finding that- maybe yoga and pilates is something that I like better. So, it’s a better motivator for me to go exercise. And it’s fine, that it’s different from other people. Yeah, I guess. Then after that, maybe the other point that can help people to be more determined is to encourage them with the idea that like everyone’s- I mean,how everyone becomes healthy will look different from another person and that’s okay. Find what works for you, and stick with it. Yeah. Uh.. I think social support is very important as well. Having friends and family that support healthy living, or do it together with you. Because if you feel like you're doing it alone, then it’s going to be very very difficult for it to be sustainable. But if you have people to journey with you, then it’s a lot easier to go through with it. Yup. I think, that’s about all. Yeah.
- **NJCL: Thank you so much. Very comprehensive. Okay, my next few set of questions, is regarding the workplace in general. So as a nurse, how do you think the workplace influences your own self regulation habits?**
- P17: Can you elaborate more this question, like for example?
- **NJCL: For example, maybe, due to you’re roster, you’re unable to go for activities- like workouts you’ve already planned.**
- P17: Okay, okay, so how the workplace affects? Okay, okay, okay. Okay. maybe on the bit more positive side, right- I mean, the kind of colleagues that you have. So my colleagues are quite like friendly. Or at least I found the friendly ones that will go like: ‘hey are you free at this timing during your off day, do you want to go exercise together?’ for example. Or ‘hey, lets go out and do this activity’ for example. So I think right, the culture of the workplace. If they are- if they bother to make friends with you and bother to like, ‘hey you’re my colleague’ like, ‘lets go through-’, like, like- life is difficult at work, so let’s help each other. Yeah, so like, that kind of culture actually is a good thing. It helps, with self-regulation outside as well, because you're doing it together. Yeah, I would say that shift work makes it difficult. The shift work does make it difficult, I mentioned it before in this interview, that it’s difficult to plan time. Yeah, I guess the good thing is the workplace culture, the bad is the shift work. I think just these two, for now.
- **NJCL: So you feel that the workplace culture among nurses actually helps to facilitate self regulation of healthy lifestyles?**
- P17: Yeah. But not everyone gets the same experience because it really depends on who your colleagues are. So I would say that, like, I'm quite blessed in this environment right now that I have colleagues who care about like other people's well-being. Because there are people who, like, you know, they go to work, they just work. And then some wards they have a very toxic culture. Yeah. But if you, you have a culture where, like your colleagues do care about you as a person not just like, you know, what you can do for them, or like to make their life easier at work kind of thing. Yeah. So then they would bother to like to care about the other aspects like of your life outside of work as well. Yeah. And they will bother to, like, you know, want to ask you out to to make sure that, like, you know, we're in this together. Yeah.
- **NJCL: Earlier you mentioned, toxic culture, right? So how do you feel about a workplace culture among nurses that hinder self-regulation of healthy lifestyles?**
- P17: So like there are workplaces where you’re colleagues will like, you know, quite common lah, people will talk bad behind your back, right. So for example, if someone keeps criticising you for something- I mean, I heard it before. I heard it before in my experience, that someone is commenting is on a person’s physical appearance. They feel like this person is overweight, and they start to label this person as like, oh because right, this person is very overweight, therefore, that’s why so lazy at work ah. Don’t want to walk, don’t want to do anything. So I think like, it’s not very healthy. Because it’s like, you don’t know what this other person is going through right? You shouldn’t be commenting about someone’s else’s lifestyle, or like whatever. So it’s very demoralising not like to that person, but to other people who hears it, right. Because you’ll be like, oh, are other people talking about me like this as well? You know? So this toxic culture makes you feel very stressed also. Like you’re constantly, on your toes right. It’s like you’re not even focusing on the work, but you’re just like what are my colleagues talking about me? And then they start to comment about your personal life. Then it’s like, what has that got to with your job? So the toxic culture has the effect of making you feel more stressed, making you feel more demoralized. Yeah. Or like, you know, maybe someone has been recovering from an injury, I mean quite common lah. And then I heard before, maybe someone is going through injury, they cannot do certain things at work. You know, as a nurse, you have to do turning, and some of these patients are very heavy, right? But if I injured my hand, I cannot turn the patient. Then because of that- maybe this person, they don’t turn their patients as often. Because of the injury, right? And then you have colleagues who go like: ‘oh this person is being lazy’ or ‘aiyah, the hand injury better already, lah. Confirm not like that’. So I think, it’s very toxic if you hear that, and then as someone recovering, right, you’re trying your best to recover, to get better, to take care of yourself. But if you hear this, then it’s not helping. Yeah. So it’s things like that. Yeah.
- **NJCL: Okay, so just to summarize briefly what you mentioned. So regarding, like the workplace culture among nurses, in helping to facilitate or hinder self-regulation of healthy lifestyles, you said that it can be positive in a way that there are, at least for you, there are colleagues who care about you outside of work, and they treat your well-being as well. So they will actually invite you out for activities together, so that you are less stressed, and maybe get some exercise at the same time, when you guys do the same activities. Then, at the same time, there’s also toxic workplaces that actually hinder your self-regulation because they demotivate you. So you’re demoralized even before you engage in self-regulation, is this correct?**
- P17: Yeah, yeah.
- **NJCL: Can. Then what do you think about the role of nurse managers in helping nurses to self-regulate their own health?**
- P17: I would say, like nurse managers, are quite important to build like, or rather like, maintain, a certain culture within the department. So for example, the reason why my workplace is quite- like for example if I roster request, they will usually grant that request. It’s because my nurse managers, do protect this quite strictly. To them, they understand that nurses have a life outside of work, and if let’s say you do need to go somewhere, and you have requested for like a certain shift, they will try their best to grant it. So I think, because they protect it quite well, so my department has this culture. I cannot say the same for the others, because I’ve heard of my other friends who struggle a lot with even getting whatever that they want to request, because their nurse managers are more concerned about the staffing for that day, for example. Then the second thing would be that, I think, having understanding nurse managers will help a lot as well. Like, for them to be approachable, and for you to be able to discuss. Because, I think, they need to know that, when they’re managing staff, right, they’re not just managing at the work. Because whatever that’s outside of work, right, can affect your performance at work also. For example, maybe in this season of time right, it’s extra stressful for this particular staff because they have family problems, right. And because they have family problems, they have other things, they don’t take care of themselves already. So, the nurse manager, when they see them at work, right, they shouldn’t go with the mindset of like, oh this person’s performance is getting worse recently, oh, I should go and scold them like for this specific reason. But they should ask like, ‘hey is there something going on outside of work that bothers you which cause you to not to be able to concentrate at work?’. And like to try and discuss together with that staff, right, like you know. I think that kind of healthy, nurse-manager staff relationship is important for the nurse themself to be able to self-regulate. Yup, I think, I think that covers almost all.
- **NJCL: Yeah. Okay. Then, my next question is, how do you think your institution can help you to self-regulate a healthier lifestyle?**
- P17: Institution…? [Long pause]. I mean, the regular health checkups are really a good avenue. I'm glad that they have it, and it's like free for like staff. [Long pause]. Actually, I think on the institutional level, like they used to have exercise classes. That staff can actually like enroll in and join in. But ever since COVID happened, I’m not sure if it’s still happening. So actually, I think, like, these are things that are quite good on the organizational level. Yeah, so it’s like whoever wants to join, they can join. I think food choices at the canteen, especially when we’re talking about healthy lifestyle, right, but then if it’s very difficult to even like choose between the canteen, to like want to eat healthily, then it creates quite a bit of a problem. Even if you want to try to get a better diet. What else? [Long pause]. I had something, but I lost my train of thought. [Participant laughs here]. Yeah, maybe let’s just leave it with these few first.
- **NJCL: Okay, sure. Earlier, you mentioned, in the institutional level, they actually organize exercise classes for the staff to join in. Have you personally joined any of these classes?**
- P17: No, not yet. I've been wanting to, actually. So I've been trying to find on the website like you know, when are the available days.. And if I can join in. But yeah, so that's the problem, because ever since COVID, I think they have slowed down the frequency of these classes. So I can't seem to join in for any of these. So yeah.
- **NJCL: I see. Okay.**
- P17: Oh, oh, I remember already. Because I remember reading an article, a research article about like how they try to lower, I think cholesterol levels for like nurses, especially because we work night shifts. Oh, oh, cholesterol and, cortisol I think. Like stress- yeah. So because, especially like night shift right, you know, if we don't sleep enough, actually, your cortisol levels rise as well, and then your body is like very hard to regulate all this other things. So naturally, your metabolism slows down, or it’s not as optimal because of night shift. But it’s not something that we can change, because we just have to work, right? So I read an article in another country where they trialed, they did an RCT. They trialed where some of the wards, you know, during breaks at night, they brought all the nurses that is on break to have like an exercise session. So maybe, 30 minutes kind of thing, yeah. Versus like wards that don’t have. And they found that it actually helped in lowering the cortisol levels. But, I don’t see it in Singapore yet, I don’t see anyone else doing this, or I don’t know if other countries are adopting this. I mean, maybe it’s just a one time article. But I just thought it was quite cool, and something that- if it works, and if there are more RCTs that show that this works, right, then maybe on an organizational level, it can be considered as well. Like during breaktimes, they have exercise sessions scheduled during the breaktime, that people can join in. Yeah.
- **NJCL: So for you, you would be keen to join this kind of exercise?**
- P17: Yeah, if there are, why not? Like, I'll go and try. [Participant laughs here].
- **NJCL: Okay. Yeah, just to confirm. Earlier, you also mentioned like food choices in the canteen. So does this mean, currently in your canteen, there's not much available healthy food choices?**
- P17: I think I would say, like average. There is like a salad store where you know, all the healthy people will go queue for. But then also like- I mean, you get bored of the food choices after a while. Yeah, I mean, even if it’s like- yeah, like you can try lah. Like, I mean in the menu, there’s unhealthy stuff, for example, like things with a lot of gravy. Like, I think we have like a chicken rendang stall, with a lot of gravy. So, I think in the hospital, they do regulate, for example. Because like, there are patients there, right? They cannot sell overly oily food. Or overly sweet, overly salty things. So they do regulate on their own already. But it’s just that, more variety would help? Yeah, I find myself going to the noodle stall more often. But then I guess, personal choice also lah, you would try to like- you will just work with what you have, I guess. I wouldn’t say that the canteen food is totally not healthy, I think do see that they try to make an effort. But then, I think also- like if I want to count my macros for example, I cannot like buy a- [Participant laughs here]. Yeah. It’s very difficult lah. Like, maybe if I go to the mixed rice stall, and I look at the meat choices, but they’re deep fried on that day. Then yeah, it’s very difficult in that sense.
- **NJCL: Other than the institution organizing exercise classes. Are there any like workplace health promotion programs in place?**
- P17: Not that I know of, I think. Yeah, I may be wrong. Maybe I just don't know enough. But yeah, not that I know of. So even if they are, it's probably not very publicized. Yeah.
- **NJCL: Okay, then I'll just summarize what you've shared so far. So when I ask, how do you think your institution can help you to self regulate a healthier lifestyle? You mentioned the exercise classes being organized, but post-COVID, it's a bit harder to find out more information on this. Then you also mentioned food choices at the canteen being limited and you wish for more variety, so you won't get bored with the same few choices, and a bit easier to also like, count your macros if you wanted to.. Then you lastly, also mentioned some research article on organizing exercise activities during night shift. And for you, you don't actually mind going for this kind of activity during your own night shift. Yeah. So other than these 3 things, is there anything else you want to add?**
- P17: Oh, maybe just on the organization level, right. Because I just remember that they do have facilities for like Badminton. Badminton, soccer, because I've heard, like some staff from other wards that they do organize like badminton sessions, soccer. So I think it's good on the organization that they built such facilities within the campus, so that, like, you know, nurses can like go after work. Oh yeah, then I remembered that there was a gym? But I don't think it's very subscribe within my organization. Yeah, so not sure if it's like the equipment, or like people prefer outside. But I just thought, that is a good like idea to have like sports equipment, available for like staff, readily available and like not too expensive, as well. Like, you know, like gym memberships are expensive outside. So, if the gym membership isn’t as expensive at work, I think more people would want to use it, lah. And it matters because- I remember when I was trying to find exercises classes right, I realise that they are all like- because most of the exercise classes are within CBD area, where like all the 9 to 5 office workers are right, but it’s so difficult for a nurse, because our hospitals are not there. And I don’t live anywhere near there as well. So, if you think about it, like it’s easier, let’s say if I’m on the way back from work to home, and on the way, there’s like an exercise place. It makes me more motivated to go- for example, if there’s a gym. makes me more motivated to hit the gym after work and then you go straight home. Rather than it’s so out of the way, then you still have to go all the way home. Then you will feel like, ah I still have to go somewhere else but I just want to head home. Yeah, so if you have these facilities within the work, or like within the campus or even super near to the campus or like, I don’t know- oh yeah maybe this is also something else that I thought of: maybe have partnerships with nearby gyms, to like give discounts to the staff that are working. I think that will help also. Because it would be like a lot easier- like oh okay, there’s like some partnership going on, so okay, let me go and sign up because it’s cheaper. Or like if it’s at work, oh after shift, I can head to the gym and then I’ll go straight home after that. Yeah, okay. I guess I hope that helps.
- **NJCL: Other than gym memberships. And maybe like the availability of the places, is there anything else you would like to add regarding how your institution can help you to self-regulate a healthier lifestyle?**
- P17: No, I think that’s all.
- **NJCL: Okay. Then, do you have anything else that you would like to speak about that was not mentioned in the interview? Any further comments you want to share?**
- P17: No, I don't think so.
- **NJCL: Okay, then this marks the end of the interview. Thank you for your time and participation.**

P17: Thank you!

-End of P17 Transcript-

# **Interview #18**

Participant and interview details
 Participant number: 18 (P18)
 Date of interview: 18 Oct 2023
 Interviewer Number: Navarro John Christopher Lambino (NJCL)
 Time IDI started: 1800
 Time IDI ended: 1905
 Observational notes: Participant seems very relaxed.

Section I: Interviewer’s summary
 The individual interview was conducted via Zoom. For context, P18 is a 25 year old, male nurse working in a public hospital ICU ward. Before the recording even started, participant seemed very casual and openly asked questions. Rapport was established, and a casual environment for the interview was established. His responses are echoed by previous interviews, however, he also provided fresh ideas on some aspects. While he mentioned self-responsibility for one’s health, he also expressed that for change to occur in the first place, people will need a reason to change. It is important to provide people this self-determination to change. When given an additional question on how nurses are seen as role models for patients, he mentioned that it is important to portray an image of healthiness. He does not feel guilty providing lifestyle advice when his own lifestyle is sub-par, but instead it allows him to be able to relate to his patients better. Along with what others have said, the first few months/probation period for new nurses has been the worst period. It was extremely stressful, and coupled with fatigue, makes it difficult to have energy for activities outside of work. For P18’s case, this was true but now that he has gotten a better grasp of his work, he has begun to take a more proactive approach to his health. Interestingly enough, his facilitator/motivator is having a sense of fulfilment with his work as it motivates him to take better care of his patients and himself. Stress is also a major part of this participant’s responses. He mentions that stress saps his mental strength/willpower to be able to do healthier lifestyle choices. Self-regulation habits improves his work performance as he says he is able to be more productive. For how his workplace influences his self-regulation habits, he provides a positive twist unlike most of the other participants as he said that he had made changes to make it easier for him to become healthier. Lastly, he mentioned that while both the nurse themselves and management have a role to play in helping manage their health, he believes that management should be doing a lot more and they have a bigger onus in ensuring that the employees health are taken care of. Perhaps questions on what will make nurses prioritize their health more and if management is doing enough for nurses.

Section II: Transcript

- **NJCL: Alright. Good evening, sir. Firstly, I would like to thank you for taking the time to speak with me today. My name is Chris, and I'm a NUS Year 4 nursing student. The aim of this interview is to get a better understanding of the perspectives nurses have on self regulating their health behaviors and how various factors may influence this. I want to find out what are your views, opinions, and experiences on this. I would really like to encourage you to speak what comes to mind. There are no right or wrong answers. May I have your permission to continue with this interview?**
- P18: Yes, you have my full permission to carry out, in accordance with any ethical regulations that your study may have.
- **NJCL: Alright. Thank you so much. And then let's start with some basic questions like, which ward are you working in right now?**
- P18: Like, what kind of ward? Or..?
- **NJCL: Yeah, your specialty?**
- P18: Okay, I'm working in a coronary care unit. So we have a cardiac ICU and high dependency beds. I work 3 rotating shifts.
- **NJCL: Could you elaborate on that?**
- P18: Okay? So what? My job scope is right on a daily basis? So I could be taking cases, patient care. So in charge, or I can be doing an assistant nurse role where I'm assisting in the routine ADLs for patients. Also, my ward has a telemetry- not say unit, but telemetry monitoring station of sorts. So there are some days where also I'll be sitting in front of a computer and monitoring patients, in other wards, and giving calls to their nurses whenever any irregular ECG rhythms are seen. And also my ICU, as with most ICUs, I believe, has a central monitoring station, can also be seated there on some shifts.
- **NJCL: Then how would you describe your workload?**
- P18: How would I describe it? I feel like, generally, it's stressful right? I think most- I mean, no matter what specialty you're in, working as a nurse in Singapore, I feel like a lot of my batch mates experience a high level of stress. But the question is, what kind of stress it is. From what I understand, general wards, in general wards, the source of stress, is like a very high patient- the nurse to patient ratio is not great. Like. For example, on a morning shift, if you are stretched, you can be taking up to 8 or 9 cases, and you might not have a lot of support from your from your assistant nurses, or ENs, if they are stretched as well. So, and especially if your patients are very heavy in terms of ADL requirement, like their ADL- if they need assistance from one or 2 people, they need to be turned, they have NG feeding. Then you actually need to also help out in a lot of these yourself. So you won't have that much time to do your staff nurse, RN responsibilities to juggle everything. So in intensive care. We're a bit lucky in the sense that you know we are not stretched as far, but patient acuity fluctuates very heavily in intensive care. So even though on paper you are like 1 is to 2, sometimes both your patients are super unstable, and they can code at any time. They can collapse at any time, and like you're carrying out a lot of changes. So it's really day to day basis. I think ICU has like some really good shifts when nothing happens, and then some really nasty shifts.
- **NJCL: Overall, can you say that you can manage?**
- P18: I would say that I'm surviving for now, and I think slowly, as I stay in this field I will- because when I started it was the worst. I felt like very stressed every day. But as time went on, I think I've been working there for more than a year now, it does, it does feel easier. So I think over time I'll be able to manage the stress better.
- **NJCL: Just to clarify, you have only been working in an ICU setting?**
- P18: I was deployed to a general ward for 2 months as a staff nurse. Right before I joined the ICU, I was in a general ward. So it was a COVID outbreak ward. Which also converted Into a general ward, general medicine ward after the COVID surge ended. So there was- so I felt surprisingly more stress there, but it was a different kind of stress. It was more the fact that that particular general ward, it was mostly taking overflow cases and overflow, meaning that, like, you are getting patients from disciplines all over. And you are trying to coordinate with a lot of different doctors for each patient. So I thought that I wasn't able to really dedicate my time into caring for one patient because I was like juggling everything. So it's a different kind of stress there. Yeah, in the general ward.
- **NJCL: Alright. Thank you for that. My next series of questions will be regarding lifestyle in general. So as a nurse, how would you define a healthy lifestyle?**
- P18: I think, having a healthy lifestyle as a nurse, it basically just means that you can make it into your fifties without like hypertension, diabetes or high cholesterol. Yeah, because I think if you can make it there. And you're like relatively healthy physically, it means you'd be like, well- of course, genetics has a big role to play, but so does lifestyle, and I think a good measure is like, how well you're doing when you're older. So it's what you do when you're younger. It's a big factor. Like I noticed that some of my patients who come in for heart attack in their forties. They have like this really high stress jobs like their managers, their lawyers. And then I just look at them. And I know, I know, like their life is really stressful. So I kinda wanna I wanna make sure that I don't go down that same road too much
- **NJCL: Earlier, you mentioned something about when you're younger. Like having habits when you're younger or something?**
- P18: Oh, yeah, I think that, anyway- I'm just saying that what everyone does in their twenties and like the early thirties will set up how their body reacts to like the lifestyle conditions when we move on to like aging forties and fifties. It's basically how much stress you're under. And then it goes back to exercise. It's basically exercise, diet, and stress. I feel these are the main factors that really decide. Well, you can't control that, right? Because you can't control your genetics. But so when I look at lifestyle, I always wanna look at how much of your micros and macronutrients you're eating, how much process food you’re eating, how much you're avoiding it. And then, when you look at stress management, it’s like what you're doing outside of work, and how important it is to like distance yourself from the pressure that you experience at work.
- **NJCL: Okay, since you mentioned exercise, diet and stress. Specifically on exercise, like physical activity, how do you feel about your current lifestyle? Feel like changing anything?**
- P18: Yeah, I started going to the gym 2 months ago, because when I started my job I wasn't exercising at all, and all the way until like July this year. And then that was the point where I felt like I was already- I settled down a bit more into my ward. I already passed probation. I don't need to- I didn't need to do as much like, spend as much time in my own, like outside of work, reading and studying. So I was like, you know what I should take more time and focus on self care, because, like, I mean. you are gonna burn out. And then it's bad for your patients as well. So you need to take care of yourself.
- **NJCL: What do you think is the main impetus that made you want to improve physical health?**
- P18: I felt sick a lot more this year. So last year, I wasn't sick then, I only, I only literally only took one day of MC like last year. It’s like 6 months, but I only took 1 day. And then this year I had like long stretches where I would be like, really sick. And then, of course, like, when you're on contact with patients. Things happen. You catch the flu, you catch COVID, and it's more expected. It's more common than like people realize. And then when you are down with COVID. That's like 4 or 5 days straight. You're out of work, and then your your body is like trying to take care, like trying to recover, that’s like 2 weeks of no gym, so it was like July, and I realize like I should probably- and then I have friends. I'm very lucky, I actually have friends who go to the gym, so I just ask them, ‘guys what gym to go you go to? Then, I sign up with them, and I go with them. So again, like social support, I think, is one of the important drivers to making lifestyle changes. I'm lucky to have it.
- **NJCL: Earlier, you mentioned that to take care of patients, you have to take care of yourself. Maybe, could you elaborate in this a bit?**
- P18: Okay. The thing is that if you're not really tiptop when you go to work. If you're not like focused, if you're tired, things will fall through the cracks, and when you're taking care of patients- you're responsible for their care plan, you're responsible- like, the thing is right, we actually- it's very difficult to be a nurse because you have to do- like holistic care really means you are looking at everything that the doctor is prescribing. You're looking at everything and all the allied health that they could be getting. And you have to think through every single thing, and wonder, is this correct? And like, especially when- okay, because house officers and medical officers rotate very frequently they might like miss out on certain things, so like very often the, the, new batch comes in like, I'm always highlighting, you know. I would suggest this, I would suggest this. You know, like suggest this change, suggest this medication, update them on the vitals, suggest, like, you know, maybe you can hold off- the blood pressure is low, we can hold off the anti-hypertensives, that kind of thing. And when you are busy and not focused, you miss these things, and then it's like to the detriment of your patient, right? And of course I know a lot of nurses also like feel guilt- they feel guilty when they miss out on these things for their patient. Like, I'm not doing the best care that I can. So I think it's important to- I mean, like in the plane, they say, right: put on your oxygen mask first before helping others next to you. So like. I think it's important for us to remember that also, we need to keep our oxygen mask on.
- **NJCL: Then, regarding diet specifically, how do you feel about your current diary habits? Do you feel like changing it?**
- P18: Okay, okay, it's not perfect. But I do try. Okay. So the thing is, I realized that after I started working. I started eating a lot more fast food. Because, like, you know, when you have a difficult shift, you go home. The, the thing that's on your- okay, on my mind personally is ‘oh, my gosh, I could go for like a burger right now, just wanna feel better’, right? And like it's like, it's like junk food like. And it's like they're like studies that show right? Like they literally market this food towards you and like it's like, feel good food. So. And when you're nurse you're always stressed, and when you’re always stressed, you always want to eat junk food. So the thing is like, I don't even like, I'm really bad, like I'm literally- I just had Mos burger for, like my wake up meal. So I'm really bad at regulating how much fast food that I'm, I'm- like’ I’m supposed to cut down my fast food intake, but otherwise, like I can get away with it, because, like I'm underweight.. But I know, like these chemicals are gonna add up. And one day I'm gonna take a blood test. If I can keep living like this, I'm gonna take a blood test. And it's like, even though my BMI is fine, I'm gonna have like high cholesterol or something like that. So I definitely need to cut down on fast food, which is the hardest dietary change. Other than that, I've been doing a good job hitting my macronutrients and micronutrients. I take a lot of protein. I take a lot of- like I tailored my supplements everyday- like the supplements I take every day, to make sure that I hit the gaps in my nutrition like in Singapore. It's very hard to hit like folid acid requirements- umm, what else? Magnesium. Because our Asian diets are very carb heavy and protein is expensive. So as a certain point is that, yeah, okay, I'll just take like some pills. It's not ideal, but it's better than nothing.
- **NJCL: Hmm! What would you say is your main impetus to changing your dietary habits?**
- P18: I think I've always- I've always had a very- I've always wanted to eat healthy even before I started nursing. But the thing is like, when I start this job, I eat worse because, like the urge to eat fast food increases. So I feel like my diet hasn't gotten better as I started working, it's gotten much worse because of stress. Yeah.
- **NJCL: Hmm. Then on this same idea, how do you feel about actually like providing dietary advice to patients when your dietary habits is a bit- as you say, not perfect?**
- P18: Yeah. So the thing is right, I can look at my patients, and I tell them you need to- you know. I can't tell them to their face like, you know you, you need to quit smok- Okay, I don't smoke, right but like if you tell someone that you need to quit smoking, it's not going to make them quit smoking. It's like I'll look at my what my patient- like I need to find a reason for them to quit smoking. If they have like a young kid, I'll look at them and I'll ask them, ‘you have kids at home right?’. And it's like, you know, like. I think it's important to take care of yourself, because I know, like my dad quit smoking, because they wanted to grow and see me grow up and like you give them a reason right? Usually it’s something like whatever, like family or kids, or like friends. Whatever lah, like so many things- well, not say so many, but like you, just go through and see what you can. You have to make it. You have to make them want to quit, and I think the same thing applies to any like lifestyle change. This- and like its the same thing for any habit changing as well. Just need to give someone a reason. You cannot force someone to do something. All you have to do is I have to show them why they want it, and then, like they will do it. [Participant is gesticulating strongly as he says these with a tone full of conviction before taking a huge gulp of his cup].
- **NJCL: Then do you believe, like, nurses are actually role models for patients in terms of like having a healthy lifestyle?**
- P18: I'm sorry. Could you repeat the question?
- **NJCL: Okay. Do you believe nurses are role models for like a healthier lifestyle?**
- P18: Yes, I think yes, it's very important for us to be role models, but I know for a fact that a lot of us are not. Not really- like, I don't want to blame ourselves, but it's really just because we are living like very stressful lives. And when you're like living a very stressful life it's like so hard to make this important changes because like you have no energy, takes willpower to like start the habits that would change. So I think, like, the thing is this, patients look up to us. So actually, we do- okay. In a sense like we have to put up that front, that image like: ‘oh, I'm living healthily. I'm- I don't smoke’. Well, most of my colleagues don't smoke so that's fine. But like ‘I'm living healthily, I’m not so stressed,’ you know? Like we put up this big smiley face in front of them, and we tell them we can do, it's possible, I believe, and like they have to buy it, we have to make sure they buy it, because, like, you know, it's our job. We care for them. We want them to be healthy. But behind, you know, behind the job, it's like, Oh, I'm living like shit also. Sorry! I'm living really badly. Also. So it's like, it's- it's kind of like that, I guess from my perspective.
- **NJCL: Does this give you any like unnecessary guilt, or like stress about not being able to maintain health in front of patients?**
- P18: Well, I, for me, I think I'm like fine, but I don't think it makes me guilty, if anything makes me able to relate to them more because I'm human, too. I have like addictions, habits, all these things as well. But I tell them like, you know, like you don't really like- it's possible, right? Because I'm struggling too. But I'm still making the best out of it. So I think, like it's possible for you to do the same thing. I don't think- I don't feel guilty at all. Actually, it's like, yeah, you just have to make it. You have to relate to your patients. You have to make them- like you're building this nurse-patient relationship right? And like, even if they are not- being imperfect, actually makes it easier for them to relate to you. That's how I feel.
- **NJCL: Okay. Okay? Then, coming back, what you mentioned earlier regarding stress. Specifically on stress management, how do you feel about your current stress management habits?**
- P18: I feel like I'm doing a much better job than I was when I first started. So when I first started working, like the focus is like just pass probation. Learn as much as I can. ICU turnover for new staff, is like, at least one in three. I heard, 1 in 2 in some intensive care units. So I really work so hard every day, go to work, learn new things, try not to make mistakes. Try not to get patient killed. I mean, of course, I have a someone following me, but like I don't want to do anything that put patient in danger, and then I go home, I have to read all this like, read even more pathophysiology because- I think in general ward is like a lot more straightforward because what you've learned in uni is really like, it's, it's the same thing. And when you go into intensive care, I realize, you really have to know, like almost as much as the doctors, because, like, you're so responsible for suggesting changes, and you have to know your patients head to toe. So there was that to get used to. And then after that, after I cleared probation, I was like, wanting to focus on de-stressing. So I picked up some new hobbies, I started working out. Meet up with like old friends, you know. Have like that social support, you know. So, I think it was mostly like that, that point that one year mark where I just stopped and think: ‘okay, well. I'm not gonna get fired. I just need to make sure I don't get my patients killed, and then, like I can spend more time focusing on making sure I don't burnout’.
- **NJCL: You've been mentioning like when you first started and stuff like that. How did lifestyle change after you started to work as a nurse?**
- P18: I stopped like engaging in a lot of hobbies. And I felt I had no energy to do anything. It’s like you have off days, but you don't feel like doing anything, you don’t feel like going out. You just wanna go home and sleep, and then you eat and then sleep again. You do your chores, and then you wake up, your off day is gone. So what I'm doing now is like, you know, I schedule things. I schedule like meeting friends, going out. Even later. I'm going to the gym. So it's like you have to have routine of sorts, and it's so hard to have a routine when you're working 3 shifts as well like the night shift comes in ruins your sleep. You spend so much time to fix your sleep, and then your day is just gone. So, it’s like you have to be very disciplined with like your schedule. You have to know how much sleep you need. You know how much sleep you're getting, know how much. And then, like you just don't get more than you need. But I think most people I see are actually sleep-deprived.. So like the advice to them, is actually need to schedule more time for sleep, and then you can do the things that you want to do. Or like, you do the things that you're interested in, if you don't want to do them at the time, because, like, at least, you are living a bit before you go for your next shift.
- **NJCL: Okay. I think, sort of answered bits and pieces here. But my next question: as a nurse, how do you feel about self-regulating a healthier lifestyle?**
- P18: How I feel? I feel like it’s the most important thing that we should be doing. But it's just so hard to do. So most people can like- I think the main reason lot of nurses- so like, my theses was actually on turnover, nurse turnover. And I feel like there's so many things that will really drive you crazy. And if you don't take the time to take care of yourself, you're definitely not gonna make it. You're gonna like, want to resign and like, if something happens, and they have like a mental health crisis. And then like, that's just the worst outcome. So I think it should be prioritized more. And I feel like the approach- like what the approaches that most hospitals are doing for their staff, I feel like it's not really enough. Because, yeah, like they send us for like resilience and everything. You learn the resilience, and it does help. But it's still a big problem, I think that there’s more that they can be doing.
- **NJCL: As in the hospital, the institution itself, has to be doing more for the nurses?**
- P18: Yeah, I think it’s the duty of management to take care of their workers. So I believe that- in Singapore you have this massive nurse resignation wave. So I think the first thing that they should be asking themselves is: have we been doing enough for our staff, right? So I think, of course, there's this responsibility on both sides right like you have to take good care of yourself and all that. But like, from my point of view- I mean, no one wants to be sick, no one wants to be stressed, right? When your patient falls sick, you can't just like blame them straight away, and I feel like it's the same logic going into whether or not nurses should be doing more to take care of themselves or whether it’s up to the organization, I think like it takes 2 hands to clap. And from what I've seen across the hospitals they could be doing- the hospitals can be doing- well, not just hospitals like any healthcare organization, they can definitely be doing more. But it could be just an issue of like Singapore workplace culture being very difficult in the first place, not just a healthcare thing.
- **NJCL: Hmm! I see. I think my next question, you also sort of answered here and there. But what do you think, are your main facilitators, slash motivators in self-regulating a healthier lifestyle?**
- P18: There's no hard and fast answer. Everyone has different motivations. Everyone has a different personality, but blanket wise, the main things that I can see, if I want to force someone to change the habits, right? You just find what's important to them. For some people it’s family, for some people it's friends, actually, for most people. So social support, I think, is the most important thing. Otherwise you have to look at what motivates them like what they, what kinds of- not everyone, for example, not everyone, is in nursing, because they want to be a nurse specifically. They are just going to work, because like they have to take a paycheck home. But like there's something in the job that they will enjoy, right? So it will be like working in the team, working in maybe a hectic environment. They find it fulfilling, and making a difference in someone else's life. Just need to take something and remind them that it's there and like, make it more appealing for them to work towards. That's all.
- **NJCL: Okay. Then, moving away from facilitators or motivators, what do you think are your main barriers and self regulating a healthier lifestyle?**
- P18: My main or like across for like nurses in general?
- **NJCL: For you, and if you want to share the across nurses as well…**
- P18: For me, I like feeling healthy. I don't like feeling tired. I don't like, like having the brain fog. When I'm awake, I always want to be like firing on all cylinders like being fit right and like- I feel like my mood is much better if I don't feel like brain fog, if I don't feel tired. So that's one motivator. I like feeling healthy. Another one is I genuinely enjoy taking care of people so like, that's a nice thing about this job. So, I still look forward to going to shift every day like no matter what. That's the thing that I like to try telling myself is like before I wake up, I go to work, and I know it's like the ward is full, it’s like all the ICU patients are sick, I'm going to have a really, really tiring shift. I just tell myself: it's okay. You just go to work, do your thing and like they're going to get better. I’m doing something meaningful. So those 2, I would say, are the biggest motivators for me, taking care of myself right now.
- **NJCL: Okay, then, what do you think is stopping you, or like a barrier for you to maintain or self-regulate a healthier lifestyle?**
- P18: Definitely the- for intensive care units right, the, the main stress comes from like the fluctuation in your workload. Not every shift is difficult, not every shift is easy, but because there's always this like your patients, are critical, even if they're stable, they're critical. Things can go down here any second of the shift. So every hour you're on shift is like, yeah, like just praying. Please let me make it to the next hour. Let me make it to the end of the shift with like nothing happening. No admission, please. Right? Oh, man I miss like, general ward admissions they were so easy. and in intensive care admission comes in, It's like the whole- you need a whole army to go into the room and settle the patient. Yeah. So it's like this. So what I'm trying to say is that having this like whiplash of stress. it can actually be more dangerous right? Because, like, then you feel stressed every second. You know, it's like, yeah, thinking every second overthinking, overthinking. So I would say that that is the main difficulty that I find trying to be not stressed. And the stress is the main thing that's causing me to not adhere to like these good lifestyle decisions. Just the stress.
- **NJCL: Hmm. okay. So I'll just briefly summarize what what you have shared with me. So in terms of facilitators and motivators for you, you mentioned social support. You also mentioned that you actually like to feel healthy. And there's also the idea of like finding meaningfulness or like purpose in your your day-to-day job. That actually motivates you-**
- P18: Yes, yes.
- **NJCL: Would you like to add anything here? Facilitators? Or motivators?**
- P18: Those are the main factors. Yeah, those are the main factors. I'm not really driven by monetary. I know, like, okay, I did my thesis- there’s a lot of other motivators like monetary incentives, career progressions. What else do I remember writing about? A good workplace environment, right? But yeah, okay, to be fair, I guess I can like, I'm very lucky to be in my ward, like the staff are very supportive, but like them, being supportive is like more like, it makes me less stressful- okay. I guess I can put this. It's more like a mediator, like, I'm not so stressed because I have very good colleagues, very supportive seniors.
- **NJCL: Then, just to clarify about the idea of like, this sense of purpose, the meaningfulness. So this motivates you to work harder? Like, how does this relate?**
- P18: Yeah, it motivates me to work harder. It motivates me to take better care of my patients, and motivates me to take a better care of myself as well. because I'm enjoying my job, right? So if I'm enjoying my job, and I want to do like the best that I can do in my job, it makes me- yeah, it's just basically my reason to really put in as much as I can in a way that's not tiring. So, in fact, like, in fact, I think that big thing is when I'm putting in effort into doing this, into improving right. It's not tiring. It's not stressful. Compared to if you're like studying for something you don't like, then, like, it’s a lot of hard work, like it drains your mental capacitiy.
- **NJCL: I see, okay, alright. Then, for barriers, you mentioned. It's mostly the stress, like the fluctuating stress. Like the difference in the patients. And because of this, you’re overthinking most of the time.. And then because of how stress you are, you are unable to actually make good lifestyle decisions. Is this correct?**
- P18: Yeah, correct. it just makes me have no energy to like pursue these kinds of like positive changes in life.
- **NJCL: Is there anything else you'd like to add for barriers other than being stressed?**
- P18: No, nothing else for me. Although I think for most a lot of people you might hear like they don't really see the importance or whatnot. Actually know, I feel like they won’t say it, but I feel like it's a big factor for others.
- **NJCL: Actually a common barrier some of the people are saying, okay maybe not barrier, because some people might say difference things. But, they say, shift work is a major factor. What are your thoughts on how shift work can facilitate or hinder self-regulation?**
- P18: Okay, that's an interesting point. So the thing is right. I managed to overcome it. It does affect me. And I feel like it does, it really does. But basically again, it goes back to the scheduling, you have to know- like, for example, I go to gym with my friends. I know that they go on like weekdays after office hours. So I know, like if I'm on day, morning shift or PM Shift, I can go. So like off day, right? So like I make it and point to myself like, as soon as my roster comes out I will literally start planning what days I'm going to the gym. So you have to be very disciplined in what you're doing to make these changes. So I know that for a lot of people, yeah, it's hard for for them. But I think I'm like quite used to it. I'm very lucky because like I used to do a lot of research on like sleep- my own personal time, not for me like professional pursuit. So I know how to cycle the sleep in the rostering. And I know that, like currently this 3 shift cycle that they're doing a lot of Singapore hospitals is really bad cause I know what they do in some other countries is that they'll do day shifts on one week, and then, like you work night shifts on one week. And it's really good because it lets you cycle like between the weeks. But what they do in Singapore is like one week, you’re like day shift, day shift, day shift, night shift, off, day shift, and then, like it's together. And you, you, like your, your off days are spent cycling in and out of day and night. So you have to be- it’s harder to do here, for sure. And also when you work shifts, you work weekends. It's harder to meet up with your friends. So again goes back to the idea of like, the days that you have like the weekends that you have off, you need to be the one to like take initiative, and meet with the friends who know you are free, and you need to find who else- you have to also like, keep a note what, what times your friends are free. So that's the thing. It does make it harder. I agree- but not for me. It does take mental energy to make this kind of dedication. So I think for a lot of people, it will be quite difficult.
- **NJCL: Okay, so you mentioned a lot on like having self discipline, right? But, can you think of any way to increase people's determination to change? Or like increase their self regulation for themselves?**
- P18: Well, yes, but like it's, it's again. It's like a case by case basis. So first of all, to convince people to make a lifestyle change. You have to make it a very simple change. A change that is easy to implement, a change that is easy for them to do it, right. So there are a lot of challenges because we're stuck in the habit, that’s the thing. I finished like a very tiring night like like, just now. I finished my night shift, I go home. It's like, I look at my phone and okay, this goes like, I look at my phone, and go ‘man, I can really go for some like Mcdonalds breakfast right now’ and like it took me a lot of effort to just like, you know, tell myself, no, just go home, make a sandwich. Go to sleep. You'll eat something healthy when you wake up. So we have to like nudge this people so like the reason I keep thinking about fast food, right? It's like, you see the ads everywhere you walk around you see a Mcdonalds on the street, and it's like you think about it. And then when you think about it, yeah, you want it. So it's like you can- like, there are multiple layers to like motivate people. So you take away the things that like they should not be doing. And then you replace it with things that they should be doing. So, of course, it's very hard to pull off, especially in Singapore. It's like you live in a city. There's like- you see everything, everywhere. So if you have like a bad habit, it’s very hard to distance yourself from it. Then, otherwise, I think I think it just goes back to- I mean, if you're thinking about researching for like interventions, right, then it would need to be something that's very specific to like a specific lifestyle change. And then you need to figure out like how to make it easy for these nurses to make that change. What are their motivations, or like what's causing them to keep engaging in like this behavior? What's causing them to like have poor sleep habits, or like- poor sleep hygiene, poor diet. What's causing them to not engage in exercise. And then just have to take those barriers away. Put in like their motivators. That’s all. That's just it.
- **NJCL: Okay? Yeah. Alright. Then, moving on to my next question, how does your self regulation habits impact your work performance?**
- P18: Oh, like, it's so straightforward because I feel so much better when I'm fit. I exercise, and I eat properly, and I sleep properly. I go to work. It's like- I'm like doing all my- like, you know. You just get everything that's faster because you have a routine. You're here. You just go and say ‘hi’ to your patient. You know how to talk to them properly, so that they won't pester you for the whole shift, and then, like, you know, they don't complain about you. And then you’re like, you just do all your shift assessments and like you're like having- like happy, they're happy. And then, like, when you're done. You go and help your colleagues also. And then, like, when you help your colleagues, they will help you in the future. So it's like you're just more productive and you get things done more efficiently. That's what really helps. You feel good because you're not stressed because you, you have so much work. Then, when you clear your workload faster, you’re less stressed. And then when you help your colleagues, they are happy. They will help you out. And then, when you finish your shift. You can go home without like worrying about anything.
- **NJCL: Okay.This is for work. But how about in your life? How do you feel self-regulation affects your life?**
- P18: Oh, I used to like compete right, like in video games. So I've always been a very. I've always been very driven to like take care, like self-care. Because I know, like you're not- you you can't like- it's so hard when you're competing at high levels. You cannot make mistakes, right? So you need to, you need to make sure that you have a good mental state. And you need to make sure that you are always like ready to make the right decision. And like all your competencies, all your skills are tip top. So in a way, it's like, it's like, I'm kind of engaging in the same behaviors I've always engaged in from like, you know, like, before, when I was in school before I graduated. It's just really this important- you have to understand the importance of like taking care of yourself so that you can really live like the best life that you can live. So once people understand that. And I think, like they are more willing to take those steps, because I know it's like difficult. A lot of lifestyle changes are very difficult. If it was easy, right, Mcdonald's would be out of business if it was so easy. But it's not so… It just starts from like that little, small baby steps. And then once people gain more confidence in themselves, they can like develop themselves to be more aware of what they need to do to improve like their well-being. And when you're like feeling good physically and mentally, you can do a lot of things that you could never do before when you're like feeling sick.
- **NJCL: You mentioned, like people need to understand the importance of taking care of yourself, and then we'll be able to make the change.Then do you feel that nurses in general understand the importance of taking care of themselves?**
- P18: Definitely! Because, like, they look at their patients, they're all sick. They're all like, really frail. They will know that like they're going down, they're going to be like them in like that in 20 to 30 years if they don't take good care of their health. So for sure they have some awareness at the back of their heads. But it's not a priority like the thing is like, if you are so stressed at work and like you're not always thinking about like- like you’re always thinking about work, in a way that you're worrying about, what can go wrong? Then you won’t really step back and have the mental energy anymore to make these lifestyle changes. So I guess my point is that they do know. But sometimes, when you're distracted by the busyness on the ground, then it becomes very hard to prioritize, and I think it's the same for a lot of things as well that are important.
- **NJCL: Okay? Can. Currently, are you using any like tools or apps to personally, self-regulate your lifestyle?**
- P18: Oh, that's an interesting question. Right now. Not any apps that are specifically tailored. At most I’ll use Notes, right. Notes and alarms. That's about it. It’s like- hmm, yeah, no, no specific apps.
- **NJCL: Then, for my next series of questions. It will be more on the workplace in general. So as a nurse, how do you think the workplace influences self-regulation habits?**
- P18: I think that working in this environment has given me a lot of self confidence in that I can go to work, I come out and it's like yo, if I can survive this job, I can do anything in life. So like, yeah, it's like- [Participant laughs here]. So I feel like- like, okay. Previously, when I was like on and off going to the gym, I realized I was- I had periods where I just stop going. And then, like, I just don't go anymore. Then I just have to remind myself now it's like- it's more important to remind myself like, why I'm doing it, and I don't, I don't like force myself to do things that I don't like, so I always find myself, I always give myself a reason to enjoy, like the lifestyle changes that I'm doing. It's like, Oh, if you go like, you should text your friend and ask them when they're going, and then you go with them. Or like, when I was like, sometimes when I don't feel like eating. I have to eat lot of food because I'm underweight. I'm trying to gain muscle mass, so it's easier to turn my patients and everything. So if I'm full or like, I can't- Oh, this was a big thing. Like after PM shift and night shift, I come back. I'm so tired I'm supposed to drink like protein shake before I go to sleep, so that I can maintain my caloric intake for the day, but some days I come back, and I’m so tired. I just want to shower and sleep and not eat anything, and I don’t care. So I had to change like, so I put my things like my supplements that’s more accessible. And I bought like a portable blender because I realized like I didn't like to shake my protein shake with my shaker bottle. I just throw out my ingredients into the blender and I just press it. So, I just made changes easier for myself. I have to do this because one thing I learned is that- actually right- this Youtube video I watched- what’s it called? Yeah, ‘Willpower is for losers’. Willpower is a muscle, right? If you force yourself to do something that you hate- like, I don't know. Like I feel like there's this misconception that people who are very successful. they have like this infinite source of willpower that they are like, you know, they're like grinding non-stop like NPCs or robots, or whatever. In reality. no, what you do is- yeah, you use your willpower to make like the changes that makes your life easier. That's what I've been doing. I think, like it's it's a good. It's a good habit, and I should do it more.
- **NJCL: Oh, I see. Yeah, it makes sense. Okay, then, maybe away from like physical facilitate self-regulation of healthier lifestyles?**
- P18: It literally all just depends on your workplace. I'm very lucky. My colleagues support me. They know that I'm stressed. They know it's stressful for new staff. so they give us advice. They don't judge us or scold us too much if we make mistakes, right. And the supervisors, your Sisters also, the same thing. It’s like- there's this balance right, they have to hit like, I know like- Sisters are always trying to promote you as fast as possible, like they give you so many responsibilities for that. Oh, you know you do these to promote you faster, it’s like career progression. But at the same time is like, they know, like this kind of thing will burnout your staff really fast. So it's again, it's like this balance, where, like, I think being able to communicate effectively with your colleagues, both your colleagues and your supervisors will really go a long way, because then they will understand like your difficulties. I don't think anyone- like people are like generally not out to sabotage each other, no matter how’s the situation, right? So if you give them like that, if like you give them- how do I say? If you're able to present your perspective, at least you give them the chance to evaluate it, and like, see where they can help you, especially as a new staff it’s very important. It's not- I know it's sometimes very easy to like- and it's true. Sometimes it's almost like they really bully the new staff, and like the boss doesn't care. They won't listen to your problem, it's true, but, like you just do what you can on your side, I know, like some wards are really tough. Some workplaces are really nasty. At the end of the day, you have to focus on the things that you can change and ignore the things that you can't change. Yeah.
- **NJCL: Are there in any instances in workplace where the workplace culture among nurses hinder your self-regulation of healthier lifestyles?**
- P18: I think it took a while for, like my bosses to understand that I wasn't like interested in like getting so many responsibilities so fast. So I just was trying to tell them. Like, Look, I'm like, fresh grad,. I'm completely new. I'm like literally trying not to drown, okay, I'm learning how to tread water, so I will take on more weights on my limbs when I'm more competent. But right now I'm trying not to drown. And I just want to help out where I can without like drowning. So once I presented my case like that, they were a lot more understanding, and like I was able to find, like the places the skill sets that I had to help my ward out specifically in things that they needed, because, like most of the time- you like- there are somethings that people don't like to do that you are good at doing so like you can fill that gap. And I realize that I had that for like some things in my ward. So I just offered to contribute there. I think this approach is has been quite mutually beneficial. So just again, just back to the idea of like trying to synergize with your workplace- sorry the question was like, What can you do, right?
- **NJCL: It was more on like how the workplace culture, the social aspect of your workplace, can-**
- P18: Oh, yeah, yeah, sorry. So I'm lucky, because at least they were willing to listen. And like the workplace culture in my ward is very like, things can be very ground up. Of course, one downside also, I realized, is sometimes the the supervisors are very resistant to change, even though you try to sell your points, sometimes they won't listen. So that's one thing. So that’s the negative is that some changes are very hard to suggest, even if you truly believe, and you present the evidence that, like it, would be beneficial for everyone if we implemented this change in this way. Whereas, on the other hand, the culture of being able to suggest smaller changes. Let’s say, if the bosses are not very keen on that something going either way, and you suggest that oh, we should do option B instead of option A. If they're okay with it, then they will listen. So that's like the good thing. So the moral of the story, I think supervisors, in terms of workplace, supervisors and seniors, giving new staff like the chance to at least voice out, and at least, if they disagree, give a valid reason- make the juniors also understand where the seniors are coming from. It’s this 2 way street, right? It's not like Senior say, oh, because I said so, that's why we can't do this. It’ll just make everyone, not just junior- so anyone who disagree with you, they don’t really want to engage with you, and then like it ruins the working relationship and the environment. This 2 way communication.
- **NJCL: Then, for you, What do you think about the role of your nurse managers in helping nurses self regulate their own health?**
- P18: What do I think about that role? Sorry.
- **NJCL: What do you think about the role of nurse managers in helping the nurses on the ground to self regulate their own health?**
- P18: I think that it's important for them to understand. First of all, shift work is extremely difficult. I know it's a necessity. But sometimes they're very rigid when it comes to suggestions. I think most wards in Singapore would benefit from switching to a weekly day, afternoon plus night shift cycle. It's like my friend, he literally did research and found out like it would be actually- it will alleviate the health effects, negative health effects of night shifting if we switch to like a week, day shift on one week, night shift on another week that kind of cycle. But no one wants to implement it. Because I guess, like, maybe manpower plus staffing plus inconvenience. But it's really important. It's something that's important for a lot of people. So that's one thing, they need to understand what's important for us. And they really need to do everything that they can to support us, especially if, like the cost is like not unreasonable. I mean from my perspective. Since a lot of other countries are doing it. I don't think it's unreasonable for us to trial. At least maybe a few wards could trial it, and if it's- if the staff are supportive, then they can go for it. There's a lot of things also that are like. There are like certain requests, like, I think some nurses actually want to switch to instead of 3 shifts like we're doing what like they do in Western countries: 12 hour shifts. So like 2 shifts only, and then you work less days. So like, again. I think a system like that has ups and downs. But at least management should understand. Like we must know, why we can’t go for this approach. And if we can't go for this approach, at least we know that at least they try, their listening, and it makes us feel supported, and when we feel supported, we don't feel as frustrated when we are working with them. That's what I feel.
- **NJCL: Currently, how do you think your institution can help you to self-regulate a healthier lifestyle?**
- P18: I think they can improve working conditions. So currently working conditions across many, many wards are very difficult, and I think because of this, is like sometimes they are bit too carefree in like changing certain policies, even though we are all like- a lot of the staff in my ward have been working for 5, 10 years. And whenever there's a new change that comes in, it feels like it's very like, for no reason kind of change. It just makes life- it makes management life more convenient, right? But it makes our life like hell on the ground, so they need to understand that any change that they make. It's like, it takes time and effort for staff on the ground to catch, learn. and apply. So if they gave us more support when implementing these changes, it would be really good. And also they should consider again, like they should understand that the ward’s really stressful for nurses. For example, a lot of my seniors right they have families and stuff, and I realize, like management can be very rigid with, like the type of shifts that they give. But like they don't consider, they don't consider like- okay, I'm going to give you a straight up example. My ward, right, has not enough staff doing permanent night shift. So I literally like I asked if I could do it. And like the reasoning that I got back was not because I was too new- I understand that like if I'm too new, I'm supposed to still work 3 shifts until I get a good grasp. But the reason that they give was that I had no valid reason to be doing permanent night, and apparently like in my ward, if you're doing perm night- like most of them, are like Malaysians, and like they travel across the causeway. And I'm like what? Your night shift is understaffed, you have someone jumping at you, who wants to do night to fix that problem and you're telling them that it's not possible. I mean. Yeah. So I feel like, like they need to understand like, there's some things that are like win win, and like they should just jump on the chance that they can get it. But I feel like, yeah, they just need to be a bit less rigid, I guess.
- **NJCL: Okay. Do you know of any workplace self promotion programs in place in your institution?**
- P18: Yeah? They run like the occasional, you know. Sometimes they will run like health events, like one off yoga, Zumba. All these weird things that I don't know what the turnout is for, because. I've never showed up to any of them. [Participant laughs here]. Other than that, they do- what else do they do? Yeah, it's at these one off sessions. And like we have a workplace gym, but like it's always crowded. So I don't go there. Yeah. Other than that. It's like, it's like, not much that I can think of.
- **NJCL: What? What do you think will increase the participation in this kind of events? What what do you think will make you want to go to these events?**
- P18: You know- Okay, I was gonna say- I don't think these kinds of events will be beneficial for me. But then I realized, like, wait, that's what most people are thinking. That's why they're not showing up! So they just have to show like- make this events more appealing. So maybe instead of like yoga, right, like Zumba, right? You can do like pop music because that's what most people listen to, and they'll have a lot of fun. And then, like maybe have something appealing. You know, it's always this like, oh, you show up, and then, like free food and like, even though the food is healthy, they'll show up because, like Singaporeans love free stuff, right? So it's like free food. What else? Just have this little more incentives. Singaporeans love like- we’re very- okay to be fair most of the nursing population in Singapore, they are not just local. But I think anyone in general, they like free things. In Singapore culture, they see something free, then they show up. And they'll queue up for so long for it. So I guess that's one possible way.
- **NJCL: I see. Okay. Then I'll just briefly summarize what you shared so far for the workplace. So regarding the question on like, How do you feel about the workplace culture among nurses, and helping to facilitate or hinder self-regulation of healthy lifestyles. So for you, you mentioned, like the positive, is your colleagues are very supportive of you. Also your supervisors. So there's a whole- you emphasised a lot on communicating and making sure everyone understands each other's perspectives so they can adjust accordingly to that. So you mentioned that having such a supportive workplace culture can help you to manage yourself better. Is that right for me to say?**
- P18: Yup. Absolutely.
- **NJCL: Okay. Then, for more of like barriers you mentioned, like sometimes they are a bit more rigid, and they're not very keen on like changing some things. So it's a bit difficult for you to adjust accordingly to their expectations.**
- P18: Yeah, yeah.
- **NJCL: Yeah. Then, you also mentioned like for the role of nurse managers in helping the ground nurses to self regulate you mentioned that they should be doing as much as they can to actually support the ground nurses.**
- P18: I really believe that management responsibility is like solely to the people on the ground, because it’s the people on the ground who would take care of your customers- clients, right? So yeah, I really believe that the responsibility is more on them.
- **NJCL: Sorry. So, when you say that the the responsibility is more on management. Are you saying that self-regulating of your own health is more- the management has a greater responsibility than the nurses themselves? Or is it a balance?**
- P18: Hmm! I think when you look at it from an outside perspective, like from an observer's perspective, yes, it's on both sides. It's both sides responsibility, but in reality, on the ground. When nurses right now are spending like all their time and effort to just take care of their patients and do the best at their job. They're not going to have a lot of time and effort left for themselves. So I believe that with all the effort that Singaporean nurses are putting into their work, I tell you, really a lot of us, we go all out, you know. Like we go above and beyond our job scope. So I really believe that because of this management is very, very like the onus is very much on them to figure out solutions to why nurses are burning out, why nurses are quitting. Because we've given so much of ourselves already in the job that we don't have much left for ourselves.
- **NJCL: I see. Hmm, hmm. Okay. Yeah. Then, moving on, you also mentioned about how, when management make makes changes there’s not much preparation for the ground to actually adjust to these changes like, it only improves the conditions for management. But the ground nurses are negatively affected. Is that right for me to say?**
- P18: I would say, not exactly negatively affected. Maybe for us, a net even. But the energy that it takes, the mental space that it takes to implement these changes, makes it kind of negative in a sense. Of course, like, if it's like a practice change where there’s a clinical and medical bene- like if there's like statistical evidence like we need to do this clinical change because he has more positive outcomes, of course, no argument in this. We would change! [Participant exclaims here]. There is no argument here. But I'm talking about like administrative processes. I'm talking about things like, oh, you know, hospital is switching to like another brand and so and so because supply chain issues. So we need to use a different like equipment. And then we have to learn how to use the equipment. And like they don't give us like more- They don't give us, because, like staffing ratios are so stretched. You know we have to like stay- not say stay back, but like you know, time on the shift is like used to like, learn these things, this, this like administrative, slash, logistical issues? I'm not talking about clinical at all like clinical things. It's a must, right? It's like, no doubt you just change it. And it's justified but I'm talking about like administrative and logistics processes.
- **NJCL: Okay, and then when I ask you on workplace health promotion programs. You mentioned the occasional health event. Healthy events like yoga, zumba, this kind of usual things. Then you mentioned that to make it maybe more appealing, it's to provide more incentives. Because everyone loves free stuff.**
- P18: Yep.
- **NJCL: Okay. Yeah. Then, lastly, when I asked you on, how do you think your institution can help you to self-regulate a healthier lifestyle, you mentioned that the- you mentioned something on the shift work, how they're very rigid. They they don't want to change the structure of shift work, but you believe that maybe if they at least provided a valid reason why they're not doing this, then it will at least make you feel supported and heard?**
- P18: Ya.
- **NJCL: Okay. Is there anything else you want to add at this juncture?**
- P18: I think that's all.
- **NJCL: Then. Okay, then, do you have anything else that you would like to share about that was not mentioned in the interview? Any further comments you want to share?**
- P18: No. I think that’s all.
- **NJCL: Then this marks the end of the interview. Thank you for your time and participation.**
- P18: Thank you.

-End of P18 Transcript-

# **Interview #19**

Participant and interview details
 Participant number: 19 (P19)
 Date of interview: 20 Oct 2023
 Interviewer Number: Navarro John Christopher Lambino (NJCL)
 Time IDI started: 1800
 Time IDI ended: 1900
 Observational notes: Participant seems extremely lethargic.

Section I: Interviewer’s summary
 The individual interview was conducted via Zoom. Initially, interview was arranged for 1430. However, P19 slept through her alarms after a tiring night shift. We eventually arranged for 1800 for the interview. The participant did not turn on her camera for this interview due to technical difficulties. For context, P19 is working in a geriatric ward, in a public community hospital. She often says overtime is a common occurrence, with many nurses only going home 2-3 hours after shift. She also mentions the heavy workload. Overall, P19 did not mention anything that was relatively new. For P19, her main motivators echo the other participants; for aesthetic reasons, and to grow old without compromised quality of life like her patients. Interestingly, she mentioned that there is a ‘self-sacrificial’ mindset prevalent in her ward. She also mentioned that her supervisors/nursing managers are out of touch with the ground, even though she believes they should be advocates for healthier work life balance. In conclusion, P19’s workplace culture seems slightly negative, with her citing reasons such as ‘high expectations’ for the very high workload and not being able to go home on time. Although, outside of the formal interview, she believes that her workplace is good as they encourage self-growth of the nurses. While the interview still yielded valuable input, I noted that the participant had lost her train of thought at a few instances. It is assumed that she was still fighting the fatigue after her night shift and the quality of her responses would be affected somewhat.

Section II: Transcript

- **NJCL: Okay. Good evening, miss. Firstly, I would like to thank you for taking the time to speak with me today. My name is Chris, and I'm a NUS Y4 nursing student.The aim of this interview is to get better understanding of the perspectives nurses have on self regulating their health behaviors, and how various factors may influence this. I want to find out what are your views, opinions and experiences on this and I would really like to encourage to speak what comes to mind, there are no right or wrong answers. May I have your permission to continue with this interview session?**
- P19: Yes.
- **NJCL: Okay, thank you so much. Then, let's start with some basic questions like, which ward are you working in right now?**
- P19: I'm working in a geriatric ward. Hmm, anything else?
- **NJCL: Could you maybe describe your work? What you have to do on a daily basis?**
- P19: Okay, so my workload typically ranges from between 8 to 13 patients, and they all have varying levels of acuity, some of them may require one man assist, some up to 3 man assist. Almost all of them are ADL dependent, so I can’t just leave them alone to ambulate by themselves. Most of the time, thy are requiring assistance to walk, either that or they are on diapers, and unable to self-care. They do require a lot of assistance for basic things such as showering, feeding, drinking water and just encouraging them for daily activities. What else, hmm? Apart from that, I do have to juggle this heavy caseload and take preceptees at the same time. Sometimes it can range from one to two preceptees at the same time. And there might not always be junior nurses around, like your ENs to help me so, I do have to oversee multiple things at the same time, while ensuring that all my patients get the best care that they can get.
- **NJCL: Would you say that your workload for you is manageable?**
- P19: It is quite heavy, because as compared to other hospitals, I do notice that my case load is almost double of them. And during some days it is really really difficult, and I almost always never go home on time, because it's either finishing up things- nursing procedures that I have to do, or documentation, or teaching the new nurses on what processes or just clearing the checklist with them. And it can be quite difficult, especially when there are a lot of expectations. And from not just our colleagues, but our supervisors and the patients as well. And I do find myself taking a lot of my own personal time just to fulfil these expectations. Other than that, what else? Hmm. Yeah, so there are a lot of projects given by RO, so like the NMs [NJCL: NM: Nursing Manager]. Or the NCs [NJCL: NC: Nursing Clinicians]. And they do try to incorporate it in our nursing, which is beneficial for us, but sometimes it eats into our time as well. So some of the things which I do feel quite redundant, we still end up having to do but it does not really value-ad to me, or nor do I, nor do I see it value-adding to patients as much. So a lot of redundant things which I feel eats into my time and takes away from a- yeah, the what's it called, me to completing my work on time and providing holistic care. Hmm.
- **NJCL: Then, would you say that doing overtime is typical in your workplace?**
- P19: Sorry?
- **NJCL: Would you say, like taking overtime, going beyond your shift hours, is a typical occurrence?**
- P19: Yes, it's a very common occurrence. Sometimes it can range from- it can go up to 2 hours. 2 to 3 hours past their shift just to finish whatever work that you have to do. And it's mostly things menial things like documentation or going through people's checklist. And yeah, I do find my colleagues staying back really late, especially if you’re taking 13 patients, and they are all very, very heavy, very ADL dependent or they have a lot of things going on. Just the other day, my friend, we were supposed to end our afternoon shift at 9pm, but she, she left work at 1am, just to finish everything that has to be done, and she's one of the exceptional people cause she does go above and beyond to make sure that her documentation is completely accurate, and all the nursing procedures that are supposed to be done is done. But I do find my colleagues sometimes they do like try and not take shortcuts, but try to finish as quickly as possible, but they do like end up over-staying past the time they’re supposed to end work, just to finish things as well.
- **NJCL: Let's see. Okay. then my next series of questions will be moving on to like lifestyle in general. So, for you, as a nurse, how would you define a healthy lifestyle?**
- P19: Having 3 meals a day, adequate sleep. Having time to rest and just relax during off days. And being able to just go out for exercise or leisurely activities. Being and calm and not stressed on a day to day basis. Hmm!
- **NJCL: You mentioned 3 meals, so specifically on dietary habits, how do you feel about your current lifestyle? You feel like changing anything?**
- P19: I admit my dietary lifestyle is really really bad, mainly because of the shift work, and also I don't really have time to eat sometimes. But recently I have. I have been making a change about like 2 months ago. But previously my main meals were breakfast and dinner, or breakfast and lunch. And the time between these meals can range up to like 12 hours so, for example, I do make it a point to eat breakfast, because I really cannot function without it. But my second meal of the day, it really depends whether I have time to go for break, or if I- yeah, if I have time to go for break- I usually eat just after I finish work. And by that time it's usually quite late. And it does not always meet the necessary nutritional requirements. I just get whatever is convenient for me on the way home. Hmm.
- **NJCL: You mentioned making a change like 2 months ago? Could you elaborate on that?**
- P19: Oh, okay. Because I am planning to get married…
- **NJCL: Oh, congrats!**
- P19: Thank you. My boyfriend saw that this lifestyle is not sustainable at all and I’m always falling sick very easily because of how I as living, so he did encourage me to take at least- If I’m not hungry, like small and frequent meals, and I do visit the gym more often, or at least do like light exercise and spend my off days more meaningfully, because on my off days usually I'm really tired, so I just spend it sleeping. But now I do like take up exercise, or like any other activities, or just spend time with friends and family. So it's a bit less taxing on my mental health because I'm actually interacting with other people instead of just shutting down. Hmm.
- **NJCL: Then for you, what would you say is your main impetus in actually changing your lifestyle?**
- P19: Mainly to look better, and also hopefully to fall sick less often because I do- because we are allocated like 14 days of MC? Per year? But I already consumed like 10 of it and most of the times the days where I fall sick. I do take really like quite some time to recover, so like 3 to 4 days to recover. And prior to this I have never fallen sick this often. So that was a big, a wake up call that I should at least get my health back together. Because yeah, falling sick- It's really the long term effects of it is really not good. I don't want to like grow old and have a bunch of different illnesses, just because I didn't take care of myself when I was younger.
- **NJCL: Then specifically on physical activity-wise, how do you feel about your current lifestyle? Do you feel like changing it?**
- P19: So far, with the changes I've made, I think it's quite okay. I go to the gym like 2 times per week, one with a PT and one on my own, and before shifts I do try my best to do like a bit of cardio, because when I'm working, I do talk quite a bit of steps lah. About 10 to 12 thousand. So just simple cardio, on the days, on days before work is sufficient, and the days where I go to the gym is, I think, quite okay. Because I'm not exactly super super active. So doing things like incrementally has been working for me. Hmm.
- **NJCL: Then, earlier, you also mentioned, like to have a healthy lifestyle, you mentioned something about- something on stress. Could you maybe elaborate on like, what kind of lifestyle habits actually tackle your stress levels?**
- P19: Oh, okay. So, with how I was living last time, like I do just relieve my stress by not even even tackling it all. I just sleep it off. So there's not really an outlet for the stress to let get out. But now- I cause. I have a lot of activities I can actually properly decompress instead of just not doing anything about it. And I mean, obviously, what's it called? Being more active does have a direct correlation to serotonin levels. So you know, I do feel that my mental health is slightly better. Could be placebo, but I feel it's like slightly better lah. And spending more time with people around me does help me to decompress better. Yeah.
- **NJCL: Can you maybe elaborate on. How, how did your lifestyle change after you started to work as a nurse?**
- P19: I immediately- I am a workaholic. I do prioritize work over my personal life. So prior to work, when I was still in school, I had allocated a lot of time for eating with friends, going out just to shop or just spending time with my family. But ever since I started work, I find that all my attention and energy has been just focused on work. I dropped all the hobbies I had and all my free time was just spent recovering from whatever I did at work. And I find myself- I mean, when you're still young and impressable, nurse you do- whenever like a senior, ask you to do something like, take out extra shifts you do feel pressured to say yes. So, during the first year of nursing I did that quite a bit, and it was at the expense of myself as well, and the time I had for myself, and for my family and friends. But as in the second year I learned how to not overstep these boundaries, but even then, also, there are quite a lot of expectations placed on you from your supervisors and colleagues as you progress along the years and the expectations required of you might almost always requires you to do go above and beyond. I do find myself taking work home. Be it small things like doing up like some report, or coming up with a teaching plan for my preceptees, or thinking of new ideas for projects or sharing for the ground nurses. And even now I still find myself not having energy or interest in the hobbies I used to have. Even meeting with friends is also quite difficult, because I mean we work shift. So I meet them like once every 3 to 4 months. That's on like a good- what’s that called? When I can actually schedule meetings with them. But most of the time can go up to 6 months. I don't see my friends for like 6 months. And personal time, at least for me, our annual leaves are balloted, so you have to fight against people to get leaves. And my leaves are quite far apart. So there is actually, like, I think, about 5 to 6 months gap before I get my next leave. So I'm continuously working for 6 months, so I get a short break and then I work again. Yeah. So even now, I feel like I'm still quite workaholic, and I don't really have time, or at least I don't allocate as much time as I should to like personal hobbies and interests. Or like having a life outside of work.
- **NJCL: What do you think will, at least personally for you, what do you think will make you want to prioritize your health more?**
- P19: What would make me want to prioritize my health more? Knowing that as a nurse, I am dispensable, and if I do resign, there are other people who would probably fill up the position. And work is not everything. If I don't find joy in this institution, I can just leave. And I can always find a new job. But my health I can't undo like whatever harm has been done. And, yeah, I do see, like a lot of my colleagues, because we don't really go to the toilet as often, and we don't really drink water as often we do. There are quite a few of them who are at risk, or have developed like UTIs or dehydration, during a shift. So I, I really don't want that to happen to me. And I don't want to worry like my partner and my family, if I anything bad happens to me. So I better make this change while I'm still physically able to. And while I'm still like, generally quite a young nurse, and I don't have like huge huge responsibilities as compared to like if I'm a SSN or something
- **NJCL: I see. Yeah, then my next question is going to be on what are your main facilitators for motivators and self regulating a healthier lifestyle?**
- P19: My partner. Because he- he does- I mean, he's been there for me like for the past few years, and he has seen how work has had a really negative impact on my mental and physical health. So he does make it a point to just call me out on it when things are getting really bad, like really out of hand. And I don't really see it initially, because you are- I'm just really deep in work. But when he points it out that this lifestyle is really unhealthy, and he realizes that I am getting very impatient and short tempered. I for myself, find that I'm really getting very irritated quickly. And angry as fast-, I never used to be like this. So seeing this change in my personality as well, really, what's that called? It's a big difference as compared to who I was, when I was in school. And him, pointing this out, and how it could affect my relationship with my friends and family. If I'm always like really short tempered, has made me worry a bit, because I don't want to lose friends and family just because of stressors at work. And I end up lashing on them. Hmm!
- **NJCL: So, just clarify a bit. Other than your partner, your main motivator/facilitator is your partner.because your partner actually like points out instances where you’re too absorbed in your work.. Is that correct? Is there anything else you would like to add here?**
- P19: Maybe. My- it's mostly my family, cause I do like vent to them quite often, and they see me getting very frustrated with the working environment. And what happens with my seniors- and cause my mom is also a nurse. So she does tell me that this is not okay, and you shouldn't be tolerating this, and you should make a change, because if you were to continue like this you would end up hating nursing and you would leave. And my worry- I, I really like nursing, so I don't want to leave, and I don't want whatever experience I'm having right now to kill the passion I have for nursing. So I just, I am. I'm trying to take her advice.
- **NJCL: Okay, then moving away from facilitators and motivators, what do you think are your main barriers in self-regulating a healthier lifestyle?**
- P19: Maybe, time and energy. Because my shifts are quite long and can range from like 8 to 12 hours, and I do live quite a distance away from the hospital I am working at. So it's like a 40 minutes to 1 hour commute to and fro. By the time I reach home it's really quite late, and I don't really have the energy to do anything else. I am thankful that my parents do cook at home, but even then, I don't really take a regular meals as often. Breakfast is usually whatever I buy at the hospital, which is either toast or bread, or whatever 7/11 has- coffee, so I don't really drink as much water as I should. And the time that I have in a day I don't really have- I don't really find time to cook, which I used to like before, and I was able to at least make a balanced meal. But now is just whatever's convenient for me, or whatever my parent buys, or if they have the energy then they'll cook. I'll just eat that. I don't really care about what I’m eating, because it's not really for enjoyment, just for sustenance. As long as I have the energy to like just do whatever I have to do. Then that's enough for me, I don’t really count or see what I'm eating.
- **NJCL: Just to briefly summarize what you've shared with me. So for the question, on what are your main facilitators, motivators, or barriers in self-regulating a healthier lifestyle. For you, your main facilitators are your partner and your family. Then for barriers it's mainly your- you mentioned the the time, like having long commutes, shifts are quite long, and also the energy or lack of energy, like the fatigue after a long shift. Is it correct?**
- P19: Hmm, correct.
- **NJCL: Is there anything else you like to add for either one of these facilitators or barriers?**
- P19: At the moment, no.
- **NJCL: Okay. Then just moving back a bit regarding like how your, how your lifestyle changed after you started to work as a nurse. Just clarify a bit, is it correct for me to say, now that you are, I guess more used to your routine, has your mental health gradually improved?**
- P19: Sorry?
- **NJCL: Regarding the question on how your lifestyle change after you started work as a nurse. Is it right for me to say that, your mental health currently now is better than when you first started out?**
- P19: Yes, as of like. I think about 2 months ago, it has not been declining.
- **NJCL: You say this is because of like-**
- P19: Because of the changes I made to my lifestyle and being called out by my family and partner.
- **NJCL: Okay.**
- P19: And also my wedding. I think, that’s also another motivator. Because it’s [Participant nervously laughing], it's next year. So I have to make a change now.
- **NJCL: I see, congrats.**
- P19: Thank you.
- **NJCL: [Long pause]. Then my next question is, how does your self-regulation habits impact your performance?**
- P19: Self-regulation habits impact my work performance? Like previously?
- **NJCL: After all the changes, that you’ve mentioned?**
- P19: Before all the changes, my concentration was affected quite a bit because my mind is everywhere, and it's I found it very difficult to focus. Yeah, because of like either insufficient sleep or I haven't eaten enough or haven't drink or had enough water. Or basically fulfilled my self-needs. So my concentration was affected, and I- not errors but I did find myself not performing as optimally as I should. I did find myself missing out on things, or struggling to meet deadlines because I just didn't have the motivation to like finish whatever I had to finish. Like this out of work, it’s not like case load things, but it's external projects that the ward has or like my ward sisters provide. Yeah, it's a lot of- sorry what was your question again?
- **NJCL: How does your personal regulation habits impact your work performance?**
- P19: Yeah, yeah, it's the concentration, concentration and just how my train of thought isn't as clear. Like, I do find- I don't judge- I do have confidence in my own nursing judgement, but I find myself like, do taking quite a bit of time to rationalize certain things like why things are done a certain way, and it takes me a while to process. I feel like I've become a bit slower in terms of thinking. Yeah. And a lot of fatigue basically, cause it's just the same thing over and over and every day. Yeah, I've become. I've become a bit jaded with the ward because of that.
- **NJCL: Then, outside of work, how do you think your self-regulation habits influence or affect your life?**
- P19: Hmm! Low motivation to do most things. So I don't really, I find myself like slacking a bit on either chores or just general- not tidiness, but how to say- I used to be really very neat, but now, because I don't have the motivations like, you know, the depression pit. Like things start to be a bit more messy as compared to usual. It's still manageable, but I do find it getting- like I never used to be messy, but until now I do see that a lot of my things are quite haphazard. And yeah, my mood is also affected. But like I mentioned earlier, because I do get short-tempered quite easily, so I do find myself lashing out or jumping like lashing out most of the time. On like, towards people. Out of the blue, unwarranted frustration. And yeah, it’s mostly that.
- **NJCL: Just to clarify this lashing out towards people is mainly due to stress?**
- P19: Ah, yes. I think it's also a habit I picked up from work, because I do notice my supervisors tend to jump to conclusions and find fault instead of like reflecting on, why did this happen? How could it be avoided? So it's a habit that I picked up from, I think things I saw at work which is really not good. Okay.
- **NJCL: Then, after being called out on like lashing out on people by your partner. Have you tried to like make changes to improve your mood?**
- P19: Oh, yes, I think it's a bit more like slightly better control? I do- I take a few seconds to cool down, at least reflect. Is this or that? Am I angry at the person, or am I angry at something else? And there are times where I do still get quite frustrated at things. But it's not as nearly as bad as last time. And I do feel slightly less tense as compared to last time. Because ah, every time I go to work I do find myself- I do feel very, very anxious and worried about, ‘oh, am I going to finish my work on time? Or am I going to- like is there any issues that going to happen at work that I'm not going to be able to solve?’ But now, it's not as bad
- **NJCL: Hmm. Are there any tools or apps that you are personally using to maybe self regulate your lifestyle?**
- P19: Yeah, I do use myfitness panel to check like, see whether I do fit my dietary requirements and I do use a mood tracking app to note down whatever I've been feeling and the causes of it as well, like the stresses that lead up to me, getting angry or frustrated.
- **NJCL: I see. Okay. Then my next series of questions will involve the workplace in general. So as a nurse, how do you think the workplace influences, your own self-regulation habits?**
- P19: I think the working environment is really important. Because I feel like, at least for me. Whenever I work with my colleagues, I do tend to mirror whatever they're coping mechanisms are, and things because most of them are my seniors, so I do look up to them a lot. And because most of us don't really go for break, because we don't have time to finish our work. Mainly because we don't want the next shift to have, like so many things to do. And it's just like a never ending cycle of work just being passed down. So we always sacrifice our break time just to make sure that our friends don't suffer, and we try to finish as much as possible. Even like things, simple things like drinking water, like going to the toilet, we do forget to go. And we don't really remind each other to go, as well. So most of our basic needs are not met. It's only until like, I think recently, after my friend got a UTI and she got admitted because of dehydration only then, like, at least me and my friend, we remind each other like, have you gone to the toilet? Have you drank water? Have you at least eaten a bit during your break time? Most of my colleagues still don't do this. And they eat when they have the time, but at least for me, and, like one of my really good friends in the ward. We are trying to just remind each other to do like these basic things.
- **NJCL: [Long pause]. Okay. Then, going into the social aspect of the workplace. How do you feel about the workplace culture among nurses in helping to a facilitate the self-regulation of healthier lifestyles?**
- P19: I feel that the culture is not really there. Because yeah, I mean, our supervisors main focus are mostly like KPIs. So they they do forget that we’re human too and it's not really a habit that is inculcated, at least for my workplace. It's almost the norm that both of us don't really go for a break especially during morning and afternoon shift. If we have the luxury to go for break means like it's not a busy shift, right? But if we don't, then it's like just regular shift. And nothing has really been done to reinforce the importance of going for your break. Because a lot of people have the mentality whereby if you spend 1 hour on your break, then you are just going to stay back another hour just to finish whatever you haven’t done. So they do forego these small things. And I do see my colleagues falling sick quite often. And yeah, it's really just, very, very tiring for everyone, because we don't even meet our basic needs. But then we are falling sick just because of that. Hmm.
- **NJCL: Then, specifically on like nurse managers. What do you think about the role of nurse managers in helping to- in helping nurses to self regulate their own health?**
- P19: I do believe that our nurse manages should be the advocates of ensuring that we all have a healthy work-life balance, and that our physical or mental health is taken care of, and we don't do things beyond our like physical capabilities. But I do find that, because the last time they take case loads, was probably like what, 5 or 10 years ago? So they don't- they’re clearly out of touch with what it's like on the ground. I do know that they probably have gone through the same thing, but then- because it it may affe- the caseload may differ, because not all of them are in the- used to work in the ward that we are at. So they might have been from other disciplines which aren't as stressful or like as heavy and they try to impose their experiences or like whatever they experienced last time on whatever we had, which is not always applicable. Because the- yeah, everything's different. It's basically a different environment. So when they do try to make- not changes- when they do try to improve workplace morale, it doesn't always work because of how out of touch they are. Yeah.
- **NJCL: Okay. Alright. Then I'll just briefly summarize what you've been sharing so far. So for you, you believe that nurse managers actually play a role in like being advocates for healthier work, life balance right? Making sure the physical, mental health of the nurses on the ground are actually taken care of. But you feel that the nurse managers, at least in your workplace, are out of touch. So they can't really relate. Like the measures that they are implementing are not very effective cause of how out of touch they are.**
- P19: Ah yes, correct.
- **NJCL: Okay, then. Moving on, my other question on: how do you feel about the workplace culture among this is in helping to facilitate self regulation of healthier lifestyles. You mentioned that the workdays culture is not really there, because, you mentioned, like your supervisors, main focus is KPIs. There's not really a habit that is inculcated amongst the nurses to like make sure they go, for, like the basic needs, such as like going for break, drinking water.**
- P19: Actually there are a lot of barriers preventing us from doing that because my nursing supervisor, actually, she herself doesn't go home on time. She always goes home really late. And for reasons like infection control, we are not allowed to put like our bottles near our nursing stations, so course, it's not easily accessible. We don't really actively go and look for bottles of spring water, and we only have like- and the toilet is quite a distance away from the patient's cubicles, or even then we forget that we need to pass urine. And stuff.
- **NJCL: Other than the like physical environment, how do you think this current workplace culture in your workplace even developed?**
- P19: Sorry?
- **NJCL: Earlier, you mentioned like you, there's physical barriers such as like your water bottle not being accessible even,the toilet being far. So what do you think like made the workplace culture how it is today?**
- P19: Mainly because of like the SOPs in place. Like things like infection control. Or keeping things neat and tidy. These are all necessary day to day things but it just inconveniences us a lot. Even then also, they should at least like provide an area for us to put our like, for example, water bottles, so we can have adequate time to like drink. But a lot of the times it’s just how healthcare is-making sure things are neat and tidy, they're not cluttering things to, uh. Yeah. Oh, sorry if I'm not making sense. I'm like zoning out a bit.
- **NJCL: It’s okay, it’s okay.**
- P19: But you get what I mean?
- **NJCL: Yeah, yeah, I understand. So for you, how can the workplace culture improve so that it's better for self-regulation?**
- P19: Hmm! I do feel that the root problem is they're not staff. So the staff nurses have to end up tanking everything and most of the time the work that we do, you don't need to be a nurse to know how to do it. Like, you don't need to be a nurse, to bring patients to the toilet to change the diaper, to feed the patients. And, you don't need to be the nurse to become like the messenger pigeon for things like what the PT or the OT has to say for the doctor. They can do it themselves. So a lot of these small small things. Like maybe, if there was actually a BCA [NJCL: BCA: Basic Care Assistant], or like a healthcare system that can help to shower patients, feed patients. Then I really wouldn’t- maybe I have time to go for a break, because I don't have to fill and complete do all the remaining ADLs. And if there are more stuff to like, take up the heavy case- or at least when I’m juggling a preceptee. Then maybe I have time to like, finish my work early and go through the checklist with them. So I do feel that having more staff would actually be very beneficial because then I can actually do everything on time and finish on time while still having, like energy left over to fulfil, like whatever other things that my supervisor wants me to do.
- **NJCL: Okay, then. Other than elevating the manpower issues, how do you think your institution can help you to self-regulate a healthier young lifestyle?**
- P19: Hmm! They have, at least for my institution, they have been trialling this staff bento. So instead of just- because I do see that most of my colleagues just eat whatever is at 7-11, because it's the only thing open. I work at [Public Hospital name] so there’s like no food options. It’s literally just 7-11 after 7pm. And they have been trying to provide staff bentos. Which in a way does- because we have to collect the bentos before a certain timing when it expires. So it does remind us that all we have to go for a break and eat before this food expires. They are doing small small things like that. They do have enrichment activities after office hours which are free, like pilates and stuff. But yeah, the sign ups are usually quite okay. But it does mean that you have to stay extra time at work, and a lot of people just want to get off work once they finish. So I do see, like small small things that they are trying to do to help to cater to our: basic needs and just help us relieve stress.
- **NJCL: Have you personally went for any of this enrichment activities?**
- P19: I have gone for terrarium making? Once. But sign ups for these are usually by first come first serve basis so sometimes it's full like, really quickly, and there’s not a lot of slots. So It depends on your luck as well. I do find that it helps. And it's a change from what I do at work, lah. I do get to interact with my colleagues in out of work environment. So I do get to see like a different part of them.
- **NJCL: Then, with ll this in mind, like the bento for the staff, enrichment activities. Would you say your institution is doing enough to actually help the nurses on the ground to self regulate their lifestyle?**
- P19: I really am not sure. But according to some workplace satisfaction survey, [Public hospital cluster name] which is the group my institution is in, apparently has really good employee satisfaction. So I’m guessing it works, in a way? I personally don't see a difference.
- **NJCL: If you could make like a suggestion for your institution. What would you suggest that it's effective to help the nurses on the ground?**
- P19: It would be, I think, reinforcing like OT. I would say like- it would be good if you have like punch cards. Staying beyond a certain hour means that you are- you should be entitled to overtime pay so at least it does. Maybe like if they see that so many people are over staying, like staying at work far too late. Maybe they'll see that all, maybe the workload is a bit too much. Then our supervisors will be pressured to not give us such a heavy case load or extra work outside of work or extra responsibilities that consumes like our time outside of work hours. I mean, people can abuse it lah, by just staying back late. But I think for the most, most of them don't really stay back without reason lah. And nowadays I mean, almost always, they do blame like us staying by late, because of poor time management. But I think they need to see that it's not because of poor time management, but because of the really really heavy case load that we cannot finish everything during our shift. But there's always the expectation that if you cannot finish, then you can just hand over to the next shift but like, out of basic courtesy, I don't think it's very nice as well. So whatever we can do during our shift, we should at least do. At least that is what my seniors have taught us, because karma lah. Like you shouldn't always push work to other people because they might end up bringing it to you, so give and take. Hmm!
- **NJCL: So so like your seniors, have made this kind of culture where the nurse feel bad in like passing the, passing the workload to the next shift.**
- P19: Yeah, as much as possible, like- because you also understand that staffing is not always great. So we do try to finish as much as possible during our shift. Like certain things, that are not- at least the time sensitive ones. The not time sensitive ones, at least they can just- the next shift can just do it. But a bulk load of it, should at least- we do try to finish it, lah. So we don't burden our friends too much during the shift, lah. Because we are already staying back so late, I don’t want my colleagues to stay back late as well. There’s the self sacrificial mindset. Yeah.
- **NJCL: Then, is it right for me to say, this self sacrificial mindset was generated by your seniors?**
- P19: I'm not sure where it’s generated from, but when I arrived at work, it was always there. Yeah. I think it depends on the rapport you have with your colleagues as well. If you don't like the colleague, I do notice that they just hand over everything to the next shift. But if they are like a good friend of yours, or at least a colleague that you don't mind working with. We do try not to overburden them and stuff.
- **NJCL: Okay. Then, final question, as far as you know, are there any workplace health promotion programs in place in your institution?**
- P19: Health, promotion. They do have this staff checkup, I think, if you are like 40 and above or like 30 and above, you get screened for like cervical or colorectal cancer. Something like that. And we do have free flu vaccines. The staff clinic does provide like free health check ups. Sometimes they do talks on health promotion. But as far as I know, not many people attend. Then did we try to bring in vendors who sell like organic. So cereal, or like fresh fruits, they do come once in a while just to encourage staff to buy like fruits Or like- what's it called? Healthy snacks to consume while they're working. I think that's about it. I can't really think of much health promotion that they do. Hmm.
- **NJCL: Then overall, would you say these are effective or ineffective?**
- P19: I mean, the health screening is effective because it's free. But I really don't know how many people sign up for it. I, personally do not sign up for it, because, there is quite significant waiting time and I find that it's a bit faster if I go to the polyclinic myself to get a health checkup. The booths that sell fruits and cereal or healthy snacks, these things I find like, that the public is mostly the one utilizing all these things and not really the staff. And all of these I feel like a very passive attempts, because it's just there. If you want it, then you just go for it. But if you don't want, then no one's going to force you to do it as well. Even the talks are also very passive, and it's also marketed very passively, like through emails. And I'm sure a lot of my colleagues also don't really read every single email they get because there is a lot of advertisements or sharings or talks. Yeah. And most of them do prioritize learning over these like health wellness talks. Yeah.
- **NJCL: Okay? And then I'll just briefly summarize what you've shared so far. So regarding how do you think your institution can help you to self regulate a healthier lifestyle. You mentioned the bentos for the staff, the enrichment activities and as far as you know or like, according to the employee, satisfaction survey, it's effective. It’s working. But personally for you, you don't really see a difference. Then you also recommended a suggestion such as like actually keeping track of like the amount of overtime that's being done which can hopefully, like potentially cause your supervisors to be pressured to decrease the workload because through these statistics, they'll find out that, hey? Maybe this too much work for the nurses. Yeah. Then you also mentioned on the self sacrificial mindset that you mentioned has always been there, that when you arrive it was already there. And IIguess you adopted such a mindset.**
- P19: Yeah, correct.
- **NJCL: Okay, then, regarding health promotion for you, there’s staff checkups, there’s free flu vaccines, there’s talks on health promotion, and then there’s vendors. You feel that this is mostly utilized by the public instead of the target population, which is the staff. And it's very- It's a very passive kind of advertising. And like measures. Is there anything else you would like to add here?**
- P19: Hmm! Oh, actually, one more thing. I think the institution should also reinforce, like not forcing us to take leaves. Because I think this is quite illegal, but at least for my supervisor, she does force us to clear like as many leaves as you can quickly. So we are entitled to family leave which is like 5 days a year. I think 3 or 5 days, I think. I mean, these leaves cannot be carried forward, so she forces us to take them early in the year. So actually, if there's any family emergency, I cannot take any family leaves because it's all utilized by her. And being forced to take AL- and if they see like, for example, that there are too many staff. I think they have their own like- what's it called? Algorithm that says, how many like number of minimum staff that should be working per shift. If it exceeds it, they do try to like force people to take leaves unwarrantedly. So this actually eats like into the balance that people have so they can't really take like certain leaves if anything happens with like their family and stuff. Yeah. So being forced to like, stay back on days where- not stay back like being forced to like, finish our work on time and being forced to like clear leaves. I feel like there is- my time is not respected. Hmm.
- **NJCL: Okay. Understand. Then, do you have anything else that you would like to speak about that was not mentioned in the interview? Any further comments you want to share?**
- P19: No.
- **NJCL: Okay. Then this marks the end of the interview. Thank you for your time and participation.**

P19: Thank you very much! Hope your paper goes well and you find more participants.

**NJCL: Thank you!**

-End of P19 Transcript-

# **Interview #20**

Participant and interview details
 Participant number: 20 (P20)
 Date of interview: 20 Oct 2023
 Interviewer Number: Navarro John Christopher Lambino (NJCL)
 Time IDI started: 2000
 Time IDI ended: 2045
 Observational notes: Participant seems ready and in a pleasant mood.

Section I: Interviewer’s summary
 The individual interview was conducted via Zoom. Interview was arranged for 1800, but was asked to delay until 2000. For context, P20 has 2 years in general ward, and 2 years in ICU in a public hospital. He is also doing a part-time degree. Using his experience in general ward and ICU, he was able to answer the questions using these experiences as a reference for a point of comparison. P20 did not mention any new data. Throughout the interview, he needed a fair bit of prompting. While his answers were relevant, they were relatively shorter than previous participants and his answers needed some interpretation and thinking before linking back to the question. Perhaps, due to P19’s interview being done right before this interview, there was seemingly a mental fatigue. Perhaps, I should have asked more prompting questions at various points to allow P20 to substantiate his points more. Interestingly enough, he mentioned that he felt guilty if he does not practice what he preach. Subsequent interviews will ask about nurses as role models for health and whether they feel this guilt of not doing what they preach to their patients in regards to health promotion. P20 responses has heavy emphasis on self-discipline and having a work-life balance. P20’s work culture also seems rather positive, citing it as a positive influence on his lifestyle. He also believes that management’s health promotion efforts can be improved, but he is relatively fine with his current lifestyle and how his institution is handling the matter of self-regulating healthier lifestyles. All in all, this interview may lack in depth, but the breadth of ideas that P20 presented are also echoed by other participants. Notably, I had an assumption beforehand that P20 was also a NUS nursing alumni like many of my previous interviewees. He was not, and actually came from polytechnic straight into the workforce.

Section II: Transcript

- **NJCL: Okay. Firstly, good evening, sir. I would like to thank you for taking the time to speak with me today. My name is Chris and I'm a NUS Y4 nursing student. The aim of this interview is to get a better understanding on the perspectives nurses have on self regulating their own health behavior, and how various factors may influence this. I want to find out what are your views, opinions, and experiences on this. I would really like to encourage you to speak what comes to mind. There are no right or wrong answers. May I have your permission to continue with this interview session?**
- P20: Yes. You have.
- **NJCL: Okay? And then let's start you some basic questions like, which ward are you working in right now?**
- P20: Currently, I’m in a multidisciplinary ICU. In [Public hospital name].
- **NJCL: Could you tell me more about like what kind of work we have to do on a daily basis?**
- P20: Hmm! So usually, if possible, if possible, they will assign you one to one patient care ratio. But it depends on the how, how the ward situation is. If you are busy, you usually take up to 2. Yeah. So when you came to the world, you have to check all your pre-sign checklist, you have to check your e-trolleys, check some PAPR, some certain equipment have to check. Then after that we will proceed go for roll call, then after that see what kind of patient assigned. So it depends whether, if you are assigned a patient with an empty bed, or you're have no patient, or you have 1 patient. So just depends whether you have admission or not.
- **NJCL: Then for you, how would you describe the workload?**
- P20: Hmm, at times can be very chill, because it's like a emergency department. There are times where it’s very quiet, and there times where it’s very, very busy, and it's very different from the ward, because in the ward you kinda, you- you know when, you kind of know what the ward routine is. But in ICU each patient is different. They have, they have that different kind of from the nursing management that you have to go through. Hmm! It's quite intensive compared to the general ward. The stress level is different. It's more like a life and death stress rather than cannot discharge, cannot charge on time. Those kind of service quality type of stress from the general ward.
- **NJCL: Then, just to clarify a bit. When you graduated and started working, did you go to the ICU straight away?**
- P20: I started in the general ward for 2 years in cardio ward. 1 year and 10 months or 11 months, so about 2 years. Then after that, they were asking who should go for advanced diploma. I was interested in critical care in advanced diploma that’s why I was transferred to ICU and I was there since, about 2 years? So probably 2 years in ICU now, and 2 years in general ward. So total 4 years.
- **NJCL: Okay, alright, I see, and thank you for that. Then my next series of questions will be regarding like healthy lifestyle, like lifestyles in general. So for you, as a nurse, how would you define a healthy lifestyle?**
- P20: I will say that, I have a balance of my work life and my social life, have a balance. So- definition of the work-life is it?
- **NJCL: Yeah. Could you elaborate more on what you mean by the balance work life-social life?**
- P20: I'll say that I am able to juggle between both. I can, I can switch, switch to go to like my social life as much as possible without being affected by my work, working life. Physically and mentally, I will say, hmm.
- **NJCL: Then, specifically on diet like, How do you feel about your current dietary habits? Do you feel like changing it?**
- P20: Hmm, yeah. Actually, to be honest, my diet has been changed quite, quite drastically for the past few years, because when I first started I was quite overweight when I first started. So so when I went to the general ward, you can’t go for break, and you would always end late. So you, every day, almost every single day I have to eat like only one, can be like one meal per day, because you don't go for break. Then same for morning shift, you end about 4 or 5 o’clock, then that's your only time. You have your, your meal. So usually it's one meal per day. Then subsequently you get more senior. You get kind of get familiarised, familiarised with the workflow, you get to go for some occasional break. Then I get to transfer the ICU and the culture’s different. I get to go for like 1 hour full break. I think most of ICU is like this. Yeah, because we have each other to take care, and we can take over each other’s work. So we get to go like 1 hour break, and usually end on time, or at most 1 hour late. So, so there’s a difference, because it was like once, one meal per day to like a more regular diet right now. But there are times that you can't even go for break in the ICU. Usually these are rare, so I get to eat a balanced diet, I would say.
- **NJCL: So right now, would you say, like, you're trying to change your diet to a healthier one?**
- P20: Also? Yeah, a little bit. Because now I get to eat both like my lunch and dinner. But the type of diet is not very optimal, right? We have usually like half is carbs. Then, you know, like proteins, fruits, and vegetables, but usually- I mean the canteen won't give you that. And I don't know why, but our our our economic stall is quite expensive. So usually we we don't go for that option. Yeah. So you either go for soup base like those Japanese cuisine, Korean cuisine. Yeah. So I will say it wasn't a very healthy diet. So, if possible, I would love to change.
- **NJCL: Currently for you, for your diet. What do you think is your main impetus to want to change your diet?**
- P20: Because I gained quite a lot of weight. [Participant laughs here]. And I get to like, you know, after 2 years I get to like eat a regular diet. I get to go for break on time, you know. So I gained quite a lot of weight. So that’s why right, that’s one of the main factors that I wanted, I wanted to, you know, to change my diet.
- **NJCL: Okay. Then, moving away from diet now on, now onto like your physical activity, how do you feel about the amount of physical activity you're having right now? Do you feel like changing it?**
- P20: It was a- okay, I think for the first years it was quite regular since I stayed near the hospital, and I get to like just walk to the hospital. And there’s a very- there’s a PCM park near that zone, and I was jogging quite a lot. And I had quite a lot of free time. So there’s always work, then I’ll go back, then I’ll go jogging. That’s it. But after 2 years, once I came to ICU, I moved kinda away from the hospital, takes about 1 hour to go there. And it’s not really accessible to PCM park, so I won’t jog there as often. And I got attached as well, so me and my partner don’t usually go for exercise, we usually go and eat a lot. So, my physical activities has dropped quite a lot, but I tried, I tried to you know. There’s like President Health Challenge, this kind of challenge. And join some interest group to go for running this kind of thing and be active as well. But definitely not as active as 2 years back.
- **NJCL: So you mentioned like, it's not as active as 2 years back, but, how did your lifestyle actually change after you started to work as a nurse?**
- P20: After I started to work as a nurse? So the first thing was when I started in general ward, I didn’t go for break. So that was the main issue there. That’s the very big difference first. I didn’t eat as much when I started work. Only after 2 years, you get more familiar then- the culture- I changed to a new ward, started a new culture. They usually like to go for break, then I get to go to a more normal diet. So I think it depends on how is the culture of the workplace is to you- are you able to- usually is the staff able to go for break or not? Is the staffing okay or not? I think it really depends on these, whether you can go for break. And whether you can go have a more regular, healthier diet.
- **NJCL: How about compared to when you were just a student to now as a nurse? Is there a difference there?**
- P20: Student, when I was still in NP, this kind of thing right? I mean we got class, usually we are more- it’s more of an expected, because usually from 8 to 5. But if you’re doing shift work, you have morning, afternoon and night shift. So if you have like night shift, this kind of thing, you have to change your rest time, your eating time all changed. Even like night shift, you have to eat in the middle of the night. Because very tiring and all this. So all these kind of things, it change. Then the next day, you have to sleep- you have to finish night shift then the next day you have to sleep, skip lunch. You only have like dinner to go.
- **NJCL: Okay, then. Away from your physical health. How do you feel about your current lifestyle in terms of like, mentally and emotionally, do you? Do you feel like changing anything regarding those aspects?**
- P20: Hmm, so far, currently, I don't. Because right now, ward culture was very good. We have a very good culture, people are nicer to each other. Yeah, that's why I think, yeah, I don't have any mental issue- mental health stress right now. I would say that the, the culture of the workplace plays a very important part because back then when I just joined ICU- when I joined the ward, the people were not very nice. So it can be a very, a very huge impact for me, for the person who just joined because you don't, you don't have friends there on that particular ward and it can be very scary, and especially if you go ICU, this kind of intensive places. And the people, there’s a lot of so called ‘dinosaurs’. So they have been there for very long, then expect you- they explain to you one time and expect you to perform the same as them. So the stress level is there. It was a very tiring 3 months when I first started the probation period. 3 months of me being there. You just have to pull through, then prove to other people that you are capable to do certain things. Yeah. Then things will start to get better after that.
- **NJCL: And so, okay, then, for my next question, what do you think are your main facilitators or motivators in self regulating a healthier lifestyle?**
- P20: My main motivators, my main motivators. I think probably usually the, you check the weighing scale, you see whether you are, you know. And usually yearly, we have a check-up. Your cholesterol level, your HB1AC, all these kind of thing from the hospital. So you can see how high your triglyceride level, these kind of things. I think you can see from the result, then you see whether you want to improve on which part of your…
- **NJCL: So other than, like checking the weighing scale like having yearly check ups. Is there anything else that motivates you to become healthier?**
- P20: Not really actually. Probably that for me, for myself. Then probably would be to be better-looking for my partner, I think.
- **NJCL: Okay? Actually, maybe you can elaborate on what do you mean by better-looking for your partner?**
- P20: [Participant laughs here]. I mean, I won’t say that my my body shape is very optimal compared to my 2 years back after I was not- I get to get breaks and I eat so much already compared to 2 years back, and my physical activity level wasn’t as good as my 2 years back. So, I would say my body shape not as good as my 2 years back where I was slimmed. So I would love to go back to that stage. I would feel more healthier, although I ate lesser.
- **NJCL: Okay. They're moving away from like facilitators or motivators. What do you think are your main barriers in self regulating a healthier lifestyle?**
- P20: I think I'm not that disciplined anymore. I think discipline plays a very important part. So I used to be able to, you know not drink like those sugary drinks. I am able to hold on to those temptations. But you know, after you started, then it's very hard to stop. Yeah, especially, you know when you are, you're not alone anymore. You're, you have to have more people's feeling- you have your partner's feelings to go to take take care of. If, whether it's he or she wants wants to drink all this kind of things, then you have to go, go accordingly.
- **NJCL: Is there anything else you want to add for barriers other than lack of discipline?**
- P20: I think it's like- you also like work for the whole shift. You feel very tired. So you want to, you know, reward yourself. I think, buy something nice and usually something nice, is usually not that healthy.
- **NJCL: Okay, make sense. Then I'll just briefly, summarize what you shared with me so far. So regarding your main facilitators or motivators. For you, it's mostly the the weighing scale, health check-ups, seeing your own statistics on your health. And also aeshetic wise, being better looking for your partner. These are your 2 main facilitators. Then for barriers, it's a lack of discipline, and also like rewarding yourself after a long, tiring shift. Is that correct?**
- P20: Yeah, I think more- lack of time as well because you don’t have much time, yeah yeah.
- **NJCL: Could you elaborate on lack of time?**
- P20: The barrier of eating healthier right?
- **NJCL: Yeah, the barrier for you. Is it because of the shift work?**
- P20: Yeah. Yeah. Because, because I think quite a lot of us are taking part time degree as well, once you started to work. Yeah, then you have other commitments. All this. So the time that we have is lesser, I would say. Then you only have that few hours of time. Sometimes after work, you have to rush to go for school and all this kind of thing, you usually don’t get to eat a very healthy meal, I would say.
- **NJCL: Is there anything else you want to add here? No? Okay. Then, my next question is on how do you think your self regulation habits impact your work performance?**
- P20: Self-regulation habits impact my work performance, ah. Probably if I get to self regulate myself more well, more disciplined, able to sleep well, lead a more healthier lifestyle. Then my work performance would probably be much more better, I would think. Because you have more concentration at work, and you won’t make so much mistake, I would think.
- **NJCL: Then, would you say you are self-regulating yourself better now? Like able to concentrate more?**
- P20: Compared to 2 years back or when I was a student?
- **NJCL: Both, ah. Both.**
- P20: Both ah. Ya because I think the more senior you have become, the more things you get to know, you kind of know the trajectory of the patient and what kind, what what are things that you expect in the ward. So you kind of manage the things well, so you are not stressful anymore.
- **NJCL: Then, just backtracking a bit, you mentioned like, not that stressful anymore. So do you do any specific activities to like alleviate your stress?**
- P20: I have some hobbies to go. Previously I used to get to run- running is one of my hobbies, which I don't do that often anymore. Now, currently I have my own like orchestra to go. I play some music so I'll go to every once per week. I’ll request like a morning shift, then I would go to the orchestra to play some instruments and go for concerts, this kind of thing. This is something- this is my hobby so usually I go there to de-stress myself, this kind of thing.
- **NJCL: Okay. Then, earlier, when I asked you on like self-regulation habits impacting your work performance you mentioned like better concentration, won't make that much mistakes, right? Then, outside of work, how do you think you being able to self-regulate will impact your life in general?**
- P20: Hmm, self-regulate as in to move myself away from stressful situation, or like when I’m met with sadness, I know how to regulate- like I meet some unhappiness, I know how to make myself more happy.
- **NJCL: Yeah, both like physical-wise, emotional, mental-wise, like being able to self regulate yourself. Mentally, emotionally and physically. Yeah, how do you think that will impact you?**
- P20: Outside of workplace, right? I think this kind of thing is depends on your own mindset. If you are very close minded or a fixed minded kind of person, you will think that all the unhappiness happens because of you, this kind of thing. But if you’re more like a growth mindset, you’re able to direct all those negativity away, you’re able to meet some things and you learn- learn from the past mistakes. What has been done, all those things. Then, you won’t be so upset and be able to move on from there.
- **NJCL: So currently. Earlier, you mentioned that your main impetus to change like, why you want to change is because like you've been gaining weight and you also wanna like aesthetically look better for your partner. How do you think you can increase this determination to change?**
- P20: How do I increase my determination to change? It will be best. If my partner can do it together with me, I will say. And the rest. I would think that it's more of your self discipline to try to improve your self discipline.
- **NJCL: Would you elaborate on that like? How? How exactly would you increase your own self discipline, at least personally for you?**
- P20: How do I increase my self-discipline? I think discipline is something you start from small things first because I think let’s say you suddenly increased, we jump straight away into reducing your weight, and then we see you back a few months later to see your progress, it can be quite tough.You need to have a lot of the willingness to have to be there But, I think, if you start small like, if I'm able to do some small thing, let's say, I fold my blanket every morning when I wake up. So you consistently do it, then, let’s say after 3 months, you have the habit of doing that then, you might think that, actually I’m able to do this, then we start with another- something else. We start from folding blankets then slowly go to stuff like eating sugary drinks, then after that we can slowly move on to running once or twice per week. We start from small things and be able to move forward with those kind of things is slightly a little bit tougher- will probably have a bit higher chance to success I would say. Then once we are successful, we are probably able to go for more tough things like losing weight. [Participant laughs here].
- **NJCL: Makes sense, makes sense. Then, what are your thoughts on nurses as role models for health, do you think, for example, your patients, look up to nurses when you know when you promote health to them?**
- P20: Yeah, I agree on this point because but it's it's very hard for us to practice also, because you have to be it's, it's very ironically like you ask your patient to go on diet but you yourself not having a you know. If you are obese then you are asking somebody, some of your patient, those obese patients, to reduce their weight, it’s not very convincing for them also lah. I will say that it will be very important for us. I think it depends on how the nurses look on their perspective right now, because if you are- if you have those mindset of I’m coming to this world to have a good life but not a long life, you probably can afford to have all these, you enjoy all this food you can have, then one day you die of heart attack, but at least you enjoyed what you have been doing, for the past, for your whole life. But if you’re looking for a, you know, I want to live long, I have yet to see my grandchild, this kind of things, then you should have tackled the patient from there lah. But of course if you are a nurse, you should advocate for this kind of things, then we should become a better role model for our patients to look up to.
- **NJCL: Then, personally, for you like, like you mentioned, if you promote like healthy eating to them, and then you yourself don't really engage in healthy eating as much, do you, for example, feel guilty or something like that?**
- P20: I will probably feel guilty, but what to do? [Participant laughs here]. But, thank God, I don't discharge patient that much. Because in ICU, it’s either they go to heaven or they transfer. Ya, so my nature of work, I don’t really need to educate them as much as when I was in the general ward. But when I use to be in the general ward, my body shape wasn’t that bad, so I was able to kind of advocate for a bit and be quite a good role model for them.
- **NJCL: Hmm. and then my next series of questions will be involving, like the workplace, the workplace in general. So for you as a nurse, how do you think the workplace influences your own like self-regulation, lifestyle habits?**
- P20: Hmm! I think if let’s say- because currently right now, our ward is very active because we have quite a few new nurses join us, and the culture has been changed. We have quite a lot of outings together. We will sometimes go organize, have hiking together, or go running together or may even go walk after work sometimes. So, I think this kind of things help with better mindset for us to help us to bounce back and be more resilient, I would say, and you know. But the downside is that we will always go and eat some good food, that’s the thing.
- **NJCL: So like for you, because the the workplace environment is like, it's a very active environment, it encourages you to, to move about more is that right for me to say?**
- P20: Yeah.
- **NJCL: Okay. Then, moving on to like the social aspect, how do you feel about the workplace culture among nurses in helping to facilitate or hinder self-regulation of healthy lifestyles?**
- P20: You mean my workplace culture?
- **NJCL: Yeah, your workplace culture with you and your fellow nurses. Do you think it helps you to facilitate a healthier lifestyle?**
- P20: Some of them. Because we usually go for break as a whole, because we will set breaks usually, so lets say 5 o’clock is our first break, 6 o’clock is our second break, so we see first who’s the first break, then we will go for break together, so I think probably if let’s say we go down together, some of them would want to eat more healthy options instead of soup- instead of laksa, we get to choose soup-based. So, some of them might want to try things together which will be able to affect you to choose not the very bad diet, I would say.
- **NJCL: So, something like peer pressure to get you to-**
- P20: Yeah, I would say that too.
- **NJCL: Then how about something like hindering you from healthier lifestyle. Is there anything in the workplace culture in your workplace that promotes bad habits, something like that?**
- P20: Hmm! I think. Probably like what I said earlier, because in the work, some more in nursing, you worked hard during your shift then when you finished, you just want to relax. Either you don’t want to exercise, you want to relax, and go back home and have a good rest, or you want to have a feast. Yeah. I think for me, that’s usually the main two goal after I want to get out of the workplace. After a really bad shift, you just want- either you want to have a very good meal with your friends or your colleagues, or you just want to go home and rest and prepare yourself for the next day.
- **NJCL: Then, talking about like nurse managers, what do you think about the role of nurse managers in helping the nurses self-regulate their own health?**
- P20: My own health?
- **NJCL: Yeah, like the health of like the ground nurses; both mentally, emotionally and physically. Spiritually, even**
- P20: Hmm! How my manager will- how to help us, is it?
- **NJCL: Do you think they play a part in helping the ground nurses?**
- P20: Because usually they will do rosters, so if you are able- because sometimes we will have shifts where its night shift, night shift, off, then morning. So it means, that on your day off itself you finish work at about 7.30 or 8 o’clock, so by the time you reach home it’s about 9, 10 even? Then you have to rest, to sleep until 3 or 4pm, then when you wake up, you only have so many hours left before you sleep again to prepare for your morning shift the next day. So by preventing this kind of thing, I think you as a manager are able to, you have a more, you give your ground staff a more healthier life. More work-life balance. Because usually after night shift then your afternoon shift, you get to rest more well, have more time to adjust your body clock I would say. And if possible, I'll say that if, if those people need to take some leave. Emergency leave, then it would- if the ward permits-I think if you are able to allow this kind of things, you will probably help the staff a lot, honestly. For their mental health at least. Because some people might be at their breaking point and they just want some leave to rest for awhile.
- **NJCL: Then for you, how do you think your institution can help you to self regulate a healthier lifestyle?**
- P20: Hmm, because I think because [Public hospital name] did quite well, because they have quite a lot of, we have October healthy living month. We’re on this month we have quite lot of events ongoing like Zumba outside of our hospital. Bowling. We use to have a yearly running thingy to participate. Next week we have captain’s ball, this kind of thing to let us have a more healthier lifestyle. We have some talks as well to let us know what kind of healthy diet we can have. Then what kind- a few talks I can remember- what kind of- some memes about running. Then, we have some interest group also, quite active. I think these few things are the things that let us, you know. I think they have these because their motto is ‘healthy living together’ if I’m not mistaken so they were trying to have a more healthier lifestyle, this kind of thing.
- **NJCL: Then, personally for you, have you attended or like participated in these events like your yearly running? Or the Captain’s ball?**
- P20: Yeah, yeah. So Captain’s ball I’m playing next week, for the first time. I’ve never played Captain’s ball before. Because- back to my point previously, because my ward is currently very active- I don’t know why. I think by nature, I see people are very competitive, so we are joining this one to want to fight for names. Then previously we had the [Public Hospiital cluster] President Challenge. Like half of our ICU joined to run to clock against other institutions to have you know, better healthy living, to fight for number 1 or number 3, this kind of thing. Ya, I think that summarizes my point
- **NJCL: So for you, would you say it's effective in encouraging the nurses to actually maintain healthier lifestyles?**
- P20: I think for institutional wise, I think they did quite a lot. So I would say, yeah, because I do see some nurses join those kind of things. To have, you know, to have a break from those kind of work.
- **NJCL: Then like other than your running, and a bunch of activities organised by the institution, as much as you know, is there any like workplace health promotion programs in place?**
- P20: Workplace health promotion program? Hmm, I don't think we have this health thing. You mean, as in my workplace. Whether I have any any health promotion program like ‘Eating healthy together’ this kind of thing? I don’t think we have. I think usually this kind of thing have to have someone to encourage- to be the one to lead and organize this kind of thing. If the ward doesn’t have, then it means doesn’t have.
- **NJCL: Okay, then, a bit unrelated, but do you currently use any like tools or apps to help you to self regulate your lifestyle?**
- P20: I used to have, because I think I have trouble sleeping. Yeah. So I used to have this pokemon sleep. Yeah. So it. It kind of help, helps me to kind of want to sleep earlier, I will say, because I want to collect more points, these kind of things. But now no more, I don’t want anymore. Very lazy to use it.
- **NJCL: But you’re still able to sleep?**
- P20: Still able to sleep, just kind of go back to the old cycle again, to force myself not to sleep so late anymore ah.
- **NJCL: And then I'll just summarize what you've been sharing with me so far, so on my question on the how do you think the workplace influences your own self regulation, habits? You mentioned that because right now, your workplace is very active, so it's, it allows for having a better mindset, to allow you to bounce back with more resilience, encourage you to eat regular meals because there’s a culture of going of going, for breaks compared to your general ward. Is that right so far?**
- P20: Yes, correct.
- **NJCL: Okay, then, on my question, on the workplace culture among nurses in helping to facilitate or hinder self-regulation. You mentioned that the workplace culture is quite positive, you know, going for breaks together. Then, while you guys go for breaks, you are like peer pressure to eat healthier- people will choose the healthier options.But then, at the same time. There's also this culture of like rewarding yourself after a long break. So you you might just want to like feast after after your shift. Then you also said, like the role of nurse managers is- because they’re in charge of the roster, they play a part by actually facilitating better work life balance for the nurses on the ground, making sure that if they want to take emergency leaves, they are able to and all this.**
- P20: Hmm. [Participant nods head vigorously].
- **NJCL: So far, is there anything else you want to add in any of this?**
- P20: No.
- **NJCL: And then, next thing is regarding the institution, you said institution wise, they actually do quite a bit, like the yearly running, captain’s balls, the talks, active interest groups. You even said like ‘Healthy living together’ is the motto. Then, just to clarify, would you say that the institution is doing sufficient for the ground nurses, or do you think they can improve, somewhat?**
- P20: I will say that everything won't be perfect. There will always have room for improvement. It'd be great that, like, you know, every month we have this. then people will definitely join, but it will be a very tremendous work for them. Which is I think, quite unrealistic for them, I would say.
- **NJCL: Then, if you would maybe make one suggestion to the institution, what what will you want them to implement so that it will effectively encourage more nurses to embrace healthier living?**
- P20: Okay, I think because I'm a more active person, if possible, if they had more incentive for people to join, to join like ah, let’s say every month, have like a running interest group- a running competition, those people who clock in certain hours of running or walking, they are able to get like, $5 or $10. I think this will encourage people to join more.
- **NJCL: Okay, then just further clarifying and summarizing, when I asked on the workplace, health promotion programs you're not really sure how, not really sure about this. Then, when I asked about the tools that you personally use for self regulating. You mentioned that last time you had used a pokemon sleep to to help you because you are having trouble with sleeping. But now you're not using it anymore. Is that all correct?**
- P20: Yeah, correct. Correct.
- **NJCL: Do you wanna add anything else here at this juncture?**
- P20: No.
- **NJCL: Okay. Then you have anything else that you would like to speak about that was not mentioned in the interview. Any further comments you want to share?**
- P20: No, I don’t, I don’t have.
- **NJCL: Then, this marks the end of the interview. Thank you for your time and participation.**
- P20: You’re welcome, it’s okay.
- -End of P20 Transcript-

# **Interview #21**

Participant and interview details
 Participant number: 21 (P21)
 Date of interview: 23 Oct 2023
 Interviewer Number: Navarro John Christopher Lambino (NJCL)
 Time IDI started: 1100
 Time IDI ended: 1154
 Observational notes: Participant seems to be in a pleasant mood.

Section I: Interviewer’s summary
 The individual interview was conducted via Zoom. Before the interview, P21 mentioned that he was on leave, which may explain his well-rested demeanour. For context, P21 is a public hospital nurse, working in an orthopaedic-surgical ward, with a nurse to patient ratio of 1:15. 2 years of nursing experience. At the end, he revealed he was working in biotech beforehand. Perhaps, I missed an opportunity to ask him more about it as a point of comparison. P21 did not introduce any new ideas or concepts that previous participants have shared. Manpower issues is a major concern for this participant, citing the stress and how his own health habits are affected due to this. He also expresses that from seeing his patient’s own conditions, it motivates him to take care of his own. The workplace culture of his ward also seems positive albeit high patient case. Throughout P21’s interviews, his responses seem very short relatively compared to other participants. As such, multiple prompting questions were utilized to ask him to elaborate more. While his answers were sometimes answering to the point, some of his answers require some interpretation before a link can be established to the question. Lastly, halfway through the interview, P21 had to excuse himself to take a delivery package from his front door. There was no other significant distractions other than this. Overall, the interview did provide some more insight of what nurses may have experienced. However, as this participant undergone a career change, I should have asked more on the comparison between the nursing occupation and his other occupation as a point of reference which my other participants may not have had.

Section II: Transcript

- **NJCL: Alright. Good morning, sir. Firstly, I would like to thank you for taking the time to speak with me today. My name is Chris, and I'm a NUS Year 4 nursing student. The aim of this interview is to get a better understanding of the perspective nurses have on self regulating their health behaviours, and how their different factors may influence this. I want to find out what are your views, opinions, and experiences on this and I would really like to encourage you to speak what comes to mind There are no right or wrong answers. May I have your permission to continue with this interview session?**
- P21: Okay, please continue. Thank you.
- **NJCL: Okay, thank you. And then let's start with some basic questions like, which ward are you working in right now?**
- P21: I'm currently working in a orthopedic surgery ward. In [Public hospital name].
- **NJCL: Can you describe to me, what kind of work do you have to do on a daily basis?**
- P21: What kind of work do I do on a daily basis? Hmm, so far, I've been managing patients ranging from handling IV medication to showering patients. Phlebotomy, it’s a thing with IV cannulas. Drawing bloods, to even changing diapers and other miscellaneous tasks. As well as liaising with doctors, physiotherapist and other allied healthcare workers.
- **NJCL: Then, how would you describe the workload?**
- P21: So currently for my workload, I’m taking. Because usually in my ward, 1 staff nurse and 1 enrolled nurse or one other staff nurse, 1 in charge and 1 junior to 15 patients. So I am usually overseeing 15 patients in a daily basis.
- **NJCL: Then. How would you would you say you're able to manage the workload?**
- P21: Been trying to manage it. But able to manage is a [Participant hesitates]. I mean I think it would be more ideal if I had lesser patients to manage, I suppose. Because I feel like my attention is stretched a bit across 15 patients. And sometimes I’m not able to give them the best care that I can because my attention is spread over so many patients, I can only allocate sometimes my attention to 1 patient when things get a bit more complicated.
- **NJCL: Hmm! Then, just to clarify a bit. When you graduated, you started work at an orthopedic ward, is it?**
- P21: Sorry?
- **NJCL: You were in an ortho ward from the very start? You didn’t transfer from another ward?**
- P21: Yes, I've been in the orthopaedic surgery ward for 2 years.
- **NJCL: Alright, then, thank you very much. My next series of questions will be regarding lifestyle in general. So for you, as a nurse, how would you define a healthy lifestyle?**
- P21: How I define a healthy lifestyle? I think it would be more of like having a balance between like not just working. Because a lot of times like we are also working like a lot of times because of shift work. And also we're not getting enough rest. So I think, it's important for healthy lifestyle for nurses would be work-life balance which is one important aspect of it that I find very important. Because, sometimes we are spending too much time at work that we don't really get to rest well or rest properly before night shift. And also for the second part, I think a strong support system, because sometimes nursing can be very emotionally taxing. And we see a lot of cases from day to day.
- **NJCL: Okay, then. specifically on diet. How do you feel about your current dietary habits? Do you feel like changing it?**
- P21: I think my current dietary habits is okay. But I think, what are things I need to look out a bit more for because I'm currently transiting back to 3 rotating shifts. Yeah. Because previously I was on permanent night shift. I am going back to 3 rotating shifts. So when I go back to tree rotating shifts. One thing I need to watch out for is something that tends to happen more often, I have noticed, is that I don’t get to eat my lunch or dinner when I go for morning or afternoon shifts, which is something that can be quite worrying sometimes. So I think that's something I need to watch out when it comes to dietary habits.
- **NJCL: But would you say, like your current dietary habits is healthy?**
- P21: I think it’s healthy because I’m not particularly eating very oily foods. I also tend to watch my diet a bit and try to eat less salty and less oily food. And also less sugar as well.
- **NJCL: Okay, then. Moving on from dietary habits. How about your physical activity-wise? How do you feel about your current lifestyle? Do you feel like changing your current physical activity levels?**
- P21: Physical activity? I think I am currently maintaining well as well. But because I’m currently doing bouldering and dance outside of work, which is something that helps me to take my mind off which and I do it on a regular basis. Maybe at least once a week for both.
- **NJCL: So your current, like physical activity level, is you would consider accebtable?**
- P21: Hmm, yes. if I have the opportunity. I would like to push it out quite a bit. But if time permits, I suppose because sometimes it's very difficult to balance between work and also my other normal activities outside of work.
- **NJCL: Will you perhaps tell me like, why you feel like you want to increase the physical activity level?**
- P21: I guess it’s more of like I want to progress in my in the physical activities also. Because for bouldering and for dance also. It's like, to want to go further into the hobby as well.
- **NJCL: Okay. Then, moving away from like physical health. Earlier, you mentioned something like having a strong support system because nursing is emotionally taxing. Do you do anything for your emotional, mental, even like spiritual aspect of your lifestyle?**
- P21: So far, I think for me it's been quite, quite a strong support system in place, because the people that I work with are people that I’m relatively close to. And not just that, but my family and my friends are also very supportive even though a lot of my friends are also in healthcare. I guess like it comes with shared understanding, because we all times we face the same thing, but in different environment, different situations, but very similar situations. So I guess it comes with that kind of shared understanding whereby we kind of understand each other.
- **NJCL: So just to clarify a bit. Currently, your emotional mental aspect is well taken care of because you have a very supportive social support system, is that right for me to say?**
- P21: Yes, right.
- **NJCL: Then, could you tell me, how did your lifestyle change after you started to work as a nurse?**
- P21: How did my lifestyle change after I started to work as a nurse? Hmm.
- **NJCL: Like maybe compare yourself now, working with you as a student?**
- P21: I think when I was a student it was like a lot less stressful. And like I had a bit more time to use on my hands. But I think the first time when like I entered the workforce as a staff nurse, there was a really big change in like a lot of stress, in that time. Because transiting from a student to a staff immediately has a lot of- there’s a lot of pressure in taking up new responsibilities, and also like. You can’t really, how to say. Now, a lot of people look up to you as a staff nurse, as compared to when you were a student. You are expected to understand and know more. So, I guess that was one aspect where there was a lot more stress compared to when I was a student. And also I think a lot of times, it was a lot more physically and mentally taxing as well. Because in orthopaedic surgery ward, I help patients with a lot of ADLs because a lot of times they come in for surgeries whereby they tend to require more rest in bed, initially, before being assessed by the physiotherapist. Then mentally also because I was pushed quite fast into managing 15 patients, from my past probation, I was actually the first to be pushed out to take more cases compared to my colleagues who came in together with me.
- **NJCL: So you mentioned on stress, right? Maybe, tell me more, what is your main coping strategy for the stress you’re feeling?**
- P21: Main coping stress strategy at that point in time, is it?
- **NJCL: Oh, so it changed, is it? Then you can share like at that time. And right now**
- P21: I think at that time, when I first started, I tend to clamp out a bit and just listen to music at night time everytime I’m home. But as of now, usually I would more likely to talk with my friends or go out. Usually, I would take a day to unwind, after my shift, which is something important for me. Because sometimes, usually the stress accumulates over the shifts. And then after that I will go and unwind after that so at least it’s a break.
- **NJCL: So there's a difference in how you deal with stress compared to before and now.What would you say caused this change?**
- P21: I think it's more of like the decision of realising that I had to adapt towards shift work. And also sometimes because- in terms of shift work we don’t really have a lot of time to rest. So I had to adjust my coping strategies with more towards what I can do with the limited time I have compared to what, uh, how I initially started with.
- **NJCL: [Long pause]. Okay. Thank you. So my next question is, I think there are bits and pieces, you answered here and there. But, as a nurse, how do you feel about self regulating a healthier lifestyle?**
- P21: How do I feel about self-regulating a healthier lifestyle? Having plans can be a bit difficult also. Especially juggling between nursing itself because we are already on 3 rotating shifts. And a lot of times it’s the physical and mental stress that gets to us. And sometimes in the end we may succumb to more unhealthy habits, more often than not. So sometimes can be really difficult especially with a lot of stresses at work. And not just stresses at work, but can also be stresses at home that people face.
- **NJCL: Other than the difficulty like, what do you think nurses should or can do to self regulate a healthier lifestyle?**
- P21: Should or can do, is it?
- **NJCL: Yes.**
- P21: To regulate a healthier lifestyle? I think more like than not is to participate in some form of physical activity, although that because, like some actually, a lot of our work also is very physically intensive. It is an aspect which is something that I realize is important is to actually keep ourselves healthy and active because a lot of times, I have colleagues also who get injured at work also because sometimes things happen. And sometimes we don’t train enough. So, it’s also important to maintain that form of or level of physical activity so at least our body can keep up with the job as well.
- **NJCL: Then, my next question is: For you, What motivates you to self-regulate a healthier lifestyle?**
- P21: What motivates me ah. I think more likely than not, I guess, I still have things to do. And also like, places I wish to travel to, in which a lot of these activities, if I do not- I’m not healthy, I probably can’t achieve. So probably, that’s the reason why. In which why, I need to keep myself fit. And also to make sure I am able enough to achieve these things I want to do, I wish to do.
- **NJCL: Then, building up on this question, what do you think are your main facilitators in self regulating a healthier lifestyle? What helps you self-regulate? Like what motivates you?**
- P21: What motivates me, ah? I think part of it is like intrinsic motivation in which I wish to achieve certain things whereby I need a healthier lifestyle to achieve so. But I heard that a lot of times also, having a company of friends helps as well. Because sometimes, doing physical activities alone can be a bit hard. But having friends around really motivates me to keep healthier also. And also helps to look out for my health also.
- **NJCL: So for you so far, your facilitators are intrinsic factors like being able to do what you want to do, when you wanna do. And also having the company of your friends. Is there any other facilitators or motivators in self-regulating a healthier lifestyle for you?**
- P21: I guess, like also, seeing, because, like we witness patients in and out of the hospital, like day to day lah. So something that also kind of hits me is that is like when I see them, in the hospital in such a condition, right? It kind of successfully reminds me that I do not wish to- that I need to keep myself healthy, and to not end up like them and also to watch out for my own health in order to make sure I can stay healthier when I reach that age.
- **NJCL: Okay. Then, moving away from facilitators and motivators, what do you think are your main barriers in self regulating a healthier lifestyle?**
- P21: Main barriers? I think long nursing shifts is one of it. For this aspect is also like like, I think it's a multi factorial like I will put it under nursing shifts. Because the thing is that, sometimes, a lot of times, one thing that I realise is, my colleagues and I realise is that we don’t really have time to go for break. That is one. So we don’t really eat until the end of the shift. For example, when you reach the end of your shift after an afternoon shift, about maybe like 9:30 pm. Like one important factor also that I sometimes often see, that there is also less food options available whereby all the food options aren’t very healthy either. Yeah, so it’s multifactorial. I don’t know how to break it down to something simpler. Just something also is the acute stress of working in a very stressful environment such that we do not have the time to even step out for a breather. So, a lot of times, my colleagues and I, we don’t even drink water or go to the toilet because we don’t have time. Other barriers include, I think maybe the tiredness from shift work. Because a lot of the time we are on 3 rotating shifts, we can be very physically and mentally exhausted, such that we do not wish to spend our off days doing more physical activities.
- **NJCL: Then, I'll just briefly summarize what you've shared with me. For your facilitators or motivators for self regulating a healthier lifestyle, you mentioned having the intrinsic factors having a company of friends help to look out for your health, and also witnessing patients and like hoping you don't end up like them. Is that correct? So far?**
- P21: Yup.
- **NJCL: Okay, then, for barriers you mentioned along the long nursing shifts. Then also, during the shifts, there might be no time to go for breaks, even drink water or go toilet. There's also less food options and the food options available are usually not very healthy. And then there's also the acute stress from working in a stressful environment in the ward, which I’m guessing will mentally tire you out from participating in physical activities outside of work. Then you also mentioned fatigue. Because you're too tired after a long shift, you don't feel like doing anything once you get out of the shift. Is this correct?**
- P21:Yeah.
- **NJCL: Is there anything you want to add for either facilitators or barriers?**
- P21: I think not for now. Okay, thank you.
- **NJCL: Can. Then, okay, my next question would be, for you, how does your self regulation habits impact your work performance?**
- P21: How does my self-regulation habits affect my work? I think, for me. I think it’s important for me to take my off days as the days to unwind down as well. Because for this, if not, work can be very stressful for me also. So I tend to go to work early, like maybe an hour, just to read up on the cases so I don’t get to sleep a lot either. Because I tend to be a slight insomniac also. I think the days to unwind really help me to get back and refocus on my job. Because if I don’t have this platform to unwind, to the point whereby I will probably be too stressed out to focus very well at work. With regards to physical activity, also, I make sure that I need to keep myself fit. And also I think another important point is the body posture when shifting patients so I at least I do not get injured on the job as well, which is something very important to me because I’ve seen a lot of my colleagues get injured at work.
- **NJCL: Okay. Then, maybe outside of work, how do you feel self-regulation affects your life?**
- P21: Outside of work? Self-regulation helps me to just be happier and more motivated in life in general. In the sense, I get to enjoy things that I wish to do, while also keeping myself healthy as well. And also like, it’s also important for me to look out for myself, its also an a way for me to unwind down, like really relax outside of work. Compared to when I’m at work and there’s a lot of things in my mind. So it helps to relax myself a bit more.
- **NJCL: Okay, just on a tangent. Are you using any tools or apps that help you to self-regulate your lifestyle?**
- P21: Not currently. Not that I know of.
- **NJCL: [Long pause]. Okay, then. My next few set of questions will be regarding the workplace in general. So like for you. As a nurse, how do you think the workplace influences your self regulation habits?**
- P21: I think like what I mentioned previously, when I went back to 3 rotating shifts, it came to the point where I had to skip meals. And also not drinking enough water. I think that’s also one issue of self-regulation, where I could control but a bit difficult because of the patient load. Then, in terms of stressors, I would say that work itself is also quite a stressful situation, in which like it tends to motivate me towards taking proper rest on my off days. So, in a sense, it makes my rest hours more essential, I would say.
- **NJCL: Sorry. Could could you elaborate on that?**
- P21: Because, like sometimes, when ever, if I'm too stressed out, right. I will. I guess it also makes me because, there was a period of time where I actually didn’t do anything on my off days. And just went back home after work to rest and just go back to work. I think at that period of time, I really felt like, like I really felt terrible. I did not have any motivation to go to work, I had no motivation to do anything else. So, in which I would say that, what I do outside of work is also very important to get myself back towards doing things, doing work itself in the hospital.
- **NJCL: Now, regarding like the social aspect of the workplace. For you, how do you feel about the workplace culture among nurses in helping to facilitate or hinder self-regulation of healthier lifestyles?**
- P21: Hmm! I think for my ward… I think it helps that. My one has a very good teamwork culture in which, like we all have to look out for each other, as well as to make sure by as much as possible, try to make sure that, that some of us can at least go for our breaks, and also to make sure that we have enough water or have enough time to go to the toilet. So I think it's this little things that can help with like, influence us to have a more healthy lifestyle, because, a lot of times our work is very stressful. But these, my colleagues also also look out for each other, and also like will help. Will also come over to help. When we need it, lah.
- **NJCL: So far, okay, so you highlighted like the teamwork culture like the positive aspect of your workplace culture. Do you think there are any negatives regarding your workplace culture that maybe hinders your self regulation of healthier lifestyles?**
- P21: Negatives, ah? I think the high patient workload. Because I have talked to other friends in other hospitals, in which I have heard that they are taking 6 to 10 patients. Whereas comparatively in my current hospital, we are taking 15. Which is a very, about 1.5 times the patient load. And it’s also something very heavy that tends to be very stressful as well.
- **NJCL: Then what do you think about the role of nurse managers in helping the ground nurses to self-regulate their own health?**
- P21: Nurse managers, is it? I think for nurse managers, something very important also, I think like middle management is also facing a lot of issues in a sense that they find it very hard to ensure adequate staffing. That is one thing. Because sometimes we tend to get called back on our off days as well. So this is what can result on a negative impact on us trying to achieve a healthier lifestyle. But I think at the same time, the nursing shortage is a worldwide phenomenon which is a bit of issue that is hard to address as well. Nevertheless, what our nurse managers can do is like, we have our allocated and protected off days as such that we do not have to be forced to come back. Even like, because I find that if we do not rest enough, then we won’t be able to do follow up properly. Which is something very important when it comes to healthcare, because not only for the time we are in healthcare but we are also constantly nursing patients everyday. [P21 Doorbell rings]. Sorry, just give me a moment. [P21 gets up to acquire his delivery].
- **NJCL: Okay, no problem. [Long pause].**
- P21: So sorry about that had to go and collect a delivery.
- **NJCL: Okay, no problem, no problem. Then my next question is: so for you, you think the nurse manager role is just to help the ground nurses ensure they have protected and allocated off days so they can rest properly. Is this correct?**
- P21: Not only that, but I feel like they can also help to encourage more healthier lifestyles in the sense that, like provided that we can actually- I think if possible, maybe they can plan activities in which they can bond colleagues as well as to give us a bit of time off work as well.
- **NJCL: So does your nursing managers in your ward do this kind of thing? Do they usually organize?**

P21: I don't think so. Not that I know of.

- **NJCL: Hmm. Okay, but you hope this happens?**
- P21: I hope so, but can’t say for sure if they will ever.
- **NJCL: Okay. Then, would you say, like the nurse managers are role models for healthier lifestyles for you? At least in your ward?**
- P21: Are my nurse managers are role models for healthy lifestyle? Don't think so. Unfortunately, no, I don't think so.
- **NJCL: Hmm. Could you like perhaps elaborate, why you don't think they’re role models for the nurses on the ground right now?**
- P21: Because more often that not, I always see them stay back very late often. Like work tends to be very busy for them also as well as us. So they also do not go for their breaks as well. And a lot of times I see them leave work late so they come in for morning shift at 7am and then they leave work at 7pm. So it feels like, although I do expect them to take their job seriously but at the same time I can see that they are also equally- we are all equally overwhelmed in the situation as well. In which all of us are not having the best lifestyle as a whole. Because we are all working very hard in healthcare.
- **NJCL: it. And okay, then, with the same idea, right. Do you think the nurses on the ground are role models for patients to self-regulate their own health?**
- P21: Nurses for patients, is it?
- **NJCL: Yeah? Like, nurses as role models for patients to self regulate their own health. Do you, do you believe in this? Do you think this is so?**
- P21: I will say yes, because, not only just that, in a sense that, like we are the ones who I hope to stay fit enough to actually take care of them, and also to encourage them to like participate in their own ADLs also. Also like important to show them- because we will not always be there to take care of them. They need, as much as possible, they also need to be independent and on their own as much as possible. So that is something we have to advocate for, and also something important and based on that, we’re a role model, ah.
- **NJCL: Hmm. Okay. Then, for example, there are some nurses who don’t have the best self-regulation habits. And then they will provide their patient’s with health advice. Do you think their own self-regulation habits will affect how well they can provide health advice to their patients?**
- P21: I can't say for sure, but overall, I feel it does not. I feel like overall they are still able to provide good advice for their patients as well. Although their self-regulation habits may not be as good, but ah, I think they are still able to provide adequate advice on the form of what to look out for, and things that they should take note of as patients who have many chronic conditions.
- **NJCL: Okay, okay. Then, lastly. on this this idea. So would you say nurses prioritize patients health more than their own health?**
- P21: I will say yes. Because, like a lot of times like when we see our call bells, or like, we see the things we need to do. A lot of times we will set aside our own health, in the aspect where ‘oh I have to go for break and like drink water and to go and recharge’ or ‘oh, I have to use the toilet’. A lot of us will just set it aside and go do it later. I need to prioritise my patients’ first. So yeah, that’s what usually happens. It’s not the best thing, but yes.
- **NJCL: hmm! Could you maybe like elaborate on why you think this happening? On why you think the nurses are not prioritising their own health as much?**
- P21: I think as nurses, we are people who are very giving. So sometimes we also tend to give a bit too much of ourselves to the job, which is something that’s very sad but very true. And like what happens is because we see someone in need, like our patients come in and they have a lot of pain, or they need assistance with going to the toilet. Or, any other miscellaneous things. They need medical treatment, or they need any help with ADLs or anything. We will always prioritize them first because we see them in need, they are highly dependent on us.
- **NJCL: Could you maybe suggest how we can get nurses to prioritize their own health more?**
- P21: Prioritize their own health more? Safer staffing ratios. Definitely, for one. I think because if we have safer staffing ratios on the ground, it will be easier for us to have a breather and go for our breaks, or to drink water. Like to just really take a break from work, because we are supposed to have allocated 1 hour break for our dinner time or actually to take a break in general. But a lot of times, for my friends or colleagues who go on breaks also, a lot of times what happens also, they will also be writing their reports. While trying to eat their dinner, in a very fast manner. Even on break we are still doing work. Like the stress is always constantly running through which isn’t good. So I think adequate staffing ratios will really help.
- **NJCL: Okay. Other than having safer staffing ratios, how do you think your institution can help you to self regulate a healthier lifestyle?**
- P21: My institution? I think it would also be organize, maybe like, seminars or like, help introduce all the nurses to healthier lifestyle habits or even like how to eat healthier, how to maybe cook healthier meals at home. This things will help a lot also, because I feel we don’t have this avenues available for us.
- **NJCL: Hmm, okay, then, just to clarify on this point. You said like you want talks to be organized for nurses on how to eat healthier. So, do you believe that nurses don’t have this knowledge, or is it lacking?**
- P21: I think it's like lacking in a sense- I would say, spend a lot of time in the hospital such that we tend to- we have that knowledge we tend to just, uhh, not use that knowledge when we go back because we’re too tired. So maybe a form of positive reinforcement, I suppose.
- **NJCL: And then currently. do you know of any workplace health promotion programs in place in your institution?**
- P21: I think for my institution, currently, they are focusing a lot on staff well-being also. So they are looking a lot into- they have quite a few mental health hotlines. In which you can speak to like counsellors or people who are willing to listen to you if you are too stressed out. And all this also. Then, they are also looking into protecting their nurses from abuse, which is also very important.
- **NJCL: Could you elaborate on your last point?**
- P21: So for, what our hospital is doing for our staff, they are also advocating for staff wellbeing by preventing abuse in general. So this is something I feel is very important as well. They also not just looking towards preventing it also to educate the public with regards to this as well, because I think something important also is that when we do our jobs also, we want to feel like we can our job more safely. Rather than if we do our jobs with the potential- like with the fear of the getting abused physically, mentally or emotionally at work.
- **NJCL: Then, I'll just briefly summarize what you shared with me regarding the last few questions. So on the question on, how do you think your institution can help you to self-regulate a healthier lifestyle? You shared that maybe they can organize like seminars or more seminars to like, maybe talk to nurses on how to better self regulate, and then you feel like, even though the nurses have the knowledge, but they tend not to use it because they're too tired. So, having a form of like positive reinforcement can help with this. Then, on the question on workplace self-promotion programs. You mentioned that the staff wellbeing is being advocated for in terms of like preventing abuse, educating the public to ensure this helps to make sure the workplace environment safer. Then there's also mental health hotlines to speak to people for those who are too stressed out for work. So this is a platform for them to talk to someone. Is this correct so far? Do you have anything else you would like to add for this part?**
- P21: I think no, not currently.
- **NJCL: Okay. Then do you have anything else that you would like to speak about that was not mentioned in the interview? Any further comments you want to share?**
- P21: I think not currently.
- **NJCL: Then, this marks the end of the interview. Thank you for your time and participation.**

P21: Thank you

-End of P21 Transcript-

# **Interview #22**

Participant and interview details
 Participant number: 22 (P22)
 Date of interview: 26 Oct 2023
 Interviewer Number: Navarro John Christopher Lambino (NJCL)
 Time IDI started: 1200
 Time IDI ended: 1320
 Observational notes: Participant sounds exhausted.

Section I: Interviewer’s summary
 The individual interview was conducted via Zoom. Due to camera issues, was unable to see P22. However, from her voice, she sounded very tired. For context, P22 is working in a public healthcare institution, with a focus on psychiatric patients. P22 has given very detailed and comprehensive answers without much prompting. The flow of the interview was very natural as her responses overlap into the following questions without needing to be asked as she would elaborate on her own without much prompting. However, much of her responses has been mentioned by previous participants. Throughout the interview, she sounded tired. Despite the high workload and staying over beyond shift hours being commonplace, she believes that the workplace culture is a positive one as she and her colleagues would be in the situation together. She also went on about how her nurse manager recently changed, and the new manager would disrupt her work-life balance as she would contact P22 outside of shift hours to work on a certain shift. She mentioned that nurse managers are not the best role models but they do try, although, they should be primarily focused on rostering before they can even begin any supplementary initiatives. She believes that management is not doing enough and there is a lot of improvements to be made such as influencing the kind of food options available at her institution. The most notable part of this interview may be on the workplace culture and her responses regarding the role of nurse managers. She emphasized on the importance of good leadership and how it may help to alleviate unnecessary stress which can then translate to healthier practices such as going for a walk after shift. In conclusion, this interview was very smooth, as many of the questions has been covered by P22’s responses before they were even asked. Perhaps, for further improvement, I could have asked her to elaborate on leadership practices that were pro-healthier lifestyle.

Section II: Transcript

- **NJCL: Alright, good afternoon, miss. Firstly, I would like to thank you for taking the time to speak with me today. My name is Chris, and I'm a NUS Year 4 nursing student. The aim of this interview is to get a better understanding on the perspective nurses have on self regulating their own health behaviors, and how various factors may influence this. I want to find out what are your views, opinions, and experiences on this. I would really like to encourage you speak what comes to mind. There are no right or wrong answers. May I have your permission to continue with this interview session?**
- P22: Yeah,
- **NJCL: Okay, thank you so much. Then let's start with some basic questions. Which ward are you working in right now?**
- P22: I'm working in an acute psychiatric ward. Yeah, in [Public Hospital name].
- **NJCL: Could I know more about like what you do on a daily basis?**
- P22: On a daily basis? It's a bit different from the general for example, medical or surgical wards. The staff to patient ratios are more of like 4 to 8 patients to around 4 nurses, with healthcare attendants la. Our responsibilities are less towards intensive bedside nursing care, ours is more observations, because psychiatry tend to be more on observing whether they have any psychotic symptoms or any low mood. We do also have to look out for patients who are suicidal when they do try to use various means to hurt themselves even with many factors removed from them. Yeah, we still do serve medications. We still do give injections. The junior roles. Junior nurses do take parameters. But environment tends to be noisier compared to the restructured hospitals because of there are patients who do shout because they cannot cope with their symptoms. There are patients who occasionally do get violent as well so we do have crises to manage these patients. We usually would have to restrain them and give them rapid tranquillizers to sedate them in order to manage their symptoms, and also to manage the rest of the ward who may be affected by these dangerous, aggressive, or violent patients. Yeah.
- **NJCL: Then, for you, how would you describe the workload. Is it manageable?**
- P22: Eh. I think every nurse will say that it's always too much. Even when I first started I thought it was too much. Then the workload got even heavier. So I think… while all of us are sort of adapting to this newer workload. There are limits. We do burn out quite a lot, especially in my ward. And the current culture is quite taxing in a sense, where we do have junior staff, who are not the most helpful, and they tend to take sick leave quite often. So it tends to burn out everyone else as well. On top of the changing profile of patients, the acuity may be… There are peak seasons, there are where there are more patients who tend to be more violent while suicidal, and intends to require more intensive care. Yeah, so there are there are multitude of factors that do contribute to the current workload, mostly due to patients and staff, staff attitudes, patient acuity, and also support from upper management
- **NJCL: When you mentioned that the workload got even heavier. So you’re saying that, when you started, it was lesser, but now it’s even more?**
- P22: So I joined right when we were in the middle of COVID. So we did have multiple outbreaks in the ward. and that required us to wear PPE. [NJCL: PPE: Personal Protective Equipment]. with no aircon or proper ventilation. But at that point in time, the consultant in charge tends to… the consultants didn't have much pressure to clear out the bits in view of the bed crunch. So because of the way our sort of reporting of, our documentation system, the workflow. The way it works. Because if we do have more long stay patients, we were required only to write a weekly report. So we had more of these long stay patients 2 years ago. And impatient length of stays were also a bit longer. partly because of the, partly because of the consultants. Like no pressure to clear beds. They themselves also tend to prefer for patients to stay a bit longer, while the rest of the MDT. Tries to arrange accommodations or like basically to ensure that patients don't readmit as often. Yeah. But right now, because there’s an additional consultant. So I started off with 2 consultants at the ward, looking after 48 patients. Now it's 3 consultants, also 48 patients. But the nursing stuff is still the same. So the number of reviews for patients in general is more frequent because there's more doctors to see so that also leads to a higher turnover rate, because with more reviews they eventually will decide that patience is well and discharged all up. Yeah. So more doctors, more turnover rates, more admissions, more discharges, or additionally, more work. And because of the higher turnover rate. In the shortest days, we would have to do more daily reports like, or at least reports per shift, like as per at the restructured hospitals. Yeah. Then, I think recently this year in particular, there were more cases with personality disorder. And they are generally not suitable for most wards, honestly speaking, like because they do tend to have a lot of chronic self harm behavior. And we have to prevent that. Although that is there, although for these patients this, this is usually their main coping mechanism. And but yeah. we need to usually have to keep really keep an eye out for them. And usually when this. A few, a few of these patients with personality disorder. They tend to get together, discuss ways to self harm or or ways to instigate other people to self harm. And yeah, so rather than just focusing on the reports. We also really have to be on the ground watching all of them making sure they don't do anything suspicious. Just yesterday there was one patient who peeled off the the wooden pieces off of the toilet door frame to use that as a sharp object to harm themselves. So we do come across incidents like this. and it is… Get very stressful, especially when they successfully hurt themselves. And then we have to go through the whole incident reporting. And you know, just raising, just alerting everyone that this incident happened, and to watch out so that they don't do it again.Yeah, so. There’s also pressure from upper management. Because for patients who are suicidal, a good majority of them tend to be those with personality, disorder. Upper management tries, or rather is advising us not to put patients on restraint when they are suicidal because there have been incidences where they have used the restraint to strangle themselves. So essentially, the nurses are put in between a rock and a hard place, because on one hand you need to keep them safe. But these people have all the time, 24/7, while in the ward to think about ways to harm themselves. Yeah. So it does really add on to the stress and burden of the nurses. Yeah.
- **NJCL: Then, just to clarify, is most of your experience in nursing in [Public Hospital name]? As in, did you start in the psychiatry ward?**
- P22: Yeah, I did start in [Public Hospital name].
- **NJCL: Oh. I see, I see. thank you for a very comprehensive context behind your workplace. Then my next series of questions will be asking more on lifestyle in general. So for you, as a nurse, how would you define a healthy lifestyle?**
- P22: Well, I will say you can just refer to the MoH guidelines [NJCL: MOH: Ministry of Health]. If you can exercise, ideally 3 to 4 times a week, 30 minutes. And eat healthy whenever you are. Okay, I don't have a proper word to describe, but only eat when your body is hungry, but at an appropriate level. Yes, of course, that's for the general population. Nursing wise. I think it's just really try your best all to meet their requirements, although it is not realistic. Yeah.
- **NJCL: Could you elaborate? Why, you think it's not realistic?**
- P22: I mean, for one thing, let’s say we start with diet.. Because normally, our whole circadian rhythm and like appetite, kind of cycle. Right now society is more of 3 meals a day. Maybe some snacks if you wanted to. And there's usually fixed timings lah. Not say fixed, but like it's a general kind of guidelines, let's say. or rough timing. So, let's say we eat breakfast and ate lunch at maybe 2 or like 1 o’clock, then dinner at 6 or 7. As a nurse it’s quite difficult to eat lunch at the same time as your patients. I think, especially in [Public Hospital cluster name],. The breaks tend to be much earlier compared to the regular mealtimes that every one is familiar with. At least in [Public Hospital name], mealtimes or rather breaktimes can be anywhere from 9:30 to maybe 11 plus maybe you'll be able to eat lunch at 12 or 1. If you are on a 12 hour shift. Or for afternoon shift. The break time is usually about usually can start after 3:30. You start work at 1.45 and your break is already at 3:30, and then you go home at 9:30 , or later. Yeah. And night shift also can be. At least for junior nurses or staff nurses. It can be from, let's say. 1 o’clock, 2 o’clock, maybe even 3 something. So it can be quite challenging, to have to to. To ignore your body signals. Your not hungry, and you still have to eat, or else you will get hungry later. And if you get hungry, And when you get hungry it's quite difficult to focus and awe, and if a crisis happens, then you don't have the energy. Blah blah blah. At least, personally, I try not to eat too much. Let’s say in a day shift, morning or afternoon, because I usually would have eaten something beforehand. In case I don’t manage to go for a break. But the thing is, because I eat light at work. But after work, I do get somewhat hungry, so I do end up eating after that. Nowadays, I am trying to push my post-AM dinner to the regular meal time.I do find it difficult to try and stay light on dinner, after afternoon shifts. Because there’s not much options in the night time. Yeah. So that’s for diet wise. Personally, I don't think I eat that much. It's just that because of the timings and the frequency of the irregular, meal times does tend to add a lot of stress to my own body. Yeah. Then, in terms of sleep, I think it's kind of expected that it also varies a lot, depending on the shift. For me. I don't. It's not too bad, because I still stay relatively nearby [Public Hospital name]. I don't really have to spend a lot of time travelling after work to get back home. Let's say, from afternoon to morning shifts. Hmm! Usually I'll I'll be able to get about 5 to 6 hours of sleep. If I can't fall asleep. Sometimes I go with only 2 hours. If I get anxious about work or something. Yeah. Then sometimes there is also night shift so it can be a struggle to slowly adapt your own body to night shift, especially if, lets say, you were on morning shift the day before. Although there’s like a good more than 24 hours to adapt your body. But it still takes a toll. A yeah. Then, I think recently. Recently we also launched a ‘flexi work scheme’ so that at least the staff has a choice as to how they want to work. So it was permanent morning, permanent afternoon, permanent night or permanent day shifts. The permanent night shift staff. They totally gave up after the 3 month trial. Then I think those in the day shifts. They are also kind of struggling, especially because. I think there was like a misunder- misconception that morning shift tends to be the busiest lah. But usually in the afternoon. That's where all the movements are happening, like admission, discharge, transfers. So there are people who eventually get tired of the routine and end up, reverting back to 2 shifts or 3 shifts. Even though this flexi scheme was supposed to be for the staff. The staff's convenience and potentially their well being. But eventually every one just reverted. Most most people reverted back to their original shifts. Yeah. Exercise wise, personally it is a challenge, I mean, if the whole day, in my case, if I have to deal with noise and a lot of patients who, let's say, require reassurance or explanations, or updates on the doctor's plans. and they don't really. Patients don't really see the nurses as people lah. It's only after, only the the long, the patients who have been in the system for a long time they will notice like, ‘oh, this staff hasn't actually gone off to eat. She's actually been sitting in the ward staying in with the patients the whole time’. There are patients who care. Yeah. But I think when you give your all while working in the in the ward. It's kind of challenging to switch your head space to like, ‘Oh, okay, I'm out of work. This is my personal time.’. Yeah. So, sometimes there is a constant worry of work. Sometimes you are too tired from working that you don't have the energy to exercise. Sometimes it's just not feasible to work, especially like, for example. sorry, not work. Not feasible to exercise, because let's say you if you are afternoon to morning shift, there's no time. Yeah. So personally I do, I still I still do try. But for my case it's more of a emotional slash, motivational kind of drive. It's usually when I'm facing ah, if I'm stressed out from, let's say, colleagues, or if I'm particularly angry about certain situations. I I do go for walks, or I do try to do some home workouts yeah. So yeah, like. For my case it's really more of a emotional aspect. Yeah. I do hear my colleagues, they do, some, one of them does like ClassPass and aerial yoga, and another one does like a kayaking. Another staff does like workout videos through an app. But I think, some of them are able to manage better than me lah.. Personally, I find that it's a bit challenging.
- **NJCL: Okay. I think I would like to clarify a few things. so earlier, when we’re talking about diet. is it common in your institution to actually forego break times?**
- P22: In fact, it's not, eh. I think when I first joined. There were staff who would, really enforce force like at least go for 45 minutes, at least go and have a meal, at least eat something. Yeah, I know my friends who are in other institutions, in other general hospitals. They won't be able to eat at all ah. But in [Public hospital name], I'm not so sure if it's because of my own ward culture. Yeah, but because of the way that we document things. I think this is another Pandora's box altogether, because my ward staff tend to be a bit more strict in terms of documentation, and like the medications and patient safety and all. So every one in the ward, or at least those who care, are quite obsessed with not making a mistake. and in order not to make a mistake, the best way is to just be in the ward. Of course you can delegate your tasks and duties to the other staff who will be around with you on the same shift. But I think depends on individual rapport as well. Yeah. But breaks wise, I think a good big percent of a time, we are able to go. It's only when there's. It's usually when it's really chaotic in the ward, or if maybe, let's say, it's Monday or Friday, when all the reviews are stacked up for the doctors. So they would likely they usually tend to push for discharges towards the end of the week. So that's usually when this there’s a lot of movement and it's quite challenging to go for breaks, because you can't tell the staff who brought the patient there like, ‘okay, wait. I need to go for a break first. Can you like, wait there?’ Yeah. So I think it's a lot of sort of many unforeseen circumstances like that that usually leads to nurses not having to take a break. Yeah. right. I mean, let's say. I mean, admission, discharges, transfers, that's one thing. Sometimes. In other wards, or when the E-room calls beforehand, we still can plan beforehand. But not everything goes to plan all the time. Sometimes admissions can be delayed an hour. There was one time it was delayed by 4 hours. Sometimes the ward situation is quite challenging. Like, let's say, there is not enough, staff or not enough experienced staff would be in the ward. If lets say someone takes a break which also poses a challenge ah. Especially during mealtimes or medication times. Usually, it’s all hands on deck. Really depends ah.
- **NJCL: Okay, then, just to briefly summarise what you've said for this. So for your ward, your ward specifically. Because of how strict the ward stuff is regarding medication and patient safety, break times for the nurses are not always followed? Sometimes you say like maybe on a Friday where there is a lot of movement, it’s a bit busier, they don’t tend to go for breaks, the nurses. Is that correct?**
- P22: Yeah, something like that. Because, like, even the ward next door, their handover is so short and concise. They are able to leave, they are all able to head into the staff pantry like 10 minutes before the end of work timing. Like they really have the time to pack their things, change their clothes and then clock out, exactly at lets say, 9:30, after afternoon shift. Our ward, our ward would leave exactly at 9.30. So I think it’s just different ward practices. Yeah. Yeah, difference in culture lah.
- **NJCL: So is it right for me to say, that the main reason, maybe the nurses aren't adhering to their meal times, for your ward is basically the ward culture?**
- P22: Yeah, it's, it's a pretty big factor. Yeah.
- **NJCL: Okay, okay. Still on diet. Earlier, you were also mentioning, like, you find it difficult to actually remain eating light meals, because you mentioned that you will eat meals during your breaks, but you find it difficult having irregular meals adds stress. With this in mind, what are your main motivations in changing your own dietary habits, currently?**
- P22: It's really just weight and self-esteem and how I look in the mirror. Yeah, I mean. of course, I try a bit harder when let's say, oh, the pants is getting a bit too tight. Maybe I should just cut down on junk food for for a bit or go and walk a bit more now. Yeah. But also these motivational factors are also weighed against the, I think, my overall mood. If I don't end the shift, well, then, usually there's a lot of anxiety, or, let's say, a lot of reflection on what I can do better. Or, in other words, a slightly low mood. So usually in these cases self care tends to get thrown out of the window.
- **NJCL: Then earlier, just to clarify you mentioned that your usual hours asleep is 5 to 6 ,but there are times when you only have 2 hours of sleep?**
- P22: Oh, no, no! That one is specific for afternoon morning shift. Because I think, especially in my first year of work, the staff that was still around back then, they had this habit of broadcasting everything into the group chat. So if somebody missed out something they would tag you, and then they would ask the whole group like, ‘oh, why is this medicine still in the medicine trolley, or like do you do this? Do you do that?’ So, for the first year of work, there’s always this impending anxiety, like, maybe I missed out something, or maybe I didn't do something that I should have. Yeah. So those thoughts tend to worm into me, especially in the first year. So that usually can cause a lack of sleep. Nowadays. It's a bit better because I’m able to sleep a bit longer compared to before. If I really can’t manage, I’ll probably just take some sleep supplements, so I do have my workarounds. But 2 hours is quite rare nowadays. As in like per sleep per night. It’s usually more 5 to 6 hours for afternoon and morning, otherwise, it can be 10 to 12, depending on how tired I am.
- **NJCL: Then earlier, regarding the the shiftwork and the flexi hours you mentioned. So you said that most people actually went back to the rotating kind of shift work. Then for you, personally in your opinion, is the rotating shift hours better than the fixed all morning, all afternoon or all night shifts?**
- P22: I think it's good to have a mix of both. Because if you are on the same shift, you are able to notice patterns, at a fixed timing, especially for psychiatry patients. And let's say, like, I do have this colleague who is on permanent morning. So she she usually helps up with showering patients, so she would know if there's a trend like, ‘oh, this patient actually has been refusing to shower for the past few days’, or she she's been refusing lunch or she has this peculiar behavior. So sometimes have having staff who are on permanent shifts is good because they can observe this trend. But, for operational efficiency, I would say, we also need rotating shifts, because usually it's, usually those who are doing afternoon and morning shift. They are the ones who would be sitting in on the doctor's rounds in the morning. So you definitely need, both both afternoon and morning shifts to really observe and document any peculiar behaviors. So this is more of almost 24 hour kind of observation kind of thing on the nursing end, because we also do provide feedback to the psychiatrists on specific behaviors lah. Yeah. I think it's also quite vexing for those on the rotating shifts, because there are times when people get called back to cover night duty because somebody MC or there's essentially lesser stuff to cover night shifts since there's more in the day. But I think my ward is still figuring things out. We are kind of just rolling with you, just dealing with whatever we have. Yeah.
- **NJCL: Then, personally for you, is there a preference? Would you prefer? Permanent or rotating shifts?**
- P22: I think. Right now I prefer rotating shifts mostly because I still want to retain my skills and remember the workflows, because even just 2 weeks away from the ward, and sometimes I may even forget certain like, let's say, night shift, if you’re on night duty on a specific Saturday, you need to check the E-trolley. Like these kinds of little things, I tend to forget quite easily if I'm not regularly doing it, so I would prefer to be on rotating shifts. Now, I am also considering to quit eventually. But, that one is probably in the next 1 to 5 years. So see how.
- **NJCL: Okay. Then, moving on to when you were mentioning about exercise, you mentioned that for you, you mentioned something about it’s an emotional motivational kind of drive to get you to exercise. Is that correct?**
- P22: Yeah. Because, personally, is usually when I have more stronger emotions. Like let's say, ‘oh, this colleague pissed me off at work. I really wanna punch her face’, but I can't. So I would just exercise or like let's say, okay, the number on the scale isn't particularly appealing, you know. Personally, I tend to be driven more by negative emotions, because exercise also is a stress reliever. Yeah.
- **NJCL: So for you in terms of exercise. Your main motivators are mostly on your negative emotions?**
- P22: It’s mostly to relieve stress.
- **NJCL: Are there other stress relievers that you use to cope with stress?**
- P22: Hmm. Talking to friends or like, sometimes there's also the occasional bingeing due to work stresses that. But that one I try to keep to a minimum. I think I think it's also recently bubble tea has been a stress reliever as well. But I am trying to cut down that in view of that recent news article. About bubble tea correlating to poor coping strategy, yeah. Not sure if it was last week or two weeks ago.
- **NJCL: Hmm, okay, then, lastly, on physical exercise. You were mentioning that some of your colleagues actually go for aerial yoga, hiking, workout videos through apps, all these kind of things. You also made a comment on, like ‘some people are able to manage better than me’. Could you maybe elaborate on that right? Maybe suggest why? Why that is so?**
- P22: Well, maybe it's maybe it's a general attitude towards work, or could be even burnout. Yeah. Or maybe time management. Yeah. I never really asked them per se on how they managed but… I mean because for me, I am more of a couch potato. So I think it's more of my existing lifestyle even before joining the workforce. Yeah. As for my colleagues, I think they also do have, I think some of them have friend groups that they go and exercise with ah, yeah. For myself, I think because most of my friends are from other institutions, or they are working office hours, or it's quite difficult to coordinate lah. Hmm! So usually I would end up exercising by myself. So there's no other external not to say forces, but drive to get me to exercise.
- **NJCL: Could you maybe elaborate on how, how do your lifestyle change after you started to work as nurse?**
- P22: I think I’ve just become less physically active. Yeah, because I was in hall, there were a lot of activities to try like sports or other cultural activities. Like there’s a lot of CCAs as well. I think the convenience of having these options, got me to exercise a fair bit. But after working, it’s a challenge because there’s so much to do. The gym in [Public hospital name] is kind of small, and no one really goes there so it’s a bit scary. Ya, I think the culture wise, the upper management is also trying having zoom yoga and stuff like that. But it usually happens during handover time. So it’s catered towards the office workers. I think diet wise also, I think previously I was more disciplined in maintaining my diet, but now because of work, the threshold for me to go eat junk food is much lower because I use it to relieve stress as well. Hmm. Sleep is of course disrupted, from having straight 8 hours of sleep every night, now it’s 5 to 6 on some days, 10 to 12 for other days, sometimes no sleep because I am preparing for night shift. But overall, I think it’s really just the fact that there is not much options to do in [Public hospital name] for exercise. At least I know in [Public Hospital cluster], there are, the gyms look fancier, ya, the [Public Hospital name] like not bad leh, then they still Anytime Fitness gym there also. As in, it’s outside. As in there is a inhouse gym staff gym, and then there is also Anytime Fitness gym for the general public. So the staff there still can choose where they want to go to if they have the time or energy to go there. But for [Public hospital name] wise, I think it’s just the physical aspect of the medical part, but no one really goes there. Yeah.
- **NJCL: Yeah. I think so far, you covered like, mostly physical health in terms of like exercise diet. Do you do anything to self-regulate your emotional or mental health? Specifically?**
- P22: Mental health? Usually I do confide in my partner or my friends when something happens. Otherwise my family as well. On days when it gets really bad, I usually go out for walks because it does help me clear my mind up. Sometimes I do try to occupy myself with other activities, so right now, I am attending a few courses to have some variety, like in a sense away from the usual routine work in the ward. Although it's more, I'm just convincing myself that it's preparation so that I'll be able to find a job and update my resume by the time I do quit.
- **NJCL: Then does spiritual aspect play anywhere for your lifestyle?**
- P22: Not really. I'm not very religious, but once in a while I try to meditate, but I think my attention span has gotten much shorter as well after starting work.
- **NJCL: Alright. Okay. And then my next question, I think you've answered bits and pieces. Yeah. But what are your main facilitators or motivators in self regulating a healthier lifestyle?**
- P22: Well. I think I was saying earlier, like self esteem, like, if I think, eating well, exercising well, sleeping. Well, it does have many positive impacts on like how you feel and like energy levels as well. There are compounding effects. But I think is a challenge to get on the wagon to start doing it. So, motivation, I think it's really just if I need to relieve stress or if I find that. I suppose it's a bit of like a body dysmorphia, you know like those guys in a gym, if I don't find like if I, if I, dislike the way I look in the mirror, then I will be slightly more driven to maybe take a walk. Or like, if let's say sometimes, after eating junk food and in like here's always that food coma, the fatigue after that. And I'll be like, Okay, I think I'll need to try and eat a bit healthier next time. Yeah. Sleep wise. It's usually when I wake up and I feel really zombified. So yeah, it's an innate drive to want to sleep after after work.
- **NJCL: Then moving away from facilitators or motivators. What do you think are your main barriers in self, regulating a healthier lifestyle?**
- P22: Hmmm. I think it's more of accessibility in terms of options. Because.. I mean, my place is just opposite a park connector, so I can walk if I want to. But other facilities like, let's say gyms, or like those yoga places, or. you know, like. Because most of these facilities, they are located in the central area. So to have to travel. to go out my way to, let's say, travel and do the… Like. for example, kick boxing. It can be a hassle, especially when I am so used to walking to and from work. Rather than to take the train. It's not something like I can just stop by on the way back home, or something like that. Yeah. As for apps-wise, usually, like those exercise apps. In the case of classpass, it’s better to go for friends. Because, personally, I am quite a shy person, so I do not want to go these classes by myself. So, having to coordinate with friends is also quite an issue.If its those workout apps, I’ll usually forget about those in the first month, so I usually stay away from these type of apps.Other factors. Hmm.I can't think of any right now.
- **NJCL: Okay, then, I'll just briefly summarize what you shared with me. So regarding like facilitator slash motivators for self regulating a healthier lifestyle. For you. It's mostly because of self-esteem, like. Maybe you don't like how you look in the mirror, or maybe you ate some junk food. Then it sorts of motivates you to eat healthier. Then there’s also the positive health effects that you mentioned like, oh actually having regular 8 hours sleep, having exercise, that there will be like compounding beneficial effects to you. Then, lastly, it's also like a form of stress relief. So you're motivated to do these activities because it's it helps you to relieve your stress.: Is this correct so far? Is there anything you want to add for facilitators?**
- P22: I suppose. Previously it used to be a family bonding experience, but my family members now, we are all doing our own things, so it's not really a motivator right now. But it used to be.
- **NJCL: Hmm. Like having other people like join you to do this kind of activities, is it?**
- P22: Yeah, yeah, like a social aspect of it.
- **NJCL:Okay, then, for barriers, you shared with me that the main issues is accessibility. Most of the facilities that organize this kind of activities are very far away from you and it’s not on the way home kind of thing, so it’s going to be quite a hassle. Then, also having friends who can come with you is a bit difficult, because coordinating this kind of thing is difficult due to difference in schedules. Are there anythings you want to add for barriers other than these 2 main things?**
- P22: I think time and energy levels ah.If let's say, I am on a 12 hour long shift, I definitely won’t have the energy to do it. Or if I am afternoon to morning, then there’s definitely no time to exercise, unless I compromise on sleep.
- **NJCL: Alright. Then my next question would be, how do you think your personal self-regulation habits impact your work performance?**
- P22: Oh, it's a very I would. I would think it's quite obvious lah, in a sense. Sleep wise, I would definite- my mood would definitely be much worse if lets say I don’t sleep enough. And sometimes I do unintentionally take it out of on my patients which kind of ruins my rapport. Yeah, so it definitely it definitely does have an impact. Diet wise, there is no direct impact per se. Because at the very most, my patient would just comment that I've gotten fatter. And that's it. It's more of maybe affecting my overall energy levels. Likewise with exercise. Yeah, because I think, after gaining lots of weight after starting to work. There is a certain like a brain fog per se. Like mental clarity, isn't quite there compared to before lah.
- **NJCL: Okay, then. So far, you shared like how it affects work performance. Do you think it also affects your life outside of work?**
- P22: Oh, yeah, it does. Because: I think right now, because of low energy, right? I couldn't exercise. So most of the things that I do is either like sleeping or using my phone, which is- sleep is fine. But I think because using my phone, there’s the whole dopamine drive, like the the dopamine rush, so I won’t say it’s the most productive. And it does eventually affect my mental health.
- **NJCL: Then, just clarify what you mentioned earlier. You said like for apps, exercise kind of apps, you tend not to really utilize them. But do you use any other like tools to personally, self-regulate your lifestyle?**
- P22: Anything else to regulate?
- **NJCL: Like, maybe to track your calories? This kind of thing.**
- P22: I used to journal, but that was before I started work. After I started. I totally just dropped the habit. Tracking calories also happened during uni, but I think I got too obsessive with the numbers, so I did lose weight, but it was very difficult to maintain. Yeah, so I, I did briefly try tracking my calories in, I think, a few months ago. But I think with the limited options of food as well, it was also not very helpful. because I was more or less eating the same thing. Yeah. Because a good majority of my food at work comes from 7/11.
- **NJCL: The next few questions, a few more, will be regarding the workplace in general. So I think this question you also sort of answered. But as a nurse, how do you think the workplace influences your self regulation habits?**
- P22: I think, with the whole culture, like the people you work with are essentially. like, let's say, If your colleagues are not…Like if there's no teamwork, and you feel like you have to take on the whole burden of the shift yourself. Like, if you do feel like, it's like your other colleagues are not doing enough to the point where you have to start micromanaging them, or if you try to give feedback to them, they start arguing back… Then like, I think in those examples, they do tend to add a lot of stress to a point where sometimes I do feel overwhelmed that when I go home I just don't want to do anything. I just need to decompress, like I just need like a silent room. Just knock out for a while. Compared to if, let's say, on on a good shift where my colleagues were helpful, they, let’s say, like I helped them do certain tasks and they cover for me later on in the shift. They, they are proactive, they tell me, when something seems odd and we can rectify the issue early, such that nothing went wrong in the shift. Those are the days when I still have energy to let say, I will still be in a decent state of mind to decide like, okay, it's a good time for work or to go and exercise at the gym or something like that.
- **NJCL: Okay, since we are talking about the social aspect of the workplace like the workplace culture among nurses,. are there instances where the workplace culture actually facilitates self-regulation of healthy lifestyles?**
- P22: I think I was mentioning earlier, like there are like online weekly Yoga sessions. But for my wards specifically, I know there are like one or 2 colleagues who do exercise together. But as a whole, I wouldn't say there’s much effort to encourage a healthier lifestyle. The only other thing I can think of is a the yearly screening. But I think every other hospital has it for this staff? Yeah.
- **NJCL: So is it right for me to say, your workplace culture, specifically in your ward, It's more of a hindrance or like a barrier than a facilitator in helping the nurses like self-regulate their lifestyles?**
- P22: Yeah, yeah. I mean, okay. Now that you know mentioned this, I think having good leadership also helps. Yeah. Because recently there was a change. there was a swap in managers from- My manager went to a different ward, and their ward sent one over. Yeah, it's good to have managers that try to support you in the way that you do your work rather than to have, let's say a so called micromanager. I think, for this particular manager, the newer one. She has certain practices that ward staff don't really agree with. Yeah. So for example, the previous manager, if she was on morning shift she would also help out to bring those ADLs-assisted patients to shower. This new manager. She either doesn't step into the dom- the the ward. The dormitory at all to help the shower, or she stands around and she does a very small bit of bedmaking or patient safety that she calls it, without actually helping to get the task done. So I think, such actions are usually quite, obvious ah. Especially when there is a direct person you can compare against, in terms of leadership styles and how they run things. So when there's lack of support from your immediate supervisor, it's also another challenge. Because when you don't feel supported, you don't feel like going to work, and when you don't feel like going to work, the easiest way is to take MC. And when you take MC, someone has to cover you. And who is the one that is usually asking to cover? It's usually the night staff or the supervisor. Yeah. so this supervisor tends to ask quite often whether I can come back on this day to work or that day to work which kind of impinges on the work-life balance that I try to have because she will usually message me outside of office hours, even though let's say we were on the same shift the previous day. And she could have just walked over to ask me, you know. So yeah, a lot of social factors that can contribute to individual nurses’ mental health, which then has a subsequent domino effect on diet, steep, lifestyle. Yeah.
- **NJCL: Okay, then, on the topic of nurse managers or supervisors. Do you think the nurse managers are role models for healthier lifestyles for the ground nurses?**
- P22: The one that is retiring soon, yes, because she does mention that she exercises. The one that transferred out, her only exercise is running up the stairs to attend crises. So I wouldn't say they're the best role models. Although they they they do try. But they're not like fitspos, you know. So I don't really think they motivate the ward staff to exercise, especially. Oh, yeah, the yearly ward bonding is also a buffet. [Participant laughs here]. So, that's not very encouraging to maintain healthy lifestyles.
- **NJCL: But for you personally, do you believe, like they should do more to encourage healthier lifestyles? Do you think it should be a part of their work?**
- P22: They should you get the roster right before they ‘let's try to do other initiatives’ first. I think they also have a lot on their plate. Yeah, because there's a lot of work groups that managers as well as the staff participate in. So additional CCAs to take up more time outside outside of work that could have spend on doing something else like maintaining a healthy lifestyle. They can, they can. It’s something they can consider, but I think there are more immediate fires to put out before this.
- **NJCL: Then moving to iinstitution wise. How do you think your institution can help you to self regulate a healthier lifestyle?**
- P22: They should subsidise gym memberships at the nicer gyms Apparently [Public hospital cluster name] has discount for virginactive. I'm not so sure about [Public hospital cluster name]. I think healthier lifestyle wise, in fact for some of the other wards, like the long stay or rehab wards. The the word nurses are the ones that are actually leading the exercises among the patients. Like, simple. Something like you will see in the park. Like some simple stretches, exercises, sometimes maybe even Zumba. But those are for the less acute patients. Yeah. So I think as an institution, there are efforts seen through the online yoga. Getting the staff to exercise with the patients. Bringing those patients who are in slow-stream rehab. Sometimes the the staff do bring patients out for walks as well and if they have to bring the patient's out for walks, they also have to walk. So, there’s that. But I think, because of the nature of acute wards in my building, it can be a challenge to promote such activities. Unless, they reduce the number of beds, or increase the number of staff. But of course this is what upper management has difficulties to resolve. So as an institution, they are trying. Just that, it hasn’t reached my building yet.
- **NJCL: For you personally, would you say that like maintaining a healthier lifestyle. The onus is mostly on the individual, or the management?**
- P22: It's mostly by the individual, but management can also do their part to support staff to maintain healthier lifestyles if they want to increase staff retention because if you have. if you have a healthier lifestyle, it also suggests that you have better work life balance, which is also one of the driving forces that can make or break staff and influence their decision to stay or to quit. It's like a 70:30 kind of aspect.
- **NJCL: Hmm. So for you right now. you would say, would you say that management has done enough for this staff?**
- P22: No. They should subsidize the gym membership! You know, like, how in NUS. I think if you show like your student pass, you can get certain discounts and stuff like that. At least for the alumni, there are certain discounts. I haven’t seen any of that in [Public hospital name]. At the very most, there’s only those corporate discounts for mobile phones. But not related to exercise or healthier meals or tools to improve your sleep, or supplements. So… sorry, I forgot your question.
- **NJCL: It was regarding whether the the institution management has actually done enough for the staff.**
- P22: Yeah. My answer is still no, ah.
- **NJCL: Okay? Then, final question is, are there workplace health promotion programs in place?**
- P22: Yeah, the online yoga. Then other wards, the do the morning exercises, along with the patients as I was saying earlier. That's about it. So far I can't remember anything else.
- **NJCL: So, I'll just summarize briefly what you shared with me. So on my question about the nurse managers being role models for healthier lifestyles. You say that they're not really the best. I mean, they try but it’s not enough. Then you also mentioned that they should focus more on the rostering first before they even try any of these initiatives.**
- P22: I mean, it's more like they have other immediate concerns. More immediate and urgent and important concerns that they need to focus on before they can even do this supplementary kind of initiatives.
- **NJCL: Then, on my question, on, how do you think your institution can help you to self-regulate a healthier lifestyle. You mentioned that the institution should subsidize gym memberships. You also mentioned that they are, there’s some effort that is seen. But maybe for the nature of your specific ward, it is not as observable. Then you you also mentioned like they can also, maybe reduce the number of beds or increase the number of staff but the upper management is also having difficulty with that. Then, the follow up question on whether the individual or the management plays a part in maintaining health of the nurses, you said that it's a 70% to 30% kind of ratio. Then, lastly, whether upper management has done enough, you send a firm no. You don't really see like- like maybe compared to NUS alumni, the kind of like incentives they have, they don't really have in your institution right now. Then, for workplace health promotion programs. You mentioned the online yoga and actually like doing morning exercises together with the patients. And you can't really remember anything else. Is that right so far? Is there? Is there anything you would like to add for this portion?**
- P22: I think that's about it.
- **NJCL:Then do you have anything else that you would like to speak about that was not mentioned in the interview? Any further comments you want to share?**
- P22: Hmm. [Long pause]. I think maybe on what upper management can do to support staff. They should also choose the vendors. You know, in the [Public hospital name] canteen, the fool is so horrible. There was one time on the workplace… Basically it's like, Facebook but for the [Public hospital name]. Someone posted a photo of rotting vegetables in the Yong Tau Foo stall. So at least I think, upper management should at least screen the vendors. Those who are more credible, or those who can provide healthier choices. Rather than what is being served now. Literally because there is one stall that is selling everything, everything in that stall is literally fried. Fried noodles, fried rice, fried chicken nuggets, fried egg, you know? They can influence the things- the items on the menu if they wanted to. Or at least get someone to serve healthier options. But ya, I think that’s about it. I have nothing else to add.
- **NJCL: Yeah, so like getting more accessible, healthier options in the institution?**
- P22: Yeah.
- **NJCL: Okay can. Alright. Then, if there's nothing else, this will mark the end of the interview. Thank you for your time and participation.**
- P22:Alright. Thank you. Sorry for dragging that out for so long.
- **NJCL: It's okay. It's okay. I'll stop the recording.**
- -End of P22 transcript-

# **Interview #23**

Participant and interview details
 Participant number: 23 (P23)
 Date of interview: 29 Oct 2023
 Interviewer Number: Navarro John Christopher Lambino (NJCL)
 Time IDI started: 1100
 Time IDI ended: 1200
 Observational notes: Participant seems well-rested.

Section I: Interviewer’s summary
 The individual interview was conducted via Zoom. For context, P23 is a nurse working in a public hospital emergency department for 3 years, straight from graduation. She is currently on hospital leave after a sports-related injury. There were no significant interruptions except P23 earpieces losing battery during the interview, where in the end she just turned on her computer audio. All her responses have been mentioned by previous participants. From P23’s responses, she is managing well, and still lives an active lifestyle. P23 has given straight to the point answers that require some prompting. She mentions that self-regulating will require actively planning to make full use of the time or else remain drained out by the situation. There is also an emphasis on people having activities outside of work and how they might have outside circumstances (taking care of family) that may hinder their ability for self-regulation. Firstly, she mentioned the importance of work-life balance. She also said that her workplace culture is both a facilitator and barrier to healthy lifestyles as they would both positively and negatively influence her lifestyle. There is also an emphasis on people being set on their ways that is difficult to change. Notably, nearing the end of the interview, I decided to ask additional questions regarding nurses as role models for patients to supplement what another participant have mentioned, as a form of constant comparison analysis. All in all, the interview went smoothly and perhaps due to her responses being very similar to the past participants, I did not prompt her more which may impact how much more she could have elaborated.

Section II: Transcript

- **NJCL: Okay. Good morning, miss. Firstly, I would like to thank you for taking the time to speak with me today. My name is Chris, and I'm a NUS Year 4 nursing student. The aim of this interviews is to get a better understanding on the perspectives nurses have on self regulating their own health behaviors, and how various factors may influence this. I want to find out what are your views, opinions, and experiences. I would really like to encourage you to speak what comes to mind. There are no right or wrong answers, may I have your permission to continue with this interview session?**
- P23: Yeah.
- **NJCL: Okay. Then let's start with some basic questions like, which ward are you working in right now?**
- P23: I'm currently in [Public hospital name] emergency.
- **NJCL: Could you tell me, like, what kind of what you have to do on a daily basis?**
- P23: Mainly a lot on symptom management and so whenever they come in, the nurses will be the one doing the triaging. Then we have to prioritize them according to the severity level. Then subsequently after doctor consultation, then the doctor probably might order some of the medicine or if there’s some procedures for the nurses to carry out, then we will just carry out from there. We will probably observe them for a period of time. If they are able to be discharged from the hospital, then they are sent back home. If they require more observation, then we will advise them to be warded for further observation. And also, it’s emergency department, so if the patient is very very severe or critically ill, then we bring them to the resus room then we will do all the resuscitative, required procedures
- **NJCL: Hmm. Could you maybe describe the workload? Is it manageable for you?**
- P23: The workflow? Whether the workflow is manageable for me?
- **NJCL: Yeah, and the workload.**
- P23: Mmm. I think so far it’s still manageable. I think maybe because I just entered this whole clinical ground. I’m still new, started about 3 years only. So, I personally like the adrenaline rush. So I’m okay with the amount of workload and things we have to do and plus in emergency department, we don't really have a lot of admin work to do. So we’re really task orientated and we just focus on whatever is needed and focus on patient care, and to really see the transition from a sick patient to a very well patient afterwards. Yeah. So workload wise, and also because we’re in emergency, we are very teamwork based. Because everyone can do each other’s job. So we don’t have to feel like we have to stay back beyond our working hours just to make sure there’s a good transition of care. Because after that, someone can just take over and read through the notes, then carry out the orders from there. So in terms of workload, I think if there’s a huge crowd in the emergency department, its definitely taxing. But overall because there's a huge team that is able to help you whenever you need help, then it's not not too bad afterwards.
- **NJCL: So to verify a bit, it’s very common in your ward to actually end on time?**
- P23: Yeah, they do. Quite common.
- **NJCL: Then another clarification is, right after your graduation, you went straight into emergency. Or?**
- P23: Yeah, I went straight to the emergency after graduation.
- **NJCL: So 3 years in emergency, right now?**
- P23: Hmm. [Participant nods head]
- **NJCL: Okay, then, my next series of questions will be regarding lifestyle in general. So for you, as a nurse, how would you define a healthy lifestyle?**
- P23: Definitely work-life balance. Because with our work, we are very shift schedule basis. So we need to learn how to find, find the external things to, external hobbies, to like occupy us outside of our shift work timing, and because sometimes our off days will be like on very random days, right? Not like every office worker on the weekends, but for us, our workdays can be on the weekends. And also can be early in the morning or from morning to the afternoon before the night shift. So we have to find all these pockets of off days to find, find out how to make use of the time. You can use it easy to sleep actually, sleep is also a very good way of resting. Yeah. But for me it's either I'll be resting, or I'll be doing other sports.
- **NJCL: Since you mentioned something on sports, so specifically on your physical activity. How do you feel about your current lifestyle. Do you feel like changing it?**
- P23: So I actually quite like what I'm able to do with my shift work. Because like, I mentioned learning tennis on the go. And for tennis wise, Initially, I felt a bit restrained, because if I want to learn tennis, it’s usually on the weekends. And I won’t be able to do that because of shift work, right? It's like it's never guaranteed. But with my shift work and if I'm able to make some compromises; like for example, I go for one-for-one coaching for tennis lessons, then I am able to schedule the time period. And most of the time the tennis coach will be freer in the morning. Which is also better for for me, because I'm also free in the morning especially after night shift or on off day. Yeah. So that's how I find or how I try to better incorporate work-life balance. Yeah. But unfortunately, I recently sustained a sport injury that is why I’m on HL [NJCL: HL: Hospital leave] Yeah. So so that's why I'm off sports for a while, but also a time for me to rest I guess
- **NJCL: So would you say your current lifestyle is very active, before your injury?**
- P23: Hmm, mm. Quite active.
- **NJCL: Then, moving away from physical activity, how much? Specifically on diet. How do you feel about your current dietary habits?**
- P23: Oh, hmm!: I think it's okay. I don't think I am very unhealthy. But I'm not in a very conscious effort to be healthy, very, very healthy, either. So I mean, especially when you're doing night shift. And then sometimes the senior doctor may order some supper. And stuff right,and then you have very irregular eating timings. And that can be quite detrimental to health, in the long run. Now, you won't see it, but probably in the future you probably will see the effects of it. And also because our eating timing during our shift is very irregular, and also for emergency, we have this mindset of just eat whenever you can because you will never know when the next flux of patients will come. So we usually send our colleagues out for breaks very very early. Like for morning shifts, we have to go for breaks at like 8-9am, and then we have to sustain until 2-3pm until we hand over to the next shift. That is because that is the timing we think can send our colleagues to go for break. Because from 9am to 10am onwards, that's when the huge crowd will come in. So we'll try our best to send our colleagues very very early. But if you think about it, eating at 8 o’clock, and sustaining until 2 to 3 pm is also not very good.Yeah. And it's how the emergency department works, I guess. Hmm!
- **NJCL: Earlier, you mentioned you're not making conscious effort to eat healthier. Could you maybe elaborate on this and why?**
- P23: Because I personally really like eating. So just the thought of eating just plainly greens or salads or organic food, then I’m not very enticed to it. But, I have been eating less sugar stuff. As in, just cut off what I prefer, because I don’t really like super sweet stuff. [Inaudible segment here]. But, if you're thinking, if you're talking about white rice or brown rice? Then no lah, I just eat whatever is there. Yeah. As long as it can sustain me then I’m okay already.
- **NJCL: Okay. What do you think will make you want to eat healthier?**
- P23: What would make me want to eat healthier? Hmm. [Long pause]. If the healthier food is nice, and its cheaper. I guess incentive to make us want to eat like healthier. I think that’s it actually.
- **NJCL: Okay, so far, what you'd be sharing with me is like the physical aspect of health, like diet, physical activity. Specifically, on maybe, like the emotional or mental aspect of your health. How do you feel about your current lifestyle?**
- P23: Hmm! The emotional aspect, ah? Hmm! I think it can be very tiring, especially after night shift. And also when. Because of shift work, we don't really have regular sleeping hours. Yeah, I think, as a nurse, you also know like the PM to AM transition. And then, if, like night shift. Yeah. And then. And often, night shift we have to regulate our body clock, and as much as you want to have a, a good sufficient amount of rest, we have to sleep in a way that will regulate our body clock to prepare for our upcoming shift again. Then after post night, what my nursing friends will recommend is to not sleep too long after night shift and force yourself to wake up at like 1pm, so you can sleep at like 10pm at night. So the next time you wake up, you have the normal cicardian rhythm and timing again. Yeah. Because if you sleep too much in one go, then your body will be not adjusted to the new; the amount of sleep you are getting. I think because of the lack of sleep, it will definitely have some impact on your emotional and tiredness level. But so far, I think. I feel like I’m still regulating it okay. Like I’m still able to plan off days for myself or off days to meet up with friends. So if I feel like I’m not in the mental capacity to meet or talk to people, I will just take my time off at home. And also with sports, then I’ll be able to go for sports then it will also make me feel better.
- **NJCL: Hm.. . Does spirituality play any part in your lifestyle?**
- P23: Spirituality… Personally, I don’t really have a religion. So, I don't really go for, like church or temple praying, or for those.
- **NJCL: Okay. Can. My next question, I think you did answer bits and pieces here. But for you, as a nurse, how do you feel about self-regulating a healthy lifestyle?**
- P23: I think it's very important. Because with our shift work, and with our schedule, it's very easy to just think that our life is purely on work. And for our work, we are on a service basis, right? So we are always talking and serving people, so it can be very draining on your emotional health. And if you are too complacent in letting work take over you, right, then it might just drain a lot of your energy. Ya, so you need to be consciously active to seek work-life balance. It doesn’t have to be active lifestyle like me, but you can always have your own way of finding what you like to do outside of work. Plus our work is very appointment basis, so if you want to meet up with friends, you also have to make appointments in advance, because you will never know when is your next shift, or whether you can get the next weekend off for example, because we have a rostering system, right? Which makes it very hard for us to plan in advance or do spontaneous trips. I mean that’s the downside. Even when balloting for AL. [NJCL: AL: Annual leave], like annual leave. We have to plan 1 year in advance. But for others, in other industries, they can do it anytime. And even if you want to ballot for AL, you can’t even guarantee that we get it. Because there are so many people who might be vying for the same slot. So, I think because of all these systems, and the rigid regimen that we have to follow, then all the more it is important to be able to segregate ourselves from work. Because, it’s very easy to just fall into the hole, the system. And just be stunned inside it and you just feel very very drained every single day.
- **NJCL: Could you maybe share with me, how did your lifestyle change after you started to work as a nurse? If there is any change?**
- P23: How my lifestyle change? Hmm.I think back then, when I was in, I was very active in like, in frisbee. I was in a frisbee club. And because frisbee is on like the weekend right, then I can go there very very regularly. But now, the moment shift work started, I found it very hard to commit to every weekend schedule. There were times, I had to go for frisbee training, and then head for night shift at the end of the day, which is like ‘oh my god, why am succumbing myself into this tiredness’. Ya, and then at one point I just asked myself if I should just let go of this commitment and then just focus on something else instead. And then back then when I just started, my work, it was during COVID. So I was the COVID batch, and that took away frisbee. So it made the decision for me to not even have team sports to begin with, and that’s why I ventured out into tennis from there. I guess that’s how my lifestyle changed. Maybe there are some commitments that I won’t be able to commit to compared to when I was just in uni. Uni you just had Monday to Friday right? Now, every week is different. Like you’ll never know when is your next off day. And also apart from frisbee. I think it also affects how you’re able to meet up with family and friends. So there are times when my family would like to meet on a certain weekends like Saturday and Sunday because that’s the best day for them. But because of the shift work, I won’t be able to go. And then maybe on public holiday when everyone is available to go, like for sure they have a public holiday off. But for nurses, we don’t have that. It’s always a public holiday in lieu. And in special events day, like Christmas day, New Years Day, like you might be working also. Ya, so that’s how your lifestyle also change. I mean, everybody will have to make some compromise here and there ah. Hmm.
- **NJCL: Okay. Then my next question for you would be, what are your main facilitators or motivators in self-regulating a healthier lifestyle?**
- P23: Hmm. Family and friends! Because they have a better understanding of each other’s life commitments. And also low maintenance friendships. We don’t have to meet everytime. Because we all know we’re in the same life stages, we’re all working, everyone’s tired. And then we don't have to meet every day. Maybe last time we are in uni, when we’re all studying the same course, then will see each other every day right? But then now we probably only see each other once or twice a year, but it's completely fine because whenever you meet them you will feel like nothing much change. And then it's like a friendship that I really appreciate. Yeah. And then, because everyone's understanding on like the life’s stages, then it makes it easier to just make all these choices lah.
- **NJCL: So, okay. So your main facilitators would be family and friends. Is there anything else here you would like to add?**
- P23: Facilitators ah?
- **NJCL: Or motivators? In helping to self-regulate a healthier lifestyle?**
- P23: Facilitators, hmm. Let me think. [Participant is plugging in her audio device. Presumably because of low battery.]. Alright, sorry, can you hear me? Because my Airpod run out of batt.
- **NJCL: Can, can. I can still hear you.**
- P23: Okay, in a very practical sense, I think money. [Participant laughs]. We still have to work, right? Ya, still have to work. And then, and also your personal expectations, I would say. Because I was a very active person back in uni, so I wouldn’t want to change much now that I’m working. Yeah, just have to make some adjustments. That will be my personal motivator. And also salary is like okay. Even though I am very tired from working, but every month I still have to go to work because it gives me the salary that I need.
- **NJCL: Then moving away from facilitators or motivators, what do you think are your main barriers to self regulating a healthy lifestyle?**
- P23: But healthier lifestyle can be like anything right? Like being active, dietary.
- **NJCL: Like with reference to how you answered earlier. Regarding work life balance, getting enough sleep.**
- P23: What are the main barriers ah. I think the main barrier is really, irregular working hours. And night shifts are very, very detrimental to your health. Like you can do it in the first few years. But then, as time goes, you realize how it really affects your life. I think that's it. Because at the moment, if you have regular schedule, it’s a lot easier for you to plan.
- **NJCL: Okay, then, I will just briefly summarize what you shared with me this question on facilitators or barriers in self-regulating a healthier lifestyle. So for you, your main facilitator are your family and friends, because they are all understanding of like life commitments, and even having low maintenance friendships, because having these kind of family and friends is helpful for, I guess, emotional aspect of your lifestyle? They are understanding of your circumstances. Then your next facilitators would be money. I guess, you can link it to, you need a salary to even do the activities that you want to do. Is that correct? Lastly, personal expectations. You were active in uni, so you wouldn’t want to lose that lifestyle, like you would want to maintain what you started in uni into your working life. Then, for your barriers, it’s a lack of regular working hours, so shift work and night shift are all very detrimental. So is there anything else you would like to add for your facilitators or barriers?**
- P23: No, I think that's it.
- **NJCL: All right. Okay, then my next question. I think you mentioned some things on this. But how does your personal self-regulation habits impact your work performance?**
- P23: My personal self-regulation habits impact my, what, sorry?
- **NJCL: Work performance.**
- P23: Sorry? Oh, work performance. I think it makes me less drained out at work. Yeah, because I am able to segregate both work and my external, I mean my life commitments. So when I go to work, I just think ‘Okay, these are just my tasks I need to do’ so it makes me a lot more focused. Like, if I don’t have enough sleep, I’ll find myself tired, or very easily triggered. Or a lot more anxious when doing certain things. But if I am actively doing things that are outside of work, then at work it makes me a lot more focused. Like better performance, I would say.
- **NJCL: Okay, then, this is in tandem with the previous question. But how do you feel self-regulation affects your life?**
- P23: Hmm! Self regulation. I think it makes me enjoy and appreciate it a lot more, when I am able to do the things that I like to do outside of work because it’s so precious time. All these precious time outside of work, right? All the more I will treasure these pockets of time that I have to do whatever I like to do. Like spend time with friends. Focus more on the quality rather than the quantity of time that you have.
- **NJCL: Yeah. So for the next question, currently, are you using any like tools or apps that help you to self-regulate your lifestyle?**
- P23: Hmm. Oh, Lumihealth. I think a lot of my nursing colleagues are using Apple watch. And then, because we can’t wear it on our hands, okay I’m not sure if it applies to all nurses, but most of the nurses in my department, we will wear it on the leg. So we are still clocking steps as we work. And it’ll help us to regulate our, to track the amount of steps that we do everyday and plus the fact you can get NTUC vouchers, it’s like a win-win. Yeah, I think it’s a very good app. And then if you do certain workouts and you clock it, like your Apple watch, then it helps you keep track of whatever you’ve done also.
- **NJCL: Hmm! It's like a step tracker and a calorie tracker kind of thing?**
- P23: I think it’s like a collaboration between step tracker and HPB, if I’m not wrong? [NJCL: HPB: Health Promotion Board]. So for a certain of steps or certain number of points that you have accumulated, then they give you like vouchers or can change to cash. It’s like a little incentive. And then it’ll make you go for like, 10 000 steps a day. Which is also promoting healthy lifestyle. Ya. And then, the thing about Apple watch sometimes is also, if you sit for too long, they’ll notify you to stand for a few minutes, every hour. But, I don’t really need to do that, because in the nursing environment, you’ll always be standing. I think that’s a very good app. I think that’s the only app that I use when it comes to active lifestyle.
- **NJCL: Then, outside of apps, do you use tools such as journaling? This kind of thing?**
- P23: For example?
- **NJCL: Like, journaling. Writing about your day.**
- P23: Oh journaling. Hmm. I take photos. Polaroid photos. To like capture the little pockets of memories. But very expensive in the long run so I don’t do that all the time. But I just take photos for like memories sake. Journaling wise, I don’t really do that. But if there are specific events, or specific day that I really like, I will just jot down my feelings in the Notes in my phone.
- **NJCL: Okay. Then my next series of questions will be regarding the workplace in general. So for you, as a nurse, how do you think the workplace influences your self-regulation habits?**
- P23: Hmm! [Long pause]. I think my workplace. I'm not sure if it happens to every ward, but in the emergency department, in my workplace at least, there’s like a massage chair for the nurses and doctors to use. But actually nobody really have time to use that, but maybe during our break, if you say you want to go for a little massage, then you can go for the massage chair. Then maybe, during night shift, during breaks, you can go for sleep workshop, for sleeping sessions. But obviously during your breaktime. Like, they’ll sleep during their break.
- **NJCL: Ah, okay.**
- P23: Promotion of healthy habits ah. Actually my, my, department has a lot of snack, which is not very good. Like a lot of tidbits. Yeah. Like all the sweet stuff. But like, it’s the most enjoyable part of the break, because you can munch on all these tidbits. Yeah. I guess it makes us happy? It’s also a way for us to regulate our emotional level. Emotional well-being. Oh, sometimes our manager will buy drinks for us! And then, maybe it’s the culture of emergency department. If you’re working on a public holiday, then some of the senior doctors will buy pizzas, or KFC to treat everyone in the department. Yeah.
- **NJCL: So you mentioned something on culture so going to the social aspect of the workplace. How do you feel about the workplace culture among nurses, in helping to facilitate or hinder self-regulation of healthy lifestyles?**
- P23: Culture. I think the culture. You mean my department, like how my workplace help to influence?
- **NJCL: Like maybe, if your nurses go for breaks together, then everyone will tend to order healthier options, then you’re peer pressured to order healthier options, something like that. Do you think there’s somekind of culture that helps to facilitate or hinder a healthier lifestyle?**
- P23: Actually, no eh. I think we are more unhealthy when we are together. Like, we will want to buy bubble tea. Waffles. Eat all the unhealthy stuff together when you’re with your friends. Unless there’s one person in the group who is very health conscious, where he or she is very good at encouraging everybody to be healthy. Then if not, I don’t think anyone care about being healthy. They just eat whatever they want. Yeah. Like even waffles, when we go and buy together, there will be some who will buy oreo and chocolate, like peanut butter. No one really goes for like plain. We’re unhealthy together.
- **NJCL: Okay, then. would you say that the workplace culture hinders more the healthier lifestyles? Rather than facilitate it?**
- P23: Yeah, I guess. Oh but there are some of my friends who go to gym together. But yeah, also because they found like-minded individuals. Yeah, like some of my colleagues will ask if we can play tennis together also. Yeah, so also depends on you lah. Because not everyone likes playing sports or going to the gym, right? Only if you find like-minded individuals, then okay, you can all go together. Yeah, I think that’s the healthier lifestyle. But in terms of dietary. I think that's the least of everyone’s priority. Because everybody just likes to eat good food, right? And good food sometimes is just not very healthy.
- **NJCL: Okay, then, just to clarify. So in terms of like workplace culture in your department, for dietary habits, you guys tend to negatively influence each other to eat less healthier. But then at the same time, there will colleagues who will ask each other to go and exercise, or go gym or even like play tennis together, so would you say it’s balanced? It would facilitate and hinder a healthy lifestyle.**
- P23: Yeah. We eat unhealthy food together but also because we eat together, we are happier. It’s nice to be eating with your friends, right? Yeah.
- **NJCL: Yeah okay. Then my next question will be regarding the nurse managers. And for you, what do you think the role of nurse managers in helping the ground nurses to self-regulate their own health?**
- P23: They're very important because they are the one who sets the culture. Because all of us just listen to our nurse manager. And then if the nurse manager is very into healthier habits, it will have the trickle effect to influence everybody in the team also. Yeah. I mean, it also depends on how close we are with the nurse manager, I feel. If our nurse managers are doing all these healthy things at her side, like in her own, but then but the rest of the department doesn’t know about it, then it doesn't really affect anything also. It could just be that their individual way of regulating. Hmm, yeah. Unless they make it a rule, that they have to eat healthy together. But, making it a rule can also be very difficult because people don’t like to be trapped inside the rule right. They always go by right. I mean, there’s always by-right, but everyone go by-left. So if you put in a very strict culture, then no one will like it also. Because it’s already at work, everybody wants to do things at their own time, and their own decisions and choices already. So if they go to work and they still have to have people telling them what to eat and what not to eat, they also feel like ‘hah, give me a break’.
- **NJCL: Early you mentioned, they’re the one that sets the culture.Then, they have a trickle effect to influence everyone right. Then would you say, like the managers are role models for the ground versus for healthier lifestyles?**
- P23: [Long pause]. I think they definitely play a role in influencing the ground nurses. But I think that, in the workplace, because we are all adults already. People already have their own decisions, their own choices. So they may not really be bothered by what you do. Or like be affected by what you do. They will just do whatever they feel will suit their lifestyle or commitment levels. Because most of my colleagues already have family commitments, right. Like, even if they want to go for healthier lifestyle, they wouldn’t do it. I mean, they won't be able to do it, because of the family commitments they have to go for. So even if our nursing managers are very very active, and advocates for healthier lifestyle, eventually it depends on how the individual sees the importance of having a healthier lifestyle to begin with. Yeah. Because, we do have nurse managers that are very healthy, but the ground nurses are also having unhealthy habits. So I’m not sure how the nursing managers will have a very strong influence when it comes to lifestyle.
- **NJCL: Then moving away from the nursing managers, and into institution wide kind of system. How do you think your institution can help you to self-regulate a healthier lifestyle?**
- P23: Hmm. [Long pause]. Maybe… Institution, I think… Maybe like, corporate discounts for gym memberships. For any outdoor activities, if there are some corporate discounts. I think it will encourage people to go for those. And for food wise, if they give us.. If it’s cheaper. If they make it cheaper for us to buy, then maybe we will order it also. Like, I’m not sure if you manage to interview anyone from [Public hospital name]. But in [Public Hospital name]...
- **NJCL: I might have.**
- P23: You might? Okay. This is hearsay ah, but in [Public hospital name], the institution made the brown rice, like healthier option cheaper. So it will encourage the staff nurses to buy all those healthier option food. But ya, I think. Corporate discounts. Especially for the younger generations. Nowadays, the new generations are a lot more active in their lifestyles, right? Then maybe they will enjoy having all these benefits. Maybe something the institution can offer.
- **NJCL: So mostly like incentive, wise for the staff and also increasing like availability of healthier options. Healthier food options.**
- P23: Mmm. Ya.
- **NJCL: Okay, can. Currently, at your workplace, is there like any health promotion programs in place?**
- P23: Health promotion programs? [Long pause]. I think there were some yoga lessons. Some meditating classes. That was organized by the hospital. But everyone very paisey to go la. [Participant laughs]. So they don’t go. I think those are the only 2 that I know of.
- **NJCL: Then, have you personally went for these programs?**
- P23: No, no.
- **NJCL: Ah, then what do you think will make you want to go for such a program?**
- P23: If my friends are going because I wouldn’t want to go alone. In my hospital basis ah? External environment is okay, but then it’s because its the workplace, it feels a bit strange. To go for all these exercise courses alone. If you’re with your friends, then you won’t really care right, because you’re with your friends? Yeah.
- **NJCL: [Long pause]. Okay, then. right now. The next question is on your lifestyle again. So would you say that, the management is doing enough to encourage nurses to go for healthier lifestyles?**
- P23: Hmm. [Long pause]. No bah. I don’t think the institution is doing alot to encourage healthier lifestyles. But maybe they are, but I’m not aware of. Yeah. But even if you ask me what they can do to make it better, I’m also not too sure. Maybe just like what I mentioned earlier, to go with all the corporate discounts. I guess people see workplace as workplace, they don’t see it as exercise ground. Or a place for them to regulate their health.
- **NJCL: Okay, just to go back on the question on nurse managers being role models for ground nurses, so you already said that they play a role in influencing, but people have their own choices. And because of this they will have their own habits that they will form, because of outside commitments, or because of their own personal personality. Then do you think nurses are role models for healthier lifestyle for patients?**
- P23: Nurses are. I think there is a society expectations that nurses should be healthy. Or healthcare professionals should be healthy. But then, I also say that nurses also eat Mcdonald’s right? [Participant laughs]. Yeah. Hmm. [Long pause]. I think… Is there a role for nurses? [Long pause]. Also bah, I don’t think the nurses have to be. Don’t have to be super healthy. But at least have some form of like know. Don’t have to be everyday brown rice, or like only eat fruits and vegetables that kind of healthy. But, maybe, cannot be in a BMI range that is very very unhealthy. Should still be of a certain standard. I think it’s not just to be a role model to the patient, but if you are very unhealthy then it makes your job very difficult for yourself, especially because nursing itself is very taxing; very procedural based, skills-based. And then if you’re very unhealthy, it might make it very difficult for you to perform this all tasks. So it’s not only just for the patient, but more on, but also for themselves.
- **NJCL: I'm not sure about your department, nurses also have to give health advice to patients, regarding changing their lifestyles. So, how do you feel about… Do you think some of the nurses are practising what they preach? How do you think this influences them?**
- P23: Influences the nurses themselves?
- **NJCL: Yeah.**
- P23: Practising what they preach ah.I think most of them will learn. I think nurses when they give patient education, they have the background knowledge of moderation. Like everything in moderation. So when we give patient education, we are giving them in the form of telling them to moderate. And regulate their own habits right. For example, drinking. We are not telling them not to drink, but maybe drink in moderation, like everything in moderation. Ya. Hmm, maybe, moderation wise, they are practising what they preach. But if we are telling people they shouldn’t drink, or they shouldn’t smoke, or they shouldn’t eat this, shouldn’t eat that. But then we ourselves are not doing it, then it’s a bit ironic lah. But actually the patients don’t know. [Participant laughs here]. Yeah. But because it’s our job right, to teach them what we have to teach. Mm. But I think, the nurses will know when to stop, because they have learned what is the overdosage period. Like overdosage range. Like they will know how to stop themselves when they know it’s a bit too much. Ya.
- **NJCL: Okay. Then, just to summarize what you shared with me for my last few questions. So for my question on, how do you think your institution can help you to self regulate a healthier lifestyle? You mentioned mostly providing incentive for the staff, also how increasing availability of healthier food options. You also mentioned that in [Public hospital name], they made brown rice cheaper for the staff as well. Then regarding health promotion programs, you mentioned that there are yoga lessons, medicating classes, organized by the hospital but you feel that everyone is too paisey to go. And you would only go for it only if you’re friends are going because you mentioned that, the workplace, people don’t really go there to self-regulate their own health.**
- P23: Yeah.
- **NJCL: Yeah, then my question on has the management actually done enough for the staff nurses, you mentioned that the institution has not done enough. Maybe the institution is doing somethings, but you’re not aware of. Is this right so far? Do you want to add anything here?**
- P23: No, I think you perfectly summarized what I said.
- **NJCL: Then, my next few questions was talking about nurses as role models for patients. You mentioned that there is a societal expectation of healthcare workers to be very healthy. But you believe that they don’t have to be that healthy, but they should at least have some standard, so that it actually. So their work won’t be as difficult for them. Because you mentioned that nursing is actually very taxing, so if they are not healthy, this might hinder them from doing all the tasks that they have to do during the shift. Then on the question on whether nurses practice what they preach. You mentioned that when giving patient education, nurses actually talk about moderation a lot. So most of the nurses will follow their own advice when it comes to moderation. And because of their background knowledge, they know how much is too much so they won’t go overboard. Is this right so far?**
- P23: Yeah. It’s true. They will just know when to stop. Hmm.
- **NJCL: Do you want to elaborate anything here?**
- P23: Hmm, hmm. [Loong pause]. Because they also have the prior medical knowledge on what causes maybe high blood pressure, like diabetes. So they will know they shouldn’t take too much sugar. Like for nurses lah. But because for patients, or the general public, they don’t really see the whole, like pathophysiology of how disease progress. So they know sugar cause diabetes. But they don’t see how it develops over time. But for nurses, or healthcare professional, they learn about it in school, so I believe it gives them a good. It will let them know how much is too much, like I said.
- **NJCL:** **Just to clarify on this point, do you think nurses who for example, they know how much is too much, but for example they still drink a lot of bubble tea. Do you think these nurses when giving health advice to their patients, do you think they feel guilty? There’s a sense of guilt?**
- P23: I don't know if they feel guilty. Maybe. Hopefully? Hmm. [Long pause]. Because ultimately, we also cannot change what they believe in, right? Or what they like to do. Ya. So even if nurses itself as a role model, but it’s also just a job for them. Ya. Not everyone see it as their role of being a role model to every patient. They probably just see nursing as a job that they go for everyday. What they like to eat, and what they like to do outside of work, is completely different, or a separate entity to them being as a nurse.
- **NJCL: Is this applicable for you?**
- P23: For me? [Long pause]. For me, I’ll usually, I will educate them in whatever I believe in, right. Like in what the institution wants me to advocate. And also because, maybe because I have my own active lifestyle, right, so when I advocate, I don’t feel as guilty because it’s actually what I am doing and practising. Like eat more fruits and vegetables. Eat healthier food. Drink less sugar. Exercise more. For me, it doesn’t really affect anything, because it’s actually what I’m doing.
- **NJCL: Okay. okay. Understand. Okay. Then, do you have anything else that you would like to speak about? That was not mentioned in the interview. Any further comments you want to share?**
- P23: No, I think it's quite comprehensive. I think. Or at least, that’s whatever I can think of at the top of my head right now. [Participant laughs here].
- **NJCL: Okay, then this marks the end of the interview. Thank you for your time and participation.**
- P23: Okay, thank you. All the best for your research! [Participant laughs here].

-End of P23 Transcript-

# **Interview #24**

Participant and interview details
 Participant number: 24 (P24)
 Date of interview: 1 Nov 2023
 Interviewer Number: Navarro John Christopher Lambino (NJCL)
 Time IDI started: 1500
 Time IDI ended: 1600
 Observational notes: Participant is in her nursing uniform and seems to be in a good mood.

Section I: Interviewer’s summary
 The individual interview was conducted via Zoom. The participant appears to be in her office, wearing her nursing uniform. P24 is 36-year-old Nurse clinician, working in a patient clinic, which is part of a public hospital. She has office working hours, and she roughly have 8-10 patients a day. Her working experience has mostly been on oncology, and now her specialty is on breast care. She has moved institutions from a public hospital to her current institution, another public hospital. Her responses tend to be on a shorter side and prompting had to be done. Most of her responses have been mentioned by previous participants. In terms of P24’s lifestyle, she leads a rather sedentary lifestyle despite not having shift work hours to worry about. When asked if she wanted to change, she is reluctant to do so, implying that her health is not much of a priority. This is further supported by her statements saying that, there is only a 50% chance she will follow the same lifestyle even if she does get cancer like her patients. She believes that nurses are not role models for health, contrary to what the other participants have said. Before employment as a nurse, her student lifestyle and working nurse lifestyle is mostly the same, indicating perhaps that habit formation may have started at a younger age. Notably, she mentions that exercise for her instead of helping to remove stress, is a stressful activity for her so she does not engage in it. She also mentions that chronic illness like arthritis is preventing her from doing certain activities. P24 is older relative to most of the other participants, hence maybe why health conditions is not a barrier for most of them. Perhaps, my subsequent interviews will need older participants. P24 throughout the interviews appears to be in a good mood, often joking and laughing as she answers. Her lifestyle is currently sedentary with no thoughts on changing it for the better, which she mentions may be attributed to her laziness and her own way of thinking. Despite this unhealthy lifestyle, P24 is still seemingly content. In conclusion, this interview has been very insightful as compared to most of the other participants, this is an older nurse with years more of experience and is not hindered by shift hours. Yet, she showcases an easy-going lifestyle that is not packed with activities outside of work.

Section II: Transcript

**NJCL: Okay, good afternoon, miss. I would like to thank you for taking the time to speak with me today. My name is Chris, and I'm a NUS Year 4 Nursing student. The aim of this interview is to get a better understanding of the perspective versus have on self-regulating their own health behaviours, and how various factors may influence this. I want to find out what are your views, opinions, and experiences onthis. I would really like to encourage you to speak what comes to mind? There are no right or wrong answers. May I have your permission to continue with this interview session?**

P24: Yes, you can.

**NJCL: Okay, thank you so much. Then let's start with some basic questions like, what are you working in right now?**

P24: I'm actually running the breast cancer service. So I run inpatient and clinic. Hello, yeah. Okay, so and so depends ah, we do go on rotation. So maybe, like a few a couple of months I'll be in the breast clinic then a couple of months I'll be covering the inpatient wards.

**NJCL: Hmm!**

P24: [Participant laughs here] So what else do you want to know about my work?

**NJCL: Could you perhaps, like describe what you have to do on a daily basis?**

P24: Daily basis in my work? Okay, so this is breast service. So I see many of my breast cancer patients. So from, let's say, pre-operative we are still like, not decided on which surgery to do so I will have to meet them to clarify their thoughts and doubts and things like that. Then once they have signed a content for their surgery, then I'll have to do what we call a pre-op counselling. That is, to prepare them for what to expect for the surgery. How to take care of the wound, especially after they are discharged home. Things like that. Then post-operatively. They will come in, maybe about once a week for their wound dressings. Then apart from this group, I sometimes do tend to what we call the fungating wounds. So lets say if I have stage 4 breast cancers with very large wounds, they will also see us sporadically. But there’s also those benign cases like uh breast abscesses, uh, excision biopsy that turned infected. So yeah, have to say, so one day down. I man the clinic for this month. So maybe we have about, I say roughly, 8 to 10 patients in a day. Around there.

**NJCL: Hmm, then for you; how would you describe the work? Is it manageable?**

P24: Work-wise? Yes, manageable. [Participant laughs here] at least now in clinic, I have a partner with me. So she's from Woodlands, so at least when they have 2 persons tending to clinic load. Usually our clinic load is actually higher than inpatient load.Yeah, so with 2 of us around. Easier to manage. Because sometimes one person can be doing what the other one can be in a call to another room with the consultant to settle something else. so more flexible this way.

**NJCL: Then can I clarify with you, your work experience before you were in the breast care service, which discipline were your workinng in?**

P24: Pretty much. All my nursing life is all in oncology. So my first 5 years was in an oncology ward at [Public Hospital]. Then my second 5 years in [Public Hospital] still, but in an outpatient setting in a cancer center. Then, since 2020, I moved out to [Public Hospital]. So now, doing breast care but still breast cancer. So it's still on oncology somehow. Yeah. So that's all my experience. Yeah.

**NJCL: I see. So it’s all been oncology. Very experienced in oncology.**

P24: Yes.

**NJCL: Thank you so much for that context. My next series of questions will be asking mostly on lifestyle. So, as a nurse, how would you define a healthy lifestyle?**

P24: How would I define? I would say. Healthy diet, exercise and learn how to moderate stress and let's see. Enough sleep. Yeah, more of the basic stuff. Yeah.

**NJCL: So specifically on what you mentioned on healthy diet, could you elaborate on it in relation to your own dietary habits?**

P24: My dietary habits? My own, my own! [Participant laughs here] Oh, it's very unhealthy. [Minor noise in the background] Sorry if you hear laughter from the background. My colleague is with me.

**NJCL: It’s okay!**

P24: So my diet. I would say. Because I'm still staying with my parents. So if she [Participant referring to her mother] cooks I have to eat home right. If not actually, majority of the meals are outside. So if outside,, you can't control how they cook. Right? Then I also have the tendency to go for fast food. That's why I’m this size! [Participant laughs here]

**NJCL: Okay. Then, would you say you are trying to change your current dietary habits?**

P24: No, I wouldn't say so. [Participant laughs here] I'm just like, okay, it is what it is. You see, I wouldn't mind so much, you know, even with a background on oncology. Yeah, I don't really care so much into my diet.

**NJCL: And then, what do you think will be an impetus to make you change your diet? What will make you want to change?**

P24: What will make me want to change? Wow! Has to be very drastic things, I guess, cause I didn't change after I got ill. Yeah, pretty much back to square one again. Only I will say to me is that if don't have control over diet as in like how, how do I phrase this properly? Okay. All my 3 meals, or whatever I put in my mouth as to be, you know, decided and prepared by somebody else or somewhere else, you know, quite fixed. I don't have a choice then, of course, it will turn healthy, But it is all up to me then. Then is not healthy any more. Yeah.

**NJCL: I see. Then, moving away from diet. You also mentioned exercise. So specifically on your physical activity, how do you feel about your current lifestyle? Do you feel like changing it?**

P24: In terms of exercise? Same thing. I pretty much until now, I don't really change anything anymore. If my current lifestyle is really like office hours. So when at work- so let’s say today when I’m at work, it’s pretty much sedentary. At most it's out of my chair to the couch, back to the chair. Ya, so even the walking is quite limited to within this clinic only. If lets say today I’m running wards, then that one will encompass more walking. Because from office to ward, then back to office; it’s everywhere you’re walking everywhere. Then let’s say if like personal, life-wise, out of work, I hardly move. Once in a while, the only exercise I actually do is actually cycling. So mostly on a weekend, but not every weekend. Depends on my mood, I guess.

**NJCL: I see. So. Currently, you don't feel like changing it as well. To decre- [Participant shakes her head] No? Oh, I see. Then could I ask the similar question like, What do you think will make you want to change your physical activity levels?**

P24: Actually, nothing much. [Participant laughs here] Ya, really nothing much. And what will make me want to change? Hmmm, no. Nothing strong enough to make me want to change as of yet.

**NJCL: Okay, I see. Okay, then earlier regarding the healthy lifestyle. You also mentioned learning how to manage stress. Could you maybe elaborate on that? Like how, how do you personally moderate your own stress?**

P24: For me? My stress level? Mmm. Some people do. like you tend to hear that moderation means you go for exercise. Things like that, right? I haven’t tried it before. It doesn't really work for me. Or should I say that exercise makes me more stress? Okay. So for me, it's more of its rest. So usually we, no matter how frustrated I get at work, or things like that. Well, I think that if I can sleep well, then, basically, I get recharged. Everything resets itself quite fast without me having to do anything. Yeah, lets say, what else can I manage my stress? Have, having a healthy vice? Yeah. So let's say, like, previously, previously, I’ve ever like, try things like Yoga. You do meditation all this. I’ve ever tried. Doesn’t really persist very long for me. Hmm. Because all this, it really needs time and dedication to go into it, and, you know, be consistent in it. So I guess I'm not a very consistent person. [Participant laughs here] Yeah. So it's a very realistic view. I don’t really force it onto myself.

**NJCL: So to clarify, for you it’s mostly through just sleeping through it to recharge?**

P24: Yes. Yes.

**NJCL: I see, I see. Cause you also mentioned getting enough sleep, basically.**

P24: Hmm! I guess right now the more important thing is like, trying to keep my, you know, they say, work-life balance, work-life balance. But to me it’s like trying, try not to continue working once we are off work hours. That is, you know, keep those hours personal, do things that you want to do. Yeah, as long as it's not work. To me, it's like a stress management. Yeah. So you can be working 24/7. I think that that's adds on to the stress. Yeah. So off office hours on weekends, you know. Have some personal time to do some personal things. Hmm.

**NJCL: Okay, I see. Maybe, could you describe to me, how did you lifestyle change after you started to work as a nurse?**

P24: How the lifestyle changed? Let me see, did it really change? Hmm… Let’s say if between student years versus work life, right, I don’t think it changed much. I really don't think it changed much. Maybe the activities that I get engaged in changes. That is that now, because I'm working, I have an income. I can choose the kind of, you know. How I want to spend my personal time. I have a more variety of things to do. Okay, compared to as students. If not, then pretty much because the hours are pretty fixed, school hours versus work hours for the time being. Lifestyle wise also almost the same. Like school life, you also eat in your school canteen. Pretty much the same now. If you eat at work, you eat in Kopitiam. [Participant laughs here]. Yeah, I would say there’s not much changes.

**NJCL: So this is student life and working life. Do you think there was any change when you we're working in the, you mentioned like you were working in the oncology ward. Before you actually transferred all the way to breast care service. So do you think there was a change in lifestyle there where I would assume there was shift hours?**

P24: Yes. Okay. So if I say we're talking about shift hours I would say, my personal time is pretty much all at home. So it's either work or home, sleep. Really like that. I, I don't really like, put in activities. Anyways, I'm not a very social person. I'm more of like stay-home person. So if lets say working shifts, then all those non work hours. I'm pretty much at home most of the time. Yeah, if compared to now with office hours, then, at least, you know permanently, my weekends are free to put in activities lah. Yeah. So at least on the weekends, or even after work, you know, having dinner out with colleagues or friends will be easier. Yeah. Also, meeting up on weekends will be easier compared to when on shifts. Yeah. So that’s the main difference only. You have better control of your time compared to shift hours.

**NJCL: Then you mentioned like, because shift hours, you usually just stay at home. But now you have office hours, so you have more, more time to control. Then, does this mean like, ever since after office hour; office hour schedule. you are engaging in more activities outside?**

P24: If compared to shift hours, then of course. Because let's say if like during shift hours, pretty much once I’m off work, I’m at home. I wouldn't lined up activities after work.Because, I don't know, although I'm younger, but with shift hours, I get more tired. Yeah. And last time I used to. My workplace is super far away from where I stay. Yeah. So the journey back and fro from home to work takes at least one and a half hours. Yeah. So that really cut down my rest time. Yeah.

**NJCL: So would you maybe share with me like examples of what kind of activities you have lined up now that you have office work?**

P24: Hmmm. What kind of activities? Mmm. Most of the time is really, makan. [Participant laughs here]. That means I dinner out. Then, uh with my long time friends we go out during the weekends, so we do what we feel like doing la. So it can be movies. It can be massage. It can be, you know, visiting some place. Things like that. [Participant laughs here] But I would say, a lot of these activities involve makan. Yeah, so usually, I think, I guess always easier to meet up and have a meal. We sit down chit-chat, you know, for the two to three hours. Yeah. Yeah, that’s the most frequent things. What else do we? In the near future I’m going travelling with a colleague also. Yeah. So we can plan all those. Other activities. My cycling. Cycling, I sometimes meet my colleagues. Yeah, so we meet out. Then pretty much spend a whole late afternoon evening together. Then if we cycle, we eat also. Then cycles some more then home. Yeah, pretty much this. Okay, after talking to you about this, I realise my life is pretty boring. Anyways [Participant laughs here].

**NJCL: [Laugh] No, no I understand. Having time for yourself is important as well.**

P24: Oh, yeah. Mahjong. My colleague just reminded me. What about mahjong. That’s like very sporadic. Maybe that few times in a year only. [Participant laughs here]. Alright, okay talking about that. Now, I look at you, drinking person. [Participant laughs here]

**NJCL: I see, I see. Okay. I think so far, you shared with me physical health wise, in terms of diet and exercise. Also shared with me like emotional and mental aspect which is like doing leisure activities, like socialising and having enough sleep. Then how about the spiritual aspect, is there, anywhere in your lifestyle where spirituality plays a part?**

P24: For me, not so much. I will say spiritual-wise, or religion-wise. I pretty much go with my mom. So if she demands my presence at temples and things like that, I yeah, I better show up. You know, or else she gives me hell. [Participant laughs here]. Other than that, if we say from my own end point; no. I wouldn't initiate this kind of yeah, activities. Yeah. Spiritual wise and things like that. Yeah, no.

**NJCL: Okay. I think. My next question. You answered a bit. But.what are your main facilitators/motivators in self-regulating a healthier lifestyle?**

P24: I would say if let's say today, I have some chronic illness, things like that. It's quite drastic. Maybe that will influence me to change. And what else? Hmm. no. My mom tried to control me. It doesn't work. [Participant laughs here]. What else is there, any hints?

**NJCL: Hmm, maybe your friends jio-ing you to go cycling?**

P24 :Hmm. Oh, yes.

**NJCL: Or is it not really applicable for you?**

P24: Hmm, let me see. Not all the time. Got la got la. If they call, I will turn up. But not all the time. [Participant laughs here]. What else is there? [Long pause]. I think hmm, yeah. That’s pretty much it.

**NJCL: Okay, hmm. Okay. Can. Then, moving away from facilitators, motivators. What do you think are the barriers like what's hindering you from achieving a healthier lifestyle?**

P24: Hmm, what’s hindering me. I guess the most important thing is the way I think. As in like my belief and thought on you know. How important these things are in my life. Can you repeat the question again?

**NJCL: What are your main barriers in self-regulating a healthier lifestyle?**

P24: Main barriers… So my own beliefs, my own thoughts. And…how do I phrase this. It’s like sometimes, okay I think I should go and exercise and things like that. But if I were to wake up in a very like bad mood, no mood. Just don't feel like doing it any more, then I wouldn’t do it. Yeah. So I guess mood and see whether I am tired or not, that would change my original plan quite a bit. Is there any other deterrent… I would think my own health? Yeah.

**NJCL: Could you elaborate?**

P24: So things like, how to say. Like chronic illnesses and things like that, because I do have a bit of arthritis. So sometimes certain activities I can’t engage. Things like brisk walk, long walks, I can’t. If not I get pains. Yeah. Then,or things like previously, I had a long phase of TB **[NJCL: Tuberculosis]** So I was on meds for a long time. So a lot of my time is you know, how to say. Rather stay home than go out. Just in case, you know. Although it's like no longer infectious. But I do have that caution of, you know, not going out. Or like during the phase where I had long meds right, then immunity is a bit on the low side. So I had to stay away from crowds, so it’s like okay, I’ll just stay at home. I pretty much always stay at home. So yeah, somehow it became like a part of me already.

**NJCL: I see, okay, I'll just briefly summarize what you went through so far. This question like regarding what are your main facilitators and motivators in self-regulating a healthier lifestyle. You mentioned that the potential of chronic illness, or like a drastic illness will probably influence you to change. Then you mentioned like, perhaps if your friends ask you go to and cycle or exercise, you’ll probably join them? Then for barriers, it’s mostly your way of thinking like-**

P24: [Participant nods] How important this is for me.

**NJCL: How important it is for you. And then you also mentioned like, maybe fatigue. Like being too tired to do all these like healthier activities, and lastly, you mentioned chronic illnesses, like how because of arthritis, you cannot engage in some of the activities or else it’ll get painful. Then you also explained your experience with TB. Because of your immunity being on the low side, you want to stay at home, because of this experience, you it tends to influence your current lifestyle until now, is that correct? s there anything else you would like to add for facilitators and barriers?**

P24: I'll say one of the major barriers will be; hmm, I'm just lazy, I shall admit to that. [Participant laughs]

**NJCL: I see. Ah then, for my next question. How do you think your personal self-regulation habits impact your work performance?**

P24: My self-regulatory habits, as in?

**NJCL: For example, having enough sleep. Spending leisure time with friends, to be less stressed. Lifestyle habits that you have currently, do you think it impacts your performance at work?**

P24: Mmm. I don’t really think so. Wait, let me get my colleague’s help. [Participant turns to her colleague in the same office as her to ask] Okay she’s not listening in. So I don’t disturb her. [Participant laughs] I don’t know how to phrase this. But at work, no matter how tired I am or how frustrated I am, I can; I'm able to still function well at work. I don't show it at all. So it’s more like you know, when the clock strikes 5:30. That’s when I shut off right, then all this will just surface. But while at work, it’s like just another me. Usually another me. I don’t know how I’m able to do that. But all these years its like that lah. Hmm, if I’m at work, I’m quite a different person compared to what you see me when at home or outside of work. So function wise. No, I don't think there's any impact.

**NJCL: Okay. Oh, how about outside of? Do you think your self regulation habits impact your life outside of work?**

P24: Hmm. [Long pause]. Impact my life outside of work… What you mean is like, these habits, self-regulatory or not, is just part of me. Does it impact my life? No, it’s part of my life. Am I making sense here? [Participant laughs]

**NJCL: Yeah. Like. when I say self-regulation habits, I’m also talking about maybe if you engage in physical activity, if you eat healthier, do you think any of this affects your life or outside of work?**

P24: Hmm, so it’s like if today let’s say I’m a very active person and I exercise and I eat healthy, would it change my life, personal life. Hmm. I don’t know. I seriously don’t know. Hard to imagine. Let’s say I theoretically want, of course it will impact.

**NJCL: How? How do you think it will impact? Hypothetically?**

P24: Well, let’s say, I guess the overall health will be better. Healthier weight and all that. What else could I do? I’m at loss for words. I don’t know how to answer your question. [Participant laughs]

**NJCL: It's okay. It's okay. I think on the same idea. Since you’re working in the breast care service centre, as I’m sure nurses do a lot of patient education. Do you think your own lifestyle habits impact how you give health advice to patients?**

P24: Ooo, okay, that does. Things like, how to say, because our patients tend to ask us. It's more on prevention and things like that. So on what should they eat, you know, exercise and things like that. Of course to a degree you can tell them on what to eat, but it’s not a 100% foolproof thing. But it does remind that sometimes when I do patient education, at the back of my mind, hmm. I’m not doing what I preaching. [Participant laughs]. Yes, very true. But it doesn't really make me want to, you know, change anything. Yeah it’s like, they’re here to see a nurse, I can tell you what a nurse should be telling you. Likewise when they ask for advice, do they need to make such changes? That’s also another thing you see. Yeah, but I do have this sense of, I’m not doing what I’m preaching. I’m very aware of that.

**NJCL: Do you feel guilty for this kind of, for not doing what you preach? Do you feel a sense of guilt?**

P24: Guilty? I won’t say it would be guilt, it’s just like sometimes if I were to teach or educate, I was thinking that you know, somehow I leave allowances to understand that no matter what I teach, they might not do it. Same like how you tell smokers, smoking gives you a higher risk of lung cancer, they still smoke. Because to me, this is like, just life. These are humans. Same like me, I’m a human too. So I also do understand the risks. Things like must eat healthy, keep a healthy weight so as to avoid a lot of chronic illnesses, diabetes and things like that. I’m also aware. But I’m the same, same as them, I might not listen. [Participant laughs] Yeah, I will not. Yeah. I will not make the drastic changes. Until you know. You know, I don't know what kind of big things has to happen to make me change. Yeah, is it? This is just basically what I see la. And I will say some of my patients, like not all. So not really guilty, but it makes more, whats the word for it? More understanding, if patients were not to heed such advices. You know, like you know? So if they were told not to do and they keep doing it. I will also accept it la. Compared to maybe 10 plus years ago when I first started out as a nurse, of course I do have that, I would say it’s more like a frustration. Yeah frustration of like, ‘yeah you come to me for advice and I tell you what you have to do but you’re not doing it. I could see the same person, talk about the same things, 4-5 times. The person is still not doing it. So when I'm younger I will say I have more difficulty accepting these kind of circumstances compared to now. I'm just living the same life as my patients, doing the same things as my patients. Yeah

**NJCL: Okay, I think you mentioned a bit earlier, but just to clarify. If you give health advice like dietary advice, it does not influence you to make any changes?**

P24: No, if I give you a direct example, because our patients tend to ask us like, how should they change their diet to, you know, reduce their cancer risk or cancer recurrence risk? So you see, our advice will be telling them like, Okay, don't need to make very drastic things that you know. Change everything organic. Or go full vegetarian. All this is a big no, no, from our advice point of view. So what we told them is that: Okay, what you need to do is try to eat whole foods, more fruits and vegetables. Best is home cook lah or self-cook rather than eat out, so that you have better control over, you know the ingredients, the oils and the things like that. What else do we tell them? We tell them to avoid things like barbecue, fermented, preserved, canned foods. These kind of things. But basically, if I say today, I'm not in their shoes. I am who I am now. At this moment, my diet actually consists of pretty much all these things. Yes,so I do know, and I can preach the things but this moment I’m not doing it you see. Yeah, I'm not doing it. I never know if one day kena [NJCL: Colloquial term for hypothetically] cancer, will I make such changes in my life? I’ll leave it like, 50% I still might not.

**NJCL: Okay. Then, just to further elaborate on this question and idea, do you think nurses are role models of health for patients?**

P24: Hmm.. Why do nurses have to be role models of health? Why would you have that concept in the first place? [Participant laughs].To me there, there is something equivalent to how do I say things like, you know, there's there's statements and say, ‘Oh, doctors, cure people, so doctors don’t fall sick’. Yes to me, nurses as models of health sort of fall in the same category. Yeah, so I don’t feel it. I don’t feel it that way. Yeah, I guess it's also part of my life, because it's like how to say, the most consistent thing, is that because I've been like overweight all my life. Yeah. So as an overweight nurse. If you were to start doing nursing, then, of course, you will see patients who are the same. You know, obesity, overweight, diabetic patients. I still have to do what I need to do, but I can't be a role model to them. Given that first place. First eyes. They set eyes on me. They see me this size, ah. I can't say anything. Yeah, from my size. I already cannot convince them anything, right? Yeah. So I wouldn't force myself down that route. To make myself a role model. I would say, I would prefer to be you know, have anythings you want to clarify, have medical or nursing advice, of course I can give it to you. But yeah, I’m just not that role model for these things. Yeah.

**NJCL: Yeah okay. Thank you so much for that answer. My next question is, a bit on a tangent, but are you using any tools or apps to self-regulate your lifestyle?**

P24: Tools or apps more modern? What do you mean?

**NJCL: Like maybe tracking your calories, tracking the amount of steps. That kind.**

P24: Tracking calories no. Tracking steps no. I ever tried an app that monitored sleep. Like sleep quality whether I was snoring and things like that. I ever played with that.Hmm, what else have I played with. Health trackers… If I were to go cycling. yeah. So like, distance measurements, the speed, all these. I do use an app for that. Yes.

**NJCL: Okay. The sleep app you played with, you're not using it anymore? Are you still actively using it?**

P24: Now it's more of an on off. Yeah. So basically the the sleep it does measure like time to fall asleep. How much time I'm asleep, how much time I am asleep I am snoring. A bit confounding because my dog sleeps with me so she snores louder than me, so the app is not accurate anymore. [Participant laughs]. Sometimes when she’s not sleeping with me, she’s sleeping with my mum. Then okay, maybe I will switch it on for the night before I sleep.

**NJCL: Ah then, besides, apps, maybe like, do you use any tools such as journaling? To self-regulate your emotions, things like that?**

P24: Sorry, what tools are that?

**NJCL: Journaling. Like writing in a journal.**

P24: Oh journaling? No. I don't write. I don't journal. I do read sometimes. Not consistent. I play a lot of game apps. Does that count? Yeah. Because sometimes, if you want a good distraction they'll take your mind off. From things that are bothering you. Yeah. I’ll start playing games.

**NJCL: Okay. Understand. Okay. Okay, can. Then, my next last questions will be regarding the workplace. So for you, as a nurse, how do you think the workplace influences, self regulation, habits?**

P24: Sorry, can you repeat? [Noise in the background]

**. NJCL: As a nurse, how do you think the workplace influences self-regulation, lifestyle, habits?**

P24: Workplace ah. Well, I would say. Let's say I’m going back to shift hours- all these is determined by the workplace actually. So your work hours, the amount of time you’re demanded to work. Okay, I won’t say demand, but expected to work. That will influence a lot on how you will regulate all these things. And same thing if like… Well, in a hospital, you pretty much only go Kopitiam, Kopitiam. So although there are some things they can change like healthy food in Kopitiam. But if you work here for 10 years, also sian, right? [NJCL: Colloquial term for getting too sick of the same thing] [Participant laughs here]. Yeah, so you eat the same food for 10 years also super sian, for all the people working here. So all these actually affects lifestyle in general. What else is there? No la, okay maybe these things. They do play a part. But to me these are areas that you can't really change them, I would say. Yeah, it's not up to you. It's, it's just like that. You come into that work environment, it’s just fixed like that. Yeah, of course, some things like work hours, things like that, of course you can a bit like negotiate with your boss and things like that. But work-life wise, quite fixed.

**NJCL: Okay. I think so far, what you mentioned is regarding like the physical environment. How about like the social aspects of the workplace? like, for example, how do you feel about the workplace culture among nurses, in helping facilitate the self regulation of healthier lifestyles?**

P24: Well, if let's say, in, in hospital setting. Of course they do have like activities planned for us. Things like you know. Sometimes after work they do have, like yoga classes, pilates classes, Zumba back classes, that you can join. Then things like they do have a gym in the hospital that you can use. Then, culture wise, of course, the immediate environment, the immediate group of people that you are with during work, right? How much are they into such activities also can influence whether they will like jio you or not. Whether you want to attend these activities or not versus having people who are always ‘no, no, no,’ you know? Very rejecting to this kind of activities also can influence you whether you will eventually end up doing these activities or not. Yeah. Hmm, what else is there? Most people wise… Culture wise… hmmm…I don’t know. They do like how to say, for, for staff. From the hospitals perspective, they do have things like counselling service, you know. If if let's say you, you need psycho-emotional help. You need psycho-emotional help, there’s people you can talk to. Yeah, we have these avenues to turn to. It’s available within the hospital setting itself. Yeah.

**NJCL: Then, for you, currently, specifically, your workplace, would you say that the workplace culture is a positive one that influences its nurses to be active, or is it a more negative one?**

P24: Uhh, negative! [Participant laughs]. Mainly, the people I’m with… No, we actually don’t jio each other for healthy lifestyle kind of activities. We only mainly jio for makan and drinks only. [Participant laughs]. I guess, I don’t know. Because we are working the same environment. We know how frustrating it is. We face the same frustrations, so end of work we want to let loose and things like that. So it’s usually makan and drinks, rather than oh, let’s go jogging. So far here, nobody utilises the gym. Even the classes I was talking about, here also don’t have. Not that I’m aware of in my immediate environment and immediate people. [Participant laughs here].

**NJCL: Then the activities you mentioned earlier, like the pilates, zumba. Have you ever attended any of these classes?**

P24: Not here. I did. I did try those when I was in [Public Hospital name]. When I was in the outpatient setting whereby you worked office hours. Easier to go for these kind of things. And that time, if you were to just give it to myself and myself only. I won’t sign up for this. So that time, I actually went, is because my immediate friend that was with me wanted to go, so jio me to go with her. So I went. [Participant laughs] Yeah, yeah. So we finished like 10 classes, not too bad.

**NJCL: Wow, 10 classes?**

P24: Yeah, yeah. Once a week kind of thing. Yeah, an hour every evening that time.

**NJCL: I see. But for your current institution you've never attended yet?**

P24: Over here? No. Mmm.

**NJCL: What do you think will make you want to go for this kind of activities here?**

P24: Same thing, ah. Peer pressure. [Participant laughs]. If, if really like give me, my own choice. I don't know. Right now, because my, my, my one and only avenue is really just cycling. As I got my own bike, I stay in the East. So I'm quite near to East Coast. Yeah. So you see, if I say, I want to exercise, it's just can grab my bike, cycle all the way there and make my rounds, then come again. Yeah. So I don’t need to jio people and things like that. It’s more free.

**NJCL: Okay, then, talking about workplace culture.What do you think about the role of nurse managers in helping like ground nurses to self-regulate their own health?**

P24: O, hmm! Let me see. The role of nurse managers…I guess something like flexibility. Essential things like, because you’re a manager, you can have the absolute control or concern to people lah. Getting to leave on time. Don't need to stay back, you know. To clear shifts. So all these will influence the staff, whether they want to go and sign up for, especially those classes that we have in the hospital. Because whether you appear or don't appear they start on time, you see. So if you get dragged back at work, you can’t finish. Or your manager doesn’t appreciate this time, you know. Because to some managers, it’s like your duty, you jolly well finish everything before you go. You better have time, you know, to prep for your next day cleaning, or things like that. I have met these kind of managers. So it makes it hard for the staff to sign up for these kind of things because when you can’t control your own time. Then, it’s like a waste of money to arrange these kind of thing when half the time you can’t attend because you’re stuck at work. Yeah, so as nurse managers, they do influence a big part on this. Whether the staff can leave on time to do these kind of activities. Then same thing. We do have managers who, yeah, over the weekend also still messaging the work group to do this, to do that. Yeah, so that one sometimes cannot siam [NJCL: Colloquial term for escape] them. Yeah, all those in the hospital, you know. Love to put ‘work-life balance’ in their mouths, yeah, but not everybody complies to it. Not everybody understands it, and not everybody agrees with it. So if really today is the day, then suey la. If your manager is the kind, workaholic, you know. At wee hours, at midnight hours. Saturday, Sunday also continue to do work one ah, then when they delegate, you also kenna. Or things like that. Run on a very tight schedule. By when? By Monday morning 1 o’clock, I want this thing on my table, that kind. Then boobian la, your weekend is all the way doing work again. [Participant lauighs here]. Yeah, so all these will affect how you live your life as well.

**NJCL: Like, the ground nurses, if they engage in all these healthier activities, it is largely influenced by nurse managers, and how their attitude is towards work-life balance? Is this correct?**

P24: Yes.

**NJC: Okay. Next question is, how do you think your institution can help you self-regulate a healthier lifestyle?**

P24: My institution? Hmm. We do have healthier options. Maybe healthier food choices. Other than Kopitiam, we do have healthier options. How else can the institution influence this. I don’t know, I feel that I am beating around the bushes back to the same things again. [Long pause].Is there anything? Wait ah, let me think first. [Long pause]. I can't come with any more new things from this institution? First, things like activities, I have mentioned previously. No, I don't have any more new things to add to this topic.

**NJCL: Okay. And then, do you think the management is actually doing enough to help the ground nurses maintain a healthier lifestyle, self-regulate a healthier lifestyle?**

P24: From management to ground? Your management refers to?

**NJCL: Like the admin, the upper management kind of people. In charge of the institution.**

P24: I would say, maybe upper management a bit too far away to really influence the ground nurses. Yeah, because they’re a few tiers up there. Ya, so the most likely people to influence is actually the manager or the clinic manager, you see. Upper management, well of course they can, you know. They are the final decision makers in whether we have these kind of services, these kind of classes in the institution. They can make that level kind of decisions. Ya, but to ground nurses directly, it seems a bit far.

**NJCL: Okay. Then let me rephrase the question; do you think the managers are doing enough for the ground nurses?**

P24: [Participant laughs here]. Place by place basis. Hmm, really depends on the kind of manager you happen to meet in the workplace. Yeah, of course we can as managers. Of course, we can always do more. Yeah, but I would say, in current settings, I won’t say healthier lifestyle is a significant part for them to emphasize.

**NJCL: Is it, managers have higher, have other priorities other than healthier lifestyle?**

P24: Mmm. Yes.

**NJCL: Okay, then. Are there, any workplace, health promotion programs in place other than the activities you mentioned earlier?**

P24: Workplace health promotion programs? Things like yearly health checks? Yeah, I think that one quite basic. I think every hospital have that. Yeah, but it's just on how how much they set the criteria. Last time in [Public Hospital name] case, there's no inclusion, exclusion, criteria as long as you want to have your blood test; the blood glucose, the cholesterol level, all these checks once a year the hospital do it free for their staff. Then further down the road, they actually put in limitations to people more than 40 years old then can sign up for that. So I don't know. That time at that time with this immediate thing. Okay, okay, you want to cut costs and things like that. Of course, priority goes to people in that age group, right? Yeah. okay loh. We do ourselves loh. Spend money on private GPs and things like that. Yeah, so that was one of it.Other health promotion things in the hospital. Let me see. Can I say like, staff benefits? So things like staff benefits where the hospital cover medical costs.I think that influences, too. What else is there? What else have you,heard of? Racking my brain here. [Participant laughs].

**NJCL: I think it's also what you mentioned regarding yearly health checks. This was quite common.**

P24: Yeah, all the hospitals would have.

**NJCL: And like what you mentioned earlier as well, like the institution, organizing activities for them. Stuff like that.**

P24: That's pretty much the same everywhere. Then. Hmm!

**NJCL: And then, do you believe like this kind of health promotion programs are enough? Sufficient for the nurses?**

P24: Hmm.. Whether sufficient or not. I don't know how to say whether sufficient, I guess you know, as humans is like, the more the better. Yeah. So it's like, how to say. At least got something it’s already not bad lah. You know. Yeah, because if let’s say more. I don’t know how much more they can do though. If I gave you a straight answer, okay lah, sufficient. Sufficient. Still better than nothing.

**NJCL: Hmm. But okay, maybe I shouldn’t have used sufficient. Do you think it’s effective enough?**

P24: Okay, that's another thing already. Whether it's effective enough. Yeah, I guess its back to the same thing. It really depends on the person, you know. How much significance, how much importance do they put into these kind of things in their life. So we really do have, we meet all kinds of people in this line of work. We do have people who are very, how to say, ‘don’t need to take care of health, must go and work and earn money’ kind, that kind. Also have.So I guess at the end of the day it still boils down to what the person wants, what the person sees as important in their life.Hmm, I mean, we can have a lot of things in a workplace, in a community, you know. Yeah. But whether or not this person will sign up for it, or go and do it or not. That's totally another story. It really depends on the person.

**NJCL: [Long pause] I think. Last question regarding this idea. How do you think you can increase a person's determination to change?**

P24: I'm still asking myself the same question. I haven't found my answer yet. [Participant laughs]. I don't have an answer to you. If I have one for myself, I will share with you. [Participant laughs].

**NJCL: And I'll just summarize what you shared with me during this last part. So regarding the role of this managers mentioned like they should have the flexibility, they should respect like the idea of work-life balance or else they will disrupt the way the ground nurses organize their time, and then it would influence them not to go for these healthier activities because of the clashing. Then, when I ask regarding like how the institution can help self-regulate a healthier lifestyle, you mentioned maybe having have more healthier food choices. Mentioned earlier already. But there are also activities organized. But for you, you feel like there’s nothing much else the institution can do to help nurses to self-regulate a healthier lifestyle. Is this correct? So far?**

P24: Yeah.

**NJCL: Then, regarding, if the managers have done enough. You mentioned that it's on a case by case basis. It really depends what kind of what type of manager you meet. But you feel like they can always do more. But you also feel like they have other priorities than having to influence a healthier lifestyle for the nurses. Is that correct?**

P24: Yeah.

**NJCL: Do you want to add anything here so far?**

P24: Hmm. No.

**NJCL: Then, when I mentioned about the workplace, health promotion programs, you mentioned the yearly health checks. Then you also mentioned like having staff benefits, which means the hospital actually cover parts of the medical cost.	Then, when I move on to the question on whether these measures are effective enough. You mentioned that it really really depends on the person and how much significance and importance they put on this kind of things for their lives. Yeah.** **It really boils down to the person that that's what you feel. And** **regarding my final question on increasing a person's determination to change, you say you also don’t really know the answer to that. Is that correct so far?**

P24: [Participant laughs]. Yes.

**NJCL: Is there anything else you would like to add?**

P24: No, nothing so far. Yeah, nothing to add so far.

**NJC: Okay, then, if anything else that you would like to speak about, that was not mentioned in the interview. Any further comments you want to share?**

P24: Hmm. no, that's about all, I guess.

**NJCL: Okay, then, this marks the end of the interview. Thank you for your time and participation.**

P24: Thank you so much.

-End of P24 transcript-
