# Supplementary material for: Self‐Regulation of Healthy Lifestyles in the Nursing Workplace: A Mixed‐Method Evaluation
Source: J Nurs Manag. 2026 Jan 15;2026:2199578. doi: 10.1155/jonm/2199578 (PMC12807584; doi:10.1155/jonm/2199578)
Supplement: Supplementary file 4 — Supporting Information 4 SM 4: An overview of the quantitative portion of the study: the questionnaire made on the Qualtrics platform that each of the participants had to do prior to being selected for the qualitative interview. [file JONM-2026-2199578-s004.docx]

**Supplementary Material 4**

An overview of the quantitative portion of the study: the questionnaire made on Qualtrics platform

1. Do you consent? (Yes/No)

Section 1: Sociodemographic details

2. Name

3. Contact number

4. Age

5. Latest weight measured (in kg)

6. Latest height measured (in m)

7. Years of experience in nursing

8. Gender

9. Ethnicity

10. Religion

11. Marital status

12. Number of children

13. Taking care of anyone else outside of work

14. Educational level

15. Current workplace

16. Position at work

17 Shifts worked

Section 2: Health habits

DIRECTIONS: This questionnaire contains 52 statements about your present way of life or personal habits. Please respond to each item as accurately as possible. Indicate the frequency with which you engage in each behaviour by selecting the appropriate option.

(Taken from standard questionnaire Health-Promoting Lifestyle Profile II by Walker et al., 1995.)

1. Discuss my problems and concerns with people close to me. N S O R

2. Choose a diet low in fat, saturated fat, and cholesterol. N S O R

3. Report any unusual signs or symptoms to a physician or other health professional. N S O R

4. Follow a planned exercise program. N S O R

5. Get enough sleep. N S O R

6. Feel I am growing and changing in positive ways. NNN S O R

7. Praise other people easily for their achievements. N S O R

8. Limit use of sugars and food containing sugar (sweets). N S O R

9. Read or watch TV programs about improving health. N S O R

10. Exercise vigorously for 20 or more minutes at least three times a week (such as N S O R

brisk walking, bicycling, aerobic dancing, using a stair climber).

11. Take some time for relaxation each day. N S O R

12. Believe that my life has purpose. N S O R

13. Maintain meaningful and fulfilling relationships with others. N S O R

14. Eat 6-11 servings of bread, cereal, rice and pasta each day. N S O R

15. Question health professionals in order to understand their instructions. N S O R

16. Take part in light to moderate physical activity (such as sustained walking N S O R

30-40 minutes 5 or more times a week).

17. Accept those things in my life which I can not change. N S O R

18. Look forward to the future. N S O R

19. Spend time with close friends. N S O R

20. Eat 2-4 servings of fruit each day. N S O R

21. Get a second opinion when I question my health care provider's advice. N S O R

22. Take part in leisure-time (recreational) physical activities (such as swimming, N S O R

dancing, bicycling).

23. Concentrate on pleasant thoughts at bedtime. N S O R

24. Feel content and at peace with myself. N S O R

25. Find it easy to show concern, love and warmth to others. N S O R

26. Eat 3-5 servings of vegetables each day. N S O R

27. Discuss my health concerns with health professionals. N S O R

28. Do stretching exercises at least 3 times per week. N S O R

29. Use specific methods to control my stress. N S O R

30. Work toward long-term goals in my life. N S O R

31. Touch and am touched by people I care about. N S O R

32. Eat 2-3 servings of milk, yogurt or cheese each day. N S O R

33. Inspect my body at least monthly for physical changes/danger signs. N S O R

34. Get exercise during usual daily activities (such as walking during lunch, using N S O R

stairs instead of elevators, parking car away from destination and walking).

35. Balance time between work and play. N S O R

36. Find each day interesting and challenging. N S O R

37. Find ways to meet my needs for intimacy. N S O R

38. Eat only 2-3 servings from the meat, poultry, fish, dried beans, eggs, and N S O R

nuts group each day.

39. Ask for information from health professionals about how to take good care N S O R

of myself.

40. Check my pulse rate when exercising. N S O R

41. Practice relaxation or meditation for 15-20 minutes daily. N S O R

42. Am aware of what is important to me in life. N S O R

43. Get support from a network of caring people. N S O R

44. Read labels to identify nutrients, fats, and sodium content in packaged food. N S O R

45. Attend educational programs on personal health care. N S O R

46. Reach my target heart rate when exercising. N S O R

47. Pace myself to prevent tiredness. N S O R

48. Feel connected with some force greater than myself. N S O R

49. Settle conflicts with others through discussion and compromise. N S O R

50. Eat breakfast. N S O R

51. Seek guidance or counseling when necessary. N S O R

52. Expose myself to new experiences and challenges. N S O R

Section 3: Stress

DIRECTIONS: The 10 questions in this scale ask you about your feelings and thoughts during the last month. In each case, you will be asked to indicate how often you felt or thought a certain way.

(Taken from standard stress assessment instrument, Perceived Stress scale by Cohen et al., 1983.)

l. In the last month, how often have you been upset because of something that happened unexpectedly?

2. In the last month, how often have you felt that you were unable to control the important things in your life?

3. In the last month, how often have you felt nervous and stressed?

4. In the last month, how often have you felt confident about your ability to handle your personal problems?

5. In the last month, how often have you felt that things were going your way?

6. In the last month, how often have you found that you could not cope with all the things that you had to do?

7. In the last month, how often have you been able to control irritations in your life?

8. In the last month, how often have you felt that you were on top of things?

9. In the last month, how often have you been angered because of things that happened that were outside of your control?

10. In the last month, how often have you felt difficulties were piling up so high that you could not overcome them?
