# Supplementary material for: Self‐Regulation of Healthy Lifestyles in the Nursing Workplace: A Mixed‐Method Evaluation
Source: J Nurs Manag. 2026 Jan 15;2026:2199578. doi: 10.1155/jonm/2199578 (PMC12807584; doi:10.1155/jonm/2199578)
Supplement: Supplementary file 6 — Supporting Information 6 SM 6: Correlation analysis results between HPLP‐II and PSS‐10 scores. [file JONM-2026-2199578-s006.docx]

Supplementary material 6

Attached here is the limited statistical analysis done using jamovi software to examine the relationship between the Health Promoting Lifestyle II and Perceived Stress Scale scores.

Correlation matrix

|  |  | Health Promoting Lifestyle Score | Perceived Stress Scale Score |
| --- | --- | --- | --- |
| Health Promoting Lifestyle Score | Spearman’s rho | - |  |
|  | df | - |  |
|  | p-value | - |  |
|  | N | - |  |
| Perceived Stress Scale Score | Spearman’s rho | -0.461*** | - |
|  | df | 65 | - |
|  | p-value | <.001 | - |
|  | N | 67 | - |

*Note.* *p<0.05 **p<.01, ***p<0.001

ρ *= -0.461*
