# Supplementary material for: Self‐Regulation of Healthy Lifestyles in the Nursing Workplace: A Mixed‐Method Evaluation
Source: J Nurs Manag. 2026 Jan 15;2026:2199578. doi: 10.1155/jonm/2199578 (PMC12807584; doi:10.1155/jonm/2199578)
Supplement: Supplementary file 7 — Supporting Information 7 SM 7: A Checklist of Mixed Methods Elements in a Submission for Advancing the Methodology of Mixed Methods Research. [file JONM-2026-2199578-s007.docx]

Supplementary Material 7

Attached here is the reporting guideline used for study

Table 1. Checklist of Mixed Methods Elements in a Submission to Advance the Methodology of Mixed Methods Research

| **Manuscript title: Self-regulation of healthy lifestyles in the nursing workplace: A mixed-method evaluation** | **Yes** | **No** | **Page number (if not applicable, indicate NA)** |
| --- | --- | --- | --- |
| **Title** | | | |
| 1. Does the title directly indicate or sufficiently allude to the methodological contribution of the study? | / |  | Cover page |
| **Abstract** | | | |
| 2. Does the abstract include an explicit statement about how the methodological contribution will advance an understanding of the chosen topic of the paper? | / |  | Abstract |
| 3. Does the abstract indicate the methodological/theoretical contribution of the study to the field of mixed methods research? | / |  | Abstract |
| **Main text of the article** | | | |
| 1. Does the main text reiterate and expand on the methodological contribution as identified in the abstract? | / |  | 2, 3 |
| 2. Does the background contain a rigorous review and citations of relevant mixed methods literature to support examining the methodological aim? | / |  | 2, 3 |
| 3. Does the paper have an explicit methodological aim? | / |  | 2, 3 |
| 4. Does the background contain an explication of the articles structure and methodological points that will be addressed in the paper? | / |  | 3 |
| 5. In the body of paper, are each of the points identified in Element 7 addressed with examples in the order specified? |  | / | NA |
| 6. In the discussion, are the explicit points made in Element 8 synthesized together to logically support the overarching methodological aim? | / |  | 19-24 |
| 7. In the discussion section, is there a specific subsection “Contribution to the Field of Mixed Methods Research” that reviews the points made and extant literature to articulate the articles novel contribution(s) to mixed methods? |  | / | NA |
| 8. Does the paper have a discussion of methodological limitations of the paper? | / |  | 24, 25 |
| 9. Does the paper have a clear writing style with sufficient headers and subheaders such that the reader can readily follow the flow and argumentation? | / |  | Headers throughout paper |
| 10. Have the references been cited according to the current American Psychological Association (APA) style? | / |  | 26-33 |
| 11. Does the paper include a figure or illustration to visualize the overarching concept of the manuscript? |  | / | NA |
| 12. Does the discussion section include recommendations for future mixed methods inquiry based on the paper’s unique contribution or limitations? | / |  | 23-25 |
| **Additional elements for empirical methodological papers Only** | | | |
| 13. Does the background of the paper include explicit statements of both the methodological purpose and purpose of the empirical study separately? | / |  | Abstract (v), 3 |
| 14. Does the description of the methods include sufficient detail about the procedures used and present these in a logical order? | / |  | 4,5 |
| 15. Does the submission include a procedural diagram of the data collection and analysis procedures as a figure? |  | / | NA |
| 16. Does the submission include a visual structure, or example, joint display, to illustrate integration and interpretation of the qualitative and quantitative findings? | / |  | 20 |
| 17. Does the discussion articulate how the use of a mixed methods approach advanced a greater understanding of the substantive topic compared with using a monomethod approach? | / |  | 23 |
